# Tunable Thiazolium Carbenes for Enantioselective Radical Three-Component Dicarbofunctionalizations

**DOI:** 10.1021/jacs.4c11947

**Published:** 2024-12-10

**Authors:** Sripati Jana, Nicolai Cramer

**Affiliations:** 1 Laboratory of Asymmetric Catalysis and Synthesis, Institute of Chemical Sciences and Engineering, Ecole Polytechnique Fédérale de Lausanne (EPFL), 1015 Lausanne, Switzerland

## Abstract

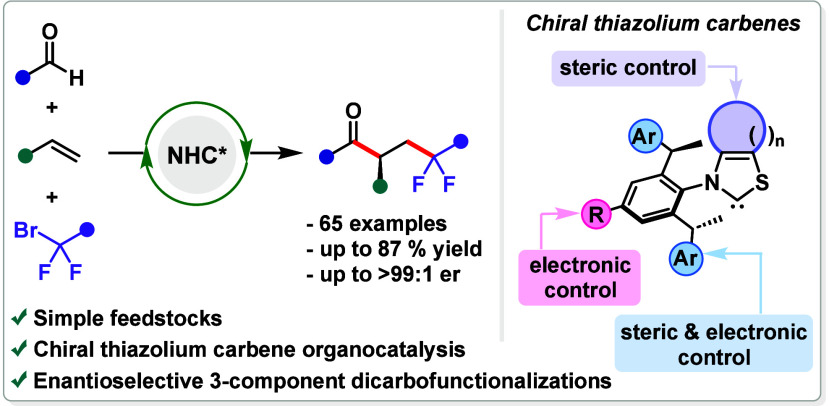

Asymmetric *N*-heterocyclic carbene (NHC) organocatalysis is a cornerstone of synthetic organic chemistry. The emerging concept of single-electron NHC catalysis broadened the scope of C–C bond-forming reactions, facilitating the synthesis of a variety of attractive racemic compounds. However, the development of effective and selective chiral NHC catalysts for asymmetric radical-mediated reactions has been challenging. In this report, we introduce a family of highly tunable chiral thiazolium carbenes with three distinct positions for broad electronic and steric modulation featuring bulky chiral flanking groups. We demonstrate the catalytic efficacy of these chiral carbenes in an enantioselective SET-type three-component acyl-difluoroalkylation of olefins using a broad range of aldehydes and difluoroalkyl bromides. This method provides straightforward access to a diverse set of β-difluoroalkylated α-chiral ketones (65 examples) with an up to 87% yield and excellent enantioselectivities of up to >99:1 er. The utility of this methodology is further outlined by enantio- and diastereoselective late-stage modifications of pharmaceutically relevant compounds and selective twofold orthogonal acyl-difluoroalkylations of linchpin reagents.

## Introduction

**Figure 1:**
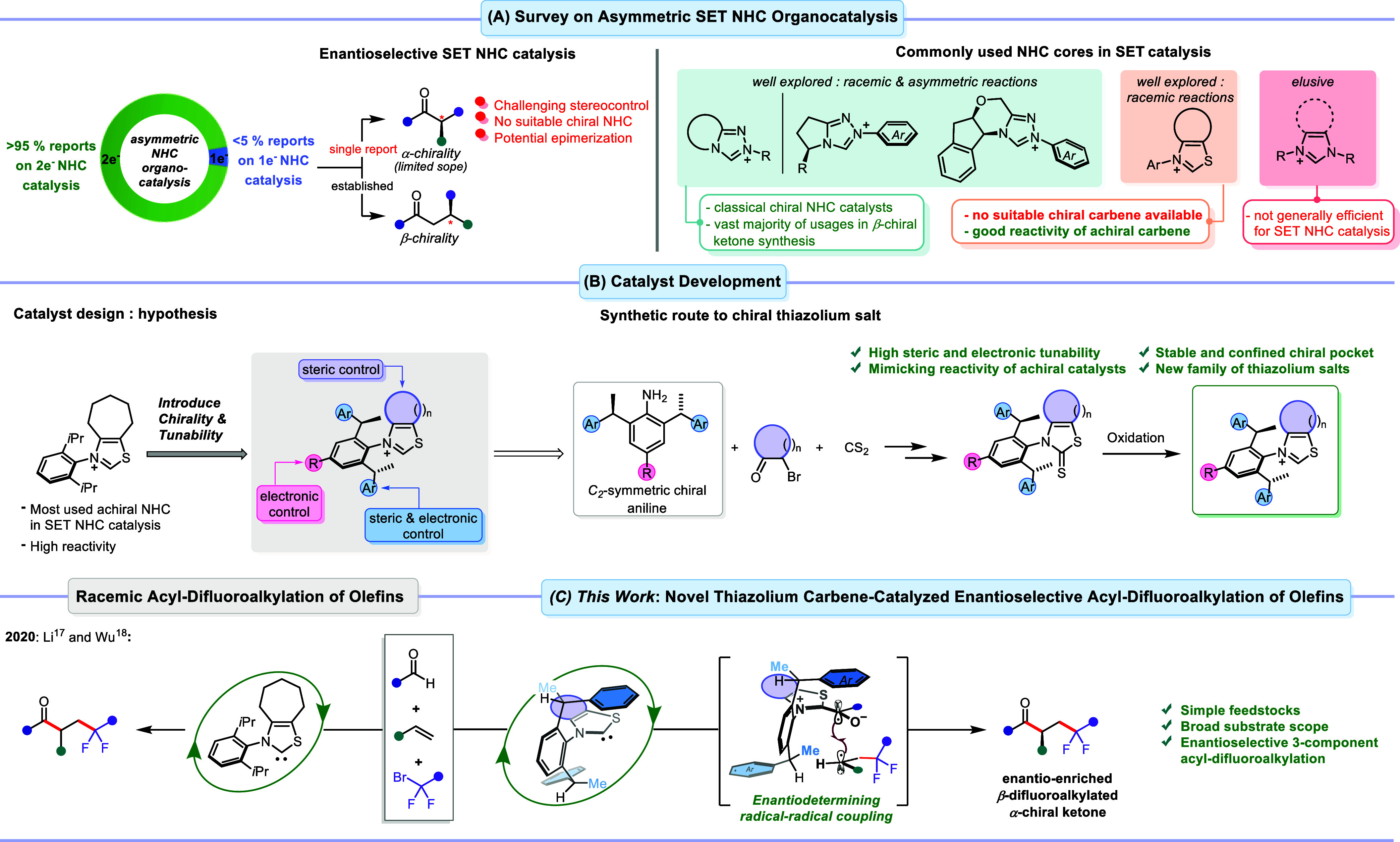
(A) Survey on asymmetric NHC organocatalysis: ionic vs radical pathways and commonly used NHC cores in SET NHC catalysis. (B) Development of a sterically and electronically tunable chiral thiazolium carbene family. (C) Application of chiral thiazolium carbene catalysts in the enantioselective three-component radical acyl-difluoroalkylation of olefins.

Since the pioneering discovery by Ukai et al. in 1943 revealing vitamin B1 (thiamine) as a catalyst for benzoin condensation,^1^ and mechanistic elucidations by Breslow in 1958,^2^*N*-heterocyclic carbene (NHC) organocatalysis has become a powerful tool for facilitating Umpolung reactions.^3^ Over the years, various activation modes driven by NHC catalysts have been developed with closed-shell mechanisms (ionic or two-electron pathways) being extensively explored. In contrast, NHC catalysis involving single-electron-transfer (SET) pathways has garnered relatively less attention. Early biological studies revealed that thiamine pyrophosphate, a cofactor in pyruvate ferredoxin oxidoreductase-catalyzed acetylation of coenzyme A, operates *via* a single-electron oxidation pathway involving the Breslow intermediate.^4,5^ In the 1990s, Fukuzumi demonstrated that Breslow intermediates possess a relatively low oxidation potential, making the oxidation of the intermediate radical cation or its deprotonated form both facile and difficult to control.^6^ Consequently, most NHC-catalyzed oxidative coupling reactions of aldehydes follow a formal electron pair transfer mechanism, resulting in the formation of electrophilic acyl azolium species.^7^ In 2008, Studer explored the SET oxidation of the Breslow intermediate to generate a radical cation, which subsequently formed an acyl azolium ion before undergoing ionic trapping. Utilizing TEMPO as a mild single-electron oxidant, their studies laid the groundwork for this process.^8^ However, achieving selective SET of NHC-bound nucleophiles to form persistent radical intermediates capable of participating in radical reactions remains a significant challenge under oxidative conditions. Recently, the single-electron transfer mechanism has been successfully harnessed to generate NHC-bound persistent ketyl radical intermediates, enabling a variety of SET-driven NHC-catalyzed transformations.^9^ This advancement has opened a new avenue for NHC-catalyzed radical reactions, significantly expanding the scope of potential applications. Despite these promising developments, most applications remain confined to achiral or racemic transformations. Concerning asymmetric catalysis, a large majority of enantioselective NHC-catalyzed transformations relies on classical ionic mechanisms and enantioselective SET NHC catalysis remains a largely underexplored area (Figure 1A).^3a,10^ While significant progress has been made in the synthesis of β-chiral carbonyl compounds,^11^ methodologies for α-chiral carbonyl compounds remain underdeveloped, largely due to challenges in controlling radical stereochemistry, limited availability of chiral NHC catalysts, and risks of epimerization. Very recently, Scheidt et al. reported moderate to good enantioselectivity in synthesizing α-chiral aliphatic ketones with Hantzsch esters and α-branched acyl imidazols.^12^ Among potential NHC cores, imidazolium carbenes have limited efficiency,^13^ whereas thiazolium^14^ and triazolium carbenes^15^ display good reactivity in racemic radical-mediated transformations albeit selectivity challenges in asymmetric reactions. While chiral triazolium-catalyzed radical-mediated asymmetric transformations are reported,^11^ the development and application of chiral thiazolium-catalyzed radical-mediated asymmetric transformations remains a substantial knowledge gap and is largely untapped. To overcome existing limitations, we aimed to develop a tunable chiral thiazolium carbene family, designed to unlock new opportunities for asymmetric radical acylation reactions, facilitating the exploration of previously inaccessible asymmetric synthesis pathways in single-electron NHC catalysis. The primary focus for the catalysts is the synthesis of β-difluoroalkylated α-chiral ketones, a compound class with significant importance across various domains due to their physical and biological properties.^16^ Despite their significance, enantioenriched β-difluoroalkylated α-chiral ketones remain exceptionally challenging to synthesize. Li^17^ and Wu^18^ independently showed an achiral thiazolium carbene-catalyzed radical acylfluoroalkylation to access racemic β-fluoroalkylated α-branched ketones and reported the inability of classical chiral NHCs to induce meaningful enantioselectivities in the process (Figure 1C, Left).

In this research, we designed a class of highly tunable chiral thiazolium carbene salts addressing the shortcomings in asymmetric catalysis, drawing inspiration from our expertise in chiral naphthyridine diimines^19^ and imidazolium IPr-type ligands,^20^ which enabled challenging enantioselective copper^21^ and nickel-catalyzed transformations.^22^ We hypothesized that the chiral *C*_*2*_-symmetric 2,6-di-(1-arylethyl)aniline platform could serve as an effective chiral element of the envisioned thiazolium carbenes (Figure 1B). The chiral 2,6-di-(1-arylethyl)aniline is a chiral surrogate mimicking the efficient 2,6-diisopropylaniline-substituted thiazolium carbene. Such a design has high tunability, offering electronic control through the substituent R on the aniline and finely adjustable steric control *via* its flanking aryl groups as well as the fused aliphatic cyclic backbone. Synthetically, the targeted chiral thiazolium salts are rapidly accessible from the chiral anilines following either the typical route for 2,6-diisopropylaniline-substituted thiazolium salts^23^ or a modified synthetic procedure for bulkier members. We present this new family of chiral thiazolium carbenes featuring three distinct points of modulation for electronic and steric tunability. Their efficacy in asymmetric catalysis is demonstrated with a highly enantioselective three-component radical dicarbofunctionalization reaction of olefins, aldehydes and difluoroalkyl bromides enabling direct access to enantioenriched β-difluoroalkylated α-chiral ketones (Figure 1C).

## Results and Discussion

### Reaction Optimization

The synthesized chiral NHC salts were evaluated as catalysts for an enantioselective three-component radical dicarbofunctionalization of olefins to produce β-difluoroalkylated α-chiral ketones (Table 1). In this respect, benzaldehyde (**1a**), styrene (**2a**) and ethyl difluorobromoacetate (**3a**) were selected as model substrates. The reactions were conducted with 5 mol% of the respective precatalyst NHC* and cesium carbonate in toluene at 60 °C. The parent library member **NHC1** acted as a promising active catalyst, yielding the desired ketone **4a** in 83% yield and a 71:29 enantiomeric ratio favoring the *S*-enantiomer (entry 1). The effect of various substitution patterns on the catalyst modulating its steric and electronic properties was tested next. An electron-donating *para*-methoxy group on the aniline aryl had a negligible impact on both the efficiency and the selectivity (entry 2). In contrast, electron-withdrawing *para*-CF_3_ slightly decreased the yield of ketone **4a** but improved its selectivity (entry 3). Glorius previously showed the relevance of varying aliphatic ring sizes on thiazolium carbene precursors and found that an increased ring size results in increased buried volume (%V_Bur_).^23^ Hypothesizing that a higher % buried volume may contribute to improved enantioselectivity, we investigated the effect of the size of the saturated backbone ring of the thiazolium carbene precursors next. Notably, **NHC4** featuring a larger eight-membered ring displayed an improved selectivity of 76:24 er (entry 4) in comparison with **NHC1** and **NHC2**. These data suggest that both an electron-withdrawing group and a sterically demanding backbone independently enhance the enantioselectivity in this transformation. Thus, we hypothesized that combining an electron-withdrawing *para*-substituent with a sterically demanding saturated backbone might be crucial for achieving high enantioselectivity. Indeed, increasing the ring size to a 12-membered saturated carbon cycle, combined with a *para*-CF_3_ group on the aniline moiety (**NHC5**), substantially improved the selectivity to 88:12 er (entry 5). We further reasoned that an increased steric demand on the chirality-bearing sidearm of the aniline motif could further enhance the enantioselectivity by creating a more confined chiral pocket for the reaction. When **NHC6** (Ar = 3,5-xylyl) was subjected to the reaction conditions, ketone **4a** was obtained with comparable yield while an improvement in enantioselectivity was observed (90:10 er). Notably, **NHC7** (Ar = 3,5-*t*Bu-C_6_H_3_) proved to be a more selective catalyst, affording product **4a** with an enhanced enantioselectivity of 93:7 er (entry 7). The improved selectivity with **NHC6** and **NHC7** are in line with our hypothesis as for both cases the steric demand is increased compared to **NHC5**. A comparative screening with well-established Rovis’s **NHC8**(24) and Sheehan’s **NHC9**(25) showed that these catalyst architectures are inefficient for this transformation, delivering **4a** in very low yield and negligible enantioselectivity (entries 8 and 9). These results are in line with the previous observations for the acyl-trifluoromethylation reaction.^17^ The best performing catalyst was next optimized with respect to other reaction parameters such as solvent, base, reaction time and temperature, catalyst loading, and reactant stoichiometries (see Supporting Information (SI) for details). Switching the solvent to methyl *tert*-butyl ether (MTBE) led to a substantial improvement in yield (79%) and enantioselectivity to 98:2 er (entry 10). Prolonging the reaction time to 24 h improved the reaction yield to 83% (entry 11). A reaction temperature of 60 °C is the balanced optimum as lower or higher temperatures cause a slower reaction (entry 12) or slight reduction in selectivity (entry 13).

**Table 1:**
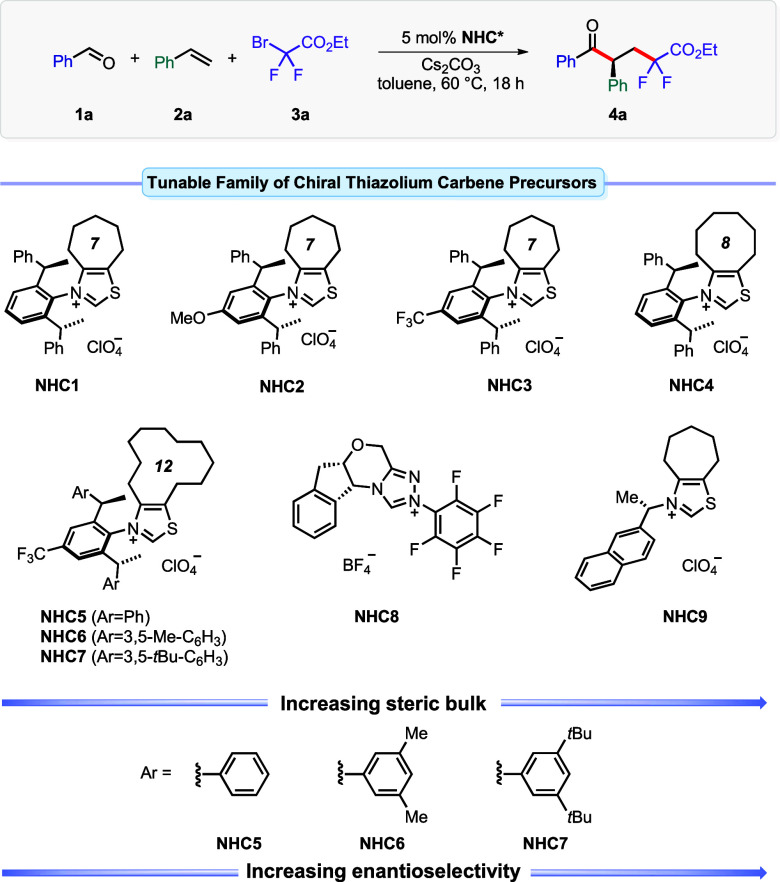
Optimization of the Chiral Thiazolium Catalyzed Asymmetric Acyl-Difluoroalkylation of Olefins

Entry^a^	NHC*	Changes	% yield^b^	er^c^
1	**NHC1**	–	83^d^	71:29
2	**NHC2**	–	81^d^	70:30
3	**NHC3**	–	76^d^	75:25
4	**NHC4**	–	79	76:24
5	**NHC5**	–	74	88:12
6	**NHC6**	–	72^d^	90:10
7	**NHC7**	–	70^d^	93:7
8	**NHC8**	–	27	56:44
9	**NHC9**	–	11	53:47
10	**NHC7**	in MTBE	79	98:2
**11**	**NHC7**	**in MTBE, 24 h**	**83^d^**	**98:2**
12	**NHC7**	in MTBE, 40 °C, 24 h	71	99:1
13	**NHC7**	in MTBE, 70 °C, 24 h	85	97.5:3.5

a Conditions: 0.1 mmol of **1a**, 0.15 mmol of **2a**, 0.2 mmol of **3a**, 5 μmol of NHC*, 0.15 mmol of Cs_2_CO_3_ in 1 mL of toluene at 60 °C for 18 h.

b ^1^H NMR yield using 1,3,5-trimethoxybenzene as an internal standard.

c Determined by chiral HPLC.

d Isolated yield.

### Substrate Scope

**Scheme 1:**
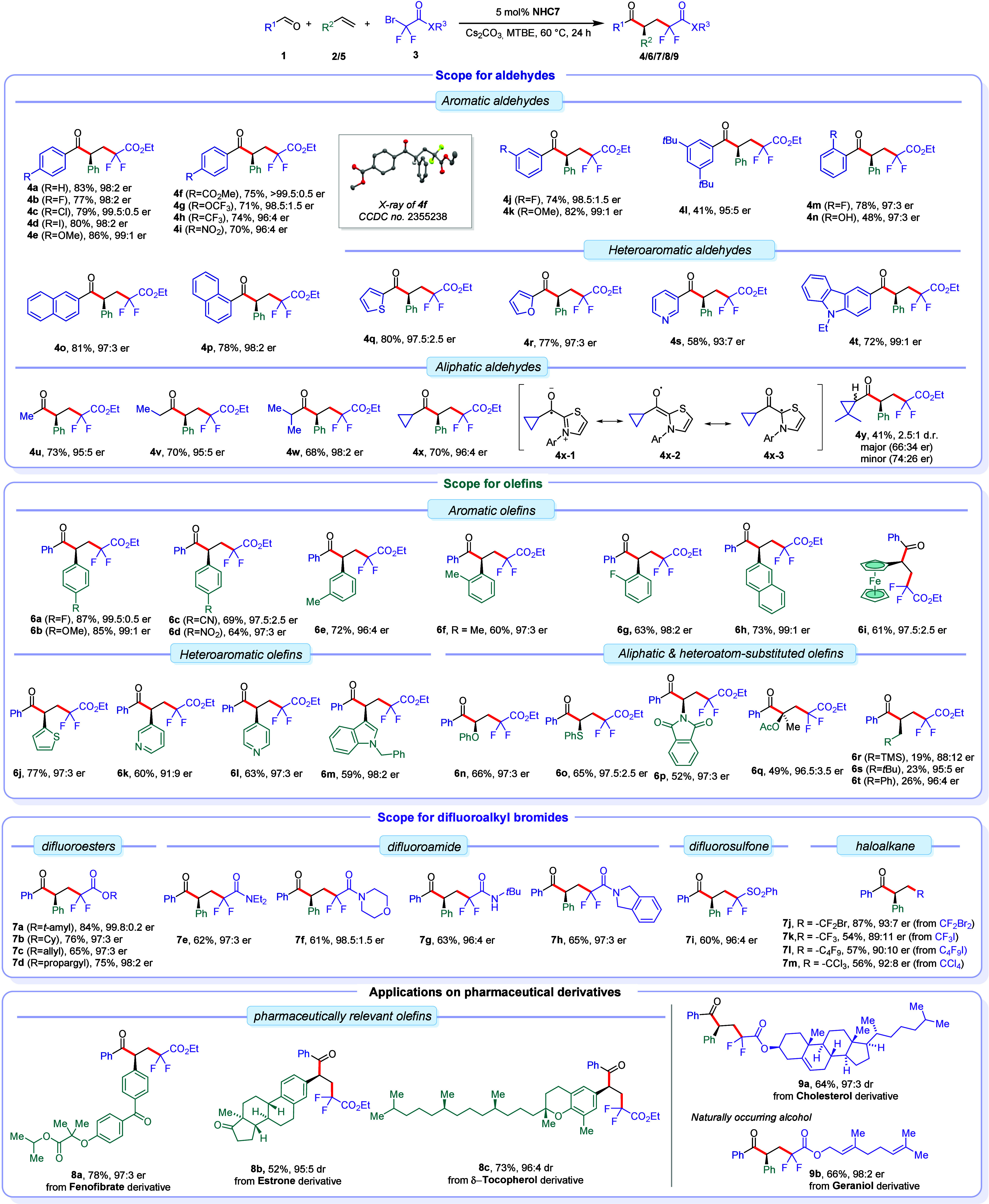
Scope for the Enantioselective Acyl-Difluoroalkylation of Olefins Conditions: 0.1 mmol of **1**, 0.15 mmol of **2**/**5**, 0.2 mmol of **3**, 5 μmol of **NHC7**, 0.15 mmol Cs_2_CO_3_ in 1 mL of MTBE at 60 °C for 24 h. Isolated yield. Enantiomeric ratio determined by chiral HPLC.·

With the optimized reaction conditions, we tested the reaction scope with various aldehydes, alkenes, and difluoroalkyl bromides (Scheme 1). A broad variety of aromatic, heteroaromatic, and aliphatic aldehydes reacted smoothly in the three-component process affording fluorinated ketones (**4a**–**4y**) in high to excellent yields and with excellent enantioselectivities. Aromatic aldehydes with electron-donating or electron-withdrawing groups at the *ortho*-, *meta*-, and *para*-positions of the aromatic ring were well compatible delivering the corresponding ketones **4a**–**4n** with excellent asymmetric inductions. The absolute configuration of the difluoroalkylated ketones was determined to be *S* by single-crystal X-ray crystallographic analysis of compound **4f**.^26^ 2-Hydroxybenzaldehyde reacted predominantly at the aldehyde site delivering product **4n** in 97:3 er while the reactive hydroxyl group remained intact despite the basic reaction conditions. 1-Naphthyl- and 2-naphthyl-substituted aldehydes underwent clean transformations yielding the corresponding ketones **4o** and **4p** in very high yields and with excellent enantioselectivities. Intriguingly, both electron-deficient and electron-rich heteroaromatic aldehydes were competent, providing corresponding ketones **4q**–**4t** in similar efficiency and selectivity. The scope of the dicarbofunctionalization could be extended to aliphatic aldehydes. For instance, aldehydes **1u**–**1x** productively engaged in the transformation with comparable reactivity and selectivity resulting in the formation of products **4u**–**4x**. Remarkably, cyclopropanecarbaldehyde exclusively produced product **4x** with an intact cyclopropyl unit. No ring-opened products that are typically generated by reactions involving cyclopropylmethyl radicals were detected. According to the mechanism^17,27^ and a critical evaluation of the Breslow enolate intermediate’s reducing ability,^28^ we suggest formation of ketyl radical intermediate **4x-1**. However, its extensive mesomeric delocalization *via***4x-2** and **4x-3** provides strong stabilization of the radical, drastically reducing its propensity for radical-clock type ring opening. Encouraged by the selective formation of compound **4x**, we extended our investigation to the more reactive racemic dimethyl-substituted cyclopropanecarboxaldehyde (**1y**). Unexpectedly, product **4y** was also formed in moderate yield, with the cyclopropane ring remaining intact. Analysis of the crude reaction mixture by both ^1^H NMR and mass spectrometry confirmed the absence of any cyclopropane ring-opening byproducts. However, **4y** was obtained with moderate diastereo- and enantioselectivity. At this stage, the underlying cause of the unsatisfactory stereoselectivities remains unclear. It could be due to a reversible ring-opening and -closing process of the cyclopropane unit or the dominance of substrate control over catalyst control in this transformation.

The behavior of various alkenes in the process was explored next. For styrene type substrates, no prominent steric and electronic substitution effect on the reaction outcome was observed. In general, the corresponding β-difluoroalkylated α-chiral ketones **6a**–**6i** were obtained in high yields with excellent enantioselectivities. Vinyl-ferrocene engaged in the acyl-difluoroalkylation and produced ketone **6i** with 97.5:2.5 er. Electron-deficient and electron-rich heteroaryl-substituted olefins, including vinyl thiophene, pyridine, and indole, were found to be compatible, providing products **6j**–**6m**. We further investigated the generality of the transformation with an array of nonconjugated alkenes under the optimized reaction conditions. Noteworthy, heteroatom (*O*-, *S*-, *N*-) substituted alkenes such as phenyl vinyl ether, phenyl vinyl sulfide, and vinyl phthalimide reacted efficiently providing access to the corresponding β-difluoroalkylated α-chiral ketones **6n**–**6p** in comparable enantioselectivities. Furthermore, the reaction of 1,1-disubstituted vinyl ester proceeded smoothly, affording functionalized ketone **6q** with a quaternary stereocenter and excellent enantioselectivity. When (1-cyclopropylvinyl)benzene was subjected to the reaction conditions, no dicarbofunctionalization occurred, but the substrate is likely consumed by radical ring-opening polymerization (see SI for details). Additionally, high levels of asymmetric induction were achieved with aliphatic terminal olefins (**6r**–**6t**), with diminished yields linked to less stabilized secondary radical intermediates. In view of the substantial synthetic value of various difluorinated compounds in modern drug discovery, we evaluated the reactivity of various difluoromethyl bromides. Different esters, amides, and sulfone substituted difluoroalkyl bromides underwent smooth single electron reduction to produce corresponding difluoroalkyl radicals for asymmetric acyldifluoroalkylation reaction of styrene **2a**. Sterically demanding *tert*-amyl ester substituted difluoromethyl bromide delivered product **7a** with an outstanding selectivity of 99.8:0.2 er. Upon subjecting allyl- and propargyl ester-substituted difluoromethyl bromides to the reaction conditions, we exclusively observed the targeted acyl-difluoroalkylation products **7c** and **7d**. Notably, no side-products *via* intramolecular radical cyclization or intermolecular radical addition to the π-bonds of the allyl or propargyl groups was detected. Secondary and tertiary amide substituted difluoromethyl bromides were compatible and afforded products **7e**–**7h** in high yield with estimable enantioselectivities. Notably, *tert*-butyl amide substituted difluoromethyl bromide reacted as expected and did not undergo any side reaction at the free NH position under the basic reaction conditions. Phenyl sulfonyl-substituted difluoromethyl bromide also reacted efficiently, yielding **7i** in 60% yield and with 96:4 er. Dibromodifluoromethane reacted very selectively a single time leading to acyl-bromodifluoromethylation of styrene. Hence, fluorinated chiral ketone **7j** can serve as an elaborated difluoroalkyl radical precursor for further transformations. Trifluoroiodomethane was tested as the substrate, yielding trifluoromethylated ketone **7k** in moderate yield and slightly reduced enantioselectivity. When perfluorobutyl iodide was used, ketone **7l** was obtained with a similar performance. Carbon tetrachloride proved to be compatible under the reaction conditions, yielding **7m** with excellent enantioselectivity and good yield. Other radical precursors such as redox-active esters and Katritzky salts were tested next (see SI for details). An applicability of the methodology for late-stage modifications, was illustrated with enantioselective acyl-difluoroalkylation of pharmaceutically relevant molecules.^29^ For instance, vinyl derivative **5a** of hyperlipemia drug fenofibrate,^30^ productively engaged in this reaction, giving acyl-difluoroalkylated fenofibrate **8a** in 78% yield and 97:3 er. Moreover, olefinic derivatives of estrone **5b** and δ-tocopherol **5c**, both featuring multiple chiral centers, were converted to the corresponding products **8b** and **8c** in high yields and excellent diastereoselectivities. Furthermore, geraniol- and cholesterol-ester-derived difluoromethyl bromides reacted smoothly to ketones **9a** and **9b** in similar selectivities and yields. The excellent diastereoselectivities of products **8b**, **8c**, and **9a** evidence that the enantio-determining C-C bond-forming event of the radical–radical recombination is under full catalyst control.

### Demonstration of Practicability

**Scheme 2:**
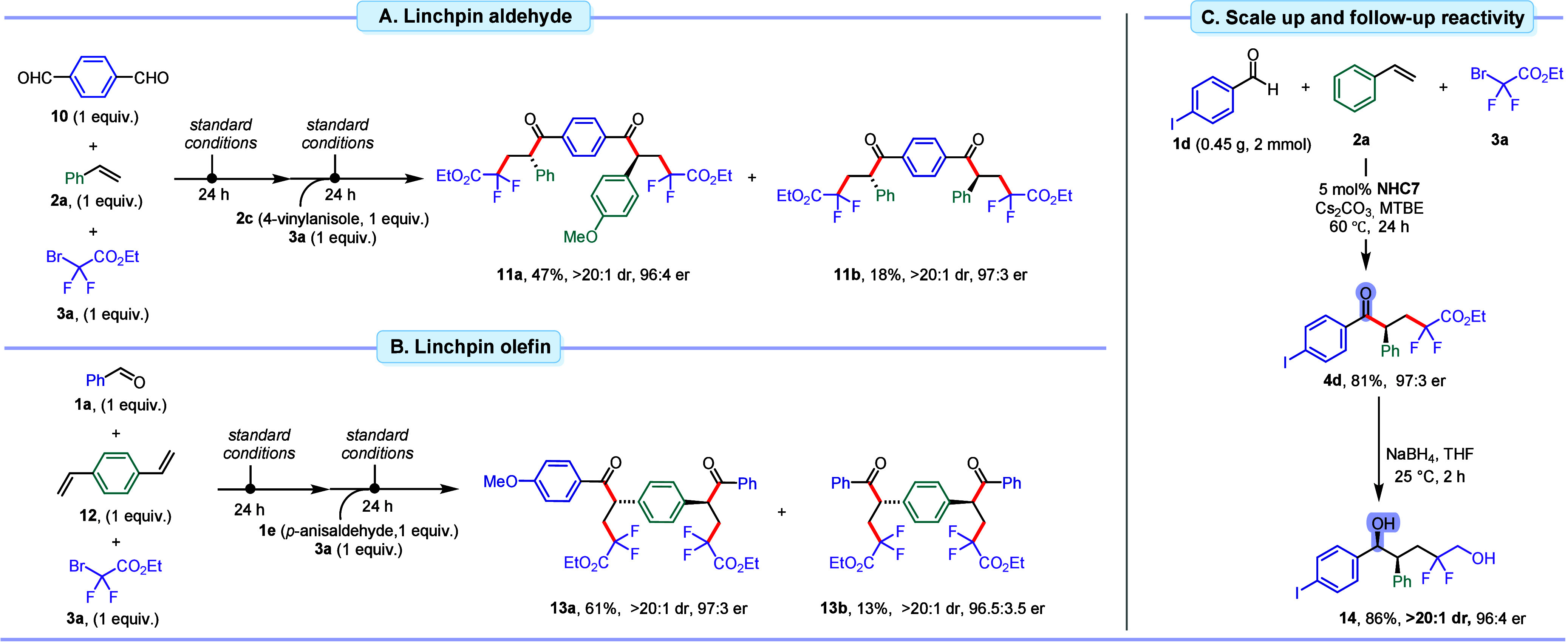
Demonstration of Practicability in Double Reactivity and Follow-up Reactions One-pot double acyl-difluoroalkylation with a linchpin aldehyde. One-pot double acyl-difluoroalkylation with a linchpin olefin. Scale-up and reduction of ketone **4d**.

Next, the synthetic utility of enantioselective three-component radical acyl-difluoroalkylations was further expanded by investigating their applicability with double-functionalized linchpin reactants. In the first setup, *bis*-aldehyde **10** was subjected to a stepwise one-pot procedure geared to install two different benzyl radical intermediates on each aldehyde location (Scheme 2A). Specifically, **10**, **3a** and 1 equiv of styrene were reacted for 24 h under standard conditions. Subsequently, 4-vinylanisole **2c** and another equivalent of ethyl difluorobromoacetate **3a** were added to the reaction mixture and allowed to react for an additional 24 h. This approach provided diketone **11a** with two different α-aryl groups in 47% yield and excellent enantio- and diastereoselectivity. Additionally, *C*_2_-symmetric diketone **11b** was observed as a minor product. In a second setup, 1,4-divinylbenzene **12** was employed as the linchpin reagent (Scheme 2B). Following a similar protocol, two different aldehydes were added to the reaction mixture along with other components. Differentially substituted product **13a** was isolated in 61% yield with excellent enantio- and diastereoselectivity. *C*_2_-symmetric diketone **13b** was produced in a 13% yield. The reaction scales well, and both yield and selectivity are virtually identical for the formation of **4d** on a 20-fold scale-up at 2 mmol (81% yield, 97:3 er) (Scheme 2C). Without erosion of optical purity, ketone **4d** was diastereoselectively reduced with NaBH_4_ to corresponding diol **14** in 86% yield and >20:1 dr.

### Proposed Mechanism

**Figure 2:**
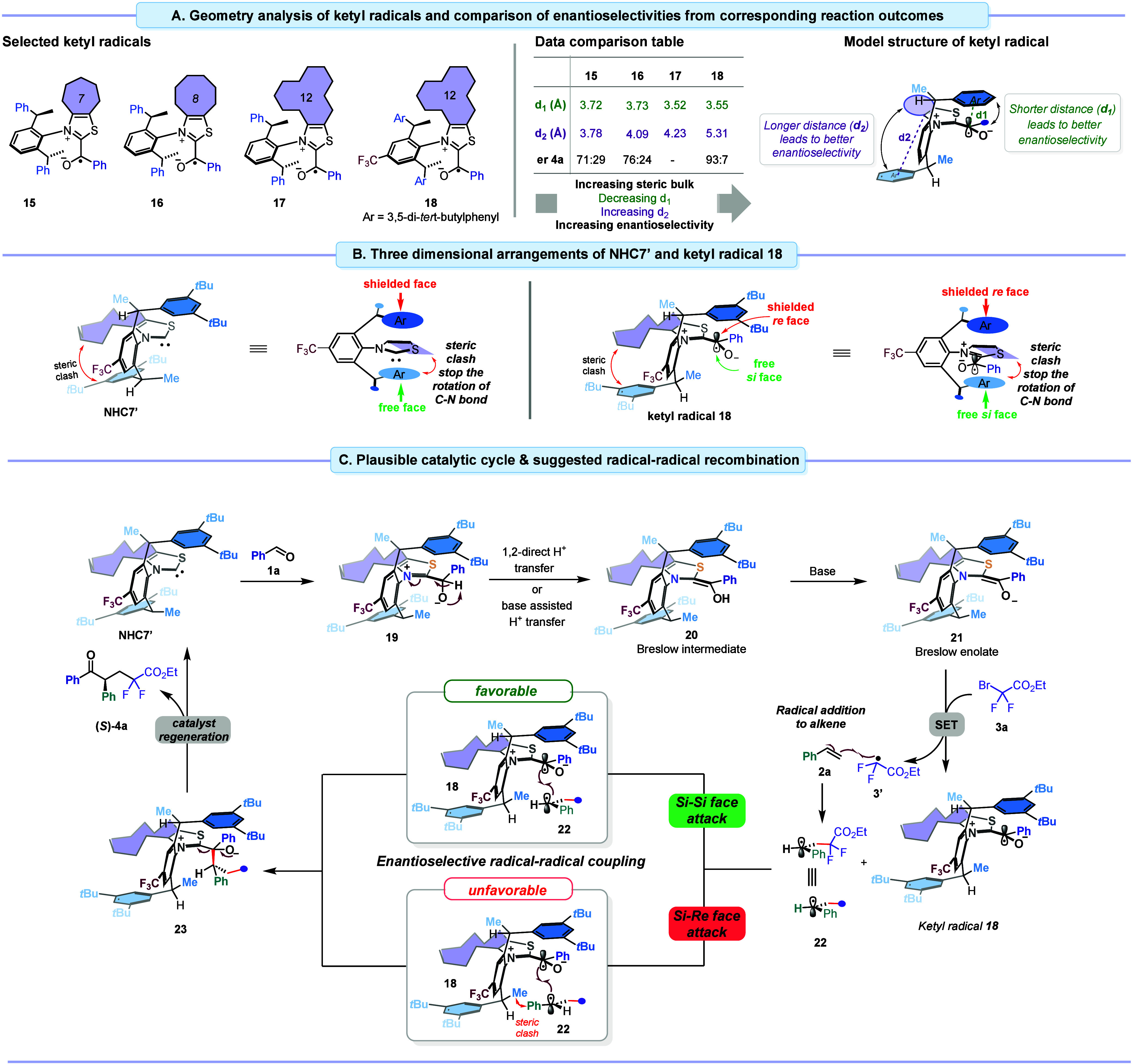
(A) Optimized geometry analysis of ketyl radicals (**15**–**18**) and comparison of enantioselectivities in corresponding reaction outcomes. (B) Three-dimensional arrangements of **NHC7′** and its corresponding ketyl radical intermediate **18**. (C) Plausible catalytic cycle and suggested radical–radical recombination.

Based on literature precedents,^12,17,27,28^ ketyl radicals serve as key intermediates in the enantio-determining radical–radical coupling events. To enhance our understanding of the origins of enantioselectivity by three-dimensional arrangements and to link structural data with experimentally observed enantioselectivity, we performed geometry optimization of selected NHCs and their corresponding ketyl radicals (see SI for details). The structural analysis of the optimized geometries revealed a trend: as the size of the aliphatic backbone increases from 7-membered to 12-membered rings, the distance (*d*_1_) between the centroid of the top arene ring of the chiral aniline motif and the active carbon atom of the ketyl radical decreases, while the distance (*d*_2_) between the centroid of the bottom arene ring and the C=C bond within the thiazole scaffold of the ketyl radical increases (Figure 2A). This trend matches an increase in enantioselectivity (Figure 2A, Comparison table). As the size of the backbone ring increases, the lower arene ring moves away from the aliphatic ring, while the top arene ring orients closer to the active carbon of the ketyl radical intermediate to achieve conformational stability. This arrangement fosters a stable and confined chiral environment and restricts the C–N rotation more with increasing ring size, which is crucial for the enantio-determining radical–radical recombination. Interestingly, the optimized geometry of the bulky ketyl radical intermediate **18** derived from the active carbene species **NHC7′** displays the longest distance (*d*_2_) of 5.31 Å and a comparable distance (*d*_1_) to radical **17**, thus providing a more confined chiral pocket compared to other radical intermediates and achieving high enantioselectivity with this NHC. The structural analysis of **18** indicates that the top *re*-face of **18** is shielded by the large 3,5-di-*tert*-butylphenyl sidearm, while the bottom *si*-face is exposed (Figure 2B). With this insight into the spatial arrangements of **NHC7′** and radical intermediate **18**, we can articulate a putative catalytic cycle and asymmetric induction model (Figure 2C). The catalytic cycle is initiated by addition of **NHC7′** onto benzaldehyde **1a** forming zwitterionic intermediate **19**, which in turn undergoes a base-assisted or direct 1,2-proton transfer to Breslow intermediate **20**. Deprotonation of **20** by the base generates the corresponding Breslow enolate **21** which has strong reducing properties. A single electron transfer from enolate **21** to difluorobromoacetate **3a** produces a reactive difluoroalkyl radical **3′** and a persistent ketyl radical intermediate **18**. Rapid addition of difluoroalkyl radical **3′** to styrene **2a** forms a difluoroalkylated benzylic 2° radical **22**. The subsequent C–C bond-forming radical–radical coupling between **18** and **22** is the enantio- and likely rate-determining step.^12^ With both radicals **18** and **22** being prochiral, there are four unique coupling approaches (*re-si*, *re-re*, *si-si*, and *si-re*) possible. As discussed, the *re*-face of ketyl radical **18** is heavily shielded, making only its *si*-face available for any productive C–C bond-forming event. Thus, radical **22** could approach in either a *si-si* or *si-re* fashion. Between these feasible coupling patterns, the *si-re* approach is disfavored by a steric clash between a backbone methyl group of **18** and the phenyl group of **22**. Conversely, the *si-si* face attack lacks such clash and is favored leading to the formation of the tetrahedral intermediate **23**. A subsequent facile extrusion of **NHC7′** delivers the *S*-enantiomer of ketone **4a** and closes the catalytic cycle.

## Conclusion

In conclusion, we introduced a novel class of chiral thiazolium carbene precursors and demonstrated their catalytic efficacy in an enantioselective three-component radical dicarbofunctionalization reaction. This family of chiral thiazolium carbenes displays high tunability, with three distinctly exploitable positions for electronic and steric modulation. The optimized thiazolium carbene catalyst exhibits high efficiency and excellent enantioselectivities in the first example of a chiral NHC-organocatalyzed radical acyl-difluoroalkylation of olefins with a range of aldehydes and various substituted difluoromethyl bromides. The reaction conditions are mild, providing a straightforward route to a wide variety of valuable enantioenriched α-chiral β-difluoroalkylated ketones with very high yields and enantioselectivities. The utility of this methodology is further outlined by enantio- and diastereoselective late-stage modifications of pharmaceutically relevant compounds and selective twofold orthogonal acyl-difluoroalkylations of linchpin reagents. Further investigations exploring the applicability of this chiral thiazolium carbene class for asymmetric SET NHC-catalyzed organic transformations are ongoing.

## Supporting Information Available

Experimental procedures, X-ray crystallography data of **4f** and spectroscopic data of all new compounds as well as computational details are available in Supporting Information. (PDF)
