# Supplementary material for: Tunable Thiazolium Carbenes for Enantioselective Radical Three-Component Dicarbofunctionalizations
Source: J Am Chem Soc. 2024 Dec 10;146(51):35199–207. doi: 10.1021/jacs.4c11947 (PMC11673126; doi:10.1021/jacs.4c11947)

Supporting Information

Tunable Thiazolium Carbenes for Enantioselective Radical Three-Component Dicarbofunctionalizations

Sripati Jana,[1] Nicolai Cramer[1*]

[1]Laboratory of Asymmetric Catalysis and Synthesis, Institute of Chemical Sciences and Engineering, Ecole Polytechnique Fédérale de Lausanne (EPFL), 1015 Lausanne, Switzerland.

*Email: nicolai.cramer@epfl.ch

Table of content

General information S2
Synthesis of chiral thiazolium salts S3
Procedure for the Synthesis of 2,6-bis(1-aryllvinyl)aniline derivatives (GP 1) S3
Procedure for the Synthesis of 2,6-bis(1-aryllvinyl)aniline derivatives (GP 2) S4
procedure for the asymmetric hydrogenation of alkenyl anilines (GP 3) S5
General procedure for the synthesis of chiral thiazolium salts (GP 4) S7
Modified procedure for the synthesis of chiral thiazolium salts (GP 5) S8
Physical data for chiral thiazolium salts S9
Synthesis of starting materials S14
Synthesis of 2,2-dimethylcyclopropane-1-carbaldehyde (1y) S14
General Procedure for the Synthesis of α-bromo-α,α-difluoroesters (GP 6) S14
General procedure for the synthesis of α-bromo-α,α-difluoroacetamides (GP 7) S16
Procedure for the Synthesis of redox-active esters S18
Proceudre for the synthesis of Katritzky salt S19
Optimization of the chiral thiazolium carbene catalyzed asymmetric acyl-difluoroalkylation of olefins S22
Table S1: Catalyst screening S22
Table S2: Solvent screening S23
Table S3: Base screening S23
Table S4: Time screening S24
Table S5: Temperature screening S24
Table S6: Stoichiometry screening S24
Table S7: Concentration screening S25
Catalysis procedures S25
Procedure for the thiazolium carbene-catalyzed three-component acyl-difluoroalkylation reaction (GP 8) S25
Procedure for the chiral thiazolium carbene-catalyzed asymmetric acyl-difluoroalkylation of olefins (GP 9) S25
Physical data S26
Reaction with linchpin aldehyde S94
Reaction with linchpin olefin S97
Scale up experiment S99
Supplementary Experiments for Revision S101
X-ray Christallographic data S105
Geometry optimization of the active carbenes and the ketyl radical intermediates S113
Optimized geometry analysis of ketyl radicals and comparison of enantioselectivities S113
Three-dimensional arrangements of NHC7' and its corresponding ketyl radical intermediate 18 S114
Stationary state coordinates S115
References S132
NMR spectra S134

General information

All reactions were carried out under a nitrogen atmosphere in flame-dried glassware with magnetic stirring, either inside a MBRAUN glove box workstation or using Schlenk techniques, unless otherwise indicated. Dichloromethane, diethyl ether, toluene, acetonitrile, and tetrahydrofuran were purified using an Innovative Technology Solvent Delivery System, degassed via the freeze-pump-thaw technique, and stored over 4 Å molecular sieves in a nitrogen-filled glove box. Reagents and solvents were purchased from Sigma Aldrich/Merck, Acros, Alfa Aesar, Abcr, or TCI, and used as received. Ethyl acetate (EtOAc), dichloromethane (DCM), and *n*-pentane, used for filtration, transfers, chromatography, and recrystallizations, were purchased from commercial sources and distilled before use. Methyl *tert*-butyl ether (MTBE), used for enantioselective catalysis, was purchased from Thermo Scientific Chemicals and stored directly in the glove box.

Flash chromatography was performed with Silicycle silica gel 60 (40-60 µm, 230–400 mesh). Analytical thin-layer chromatography (TLC) was performed with commercial glass plates coated with 0.25 mm silica gel (E. Merck, Kieselgel 60 F254). Compounds were visualized either under UV light at 254 nm or by dipping the plates in an aqueous potassium permanganate solution followed by heating.

Proton nuclear magnetic resonance (^{1}H NMR) spectra were acquired on a Bruker AVANCE NEO-400 (400 MHz), AVANCE III-400 (400 MHz), or AVANCE II-800 (800 MHz) spectrometer at 298 K unless otherwise noted. Chemical shifts (δ) are reported in parts per million (ppm) relative to the residual signal of deuterated chloroform ($CDCl_3$, δ 7.26 ppm). Splitting patterns are designated as follows: bs (broad singlet), s (singlet), d (doublet), t (triplet), q (quartet), hept (heptet), m (multiplet), and br (broad). Proton-decoupled carbon-13 nuclear magnetic resonance ($^{13}C\{^1H\}$ NMR) spectra were acquired on a Bruker AVANCE III-400 (101 MHz) or AVANCE II-800 (201 MHz) spectrometer at 298 K unless otherwise noted. The assignment of primary (CH_3), secondary (CH_2), tertiary (CH), and quaternary (Cquat) carbon atoms was made using DEPT-135 spectra. Chemical shifts are reported relative to $CDCl_3$ (δ 77.16 ppm). Fluorine-19 NMR (^{19}F NMR) spectra were acquired on a Bruker AVANCE III-400 (376 MHz) spectrometer at 298 K unless otherwise noted.

Geometry optimization were performed with the Gaussian 09 program. Structure optimization was performed using either B3LYP or (U)B3LYP functional, and the def2svp basis set for all atoms.

Infrared (IR) spectra were recorded on an Alpha-P Bruker FT-IR spectrometer, with absorbance frequencies reported in reciprocal centimeters (cm^{-1}).

High-resolution mass spectrometry (HRMS) data were acquired on an Agilent LC-MS TOF (Multimode: ESI + APCI) or an LTQ Orbitrap FTMS instrument (LTQ Orbitrap Elite FTMS, Thermo Scientific, Bremen, Germany) equipped with an Ion Max APPI ionization source with a VUV Kr lamp (Syagen, CA, USA). Samples were diluted in DCM or THF and directly infused into the mass spectrometer. FT-MS spectra were recorded in the 100–1000 m/z range in positive mode with a resolution set to 120,000. HRMS analyses were performed by Dr. L. Menin's team at the mass spectrometry platform of ISIC at EPF Lausanne.

Optical rotations were measured on a Polartronic M polarimeter using a 10.0 cm cell with a Na 589 nm filter.

The enantiomeric ratio of compounds was measured on an Agilent HPLC 1260 Infinity or Shimadzu Prominence UFLC XR system using a chiral CHIRALPAK column.

X-ray analysis was performed by Dr. R. Scopelliti at EPF Lausanne.

Synthesis of chiral thiazolium salts

General procedure for the Synthesis of 2,6-bis(1-aryllvinyl)aniline derivatives (GP 1)

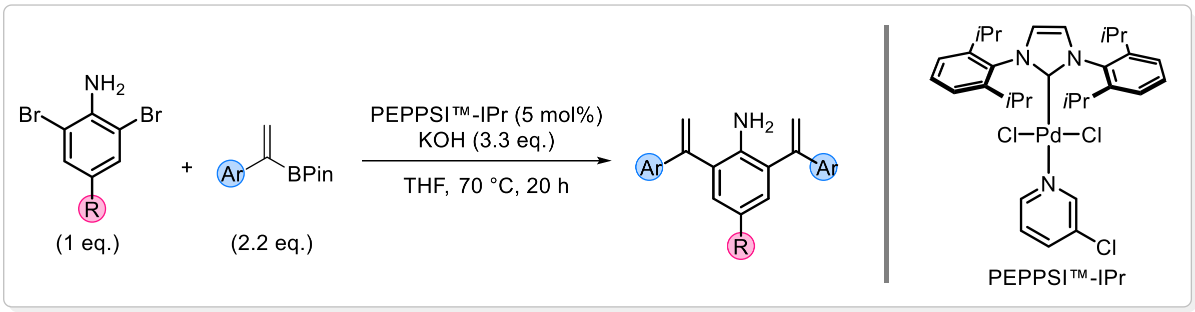

According to the previously reported literature:[1,2] In a Schlenk tube, 2,6-dibromo-substituted aniline (1 eq.), PEPPSI™-IPr (5 mol%), and powdered KOH (2.2 eq.) were dissolved in THF (16.5 mL/mmol). A solution of boronate (2.2 eq.) in THF (3.5 mL/mmol) was then added to this mixture. The resulting mixture was degassed using the freeze-pump-thaw method and then stirred for 12 hours at 70 °C. After this, an additional portion of KOH (1.1 eq.) was added, and the mixture was heated for another 8 hours. The reaction mixture was passed through a silica pad, washed with EtOAc, and concentrated under reduced pressure. The residue was then purified by silica gel column chromatography (*n*-pentane : EtOAc) to obtain the desired product.

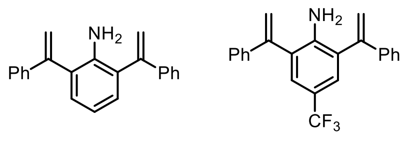

aa **ab**

Compounds **aa** and **ab** have been previously reported in the literature.[2] They were synthesized according to the general procedure 1 (**GP 1**). The spectral data are in accordance with previous literature.

2,6-Bis(1-(3,5-dimethylphenyl)vinyl)-4-(trifluoromethyl)aniline (ac)

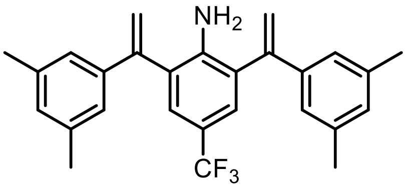

The title compound was synthesized on a 2.0 mmol scale according to the general procedure 1 (GP 1) and was obtained as a viscous yellow oil after silica gel column chromatography (*n*-pentane : ethylacetate 60:1). Yield: 69% (581 mg). $\mathbf{R_f}$ = 0.35 (*n*-pentane : EtOAc 60:1).
^{1}H NMR (400 MHz, Chloroform-*d*): δ = 7.40 (s, 2H), 6.97 – 6.93 (m, 6H), 5.80 (d, *J* = 1.4 Hz, 2H), 5.37 (d, *J* = 1.4 Hz, 2H), 3.12 (bs, 2H), 2.28 (s, 12H) ppm.
^{13}C NMR (101 MHz, Chloroform-*d*): δ = 146.5, 144.6, 138.7, 138.2, 130.1, 127.1 (q, *J* = 3.7 Hz), 127.1, 124.9 (d, *J* = 270.8 Hz), 124.2, 119.1 (q, *J* = 32.5 Hz), 117.0, 21.3 ppm.
^{19}F NMR (376 MHz, Chloroform-*d*): δ = -60.74 ppm.
HRMS (ESI/QTOF): *m/z*: $[M + H]^+$ Calcd for $C_{27}H_{27}F_3N^+$ 422.2090; Found 422.2085.
IR (ATR): 1618, 1597, 1303, 1111, 900, 852 cms^{-1}.

2,6-Bis(1-(3,5-di-*tert*-butylphenyl)vinyl)-4-(trifluoromethyl)aniline (ad)

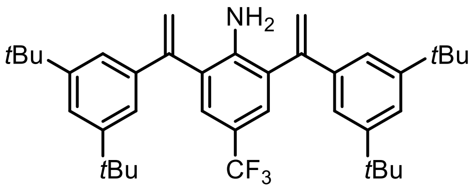

The title compound was synthesized on a 2.0 mmol scale according to the general procedure 1 (GP 1) and was obtained as a viscous yellow oil after silica gel column chromatography (*n*-pentane : ethylacetate 80:1). Yield: 57% (671 mg). **R$_f$** = 0.50 (*n*-pentane : EtOAc 80:1).
^{1}H NMR (400 MHz, Chloroform-*d*): δ = 7.38 (t, *J* = 1.8 Hz, 2H), 7.35 – 7.34 (m, 2H), 7.21 (d, *J* = 1.8 Hz, 4H), 5.85 (d, *J* = 1.3 Hz, 2H), 5.38 (d, *J* = 1.4 Hz, 2H), 5.04 (bs, 2H), 1.28 (s, 36H) ppm.
^{13}C NMR (101 MHz, Chloroform-*d*): δ = 151.0, 147.0, 144.8, 138.2, 127.01, 127.00 (q, *J* = 4.0 Hz), 124.9 (q, *J* = 270.8 Hz), 122.5, 120.9, 118.8 (q, *J* = 32.4 Hz), 116.4, 34.8, 31.4 ppm.
^{19}F NMR (376 MHz, Chloroform-*d*): δ = -60.95 ppm.
HRMS (ESI/QTOF): *m/z*: [M + H]$^+$ Calcd for $C_{39}H_{51}F_3N^+$ 590.3968; Found 590.3964.
IR (ATR): 2962, 1619, 1590, 1361, 1305, 1114, 879 cm^{-1}.

General procedure for the Synthesis of 2,6-bis(1-aryllvinyl)aniline derivatives (GP 2)

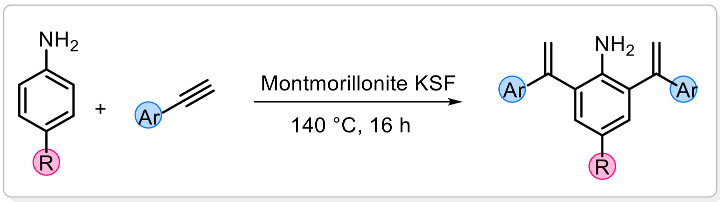

According to the previously reported literature:[3] A 25 mL microwave tube equipped with a stirring bar was charged with *para*-substituted aniline (1 eq.), montmorillonite KSF (100 mg/mmol), and phenylacetylene (2.1 eq.) under a nitrogen atmosphere. The tube was heated to 150 °C and stirred vigorously for 16 hours. After cooling to room temperature, the reaction mixture was diluted with EtOAc and filtered. The solid residue was then washed with EtOAc. The solvent was removed from the filtrate under reduced pressure, and the resulting residue was purified by silica gel column chromatography (*n*-pentane: ethylacetate) to obtain the desired product.

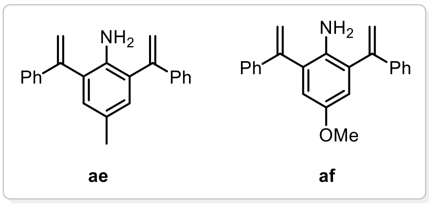

Compounds **ae** and **af** have been previously reported in the literature.[3] They were synthesized according to the general procedure 2 (**GP 2**). The spectral data are in accordance with previous literature.[3]

General procedure for the asymmetric hydrogenation of alkenyl anilines (GP 3)

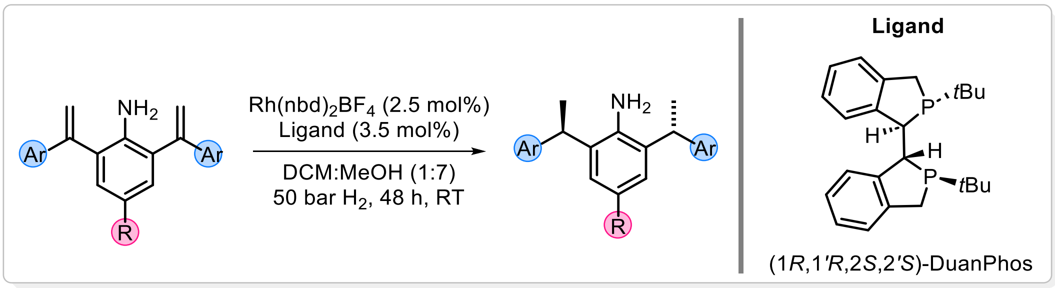

C2-Symmetric chiral anilines were synthesized following a reported procedure.[1,2] In a microwave tube, $[Rh(nbd)_2]BF_4$ (2.5 mol%) and (1*R*,1'*R*,2*S*,2'*S*)-DuanPhos (3.5 mol%) were dissolved in DCM under a nitrogen atmosphere and stirred for 20 minutes. A solution of alkenyl aniline (1 eq.) in DCM was then added, followed by the addition of MeOH (ratio MeOH/DCM: >7/1, 0.25 M). The microwave tube was transferred to an autoclave, and the reactor was purged three times with H_2. The reactor was then pressurized to 50 bar H_2, and the mixture was stirred at ambient temperature for 48 hours. The reaction mixture was concentrated, and the resulting residue was purified by silica gel column chromatography (*n*-pentane: ethylacetate) to obtain the desired chiral aniline.

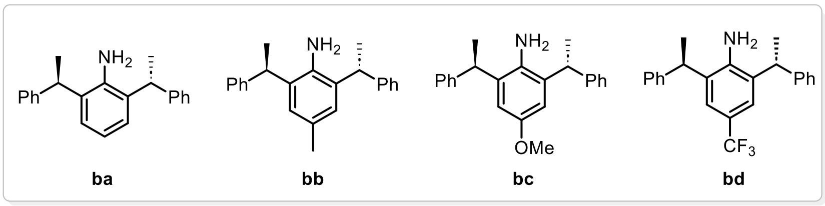

Compounds **ba** – **bd** have been previously reported in the literature.[1,2] They were synthesized according to the general procedure 3 (**GP 3**). The spectral data are in accordance with previous literature.[2]

2,6-Bis((*R*)-1-(3,5-dimethylphenyl)ethyl)-4-(trifluoromethyl)aniline (be)

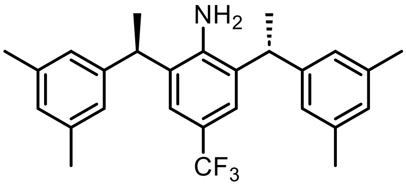

The title compound was synthesized on a 3.0 mmol scale according to the general procedure 3 (**GP 3**) and was obtained as a viscous pink oil after silica gel column chromatography (*n*-pentane : ethylacetate 90:1). Yield: 89% (1.1 g). $\mathbf{R_f}$ = 0.45 (*n*-pentane : ethylacetate 90:1).

^{1}H NMR (400 MHz, Chloroform-*d*): δ = 7.51 (s, 2H), 6.82 (s, 2H), 6.70 (s, 4H), 3.86 (q, *J* = 7.1 Hz, 2H), 3.72 (bs, 2H), 2.24 (s, 12H), 1.60 (d, *J* = 7.1 Hz, 6H) ppm.

^{13}C NMR (101 MHz, Chloroform-*d*): δ = 145.6, 145.0, 138.5, 129.1, 128.5, 125.6 (q, *J* = 270.9 Hz), 125.1, 122.5 (q, *J* = 4.0 Hz), 119.0 (q, *J* = 31.9 Hz), 40.7, 22.6, 21.4 ppm.

^{19}F NMR (376 MHz, Chloroform-*d*): δ = -60.41 ppm.

HRMS (ESI/QTOF): *m/z*: $[M + H]^+$ Calcd for $C_{27}H_{31}F_3N^+$ 426.2403; Found 426.2405.

IR (ATR): 2968, 1627, 1452, 1328,1150, 1108, 781 cm^{-1}.

$[\alpha]_D^{20}$ = +10.6 (c = 0.5, $CHCl_3$).

Chiral HPLC: (Chiralpak IB, 0.2 % *i*PrOH/hexane, 1.0 mL/min, 210 nm): t_R (*R*,*R*) = 6.09 min, >99.9:0.1 er.

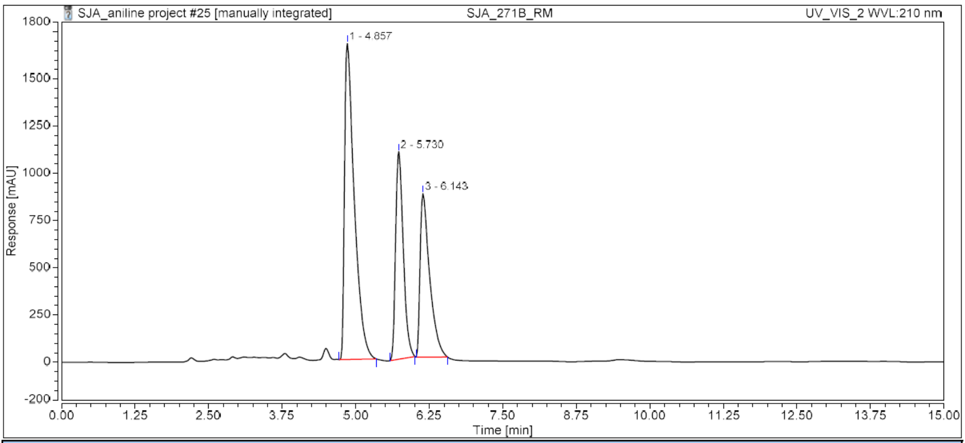

Integration Results

No.	Peak Name	Retention Time min	Area mAU*min	Height mAU	Relative Area %	Relative Height %	Amount n.a.
1		4.857	322.538	1672.144	49.62	45.98	n.a.
2		5.730	165.319	1098.735	25.43	30.21	n.a.
3		6.143	162.139	865.872	24.94	23.81	n.a.
**Total:**			**649.996**	**3636.751**	**100.00**	**100.00**	

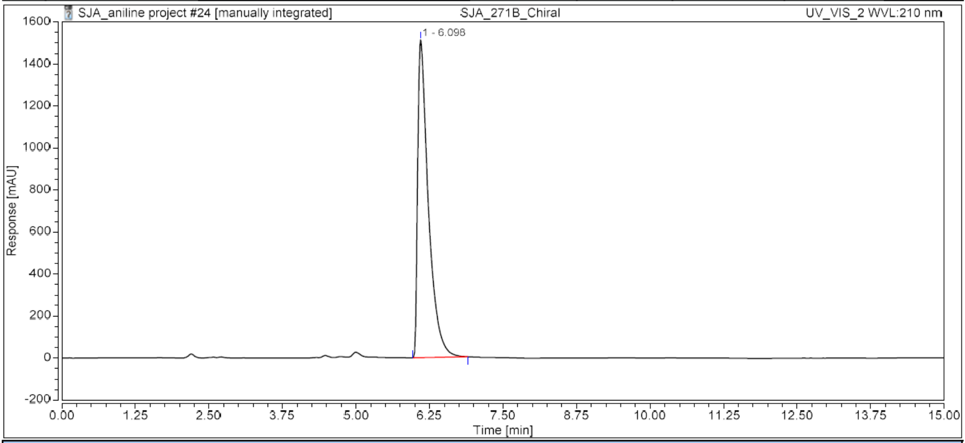

Integration Results

No.	Peak Name	Retention Time min	Area mAU*min	Height mAU	Relative Area %	Relative Height %	Amount n.a.
1		6.098	318.282	1513.270	100.00	100.00	n.a.
**Total:**			**318.282**	**1513.270**	**100.00**	**100.00**	

2,6-Bis((*R*)-1-(3,5-di-*tert*-butylphenyl)ethyl)-4-(trifluoromethyl)aniline (bf)

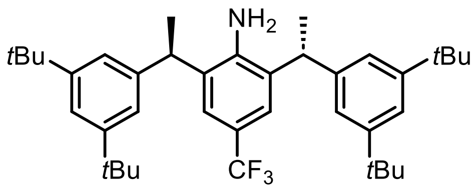

The title compound was synthesized on a 3.0 mmol scale according to the general procedure 3 (**GP 3**) and was obtained as a viscous colorless oil after silica gel column chromatography (*n*-pentane : ethylacetate 90:1). Yield: 92% (1.6 g); >99.9% ee. $\mathbf{R_f}$ = 0.48 (*n*-pentane : ethylacetate 90:1).

^{1}H NMR (400 MHz, Chloroform-*d*): δ = 7.45 (s, 2H), 7.25 (t, *J* = 1.8 Hz, 2H), 6.96 (d, *J* = 1.8 Hz, 4H), 3.97 (q, *J* = 7.1 Hz, 2H), 3.74 (bs, 2H), 1.62 (d, *J* = 7.1 Hz, 6H), 1.25 (s, 36H) ppm.

^{13}C NMR (101 MHz, Chloroform-*d*): δ = 151.2, 145.4, 143.8, 129.7, 125.6 (q, *J* = 270.7 Hz), 122.6 (q, *J* = 3.9 Hz), 121.5, 120.6, 119.3 (q, *J* = 31.9 Hz), 41.0, 34.9, 31.5, 22.3 ppm.

^{19}F NMR (376 MHz, Chloroform-*d*): δ = -60.66 ppm.

HRMS (ESI/QTOF): *m/z*: $[M + H]^+$ Calcd for $C_{39}H_{55}F_3N^+$ 594.4281; Found 594.4284.

IR (ATR): 2963, 1596, 1362, 1268, 1111, 758 cm^{-1}.

$[\alpha]_D^{20}$ = +102.6 (c = 0.5, $CHCl_3$).

Chiral HPLC: (Chiralpak IG, 0.1 % *i*PrOH/hexane, 1.0 mL/min, 254 nm): t_R (*R*,*R*) = 4.56 min, >99.9:0.1 er.

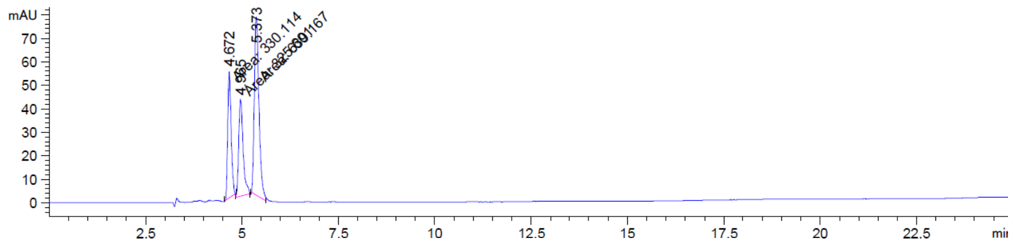

Peak #	RetTime [min]	Type	Width [min]	Area [mAU*s]	Height [mAU]	Area %
1	4.672	MM	0.1017	330.11359	54.11854	25.4937
2	4.965	MM	0.1314	325.60065	41.28902	25.1452
3	5.373	MM	0.1401	639.16730	76.01381	49.3611

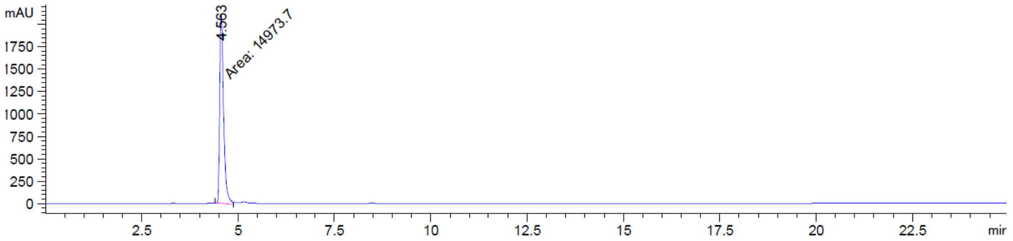

Peak #	RetTime [min]	Type	Width [min]	Area [mAU*s]	Height [mAU]	Area %
1	4.563	MM	0.1173	1.49737e4	2126.96973	100.0000

General procedure for the synthesis of chiral thiazolium salts (GP 4)

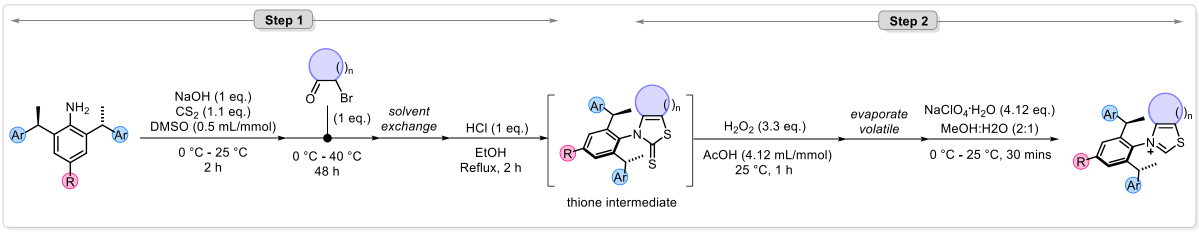

According to a slightly modified procedure previously reported in the literature by Bach[4] and Glorius.[5]

Step 1

A solution of chiral aniline (1 eq.) in DMSO (0.5 mL/mmol) was treated with 20 N aqueous NaOH (1 eq.) at ambient temperature. The mixture was then cooled to 0 °C, and CS_2 (1.1 eq.) was added dropwise, resulting in a color change from pale yellow to dark red. The reaction mixture was allowed to return to ambient temperature (25 °C) and stirred for 2 hours, during which the color gradually changed from dark red to yellow or orange. The mixture was then cooled to 0 °C again and stirred for 5 minutes. After that, *α*-bromo ketone (1 eq.) was added either dropwise (for liquid) or portion-wise (for solid) at 0 °C, and the mixture was stirred at 40 °C for 48 hours. Following this, the reaction mixture was cooled to 0 °C and quenched with water (1 mL/mmol). The mixture was extracted with ethyl acetate, and the organic fractions were dried over Na_2SO_4. The solvent was then evaporated under reduced pressure, yielding a yellow solid. This yellow solid was dissolved in EtOH (1 mL/mmol), and 37% fuming HCl (0.05 mL/mmol) was added. The resulting mixture was refluxed for 2 hours, during which the color changed from yellow or orange to greenish-black. The reaction mixture was then cooled to room temperature, extracted with ethyl acetate, dried over Na_2SO_4, and the solvent was evaporated under reduced pressure to obtain a crude mixture of thione. This mixture was partially purified using silica gel flash column chromatography (*n*-pentane/ethylacetate). (*Note: During purification, a mixture of unidentifiable products and the desired thione product was often encountered. Therefore, the percentage of purity of the thione was determined by* ^{1}*H NMR before proceeding to the next step.*)

Step 2

Thione (1 eq.) was dissolved in acetic acid (4.5 mL/mmol), and 30% H_2O_2 (3.3 eq.) was added while cooling the reaction mixture with a water bath. The mixture was then stirred at ambient temperature (25 °C) for 1 hour. Afterward, the solvent was removed under reduced pressure, and the residue was dissolved in methanol (0.5 mL/mmol). The resulting mixture was cooled to 0 °C, and a solution of $NaClO_4 \cdot H_2O$ (4.12 eq.) in a methanol/water mixture (2:1, total volume 3.5 mL/mmol) was added slowly. After stirring for 10 minutes at 0 °C, the reaction mixture was stirred for an additional 20 minutes at ambient temperature. The reaction mixture was then diluted and extracted with DCM. The organic fractions were dried over Na_2SO_4 and evaporated under reduced pressure. The crude residue was purified by silica gel column chromatography using a DCM/MeOH mixture as the eluent, yielding pure chiral thiazolium salts.

Modified procedure for the synthesis of chiral thiazolium salts (GP 5)

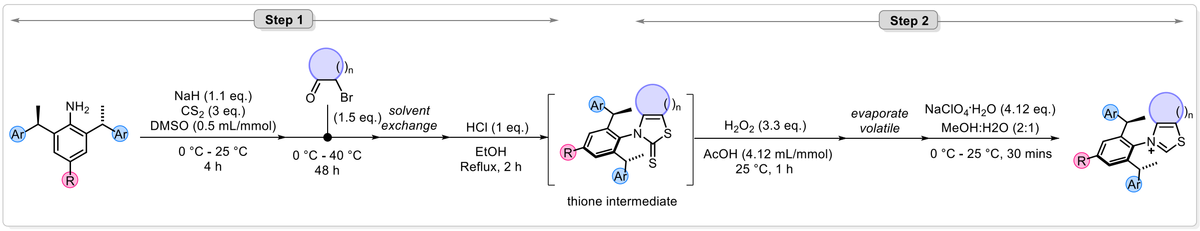

According to the slightly modified procedure previously reported in the literature by Bach[4] and Glorius.[5]

Step 1

A solution of chiral aniline (1 eq.) in DMSO (0.5 mL/mmol) was treated with 60% NaH (1.1 eq.) at 0 °C. CS_2 (3 eq.) was added dropwise, resulting in a color change from pale yellow to dark red. The reaction mixture was allowed to return to ambient temperature (25 °C) and stirred for 4 hours, during which the color gradually changed from dark red to light red. The mixture was then cooled to 0 °C again and stirred for 5 minutes. After that, α-bromo ketone (1.5 eq.) was added portion-wise at 0 °C, and the mixture was stirred at 40 °C for 48 hours. Following this, the reaction mixture was cooled to 0 °C and quenched with water (1 mL/mmol). The mixture was extracted with ethylacetate, and the organic fractions were dried over Na_2SO_4. The solvent was then evaporated under reduced pressure, yielding a pale red solid. This solid was dissolved in EtOH (1 mL/mmol), and 37% fuming HCl (0.05 mL/mmol) was added. The resulting mixture was refluxed for 2 hours, during which the color changed from red to greenish black. The reaction mixture was then cooled to room temperature, extracted with ethylacetate, dried over Na_2SO_4, and the solvent was evaporated under reduced pressure to obtain a crude mixture of thione intermediate. This mixture was partially purified using silica gel flash column chromatography (*n*-pentane/ethylacetate). (*Note: During purification, a mixture of unidentifiable products and the desired thione intermediate was often encountered. Therefore, the percentage of purity of the thione was determined by ^{1}H NMR before proceeding to the next step*).

Step 2

Thione (1 eq.) was dissolved in acetic acid (4.5 mL/mmol), and 30% H_2O_2 (3.3 eq.) was added while cooling the reaction mixture with a water bath. The mixture was then stirred at ambient temperature (25 °C) for 1 hour. Afterward, the solvent was removed under reduced pressure, and the residue was dissolved in methanol (0.5 mL/mmol). The resulting mixture was cooled to 0 °C, and a solution of $NaClO_4 \cdot H_2O$ (4.12 eq.) in a methanol/water mixture (2:1, total volume 3.5 mL/mmol) was added slowly. After stirring for 10 minutes at 0 °C, the reaction mixture was stirred for an additional 20 minutes at ambient temperature. The reaction mixture was then diluted and extracted with DCM. The organic fractions were dried over Na_2SO_4 and evaporated under reduced pressure. The crude residue was purified by silica gel column chromatography using a DCM/MeOH mixture as the eluent, yielding pure chiral thiazolium salts.

Physical data for chiral thiazolium salts

3-(2,6-Bis((*R*)-1-phenylethyl)phenyl)-5,6,7,8-tetrahydro-4*H*-cyclohepta[*d*]thiazol-3-ium perchlorate (NHC1)

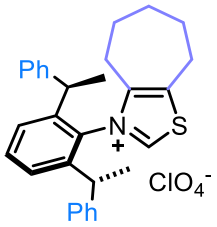

The title compound was synthesized on a 0.5 mmol scale according to the general procedure (**GP 4**) and was obtained as a colorless solid after silica gel column chromatography (DCM: methanol 20:1). Yield: 62% (166.5 mg). **R$_f$** = 0.28 (DCM: methanol 20:1).

MP: 181-182 °C

^{1}H NMR (400 MHz, Chloroform-*d*): δ = 8.77 (s, 1H), 7.85 – 7.76 (m, 2H), 7.68 (dd, *J* = 7.4, 1.9 Hz, 1H), 7.24 – 7.19 (m, 3H), 7.17 – 7.11 (m, 3H), 6.75 – 6.70 (m, 2H), 6.66 – 6.61 (m, 2H), 3.89 (q, *J* = 6.9 Hz, 1H), 3.37 (q, *J* = 7.1 Hz, 1H), 3.04 – 2.95 (m, 1H), 2.69 – 2.60 (m, 1H), 1.98 – 1.80 (m, 3H), 1.66 – 1.56 (m, 7H), 1.49 – 1.36 (m, 1H), 1.31 – 1.15 (m, 2H), 1.01 – 0.90 (m, 1H) ppm.

^{13}C NMR (101 MHz, Chloroform-*d*): δ = 156.4, 147.7, 144.1, 143.3, 142.1, 140.4, 138.4, 134.9, 131.8, 129.3, 128.6, 127.6, 127.4, 127.2, 126.7, 126.3, 125.8, 40.6, 39.8, 30.4, 27.9, 26.4, 26.2, 24.8, 22.7, 22.0 ppm.

HRMS (ESI/QTOF): *m/z*: [M]$^+$ Calcd for $C_{30}H_{32}NS^+$ 438.2250; Found 438.2257.

IR (ATR): 2931, 2856, 1493, 1450, 1087, 1028, 758, 572 cm^{-1}.

$[\alpha]_D^{20}$ = +90.0 (c = 0.5, $CHCl_3$).

3-(4-Methoxy-2,6-bis((*R*)-1-phenylethyl)phenyl)-5,6,7,8-tetrahydro-4*H*-cyclohepta[*d*]thiazol-3-ium perchlorate (NHC2)

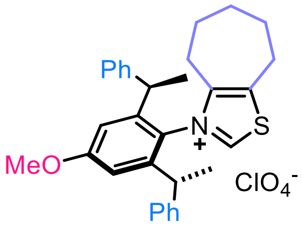

The title compound was synthesized on a 0.5 mmol scale according to the general procedure (**GP 4**) and was obtained as a pale-yellow solid after silica gel column chromatography (DCM: methanol 10:1). Yield: 67% (190 mg). **R$_f$** = 0.26 (DCM: methanol 10:1).

MP: 191-192 °C

^{1}H NMR (400 MHz, Chloroform-*d*): δ = 8.71 (s, 1H), 7.24 – 7.19 (m, 4H), 7.16 – 7.11 (m, 4H), 6.76 – 6.71 (m, 2H), 6.67 – 6.63 (m, 2H), 4.02 (s, 3H), 3.86 (q, *J* = 6.8 Hz, 1H), 3.33 (q, *J* = 7.0 Hz, 1H), 3.04 – 2.94 (m, 1H), 2.70 – 2.57 (m, 1H), 2.02 – 1.76 (m, 3H), 1.70 – 1.60 (m, 1H), 1.57 – 1.52 (m, 6H), 1.48 – 1.37 (m, 1H), 1.32 – 1.14 (m, 2H), 1.01 – 0.96 (m, 1H) ppm.

^{13}C NMR (101 MHz, Chloroform-*d*): δ = 161.9, 157.2, 148.4, 144.3, 144.1, 143.52, 142.3, 138.5, 129.6, 128.9, 127.94, 127.90, 127.4, 127.0, 126.1, 112.4, 112.3, 55.9, 41.0, 40.2, 30.8, 28.2, 26.8, 26.5, 25.1, 23.0, 22.2 ppm.

HRMS (ESI/QTOF): *m/z*: [M]$^+$ Calcd for $C_{31}H_{34}NOS^+$ 468.2356; Found 468.2359.

IR (ATR): 2962, 1598, 1508, 1091, 622 cm^{-1}.

$[\alpha]_D^{20}$ = +37.0 (c = 1.0, $CHCl_3$).

3-(2,6-Bis((*R*)-1-phenylethyl)-4-(trifluoromethyl)phenyl)-5,6,7,8-tetrahydro-4*H*-cyclohepta[*d*]thiazol-3-ium perchlorate (NHC3)

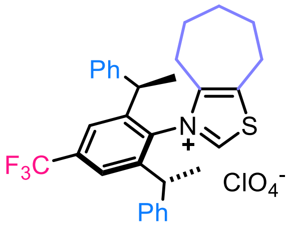

The title compound was synthesized on a 0.5 mmol scale according to the general procedure (**GP 4**) and was obtained as a colorless solid after silica gel column chromatography (DCM: methanol 20:1). Yield: 49% (148 mg). $\mathbf{R_f}$ = 0.32 (DCM: methanol 20:1).

MP: 184-185 °C

^{1}H NMR (400 MHz, Chloroform-*d*): δ = 8.89 (s, 1H), 8.03 (s, 1H), 7.91 (s, 1H), 7.25 – 7.22 (m, 3H), 7.18 – 7.12 (m, 3H), 6.70 (dd, *J* = 7.4, 2.1 Hz, 2H), 6.61 (dd, *J* = 6.6, 3.0 Hz, 2H), 4.02 (q, *J* = 6.9 Hz, 1H), 3.42 (q, *J* = 7.0 Hz, 1H), 3.06 – 2.96 (m, 1H), 2.72 – 2.58 (m, 1H), 2.05 – 1.82 (m, 3H), 1.65 – 1.59 (m, 7H), 1.49 – 1.38 (m, 1H), 1.32 – 1.24 (m, 2H), 0.99 – 0.85 (m, 1H) ppm.

^{13}C NMR (101 MHz, Chloroform-*d*): δ = 155.9, 146.4, 143.2, 142.4, 141.5, 141.2, 138.0, 136.7, 133.0 (q, *J* = 32.7 Hz), 128.7, 127.9, 127.1, 126.3, 126.2, 124.8, 123.6 (q, *J* = 3.8 Hz), 122.26 (q, *J* = 273.6 Hz), 122.4 (q, *J* = 3.7 Hz), 39.9, 39.1, 29.5, 27.0, 25.5, 25.4, 24.0, 21.7, 21.1 ppm.

^{19}F NMR (376 MHz, Chloroform-*d*): δ = -62.73 ppm.

HRMS (ESI/QTOF): *m/z*: [M]$^+$ Calcd for $C_{31}H_{31}F_3NS^+$ 506.2124; Found 506.2131.

IR (ATR): 2925, 1669, 1170, 1093, 623 cm^{-1}.

$[\alpha]_D^{20}$ = +32.0 (c = 1.0, $CHCl_3$).

3-(2,6-Bis((*R*)-1-phenylethyl)phenyl)-4,5,6,7,8,9-hexahydrocycloocta[*d*]thiazol-3-ium perchlorate (NHC4)

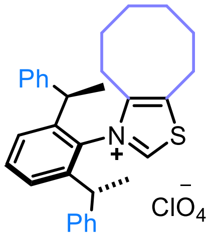

The title compound was synthesized on a 0.5 mmol scale according to the general procedure (**GP 4**) and was obtained as a colorless solid after silica gel column chromatography (DCM: methanol 20:1). Yield: 39% (107.5 mg). $\mathbf{R_f}$ = 0.28 (DCM: methanol 20:1).

MP: 176-177 °C

^{1}H NMR (400 MHz, Chloroform-*d*): δ = 8.73 (s, 1H), 7.87 – 7.78 (m, 2H), 7.71 (dd, *J* = 7.4, 1.8 Hz, 1H), 7.26 – 7.22 (m, 3H), 7.16 – 7.11 (m, 3H), 6.82 – 6.76 (m, 2H), 6.65 – 6.59 (m, 2H), 3.73 (q, *J* = 6.9 Hz, 1H), 3.43 (q, *J* = 7.1 Hz, 1H), 3.14 – 3.01 (m, 1H), 2.80 – 2.67 (m, 1H), 1.98 – 1.86 (m, 1H), 1.82 – 1.66 (m, 2H), 1.60 – 1.58 (m, 3H), 1.57 – 1.54 (m, 3H), 1.50 – 1.38 (m, 5H), 1.36 – 1.30 (m, 2H) ppm.

^{13}C NMR (101 MHz, $CDCl_3$): δ = 157.8, 145.8, 143.6, 143.4, 142.3, 140.5, 137.5, 134.8, 132.0, 129.6, 128.6, 128.0, 127.8, 127.4, 126.9, 126.5, 126.3, 41.0, 40.1, 29.3, 26.3, 25.8, 25.5, 24.5, 24.2, 23.2, 22.2 ppm.

HRMS (ESI/QTOF): *m/z*: [M]$^+$ Calcd for $C_{31}H_{34}NS^+$ 452.2406; Found 452.2422.

IR (ATR): 2930, 1450, 1089, 759, 702, 666 cm^{-1}.

$[\alpha]_D^{20}$ = +33.0 (c = 1.0, $CHCl_3$).

3-(2,6-Bis((*R*)-1-phenylethyl)-4-(trifluoromethyl)phenyl)-4,5,6,7,8,9,10,11,12,13-decahydrocyclododeca[*d*]thiazol-3-ium perchlorate (NHC5)

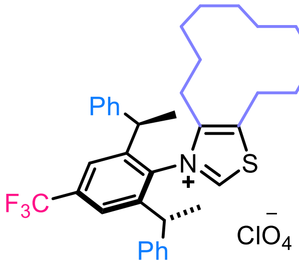

The title compound was synthesized on a 0.5 mmol scale according to the general procedure (**GP 4/GP 5**) and was obtained as a colorless foam after silica gel column chromatography (DCM: methanol 40:1). Yield: 19% (64 mg) by **GP A**; Yield: 37% (124 mg) by **GP B**. $\mathbf{R_f}$ = 0.32 (DCM: methanol 40:1).
^{1}H NMR (400 MHz, Chloroform-*d*): δ = 8.85 (s, 1H), 8.05 (s, 1H), 7.92 (s, 1H), 7.30 – 7.26 (m, 3H), 7.20 – 7.14 (m, 3H), 6.83 – 6.78 (m, 2H), 6.60 – 6.56 (m, 2H), 3.82 (q, *J* = 6.9 Hz, 1H), 3.45 (q, *J* = 7.0 Hz, 1H), 2.87 – 2.67 (m, 2H), 2.04 – 1.89 (m, 1H), 1.81 – 1.66 (m, 2H), 1.626 – 1.60 (m, 6H), 1.52 – 1.28 (m, 11H), 1.20 – 1.01 (m, 4H) ppm.
^{13}C NMR (101 MHz, Chloroform-*d*): δ = 159.3, 144.5, 144.1, 142.4, 142.3, 142.0, 141.0, 137.3, 134.0 (q, *J* = 32.9 Hz), 129.7, 128.8, 128.2, 127.2, 127.1, 126.0, 124.8 (q, *J* = 3.4 Hz), 123.14 (q, *J* = 3.6 Hz), 123.10 (d, *J* = 273.5 Hz), 41.3, 40.0, 29.3, 25.59, 25.55, 25.3, 25.1, 24.2, 23.8, 23.1, 23.1, 22.2, 21.8, 21.7 ppm.
^{19}F NMR (376 MHz, Chloroform-*d*): δ = -62.68 ppm.
HRMS (ESI/QTOF): *m/z*: [M]$^+$ Calcd for $C_{36}H_{41}F_3NS^+$ 576.2906; Found 576.2921.
IR (ATR): 2929, 2856, 1495, 1451, 1094, 763, 702 cm^{-1}.
$[\alpha]_D^{20}$ = +27.0 (c = 1.0, $CHCl_3$).

3-(2,6-Bis((*R*)-1-(3,5-dimethylphenyl)ethyl)-4-(trifluoromethyl)phenyl)-4,5,6,7,8,9,10,11,12,13-decahydrocyclododeca[*d*]thiazol-3-ium perchlorate (NHC6)

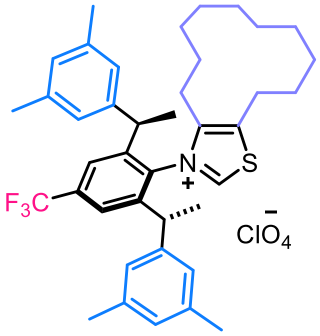

The title compound was synthesized on a 0.5 mmol scale according to the general procedure (**GP 5**) and was obtained as a colorless foam after silica gel column chromatography (DCM: methanol 50:1). Yield: 35% (128 mg). $\mathbf{R_f}$ = 0.30 (DCM: methanol 50:1).
^{1}H NMR (400 MHz, Chloroform-*d*): δ = 8.83 (s, 1H), 8.03 (d, *J* = 1.9 Hz, 1H), 7.90 (d, *J* = 1.9 Hz, 1H), 6.92 (s, 1H), 6.80 (s, 1H), 6.35 (s, 1H), 6.10 (s, 2H), 3.75 (q, *J* = 6.8 Hz, 1H), 3.41 (q, *J* = 7.0 Hz, 1H), 2.86 – 2.65 (m, 2H), 2.30 – 2.24 (m, 1H), 2.22 (s, 6H), 2.17 (s, 6H), 1.99 – 1.87 (m, 1H), 1.84 – 1.73 (m, 1H), 1.71 – 1.63 (m, 2H), 1.60 (d, *J* = 6.9 Hz, 3H), 1.54 – 1.58 (m, 3H), 1.48 – 1.34 (m, 10H), 1.21 – 1.02 (m, 4H) ppm.
^{13}C NMR (101 MHz, Chloroform-*d*): δ = 160.3, 144.9, 144.5, 142.9, 142.6, 142.4, 140.5, 139.7, 138.6, 137.7 (q, *J* = 1.3 Hz) 134.1 (q, *J* = 32.8 Hz), 130.2, 128.8, 125.3, 124.9 (q, *J* = 3.4 Hz), 124.0, 123.5 (q, *J* = 273.5 Hz), 123.2 (q, *J* = 3.8 Hz), 41.6, 40.2, 29.9, 29.8, 26.0, 25.7, 24.6, 24.5, 23.4, 23.2, 22.8, 22.2, 22.0, 21.4, 21.3 ppm.
^{19}F NMR (376 MHz, Chloroform-*d*): δ = -62.62 ppm.
HRMS (ESI/QTOF): *m/z*: [M]$^+$ Calcd for $C_{40}H_{49}F_3NS^+$ 632.3532; Found 632.3547.
IR (ATR): 2922, 2853, 1466, 1451, 1090, 754, 622 cm^{-1}.

$[\alpha]_D^{20}$ = +41.0 (c = 1.0, $CHCl_3$).

3-(2,6-Bis((*R*)-1-(3,5-di-tert-butylphenyl)ethyl)-4-(trifluoromethyl)phenyl)-4,5,6,7,8,9,10,11,12,13-decahydrocyclododeca[*d*]thiazol-3-ium perchlorate (NHC7)

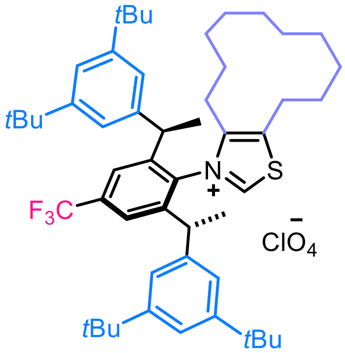

The title compound was synthesized on a 1.0 mmol scale according to the general procedure (**GP 5**) and was obtained as a colorless foam after silica gel column chromatography (DCM: methanol 80:1). Yield: 29% (260 mg). $\mathbf{R_f}$ = 0.30 (DCM: methanol 80:1).

^{1}H NMR (400 MHz, Chloroform-*d*): δ = 8.56 (s, 1H), 8.07 (s, 1H), 7.95 (s, 1H), 7.34 (t, *J* = 1.8 Hz, 1H), 7.22 (t, *J* = 1.7 Hz, 1H), 6.76 (d, *J* = 1.8 Hz, 2H), 6.39 (d, *J* = 1.7 Hz, 2H), 3.77 (q, *J* = 7.2 Hz, 1H), 3.36 (q, *J* = 7.2 Hz, 1H), 2.89 – 2.74 (m, 2H), 2.03 – 1.70 (m, 2H), 1.63 – 1.58 (m, 6H), 1.50 – 1.44 (m, 4H), 1.40 – 1.36 (m, 4H), 1.27 – 1.21 (m, 42H), 1.16 – 1.01 (m, 2H) ppm.

^{13}C NMR (101 MHz, Chloroform-*d*): δ = 160.1, 144.7, 144.3, 142.6, 142.4, 142.2, 140.3, 139.5, 138.4, 137.5, 133.9 (q, *J* = 33.0 Hz), 130.0, 128.6, 125.1, 124.7 (q, *J* = 3.6 Hz), 123.8, 123.3 (q, *J* = 273.7 Hz), 123.0 (q, *J* = 3.2 Hz), 41.4, 40.0, 36.3, 29.7, 29.6, 25.8, 25.5, 24.4, 24.3, 23.2, 23.0, 22.6, 22.0, 21.8, 21.2, 21.1 ppm.

^{19}F NMR (376 MHz, Chloroform-*d*): δ = -62.59 ppm.

HRMS (ESI/QTOF): *m/z*: $[M]^+$ Calcd for $C_{52}H_{73}F_3NS^+$ 800.5410; Found 800.5415.

IR (ATR): 2926, 2854, 1452, 1349, 1133, 622 cm^{-1}.

$[\alpha]_D^{20}$ = +46.0 (c = 1.0, $CHCl_3$).

3-(4-Methyl-2,6-bis((*R*)-1-phenylethyl)phenyl)-5,6,7,8-tetrahydro-4*H*-cyclohepta[*d*]thiazol-3-ium perchlorate (NHC10)

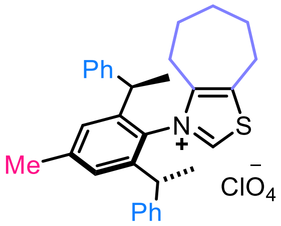

The title compound was synthesized on a 0.5 mmol scale according to the general procedure (**GP 4**) and was obtained as a colorless solid after silica gel column chromatography (DCM: methanol 20:1). Yield: 64% (177 mg). $\mathbf{R_f}$ = 0.30 (DCM: methanol 20:1).

MP: 186-187 °C

^{1}H NMR (400 MHz, Chloroform-*d*): δ = 8.73 (s, 1H), 7.54 (s, 1H), 7.43 (s, 1H), 7.24 – 7.18 (m, 3H), 7.16 – 7.11 (m, 3H), 6.75 – 6.69 (m, 2H), 6.67 – 6.61 (m, 2H), 3.84 (q, *J* = 6.9 Hz, 1H), 3.33 (q, *J* = 7.1 Hz, 1H), 3.04 – 2.94 (m, 1H), 2.67 – 2.57 (m, 4H), 1.99 – 1.89 (m, 2H), 1.87 – 1.79 (m, 1H), 1.65 – 1.58 (m, 1H), 1.57 – 1.52 (m, 6H), 1.47 – 1.36 (m, 1H), 1.27 – 1.17 (m, 2H), 1.0 – 0.9 (m, 1H) ppm.

^{13}C NMR (101 MHz, Chloroform-*d*): δ = 156.8, 148.1, 144.5, 143.7, 142.5, 142.0, 140.3, 138.6, 132.7, 129.5, 128.9, 128.2, 127.8, 127.5, 127.2, 127.0, 126.1, 40.8, 40.0, 30.8, 28.2, 26.7, 26.5, 25.1, 23.0, 22.3, 22.2 ppm.

HRMS (ESI/QTOF): *m/z*: $[M]^+$ Calcd for $C_{31}H_{34}NS^+$ 452.2406; Found 452.2419.

IR (ATR): 2931, 1450, 1088, 912, 763, 729 cm^{-1}.
$[\alpha]_D^{20}$ = +36.0 (c = 1.0, $CHCl_3$).

3-(2,6-Bis((*R*)-1-(3,5-dimethylphenyl)ethyl)phenyl)-5,6,7,8-tetrahydro-4*H*-cyclohepta[*d*]thiazol-3-ium perchlorate (NHC11)

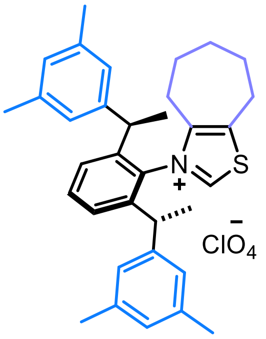

The title compound was synthesized on a 0.5 mmol scale according to the general procedure (**GP 4**) and was obtained as a colorless solid after silica gel column chromatography (DCM: methanol 20:1). Yield: 60% (178 mg). **R$_f$** = 0.32 (DCM: methanol 20:1).
MP: 178-179 °C
^{1}H NMR (400 MHz, Chloroform-*d*): δ = 8.75 (s, 1H), 7.83 – 7.74 (m, 2H), 7.68 – 7.63 (m, 1H), 6.86 (s, 1H), 6.77 (s, 1H), 6.31 (s, 2H), 6.18 (s, 2H), 3.79 (q, *J* = 6.8 Hz, 1H), 3.29 (q, *J* = 7.0 Hz, 1H), 3.02 – 2.93 (m, 1H), 2.70 – 2.60 (m, 1H), 2.24 – 2.12 (m, 13H), 2.03 – 1.92 (m, 2H), 1.89 – 1.80 (m, 1H), 1.69 – 1.61(m, 1H), 1.56 – 1.52 (m, 6H), 1.51 – 1.41 (m, 1H), 1.24 – 1.17 (m, 1H), 1.15 – 1.05 (m, 1H) ppm.
^{13}C NMR (101 MHz, $CDCl_3$): δ = 157.5, 147.9, 144.4, 143.6, 142.6, 140.8, 139.3, 138.4, 138.3, 135.1, 132.0, 129.6, 128.5, 127.7, 126.3, 125.2, 123.8, 40.9, 40.1, 31.0, 29.8, 28.1, 26.9, 26.7, 25.1, 23.0, 22.3, 21.4, 21.3 ppm.
HRMS (ESI/QTOF): *m/z*: [M]$^+$ Calcd for $C_{34}H_{40}NS^+$ 494.2876; Found 494.2892.
IR (ATR): 2924, 1953, 1558, 1265, 1092, 737 cm^{-1}.
$[\alpha]_D^{20}$ = +57.4 (c = 1.0, $CHCl_3$).

3-(4-Methyl-2,6-bis((*R*)-1-phenylethyl)phenyl)-4,5,6,7,8,9,10,11,12,13-decahydrocyclododeca[*d*]thiazol-3-ium perchlorate (NHC12)

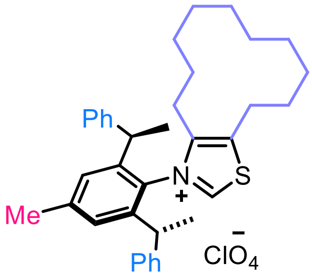

The title compound was synthesized on a 0.5 mmol scale according to the general procedure (**GP 5**) and was obtained as a colorless solid after silica gel column chromatography (DCM: methanol 40:1). Yield: 39% (121 mg). **R$_f$** = 0.30 (DCM: methanol 40:1).
^{1}H NMR (400 MHz, Chloroform-*d*): δ = 8.67 (s, 1H), 7.55 (s, 1H), 7.46 (s, 1H), 7.26 – 7.23 (m, 3H), 7.18 – 7.13 (m, 3H), 6.86 – 6.83 (m, 2H), 6.66 – 6.59 (m, 2H), 3.63 (q, *J* = 6.9 Hz, 1H), 3.34 (q, *J* = 7.0 Hz, 1H), 2.84 – 2.67 (m, 2H), 2.63 (s, 3H), 1.99 – 1.88 (m, 1H), 1.81 – 1.70 (m, 2H), 1.56 – 1.53 (m, 6H), 1.51 – 1.40 (m, 6H), 1.33 – 1.26 (m, 4H), 1.19 – 1.01 (m, 5H) ppm.
^{13}C NMR (101 MHz, Chloroform-*d*): δ = 159.4, 145.3, 143.8, 143.7, 142.6, 142.2, 140.8, 140.2, 132.4, 129.7, 128.8, 128.6, 128.1, 127.6, 127.2, 127.0, 126.5, 41.3, 40.0, 29.6, 25.9, 25.7, 25.5, 25.4, 24.6, 24.1, 23.7, 23.4, 22.5, 22.3, 22.2, 22.0 ppm.
HRMS (ESI/QTOF): *m/z*: [M]$^+$ Calcd for $C_{36}H_{44}NS^+$ 522.3189; Found 522.3200.

IR (ATR): 2929, 2857, 1493, 1450, 1093, 762, 730, 702 cm^{-1}.
$[\alpha]_D^{20}$ = +31.6 (c = 1.0, $CHCl_3$).

Synthesis of starting materials

Synthesis of 2,2-dimethylcyclopropane-1-carbaldehyde (1y)

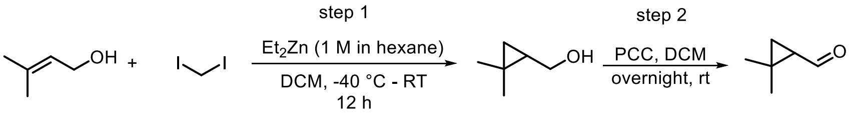

Synthesized following the literature procedure.[6]
Step 1: To a solution of the 3-methylbut-2-en-1-ol (1.0 eq.) in anhydrous DCM (3.5 mL/mmol) at -40 °C, $ZnEt_2$ (1 M in hexane, 2.5 eq.) was added dropwise over 10 minutes. The reaction mixture was stirred for 5 minutes before neat CH_2I_2 (2.0 eq.) was added dropwise over 5 minutes. The reaction was then allowed to warm to room temperature and stirred for overnight. The mixture was quenched with saturated aqueous NH_4Cl, the phases were separated, and the aqueous layer was extracted with DCM. The combined organic extracts were dried over $MgSO_4$, filtered, and concentrated in vacuo. The crude product was purified by flash column chromatography using 5% methanol in methylene chloride as the eluent, yielding pure (2,2-dimethylcyclopropyl)methanol as a colorless liquid (81% yield).
^{1}H NMR (400 MHz, Chloroform-*d*): δ = 5.65 (dd, *J* = 11.4, 6.7 Hz, 1H), 5.48 (dd, *J* = 11.4, 8.4 Hz, 1H), 3.51 (bs, 1H), 3.08 (s, 3H), 3.04 (s, 3H), 2.93 – 2.82(m, 1H), 2.44 (dd, *J* = 8.5, 4.4 Hz, 1H), 2.11-2.07 (m, 1H) ppm.
Physical data is in accordance with the literature.[6]

Step 2: To an ice-cooled solution of (2,2-dimethylcyclopropyl)methanol (1 eq.) in dry CH_2Cl_2 (3 mL/mmol), PCC (1.0 eq.) was added. The reaction mixture was stirred at ambient temperature overnight. Ether was then added, and the mixture was filtered through a Celite bed. The black residue was washed with ether, and the combined filtrate was concentrated under reduced pressure (30 °C, 300 mBar). The crude product was purified by silica gel chromatography using CH_2Cl_2 as the eluent, yielding 2,2-dimethylcyclopropane-1-carbaldehyde (74%) as a colorless oil.
^{1}H NMR (400 MHz, Chloroform-*d*): δ = 9.33 (d, *J* = 5.5 Hz, 1H), 1.70 (dt, *J* = 8.0, 5.3 Hz, 1H), 1.34 (t, *J* = 4.9 Hz, 1H), 1.27 (s, 3H), 1.19 (s, 3H), 1.07 (dd, *J* = 8.0, 4.5 Hz, 1H) ppm.
Physical data is in accordance with the literature.[6]

General Procedure for the Synthesis of *α*-bromo-*α*,*α*-difluoroesters (GP 6)

α-Bromo-*α*,*α*-difluoroesters **3b-3e, ca, cb** were prepared according to the literature procedure.[7]
α-Bromo-*α*,*α*-difluoroacetic acid (1.0 eq.) was dissolved in dry DCM (c = 0.45 M) in a dry round-bottom flask under a nitrogen atmosphere. Oxalyl chloride (1.1 eq.) was then gradually added to the reaction mixture over 5 minutes while stirring at room temperature. Following this, two drops of DMF were added, and the mixture was stirred for an additional 2 hours at room temperature until gas evolution ceased. The corresponding alcohol (2.0 eq.) and triethylamine (1.1 eq.), dissolved in dry DCM (c = 0.2 M), were then added dropwise to the reaction at 0 °C over a period of 20 minutes. The cooling bath was then removed, and the reaction mixture was stirred at room temperature for 3 hours. The crude reaction mixture was quenched with water and extracted with DCM. The combined organic layers were washed with a saturated $NaHCO_3$ solution, dried over $MgSO_4$, filtered, and concentrated under reduced pressure. The resulting residue was purified by silica gel column chromatography using an *n*-pentane : diethyl ether mixture to obtain the desired product.

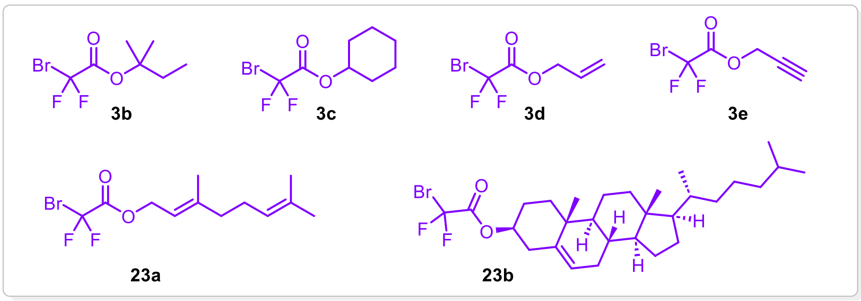

tert-Pentyl 2-bromo-2,2-difluoroacetate (3b)

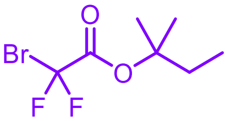

The titled compound was synthesized following the general procedure 6 (**GP 6**) and obtained after silica gel flash chromatography using *n*-pentane : diethyl ether (50:1) as a colorless, volatile liquid (53% yield).
^{1}H NMR (400 MHz, Chloroform-*d*): δ = 1.87 (q, *J* = 7.5 Hz, 2H), 1.54 (s, 6H), 0.96 (t, *J* = 7.5 Hz, 3H) ppm.
^{19}F NMR (376 MHz, Chloroform-*d*): δ = -60.65 ppm.
Physical data is in accordance with the literature.[7]

Cyclohexyl 2-bromo-2,2-difluoroacetate (3c)

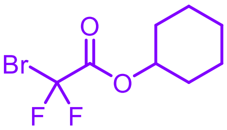

The titled compound was synthesized following the general procedure 6 (**GP 6**) and obtained after silica gel flash chromatography using *n*-pentane : diethyl ether (50:1) as a colorless, volatile liquid (64% yield).
^{1}H NMR (400 MHz, Chloroform-*d*): δ = 5.02 – 4.92 (m, 1H), 1.96 – 1.86 (m, 2H), 1.84 – 1.72 (m, 2H), 1.68 – 1.50 (m, 3H), 1.49 – 1.28 (m, 3H) ppm.
^{19}F NMR (376 MHz, Chloroform-*d*): δ = -60.84 ppm.
Physical data is in accordance with the literature.[8]

Allyl 2-bromo-2,2-difluoroacetate (3d)

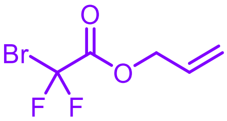

The titled compound was synthesized following the general procedure 6 (**GP 6**) and obtained after silica gel flash chromatography using *n*-pentane : diethyl ether (40:1) as a colorless, volatile liquid (69% yield).
^{1}H NMR (400 MHz, Chloroform-*d*): δ = 6.01 – 5.92 (m, 1H), 5.55 – 5.31 (m, 2H), 4.83 (dt, *J* = 5.9, 1.3 Hz, 2H) ppm.
^{19}F NMR (376 MHz, Chloroform-*d*): δ = -60.67 ppm.
Physical data is in accordance with the literature.[9]

Prop-2-yn-1-yl 2-bromo-2,2-difluoroacetate (3e)

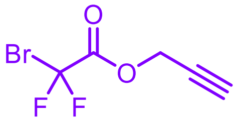

The titled compound was synthesized following the general procedure 6 (**GP 6**) and obtained after silica gel flash chromatography using *n*-pentane : diethyl ether (40:1) as a colorless, volatile liquid (59% yield).
^{1}H NMR (400 MHz, Chloroform-*d*): δ = 4.93 (d, *J* = 2.5 Hz, 2H), 2.63 (t, *J* = 2.5 Hz, 1H) ppm.
^{19}F NMR (376 MHz, Chloroform-*d*): δ = -60.97 ppm.
Physical data is in accordance with the literature.[10]

(*E*)-3,7-Dimethylocta-2,6-dien-1-yl 2-bromo-2,2-difluoroacetate (ca)

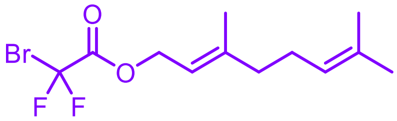

The titled compound was synthesized following the general procedure 6 (**GP 6**) and obtained after silica gel flash chromatography using *n*-pentane : diethyl ether (40:1) as a colorless oil (72% yield).
^{1}H NMR (400 MHz, Chloroform-*d*): δ = 5.43 – 5.37 (m, 1H), 5.10 – 5.03 (m, 1H), 4.85 (d, *J* = 7.4 Hz, 2H), 2.16 – 2.05 (m, 4H), 1.76 (d, *J* = 1.3 Hz, 3H), 1.68 (d, *J* = 1.4 Hz, 3H), 1.60 (s, 3H) ppm.
^{13}C NMR (101 MHz, Chloroform-d): δ = 159.6 (t, J = 31.1 Hz), 145.7, 132.1, 123.3, 115.9, 108.9 (t, J = 314.5 Hz), 65.0, 39.5, 26.1, 25.6, 17.7, 16.6 ppm.
^{19}F NMR (376 MHz, Chloroform-*d*): δ = -60.52 ppm.
HRMS (ESI/QTOF): *m/z*: [M + H]$^+$ Calcd for $C_{12}H_{18}BrF_2O_2^+$ 311.0453; Found 311.0448.
IR (ATR): 2918, 1772, 1291, 1122, 943, 709 cm^{-1}.

(3*S*,8*S*,9*S*,10*R*,13*R*,14*S*,17*R*)-10,13-Dimethyl-17-((*R*)-6-methylheptan-2-yl)-2,3,4,7,8,9,10,11,12,13,14,15,16,17-tetradecahydro-1*H*-cyclopenta[*a*]phenanthren-3-yl 2-bromo-2,2-difluoroacetate (cb)

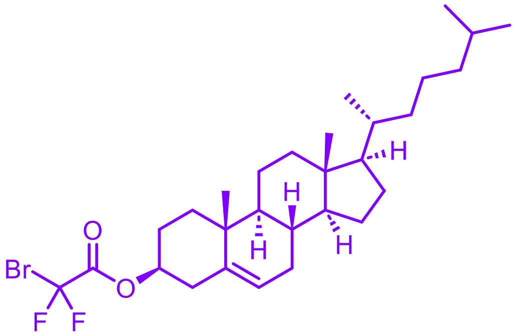

The titled compound was synthesized following the general procedure 6 (**GP 6**) and obtained after silica gel flash chromatography using *n*-pentane : diethyl ether (60:1) as a colorless solid (51% yield).
^{1}H NMR (400 MHz, Chloroform-d): δ = 5.50 – 5.35 (m, 1H), 4.87 – 4.86 (m, 1H), 2.53 – 2.32 (m, 2H), 2.08 – 1.66 (m, 6H), 1.64 – 1.57 (m, 1H), 1.54 – 1.53 (m, 1H), 1.52 – 0.94 (m, 21H), 0.92 (d, J = 6.6 Hz, 3H), 0.87 (dd, J = 6.6, 1.9 Hz, 6H), 0.68 (s, 3H) ppm.
^{19}F NMR (376 MHz, Chloroform-*d*): δ = -60.88 ppm.
Physical data is in accordance with the literature.[11]

General procedure for the synthesis of *α*-bromo-*α*,*α*-difluoroacetamides (GP 7)

α-Bromo-*α*,*α*-difluoroacetamides **3f-3i** were prepared according to a modified literature procedure.[7] To a round-bottom flask equipped with a stir bar was added ethyl bromodifluoroacetate (1 mmol) and an equimolar amount of amine (1 mmol, 1 eq.). The mixture was stirred at room temperature and

monitored by TLC. Upon complete consumption of the amine, the reaction mixture was quenched with 10% aqueous HCl and extracted with DCM (3 x 10 mL). The combined organic extracts were washed with water (1 x 20 mL) and brine (1 x 20 mL). The organic layers were then combined, dried over $MgSO_4$, filtered, and concentrated. The resulting residue was purified by silica gel flash chromatography using an *n*-pentane : diethyl ether mixture to obtain the desired amides.

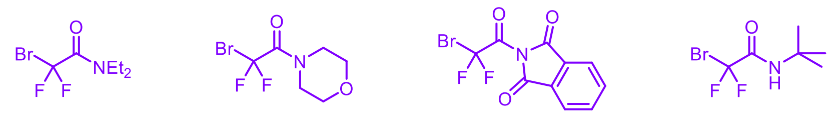

3f **3g** **3h** **3i**

2-Bromo-*N*,*N*-diethyl-2,2-difluoroacetamide (3f)

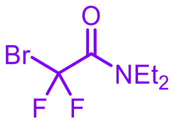

The titled compound was synthesized following the general procedure 7 (**GP 7**) and obtained after silica gel flash chromatography using *n*-pentane : diethyl ether (9:1) as a colorless oil (76% yield).
^{1}H NMR (400 MHz, Chloroform-*d*): δ = 3.52 (q, *J* = 7.0 Hz, 2H), 3.42 (q, *J* = 7.1 Hz, 2H), 1.24 (t, *J* = 7.1 Hz, 3H), 1.19 (t, *J* = 7.1 Hz, 3H) ppm.
^{19}F NMR (376 MHz, Chloroform-*d*): δ = -54.35 ppm.
Physical data is in accordance with the literature.[7]

2-Bromo-2,2-difluoro-1-morpholinoethan-1-one (3g)

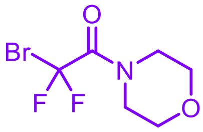

The titled compound was synthesized following the general procedure 7 (**GP 7**) and obtained after silica gel flash chromatography using *n*-pentane : diethyl ether (4:1) as a colorless oil (59% yield).
^{1}H NMR (400 MHz, Chloroform-*d*): δ = 3.78 – 3.66 (m, 8H) ppm.
^{19}F NMR (376 MHz, Chloroform-*d*): δ = -54.47 ppm.
Physical data is in accordance with the literature.[7]

2-Bromo-2,2-difluoro-1-(isoindolin-2-yl)ethan-1-one (3h)

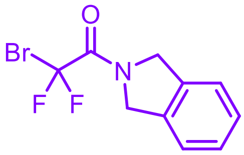

The titled compound was synthesized following the general procedure 7 (**GP 7**) and obtained after silica gel flash chromatography using *n*-pentane : diethyl ether (4:1) as a colorless solid (84% yield).
^{1}H NMR (400 MHz, Chloroform-*d*): δ = 7.38 – 7.27 (m, 4H), 5.05 (s, 2H), 4.91 (s, 2H) ppm.
^{19}F NMR (376 MHz, Chloroform-*d*): δ = -57.31 ppm.
Physical data is in accordance with the literature.[12]

2-Bromo-*N*-(*tert*-butyl)-2,2-difluoroacetamide (3i)

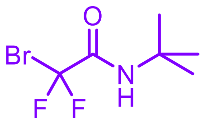

The titled compound was synthesized following the general procedure 7 (**GP 7**) and obtained after silica gel flash chromatography using *n*-pentane : diethyl ether (9:1) as a colorless solid (59% yield).
^{1}H NMR (400 MHz, Chloroform-*d*): δ = 5.95 (bs, 1H), 1.42 (s, 9H) ppm.
^{19}F NMR (376 MHz, Chloroform-*d*): δ = -60.26 ppm.
Physical data is in accordance with the literature.[7]

((Bromodifluoromethyl)sulfonyl)benzene (3j)

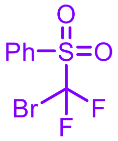

Synthesized according to the literature procedure.[13]
Step 1: To a suspension of NaH 60% (1.5 eq.) in anhydrous DMF, was slowly added thiophenol (1 eq.) at 0 °C over a period of 30 min. The mixture was cooled to -35 °C (internal temp.) 15 min before the addition of CF_2Br_2 (3 eq.). The reaction mixture was stirred for 3 h at -35 °C and 30 min from -35 °C to room temperature. The reaction flask was cooled in an ice-water bath and the excess of NaH was quenched by dropwise addition of water. The aqueous phase was extracted with Et_2O, the combined organic layers washed with water, brine, and dried over $MgSO_4$. Filtration and solvent evaporation left a crude product that was purified by distillation and bromodifluoromethylthiobenzene was obtained as colorless liquid (62% yield). Fractional distillation b.p. 97°C / 34 mmHg.
Step 2: To a solution of bromodifluoromethylthiobenzene (1 eq.) in dry DCM, was slowly added *m*-CPBA (3 eq.) at 0 °C. The reaction was stirred at room temperature for 24 h. The reaction mixture was concentrated, dissolved in ethylacetate and washed with 10% NaOH and brine. Combined organic phase were dried over $MgSO_4$. The solvent was removed in vacuo and the crude product was purified via silica gel column chromatography (pentane : ethylacetate 9:1) to obtain the pure product as colorless solid (87% yield).
^{1}H NMR (400 MHz, Chloroform-*d*): δ = 8.07 – 8.01 (m, 2H), 7.86 – 7.80 (m, 1H), 7.70 – 7.64 (m, 2H) ppm.
^{19}F NMR (376 MHz, Chloroform-*d*): δ = -57.56 ppm.
Physical data is in accordance with the literature.[13]

Procedure for the Synthesis of redox-active esters

Synthesized following the literature procedure.[14] In a flask containing a magnetic stirring bar, carboxylic acid (1.2 eq.), *N*-hydroxyphthalimide (1 eq.), and 4-dimethylaminopyridine (5 mol%) were added. CH_2Cl_2 (3 mL/mmol) was then added to the mixture, followed by the slow addition of a solution of *N*,*N*'-dicyclohexylcarbodiimide (1.2 eq.) in CH_2Cl_2 (1 mL/mmol) at room temperature. The reaction mixture was stirred at room temperature for 1–3 hours until the complete conversion of *N*-hydroxyphthalimide was observed. The white precipitate formed was filtered off, and the resulting solution was concentrated using a rotary evaporator. The crude product was then purified by flash column chromatography using a petroleum ether/ethyl acetate eluent system.

1,3-dioxoisoindolin-2-yl pivalate (24)

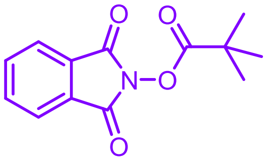

The titled compound was synthesized following the above procedure and obtained after silica gel flash chromatography using *n*-pentane : diethyl ether (10:1) as a colorless solid (68% yield).
^{1}H NMR (400 MHz, Chloroform-*d*): δ = 7.90 – 7.83 (m, 2H), 7.81 – 7.74 (m, 2H), 1.43 (s, 9H) ppm.
Physical data is in accordance with the literature.[14]

1,3-dioxoisoindolin-2-yl 2-phenylpropanoate (25)

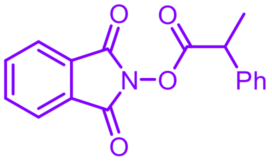

The titled compound was synthesized following the above procedure and obtained after silica gel flash chromatography using *n*-pentane : diethyl ether (10:1) as a colorless solid (72% yield).
^{1}H NMR (400 MHz, Chloroform-*d*): δ = 7.86 (dd, *J* = 5.5, 3.1 Hz, 2H), 7.77 (dd, *J* = 5.5, 3.1 Hz, 2H), 7.44 – 7.28 (m, 5H), 4.12 (q, *J* = 7.2 Hz, 1H), 1.68 (d, *J* = 7.2 Hz, 3H) ppm.
Physical data is in accordance with the literature.[15]

Proceudre for the synthesis of Katritzky salt

According to the literature procedure.[16] Amino acid methyl ester (1.0 eq.), 2,4,6-triphenylpyrylium tetrafluoroborate (1.0 eq.), and activated 4 Å molecular sieves (0.5 g/mmol) were added to a round-bottom flask, which was then sealed with a septum. CH_2Cl_2 (0.5 M) was added, followed by the addition of triethylamine (TEA, 2.0 eq.). The reaction mixture was stirred at room temperature for 30 minutes, after which acetic acid (2.0 eq.) was added. The mixture was stirred for 5 hours at room temperature, then filtered through a short Celite pad. The flask and Celite were rinsed with CH_2Cl_2, and the solvent was concentrated. The product was purified by silica gel chromatography using a CH_2Cl_2 (9:1) eluent system.

1-(1-Methoxy-1-oxo-3-phenylpropan-2-yl)-2,4,6-triphenylpyridin-1-ium tetrafluoroborate (26)

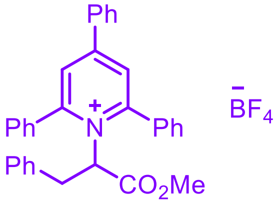

The titled compound was synthesized following the above procedure and obtained after silica gel flash chromatography using DCM : acetone (20:1 – 9:1) as a yellow solid (72% yield).
^{1}H NMR (400 MHz, Chloroform-*d*): δ = 7.97 (s, 2H), 7.85 (dd, *J* = 8.1, 1.6 Hz, 2H), 7.83 – 7.47 (m, 12H), 7.14 – 7.03 (m, 3H), 6.81 – 6.75 (m, 2H), 5.66 (dd, *J* = 8.2, 3.8 Hz, 1H), 3.68 (s, 3H), 3.51 (dd, *J* = 14.4, 3.7 Hz, 1H), 2.90 (dd, *J* = 14.4, 8.2 Hz, 1H) ppm.
Physical data is in accordance with the literature.[17]

Isopropyl 2-methyl-2-(4-(4-vinylbenzoyl)phenoxy)propanoate (5a)

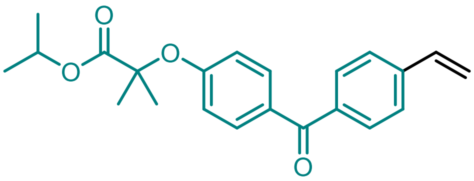

Synthesized according to the literature procedure.[18]
Fenofibrate (1 eq.), potassium vinyltrifluoroborate (1.75 eq.), palladium(II) chloride (5 mol%), RuPhos (10 mol%) and cesium carbonate (3 eq.) were added to a solution of in THF /water (7/1, 2.5 mL/mmol). The reaction mixture was degassed by nitrogen sparging for 5 min, and then stirred at 85 °C in oil bath for 48 h under nitrogen. More water was added, and the mixture was extracted three times with Et_2O. The combined organic fractions were washed once with water, once with brine and dried over anhydrous $MgSO_4$, concentrated in vacuum, and the crude product was purified by flash column

chromatography using pentane/EtOAc (15:1 to 9:1) to afford the product **5a** as colorless solid (91% yield).
^{1}H NMR (400 MHz, Chloroform-*d*): δ = 7.74 (t, *J* = 8.7 Hz, 4H), 7.49 (d, *J* = 8.3 Hz, 2H), 6.89 – 6.84 (m, 2H), 6.78 (dd, *J* = 17.6, 10.9 Hz, 1H), 5.88 (d, *J* = 17.6 Hz, 1H), 5.39 (d, *J* = 10.9 Hz, 1H), 5.09 (hept, *J* = 6.3 Hz, 1H), 1.66 (s, 6H), 1.20 (d, *J* = 6.3 Hz, 6H) ppm.
Physical data is in accordance with the literature.[13]

(8*R*,9*S*,13*S*,14*S*)-13-methyl-3-vinyl-6,7,8,9,11,12,13,14,15,16-decahydro-17*H*-cyclopenta[*a*]phenanthren-17-one (5b)

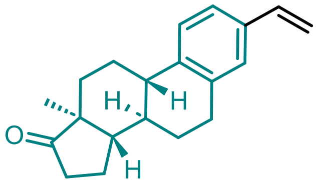

Synthesized according to the literature procedure.[19]
***Step 1*:** An oven-dried Schlenk flask was charged under nitrogen atmosphere with (+)-Estrone (1.0 eq.), triethylamine (2.0 eq.), and dry DCM (2.5 mL/mmol). The mixture was cooled to 0 °C, and triflic anhydride (1.1 eq.) was added dropwise via syringe. The reaction was stirred at 0 °C for 2 hours, after which it was poured into a saturated aqueous solution of $NaHCO_3$. The resulting biphasic mixture was extracted with DCM. The combined organic layers were dried over $MgSO_4$, and the solvent was removed under reduced pressure. The crude product was purified by column chromatography (*n*-pentane : EtOAc, 4:1) to yield 3-(trifluoromethanesulfonyl)estrone (61%) as a colorless solid.
Step 2: The Schlenk flask was then charged with 3-(trifluoromethanesulfonyl)estrone (1.0 eq.), SPhos (0.1 eq.), K_3PO_4 (3.0 eq.), 4,4,5,5-tetramethyl-2-vinyl-1,3,2-dioxaborolane (2.0 eq.), 1,4-dioxane (7.5 ml), and water (1.0 mL/mmol). The reaction vessel was sealed with a septum, evacuated, and refilled with nitrogen (repeated 3 times). $Pd(OAc)_2$ (5 mol%) was added under nitrogen flow, and the reaction mixture was heated to 80 °C and stirred for 22 hours. After cooling to room temperature, the reaction mixture was diluted with EtOAc and filtered through a plug of silica. The organic phase was washed with brine dried over $MgSO_4$, and the solvent was removed under reduced pressure. The residue was purified by column chromatography (*n*-pentane : ethylacetate, 15:1) to yield the product **5b** (86% yield) as a colorless solid.
^{1}H NMR (400 MHz, Chloroform-*d*): δ = 7.27 – 7.17 (m, 2H), 7.14 – 7.11 (m, 1H), 6.65 (dd, *J* = 17.6, 10.9 Hz, 1H), 5.69 (d, *J* = 17.6 Hz, 1H), 5.18 (d, *J* = 10.9 Hz, 1H), 2.90 (dd, *J* = 9.0, 4.3 Hz, 1H), 2.54 – 2.37 (m, 2H), 2.33 – 2.22 (m, 1H), 2.19 – 1.92 (m, 4H), 1.67 – 1.36 (m, 7H), 0.89 (s, 3H) ppm.
Physical data is in accordance with the literature.[19]

(*R*)-2,8-dimethyl-2-((4*R*,8*R*)-4,8,12-trimethyltridecyl)-6-vinylchromane (5c)

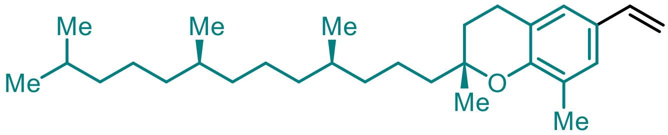

Synthesized according to the literature procedure.[20]
Step 1: To a solution of *δ*-tocopherol (1 eq.) in anhydrous DCM (6.5 mL/mmol), triethylamine (2 eq.) and trifluoromethanesulfonic anhydride (1.2 eq.) were added dropwise at 0 °C. The resulting brown mixture was gradually warmed to room temperature and stirred for 5 hours. The reaction was quenched by adding a saturated aqueous solution of $NaHCO_3$, and the crude product was extracted with DCM. The combined organic layers were dried over $MgSO_4$. After evaporation of the solvent, the concentrate was purified by silica gel column chromatography (*n*-pentane : ethylacetate, 20:1), yielding the desired trifluoromethanesulfonyl-substituted tocopherol derivative as a colorless oil (81% yield).

Step 2: A mixture of trifluoromethanesulfonyl-substituted tocopherol derivative (1 eq.), potassium trifluoroborate (2 eq.), palladium(II) chloride (10 mol%), RuPhos (20 moL%), and cesium carbonate (3 eq.) was placed in a round-bottom flask. The flask was evacuated and filled with nitrogen three times. To the flask, 2.8 mL/mmol of a THF/H_2O (8:1) mixture was added, and the resulting dark brown mixture was stirred at 85 °C for 24 hours. After cooling, additional water was added, and the crude product was extracted with diethyl ether. The combined organic layers were dried over $MgSO_4$. After solvent evaporation, the crude product was purified by silica gel column chromatography(*n*-pentane : ethylacetate, 50:1), yielding the desired vinyl derivative of tocopherol **5c** as a colorless oil (87% yield).
^{1}H NMR (400 MHz, Chloroform-*d*): δ = 7.05 (d, *J* = 2.3 Hz, 1H), 6.95 (d, *J* = 2.2 Hz, 1H), 6.59 (dd, *J* = 17.6, 10.9 Hz, 1H), 5.59 – 5.50 (m, 1H), 5.06 – 5.01 (m, 1H), 2.78 – 2.70 (m, 2H), 2.16 (s, 3H), 1.86 – 1.69 (m, 2H), 1.64 – 0.97 (m, 24H), 0.89 – 0.82 (m, 12H) ppm.
Physical data is in accordance with the literature.[20]

Optimization of the chiral thiazolium carbene catalyzed asymmetric acyl-difluoroalkylation of olefins

Table S1: Catalyst screening

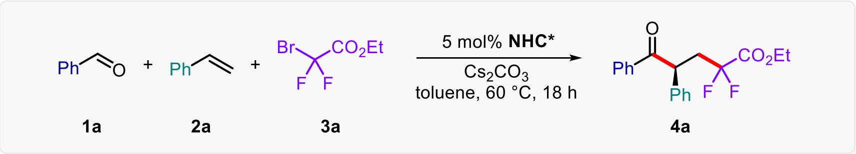

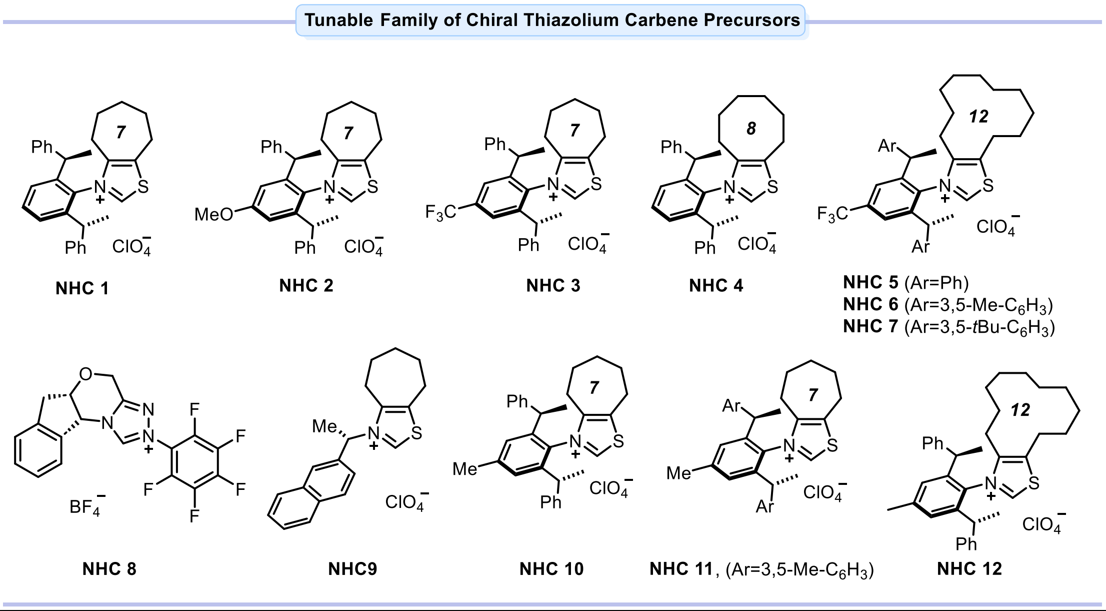

Entry[a]	NHC*	% Yield 4a[b]	e.r. 4a[c]
1	**NHC1**	83[d]	71:29
2	**NHC2**	81[d]	70:30
3	**NHC3**	76[d]	75:25
4	**NHC4**	79	76:24
5	**NHC5**	74	88:12
6	**NHC6**	72[d]	90:10
7	**NHC7**	70[d]	93:7
8	**NHC8**	27	56:44
9	**NHC9**	11	53:47
10	**NHC10**	82	70:30
11	**NHC11**	77	73:27
12	**NHC12**	78	82:18

[a] Conditions: 0.1 mmol **1a**, 0.15 mmol **2a**, 0.2 mmol **3a**, 5 μmol **NHC***, 0.15 mmol Cs_2CO_3 in 1mL toluene at 60 °C for 18 h. [b] ^{1}H-NMR yield using 1,3,5-trimethoxybenzene as an internal standard. [c] Determined by chiral HPLC. [d] Isolated yield.

Table S2: Solvent screening

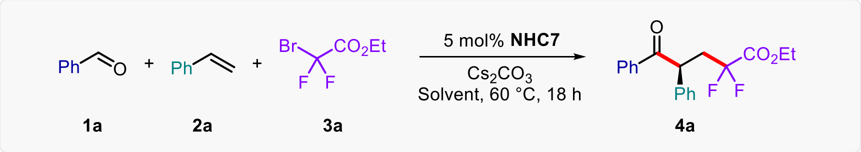

Entry[a]	Solvent	% Yield 4a[b]	e.r. 4a[c]
1	**Toluene**	70[d]	93:7
2	**DCM**	76	84:16
3	**DMSO**	74	50:50
4	**THF**	62	90:10
5	**1,4-Dioxane**	71	89:11
6	***p*-Xylene**	63	91:9
7	**$PhCF_3$**	51	85:15
8	**CPME**	81	95:5
9	**DIPE**	76	94.5:5.5
10	**MTBE**	79	98:2

[a] Conditions: 0.1 mmol **1a**, 0.15 mmol **2a**, 0.2 mmol **3a**, 5 µmol **NHC7**, 0.15 mmol Cs_2CO_3 in 1mL solvent at 60 °C for 18 h. [b] ^{1}H-NMR yield using 1,3,5-trimethoxybenzene as an internal standard. [c] Determined by chiral HPLC. [d] Isolated yield. DCM – Dichloromethane; DMSO – Dimethylsulfoxide; THF – Tetrahydrofuran; CPME - Cyclopentyl methyl ether; DIPE - Diisopropyl ether; MTBE - Methyl *tert*-butyl ether.

Table S3: Base screening

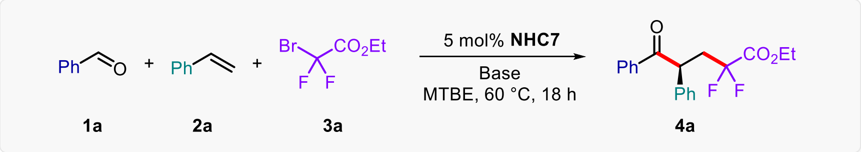

Entry[a]	Base	% Yield 4a[b]	e.r. 4a[c]
1	**Cs_2CO_3**	79[d]	98:2
2	**K_2CO_3**	74[d]	98:2
3	**Na_2CO_3**	trace	*n.d.*
4	**CsOAc**	70	97:3
4	**KOAc**	73	98:2
5	**DBU**	55	90:10
6	**DIPEA**	53	91:9
7	**DABCO**	49	88:12
8	**Et3N**	67	89:11
9	**Cs_2CO_3 (1.0 eq.)**[e]	71	98:2
10	**Cs_2CO_3 (2.0 eq.)**[e]	78	97:3

[a] Conditions: 0.1 mmol **1a**, 0.15 mmol **2a**, 0.2 mmol **3a**, 5 µmol **NHC7**, 0.15 mmol base in 1mL MTBE at 60 °C for 18 h. [b] ^{1}H-NMR yield using 1,3,5-trimethoxybenzene as an internal standard. [c] Determined by chiral HPLC. [d] Isolated yield. [e] Different base stoichiometries.

Table S4: Time screening

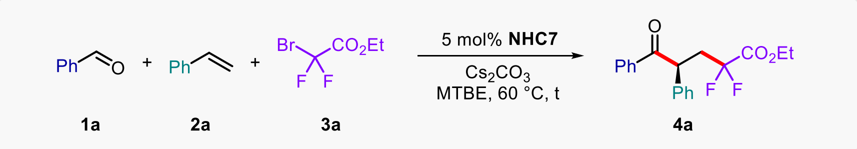

Entry[a]	time (h)	% Yield 4a[b]	e.r. 4a[c]
1	**18**	79[d]	98:2
2	**24**	83[d]	98:2
3	**30**	83	98:2

[a] Conditions: 0.1 mmol **1a**, 0.15 mmol **2a**, 0.2 mmol **3a**, 5 µmol **NHC7**, 0.15 mmol Cs_2CO_3 in 1mL MTBE at 60 °C for t h. [b] ^{1}H-NMR yield using 1,3,5-trimethoxybenzene as an internal standard. [c] Determined by chiral HPLC. [d] Isolated yield.

Table S5: Temperature screening

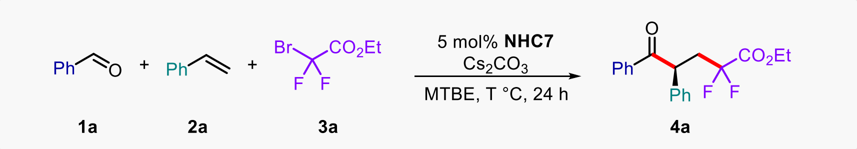

Entry[a]	Temeprature (°C)	% Yield 4a[b]	e.r. 4a[c]
1	**60**	83[d]	98:2
2	**40**	71	99:1
3	**70**	85	97.5:3.5

[a] Conditions: 0.1 mmol **1a**, 0.15 mmol **2a**, 0.2 mmol **3a**, 5 µmol **NHC7**, 0.15 mmol Cs_2CO_3 in 1mL MTBE at T °C for 24 h. [b] ^{1}H-NMR yield using 1,3,5-trimethoxybenzene as internal standard. [c] Determined by chiral HPLC. [d] Isolated yield.

Table S6: Stoichiometry screening

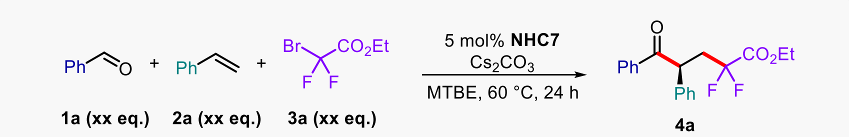

Entry[a]	1a (xx eq.)	2a (xx eq.)	3a (xx eq.)	% Yield 4a[b]	e.r. 4a[c]
1	**1**	**1.5**	**2**	83[d]	98:2
2	**1.5**	**1**	**2**	61	97:3
3	**1**	**1.5**	**1**	55	98:2
4	**1**	**2**	**2**	80	98:2
5	**1**	**1.5**	**3**	84	98:2
6[e]	**1**	**1.5**	**2**	86	98:2

[a] Conditions: xx mmol **1a**, xx mmol **2a**, xx mmol **3a**, 5 µmol **NHC7**, 0.15 mmol Cs_2CO_3 in 1mL MTBE at 60 °C for 24 h. [b] ^{1}H-NMR yield using 1,3,5-trimethoxybenzene as an internal standard. [c] Determined by chiral HPLC. [d] Isolated yield. [e] 10 mol% of **NHC7** loading.

Table S7: Concentration screening

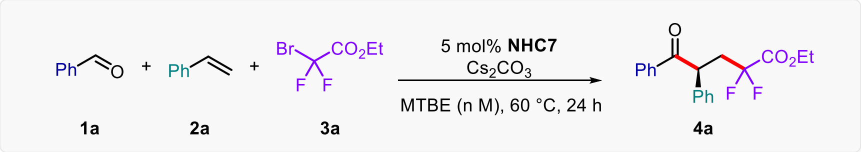

Entry[a]	MTBE (mL)	Concentration (n M)	% Yield 4a[b]	e.r. 4a[c]
1	**1**	0.1	83[d]	98:2
2	**0.5**	0.2	85	96:2
3	**2**	0.05	81	98:2

[a] Conditions: 0.1 mmol **1a**, 0.15 mmol **2a**, 0.2 mmol **3a**, 5 µmol **NHC7**, 0.15 mmol Cs_2CO_3 in *n* mL MTBE at 60 °C for 24 h. [b] ^{1}H-NMR yield using 1,3,5-trimethoxybenzene as an internal standard. [c] Determined by chiral HPLC. [d] Isolated yield.

Catalysis procedures

General procedure for the thiazolium carbene-catalyzed three-component acyl-difluoroalkylation reaction (GP 8)

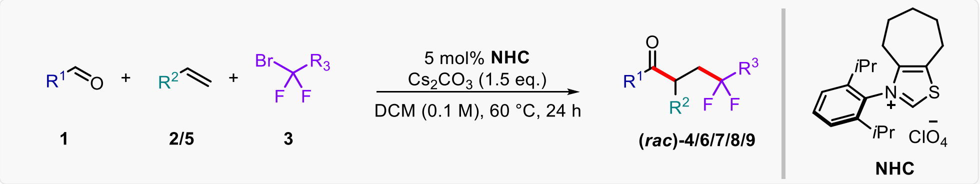

According to the literature procedure.[21]

In a nitrogen-filled glove box, an oven-dried 5 mL reaction tube was charged with the thiazolium salt **NHC** (2.0 mg, 0.005 mmol, 5 mol%). Dry, degassed DCM (1 mL) was then added. Subsequently, aldehyde **1** (0.1 mmol, 1 eq.), olefin **2** or **5** (0.15 mmol, 1.5 eq.), difluoroalkyl bromide **3** (0.2 mmol, 2 eq.), and Cs_2CO_3 (49 mg, 0.15 mmol, 1.5 eq.) were added to the reaction tube, which was then sealed with a crimper. The reaction tube was removed from the glove box and stirred at 60 °C for 24 hours. Afterward, the reaction mixture was filtered through a short silica gel pad (2 cm), and the solvent was evaporated under reduced pressure. The crude product mixture was purified by silica gel column chromatography using an *n*-pentane : ethylacetate mixture as the eluent to obtain the racemic mixture of desired product.

General procedure for the chiral thiazolium carbene-catalyzed asymmetric acyl-difluoroalkylation reaction of olefins (GP 9)

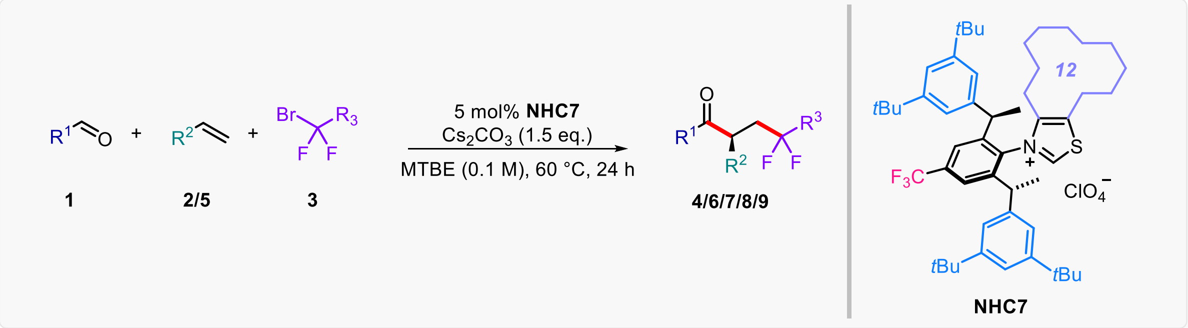

In a nitrogen-filled glove box, an oven-dried 5 mL reaction tube was charged with chiral thiazolium salt **NHC7** (4.5 mg, 5 mol%). Then, 1 mL of dry and degassed methyl *tert*-butyl ether (MTBE) was added. Subsequently, aldehyde **1** (0.1 mmol, 1 eq.), olefin **2** or **5** (0.15 mmol, 1.5 eq.), difluoroalkyl bromide **3** (0.2 mmol, 2 eq.), and Cs_2CO_3 (49 mg, 0.15 mmol, 1.5 eq.) were added to the reaction tube, which was then sealed with a crimper. The reaction tube was removed from the glove box and stirred at

60 °C for 24 hours. The reaction mixture was then filtered through a short pad of silica gel (4 cm), and the solvent was evaporated under reduced pressure. The crude product mixture was purified by silica gel column chromatography using *n*-pentane : ethylacetate mixture as eluent to obtain corresponding enantioenriched β-difluoroalkylated α-chiral ketones (**4**/**6**/**7**/**8**/**9**).

Physical data

Ethyl (*S*)-2,2-difluoro-5-oxo-4,5-diphenylpentanoate (4a)

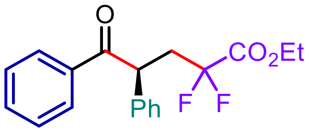

The title compound was synthesized according to the general procedure (**GP 9**) and was obtained after silica gel column chromatography (*n*-pentane : ethylacetate 20:1) as a colorless oil (83 % Yield, 28 mg, 96 % ee). **R_f** = 0.22 (*n*-pentane : ethylacetate 20:1).
^{1}H NMR (400 MHz, Chloroform-*d*): δ = 7.96 – 7.90 (m, 2H), 7.50 – 7.43 (m, 1H), 7.40 – 7.33 (m, 2H), 7.30 – 7.23 (m, 4H), 7.23 – 7.16 (m, 1H), 4.93 (dd, *J* = 8.0, 5.0 Hz, 1H), 4.15 (dq, *J* = 10.7, 7.2 Hz, 1H), 4.03 (dq, *J* = 10.8, 7.1 Hz, 1H), 3.33 – 3.17 (m, 1H), 2.45 – 2.60 (m, 1H), 1.20 (t, *J* = 7.2 Hz, 3H) ppm.
^{13}C NMR (101 MHz, Chloroform-*d*): δ = 197.7, 164.2 (t, *J* = 32.5 Hz), 138.2, 136.2, 133.6, 129.6, 129.2, 129.0, 128.7, 128.1, 115.7 (t, *J* = 250.4 Hz), 63.3, 47.3 (t, *J* = 3.8 Hz), 38.6 (t, *J* = 23.3 Hz), 14.1 ppm.
^{19}F NMR (376 MHz, $CDCl_3$): δ = -103.95 (dt, *J* = 260.9, 16.2 Hz), -104.85 (dt, *J* = 260.9, 16.2 Hz) ppm.
HRMS (APCI/QTOF): *m/z*: $[M + H]^+$ Calcd for $C_{19}H_{19}F_2O_3^+$: 333.1297; Found 333.1294.
IR (ATR): 1762, 1682, 1597, 1429, 1207, 1002, 656 cm^{-1}.
$[\alpha]_D^{20}$ = +61.2 (c = 0.5, $CHCl_3$).
Chiral HPLC: (Chiralpak IC, 1 % *i*PrOH/hexane, 1.0 mL/min, 210 nm): tR (minor) 10.27 min, tR (major) 13.93 min, 98:2 *er.*

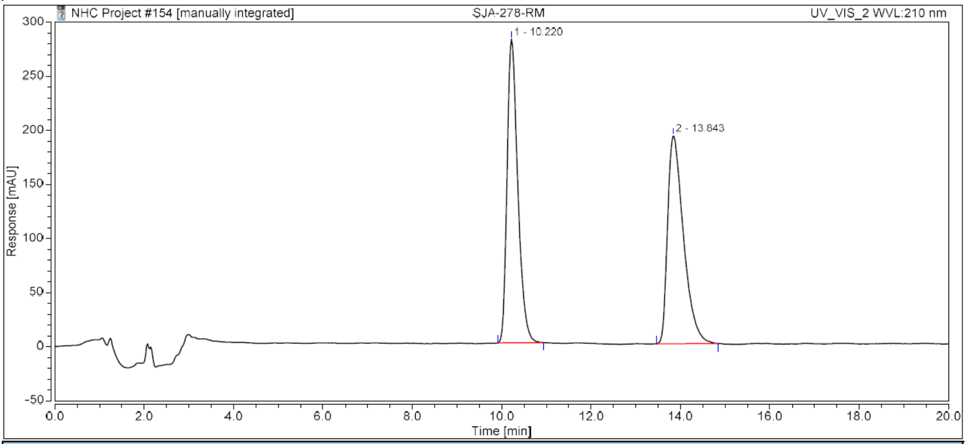

Integration Results

No.	Peak Name	Retention Time min	Area mAU*min	Height mAU	Relative Area %	Relative Height %	Amount
1		10.220	78.021	280.899	49.69	59.38	n.a.
2		13.843	78.985	192.176	50.31	40.62	n.a.
**Total:**			**157.006**	**473.075**	**100.00**	**100.00**	

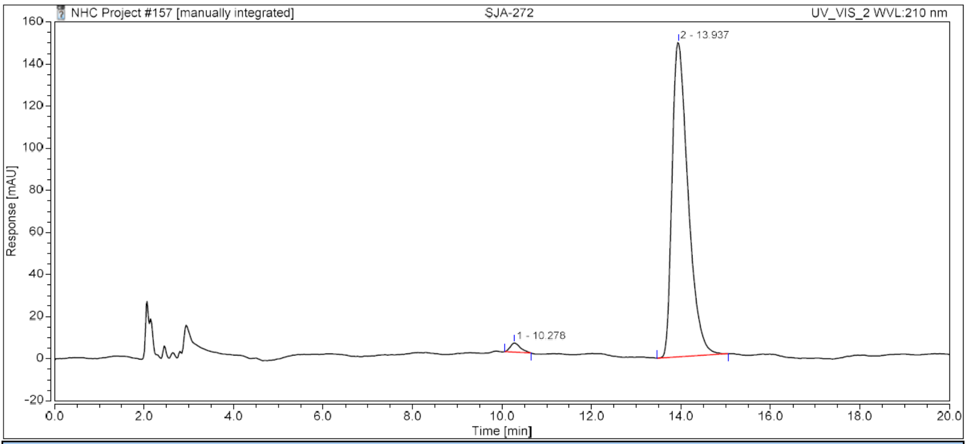

Integration Results							
No.	Peak Name	Retention Time min	Area mAU*min	Height mAU	Relative Area %	Relative Height %	Amount n.a.
1		10.278	1.133	4.350	1.80	2.83	n.a.
2		13.937	61.848	149.465	98.20	97.17	n.a.
**Total:**			**62.981**	**153.814**	**100.00**	**100.00**	

Ethyl (*S*)-2,2-difluoro-5-(4-fluorophenyl)-5-oxo-4-phenylpentanoate (4b)

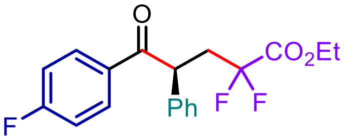

The title compound was synthesized according to the general procedure (**GP 9**) and was obtained after silica gel column chromatography (*n*-pentane : ethylacetate 30:1) as a colorless oil (77 % Yield, 27 mg, 96 % ee). $\mathbf{R_f}$ = 0.20 (*n*-pentane : ethylacetate 30:1).

^{1}H NMR (400 MHz, Chloroform-*d*): δ = 8.01 – 7.95 (m, 2H), 7.33 – 7.20 (m, 5H), 7.10 – 7.03 (m, 2H), 4.90 (dd, *J* = 8.1, 4.9 Hz, 1H), 4.23 – 4.13 (m, 1H), 4.12 – 4.03 (m, 1H), 3.35 – 3.18 (m, 1H), 2.59 – 2.54 (m, 1H), 1.24 (t, *J* = 7.1 Hz, 3H) ppm.

^{13}C NMR (101 MHz, Chloroform-*d*): δ = 195.8, 165.8 (d, *J* = 255.3 Hz), 163.9 (t, *J* = 32.7 Hz), 137.8, 132.3 (d, *J* = 3.2 Hz), 131.6 (d, *J* = 9.2 Hz), 129.4, 128.3, 127.9, 115.9 (d, *J* = 22.0 Hz), 115.3 (t, *J* = 250.7 Hz), 63.0, 47.0 (t, *J* = 3.8 Hz), 38.2 (t, *J* = 23.3 Hz), 13.9 ppm.

^{19}F NMR (376 MHz, $CDCl_3$): δ = -104.06 (dt, *J* = 258.4, 16.2 Hz),-104.64 - -104.73 (m), -104.82 (dt, *J* = 258.4, 16.2 Hz) ppm.

HRMS (ESI/QTOF): *m/z*: $[M + Na]^+$ Calcd. for $C_{19}H_{17}F_3NaO_3^+$: 373.1022; Found 373.1028.

IR (ATR): 1764, 1684, 1560, 1409, 1373, 970, 896, 685 cm^{-1}.

$[\alpha]_D^{20}$ = +58.2 (c = 0.5, $CHCl_3$).

Chiral HPLC: (Chiralpak IC, 1 % *i*PrOH/hexane, 1.0 mL/min, 210 nm): tR (minor) 8.63 min, tR (major) 12.01 min, 98:2*er.*

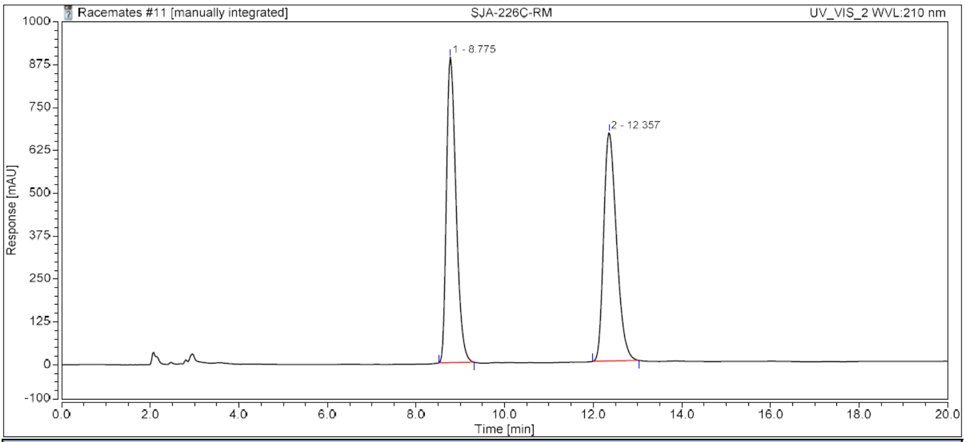

Integration Results

No.	Peak Name	Retention Time min	Area mAU*min	Height mAU	Relative Area %	Relative Height %	Amount n.a.
1		8.775	220.597	891.743	49.59	57.22	n.a.
2		12.357	224.219	666.704	50.41	42.78	n.a.
**Total:**			**444.816**	**1558.447**	**100.00**	**100.00**	

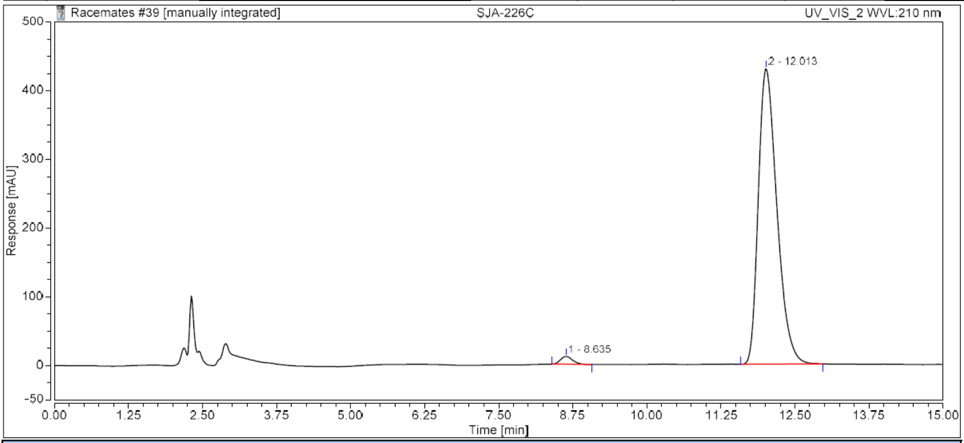

Integration Results

No.	Peak Name	Retention Time min	Area mAU*min	Height mAU	Relative Area %	Relative Height %	Amount n.a.
1		8.635	2.665	11.342	1.67	2.57	n.a.
2		12.013	156.609	430.302	98.33	97.43	n.a.
**Total:**			**159.274**	**441.644**	**100.00**	**100.00**	

Ethyl (*S*)-5-(4-chlorophenyl)-2,2-difluoro-5-oxo-4-phenylpentanoate (4c)

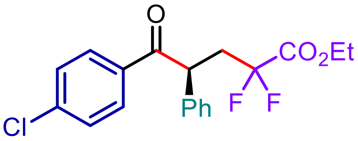

The title compound was synthesized according to the general procedure (**GP 9**) and was obtained after silica gel column chromatography (*n*-pentane : ethylacetate 20:1) as a colorless oil (79 % Yield, 29 mg, 99% ee). **R_f** = 0.20 (*n*-pentane : ethylacetate 20:1).

^{1}H NMR (400 MHz, $CDCl_3$): δ = 7.89 – 7.85 (m, 2H), 7.37 – 7.33 (m, 2H), 7.31 – 7.19 (m, 5H), 4.88 (dd, *J* = 8.1, 4.9 Hz, 1H), 4.17 (dq, *J* = 10.8, 7.2 Hz, 1H), 4.06 (dq, *J* = 10.8, 7.1 Hz, 1H), 3.33 – 3.17 (m, 1H), 2.58 – 2.43 (m, 1H), 1.23 (t, *J* = 7.2 Hz, 3H) ppm.

^{13}C NMR (101 MHz, $CDCl_3$): δ = 196.2, 163.8 (t, *J* = 32.5 Hz), 139.8, 137.6, 134.2, 130.3, 129.4, 129.1, 128.3, 128.0, 115.3 (t, *J* = 250.7 Hz), 63.1, 47.1 (t, *J* = 3.9 Hz), 38.2 (t, *J* = 23.3 Hz), 13.9 ppm.

^{19}F NMR (376 MHz, $CDCl_3$): δ = -104.01 (dt, *J* = 259.5, 16.6 Hz), -104.85 (dt, *J* = 259.5, 16.6 Hz) ppm.

HRMS (ESI/QTOF): *m/z*: $[M + Na]^+$ Calcd. for $C_{19}H_{17}ClF_2NaO_3^+$: 389.0726; Found 389.0709.

IR (ATR): 1764, 1684, 1588, 1571, 1400, 1306, 1093, 831, 638 cm^{-1}.

$[\alpha]_D^{20}$ = +117.3 (c = 0.5, $CHCl_3$).

Chiral HPLC: (Chiralpak IC, 1 % *i*PrOH/hexane, 1.0 mL/min, 210 nm): tR (minor) 8.62 min, tR (major) 11.84 min, 99.5:0.5 *er*.

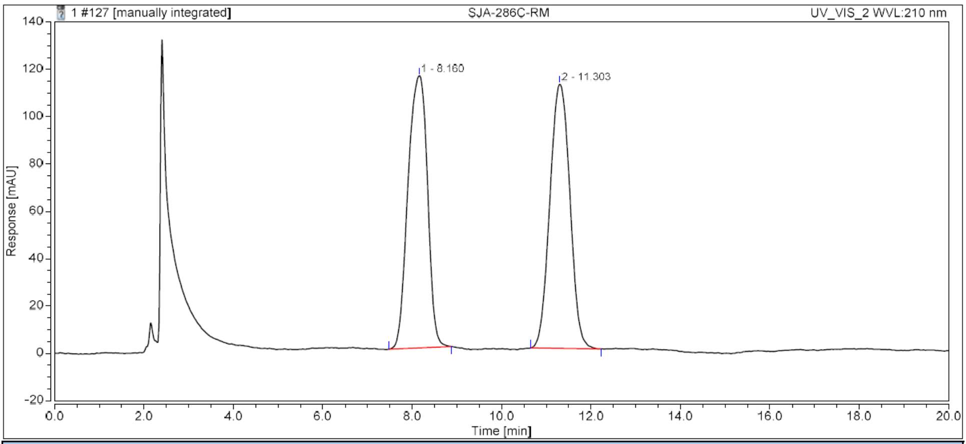

Integration Results

No.	Peak Name	Retention Time min	Area mAU*min	Height mAU	Relative Area %	Relative Height %	Amount n.a.
1		8.160	57.912	115.041	50.04	50.76	n.a.
2		11.303	57.817	111.613	49.96	49.24	n.a.
Total:			115.730	226.654	100.00	100.00	

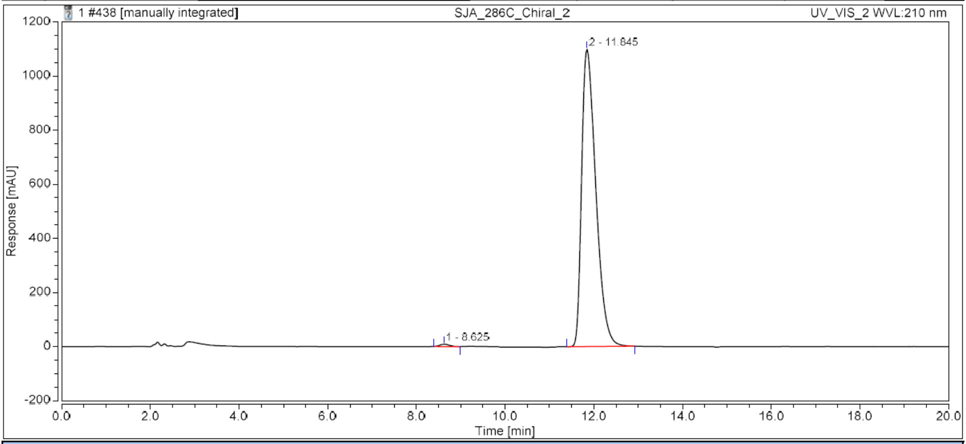

Integration Results

No.	Peak Name	Retention Time min	Area mAU*min	Height mAU	Relative Area %	Relative Height %	Amount n.a.
1		8.625	2.257	8.785	0.55	0.79	n.a.
2		11.845	404.562	1099.387	99.45	99.21	n.a.
Total:			406.820	1108.172	100.00	100.00	

Ethyl (*S*)-2,2-difluoro-5-(4-iodophenyl)-5-oxo-4-phenylpentanoate (4d)

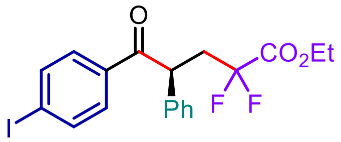

The title compound was synthesized according to the general procedure (**GP 9**) and was obtained after silica gel column chromatography (*n*-pentane : ethylacetate 30:1) as a colorless solid (80 % Yield, 37 mg, 96 % ee). $\mathbf{R_f}$ = 0.26 (*n*-pentane : ethylacetate 30:1).

MP: 178-179 °C

^{1}**H NMR** (400 MHz, Chloroform-*d*): δ = 7.71 – 7.64 (m, 2H), 7.60 – 7.54 (m, 2H), 7.26 – 7.11 (m, 5H), 4.79 (dd, *J* = 8.0, 4.9 Hz, 1H), 4.10 (dq, *J* = 10.7, 7.2 Hz, 1H), 4.00 (dq, *J* = 10.7, 7.1 Hz, 1H), 3.27 – 3.11 (m, 1H), 2.52 – 2.36 (m, 1H), 1.17 (t, *J* = 7.2 Hz, 3H) ppm.

^{13}**C NMR** (101 MHz, Chloroform-*d*): δ = 196.7, 163.8 (t, *J* = 32.5 Hz), 138.0, 137.5, 135.1, 130.3, 129.4, 128.3, 128.0, 115.3 (t, *J* = 250.8 Hz), 101.4, 63.1, 47.0 (t, *J* = 3.9 Hz), 38.1 (t, *J* = 23.4 Hz), 13.9 ppm.

^{19}**F NMR** (376 MHz, Chloroform-*d*): δ = -104.01 (dt, *J* = 257.6, 16.7 Hz), -104.79 (dt, *J* = 257.6, 16.7 Hz) ppm.

HRMS (ESI/QTOF): *m/z*: $[M + Na]^+$ Calcd for $C_{19}H_{17}F_2INaO_3^+$: 481.0083; Found 481.0073.

IR (ATR): 1765, 1684, 1600, 1580, 1561, 1428, 1261, 1029, 775, 498 cm^{-1}.

$[\alpha]_D^{20}$ = +69.3 (c = 0.25, $CHCl_3$).

Chiral HPLC: (Chiralpak IC, 1 % *i*PrOH/hexane, 1.0 mL/min, 210 nm): tR (minor) 10.19 min, tR (major) 13.99 min, 98:2 *er.*

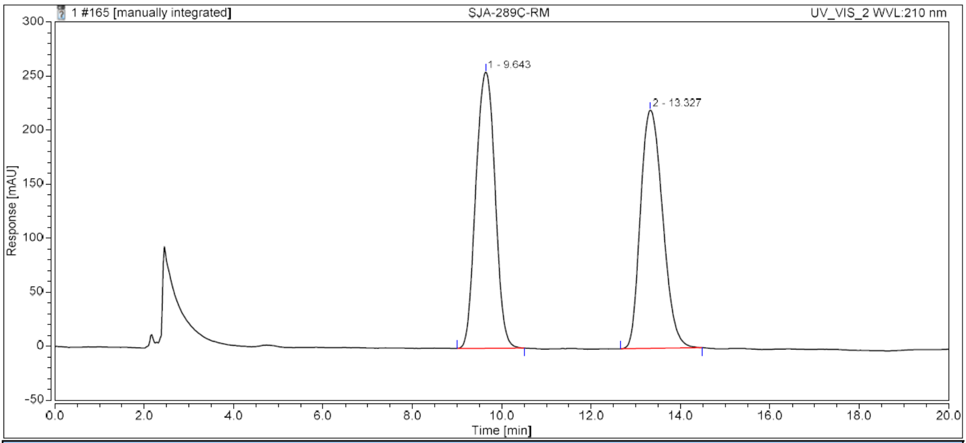

Integration Results

No.	Peak Name	Retention Time min	Area mAU*min	Height mAU	Relative Area %	Relative Height %	Amount n.a.
1		9.643	127.161	255.626	50.05	53.69	n.a.
2		13.327	126.906	220.461	49.95	46.31	n.a.
Total:			254.067	476.086	100.00	100.00	

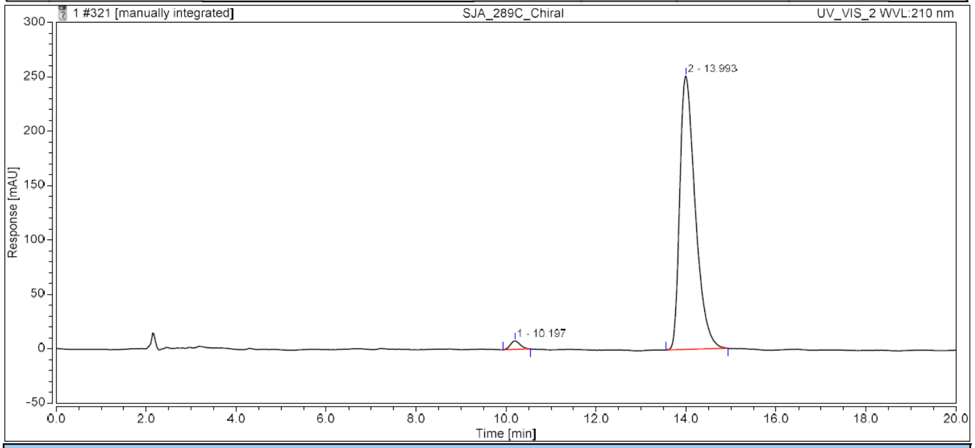

Integration Results

No.	Peak Name	Retention Time min	Area mAU*min	Height mAU	Relative Area %	Relative Height %	Amount n.a.
1		10.197	1.952	7.797	1.91	3.01	n.a.
2		13.993	100.228	251.622	98.09	96.99	n.a.
Total:			102.181	259.419	100.00	100.00	

Ethyl (*S*)-2,2-difluoro-5-(4-methoxyphenyl)-5-oxo-4-phenylpentanoate (4e)

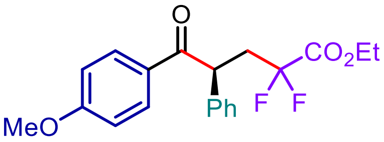

The title compound was synthesized according to the general procedure (**GP 9**) and was obtained after silica gel column chromatography (*n*-pentane : ethylacetate 9:1) as a colorless oil (86 % Yield, 31 mg, 98 % ee). $\mathbf{R_f}$ = 0.24 (*n*-pentane : ethylacetate 9:1).

^{1}**H NMR** (400 MHz, Chloroform-*d*): δ = 7.94 – 7.88 (m, 2H), 7.27 – 7.21 (m, 4H), 7.21 – 7.15 (m, 1H), 6.86 – 6.81 (m, 2H), 4.86 (dd, *J* = 7.9, 5.1 Hz, 1H), 4.17 – 4.08 (m, 1H), 4.05 – 3.95 (m, 1H), 3.78 (s, 3H), 3.30 – 3.13 (m, 1H), 2.57 – 2.42 (m, 1H), 1.19 (t, *J* = 7.2 Hz, 3H) ppm.

^{13}**C NMR** (101 MHz, Chloroform-*d*): δ = 195.8, 163.9 (t, *J* = 32.5 Hz), 163.7, 138.4, 131.3, 129.3, 128.8, 128.3, 127.72, 115.5 (t, *J* = 251 Hz), 113.9, 63.0, 55.5, 46.6 (t, *J* = 4.0 Hz), 38.3 (t, *J* = 23.3 Hz), 13.8 ppm.

^{19}**F NMR** (376 MHz, Chloroform-*d*): δ = -103.59 – -105.14 (m) ppm.

HRMS (ESI/QTOF): *m/z*: $[M + H]^+$ Calcd. for $C_{20}H_{21}F_2O_4^+$: 363.1402; Found 363.1408.

IR (ATR): 1765, 1673, 1599, 1259, 1168, 780, 742 cm^{-1}.

$[\alpha]_D^{20}$ = +80.1 (c = 1, $CHCl_3$).

Chiral HPLC: (Chiralpak IC, 3 % *i*PrOH/hexane, 1.0 mL/min, 210 nm): tR (minor) 11.76 min, tR (major) 19.06 min, 99:1 *er*.

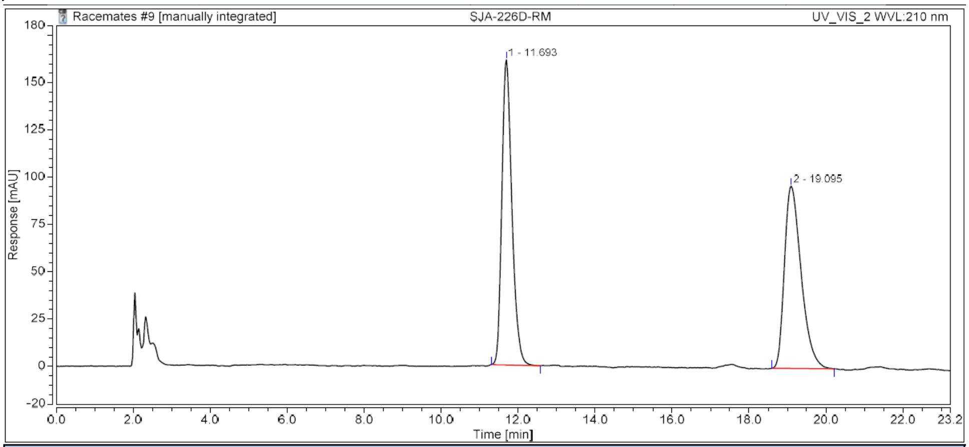

Integration Results							
No.	Peak Name	Retention Time min	Area mAU*min	Height mAU	Relative Area %	Relative Height %	Amount n.a.
1		11.693	48.424	161.306	50.05	62.59	n.a.
2		19.095	48.322	96.405	49.95	37.41	n.a.
Total:			96.746	257.711	100.00	100.00	

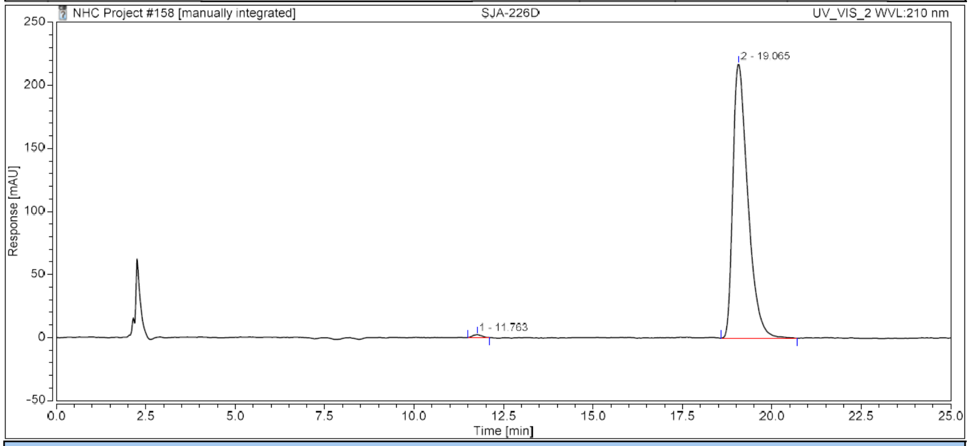

Integration Results							
No.	Peak Name	Retention Time min	Area mAU*min	Height mAU	Relative Area %	Relative Height %	Amount n.a.
1		11.763	0.684	2.389	0.64	1.09	n.a.
2		19.065	105.436	217.558	99.36	98.91	n.a.
Total:			106.120	219.947	100.00	100.00	

Methyl (*S*)-4-(5-ethoxy-4,4-difluoro-5-oxo-2-phenylpentanoyl)benzoate (4f)

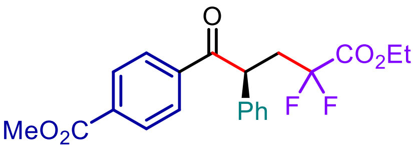

The title compound was synthesized according to the general procedure (**GP 9**) and was obtained after silica gel column chromatography (*n*-pentane : ethylacetate 9:1) as a colorless oil (75 % Yield, 29 mg, 99 % ee). **R**$_f$ = 0.26 (*n*-pentane : ethylacetate 9:1).

MP: 137-139 °C

^{1}H NMR (400 MHz, $CDCl_3$): δ = 8.07 – 8.02 (m, 2H), 8.00 – 7.96 (m, 2H), 7.33 – 7.20 (m, 5H), 4.95 (dd, *J* = 8.0, 4.9 Hz, 1H), 4.19 (dq, *J* = 10.7, 7.1 Hz, 1H), 4.08 (dq, *J* = 10.7, 7.1 Hz, 1H), 3.92 (s, 3H), 3.36 – 3.21 (m, 1H), 2.63 – 2.45 (m, 1H), 1.25 (t, *J* = 7.1 Hz, 3H) ppm.

^{13}C NMR (101 MHz, $CDCl_3$): δ = 196.9, 166.1, 163.7 (t, *J* = 32.4 Hz), 139.1, 137.2, 133.9, 129.8, 129.3, 128.7, 128.2, 127.9, 115.1 (t, *J* = 250.6 Hz), 63.0, 52.4, 47.3 (t, *J* = 4.0 Hz), 38.0 (t, *J* = 23.3 Hz), 13.7 ppm.

^{19}F NMR (376 MHz, $CDCl_3$): δ = -104.00 (dt, *J* = 260.3, 16.6 Hz), -104.85 (dt, *J* = 260.3, 16.6 Hz) ppm.

HRMS (ESI/QTOF): *m/z*: [M + Na]$^+$ Calcd. for $C_{21}H_{20}F_2NaO_5^+$: 413.1171; Found 413.1173.

IR (ATR): 1766, 1726, 1688, 1601, 1406, 1107, 857, 539, 427 cm^{-1}.

$[\alpha]_D^{20}$ = +86.2 (c = 2, $CHCl_3$).

Chiral HPLC: (Chiralpak IC, 10 % *i*PrOH/hexane, 1.0 mL/min, 210 nm): tR (major) 7.96 min, tR (minor) 9.41 min, >99.5:0.5 *er.*

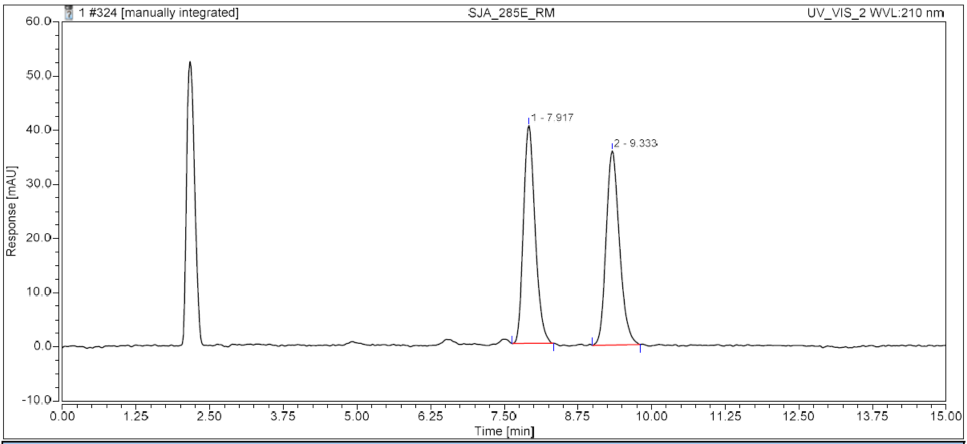

Integration Results							
No.	Peak Name	Retention Time min	Area mAU*min	Height mAU	Relative Area %	Relative Height %	Amount n.a.
1		7.917	9.245	40.289	49.50	52.90	n.a.
2		9.333	9.430	35.865	50.50	47.10	n.a.
**Total:**			**18.675**	**76.153**	**100.00**	**100.00**	

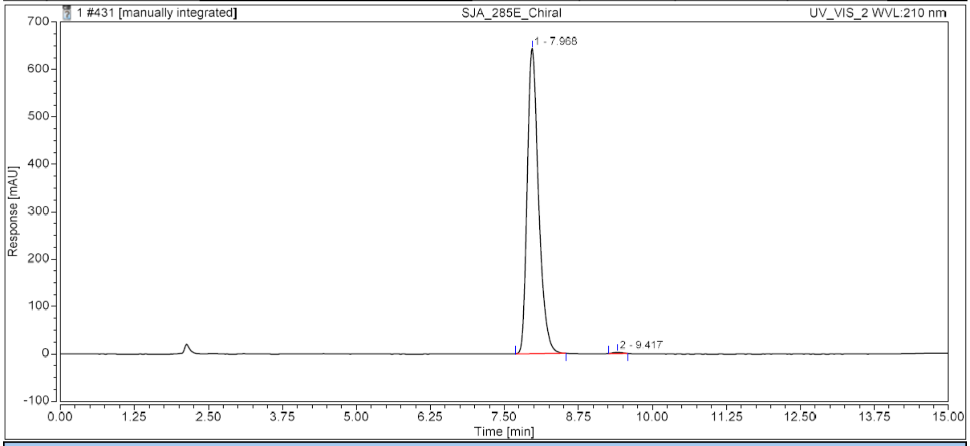

Integration Results							
No.	Peak Name	Retention Time min	Area mAU*min	Height mAU	Relative Area %	Relative Height %	Amount n.a.
1		7.968	142.867	643.786	99.66	99.59	n.a.
2		9.417	0.485	2.649	0.34	0.41	n.a.
**Total:**			**143.352**	**646.435**	**100.00**	**100.00**	

Ethyl (*S*)-2,2-difluoro-5-oxo-4-phenyl-5-(4-(trifluoromethoxy)phenyl)pentanoate (4g)

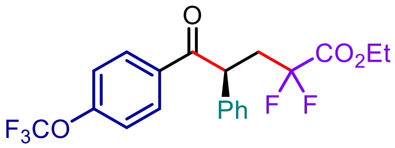

The title compound was synthesized according to the general procedure (**GP 9**) and was obtained after silica gel column chromatography (*n*-pentane : ethylacetate 30:1) as a colorless oil (71 % Yield, 29.5 mg, 97 % ee). $\mathbf{R_f}$ = 0.36 (*n*-pentane : ethylacetate 30:1).

^{1}H NMR (400 MHz, Chloroform-*d*): δ = 8.05 – 7.95 (m, 2H), 7.34 – 7.20 (m, 7H), 4.91 (dd, *J* = 8.1, 4.8 Hz, 1H), 4.19 (dq, *J* = 10.7, 7.2 Hz, 1H), 4.09 (dq, *J* = 10.7, 7.2 Hz, 1H), 3.36 – 3.20 (m, 1H), 2.60 – 2.43 (m, 1H), 1.26 (t, *J* = 7.2 Hz, 3H) ppm.

^{13}C NMR (101 MHz, Chloroform-*d*): δ = 196.2, 164.2 (t, *J* = 32.4 Hz), 153.1, 137.8, 134.4, 131.3, 129.8, 128.6, 128.3, 120.7, 120.6 (q, *J* = 259.1 Hz), 115.6 (t, *J* = 250.6 Hz), 63.4, 47.5 (t, *J* = 4.0 Hz), 38.6 (t, *J* = 23.5 Hz), 14.2 ppm.

^{19}F NMR (376 MHz, Chloroform-*d*): δ = -57.60, -104.07 (dt, *J* = 259.4, 16.7 Hz), -104.88 (d, *J* = 259.4, 16.7 Hz) ppm.

HRMS (APCI/QTOF): *m/z*: $[M + Na]^+$ Calcd. for $C_{20}H_{17}F_5NaO_4^+$: 439.0939; Found 439.0929.

IR (ATR): 1765, 1687, 1602, 1255, 1207, 1167, 1096, 701 cm^{-1}.
$[\alpha]_D^{20}$ = +81.3 (c = 0.5, $CHCl_3$).
Chiral HPLC: (Chiralpak IC, 1 % *i*PrOH/hexane, 1.0 mL/min, 210 nm): tR (minor) 5.40 min, tR (major) 7.11 min, 98.5:1.5 *er.*

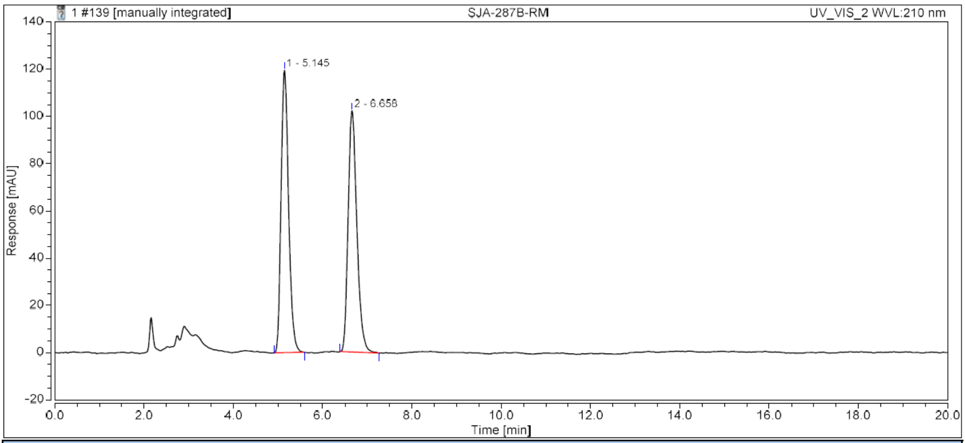

Integration Results							
No.	Peak Name	Retention Time min	Area mAU*min	Height mAU	Relative Area %	Relative Height %	Amount n.a.
1		5.145	23.199	119.594	50.10	53.92	n.a.
2		6.658	23.104	102.188	49.90	46.08	n.a.
Total:			46.303	221.782	100.00	100.00	

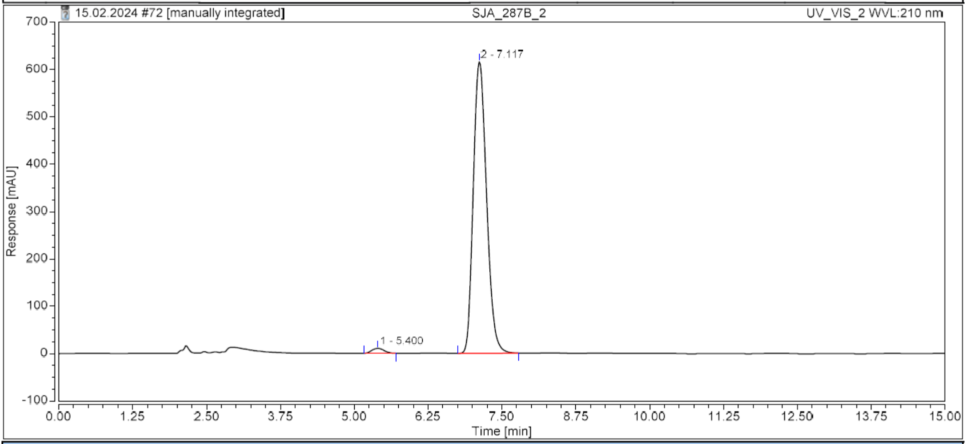

Integration Results							
No.	Peak Name	Retention Time min	Area mAU*min	Height mAU	Relative Area %	Relative Height %	Amount n.a.
1		5.400	2.464	10.431	1.51	1.66	n.a.
2		7.117	160.289	616.100	98.49	98.34	n.a.
Total:			162.753	626.531	100.00	100.00	

Ethyl (*S*)-2,2-difluoro-5-oxo-4-phenyl-5-(4-(trifluoromethyl)phenyl)pentanoate (4h)

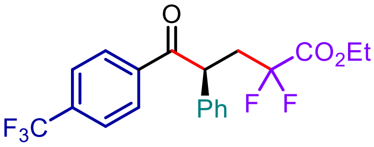

The title compound was synthesized according to the general procedure (**GP 9**) and was obtained after silica gel column chromatography (*n*-pentane : ethylacetate 30:1) as a colorless oil (74 % Yield, 30 mg, 92 % ee). $\mathbf{R_f}$ = 0.40 (*n*-pentane : ethylacetate 30:1).
^{1}H NMR (400 MHz, Chloroform-*d*): δ = 8.05 (d, *J* = 8.2 Hz, 2H), 7.67 (d, *J* = 8.2 Hz, 2H), 7.36 – 7.23 (m, 5H), 4.94 (dd, *J* = 8.2, 4.7 Hz, 1H), 4.21 (dq, *J* = 10.8, 7.1 Hz, 1H), 4.11 (dq, *J* = 10.8, 7.1 Hz, 1H), 3.39 – 3.22 (m, 1H), 2.62 – 2.47 (m, 1H), 1.27 (t, *J* = 7.1 Hz, 3H) ppm.
^{13}C NMR (101 MHz, Chloroform-*d*): δ = 196.2, 163.5 (t, *J* = 32.4 Hz), 138.4, 136.9, 134.2 (q, *J* = 32.7 Hz), 129.2, 128.9, 128.0, 127.8, 125.5 (q, *J* = 3.8 Hz), 123.2 (q, *J* = 272.8 Hz), 114.9 (t, *J* = 250.8 Hz), 62.8, 47.1 (t, *J* = 4.0 Hz), 37.8 (t, *J* = 23.5 Hz), 13.6 ppm.

[19]**F NMR** (376 MHz, Chloroform-*d*): δ = -63.25, -104.06 (dt, *J* = 260.0, 16.7 Hz), -104,86 (dt, *J* = 260.0, 16.7 Hz) ppm.
HRMS (APCI/QTOF): *m/z*: $[M + Na]^+$ Calcd. for $C_{20}H_{17}F_5NaO_3^+$: 423.0990; Found 423.0973.
IR (ATR): 1762, 1691, 1582, 1494, 1409, 1260, 1015, 797, 701, 640 cm^{-1}.
$[\alpha]_D^{20}$ = +50.2 (c = 0.5, $CHCl_3$).
Chiral HPLC: (Chiralpak IC, 1 % *i*PrOH/hexane, 1.0 mL/min, 254 nm): tR (minor) 9.06 min, tR (major) 11.57 min, 96:4 *er.*

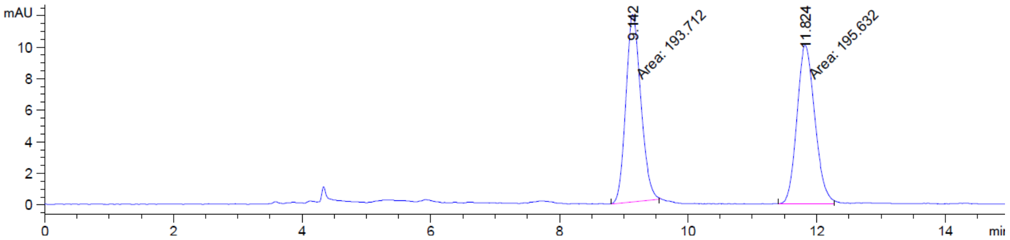

Peak #	RetTime [min]	Type	Width [min]	Area [mAU*s]	Height [mAU]	Area %
1	9.142	MM	0.2713	193.71199	11.89879	49.7534
2	11.824	MM	0.3233	195.63243	10.08666	50.2466

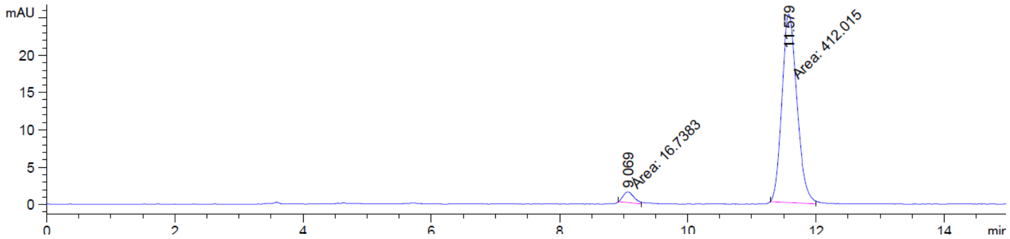

Peak #	RetTime [min]	Type	Width [min]	Area [mAU*s]	Height [mAU]	Area %
1	9.069	MM	0.1956	16.73833	1.42636	3.9040
2	11.579	MM	0.2722	412.01541	25.22833	96.0960

Ethyl (*S*)-2,2-difluoro-5-(4-nitrophenyl)-5-oxo-4-phenylpentanoate (4i)

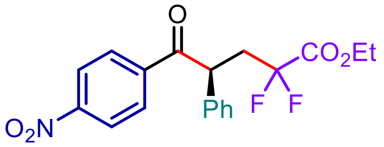

The title compound was synthesized according to the general procedure (**GP 9**) and was obtained after silica gel column chromatography (*n*-pentane : ethylacetate 4:1) as a yellow oil (70 % Yield, 26.4 mg, 92 % ee). $\mathbf{R_f}$ = 0.24 (*n*-pentane : ethylacetate 4:1).
[1]**H NMR** (400 MHz, Chloroform-*d*): δ = 8.27 – 8.20 (m, 2H), 8.11 – 8.05 (m, 2H), 7.35 – 7.29 (m, 2H), 7.28 – 7.23 (m, 3H), 4.93 (dd, *J* = 8.2, 4.6 Hz, 1H), 4.21 (dq, *J* = 10.8, 7.1 Hz, 1H), 4.13 (dq, *J* = 10.8, 7.2 Hz, 1H), 3.40 – 3.32 (m, 1H), 2.62 – 2.42 (m, 1H), 1.28 (t, *J* = 7.1 Hz, 3H) ppm.
[13]**C NMR** (101 MHz, Chloroform-*d*): δ = 196.0, 163.7 (t, *J* = 32.4 Hz), 150.2, 140.5, 136.6, 129.7, 129.6, 128.2, 123.8, 115.0 (t, *J* = 251.0 Hz), 63.1, 47.7 (t, *J* = 4.0 Hz), 37.9 (t, *J* = 23.4 Hz), 13.8 ppm.
[19]**F NMR** (376 MHz, Chloroform-*d*): δ = -104.04 (dt, *J* = 260.2, 16.6 Hz), -104.88 (dt, *J* = 260.2, 16.6 Hz) ppm.
HRMS (ESI/QTOF): *m/z*: $[M + H_{-1}]^-$ Calcd. for $C_{19}H_{16}F_2NO_5^-$: 376.1002; Found 376.0998.
IR (ATR): 1760, 1691, 1602, 1525, 1374, 1047, 828, 726, 541 cm^{-1}.
$[\alpha]_D^{20}$ = +58.2 (c = 1, $CHCl_3$).
Chiral HPLC: (Chiralpak IB, 1 % *i*PrOH/hexane, 1.0 mL/min, 210 nm): tR (minor) 12.12 min, tR (major) 13.21 min, 96:4 *er.*

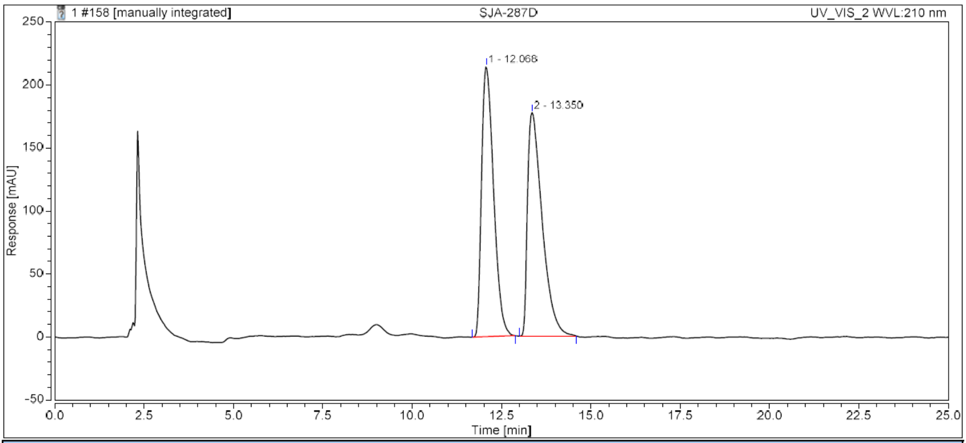

Integration Results							
No.	Peak Name	Retention Time min	Area mAU*min	Height mAU	Relative Area %	Relative Height %	Amount n.a.
1		12.068	83.720	214.413	49.86	54.72	n.a.
2		13.350	84.175	177.451	50.14	45.28	n.a.
**Total:**			**167.894**	**391.864**	**100.00**	**100.00**	

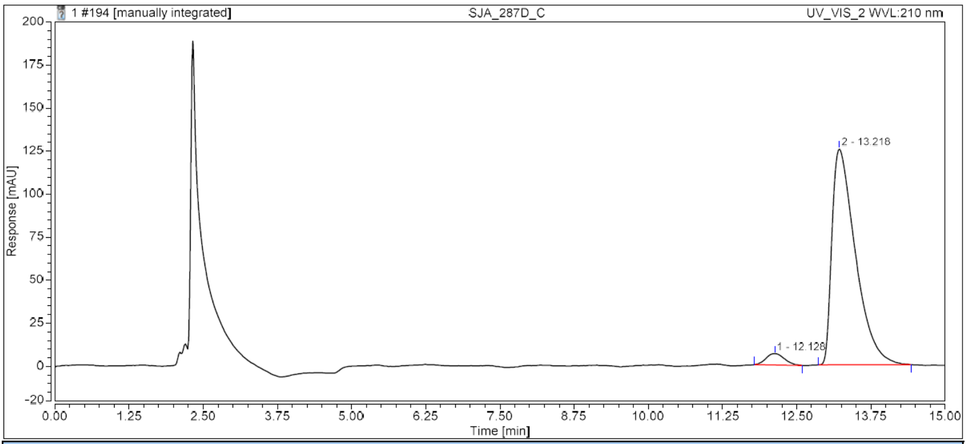

Integration Results							
No.	Peak Name	Retention Time min	Area mAU*min	Height mAU	Relative Area %	Relative Height %	Amount n.a.
1		12.128	2.376	6.608	3.97	5.01	n.a.
2		13.218	57.482	125.356	96.03	94.99	n.a.
**Total:**			**59.858**	**131.964**	**100.00**	**100.00**	

Ethyl (*S*)-2,2-difluoro-5-(3-fluorophenyl)-5-oxo-4-phenylpentanoate (4j)

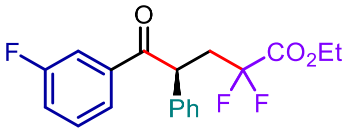

The title compound was synthesized according to the general procedure (**GP 9**) and was obtained after silica gel column chromatography (*n*-pentane : ethylacetate 30:1) as a colorless oil (74 % Yield, 26 mg, 97 % ee). $\mathbf{R_f}$ = 0.24 (*n*-pentane : ethylacetate 30:1).

^{1}H NMR (400 MHz, Chloroform-*d*): δ = 7.75 – 7.71 (m, 1H), 7.65 – 7.60 (m, 1H), 7.41 – 7.17 (m, 7H), 4.89 (dd, *J* = 8.1, 4.8 Hz, 1H), 4.24 – 4.14 (m, 1H), 4.13 – 4.03 (m, 1H), 3.35 – 3.19 (m, 1H), 2.61 – 2.45 (m, 1H), 1.25 (t, *J* = 7.1 Hz, 3H) ppm.

^{13}C NMR (101 MHz, Chloroform-*d*): δ = 195.1 (d, *J* = 2.2 Hz), 162.7 (t, *J* = 32.5 Hz), 161.7 (d, *J* = 248.0 Hz), 136.9 (d, *J* = 6.2 Hz), 136.3, 129.2 (d, *J* = 7.6 Hz), 128.3, 127.1, 126.8, 123.5 (d, *J* = 3.0 Hz), 119.2 (d, *J* = 21.5 Hz), 114.5 (d, *J* = 22.3 Hz), 114.1 (t, *J* = 250.9 Hz), 61.9, 46.1 (t, *J* = 4.1 Hz), 37.1 (t, *J* = 23.5 Hz), 12.7 ppm.

^{19}F NMR (376 MHz, Chloroform-*d*): δ = -104.05 (dt, *J* = 260.2, 16.5 Hz), -104.83 (dt, *J* = 260.2, 16.5 Hz), -111.59 (td, J = 8.7, 5.4 Hz) ppm.

HRMS (ESI/QTOF): *m/z*: $[M + Na]^+$ Calcd. for $C_{19}H_{17}F_3NaO_3^+$: 373.1022; Found 373.1027.

IR (ATR): 1767, 1687, 1562, 1519, 1485, 1304, 1261, 1096, 978, 877, 546 cm^{-1}.

$[\alpha]_D^{20}$ = +41 (c = 0.5, $CHCl_3$).

Chiral HPLC: (Chiralpak IC, 1 % *i*PrOH/hexane, 1.0 mL/min, 210 nm): tR (minor) 7.03 min, tR (major) 8.56 min, 98.5:1.5 *er.*

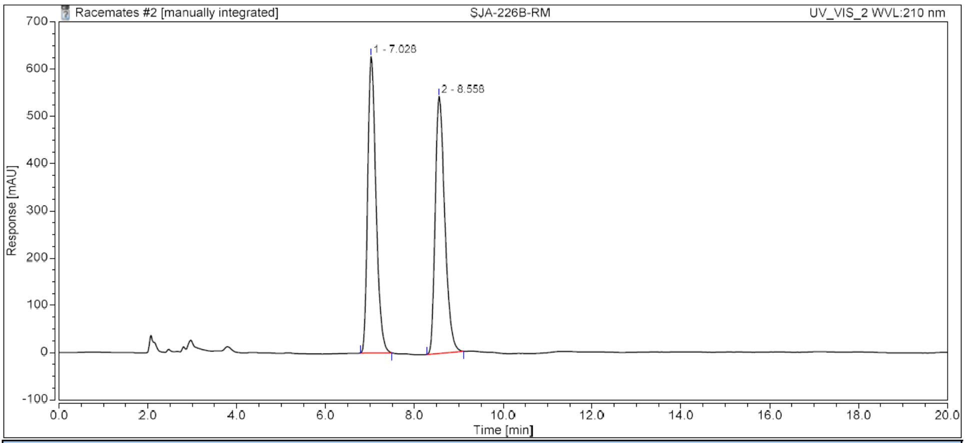

Integration Results

No.	Peak Name	Retention Time min	Area mAU*min	Height mAU	Relative Area %	Relative Height %	Amount n.a.
1		7.028	135.666	627.700	49.99	53.56	n.a.
2		8.558	135.719	544.302	50.01	46.44	n.a.
**Total:**			**271.385**	**1172.002**	**100.00**	**100.00**	

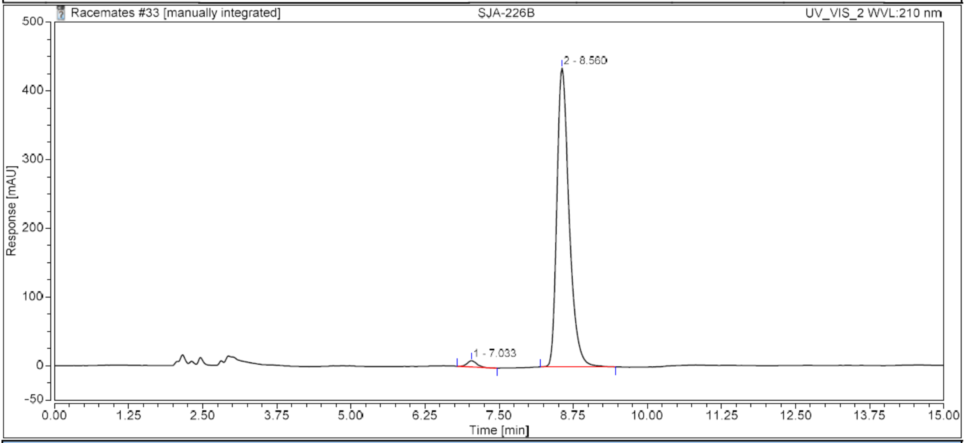

Integration Results

No.	Peak Name	Retention Time min	Area mAU*min	Height mAU	Relative Area %	Relative Height %	Amount n.a.
1		7.033	1.682	9.033	1.59	2.03	n.a.
2		8.560	104.039	435.106	98.41	97.97	n.a.
**Total:**			**105.721**	**444.139**	**100.00**	**100.00**	

Ethyl (*S*)-2,2-difluoro-5-(3-methoxyphenyl)-5-oxo-4-phenylpentanoate (4k)

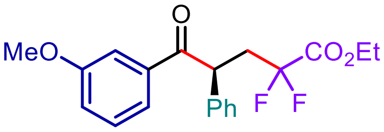

The title compound was synthesized according to the general procedure (**GP 9**) and was obtained after silica gel column chromatography (*n*-pentane : ethylacetate 9:1) as a colorless oil (82 % Yield, 30 mg, 98 % ee). **R$_f$** = 0.25 (*n*-pentane : ethylacetate 9:1).

^{1}H NMR (400 MHz, $CDCl_3$): δ = 7.56 – 7.52 (m, 1H), 7.47 – 7.45 (m, 1H), 7.31 – 7.25 (m, 5H), 7.24 – 7.19 (m, 1H), 7.05 – 7.01 (m, 1H), 4.92 (dd, *J* = 8.0, 5.0 Hz, 1H), 4.16 (dq, *J* = 10.7, 7.2 Hz, 1H), 4.05 (dq, *J* = 10.8, 7.2 Hz, 1H), 3.79 (s, 3H), 3.33 – 3.17 (m, 1H), 2.60 – 2.46 (m, 1H), 1.23 (t, *J* = 7.2 Hz, 3H) ppm.

^{13}C NMR (101 MHz, $CDCl_3$): δ = 197.5, 164.2 (t, *J* = 32.4 Hz), 160.2, 138.2, 137.6, 130.0, 129.6, 128.7, 128.1, 121.8, 120.1, 115.7 (t, *J* = 250.6 Hz), 113.6, 63.3, 55.8, 47.4 (t, *J* = 3.9 Hz), 38.6 (t, *J* = 23.3 Hz), 14.2 ppm.

^{19}F NMR (376 MHz, $CDCl_3$): δ = -103.89 (dt, *J* = 258.2, 16.7 Hz), -104.85 (dt, *J* = 258.2, 16.7 Hz) ppm.

HRMS (ESI/QTOF): *m/z*: [M + Na]+ Calcd. for $C_{20}H_{20}F_2NaO_4^+$: 385.1222; Found 385.1217.

IR (ATR): 1766, 1684, 1597, 1582, 1265, 1097, 1073, 730, 700 cm^{-1}.

$[\alpha]_D^{20}$ = +104.0 (c = 0.5, $CHCl_3$).

Chiral HPLC: (Chiralpak IC, 3 % *i*PrOH/hexane, 1.0 mL/min, 210 nm): tR (minor) 7.03 min, tR (major) 10.65 min, 99:1 *er.*

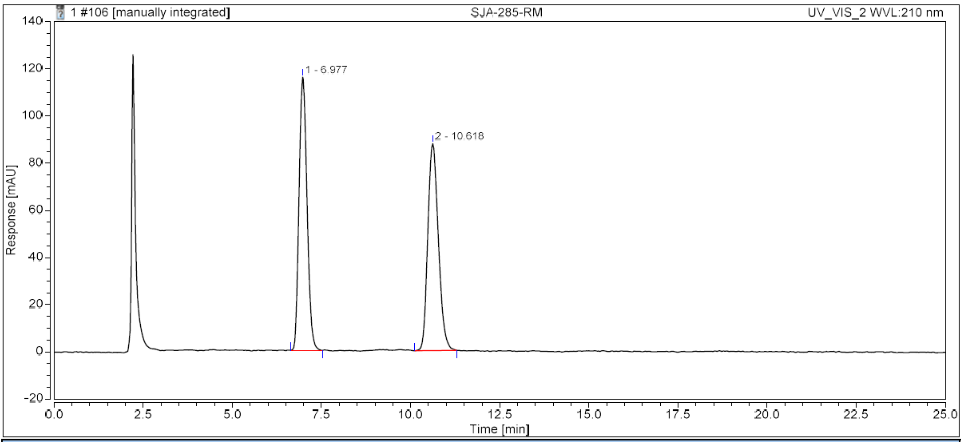

Integration Results							
No.	Peak Name	Retention Time min	Area mAU*min	Height mAU	Relative Area %	Relative Height %	Amount n.a.
1		6.977	30.116	115.790	50.05	56.89	n.a.
2		10.618	30.059	87.749	49.95	43.11	n.a.
Total:			60.175	203.540	100.00	100.00	

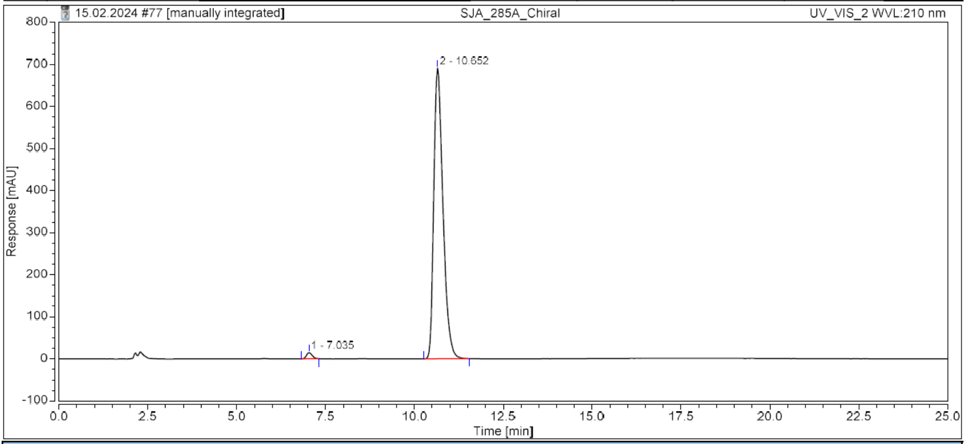

Integration Results							
No.	Peak Name	Retention Time min	Area mAU*min	Height mAU	Relative Area %	Relative Height %	Amount n.a.
1		7.035	2.787	14.723	1.33	2.08	n.a.
2		10.652	206.819	691.489	98.67	97.92	n.a.
Total:			209.605	706.212	100.00	100.00	

Ethyl (*S*)-5-(3,5-di-*tert*-butylphenyl)-2,2-difluoro-5-oxo-4-phenylpentanoate (4l)

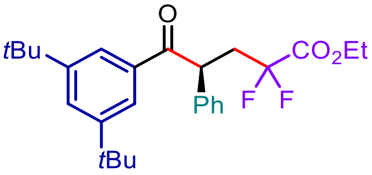

The title compound was synthesized according to the general procedure (**GP 9**) and was obtained after silica gel column chromatography (*n*-pentane : ethylacetate 40:1) as a colorless oil (41 % Yield, 18 mg, 90 % ee). $\mathbf{R_f}$ = 0.52 (*n*-pentane : ethylacetate 40:1).

^{1}H NMR (400 MHz, Chloroform-*d*): δ = 7.82 (d, *J* = 1.9 Hz, 2H), 7.56 (t, *J* = 1.8 Hz, 1H), 7.34 – 7.27 (m, 4H), 7.24 – 7.19 (m, 1H), 4.93 (dd, *J* = 7.3, 5.7 Hz, 1H), 4.19 – 4.10 (m 1H), 4.07 – 3.98 (m, 1H), 3.30 – 3.15 (m, 1H), 2.66 – 2.50 (m, 1H), 1.29 (s, 18H), 1.20 (t, *J* = 7.1 Hz, 3H) ppm.

^{13}C NMR (101 MHz, Chloroform-*d*): δ = 197.7, 163.9 (t, *J* = 32.8 Hz), 151.2, 138.4, 135.2, 129.2, 128.5, 127.7, 127.6, 123.4, 118.5 (t, *J* = 249 Hz), 62.9, 47.4 (t, *J* = 4.0 Hz), 38.3 (t, *J* = 23.3 Hz), 35.0, 31.4, 13.8 ppm.

^{19}F NMR (376 MHz, $CDCl_3$): δ = -103.56 (dt, J = 259.9, 16.8 Hz)), -104.79 (dt, J = 259.9, 16.8 Hz) ppm.

HRMS (ESI/QTOF): *m/z*: [M + H]$^+$ Calcd. for $C_{27}H_{35}F_2O_3^+$: 445.2549; Found 445.2553.

IR (ATR): 2962, 1767, 1680, 1597, 1394, 1307, 1178, 1030, 898, 822, 778, 638, 573 cm^{-1}.

$[\alpha]_D^{20}$ = +37.2 (c = 0.5, $CHCl_3$).

Chiral HPLC: (Chiralpak IC, 0.3 % *i*PrOH/hexane, 1.0 mL/min, 254 nm): tR (minor) 8.59 min, tR (major) 10.42 min, 95:5 *er.*

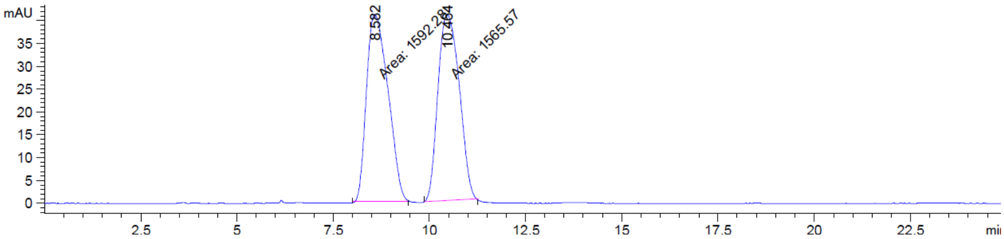

Peak #	RetTime [min]	Type	Width [min]	Area [mAU*s]	Height [mAU]	Area %
1	8.582	MM	0.6476	1592.27991	40.97897	50.4229
2	10.464	MM	0.6411	1565.57227	40.70322	49.5771

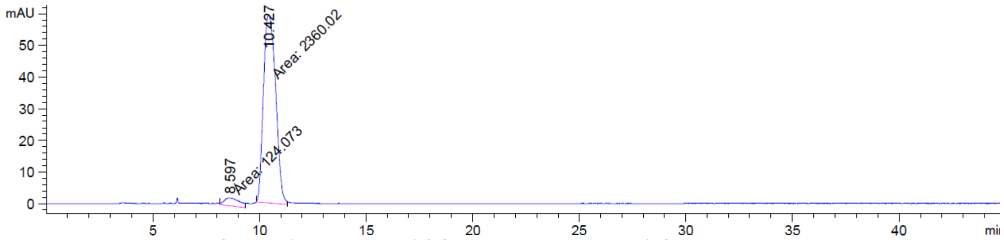

Peak #	RetTime [min]	Type	Width [min]	Area [mAU*s]	Height [mAU]	Area %
1	8.597	MM	0.8445	124.07339	2.44853	4.9947
2	10.427	MM	0.6615	2360.01831	59.46260	95.0053

Ethyl (*S*)-2,2-difluoro-5-(2-fluorophenyl)-5-oxo-4-phenylpentanoate (4m)

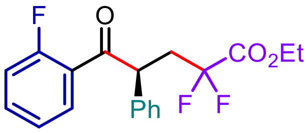

The title compound was synthesized according to the general procedure (**GP 9**) and was obtained after silica gel column chromatography (*n*-pentane : ethylacetate 30:1) as a colorless oil (78 % Yield, 27 mg, 94% ee). $\mathbf{R_f}$ = 0.34 (*n*-pentane : ethylacetate 30:1).

^{1}H NMR (400 MHz, Chloroform-*d*): δ = 7.68 (td, *J* = 7.6, 1.9 Hz, 1H), 7.40 – 7.32 (m, 1H), 7.22 – 7.11 (m, 5H), 7.10 – 7.04 (m, 1H), 7.00 – 6.93 (m, 1H), 4.83 (dd, *J* = 7.8, 5.3 Hz, 1H), 4.10 (dq, *J* = 10.8, 7.2 Hz, 1H), 4.02 (dq, *J* = 10.7, 7.1 Hz, 1H), 3.32 – 3.10 (m, 1H), 2.52 – 2.36 (m, 1H), 1.19 (t, *J* = 7.1 Hz, 3H) ppm.

^{13}C NMR (101 MHz, Chloroform-*d*): δ = 196.1 (d, *J* = 4.3 Hz), 163.8 (t, *J* = 32.7 Hz), 161.0 (d, *J* = 254.8 Hz), 136.8, 134.6 (d, *J* = 9.1 Hz), 131.2 (d, *J* = 2.8 Hz), 128.9, 128.6, 127.7, 125.0 (d, *J* = 12.2 Hz), 124.4 (d, *J* = 3.7 Hz), 116.71 (d, J = 24.1 Hz), 115.2 (t, *J* = 250.7 Hz), 62.9, 50.6 (dt, *J* = 7.6, 3.8 Hz), 37.8 (t, *J* = 23.3 Hz), 13.7 ppm.

^{19}F NMR (376 MHz, Chloroform-*d*): δ = -103.70 (dt, *J* = 260.7, 16.0 Hz), -104.79 (dt, *J* = 260.7, 16.0 Hz), -109.32 (dt, *J* = 11.8, 6.4 Hz) ppm.

HRMS (ESI/QTOF): *m/z*: $[M + Na]^+$ Calcd. for $C_{19}H_{17}F_3NaO_3^+$: 373.1022; Found 373.1010.

IR (ATR): 1765, 1685, 1609, 1578, 1430, 1204, 1053, 846, 700, 410 cm^{-1}.

$[\alpha]_D^{20}$ = +43.2 (c = 1, $CHCl_3$).

Chiral HPLC: (Chiralpak IC, 1 % *i*PrOH/hexane, 1.0 mL/min, 210 nm): tR (minor) 9.12 min, tR (major) 11.85 min, 97:3 *er.*

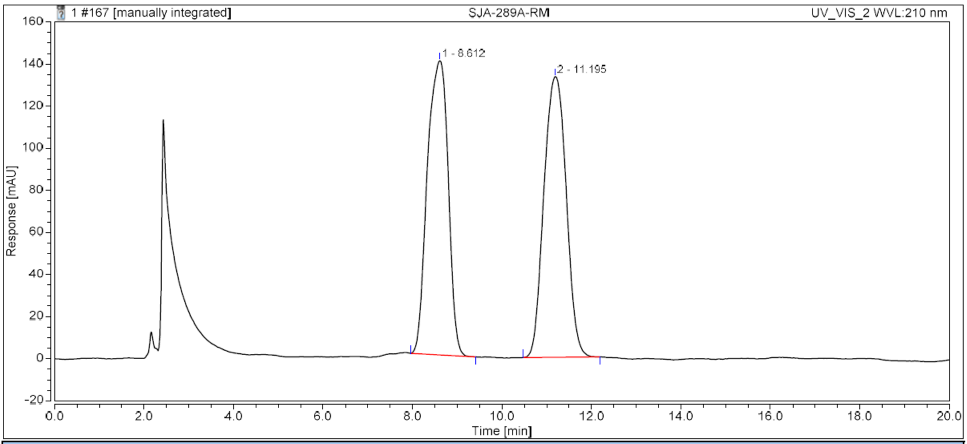

Integration Results							
No.	Peak Name	Retention Time min	Area mAU*min	Height mAU	Relative Area %	Relative Height %	Amount n.a.
1		8.612	75.409	139.854	49.43	51.16	n.a.
2		11.195	77.134	133.520	50.57	48.84	n.a.
Total:			152.543	273.375	100.00	100.00	

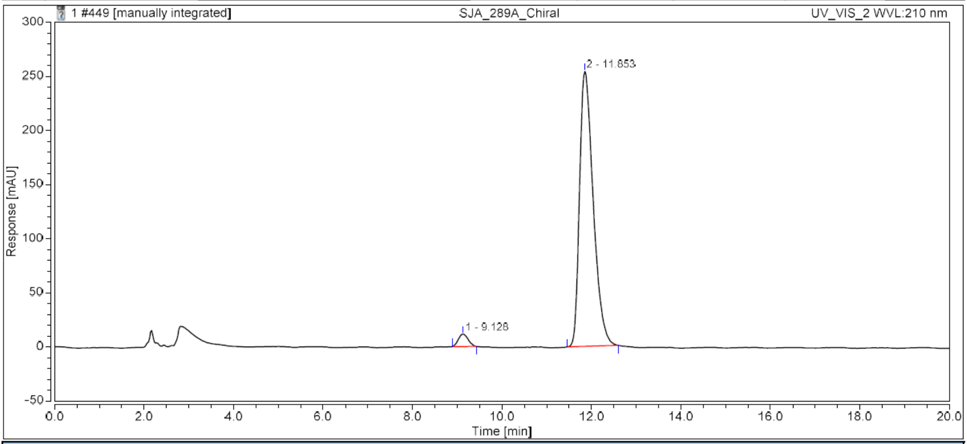

Integration Results							
No.	Peak Name	Retention Time min	Area mAU*min	Height mAU	Relative Area %	Relative Height %	Amount n.a.
1		9.128	2.894	11.505	3.13	4.33	n.a.
2		11.853	89.720	254.169	96.87	95.67	n.a.
Total:			92.614	265.673	100.00	100.00	

Ethyl (*S*)-2,2-difluoro-5-(2-hydroxyphenyl)-5-oxo-4-phenylpentanoate (4n)

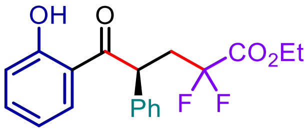

The title compound was synthesized according to the general procedure (**GP 9**) and was obtained after silica gel column chromatography (*n*-pentane : ethylacetate 20:1) as a colorless oil (48 % Yield, 17 mg, 94 % ee). $\mathbf{R_f}$ = 0.22 (*n*-pentane : ethylacetate 20:1).

^{1}H NMR (400 MHz, $CDCl_3$): δ = 12.13 (s, 1H), 7.84 (dd, *J* = 8.1, 1.6 Hz, 1H), 7.46 – 7.40 (m, 1H), 7.36 – 7.31 (m, 4H), 7.30 – 7.24 (m, 1H), 6.97 (dd, *J* = 8.5, 1.1 Hz, 1H), 6.86 (ddd, *J* = 8.2, 7.2, 1.2 Hz, 1H), 5.02 (dd, *J* = 8.3, 4.8 Hz, 1H), 4.21 (dq, *J* = 10.8, 7.2 Hz, 1H), 4.09 (dq, *J* = 10.8, 7.2 Hz, 1H), 3.36 – 3.20 (m, 1H), 2.62 – 2.47 (m, 1H), 1.25 (t, *J* = 7.2 Hz, 3H) ppm.

^{13}C NMR (101 MHz, $CDCl_3$): δ = 203.0, 163.3, 137.6, 136.7, 130.2, 129.3, 128.0, 127.9, 119.1, 118.7, 118.2, 115.0, 63.0, 46.2 (t, *J* = 3.8 Hz), 37.8 (t, *J* = 23.3 Hz), 13.7 ppm.

^{19}F NMR (376 MHz, $CDCl_3$): δ = -104.39 (td, *J* = 16.6, 10.0 Hz) ppm.

HRMS (ESI/QTOF): *m/z*: $[M + Na]^+$ Calcd. for $C_{19}H_{18}F_2NaO_4^+$: 371.1065; Found 371.1059.

IR (ATR): 1766, 1637, 1614, 1374, 1245, 1045, 829, 617, 446 cm^{-1}.

$[\alpha]_D^{20}$ = +89.0 (c = 0.5, $CHCl_3$).

Chiral HPLC: (Chiralpak IC, 1 % *i*PrOH/hexane, 1.0 mL/min, 210 nm): tR (minor) 6.67 min, tR (major) 8.07 min, 97:3 *er*.

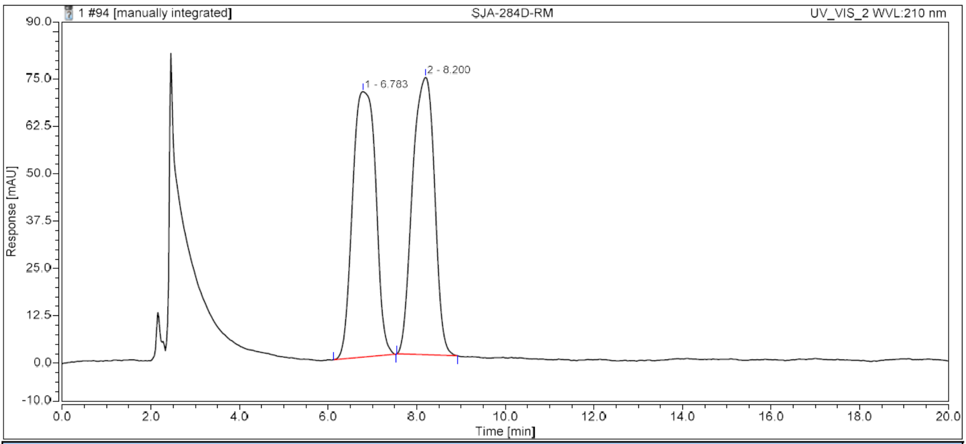

Integration Results							
No.	Peak Name	Retention Time min	Area mAU*min	Height mAU	Relative Area %	Relative Height %	Amount n.a.
1		6.783	42.716	70.105	50.46	48.90	n.a.
2		8.200	41.930	73.253	49.54	51.10	n.a.
Total:			84.646	143.358	100.00	100.00	

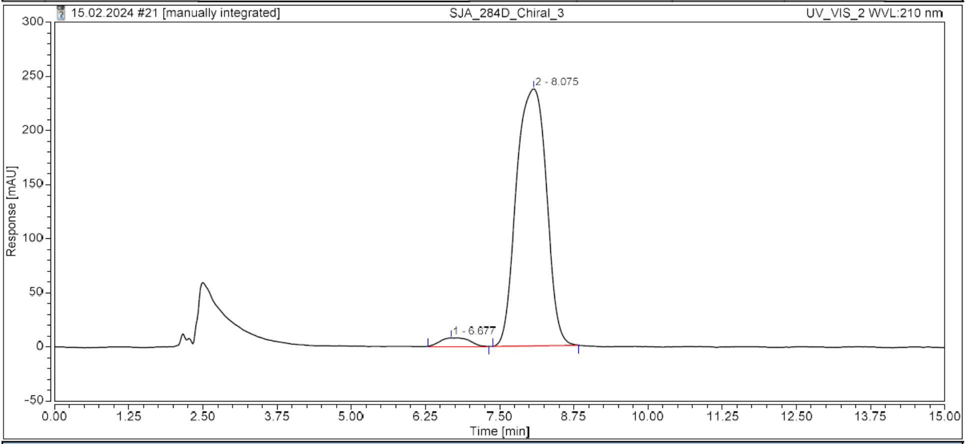

Integration Results							
No.	Peak Name	Retention Time min	Area mAU*min	Height mAU	Relative Area %	Relative Height %	Amount n.a.
1		6.677	4.357	7.682	3.04	3.14	n.a.
2		8.075	139.159	237.289	96.96	96.86	n.a.
Total:			143.516	244.971	100.00	100.00	

Ethyl (*S*)-2,2-difluoro-5-(naphthalen-2-yl)-5-oxo-4-phenylpentanoate (4o)

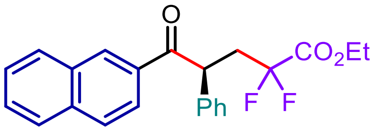

The title compound was synthesized according to the general procedure (**GP 9**) and was obtained after silica gel column chromatography (*n*-pentane : ethylacetate 20:1) as a colorless oil (81 % Yield, 31 mg, 94 % ee). **R$_f$** = 0.20 (*n*-pentane : ethylacetate 20:1).

^{1}H NMR (400 MHz, Chloroform-*d*): δ = 8.48 (s, 1H), 7.99 (dd, *J* = 8.6, 1.8 Hz, 1H), 7.94 – 7.88 (m, 1H), 7.83 – 7.78 (m, 2H), 7.58 – 7.48 (m, 2H), 7.37 – 7.32 (m, 2H), 7.30 – 7.25 (m, 2H), 7.21 – 7.16 (m, 1H), 5.11 (dd, *J* = 8.0, 5.0 Hz, 1H), 4.16 (dq, *J* = 10.8, 7.2 Hz, 1H), 4.05 (dq, *J* = 10.8, 7.1 Hz, 1H), 3.40 – 3.25 (m, 1H), 2.67 – 2.51 (m, 1H), 1.21 (t, *J* = 7.2 Hz, 3H) ppm.

^{13}C NMR (101 MHz, Chloroform-*d*): δ = 197.7, 164.2 (t, *J* = 32.4 Hz), 138.3, 136.0, 133.5, 132.8, 131.1, 130.1, 129.7, 129.1, 128.9, 128.7, 128.1, 127.2, 124.8, 115.8 (t, *J* = 250.6 Hz), 63.3, 47.4 (t, *J* = 4.1 Hz), 38.6 (t, *J* = 23.3 Hz), 14.2 ppm.

^{19}F NMR (376 MHz, $CDCl_3$): δ = -103.89 (dt, *J* = 256.8, 16.5 Hz), -104.66 (dt, *J* = 256.8, 16.5 Hz) ppm.

HRMS (ESI/QTOF): *m/z*: [M + Na]$^+$ Calcd. for $C_{23}H_{20}F_2NaO_3^+$: 405.1273; Found 405.1263.

IR (ATR): 1761, 1676, 1626, 1468, 1352, 1209, 1173, 1071, 927, 699, 595 cm^{-1}.
$[\alpha]_D^{20}$ = -42.2 (c = 0.3, $CHCl_3$).
Chiral HPLC: (Chiralpak IC, 3 % *i*PrOH/hexane, 1.0 mL/min, 210 nm): tR (minor) 7.74 min, tR (major) 11.99 min, 97:3 *er.*

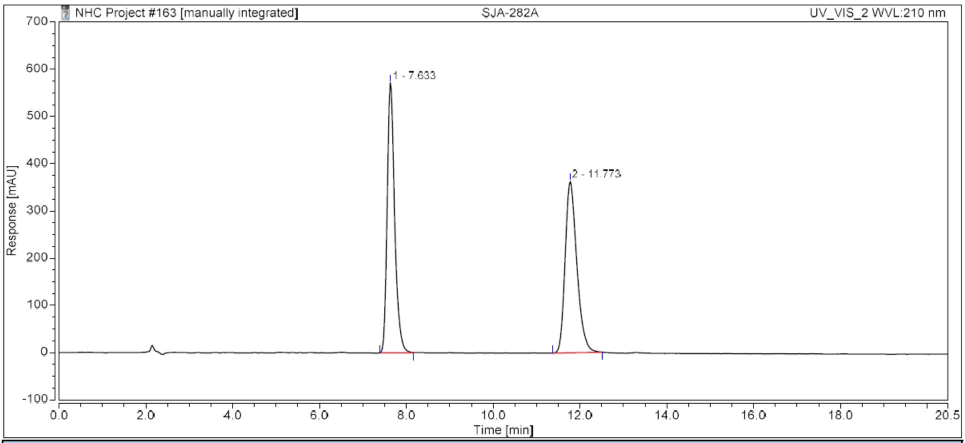

Integration Results							
No.	Peak Name	Retention Time min	Area mAU*min	Height mAU	Relative Area %	Relative Height %	Amount n.a.
1		7.633	113.793	571.775	50.08	61.16	n.a.
2		11.773	113.420	363.042	49.92	38.84	n.a.
Total:			227.212	934.817	100.00	100.00	

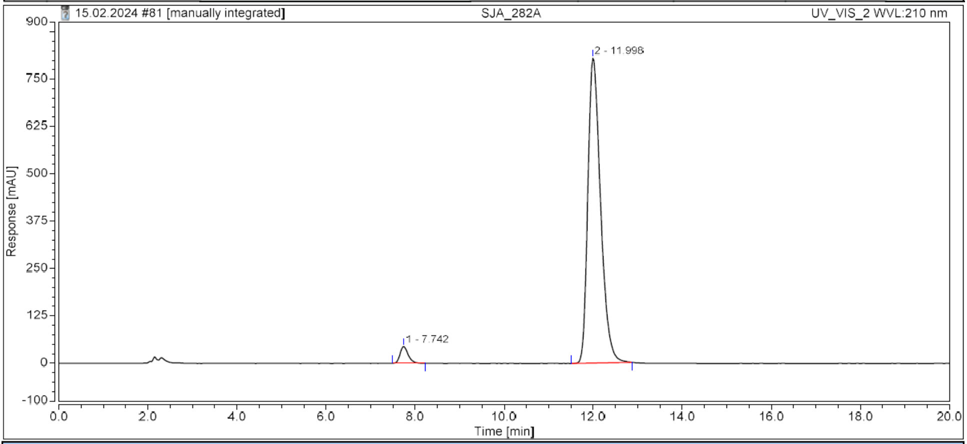

Integration Results							
No.	Peak Name	Retention Time min	Area mAU*min	Height mAU	Relative Area %	Relative Height %	Amount n.a.
1		7.742	9.343	44.381	3.39	5.22	n.a.
2		11.998	265.943	805.172	96.61	94.78	n.a.
Total:			275.286	849.553	100.00	100.00	

Ethyl (*S*)-2,2-difluoro-5-(naphthalen-1-yl)-5-oxo-4-phenylpentanoate (4p)

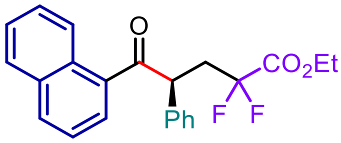

The title compound was synthesized according to the general procedure (**GP 9**) and was obtained after silica gel column chromatography (*n*-pentane : ethylacetate 20:1) as a colorless oil (78 % Yield, 30 mg, 96 % ee). **R$_f$** = 0.20 (*n*-pentane : ethylacetate 20:1).
^{1}H NMR (400 MHz, Chloroform-*d*): δ = 8.32 – 8.25 (m, 1H), 7.97 – 7.87 (m, 2H), 7.84 – 7.79 (m, 1H), 7.54 – 7.43 (m, 3H), 7.31 – 7.22 (m, 4H), 7.21 – 7.14 (m, 1H), 4.98 (dd, *J* = 8.2, 4.7 Hz, 1H), 4.21 (dq, J = 11.2, 7.4 Hz, 1H), 4.12 (dq, *J* = 10.8, 7.4 Hz, 1H), 3.53 – 3.36 (m, 1H), 2.68 – 2.54 (m, 1H), 1.27 (t, *J* = 7.1 Hz, 3H) ppm.
^{13}C NMR (101 MHz, Chloroform-*d*): δ = 201.0, 164.3 (t, *J* = 32.4 Hz), 137.6, 136.0, 134.2, 133.1, 131.0, 129.5, 128.77, 128.71, 128.3, 128.1, 127.8, 126.9, 125.9, 124.7, 115.8 (d, J = 250.7 Hz), 63.4, 50.7 (t, *J* = 3.8 Hz), 38.1 (t, *J* = 23.3 Hz), 14.2 ppm.

^{19}F NMR (376 MHz, $CDCl_3$): δ = -103.97 (dt, *J* = 258.1, 16.4 Hz), -104.87 (dt, *J* = 258.1, 16.4 Hz) ppm.
HRMS (ESI/QTOF): *m/z*: $[M + H]^+$ Calcd. for $C_{23}H_{21}F_2O_3^+$: 383.1453; Found 383.1440.
IR (ATR): 1762, 1683, 1592, 1507, 1410, 1033, 926, 775, 745, 506 cm^{-1}.
$[\alpha]_D^{20}$ = +55.5 (c = 0.3, $CHCl_3$).
Chiral HPLC: (Chiralpak IC, 1 % *i*PrOH/hexane, 1.0 mL/min, 210 nm): tR (minor) 11.87 min, tR (major) 15.78 min, 98:2 *er*.

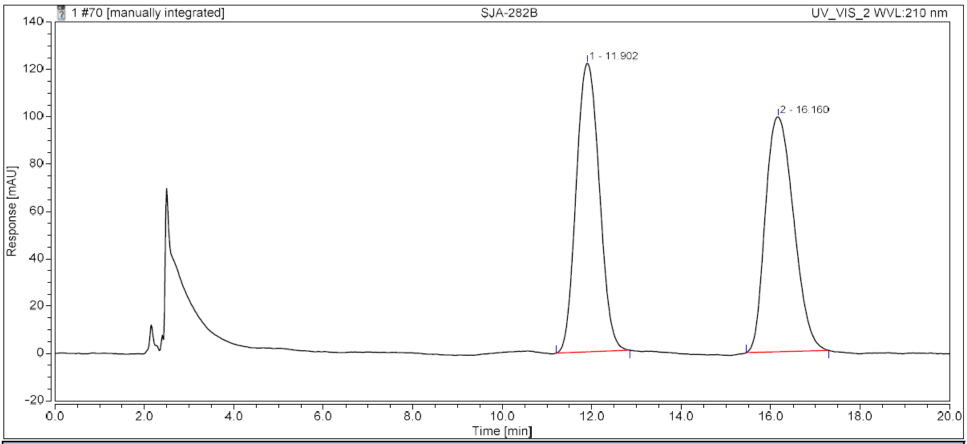

Integration Results

No.	Peak Name	Retention Time min	Area mAU*min	Height mAU	Relative Area %	Relative Height %	Amount n.a.
1		11.902	73.079	121.922	50.09	55.13	n.a.
2		16.160	72.815	99.244	49.91	44.87	n.a.
Total:			145.894	221.166	100.00	100.00	

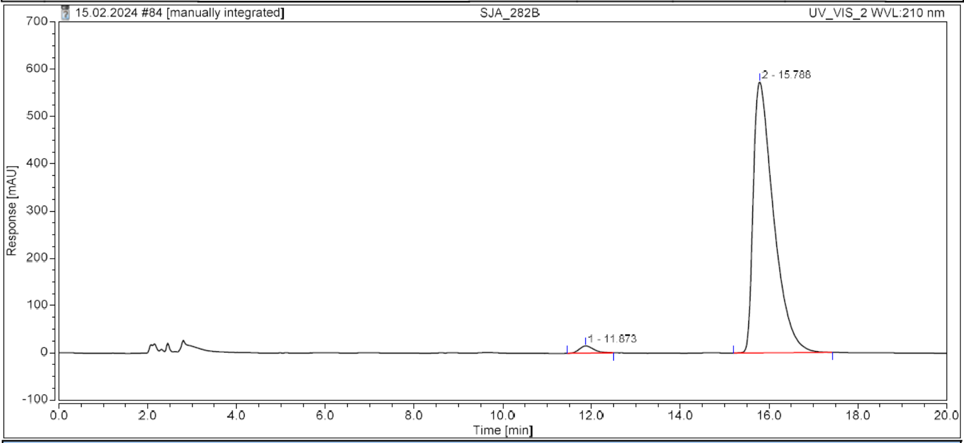

Integration Results

No.	Peak Name	Retention Time min	Area mAU*min	Height mAU	Relative Area %	Relative Height %	Amount n.a.
1		11.873	5.670	15.160	1.83	2.58	n.a.
2		15.788	303.366	573.368	98.17	97.42	n.a.
Total:			309.036	588.529	100.00	100.00	

Ethyl (S)-2,2-difluoro-5-oxo-4-phenyl-5-(thiophen-2-yl)pentanoate (4q)

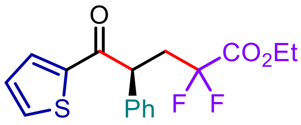

The title compound was synthesized according to the general procedure (**GP 9**) and was obtained after silica gel column chromatography (*n*-pentane : ethylacetate 20:1 to 9:1) as a pale yellow oil (80 % Yield, 27 mg, 95 % ee). R_f = 0.2 (*n*-pentane : ethylacetate 20:1).
^{1}H NMR (400 MHz, $CDCl_3$): δ = 7.73 (dd, *J* = 3.8, 1.1 Hz, 1H), 7.59 (dd, *J* = 5.0, 1.1 Hz, 1H), 7.36 – 7.28 (m, 4H), 7.27 – 7.22 (m, 1H), 7.06 (dd, *J* = 5.0, 3.9 Hz, 1H), 4.75 (dd, *J* = 7.8, 5.2 Hz, 1H), 4.17 (dq, *J* = 10.7, 7.2 Hz, 1H), 4.07 (dq, *J* = 10.7, 7.1 Hz, 1H), 3.31 – 3.15 (m, 1H), 2.61 – 2.47 (m, 1H), 1.24 (t, *J* = 7.1 Hz, 3H) ppm.
^{13}C NMR (101 MHz, $CDCl_3$): δ = 190.1, 163.7 (t, *J* = 32.5 Hz), 142.8, 137.9, 134.3, 132.9, 129.2, 128.2, 127.8, 115.2(t, *J* = 250.9 Hz), 62.9, 48.3 (t, *J* = 3.9 Hz), 37.9 (t, *J* = 23.6 Hz), 13.7 ppm.

[19]**F NMR** (376 MHz, $CDCl_3$): δ = -104.01 (dt, *J* = 259.5, 16.6 Hz), -104.92 (dt, *J* = 260.7, 16.7 Hz) ppm.
HRMS (ESI/QTOF): *m/z*: [M + Na]$^+$ Calcd. for $C_{17}H_{16}F_2NaO_3S^+$: 361.0680; Found 361.0672.
IR (ATR): 1763, 1660, 1600, 1518, 1413, 1264, 1029, 779, 563, 412 cm^{-1}.
$[\alpha]_D^{20}$ = +83.6 (c = 0.5, $CHCl_3$).
Chiral HPLC: (Chiralpak IC, 3 % *i*PrOH/hexane, 1.0 mL/min, 210 nm): tR (minor) 9.68 min, tR (major) 16.25 min, 97.5:2.5 *er.*

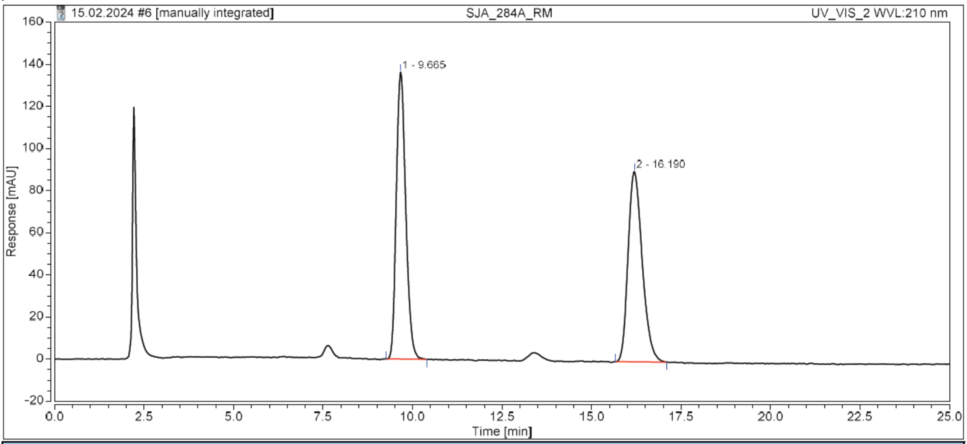

Integration Results

No.	Peak Name	Retention Time min	Area mAU*min	Height mAU	Relative Area %	Relative Height %	Amount n.a.
1		9.665	41.579	136.232	50.09	60.08	n.a.
2		16.190	41.435	90.517	49.91	39.92	n.a.
Total:			83.014	226.749	100.00	100.00	

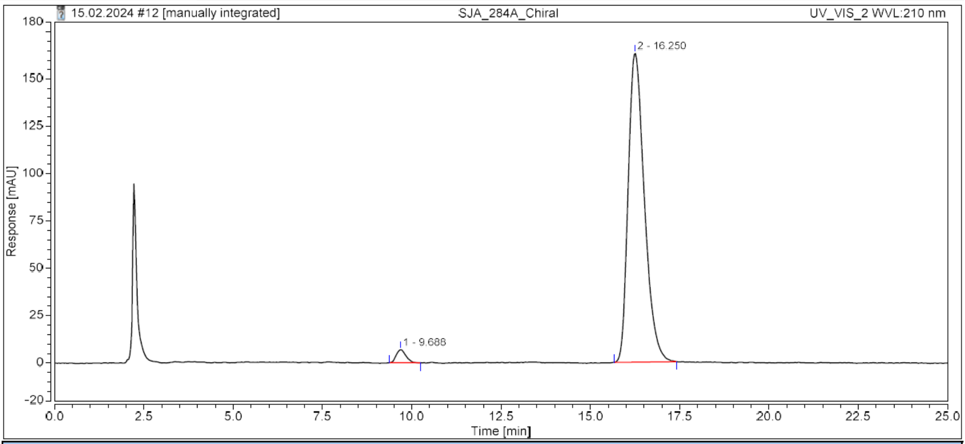

Integration Results

No.	Peak Name	Retention Time min	Area mAU*min	Height mAU	Relative Area %	Relative Height %	Amount n.a.
1		9.688	2.128	6.855	2.42	4.03	n.a.
2		16.250	85.920	163.234	97.58	95.97	n.a.
Total:			88.049	170.089	100.00	100.00	

Ethyl (S)-2,2-difluoro-5-(furan-2-yl)-5-oxo-4-phenylpentanoate (4r)

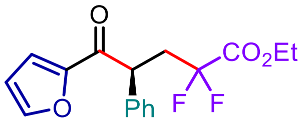

The title compound was synthesized according to the general procedure (**GP 9**) and was obtained after silica gel column chromatography (*n*-pentane : ethylacetate 20:1 to 9:1) as a coloreless oil (77 % Yield, 25 mg, 94 % ee). **R**$_f$ = 0.22 (*n*-pentane : ethylacetate 20:1).
[1]**H NMR** (400 MHz, $CDCl_3$): δ = 7.55 (dd, *J* = 1.7, 0.8 Hz, 1H), 7.36 – 7.28 (m, 4H), 7.27 – 7.20 (m, 2H), 6.49 (dd, *J* = 3.6, 1.7 Hz, 1H), 4.76 (dd, *J* = 8.0, 5.2 Hz, 1H), 4.17 (dq, *J* = 10.7, 7.2 Hz, 1H), 4.08 (dq, *J* = 10.7, 7.2 Hz, 1H), 3.30 – 3.14 (m, 1H), 2.61 – 2.46 (m, 1H), 1.25 (t, *J* = 7.1 Hz, 3H) ppm.
[13]**C NMR** (101 MHz, $CDCl_3$): δ = 186.6, 164.1 (t, *J* = 32.6 Hz), 152.1, 147.2, 137.9, 129.4, 128.8, 128.2, 118.9, 116.9 (d, *J* = 250.3 Hz), 112.9, 63.3, 47.4 (t, *J* = 3.9 Hz), 37.6 (t, *J* = 23.6 Hz), 14.2 ppm.

^{19}F NMR (376 MHz, $CDCl_3$): δ = -104.05 (dt, *J* = 259.4, 16.7 Hz), -105.13 (dt, *J* = 259.4, 16.7 Hz) ppm.
HRMS (ESI/QTOF): *m/z*: $[M + Na]^+$ Calcd. for $C_{17}H_{16}F_2NaO_4^+$: 345.0909; Found 345.0898.
IR (ATR): 1764, 1674, 1600, 1430 ,1273, 1029, 852, 642, 452 cm^{-1}.
$[\alpha]_D^{20}$ = +81.6 (c = 0.5, $CHCl_3$).
Chiral HPLC: (Chiralpak IC, 3 % *i*PrOH/hexane, 1.0 mL/min, 210 nm): tR (minor) 13.11 min, tR (major) 20.65 min, 97:3 *er*.

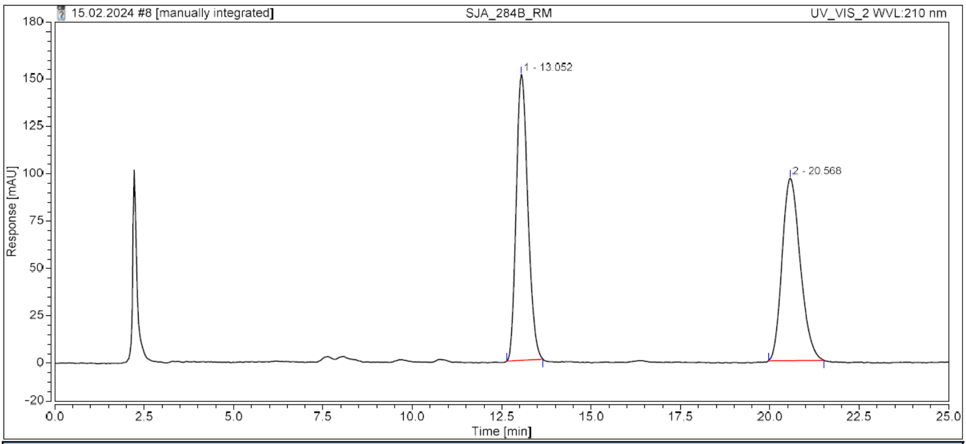

Integration Results

No.	Peak Name	Retention Time min	Area mAU*min	Height mAU	Relative Area %	Relative Height %	Amount n.a.
1		13.052	57.000	151.040	50.16	61.08	n.a.
2		20.568	56.632	96.234	49.84	38.92	n.a.
**Total:**			**113.632**	**247.274**	**100.00**	**100.00**	

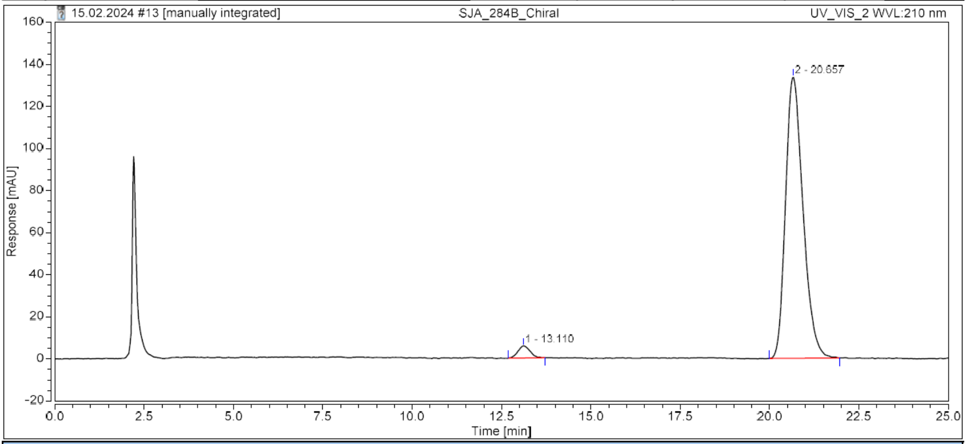

Integration Results

No.	Peak Name	Retention Time min	Area mAU*min	Height mAU	Relative Area %	Relative Height %	Amount n.a.
1		13.110	2.243	5.726	2.87	4.11	n.a.
2		20.657	75.913	133.755	97.13	95.89	n.a.
**Total:**			**78.156**	**139.481**	**100.00**	**100.00**	

Ethyl (*S*)-2,2-difluoro-5-oxo-4-phenyl-5-(pyridin-3-yl)pentanoate (4s)

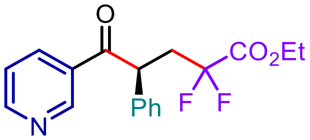

The title compound was synthesized according to the general procedure (**GP 9**) and was obtained after silica gel column chromatography (*n*-pentane : ethylacetate 9:1) as a pale yellow oil (58 % Yield, 19 mg, 86 % ee). $\mathbf{R_f}$ = 0.22 (*n*-pentane : ethylacetate 9:1).
^{1}H NMR (400 MHz, $CDCl_3$): δ = 9.15 (dd, *J* = 2.3, 0.9 Hz, 1H), 8.69 (dd, *J* = 4.8, 1.7 Hz, 1H), 8.19 (ddd, *J* = 8.0, 2.3, 1.7 Hz, 1H), 7.37 – 7.20 (m, 6H), 4.89 (dd, *J* = 8.1, 4.8 Hz, 1H), 4.19 (dq, *J* = 10.8, 7.2 Hz, 1H), 4.10 (dq, *J* = 10.8, 7.2 Hz, 1H), 3.36 – 3.20 (m, 1H), 2.59 – 2.45 (m, 1H), 1.25 (t, *J* = 7.2 Hz, 3H) ppm.
^{13}C NMR (101 MHz, $CDCl_3$): δ = 196.4, 163.8 (t, *J* = 32.0 Hz), 153.6, 150.3, 137.0, 136.2, 131.3, 129.6, 128.4, 128.2, 123.7, 115.2 (t, *J* = 251.0 Hz), 63.1, 47.6 (t, *J* = 3.8 Hz), 38.0 (t, *J* = 23.5 Hz), 13.9 ppm.

^{19}F NMR (376 MHz, $CDCl_3$): δ = -104.00 (dt, *J* = 260.0, 16.6 Hz), -104.85 (dt, *J* = 260.0, 16.6 Hz) ppm.
HRMS (APCI/QTOF): *m/z*: $[M + H]^+$ Calcd. for $C_{18}H_{18}F_2NO_3^+$: 334.1249; Found 334.1234.
IR (ATR): 1766, 1689, 1585, 1304, 1264, 1024, 621 cm^{-1}.
$[\alpha]_D^{20}$ = +43.0 (c = 0.25, $CHCl_3$).
Chiral HPLC: (Chiralpak IA, 5 % *i*PrOH/hexane, 1.0 mL/min, 210 nm): tR (minor) 8.97 min, tR (major) 10.56 min, 93:7 *er*.

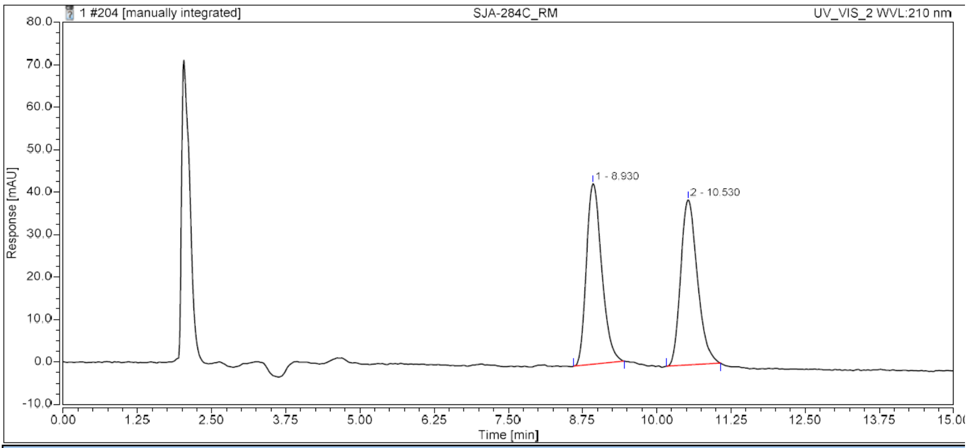

Integration Results

No.	Peak Name	Retention Time min	Area mAU*min	Height mAU	Relative Area %	Relative Height %	Amount n.a.
1		8.930	12.714	42.535	50.04	52.22	n.a.
2		10.530	12.693	38.918	49.96	47.78	n.a.
Total:			25.407	81.453	100.00	100.00	

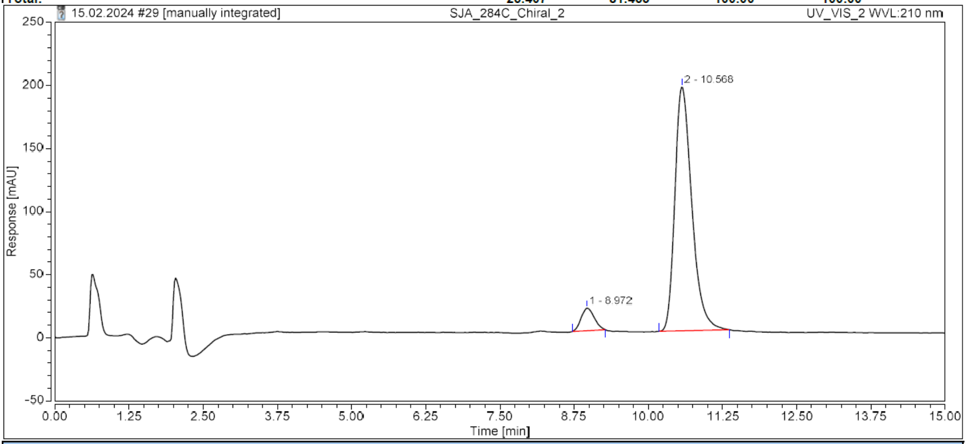

Integration Results

No.	Peak Name	Retention Time min	Area mAU*min	Height mAU	Relative Area %	Relative Height %	Amount n.a.
1		8.972	4.591	17.912	6.68	8.48	n.a.
2		10.568	64.154	193.427	93.32	91.52	n.a.
Total:			68.745	211.339	100.00	100.00	

Ethyl (*S*)-5-(9-ethyl-9H-carbazol-3-yl)-2,2-difluoro-5-oxo-4-phenylpentanoate (4t)

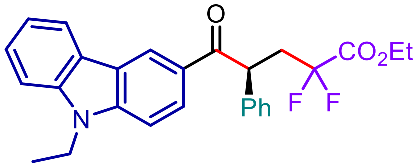

The title compound was synthesized according to the general procedure (**GP 9**) and was obtained after silica gel column chromatography (*n*-pentane : ethylacetate 9:1) as a colorless solid (72 % Yield, 32 mg, 98 % ee). **R_f** = 0.28 (*n*-pentane : ethylacetate 9:1).
MP: 187-188 °C
^{1}H NMR (400 MHz, Chloroform-*d*): δ = 8.77 (d, *J* = 1.8 Hz, 1H), 8.17 – 8.06 (m, 2H), 7.52 – 7.46 (m, 1H), 7.42 – 7.37 (m, 3H), 7.35 – 7.24 (m, 4H), 7.21 – 7.16 (m, 1H), 5.12 (dd, *J* = 7.8, 5.2 Hz, 1H), 4.33 (q, *J* = 7.2 Hz, 2H), 4.16 (dq, *J* = 10.8, 7.2 Hz, 1H), 4.04 (dq, *J* = 10.8, 7.1 Hz, 1H), 3.41 – 3.26 (m, 1H), 2.69 – 2.54 (m, 1H), 1.40 (t, *J* = 7.2 Hz, 3H), 1.21 (t, *J* = 7.1 Hz, 3H) ppm.

^{13}C NMR (101 MHz, Chloroform-*d*): δ = 197.0, 164.3 (t, *J* = 32.7 Hz), 143.2, 141.0, 139.1, 129.5, 128.7, 127.9, 127.5, 127.4, 126.9, 123.6, 123.2, 122.9, 121.1, 120.5, 115.9 (t, *J* = 250.5 Hz), 109.4, 108.5, 63.3, 47.1 (t, *J* = 3.8 Hz), 38.8 (t, *J* = 23.3 Hz), 38.2, 14.23, 14.20 ppm.
^{19}F NMR (376 MHz, $CDCl_3$): δ = -103.81 (dt, *J* = 258.6, 16.7 Hz), -104.58 (dt, *J* = 258.6, 16.7 Hz) ppm.
HRMS (ESI/QTOF): *m/z*: $[M + H]^+$ Calcd. for $C_{27}H_{26}F_2NO_3^+$ 450.1875; Found 450.1878.
IR (ATR): 1765, 1668, 1625, 1592, 1437, 1233, 1072, 848, 720 cm^{-1}.
$[\alpha]_D^{20}$ = +32.5 (c = 0.5, $CHCl_3$).
Chiral HPLC: (Chiralpak IC, 15 % *i*PrOH/hexane, 1.0 mL/min, 210 nm): tR (major) 7.98 min, tR (minor) 14.66 min, 99:1 *er*.

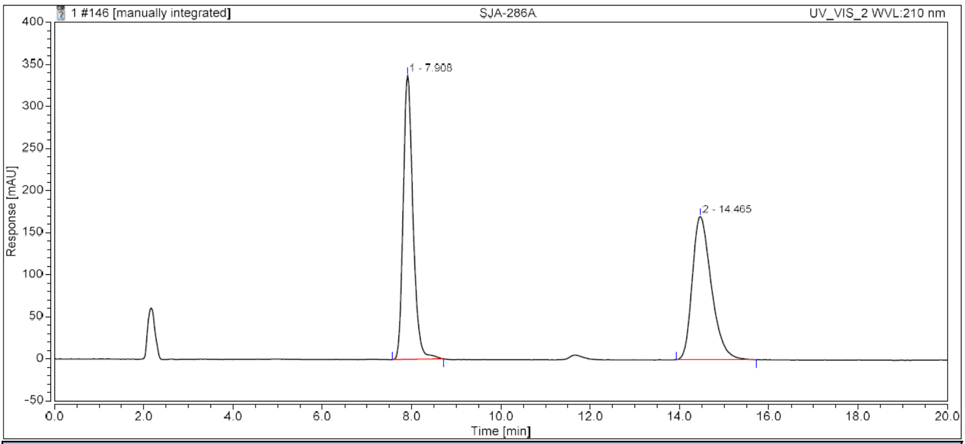

Integration Results							
No.	Peak Name	Retention Time min	Area mAU*min	Height mAU	Relative Area %	Relative Height %	Amount n.a.
1		7.908	85.717	337.871	50.38	66.49	n.a.
2		14.465	84.419	170.264	49.62	33.51	n.a.
Total:			170.136	508.135	100.00	100.00	

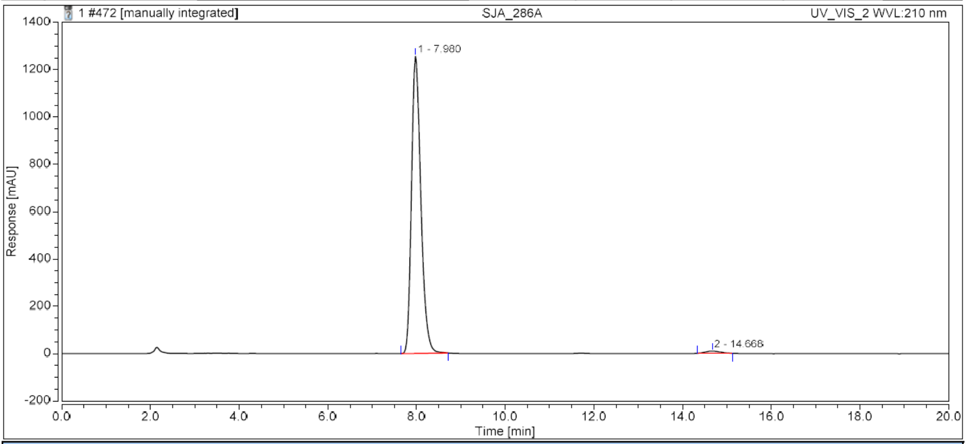

Integration Results							
No.	Peak Name	Retention Time min	Area mAU*min	Height mAU	Relative Area %	Relative Height %	Amount n.a.
1		7.980	303.995	1255.162	98.78	99.27	n.a.
2		14.668	3.766	9.231	1.22	0.73	n.a.
Total:			307.761	1264.393	100.00	100.00	

Ethyl (*S*)-2,2-difluoro-5-oxo-4-phenylhexanoate (4u)

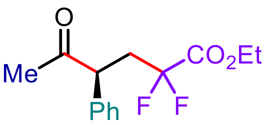

The title compound was synthesized according to the general procedure (**GP 9**) and was obtained after silica gel column chromatography (*n*-pentane : ethylacetate 20:1) as a pale yellow oil (73 % Yield, 20 mg, 90% ee). **R_f** = 0.18 (*n*-pentane : ethylacetate 20:1).
^1H NMR (400 MHz, Chloroform-*d*): δ = 7.37 – 7.25 (m, 3H), 7.22 – 7.18 (m, 2H), 4.20 – 4.03 (m, 2H), 4.03 – 3.97 (m, 1H), 3.17 – 3.01 (m, 1H), 2.45 – 2.28 (m, 1H), 2.07 (s, 3H), 1.27 (t, *J* = 7.2 Hz, 3H) ppm.

^{13}**C NMR** (101 MHz, Chloroform-*d*): δ = 205.5, 163.9 (t, *J* = 32.4 Hz), 137.4, 129.3, 128.4, 128.0, 115.4 (t, *J* = 250.4 Hz), 63.0, 52.6 (t, *J* = 3.8 Hz), 36.7 (t, *J* = 23.6 Hz), 28.8, 13.9 ppm.
^{19}**F NMR** (377 MHz, Chloroform-*d*): δ = -104.10 (dt, *J* = 258.8, 16.2 Hz), -105.17 (dt, *J* = 259.0, 16.8 Hz) ppm.
HRMS (APCI/QTOF): *m/z*: $[M + H]^+$ Calcd. for $C_{14}H_{17}F_2O_3^+$ 271.1140; Found 271.1136.
IR (ATR): 1765, 1718, 1257, 1213, 1014, 772, 749 cm^{-1}.
$[\alpha]_D^{20}$ = +98.0 (c = 0.5, $CHCl_3$).
Chiral HPLC: (Chiralpak IC, 1 % *i*PrOH/hexane, 1.0 mL/min, 210 nm): tR (major) 9.30 min, tR (minor) 11.14 min, 95:5 *er.*

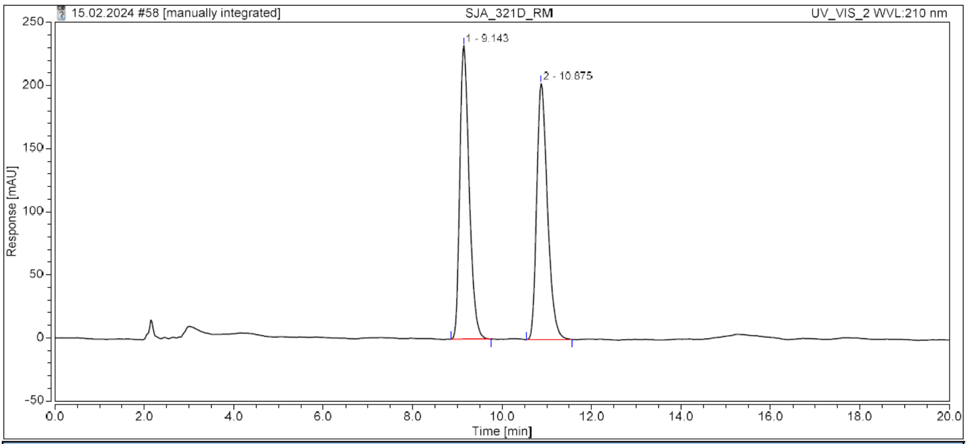

Integration Results

No.	Peak Name	Retention Time min	Area mAU*min	Height mAU	Relative Area %	Relative Height %	Amount n.a.
1		9.143	56.520	232.532	49.97	53.40	n.a.
2		10.875	56.585	202.961	50.03	46.60	n.a.
**Total:**			**113.105**	**435.493**	**100.00**	**100.00**	

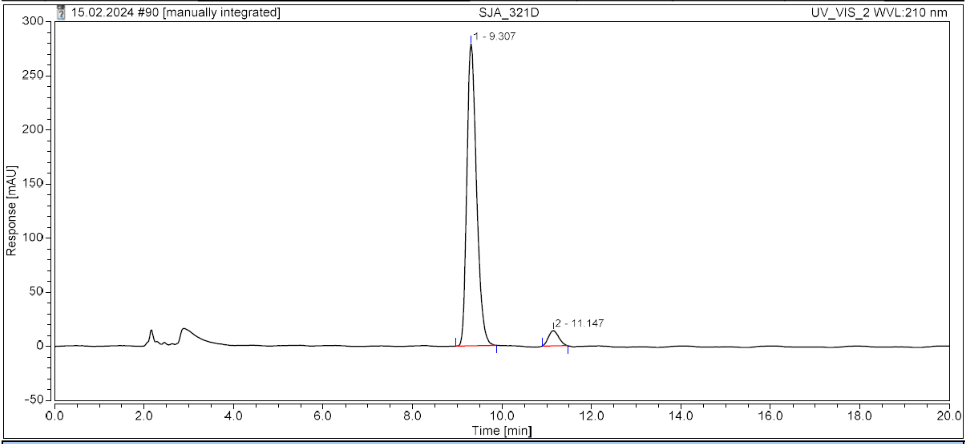

Integration Results

No.	Peak Name	Retention Time min	Area mAU*min	Height mAU	Relative Area %	Relative Height %	Amount n.a.
1		9.307	70.077	278.960	94.93	95.18	n.a.
2		11.147	3.744	14.116	5.07	4.82	n.a.
**Total:**			**73.821**	**293.076**	**100.00**	**100.00**	

Ethyl (*S*)-2,2-difluoro-5-oxo-4-phenylheptanoate (4v)

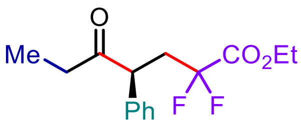

The title compound was synthesized according to the general procedure (**GP 9**) and was obtained after silica gel column chromatography (*n*-pentane : ethylacetate 20:1) as a pale yellow oil (70 % Yield, 20 mg, 90% ee). $\mathbf{R_f}$ = 0.22 (*n*-pentane : ethylacetate 20:1).
^{1}**H NMR** (400 MHz, Chloroform-*d*): δ = 7.36 – 7.23 (m, 3H), 7.22 – 7.15 (m, 2H), 4.21 – 4.05 (m, 2H), 4.01 (dd, *J* = 7.6, 5.4 Hz, 1H), 3.19 – 3.03 (m, 1H), 2.45 – 2.29 (m, 3H), 1.28 (t, *J* = 7.2 Hz, 3H), 0.96 (t, *J* = 7.3 Hz, 3H) ppm.

^{13}C NMR (101 MHz, Chloroform-*d*): δ = 208.3, 163.8 (t, *J* = 32.7 Hz), 137.5, 129.1, 128.2, 127.8, 115.2 (t, *J* = 250.4 Hz), 62.9, 51.4 (t, *J* = 3.8 Hz), 36.8 (t, *J* = 23.5 Hz), 34.7, 13.8, 7.8 ppm.
^{19}F NMR (376 MHz, Chloroform-*d*): δ = -104.17 (dt, *J* = 258.6, 16.2 Hz), -105.27 (dt, *J* = 258.7, 16.9 Hz) ppm.
HRMS (ESI/QTOF): *m/z*: [M + Na]$^+$ Calcd. for $C_{15}H_{18}F_2NaO_3^+$ 307.1116; Found 307.1126.
IR (ATR): 1766, 1718, 1233, 1099, 1074, 1045, 701 cm^{-1}.
$[\alpha]_D^{20}$ = +83.0 (c = 0.5, $CHCl_3$).
Chiral HPLC: (Chiralpak IC, 1 % *i*PrOH/hexane, 1.0 mL/min, 254 nm): tR (major) 12.94 min, tR (minor) 15.99 min, 95:5 *er.*

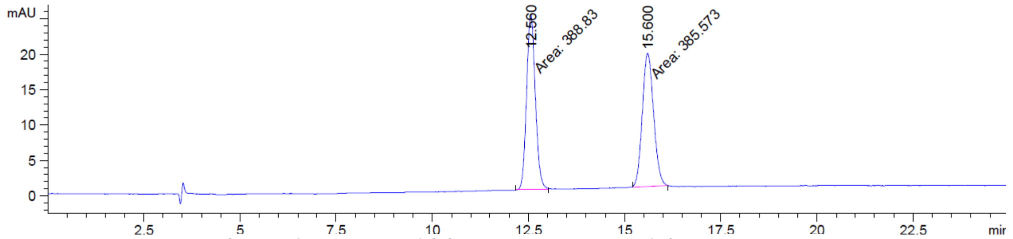

Peak #	RetTime [min]	Type	Width [min]	Area [mAU*s]	Height [mAU]	Area %
1	12.560	MM	0.2624	388.83023	24.69400	50.2103
2	15.600	MM	0.3409	385.57275	18.84844	49.7897

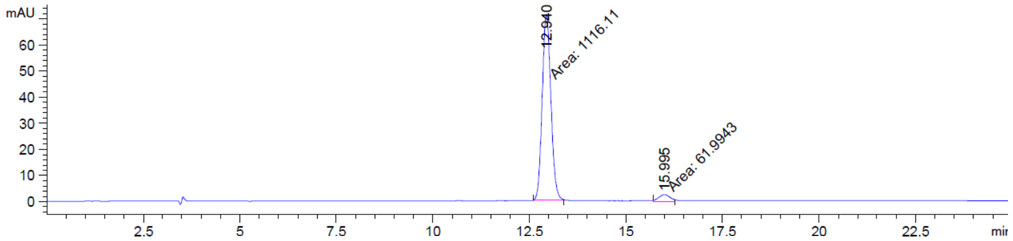

Peak #	RetTime [min]	Type	Width [min]	Area [mAU*s]	Height [mAU]	Area %
1	12.940	MM	0.2610	1116.10510	71.26011	94.7378
2	15.995	MM	0.3659	61.99434	2.82364	5.2622

Ethyl (*S*)-2,2-difluoro-6-methyl-5-oxo-4-phenylheptanoate (4w)

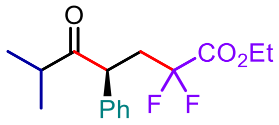

The title compound was synthesized according to the general procedure (**GP 9**) and was obtained after silica gel column chromatography (*n*-pentane : ethylacetate 20:1) as a pale yellow oil (68 % Yield, 20 mg, 96% ee). **R$_f$** = 0.25 (*n*-pentane : ethylacetate 20:1).
^{1}H NMR (400 MHz, Chloroform-*d*): δ = 7.35 – 7.26 (m, 3H), 7.21 – 7.17 (m, 2H), 4.25 – 3.99 (m, 3H), 3.18 – 2.98 (m, 1H), 2.65 (hept, *J* = 6.9 Hz, 1H), 2.44 – 2.26 (m, 1H), 1.28 (t, *J* = 7.1 Hz, 3H), 1.11 (d, *J* = 7.1 Hz, 3H), 0.87 (d, *J* = 6.7 Hz, 3H) ppm.
^{13}C NMR (101 MHz, Chloroform-*d*): δ = 211.4, 163.8 (t, *J* = 32.6 Hz), 137.4, 129.1, 128.4, 127.8, 115.3 (t, *J* = 250.6 Hz), 62.8, 49.9 (t, *J* = 3.6 Hz), 39.5, 37.0 (t, *J* = 23.3 Hz), 19.0, 18.2, 13.8 ppm.
^{19}F NMR (376 MHz, Chloroform-*d*): δ = -104.20 (ddd, *J* = 259.4, 18.1, 14.0 Hz), -104.93 – -105.76 (m) ppm.
HRMS (ESI/QTOF): *m/z*: [M + Na]$^+$ Calcd. for $C_{16}H_{20}F_2NaO_3^+$ 321.1273; Found 321.1279.
IR (ATR): 1763, 1715. 1052, 722, 606 cm^{-1}.
$[\alpha]_D^{20}$ = +87.0 (c = 0.6, $CHCl_3$).
Chiral HPLC: (Chiralpak IC, 1 % *i*PrOH/hexane, 1.0 mL/min, 254 nm): tR (minor) 11.80 min, tR (major) 13.37 min, 98:2 *er.*

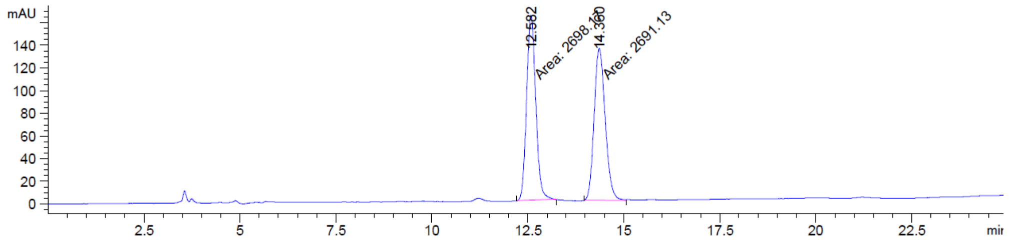

Peak #	RetTime [min]	Type	Width [min]	Area [mAU*s]	Height [mAU]	Area %
1	12.582	MM	0.2763	2698.16553	162.75337	50.0652
2	14.360	MM	0.3351	2691.13477	133.83632	49.9348

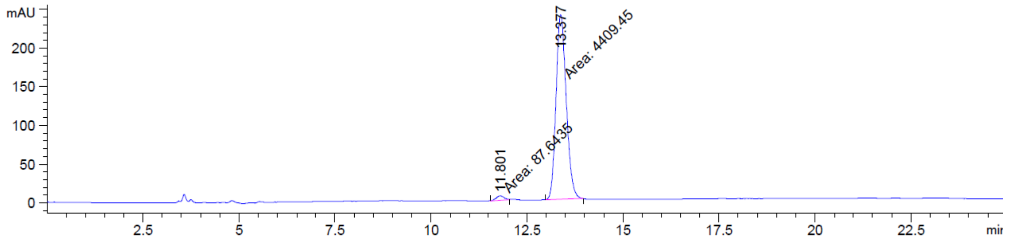

Peak #	RetTime [min]	Type	Width [min]	Area [mAU*s]	Height [mAU]	Area %
1	11.801	MM	0.2468	87.64351	5.91981	1.9489
2	13.377	MM	0.3079	4409.44971	238.70168	98.0511

Ethyl (*S*)-5-cyclopropyl-2,2-difluoro-5-oxo-4-phenylpentanoate (4x)

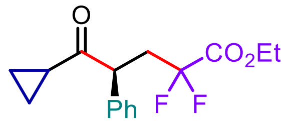

The title compound was synthesized according to the general procedure (**GP 9**) and was obtained after silica gel column chromatography (*n*-pentane : ethylacetate 20:1) as a pale yellow oil (70 % Yield, 21 mg, 92% ee). **R_f** = 0.26 (*n*-pentane : ethylacetate 20:1).

^{1}H NMR (400 MHz, Chloroform-*d*): δ = 7.36 – 7.26 (m, 3H), 7.23 – 7.18 (m, 2H), 4.18 – 4.00 (m, 3H), 3.17 – 3.01 (m, 1H), 2.46 – 2.29 (m, 1H), 1.89 – 1.79 (m, 1H), 1.26 (t, *J* = 7.2 Hz, 3H), 1.05 – 0.98 (m, 1H), 0.96 – 0.82 (m, 2H), 0.74 – 0.66 (m, 1H) ppm.

^{13}C NMR (101 MHz, Chloroform-*d*): δ = 207.6, 163.8 (t, *J* = 32.6 Hz), 137.5, 129.1, 128.6, 127.7, 115.3 (t, *J* = 250.3 Hz), 62.8, 52.5 (t, *J* = 3.9 Hz), 36.6 (t, *J* = 23.6 Hz), 20.3, 13.8, 11.8, 11.5 ppm.

^{19}F NMR (376 MHz, Chloroform-*d*): δ = -103.95 (dt, *J* = 259.2, 16.2 Hz), -105.05 (dt, *J* = 258.8, 16.9 Hz) ppm.

HRMS (ESI/QTOF): *m/z*: $[M + Na]^+$ Calcd. for $C_{16}H_{18}F_2NaO_3^+$ 319.1116; Found 319.1120.

IR (ATR): 1765, 1697, 1193, 1038, 666, cm^{-1}.

$[\alpha]_D^{20}$ = +94.0 (c = 0.8, $CHCl_3$).

Chiral HPLC: (Chiralpak IC, 1 % *i*PrOH/hexane, 1.0 mL/min, 210 nm): tR (minor) 10.34 min, tR (major) 11.67 min, 96:4 *er.*

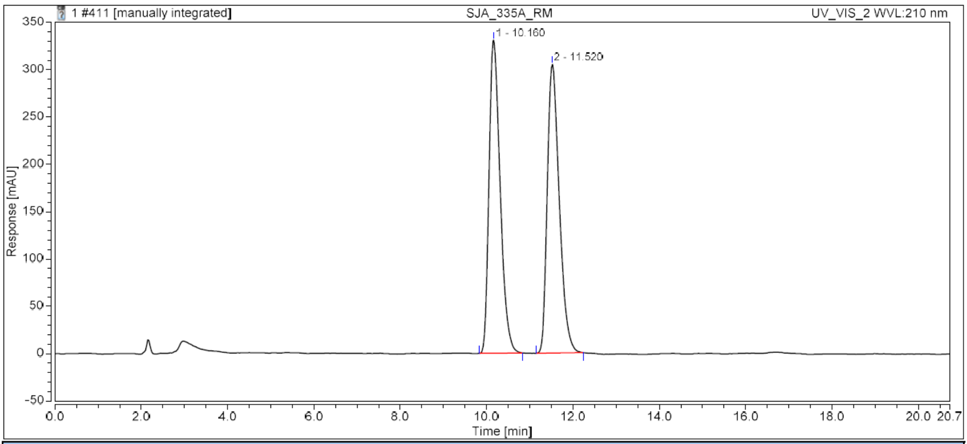

Integration Results							
No.	Peak Name	Retention Time min	Area mAU*min	Height mAU	Relative Area %	Relative Height %	Amount n.a.
1		10.160	98.694	330.854	50.10	52.00	n.a.
2		11.520	98.318	305.353	49.90	48.00	n.a.
Total:			197.012	636.207	100.00	100.00	

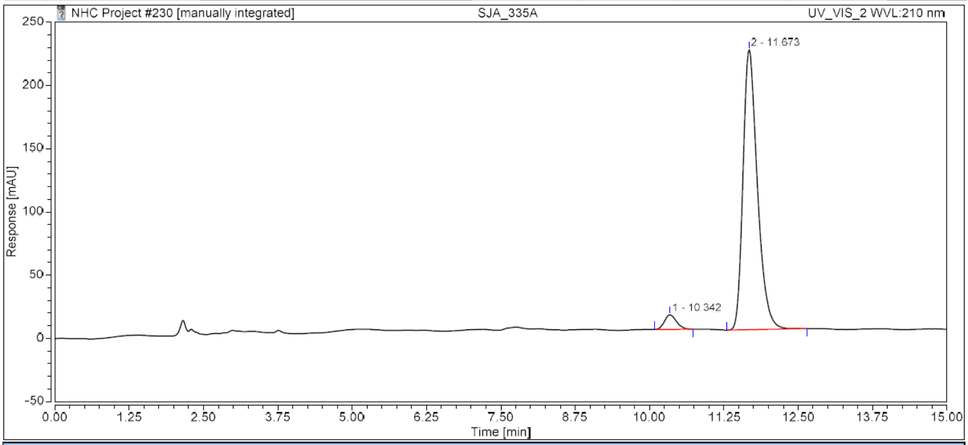

Integration Results							
No.	Peak Name	Retention Time min	Area mAU*min	Height mAU	Relative Area %	Relative Height %	Amount n.a.
1		10.342	2.666	11.466	4.11	4.92	n.a.
2		11.673	62.153	221.475	95.89	95.08	n.a.
Total:			64.819	232.941	100.00	100.00	

Ethyl (4*S*)-5-(2,2-dimethylcyclopropyl)-2,2-difluoro-5-oxo-4-phenylpentanoate (4y)

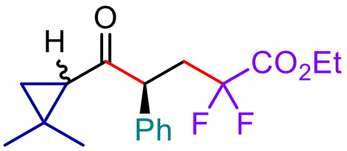

The title compound was synthesized according to the general procedure (**GP 9**) and was obtained after silica gel column chromatography (*n*-pentane : ethylacetate 30:1) as a colorless oil (41 % Yield, 13.3 mg, 2.5:1 d.r., major 32%ee, minor 48% ee). $\mathbf{R_f}$ = 0.28 (*n*-pentane : ethylacetate 30:1).

^{1}H NMR (400 MHz, Chloroform-*d*): δ = 7.44 – 7.13 (m, 5H), 4.23 – 3.95 (m, 3H), 3.21 – 2.99 (m, 1H), 2.53 – 2.28 (m, 1H), 1.74 (ddd, *J* = 12.8, 7.5, 5.6 Hz, 1H), 1.30 – 1.24 (m, 3H), 1.23 – 1.18 (m, 1H)1.16 (s, 2H), 1.11 (s, 2H), 0.81 (dd, *J* = 7.4, 4.1 Hz, 0.29H, minor), 0.76 (s, 0.8H), 0.73 (dd, *J* = 7.6, 4.0 Hz, 0.71H, major), 0.58 (s, 0.8H) ppm.

^{13}C NMR (101 MHz, Chloroform-*d*): δ = 204.9, 204.1, 163.9 (t, *J* = 32.7 Hz), 163.8 (t, *J* = 32.6 Hz), 137.9, 136.8, 129.1, 128.7, 128.5, 128.5, 127.6, 127.6, 115.5 (t, *J* = 250.0 Hz), 115.3 (t, *J* = 250.5 Hz), 62.8, 53.3(t, *J* = 3.6 Hz), 53.2 (t, *J* = 4.1 Hz), 36.5 (t, *J* = 23.3 Hz), 35.9 (t, *J* = 23.6 Hz), 35.6, 34.7, 29.7, 27.9, 27.7, 27.1, 26.3, 24.0, 23.2, 18.2, 17.7, 13.8, 13.7 ppm.

^{19}F NMR (376 MHz, Chloroform-*d*): δ = -103.43 – -105.66 (m) ppm.

HRMS (ESI/QTOF) m/z: $[M + Na]^+$ Calcd for $C_{18}H_{22}F_2NaO_3^+$ 347.1429; Found 347.1440.

IR (ATR): 2926, 1764, 1698, 1096, 759 cm^{-1}.

$[\alpha]_D^{20}$ = +13.2 (c = 0.3, $CHCl_3$).

Chiral HPLC (major diastereomer): (Chiralpak IG, 1 % *i*PrOH/hexane, 1.0 mL/min, 210 nm): tR (major) 9.54 min, tR (minor) 10.36 min, 66:34 *er.*

Chiral HPLC (minor diastereomer): (Chiralpak IG, 1 % *i*PrOH/hexane, 1.0 mL/min, 210 nm): tR (major) 12.01 min, tR (minor) 22.06 min, 74:26 *er.*

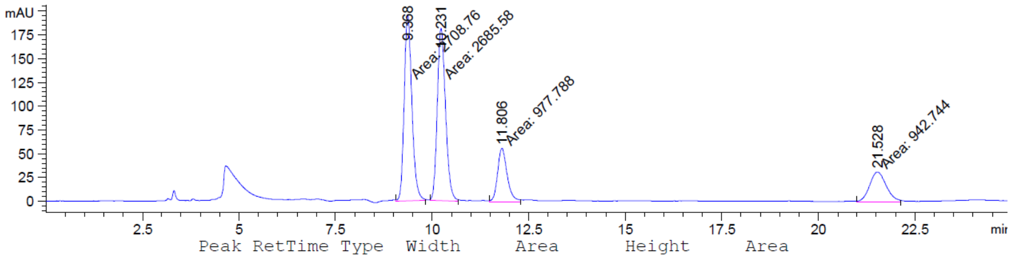

Peak #	RetTime [min]	Type	Width [min]	Area [mAU*s]	Height [mAU]	Area %
1	9.368	MM	0.2308	2708.76270	195.58315	37.0309
2	10.231	MM	0.2476	2685.58276	180.77335	36.7140
3	11.806	MM	0.2891	977.78833	56.36652	13.3671
4	21.528	MM	0.5012	942.74438	31.34852	12.8880

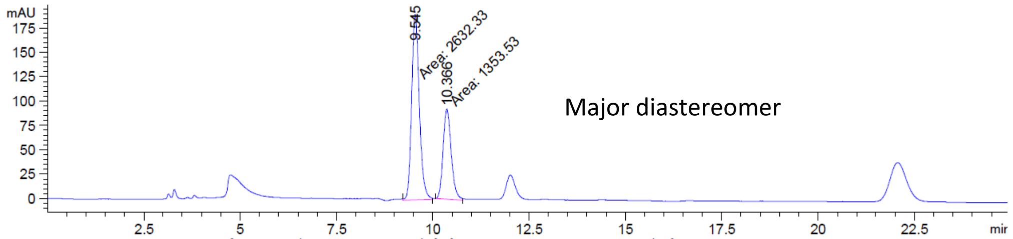

Peak #	RetTime [min]	Type	Width [min]	Area [mAU*s]	Height [mAU]	Area %
1	9.545	MM	0.2302	2632.33008	190.60245	66.0417
2	10.366	MM	0.2440	1353.52832	92.45497	33.9583

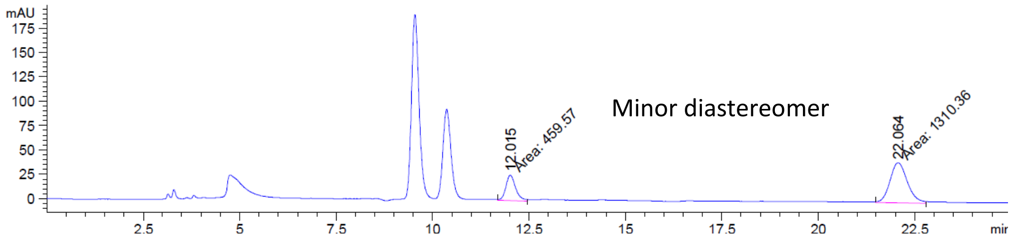

Peak #	RetTime [min]	Type	Width [min]	Area [mAU*s]	Height [mAU]	Area %
1	12.015	MM	0.2941	459.56973	26.04015	25.9654
2	22.064	MM	0.5263	1310.36304	41.49540	74.0346

Ethyl (S)-2,2-difluoro-4-(4-fluorophenyl)-5-oxo-5-phenylpentanoate (6a)

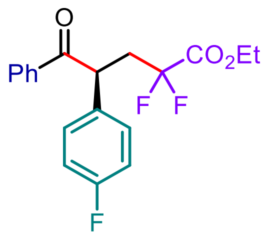

The title compound was synthesized according to the general procedure (**GP 9**) and was obtained after silica gel column chromatography (*n*-pentane : ethylacetate 20:1) as a coloreless oil (87 % Yield, 30 mg, 99% ee). $\mathbf{R_f}$ = 0.28 (*n*-pentane : ethylacetate 20:1).

^{1}H NMR (400 MHz, Chloroform-*d*): δ = 7.97 – 7.91 (m, 2H), 7.55 – 7.49 (m, 1H), 7.45 – 7.38 (m, 2H), 7.30 – 7.25 (m, 2H), 7.03 – 6.94 (m, 2H), 4.96 (dd, *J* = 7.8, 5.2 Hz, 1H), 4.20 (dq, *J* = 10.7, 7.2 Hz, 1H), 4.10 (dq, *J* = 10.7, 7.1 Hz, 1H), 3.32 – 3.15 (m, 1H), 2.60 – 2.45 (m, 1H), 1.25 (t, *J* = 7.1 Hz, 3H) ppm.

^{13}C NMR (101 MHz, Chloroform-*d*): δ = 197.0, 163.5 (t, *J* = 32.6 Hz), 162.0 (d, *J* = 247.1 Hz), 135.4, 133.3 (d, *J* = 3.6 Hz), 133.2, 129.7 (d, *J* = 8.3 Hz), 128.6, 128.5, 116.0 (d, *J* = 21.9 Hz), 115.0 (t, *J* = 251.0 Hz), 62.8, 45.7 (t, *J* = 3.9 Hz), 37.9 (t, *J* = 23.3 Hz), 13.5 ppm.
^{19}F NMR (376 MHz, Chloroform-*d*): δ = -104.41 (td, *J* = 16.7, 12.2 Hz), -114.21 – -114.36 (m) ppm.
HRMS (ESI/QTOF): *m/z*: [M + Na]$^+$ Calcd. for $C_{19}H_{17}F_3NaO_3^+$ 373.1022; Found 373.1026.
IR (ATR): 1763, 1682, 1598, 1580, 1508, 1341, 1208, 1002, 839, 638, 431 cm^{-1}.
$[\alpha]_D^{20}$ = +46.6 (c = 0.3, $CHCl_3$).
Chiral HPLC: (Chiralpak IB, 1 % *i*PrOH/hexane, 1.0 mL/min, 210 nm): tR (major) 4.47 min, tR (minor) 5.15 min, 99.5:0.5 *er.*

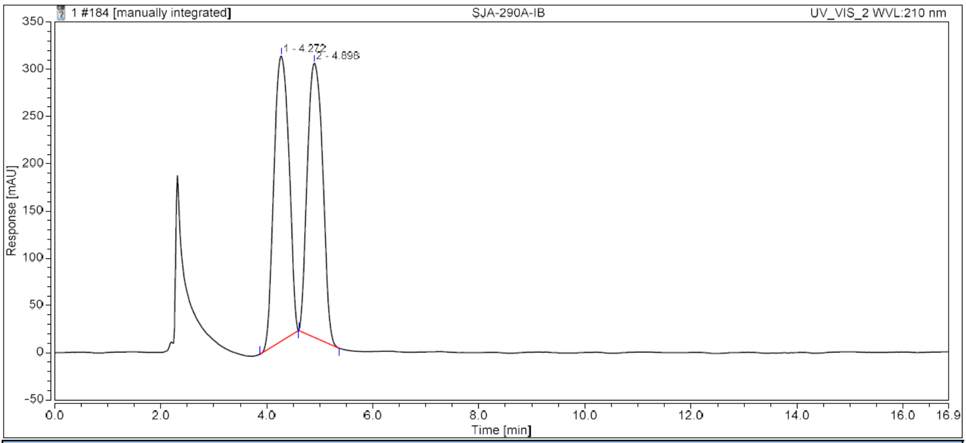

Integration Results							
No.	Peak Name	Retention Time min	Area mAU*min	Height mAU	Relative Area %	Relative Height %	Amount n.a.
1		4.272	99.017	302.156	50.35	51.00	n.a.
2		4.898	97.628	290.340	49.65	49.00	n.a.
Total:			196.644	592.496	100.00	100.00	

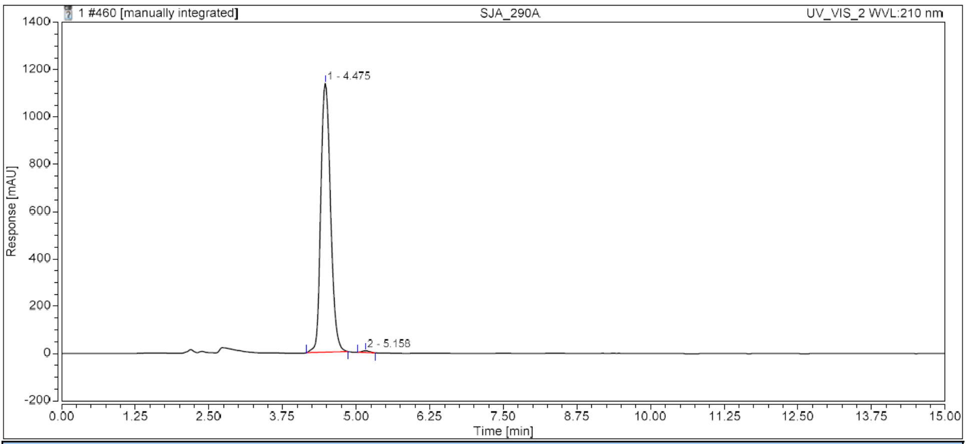

Integration Results							
No.	Peak Name	Retention Time min	Area mAU*min	Height mAU	Relative Area %	Relative Height %	Amount n.a.
1		4.475	217.461	1136.748	99.50	99.39	n.a.
2		5.158	1.084	6.989	0.50	0.61	n.a.
Total:			218.544	1143.737	100.00	100.00	

Ethyl (*S*)-2,2-difluoro-4-(4-methoxyphenyl)-5-oxo-5-phenylpentanoate (6b)

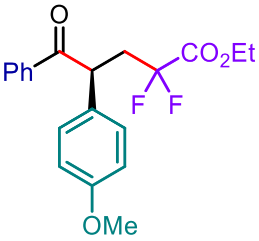

The title compound was synthesized according to the general procedure (**GP 9**) and was obtained after silica gel column chromatography (*n*-pentane : ethylacetate 9:1) as a coloreless oil (85 % Yield, 31 mg, 98% ee). **R$_f$** = 0.24 (*n*-pentane : ethylacetate 9:1).
^{1}H NMR (400 MHz, Chloroform-*d*): δ = 7.97 – 7.88 (m, 2H), 7.50 – 7.43 (m, 1H), 7.42 – 7.34 (m, 2H), 7.21 – 7.15 (m, 2H), 6.84 – 6.75 (m, 2H), 4.88 (dd, *J* = 7.9, 5.2 Hz, 1H), 4.15 (dq, *J* = 10.7, 7.2 Hz, 1H), 4.04 (dq, *J* = 10.8, 7.2 Hz, 1H), 3.72 (s, 3H), 3.30 – 3.31 (m, 1H), 2.58 – 2.43 (m, 1H), 1.21 (t, *J* = 7.2 Hz, 3H) ppm.
^{13}C NMR (101 MHz, Chloroform-*d*): δ = 197.9, 164.2 (t, *J* = 32.7 Hz), 159.5, 136.3, 133.5, 130.0, 129.8, 129.2, 129.0, 115.8 (t, *J* = 250.6 Hz), 115.0, 63.3, 55.6, 46.4 (t, *J* = 3.9 Hz), 38.6 (t, *J* = 23.3 Hz), 14.1 ppm.
^{19}F NMR (376 MHz, Chloroform-*d*): δ = -103.93 (dt, *J* = 259.3, 16.5 Hz), -104.77 (dt, *J* = 259.3, 16.5 Hz) ppm.
HRMS (ESI/QTOF): *m/z*: [M + Na]$^+$ Calcd. for $C_{20}H_{20}F_2NaO_4^+$ 385.1222; Found 385.1229.
IR (ATR): 1765, 1682, 1609, 1511, 1253, 1179, 1092, 690 cm^{-1}.
$[\alpha]_D^{20}$ = +34.2 (c = 0.4, $CHCl_3$).
Chiral HPLC: (Chiralpak IA, 1 % *i*PrOH/hexane, 1.0 mL/min, 210 nm): tR (major) 10.82 min, tR (minor) 14.83 min, 99:1 *er.*

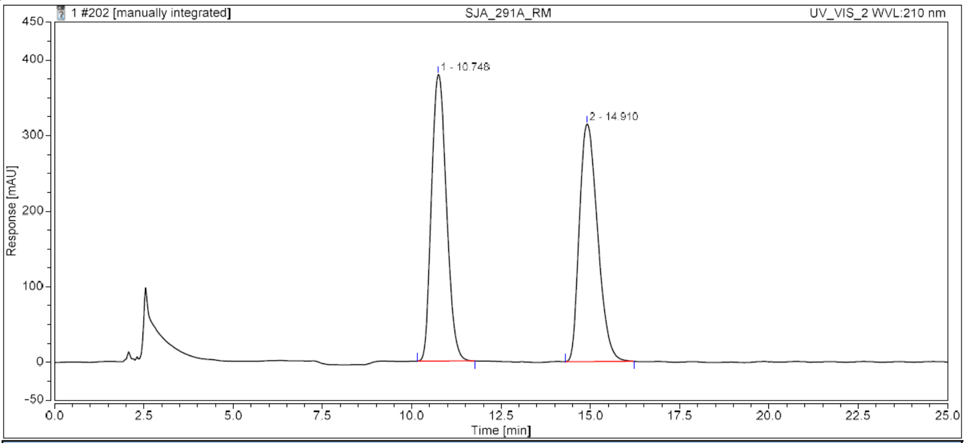

Integration Results							
No.	Peak Name	Retention Time<br>min	Area<br>mAU*min	Height<br>mAU	Relative Area<br>%	Relative Height<br>%	Amount<br>n.a.
1		10.748	184.347	379.509	49.78	54.69	n.a.
2		14.910	186.013	314.395	50.22	45.31	n.a.
Total:			370.361	693.904	100.00	100.00	

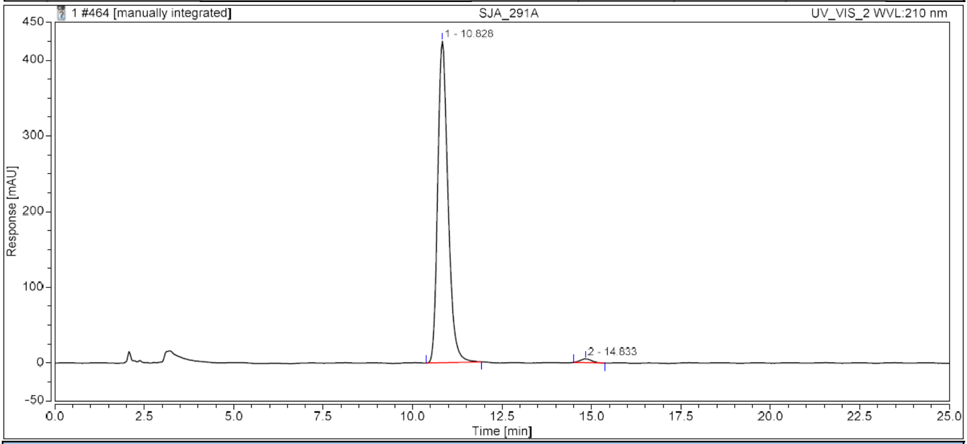

Integration Results							
No.	Peak Name	Retention Time<br>min	Area<br>mAU*min	Height<br>mAU	Relative Area<br>%	Relative Height<br>%	Amount<br>n.a.
1		10.828	141.858	424.724	98.75	98.87	n.a.
2		14.833	1.799	4.856	1.25	1.13	n.a.
Total:			143.656	429.580	100.00	100.00	

Ethyl (*S*)-4-(4-cyanophenyl)-2,2-difluoro-5-oxo-5-phenylpentanoate (6c)

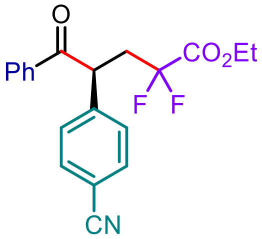

The title compound was synthesized according to the general procedure (**GP 9**) and was obtained after silica gel column chromatography (*n*-pentane : ethylacetate 9:1) as a coloreless oil (69 % Yield, 25 mg, 95% ee). **R$_f$** = 0.2 (*n*-pentane : ethylacetate 9:1).

^{1}H NMR (400 MHz, Chloroform-*d*): δ = 7.97 – 7.87 (m, 2H), 7.62 – 7.57 (m, 2H), 7.57 – 7.52 (m, 1H), 7.46 – 7.40 (m, 4H), 5.05 (dd, *J* = 7.7, 5.2 Hz, 1H), 4.29 – 4.07 (m, 2H), 3.35 – 3.19 (m, 1H), 2.61 – 2.44 (m, 1H), 1.26 (t, *J* = 7.2 Hz, 3H) ppm.

^{13}C NMR (101 MHz, Chloroform-*d*): δ = 196.8, 163.9 (t, *J* = 32.2 Hz), 143.5, 135.7, 134.2, 133.4, 129.5, 129.3, 129.2, 118.7, 115.4 (t, *J* = 251.7 Hz), 112.2, 63.6, 47.0 (t, *J* = 3.5 Hz), 38.3 (t, *J* = 23.3 Hz), 14.2 ppm.

^{19}F NMR (376 MHz, Chloroform-*d*): δ = -103.95 (ddd, *J* = 260.7, 18.2, 14.0 Hz), -104.90 (ddd, *J* = 260.7, 18.4, 15.2 Hz) ppm.

HRMS (ESI/QTOF): *m/z*: [M + Na]$^+$ Calcd. for $C_{20}H_{17}F_2NNaO_3^+$ 380.1069; Found 380.1066.

IR (ATR): 1765, 1685, 1326, 1092, 724, 662 cm^{-1}.

$[\alpha]_D^{20}$ = +89.3 (c = 0.4, $CHCl_3$).

Chiral HPLC: (Chiralpak IB, 3 % *i*PrOH/hexane, 1.0 mL/min, 210 nm): tR (minor) 8.04 min, tR (major) 9.39 min, 97.5:2.5 *er.*

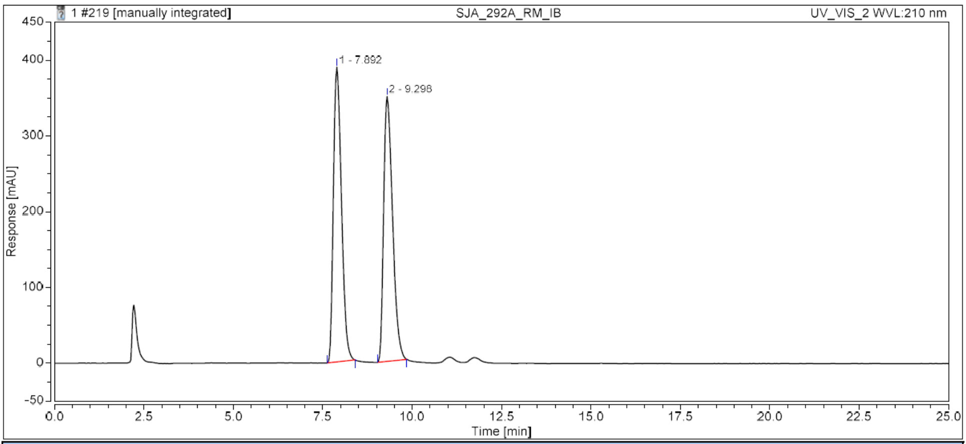

Integration Results

No.	Peak Name	Retention Time min	Area mAU*min	Height mAU	Relative Area %	Relative Height %	Amount n.a.
1		7.892	101.513	389.142	50.12	52.68	n.a.
2		9.298	101.009	349.500	49.88	47.32	n.a.
**Total:**			**202.522**	**738.641**	**100.00**	**100.00**	

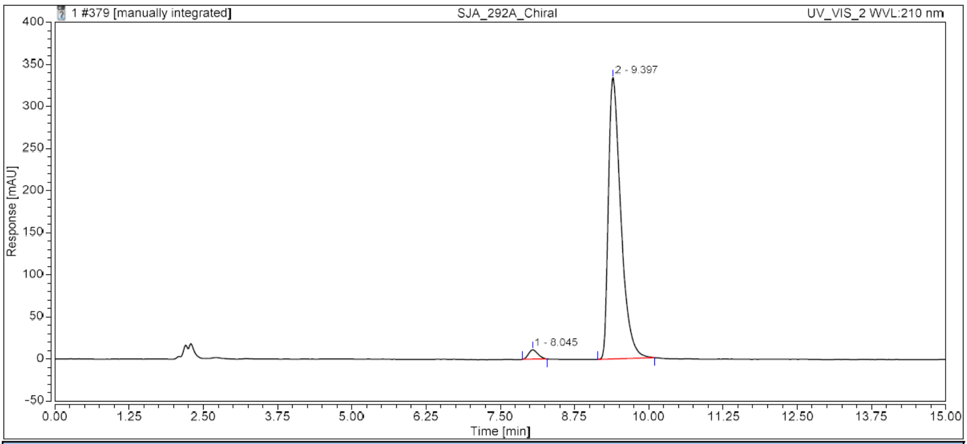

Integration Results

No.	Peak Name	Retention Time min	Area mAU*min	Height mAU	Relative Area %	Relative Height %	Amount n.a.
1		8.045	2.041	11.105	2.47	3.21	n.a.
2		9.397	80.763	334.411	97.53	96.79	n.a.
**Total:**			**82.804**	**345.516**	**100.00**	**100.00**	

Ethyl (*S*)-2,2-difluoro-4-(4-nitrophenyl)-5-oxo-5-phenylpentanoate (6d)

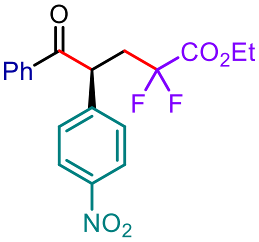

The title compound was synthesized according to the general procedure (**GP 9**) and was obtained after silica gel column chromatography (*n*-pentane : ethylacetate 4:1) as a yellow oil (64 % Yield, 24 mg, 94% ee). **R**$_f$ = 0.20 (*n*-pentane : ethylacetate 4:1).

^{1}H NMR (400 MHz, Chloroform-*d*): δ = 8.18 – 8.14 (m, 2H), 7.96 – 7.91 (m, 2H), 7.57 – 7.48 (m, 3H), 7.46 – 7.40 (m, 2H), 5.12 (dd, *J* = 7.6, 5.3 Hz, 1H), 4.28 – 4.12 (m, 2H), 3.37 – 3.21 (m, 1H), 2.62 – 2.46 (m, 1H), 1.27 (t, *J* = 7.1 Hz, 3H) ppm.

^{13}C NMR (101 MHz, Chloroform-*d*): δ = 196.7, 163.9 (t, *J* = 32.1 Hz), 147.8, 145.5, 135.7, 134.3, 129.6, 129.3, 129.2, 124.8, 115.4 (t, *J* = 251.4 Hz), 63.6, 46.8 (t, *J* = 3.5 Hz), 38.3 (t, *J* = 23.3 Hz), 14.2 ppm.

^{19}F NMR (376 MHz, Chloroform-*d*): δ = -103.8 (ddd, *J* = 260.7, 19.4, 13.8 Hz), -104.9 (ddd, *J* = 261.0, 18.7, 14.7 Hz) ppm.

HRMS (ESI/QTOF): *m/z*: [M + Na]$^+$ Calcd. for $C_{19}H_{17}F_2NNaO_5^+$ 400.0967; Found 400.0964.

IR (ATR): 1765, 1686, 1597, 1523, 1348, 1091, 858, 710 cm^{-1}.

$[\alpha]_D^{20}$ = +74.6 (c = 0.4, $CHCl_3$).

Chiral HPLC: (Chiralpak IB, 3 % *i*PrOH/hexane, 1.0 mL/min, 210 nm): tR (minor) 7.53 min, tR (major) 9.43 min, 97:3 *er*.

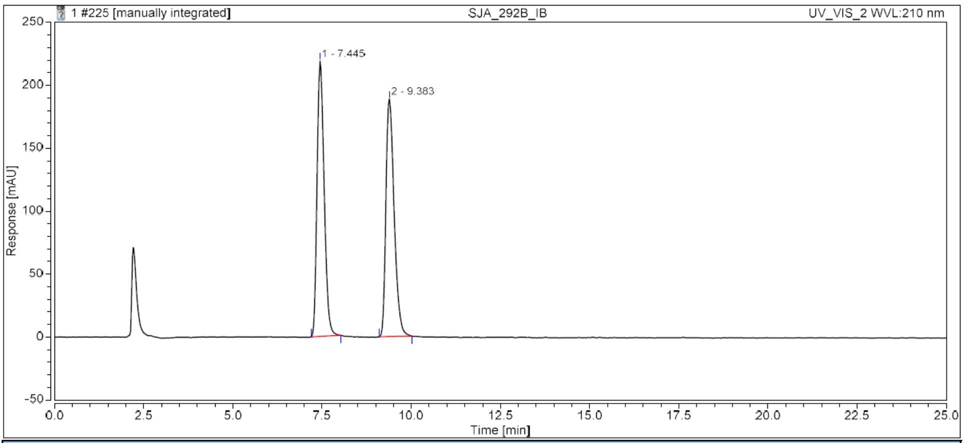

Integration Results

No.	Peak Name	Retention Time min	Area mAU*min	Height mAU	Relative Area %	Relative Height %	Amount n.a.
1		7.445	50.525	218.789	50.01	53.68	n.a.
2		9.383	50.506	188.802	49.99	46.32	n.a.
**Total:**			**101.031**	**407.591**	**100.00**	**100.00**	

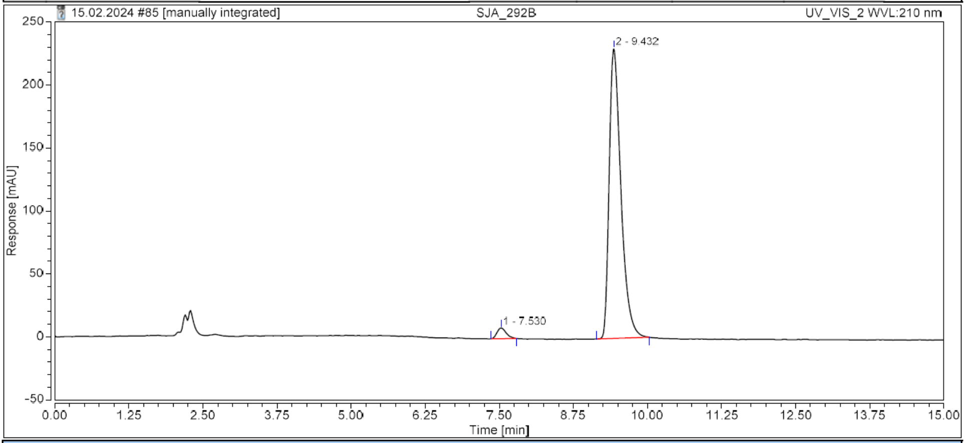

Integration Results

No.	Peak Name	Retention Time min	Area mAU*min	Height mAU	Relative Area %	Relative Height %	Amount n.a.
1		7.530	1.504	8.410	2.78	3.53	n.a.
2		9.432	52.649	229.928	97.22	96.47	n.a.
**Total:**			**54.153**	**238.337**	**100.00**	**100.00**	

Ethyl (*S*)-2,2-difluoro-5-oxo-5-phenyl-4-(*m*-tolyl)pentanoate (6e)

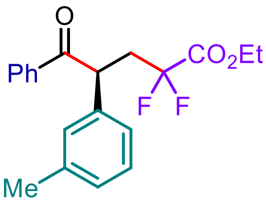

The title compound was synthesized according to the general procedure (**GP 9**), and was obtained after silica gel column chromatography (*n*-pentane : ethylacetate 20:1) as a colorless oil (72 % Yield, 25 mg, 92 % ee). $\mathbf{R_f}$ = 0.26 (*n*-pentane : ethylacetate 20:1).

^{1}H NMR (400 MHz, Chloroform-*d*): δ = 7.98 – 7.93 (m, 2H), 7.53 – 7.46 (m, 1H), 7.43 – 7.36 (m, 2H), 7.21 – 7.14 (m, 1H), 7.11 – 7.07 (m, 2H), 7.04 – 7.01 (m, 1H), 4.91 (dd, *J* = 8.2, 4.8 Hz, 1H), 4.18 (dq, *J* = 10.7, 7.2 Hz, 1H), 4.06 (dq, *J* = 10.8, 7.1 Hz, 1H), 3.35 – 3.19 (m, 1H), 2.57 – 2.43 (m, 1H), 2.29 (s, 3H), 1.23 (t, *J* = 7.2 Hz, 3H) ppm.

^{13}C NMR (101 MHz, Chloroform-*d*): δ = 197.8, 164.2 (t, *J* = 32.7 Hz), 139.4, 138.1, 136.3, 133.6, 129.5, 129.3, 129.1, 129.0, 128.9, 125.8, 115.7 (t, *J* = 254.8 Hz), 63.3, 47.2 (t, *J* = 4.0 Hz), 38.7 (t, *J* = 23.3 Hz), 21.8, 14.1 ppm.

^{19}F NMR (376 MHz, Chloroform-*d*): δ = -104.41 (td, *J* = 16.5, 9.1 Hz) ppm.

HRMS (ESI/QTOF): *m/z*: $[M + Na]^+$ Calcd. for $C_{20}H_{20}F_2NaO_3^+$ 369.1273; Found 369.1270.

IR (ATR): 1764, 1682, 1597, 1580, 1489, 1319, 1046, 738, 595, 445 cm^{-1}.

$[\alpha]_D^{20}$ = +51.4 (c = 0.7, $CHCl_3$).
Chiral HPLC: (Chiralpak IC, 1 % *i*PrOH/hexane, 1.0 mL/min, 210 nm): tR (minor) 9.80 min, tR (major) 13.56 min, 96:4 *er.*

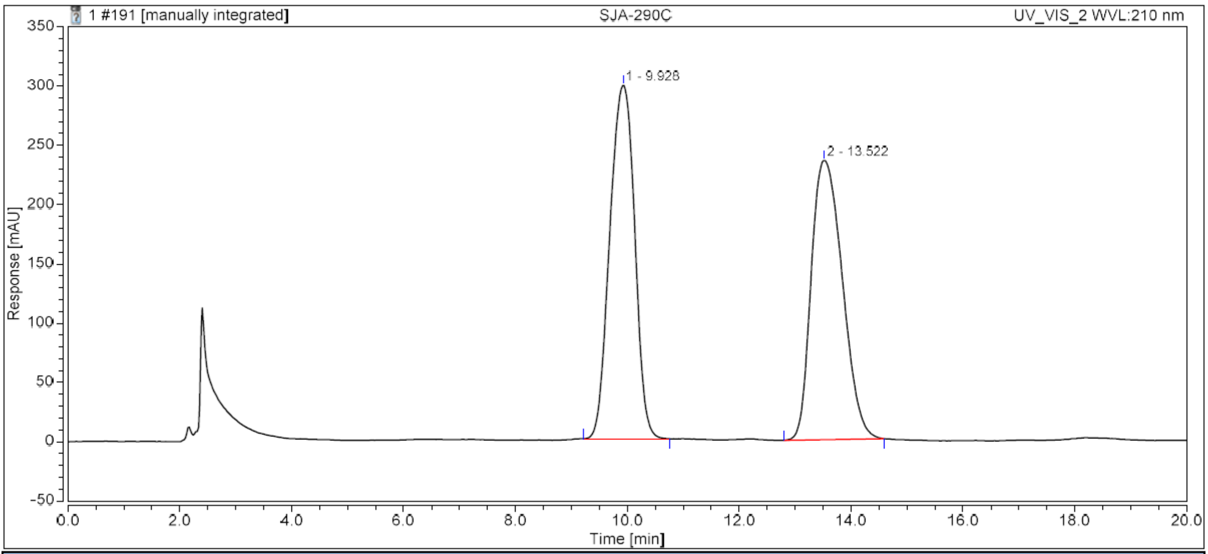

Integration Results							
No.	Peak Name	Retention Time min	Area mAU*min	Height mAU	Relative Area %	Relative Height %	Amount n.a.
1		9.928	153.288	298.254	50.24	55.87	n.a.
2		13.522	151.816	235.615	49.76	44.13	n.a.
**Total:**			**305.103**	**533.869**	**100.00**	**100.00**	

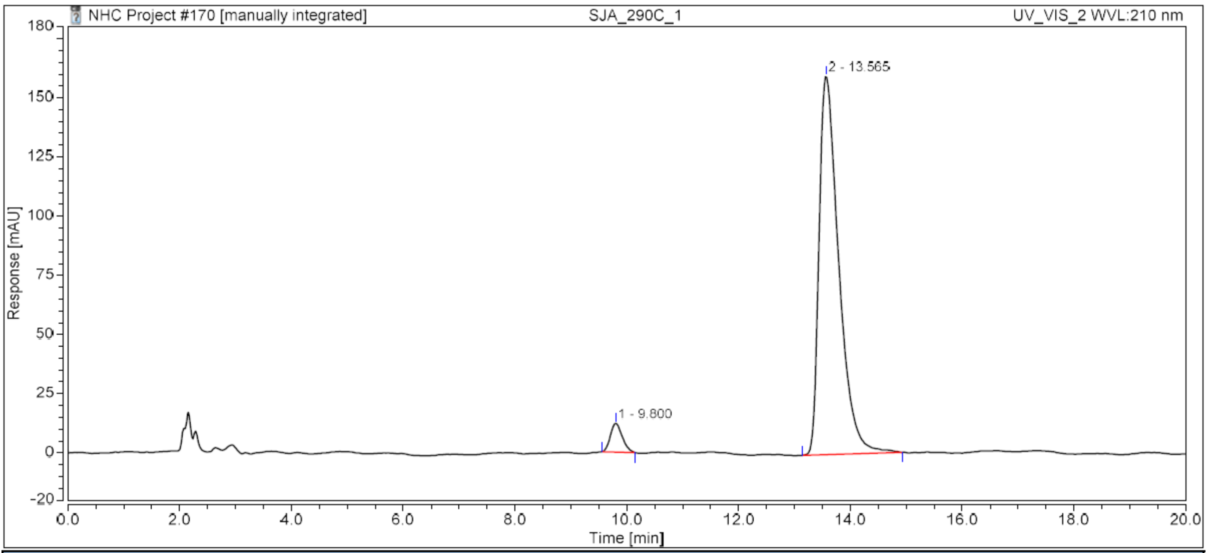

Integration Results							
No.	Peak Name	Retention Time min	Area mAU*min	Height mAU	Relative Area %	Relative Height %	Amount n.a.
1		9.800	2.975	12.136	4.37	7.05	n.a.
2		13.565	65.107	159.906	95.63	92.95	n.a.
**Total:**			**68.082**	**172.041**	**100.00**	**100.00**	

Ethyl (*S*)-2,2-difluoro-5-oxo-5-phenyl-4-(*o*-tolyl)pentanoate (6f)

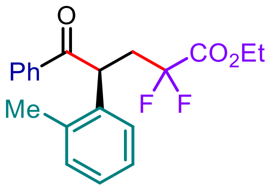

The title compound was synthesized according to the general procedure (**GP 9**), and was obtained after silica gel column chromatography (*n*-pentane : ethylacetate 20:1) as a colorless oil (60 % Yield, 21 mg, 94 % ee). **R$_f$** = 0.26 (*n*-pentane : ethylacetate 20:1).

^{1}H NMR (400 MHz, Chloroform-*d*): δ = 7.87 – 7.79 (m, 2H), 7.51 – 7.45 (m, 1H), 7.41 – 7.35 (m, 2H), 7.24 – 7.19 (m, 1H), 7.16 – 7.01 (m, 3H), 5.09 (dd, *J* = 9.0, 3.5 Hz, 1H), 4.22 (dq, *J* = 10.8, 7.2 Hz, 1H), 4.11 (dq, *J* = 10.8, 7.2 Hz, 1H), 3.40 – 3.24 (m, 1H), 2.55 (s, 3H), 2.40 – 2.25 (m, 1H), 1.25 (t, *J* = 7.1 Hz, 3H) ppm.

^{13}C NMR (101 MHz, Chloroform-*d*): δ = 197.8, 163.6 (t, *J* = 32.4 Hz), 136.19, 136.10, 135.0, 132.9, 131.2, 128.4, 128.3, 127.5, 127.1, 126.6, 115.0 (t, *J* = 250.5 Hz), 62.8, 43.0 (t, *J* = 3.8 Hz), 37.4 (t, *J* = 23.6 Hz), 19.4, 13.5 ppm.

^{19}F NMR (376 MHz, Chloroform-*d*): δ = -103.68 (ddd, *J* = 257.8, 17.8, 14.6 Hz), -105.38 (dt, *J* = 256.8, 17.9 Hz) ppm.

HRMS (ESI/QTOF): *m/z*: $[M + Na]^+$ Calcd. for $C_{20}H_{20}F_2NaO_3^+$ 369.1273; Found 369.1275.

IR (ATR): 1767, 1685, 1579, 1210, 1027, 970, 780 cm^{-1}.

$[\alpha]_D^{20}$ = +36.2 (c = 1, $CHCl_3$).

Chiral HPLC: (Chiralpak IC, 1 % *i*PrOH/hexane, 1.0 mL/min, 210 nm): tR (minor) 9.25 min, tR (major) 11.89 min, 97:3 *er.*

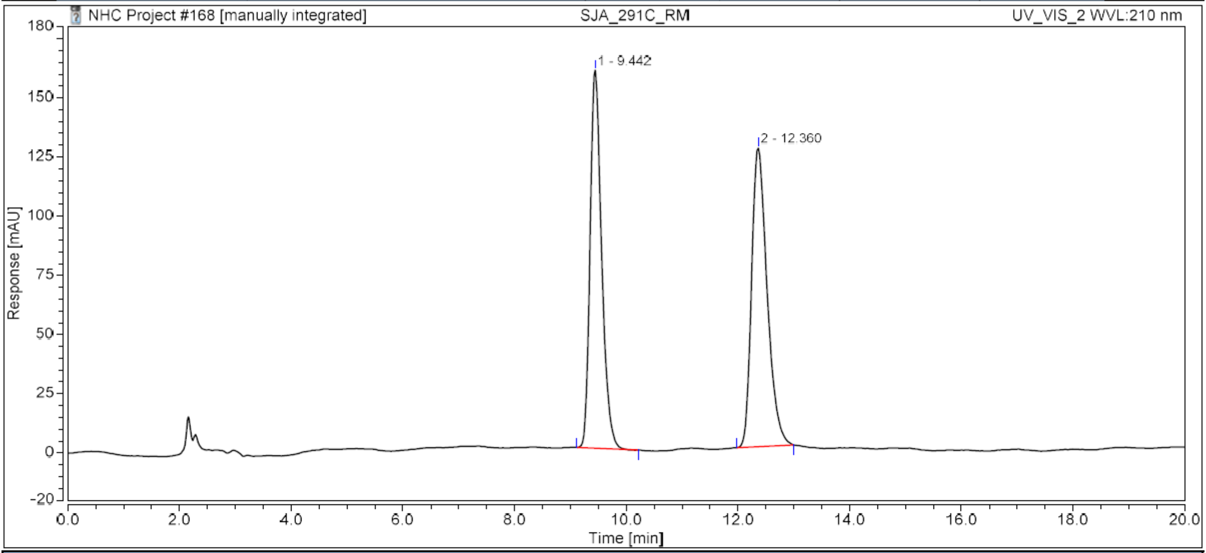

Integration Results							
No.	Peak Name	Retention Time min	Area mAU*min	Height mAU	Relative Area %	Relative Height %	Amount n.a.
1		9.442	38.147	159.664	48.40	55.84	n.a.
2		12.360	40.676	126.255	51.60	44.16	n.a.
**Total:**			**78.823**	**285.919**	**100.00**	**100.00**	

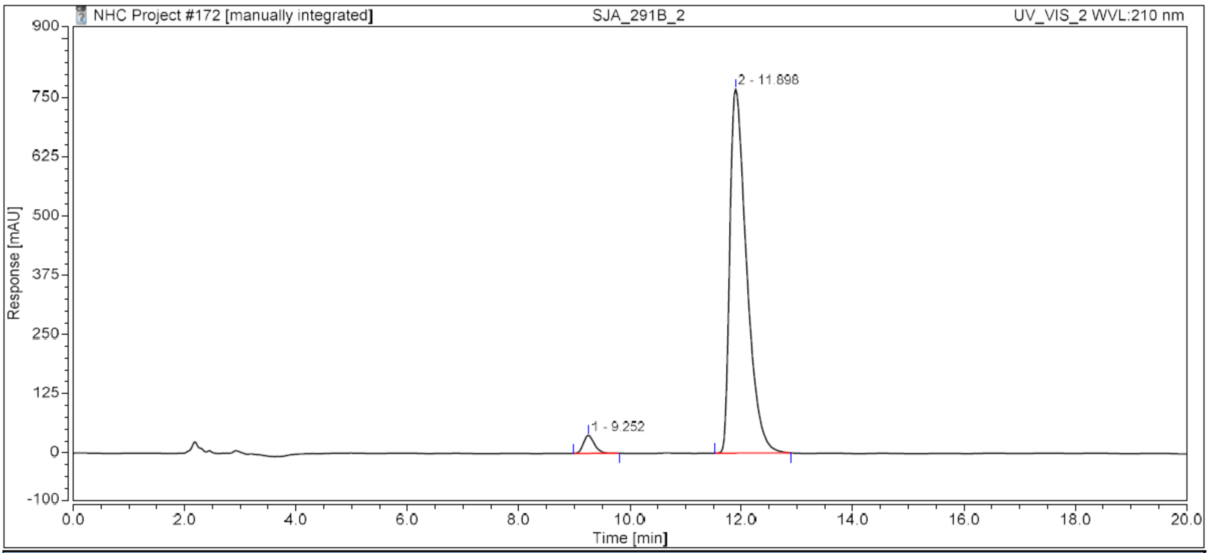

Integration Results

No.	Peak Name	Retention Time min	Area mAU*min	Height mAU	Relative Area %	Relative Height %	Amount n.a.
1		9.252	8.567	38.010	3.10	4.71	n.a.
2		11.898	267.466	769.496	96.90	95.29	n.a.
**Total:**			**276.034**	**807.506**	**100.00**	**100.00**	

Ethyl (*S*)-2,2-difluoro-4-(2-fluorophenyl)-5-oxo-5-phenylpentanoate (6g)

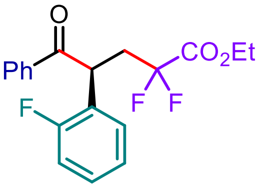

The title compound was synthesized according to the general procedure (**GP 9**), and was obtained after silica gel column chromatography (*n*-pentane : ethylacetate 20:1) as a colorless oil (63 % Yield, 22 mg, 96 % ee). **R$_f$** = 0.28 (*n*-pentane : ethylacetate 20:1).

^{1}H NMR (400 MHz, Chloroform-*d*): δ = 8.01 – 7.92 (m, 2H), 7.54 – 7.47 (m, 1H), 7.44 – 7.37 (m, 2H), 7.24 – 7.17 (m, 2H), 7.10 – 7.02 (m, 2H), 5.33 (dd, *J* = 7.6, 5.4 Hz, 1H), 4.21 (dq, *J* = 10.8, 7.2 Hz, 1H), 4.12 (dq, *J* = 10.8, 7.2 Hz, 1H), 3.33 – 3.18 (m, 1H), 2.60 – 2.45 (m, 1H), 1.25 (t, *J* = 7.1 Hz, 3H) ppm.

^{13}C NMR (101 MHz, Chloroform-*d*): δ = 197.1, 164.1 (t, *J* = 32.4 Hz), 160.1 (d, *J* = 246.5 Hz), 135.8, 133.9, 130.0 (d, *J* = 8.4 Hz), 129.4 (d, *J* = 3.3 Hz), 129.17, 129.10, 125.4 (d, *J* = 15.3 Hz), 125.2 (d, *J* = 3.5 Hz), 116.4 (d, *J* = 22.7 Hz), 115.6 (t, *J* = 251.0 Hz), 63.4, 38.9 (q, *J* = 3.8 Hz), 37.6 (t, *J* = 23.5 Hz), 14.2 ppm.

^{19}F NMR (376 MHz, Chloroform-*d*): δ = -104.64 (t, *J* = 16.4 Hz), -117.45 (dt, *J* = 11.0, 6.6 Hz) ppm.

HRMS (ESI/QTOF): *m/z*: $[M + Na]^+$ Calcd. for $C_{19}H_{17}F_3NaO_3^+$ 373.1022; Found 373.1023.

IR (ATR): 1763, 1685, 1596, 1583, 1491, 1320, 1078, 849, 659, 470 cm^{-1}.

$[\alpha]_D^{20}$ = +41.2 (c = 0.6, $CHCl_3$).

Chiral HPLC: (Chiralpak IC, 0.5 % *i*PrOH/hexane, 1.0 mL/min, 210 nm): tR (minor) 18.05 min, tR (major) 20.55 min, 98:2 *er*.

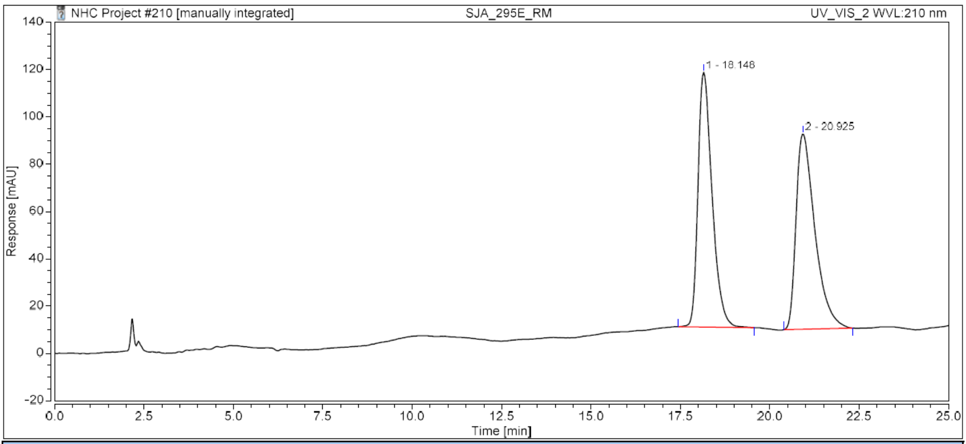

Integration Results							
No.	Peak Name	Retention Time min	Area mAU*min	Height mAU	Relative Area %	Relative Height %	Amount n.a.
1		18.148	49.272	107.728	49.46	56.61	n.a.
2		20.925	50.339	82.583	50.54	43.39	n.a.
Total:			99.611	190.311	100.00	100.00	

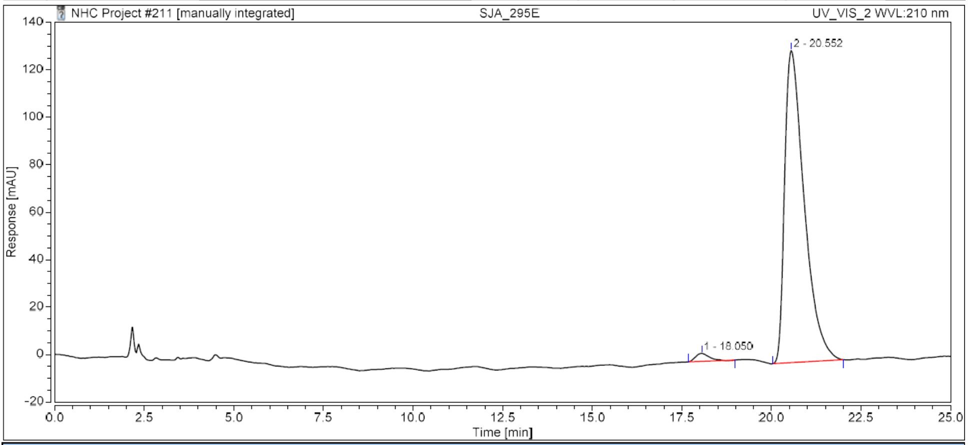

Integration Results							
No.	Peak Name	Retention Time min	Area mAU*min	Height mAU	Relative Area %	Relative Height %	Amount n.a.
1		18.050	1.444	3.371	1.71	2.50	n.a.
2		20.552	83.166	131.562	98.29	97.50	n.a.
Total:			84.610	134.933	100.00	100.00	

Ethyl (*S*)-2,2-difluoro-4-(naphthalen-2-yl)-5-oxo-5-phenylpentanoate (6h)

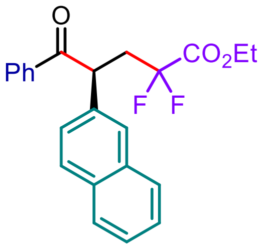

The title compound was synthesized according to the general procedure (**GP 9**), and was obtained after silica gel column chromatography (*n*-pentane : ethylacetate 20:1) as a colorless oil (73 % Yield, 28 mg, 98 % ee). **R**$_f$ = 0.22 (*n*-pentane : ethylacetate 20:1).

^{1}H NMR (400 MHz, Chloroform-*d*): δ = 8.01 – 7.97 (m, 2H), 7.81 – 7.71 (m, 4H), 7.49 – 7.41 (m, 4H), 7.40 – 7.34 (m, 2H), 5.12 (dd, *J* = 7.9, 5.0 Hz, 1H), 4.10 (dq, *J* = 10.7, 7.2 Hz, 1H), 3.98 (dq, *J* = 10.7, 7.1 Hz, 1H), 3.43 – 3.28 (m, 1H), 2.70 – 2.55 (m, 1H), 1.17 (t, *J* = 7.2 Hz, 3H) ppm.

^{13}C NMR (101 MHz, Chloroform-*d*): δ = 197.2, 163.8 (t, *J* = 32.5 Hz), 135.8, 135.2, 133.5, 133.2, 132.6, 129.2, 128.8, 128.6, 127.8, 127.6, 127.4, 126.5, 126.3, 125.8, 115.3 (t, *J* = 250.4 Hz), 62.9, 47.0 (t, *J* = 3.8 Hz), 38.2 (t, *J* = 23.3 Hz), 13.6 ppm.

^{19}F NMR (376 MHz, Chloroform-*d*): δ = -103.87 (dt, *J* = 259.5, 16.6 Hz), -104.67 (dt, *J* = 259.5, 16.65 Hz) ppm.

HRMS (ESI/QTOF): *m/z*: $[M + Na]^+$ Calcd. for $C_{23}H_{20}F_2NaO_3^+$ 405.1273; Found 405.1276.
IR (ATR): 1765, 1682, 1207, 1094, 689 cm^{-1}.
$[\alpha]_D^{20}$ = +57.2 (c = 0.5, $CHCl_3$).
Chiral HPLC: (Chiralpak IA, 1 % *i*PrOH/hexane, 1.0 mL/min, 210 nm): tR (major) 10.37 min, tR (minor) 13.53 min, 99:1 *er.*

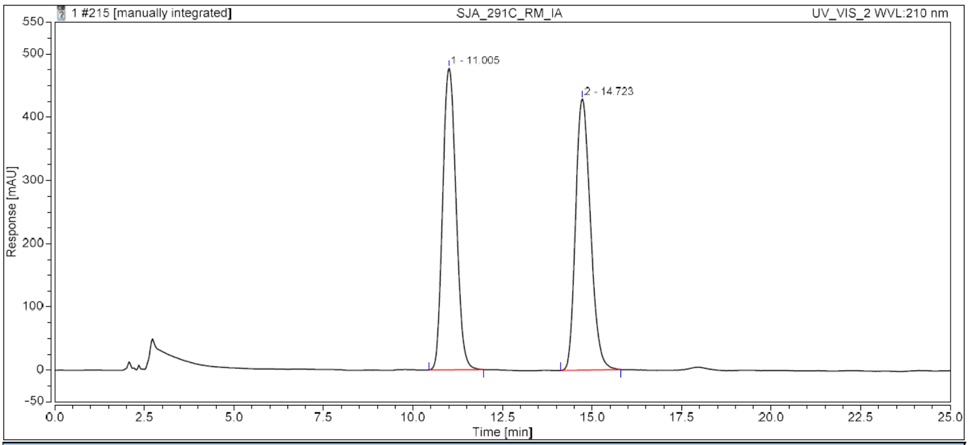

Integration Results

No.	Peak Name	Retention Time min	Area mAU*min	Height mAU	Relative Area %	Relative Height %	Amount n.a.
1		11.005	206.294	477.375	49.95	52.66	n.a.
2		14.723	206.748	429.102	50.05	47.34	n.a.
**Total:**			**413.042**	**906.477**	**100.00**	**100.00**	

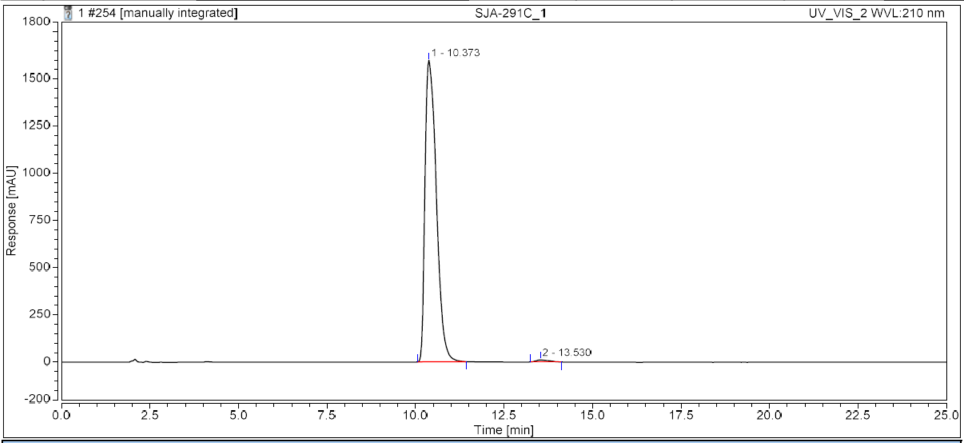

Integration Results

No.	Peak Name	Retention Time min	Area mAU*min	Height mAU	Relative Area %	Relative Height %	Amount n.a.
1		10.373	591.809	1596.991	99.25	99.35	n.a.
2		13.530	4.491	10.529	0.75	0.65	n.a.
**Total:**			**596.300**	**1607.520**	**100.00**	**100.00**	

Ethyl (S)-2,2-difluoro-4-(2-ferrocenyl)-5-oxo-5-phenylpentanoate (6i)

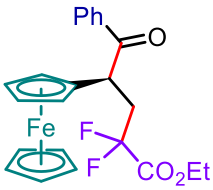

The title compound was synthesized according to the general procedure (**GP 9**), and was obtained after silica gel column chromatography (*n*-pentane : ethylacetate 20:1) as a yellow (61 % Yield, 27 mg, 95 % ee). $\mathbf{R_f}$ = 0.24 (*n*-pentane : ethylacetate 20:1).
^{1}**H NMR** (400 MHz, Chloroform-*d*): δ = 8.06 – 8.01 (m, 2H), 7.62 – 7.57 (m, 1H), 7.53 – 7.48 (m, 2H), 4.69 (dd, *J* = 10.2, 2.4 Hz, 1H), 4.29 – 4.14 (m, 2H), 4.12 – 4.08 (m, 3H), 4.02 (s, 5H), 3.99 (q, *J* = 1.7 Hz, 1H), 3.36 – 3.18 (m, 1H), 2.77 – 2.61 (m, 1H), 1.26 (t, *J* = 7.1 Hz, 3H) ppm.

^{13}C NMR (101 MHz, Chloroform-*d*): δ = 197.7, 163.9 (t, *J* = 32.5 Hz), 136.9, 133.45, 128.89, 128.86, 115.4 (t, *J* = 250.0 Hz), 86.5, 68.9, 68.18, 68.14, 67.8, 67.0, 63.1, 38.87, 38.83, 38.80 (t, *J* = 23.4 Hz), 13.9 ppm.
^{19}F NMR (376 MHz, Chloroform-*d*): δ = -103.95 (ddd, *J* = 259.4, 19.3, 15.3 Hz), -104.80 (ddd, *J* = 259.4, 19.3, 15.3 Hz) ppm.
HRMS (ESI/QTOF): *m/z*: [M + Na]$^+$ Calcd. for $C_{23}H_{22}F_2FeNaO_3^+$ 463.0779; Found 463.0778.
IR (ATR): 1765, 1684, 1093, 851, 531 cm^{-1}.
$[\alpha]_D^{20}$ = +32.2 (c = 0.6, $CHCl_3$).
Chiral HPLC: (Chiralpak IC, 3 % *i*PrOH/hexane, 1.0 mL/min, 210 nm): tR (minor) 10.15 min, tR (major) 12.97 min, 97.5:2.5 *er.*

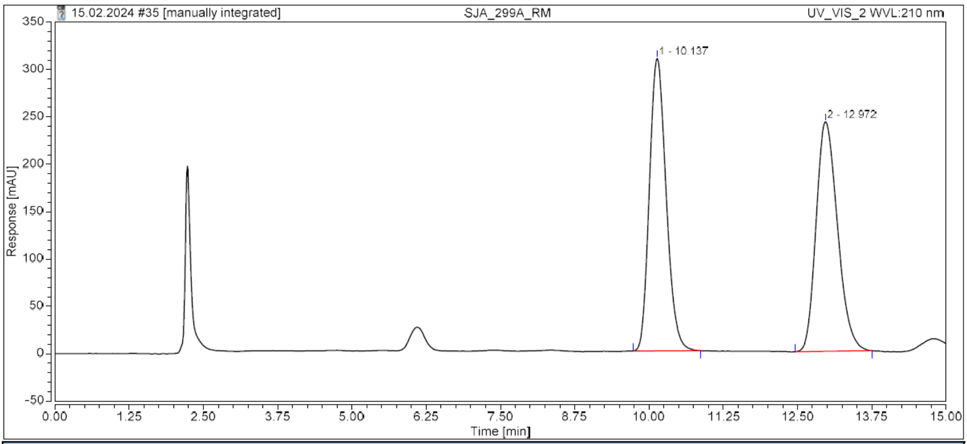

Integration Results							
No.	Peak Name	Retention Time min	Area mAU*min	Height mAU	Relative Area %	Relative Height %	Amount n.a.
1		10.137	100.909	308.837	49.90	56.03	n.a.
2		12.972	101.314	242.410	50.10	43.97	n.a.
**Total:**			**202.223**	**551.247**	**100.00**	**100.00**	

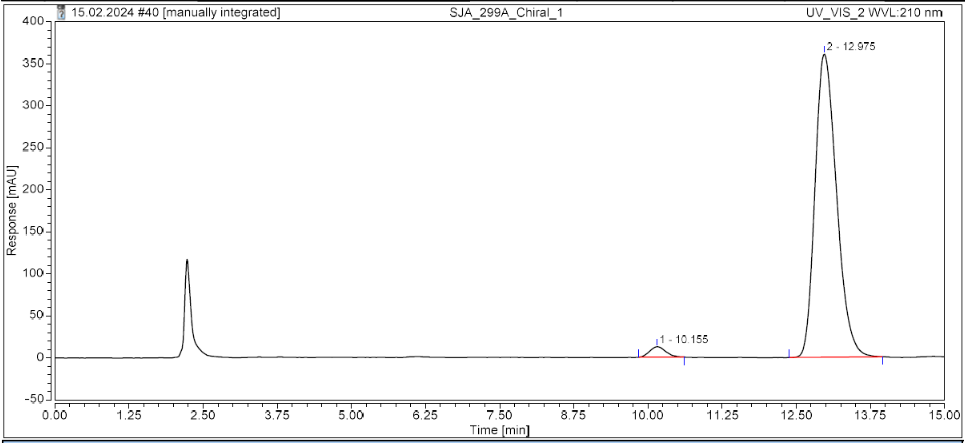

Integration Results							
No.	Peak Name	Retention Time min	Area mAU*min	Height mAU	Relative Area %	Relative Height %	Amount n.a.
1		10.155	3.881	12.395	2.49	3.32	n.a.
2		12.975	152.098	360.957	97.51	96.68	n.a.
**Total:**			**155.979**	**373.352**	**100.00**	**100.00**	

Ethyl (*R*)-2,2-difluoro-5-oxo-5-phenyl-4-(thiophen-2-yl)pentanoate (6j)

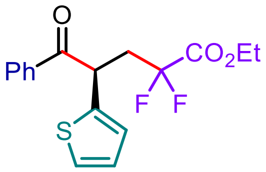

The title compound was synthesized according to the general procedure (**GP 9**), and was obtained after silica gel column chromatography (*n*-pentane : ethylacetate 20:1) as a pale orange oil (77 % Yield, 26 mg, 94 % ee). **R_f** = 0.22 (*n*-pentane : ethylacetate 20:1).

^{1}H NMR (400 MHz, Chloroform-*d*): δ = 8.02 – 7.97 (m, 2H), 7.57 – 7.51 (m, 1H), 7.48 – 7.40 (m, 2H), 7.19 (dd, *J* = 5.0, 1.4 Hz, 1H), 6.94 – 6.86 (m, 2H), 5.27 (dd, *J* = 8.4, 4.6 Hz, 1H), 4.22 (dq, *J* = 10.7, 7.2 Hz, 1H), 4.13 (dq, *J* = 10.8, 7.1 Hz, 1H), 3.38 – 3.22 (m, 1H), 2.70 – 2.55 (m, 1H), 1.25 (t, *J* = 7.2 Hz, 3H) ppm.
^{13}C NMR (101 MHz, Chloroform-*d*): δ = 196.1, 163.6 (t, *J* = 32.3 Hz), 139.7, 135.4, 133.5, 128.8, 128.7, 127.2, 126.5, 125.7, 114.9 (t, *J* = 251.0 Hz), 63.0, 41.3 (t, *J* = 4.2 Hz), 38.8 (t, *J* = 23.5 Hz), 13.7 ppm.
^{19}F NMR (376 MHz, Chloroform-*d*): δ = -104.17 (dt, *J* = 261.3, 16.6 Hz), -104.92 (dt, *J* = 261.3, 16.6 Hz) ppm.
HRMS (ESI/QTOF): *m/z*: [M + Na]$^+$ Calcd. for Calcd for $C_{17}H_{16}F_2NaO_3S^+$ 361.0680; Found 361.0688.
IR (ATR): 1766, 1686, 1279, 1093, 777, 689 cm^{-1}.
$[\alpha]_D^{20}$ = +32.2 (c = 0.6, $CHCl_3$).
Chiral HPLC: (Chiralpak IC, 3 % *i*PrOH/hexane, 1.0 mL/min, 210 nm): tR (minor) 6.67 min, tR (major) 8.20 min, 97:3 *er*.

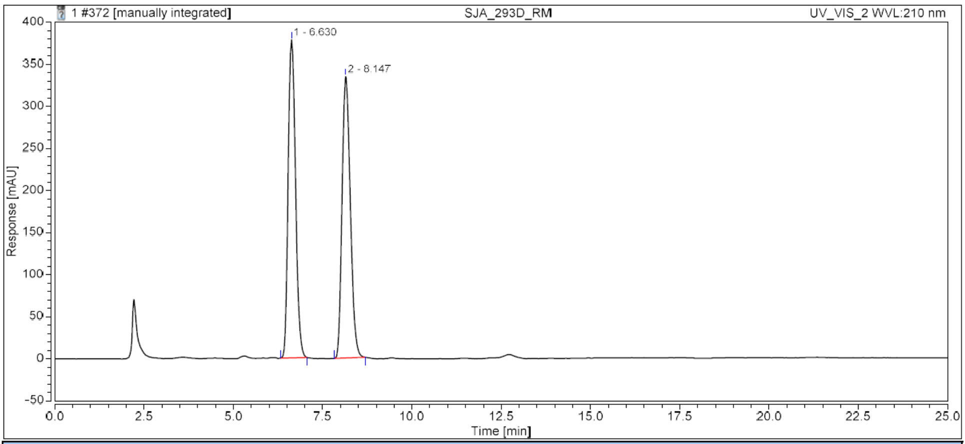

Integration Results

No.	Peak Name	Retention Time min	Area mAU*min	Height mAU	Relative Area %	Relative Height %	Amount n.a.
1		6.630	90.060	378.131	50.08	53.06	n.a.
2		8.147	89.782	334.553	49.92	46.94	n.a.
Total:			179.842	712.684	100.00	100.00	

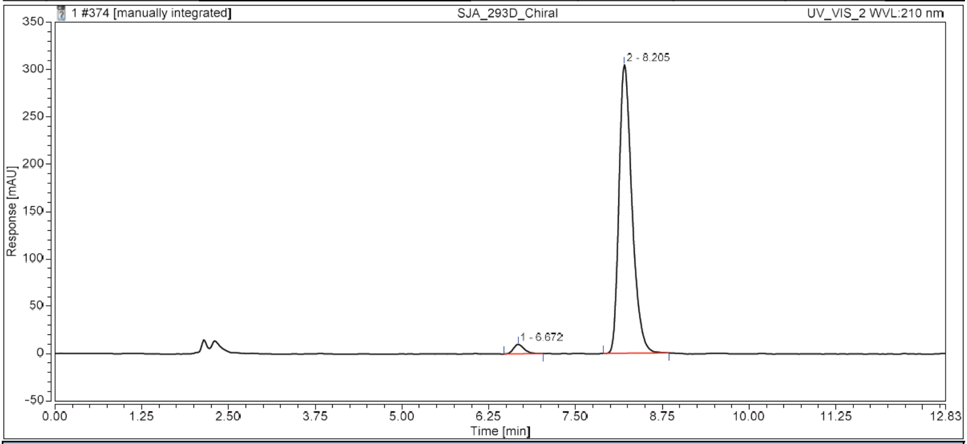

Integration Results

No.	Peak Name	Retention Time min	Area mAU*min	Height mAU	Relative Area %	Relative Height %	Amount n.a.
1		6.672	1.777	9.953	2.62	3.16	n.a.
2		8.205	66.065	304.948	97.38	96.84	n.a.
Total:			67.842	314.902	100.00	100.00	

Ethyl (*S*)-2,2-difluoro-5-oxo-5-phenyl-4-(pyridin-3-yl)pentanoate (6k)

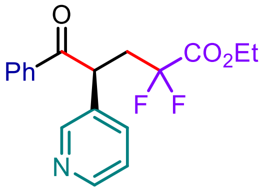

The title compound was synthesized according to the general procedure (**GP 9**), and was obtained after silica gel column chromatography (*n*-pentane : ethylacetate 4:1) as a colorless oil (60 % Yield, 20 mg, 82 % ee). **R_f** = 0.22 (*n*-pentane : ethylacetate 4:1).

^{1}H NMR (400 MHz, Chloroform-*d*): δ = 8.62 (d, *J* = 2.2 Hz, 1H), 8.48 (dd, *J* = 4.8, 1.6 Hz, 1H), 7.98 – 7.90 (m, 2H), 7.61 (dt, *J* = 8.0, 2.0 Hz, 1H), 7.56 – 7.50 (m, 1H), 7.45 – 7.39 (m, 2H), 7.22 (dd, *J* = 7.9, 4.8 Hz, 1H), 5.02 (dd, *J* = 7.8, 5.3 Hz, 1H), 4.24 – 4.07 (m, 2H), 3.27 (tdd, *J* = 16.5, 15.0, 7.7 Hz, 1H), 2.54 (tdd, *J* = 16.5, 15.0, 5.4 Hz, 1H), 1.25 (t, *J* = 7.1 Hz, 3H) ppm.

^{13}C NMR (101 MHz, Chloroform-*d*): δ = 197.1, 164.0 (t, *J* = 32.4 Hz), 150.3, 149.6, 135.79, 135.75, 134.14, 134.11, 129.3, 129.2, 124.4, 114.2 (d, *J* = 251.1 Hz), 63.5, 44.4 (t, *J* = 3.8 Hz), 38.3 (t, *J* = 23.3 Hz), 14.2 ppm.

^{19}F NMR (376 MHz, Chloroform-*d*): δ = -104.34 – -104.45 (m) ppm.

HRMS (ESI/QTOF): *m/z*: $[M + H]^+$ Calcd. for $C_{18}H_{18}F_2NO_3^+$ 334.1249; Found 334.1255.

IR (ATR): 1766, 1684, 1598, 1448, 1338, 1189, 707 cm^{-1}.

$[\alpha]_D^{20}$ = +27.2 (c = 1.0, $CHCl_3$).

Chiral HPLC: (Chiralpak IC, 7 % *i*PrOH/hexane, 1.0 mL/min, 210 nm): tR (minor) 19.98 min, tR (major) 22.45 min, 91:9 *er.*

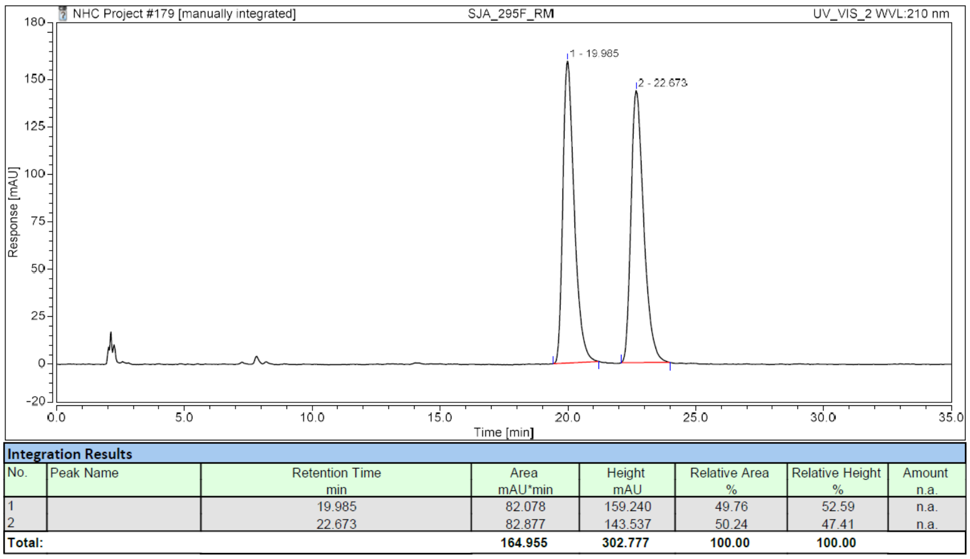

Integration Results

No.	Peak Name	Retention Time min	Area mAU*min	Height mAU	Relative Area %	Relative Height %	Amount n.a.
1		19.985	82.078	159.240	49.76	52.59	n.a.
2		22.673	82.877	143.537	50.24	47.41	n.a.
**Total:**			**164.955**	**302.777**	**100.00**	**100.00**	

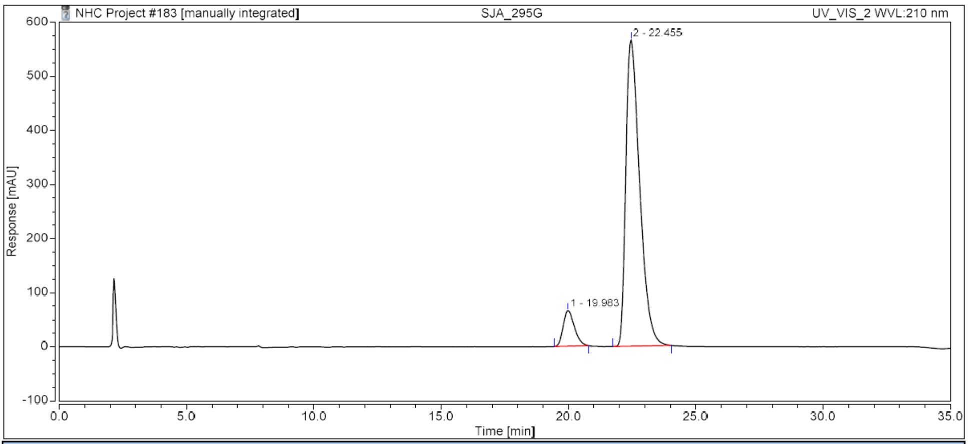

Integration Results							
No.	Peak Name	Retention Time min	Area mAU*min	Height mAU	Relative Area %	Relative Height %	Amount n.a.
1		19.983	33.684	65.851	8.60	10.41	n.a.
2		22.455	357.920	566.474	91.40	89.59	n.a.
Total:			391.604	632.324	100.00	100.00	

Ethyl (*S*)-2,2-difluoro-5-oxo-5-phenyl-4-(pyridin-4-yl)pentanoate (6l)

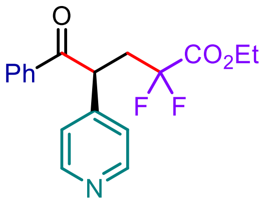

The title compound was synthesized according to the general procedure (**GP 9**), and was obtained after silica gel column chromatography (*n*-pentane : ethylacetate 4:1) as a colorless oil (63 % Yield, 21 mg, 94% ee). **R_f** = 0.20 (*n*-pentane : ethylacetate 4:1).

^{1}H NMR (400 MHz, Chloroform-*d*): δ = 8.56 – 8.52 (m, 2H), 7.96 – 7.91 (m, 2H), 7.58 – 7.51 (m, 1H), 7.47 – 7.40 (m, 2H), 7.25 – 7.23 (m, 2H), 4.97 (dd, *J* = 7.8, 5.0 Hz, 1H), 4.27 – 4.09 (m, 2H), 3.36 – 3.20 (m, 1H), 2.59 – 2.44 (m, 1H), 1.26 (t, *J* = 7.2 Hz, 3H) ppm.

^{13}C NMR (101 MHz, Chloroform-*d*): δ = 196.0, 163.3 (t, *J* = 32.4 Hz), 150.4, 146.5, 135.1, 133.6, 128.6, 128.6, 123.1, 114.7 (t, *J* = 251.4 Hz), 62.9, 45.8 (t, *J* = 3.7 Hz), 37.4 (t, *J* = 23.3 Hz), 13.5 ppm.

^{19}F NMR (376 MHz, Chloroform-*d*): δ = -103.53 – -105.30 (m) ppm.

HRMS (ESI/QTOF): *m/z*: $[M + H]^+$ Calcd. for $C_{18}H_{18}F_2NO_3^+$ 334.1249; Found 334.1252.

IR (ATR): 1766, 1686, 1596, 1448, 1338, 1277, 1192, 784 cm^{-1}.

$[\alpha]_D^{20}$ = +34.3 (c = 1.0, $CHCl_3$).

Chiral HPLC: (Chiralpak IC, 7 % *i*PrOH/hexane, 1.0 mL/min, 254 nm): tR (minor) 11.05 min, tR (major) 13.55 min, 97:3 *er.*

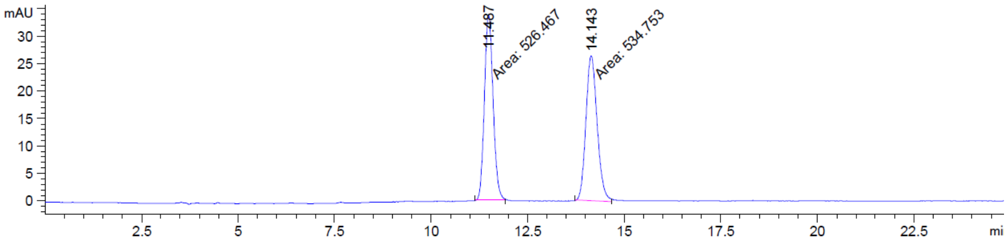

Peak #	RetTime [min]	Type	Width [min]	Area [mAU*s]	Height [mAU]	Area %
1	11.487	MM	0.2594	526.46747	33.82336	49.6096
2	14.143	MM	0.3371	534.75293	26.43519	50.3904

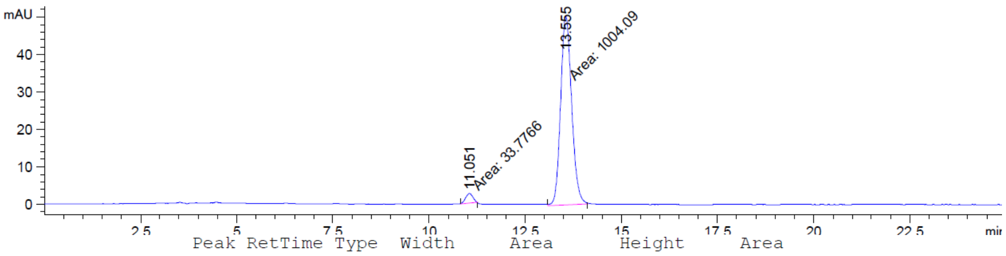

Peak #	RetTime [min]	Type	Width [min]	Area [mAU*s]	Height [mAU]	Area %
1	11.051	MM	0.2178	33.77656	2.58476	3.2544
2	13.555	MM	0.3317	1004.09265	50.45358	96.7456

Ethyl (*S*)-4-(1-benzyl-1*H*-indol-3-yl)-2,2-difluoro-5-oxo-5-phenylpentanoate (6m)

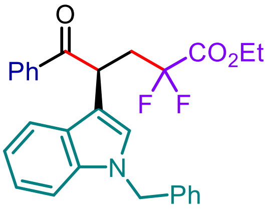

The title compound was synthesized according to the general procedure (**GP 9**), and was obtained after silica gel column chromatography (*n*-pentane : ethylacetate 9:1) as a colorless oil (59 % Yield, 27 mg, 96 % ee). $\mathbf{R_f}$ = 0.24 (*n*-pentane : ethylacetate 9:1).

^{1}H NMR (400 MHz, Chloroform-*d*): δ = 7.98 – 7.93 (m, 2H), 7.78 – 7.73 (m, 1H), 7.49 – 7.43 (m, 1H), 7.37 – 7.31 (m, 2H), 7.24 – 7.14 (m, 6H), 6.99 – 6.95 (m, 2H), 6.93 (s, 1H), 5.27 – 5.17 (m, 3H), 4.04 (dq, *J* = 10.7, 7.2 Hz, 1H), 3.89 (dq, *J* = 10.8, 7.2 Hz, 1H), 3.38 – 3.22 (m, 1H), 2.79 – 2.62 (m, 1H), 1.11 (t, *J* = 7.1 Hz, 3H) ppm.

^{13}C NMR (101 MHz, Chloroform-*d*): δ = 197.3, 163.9 (t, *J* = 32.5 Hz), 137.0, 136.8, 136.0, 132.9, 128.7, 128.6, 128.5, 127.6, 127.5, 126.6, 126.4, 122.4, 120.0, 118.9, 115.5 (t, *J* = 250.3 Hz), 111.4, 110.1, 62.7, 50.1, 38.2 (t, *J* = 4.3 Hz), 37.4 (t, *J* = 23.2 Hz), 13.6 ppm.

^{19}F NMR (376 MHz, Chloroform-*d*): δ = -103.9 (dt, *J* = 258.9, 16.4 Hz), -105.3 (dt, *J* = 259.4, 16.7 Hz) ppm.

HRMS (ESI/QTOF): *m/z*: $[M + Na]^+$ Calcd. for $C_{28}H_{25}F_2NNaO_3^+$ 484.1695; Found 484.1698.

IR (ATR): 1768, 1682, 1333, 1299, 1089, 974, 884 cm^{-1}.

$[\alpha]_D^{20}$ = +59.2 (c = 0.5, $CHCl_3$).

Chiral HPLC: (Chiralpak IC, 3 % *i*PrOH/hexane, 1.0 mL/min, 210 nm): tR (minor) 9.23 min, tR (major) 12.17 min, 98:2 *er.*

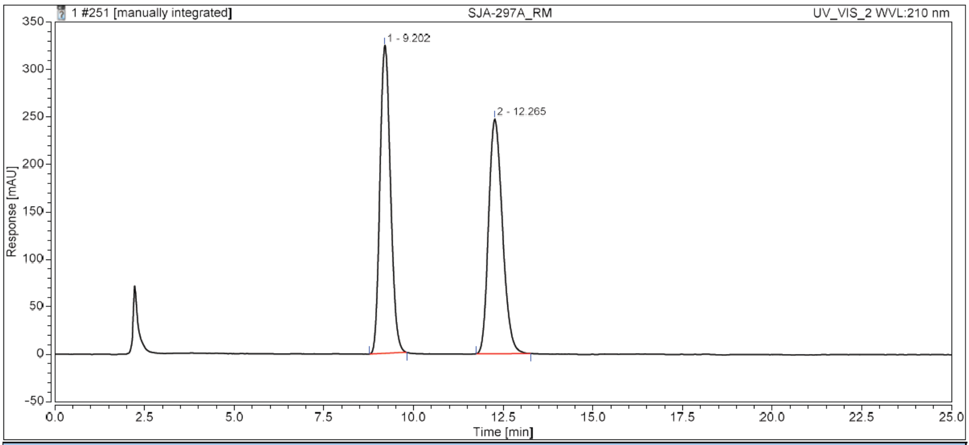

Integration Results

No.	Peak Name	Retention Time min	Area mAU*min	Height mAU	Relative Area %	Relative Height %	Amount n.a.
1		9.202	110.439	324.685	49.94	56.75	n.a.
2		12.265	110.689	247.441	50.06	43.25	n.a.
Total:			221.127	572.125	100.00	100.00	

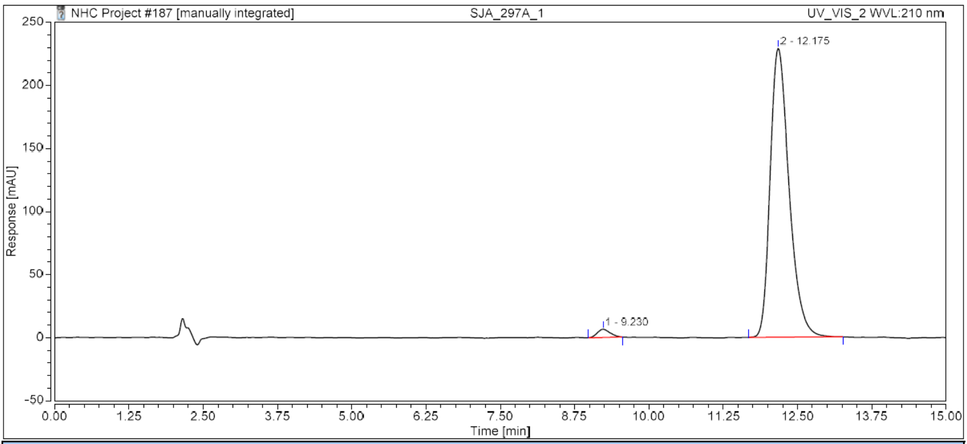

Integration Results

No.	Peak Name	Retention Time min	Area mAU*min	Height mAU	Relative Area %	Relative Height %	Amount n.a.
1		9.230	1.622	6.507	1.88	2.76	n.a.
2		12.175	84.493	229.003	98.12	97.24	n.a.
**Total:**			**86.115**	**235.510**	**100.00**	**100.00**	

Ethyl (*R*)-2,2-difluoro-5-oxo-4-phenoxy-5-phenylpentanoate (6n)

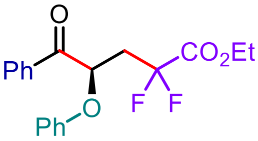

The title compound was synthesized according to the general procedure (**GP 9**), and was obtained after silica gel column chromatography (*n*-pentane : ethylacetate 20:1) as a colorless oil (66 % Yield, 23 mg, 94 % ee). $\mathbf{R_f}$ = 0.22 (*n*-pentane : ethylacetate 20:1).

^{1}**H NMR** (400 MHz, Chloroform-*d*): δ = 8.08 – 8.03 (m, 2H), 7.66 – 7.59 (m, 1H), 7.54 – 7.47 (m, 2H), 7.25 – 7.20 (m, 2H), 6.99 – 63.93 (m, 1H), 6.85 – 6.80 (m, 2H), 5.69 (ddd, *J* = 9.2, 3.3, 1.2 Hz, 1H), 4.37 – 4.21 (m, 2H), 3.00 – 2.84 (m, 1H), 2.83 – 2.70 (m, 1H), 1.27 (t, *J* = 7.1 Hz, 3H) ppm.

^{13}**C NMR** (101 MHz, Chloroform-*d*): δ = 195.6, 163.4 (t, *J* = 33.4 Hz), 156.7, 134.2, 133.6, 129.7, 129.0, 128.8, 122.2, 115.3, 114.5 (t, *J* = 250.3 Hz), 74.3 (dd, *J* = 7.2, 2.5 Hz), 63.2, 37.7 (t, *J* = 25.0 Hz), 13.8 ppm.

^{19}**F NMR** (376 MHz, Chloroform-*d*): δ = -101.73 (ddd, *J* = 260.8, 12.0, 10.3 Hz), -107.46 (ddd, *J* = 260.8, 22.9, 13.1 Hz) ppm.

HRMS (ESI/QTOF): *m/z*: [M + Na]$^+$ Calcd. for $C_{19}H_{18}F_2NaO_4^+$ 371.1065; Found 371.1068.

IR (ATR): 1769, 1701, 1596, 1494, 1236, 1130, 753 cm^{-1}.

$[\alpha]_D^{20}$ = +14.2 (c = 0.8, $CHCl_3$).

Chiral HPLC: (Chiralpak IC, 1 % *i*PrOH/hexane, 1.0 mL/min, 210 nm): tR (minor) 11.22 min, tR (major) 13.48 min, 97:3 *er.*

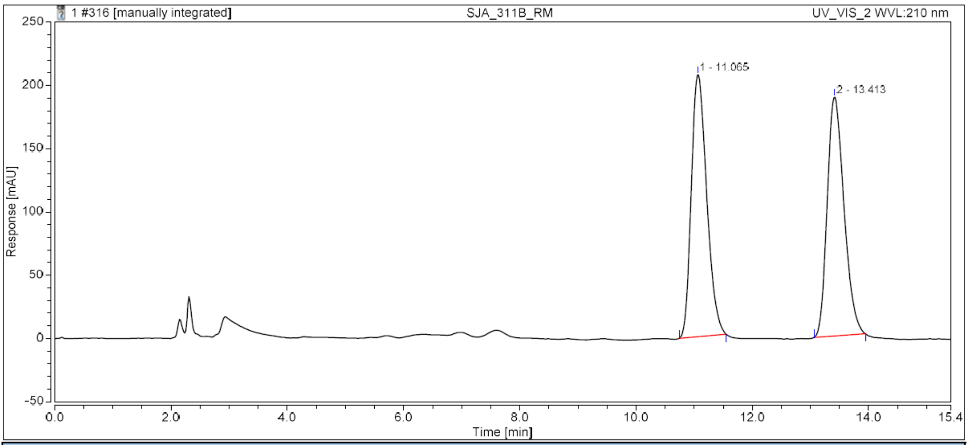

Integration Results							
No.	Peak Name	Retention Time min	Area mAU*min	Height mAU	Relative Area %	Relative Height %	Amount n.a.
1		11.065	63.541	207.366	50.20	52.34	n.a.
2		13.413	63.033	188.808	49.80	47.66	n.a.
Total:			126.575	396.174	100.00	100.00	

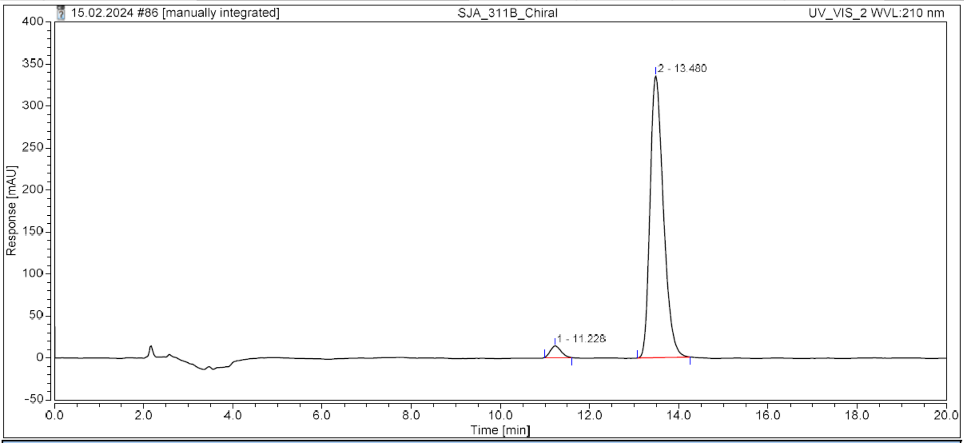

Integration Results							
No.	Peak Name	Retention Time min	Area mAU*min	Height mAU	Relative Area %	Relative Height %	Amount n.a.
1		11.228	3.811	13.765	3.17	3.94	n.a.
2		13.480	116.558	336.028	96.83	96.06	n.a.
Total:			120.369	349.793	100.00	100.00	

Ethyl (*R*)-2,2-difluoro-5-oxo-5-phenyl-4-(phenylthio)pentanoate (6o)

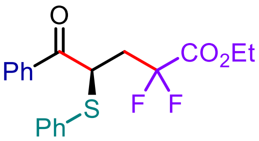

The title compound was synthesized according to the general procedure (**GP 9**), and was obtained after silica gel column chromatography (*n*-pentane : ethylacetate 20:1) as a colorless oil (65 % Yield, 24 mg, 95 % ee). **R**$_f$ = 0.24 (*n*-pentane : ethylacetate 20:1).

^{1}H NMR (400 MHz, Chloroform-*d*): δ = 7.97 – 7.89 (m, 2H), 7.63 – 7.54 (m, 1H), 7.51 – 7.41 (m, 2H), 7.40 – 7.25 (m, 5H), 4.77 (dd, *J* = 8.8, 4.1 Hz, 1H), 4.31 – 4.12 (m, 2H), 3.13 – 2.97 (m, 1H), 2.74 – 2.59 (m, 1H), 1.24 (t, *J* = 7.2 Hz, 3H) ppm.

^{13}C NMR (101 MHz, Chloroform-*d*): δ = 193.1, 163.8 (t, *J* = 32.2 Hz), 135.4, 135.1, 133.5, 130.4, 129.5, 129.3, 128.8, 114.9 (t, *J* = 251.5 Hz), 63.2, 44.1 (t, *J* = 3.9 Hz), 36.5 (t, *J* = 23.9 Hz), 13.8 ppm.

^{19}F NMR (376 MHz, Chloroform-*d*): δ = -103.66 (dt, *J* = 258.2, 16.6 Hz), -104.67 (dt, *J* = 258.2, 16.6 Hz) ppm.

HRMS (ESI/QTOF): *m/z*: [M + Na]$^+$ Calcd. for $C_{19}H_{18}F_2NaO_3S^+$ 387.0837; Found 387.0842.

IR (ATR): 1764, 1681, 1374, 1095, 1007, 688 cm^{-1}.

$[\alpha]_D^{20}$ = +19.2 (c = 0.6, $CHCl_3$).

Chiral HPLC: (Chiralpak IC, 1 % *i*PrOH/hexane, 1.0 mL/min, 210 nm): tR (minor) 12.05 min, tR (major) 15.58 min, 97.5:2.5 *er.*

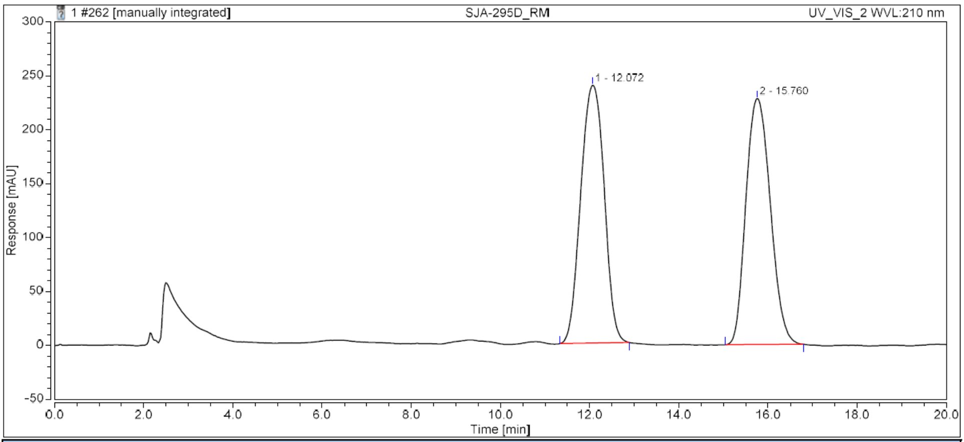

Integration Results

No.	Peak Name	Retention Time min	Area mAU*min	Height mAU	Relative Area %	Relative Height %	Amount n.a.
1		12.072	145.944	239.169	50.16	51.16	n.a.
2		15.760	145.013	228.288	49.84	48.84	n.a.
**Total:**			**290.958**	**467.457**	**100.00**	**100.00**	

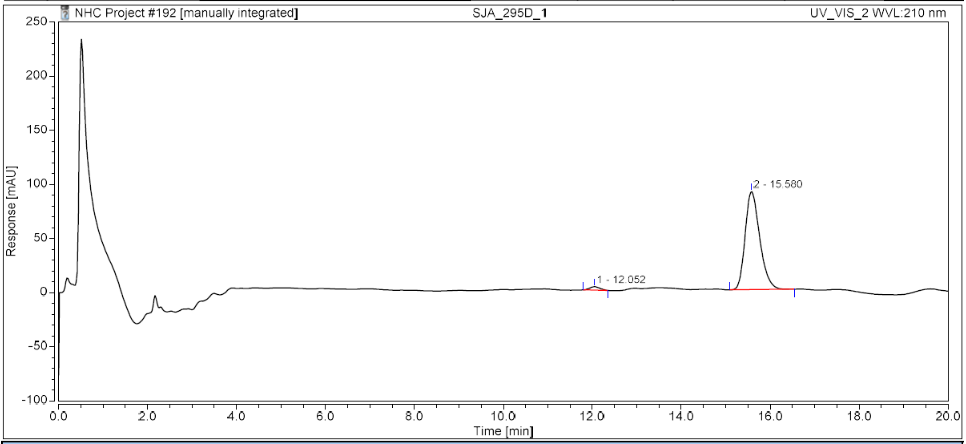

Integration Results

No.	Peak Name	Retention Time min	Area mAU*min	Height mAU	Relative Area %	Relative Height %	Amount n.a.
1		12.052	0.827	3.087	2.35	3.30	n.a.
2		15.580	34.445	90.544	97.65	96.70	n.a.
**Total:**			**35.272**	**93.631**	**100.00**	**100.00**	

Ethyl (*R*)-4-(1,3-dioxoisoindolin-2-yl)-2,2-difluoro-5-oxo-5-phenylpentanoate (6p)

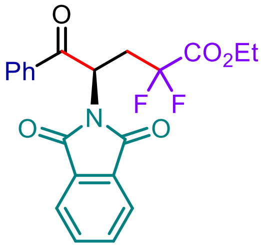

The title compound was synthesized according to the general procedure (**GP 9**), and was obtained after silica gel column chromatography (*n*-pentane : ethylacetate 4:1) as a yellow oil (52 % Yield, 21 mg, 94 % ee). $\mathbf{R_f}$ = 0.22 (*n*-pentane : ethylacetate 4:1).

^{1}H NMR (400 MHz, Chloroform-*d*): δ = 7.84 – 7.78 (m, 4H), 7.73 – 7.67 (m, 2H), 7.53 – 7.47 (m, 1H), 7.42 – 7.37 (m, 2H), 5.94 (dd, *J* = 9.8, 3.4 Hz, 1H), 4.33 – 4.20 (m, 2H), 3.26 – 3.00 (m, 2H), 1.32 (t, *J* = 7.1 Hz, 3H) ppm.

^{13}C NMR (101 MHz, Chloroform-*d*): δ = 193.9, 167.0, 163.3 (t, *J* = 32.2 Hz), 134.4, 133.5, 131.5, 128.8, 128.1, 123.7, 115.1 (t, *J* = 251.6 Hz), 63.3, 49.0 (t, *J* = 3.4 Hz), 32.4 (t, *J* = 22.9 Hz), 13.8 ppm.

^{19}**F NMR** (376 MHz, Chloroform-*d*): δ = -104.64 (ddd, *J* = 267.8, 20.2, 12.5 Hz), -105.73 (ddd, *J* = 267.8, 19.4, 15.3 Hz) ppm.
HRMS (ESI/QTOF) m/z: $[M + Na]^+$ Calcd for $C_{21}H_{17}F_2NNaO_5^+$ 424.0967; Found 424.0974
IR (ATR): 1767, 1716, 1381, 719 cm^{-1}.
$[\alpha]_D^{20}$ = +24.2 (c = 0.5, $CHCl_3$).
Chiral HPLC: (Chiralpak IC, 15 % *i*PrOH/hexane, 1.0 mL/min, 210 nm): tR (major) 7.43 min, tR (minor) 12.39 min, 97:3 *er.*

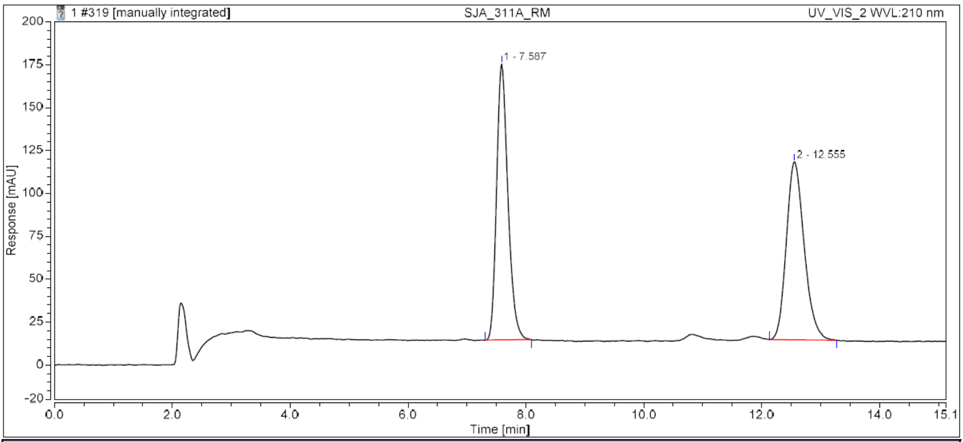

Integration Results

No.	Peak Name	Retention Time min	Area mAU*min	Height mAU	Relative Area %	Relative Height %	Amount n.a.
1		7.587	35.935	161.044	49.68	60.80	n.a.
2		12.555	36.399	103.820	50.32	39.20	n.a.
Total:			72.334	264.865	100.00	100.00	

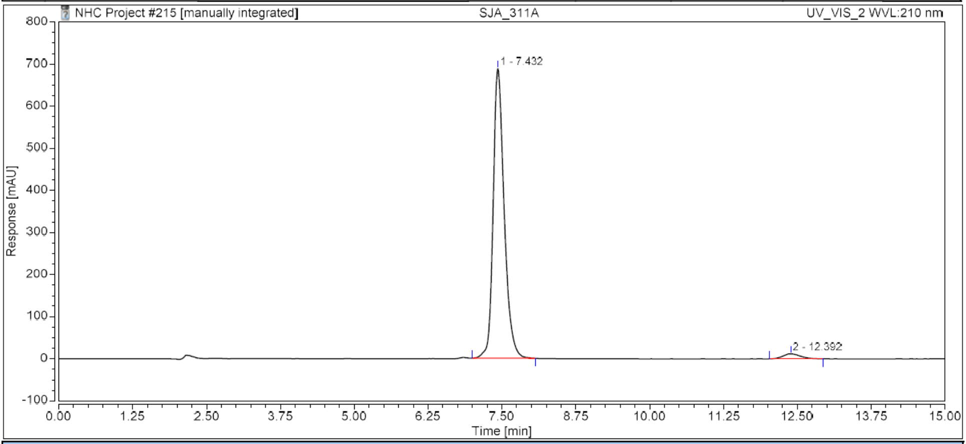

Integration Results

No.	Peak Name	Retention Time min	Area mAU*min	Height mAU	Relative Area %	Relative Height %	Amount n.a.
1		7.432	148.923	688.172	97.35	98.29	n.a.
2		12.392	4.058	11.952	2.65	1.71	n.a.
Total:			152.980	700.124	100.00	100.00	

Ethyl (*R*)-4-acetoxy-2,2-difluoro-4-methyl-5-oxo-5-phenylpentanoate (6q)

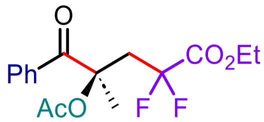

The title compound was synthesized according to the general procedure (**GP 9**), and was obtained after silica gel column chromatography (*n*-pentane : ethylacetate 9:1) as a colorless oil (49 % Yield, 16 mg, 93 % ee). $\mathbf{R_f}$ = 0.20 (*n*-pentane : ethylacetate 9:1).
^{1}**H NMR** (400 MHz, Chloroform-*d*): δ = 8.06 – 7.99 (m, 2H), 7.60 – 7.50 (m, 1H), 7.47 – 7.42 (m, 2H), 4.37 (q, *J* = 7.1 Hz, 2H), 3.36 – 3.21 (m, 1H), 2.99 – 2.85 (m, 1H), 1.96 (s, 3H), 1.89 (s, 3H), 1.41 (t, *J* = 7.2 Hz, 3H) ppm.
^{13}**C NMR** (101 MHz, Chloroform-*d*): δ = 197.3, 170.0, 163.8 (t, *J* = 32.2 Hz), 133.4, 132.9, 128.5, 115.4 (t, *J* = 254.0 Hz), 83.01 (d, *J* = 3.2 Hz), 63.17, 38.5 (t, *J* = 22.2 Hz), 23.49 (t, *J* = 2.5 Hz), 21.20, 13.95 ppm.

^{19}F NMR (376 MHz, Chloroform-*d*): δ = -100.51 (ddd, *J* = 263.9, 20.9, 10.8 Hz), -103.91 (ddd, *J* = 263.8, 21.3, 16.7 Hz) ppm.
HRMS (ESI/QTOF): *m/z*: [M + Na]$^+$ Calcd. for $C_{16}H_{18}F_2NaO_5^+$ 351.1015; Found 351.1027.
IR (ATR): 2924, 1744, 1687, 1139, 1062, 712 cm^{-1}.
$[\alpha]_D^{20}$ = +17.2 (c = 1, $CHCl_3$).
Chiral HPLC: (Chiralpak IC, 3 % *i*PrOH/hexane, 1.0 mL/min, 210 nm): tR (minor) 11.92 min, tR (major) 13.45 min, 96.5:3.5 *er.*

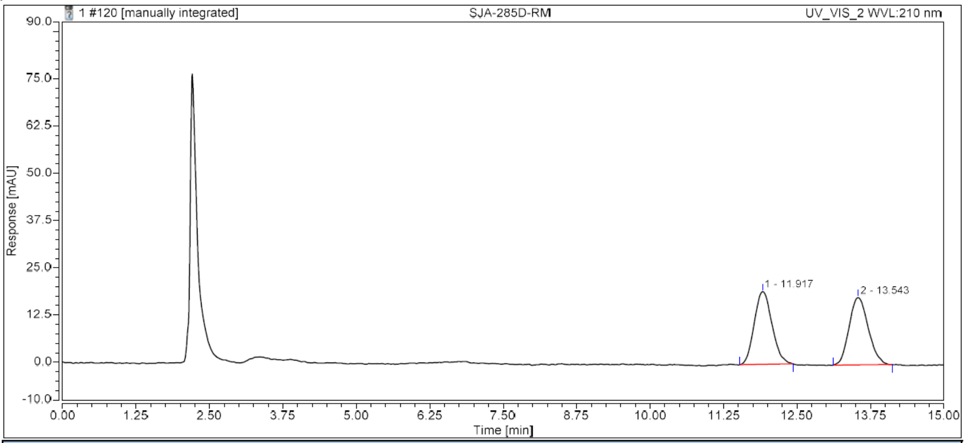

Integration Results

No.	Peak Name	Retention Time min	Area mAU*min	Height mAU	Relative Area %	Relative Height %	Amount n.a.
1		11.917	6.749	19.276	50.08	52.00	n.a.
2		13.543	6.727	17.790	49.92	48.00	n.a.
**Total:**			**13.475**	**37.066**	**100.00**	**100.00**	

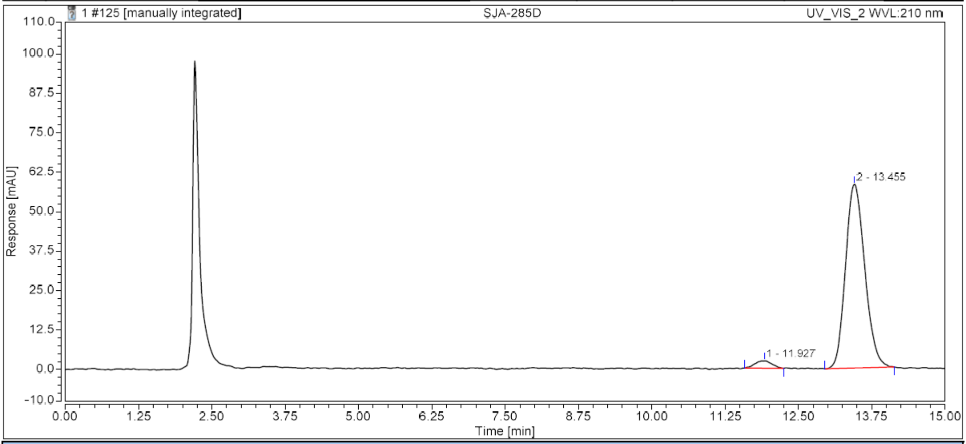

Integration Results

No.	Peak Name	Retention Time min	Area mAU*min	Height mAU	Relative Area %	Relative Height %	Amount n.a.
1		11.927	0.795	2.360	3.46	3.89	n.a.
2		13.455	22.173	58.270	96.54	96.11	n.a.
**Total:**			**22.968**	**60.630**	**100.00**	**100.00**	

Ethyl (*R*)-2,2-difluoro-5-oxo-5-phenyl-4-((trimethylsilyl)methyl)pentanoate (6r)

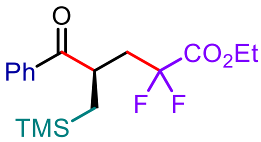

The title compound was synthesized according to the general procedure (**GP 9**), and was obtained after silica gel column chromatography (*n*-pentane : ethylacetate 30:1) as a colorless oil (19 % Yield, 6.5 mg, 76 % ee). **R$_f$** = 0.40 (*n*-pentane : ethylacetate 30:1).
^{1}H NMR (400 MHz, Chloroform-*d*): δ = 7.97 – 7.92 (m, 2H), 7.60 – 7.55 (m, 1H), 7.52 – 7.45 (m, 2H), 4.31 – 4.11 (m, 2H), 3.88 – 3.79 (m, 1H), 3.04 – 2.86 (m, 1H), 2.25 – 2.10 (m, 1H), 1.24 (t, *J* = 7.1 Hz, 3H), 0.97 (dd, *J* = 14.9, 4.7 Hz, 1H), 0.75 (dd, *J* = 14.9, 9.4 Hz, 1H), 0.05 (s, 9H) ppm.

^{13}C NMR (101 MHz, Chloroform-*d*): δ = 202.5, 164.9 (t, *J* = 32.7 Hz), 136.6, 134.1, 129.6, 129.4, 116.3 (t, *J* = 250.7 Hz), 63.8, 38.3 (t, *J* = 23.0 Hz), 36.2 (t, *J* = 3.7 Hz), 22.3, 14.6, 0.0 ppm.
^{19}F NMR (376 MHz, Chloroform-*d*): δ = -103.38 (ddd, *J* = 258.0, 19.3, 12.7 Hz), -105.45 (ddd, *J* = 258.2, 20.5, 16.5 Hz) ppm.
HRMS (ESI/QTOF): *m/z*: [M + Na]$^+$ Calcd. for $C_{17}H_{24}F_2NaO_3Si^+$ 365.1355; Found 365.1358.
IR (ATR): 2954, 2924, 2873, 1767, 1726, 1685, 1081, 842, 797 cm^{-1}.
$[\alpha]_D^{20}$ = +41.2 (c = 1.0, $CHCl_3$).
Chiral HPLC: (Chiralpak IC, 0.1 % *i*PrOH/hexane, 1.0 mL/min, 210 nm): tR (minor) 7.84 min, tR (major) 8.43 min, 88:12 *er.*

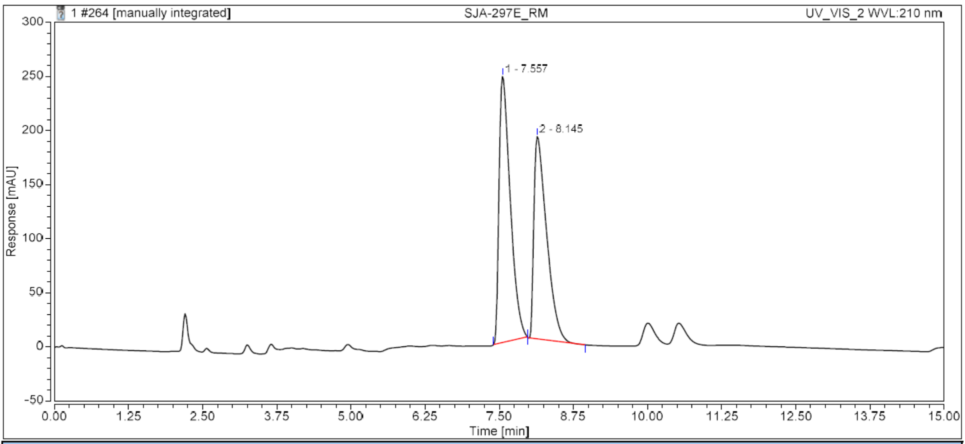

Integration Results

No.	Peak Name	Retention Time min	Area mAU*min	Height mAU	Relative Area %	Relative Height %	Amount n.a.
1		7.557	53.581	246.233	53.59	56.79	n.a.
2		8.145	46.398	187.328	46.41	43.21	n.a.
**Total:**			**99.979**	**433.561**	**100.00**	**100.00**	

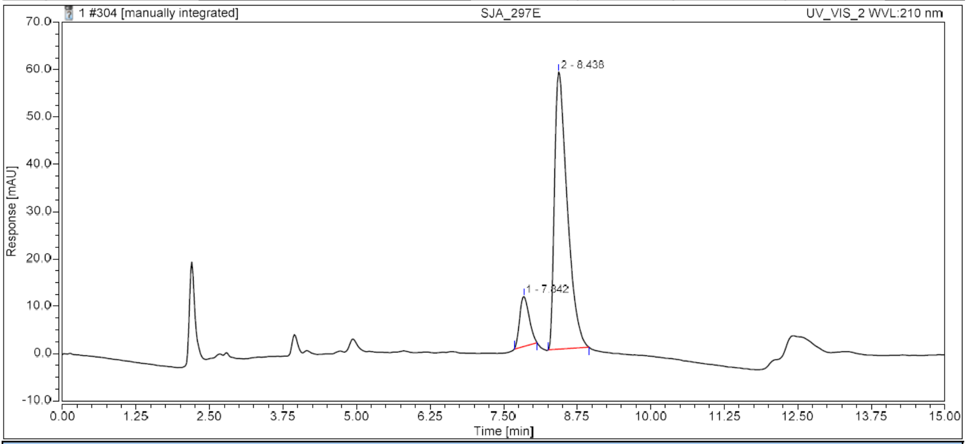

Integration Results

No.	Peak Name	Retention Time min	Area mAU*min	Height mAU	Relative Area %	Relative Height %	Amount n.a.
1		7.842	1.970	10.563	11.79	15.28	n.a.
2		8.438	14.735	58.562	88.21	84.72	n.a.
**Total:**			**16.706**	**69.125**	**100.00**	**100.00**	

Ethyl (*R*)-4-benzoyl-2,2-difluoro-6,6-dimethylheptanoate (6s)

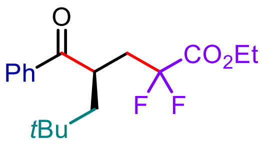

The title compound was synthesized according to the general procedure (**GP 9**), and was obtained after silica gel column chromatography (*n*-pentane : ethylacetate 30:1) as a colorless oil (23 % Yield, 7.5 mg, 90 % ee). **R$_f$** = 0.38 (*n*-pentane : ethylacetate 30:1).

^{1}H NMR (400 MHz, Chloroform-*d*): δ = 8.04 – 7.95 (m, 2H), 7.61 – 7.55 (m, 1H), 7.51 – 7.46 (m, 2H), 4.31 – 4.17 (m, 2H), 3.92 – 3.84 (m, 1H), 2.80 – 2.63 (m, 1H), 2.35 – 2.19 (m, 1H), 1.87 (dd, *J* = 14.2, 6.7 Hz, 1H), 1.42 (dd, *J* = 14.3, 4.9 Hz, 1H), 1.28 (t, *J* = 7.2 Hz, 3H), 0.88 (s, 9H) ppm.
^{13}C NMR (101 MHz, Chloroform-*d*): δ = 202.5, 164.0 (t, *J* = 32.6 Hz), 136.3, 133.3, 128.9, 128.6, 115.6 (t, *J* = 251.0 Hz), 63.1, 46.4, 38.2 (t, *J* = 22.6 Hz), 35.7 (t, *J* = 3.0 Hz), 31.5, 29.9, 13.9 ppm.
^{19}F NMR (376 MHz, Chloroform-*d*): δ = -104.35 (t, *J* = 17.4 Hz) ppm.
HRMS (ESI/QTOF): *m/z*: $[M + H]^+$ Calcd. for $C_{18}H_{25}F_2O_3^+$ 327.1766; Found 327.1756.
IR (ATR): 1768, 1683, 1293, 1090, 870 cm^{-1}.
$[\alpha]_D^{20}$ = +33.4 (c = 0.8, $CHCl_3$).
Chiral HPLC: (Chiralpak IC, 0.5 % *i*PrOH/hexane, 1.0 mL/min, 210 nm): tR (minor) 12.65 min, tR (major) 14.84 min, 95:5 *er.*

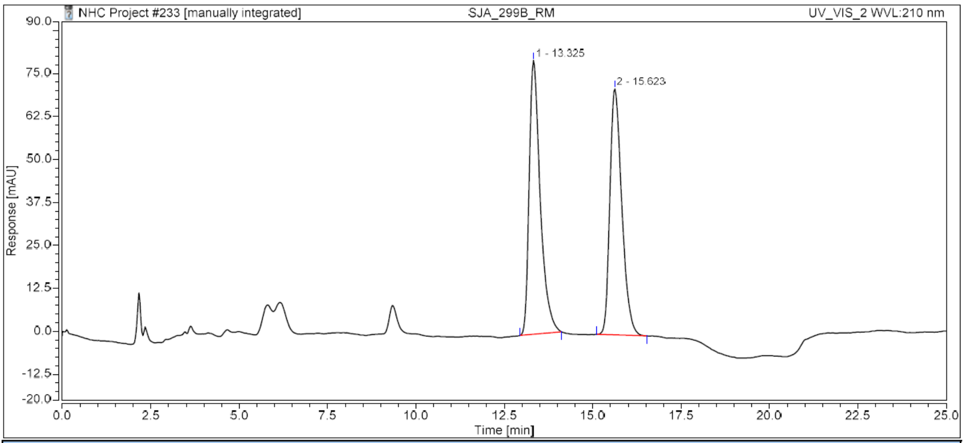

Integration Results

No.	Peak Name	Retention Time min	Area mAU*min	Height mAU	Relative Area %	Relative Height %	Amount n.a.
1		13.325	28.857	79.741	50.75	52.67	n.a.
2		15.623	28.007	71.654	49.25	47.33	n.a.
**Total:**			**56.864**	**151.395**	**100.00**	**100.00**	

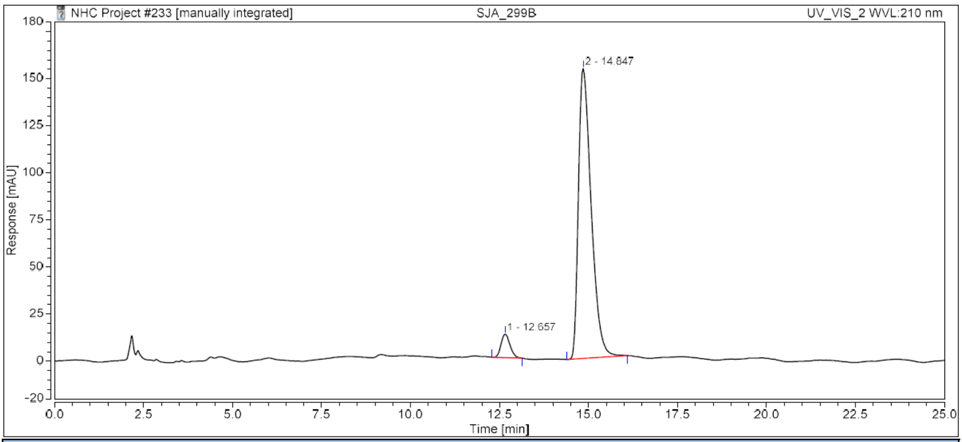

Integration Results

No.	Peak Name	Retention Time min	Area mAU*min	Height mAU	Relative Area %	Relative Height %	Amount n.a.
1		12.657	3.621	12.483	5.37	7.50	n.a.
2		14.847	63.862	153.892	94.63	92.50	n.a.
**Total:**			**67.483**	**166.375**	**100.00**	**100.00**	

Ethyl (*R*)-4-benzyl-2,2-difluoro-5-oxo-5-phenylpentanoate (6t)

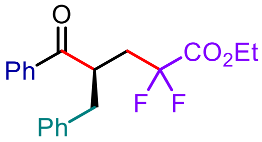

The title compound was synthesized according to the general procedure (**GP 9**), and was obtained after silica gel column chromatography (*n*-pentane : ethylacetate 20:1) as a colorless oil (26 % Yield, 9 mg, 92 % ee). **R$_f$** = 0.30 (*n*-pentane : ethylacetate 20:1).

^{1}H NMR (400 MHz, Chloroform-*d*): δ = 7.94 – 7.88 (m, 2H), 7.58 – 7.53 (m, 1H), 7.47 – 7.42 (m, 2H), 7.29 – 7.22 (m, 2H), 7.21 – 7.12 (m, 3H), 4.26 – 4.03 (m, 3H), 3.08 (dd, *J* = 13.8, 6.2 Hz, 1H), 2.96 – 2.78 (m, 1H), 2.71 (dd, *J* = 13.8, 8.4 Hz, 1H), 2.28 – 2.13 (m, 1H), 1.22 (t, *J* = 7.2 Hz, 3H) ppm.
^{13}C NMR (101 MHz, Chloroform-*d*): δ = 201.3, 164.4 (d, *J* = 109.8 Hz), 137.7, 136.2, 133.2, 129.0, 128.7, 128.6, 128.3, 126.8, 114.1 (d, *J* = 250.7 Hz), 62.9, 41.3, 39.1, 35.2 (t, *J* = 23.5 Hz), 13.7 ppm.
^{19}F NMR (376 MHz, Chloroform-*d*): δ = -103.85 (ddd, *J* = 260.0, 19.2, 12.7 Hz), -105.45 (ddd, *J* = 260.0, 20.5, 16.5 Hz) ppm.
HRMS (ESI/QTOF): *m/z*: $[M + H]^+$ Calcd. for $C_{20}H_{21}F_2O_3^+$: 347.1453; Found 347.1453.
IR (ATR): 2924, 1762, 1682, 1213, 1093, 698 cm^{-1}.
$[\alpha]_D^{20}$ = +29.4 (c = 1.0, $CHCl_3$).
Chiral HPLC: (Chiralpak IC, 1 % *i*PrOH/hexane, 1.0 mL/min, 210 nm): tR (minor) 13.47 min, tR (major) 15.39 min, 96:4 *er.*

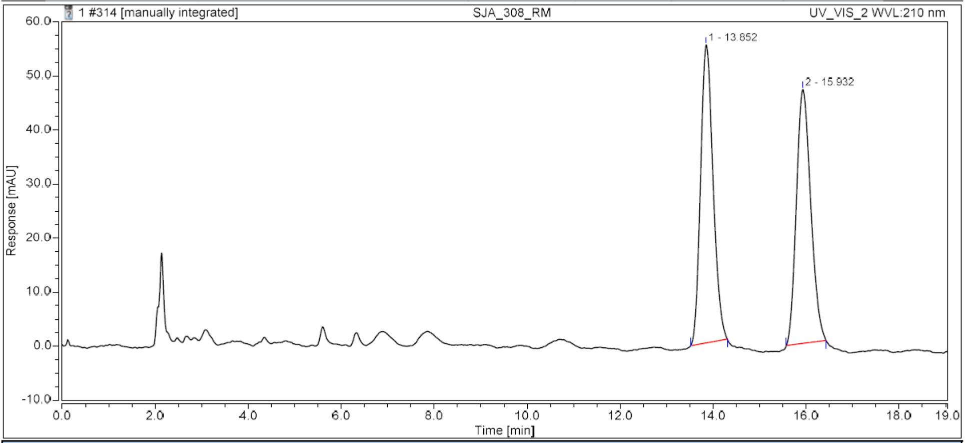

Integration Results

No.	Peak Name	Retention Time min	Area mAU*min	Height mAU	Relative Area %	Relative Height %	Amount n.a.
1		13.852	17.143	55.145	50.49	53.97	n.a.
2		15.932	16.809	47.029	49.51	46.03	n.a.
Total:			33.952	102.174	100.00	100.00	

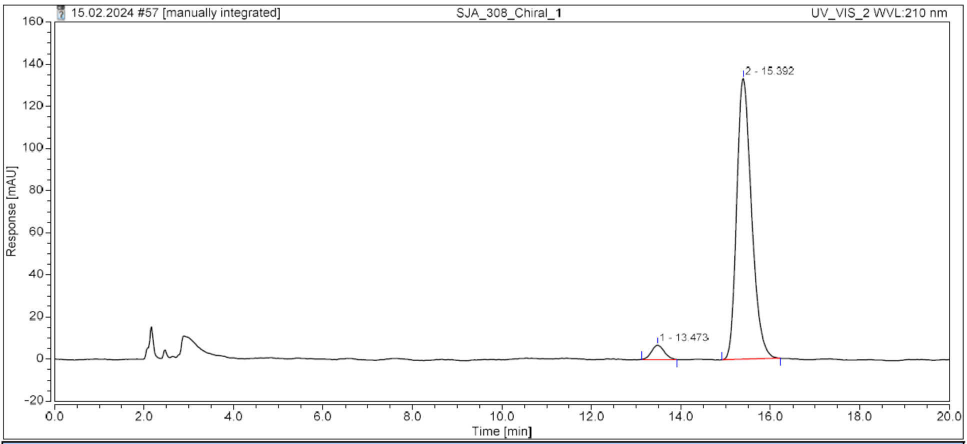

Integration Results

No.	Peak Name	Retention Time min	Area mAU*min	Height mAU	Relative Area %	Relative Height %	Amount n.a.
1		13.473	2.209	6.803	4.13	4.85	n.a.
2		15.392	51.301	133.510	95.87	95.15	n.a.
Total:			53.510	140.313	100.00	100.00	

tert-Pentyl (*S*)-2,2-difluoro-5-oxo-4,5-diphenylpentanoate (7a)

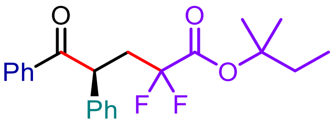

The title compound was synthesized according to the general procedure (**GP 9**), and was obtained after silica gel column chromatography (*n*-pentane : ethylacetate 20:1) as a colorless oil (84 % Yield, 31 mg, >99 % ee). **R_f** = 0.24 (*n*-pentane : ethylacetate 20:1).

^{1}H NMR (400 MHz, Chloroform-*d*): δ = 7.98 – 7.93 (m, 2H), 7.53 – 7.46 (m, 1H), 7.43 – 7.37 (m, 2H), 7.33 – 7.26 (m, 4H), 7.24 – 7.18 (m, 1H), 4.97 (dd, *J* = 8.2, 4.5 Hz, 1H), 3.38 – 3.21 (m, 1H), 2.53 – 2.38 (m, 1H), 1.84 – 1.70 (m, 2H), 1.43 (s, 3H), 1.41 (s, 3H), 0.88 (t, *J* = 7.5 Hz, 3H) ppm.
^{13}C NMR (101 MHz, Chloroform-*d*): δ = 197.4, 162.7 (t, *J* = 31.8 Hz), 138.2, 136.0, 133.1, 129.2, 128.8, 128.6, 128.2, 127.6, 115.2 (t, *J* = 251.4 Hz), 87.2, 46.6 (t, *J* = 3.5 Hz), 38.0 (t, *J* = 23.0 Hz), 33.3, 25.0, 8.0 ppm.
^{19}F NMR (376 MHz, Chloroform-*d*): δ = -104.28 (td, *J* = 16.8, 6.2 Hz) ppm.
HRMS (ESI/QTOF): *m/z*: [M + Na]$^+$ Calcd. for $C_{22}H_{24}F_2NaO_3^+$ 397.1586; Found 397.1579.
IR (ATR): 1759, 1685, 1448, 1295, 689 cm^{-1}.
$[\alpha]_D^{20}$ = +54.2 (c = 0.6, $CHCl_3$).
Chiral HPLC: (Chiralpak IC, 1 % *i*PrOH/hexane, 1.0 mL/min, 210 nm): tR (minor) 6.81 min, tR (major) 9.08 min, 99.8:0.2 *er*.

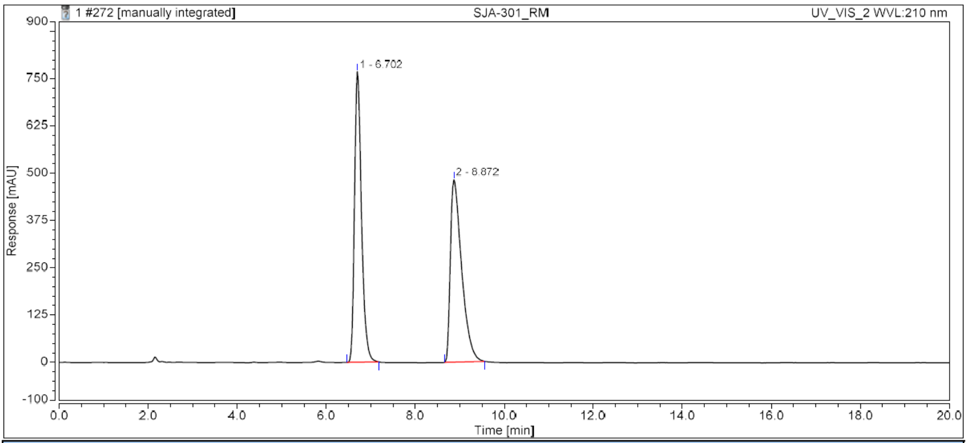

Integration Results							
No.	Peak Name	Retention Time min	Area mAU*min	Height mAU	Relative Area %	Relative Height %	Amount n.a.
1		6.702	139.209	768.319	49.63	61.40	n.a.
2		8.872	141.261	482.991	50.37	38.60	n.a.
Total:			280.470	1251.310	100.00	100.00	

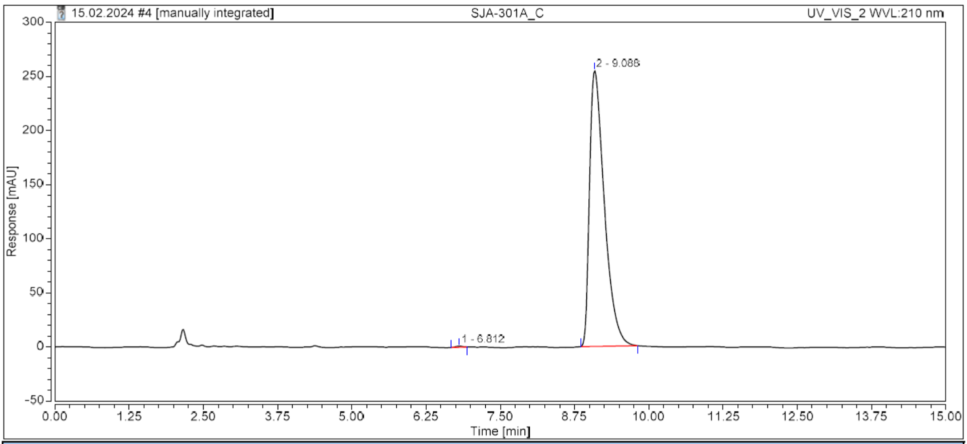

Integration Results							
No.	Peak Name	Retention Time min	Area mAU*min	Height mAU	Relative Area %	Relative Height %	Amount n.a.
1		6.812	0.132	0.965	0.18	0.38	n.a.
2		9.088	73.303	254.947	99.82	99.62	n.a.
Total:			73.435	255.911	100.00	100.00	

Cyclohexyl (*S*)-2,2-difluoro-5-oxo-4,5-diphenylpentanoate (7b)

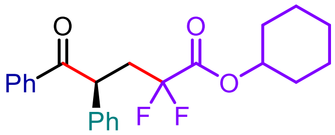

The title compound was synthesized according to the general procedure (**GP 9**), and was obtained after silica gel column chromatography (*n*-pentane : ethylacetate 20:1) as a colorless oil (76 % Yield, 29 mg,94% ee). **R**$\mathbf{R_f}$ = 0.26 (*n*-pentane : ethylacetate 20:1).
^{1}H NMR (400 MHz, Chloroform-*d*): δ = 7.98 – 7.92 (m, 2H), 7.52 – 7.46 (m, 1H), 7.42 – 7.36 (m, 2H), 7.32 – 7.26 (m, 4H), 7.25 – 7.18 (m, 1H), 4.97 (dd, *J* = 8.0, 4.8 Hz, 1H), 4.80 – 4.72 (m, 1H), 3.38 – 3.22 (m, 1H), 2.58 – 2.43 (m, 1H), 1.92 – 1.65 (m, 4H), 1.55 – 1.19 (m, 6H) ppm.
^{13}C NMR (101 MHz, Chloroform-*d*): δ = 197.5, 163.4 (t, *J* = 32.3 Hz), 138.1, 136.0, 133.3, 129.3, 128.9, 128.7, 128.3, 127.7, 115.4 (t, *J* = 251.0 Hz), 76.1, 46.9 (t, *J* = 3.7 Hz), 38.2 (t, *J* = 23.2 Hz), 31.1 (d, *J* = 3.5 Hz), 25.2, 23.5 ppm.
^{19}F NMR (376 MHz, Chloroform-*d*): δ = -104.44 (t, *J* = 16.7 Hz) ppm.
HRMS (ESI/QTOF): m/z: $[M + Na]^+$ Calcd. for $C_{23}H_{24}F_2NaO_3^+$ 409.1586; Found 409.1575.
IR (ATR): 2938, 1758, 1684, 1494, 1209, 1045, 698 cm^{-1}.
$[\alpha]_D^{20}$ = +32.6 (c = 0.8, $CHCl_3$).
Chiral HPLC: (Chiralpak IC, 1 % *i*PrOH/hexane, 1.0 mL/min, 210 nm): tR (minor) 9.35 min, tR (major) 11.52 min, 97:3 *er*.

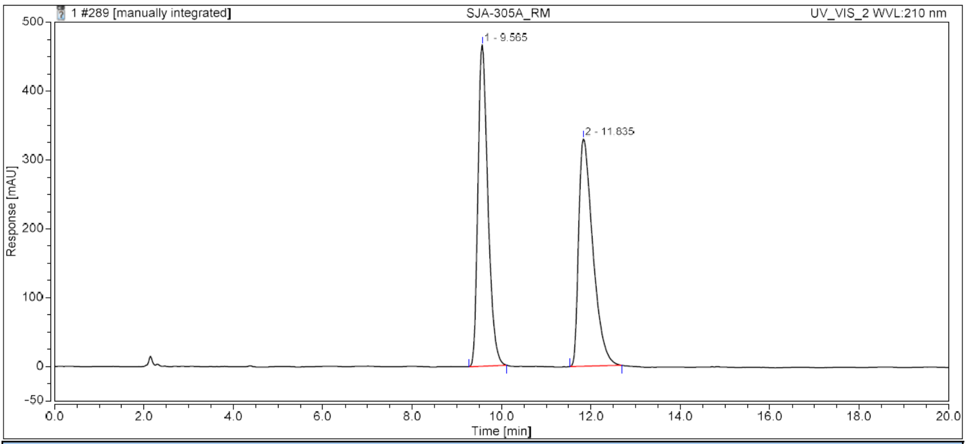

Integration Results

No.	Peak Name	Retention Time min	Area mAU*min	Height mAU	Relative Area %	Relative Height %	Amount n.a.
1		9.565	120.012	467.128	49.76	58.57	n.a.
2		11.835	121.171	330.388	50.24	41.43	n.a.
**Total:**			**241.183**	**797.516**	**100.00**	**100.00**	

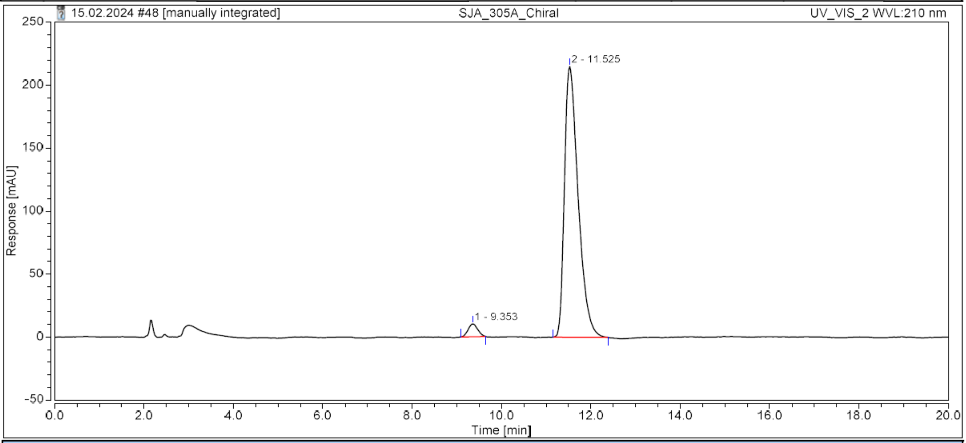

Integration Results

No.	Peak Name	Retention Time min	Area mAU*min	Height mAU	Relative Area %	Relative Height %	Amount n.a.
1		9.353	2.556	10.194	3.24	4.53	n.a.
2		11.525	76.307	215.063	96.76	95.47	n.a.
**Total:**			**78.864**	**225.257**	**100.00**	**100.00**	

Allyl (*S*)-2,2-difluoro-5-oxo-4,5-diphenylpentanoate (7c)

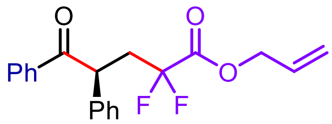

The title compound was synthesized according to the general procedure (**GP 9**), and was obtained after silica gel column chromatography (*n*-pentane : ethylacetate 20:1) as a colorless oil (65 % Yield, 22 mg, 94 % ee). $\mathbf{R_f}$ = 0.22 (*n*-pentane : ethylacetate 20:1).

^{1}H NMR (400 MHz, Chloroform-*d*): δ = 7.97 – 7.93 (m, 2H), 7.52 – 7.46 (m, 1H), 7.42 – 7.36 (m, 2H), 7.29 (d, *J* = 4.3 Hz, 4H), 7.25 – 7.19 (m, 1H), 5.88 – 5.77 (m, 1H), 5.35 – 5.24 (m, 2H), 4.95 (dd, *J* = 7.9, 5.1 Hz, 1H), 4.63 – 4.56 (m, 1H), 4.51 – 4.43 (m, 1H), 3.36 – 3.19 (m, 1H), 2.64 – 2.48 (m, 1H) ppm.

^{13}C NMR (101 MHz, Chloroform-*d*): δ = 197.4, 163.6 (t, *J* = 33.0 Hz), 137.8, 135.9, 133.3, 130.5, 129.3, 129.0, 128.7, 128.4, 127.8, 120.0, 115.4 (t, *J* = 250.8 Hz), 67.2, 47.0 (t, *J* = 3.8 Hz), 38.3 (t, *J* = 23.3 Hz) ppm.

^{19}F NMR (376 MHz, Chloroform-*d*): δ = -103.77 (dt, *J* = 259.5, 16.5 Hz), -104.59 (dt, J = 259.5, 16.5 Hz) ppm.

HRMS (ESI/QTOF): m/z: [M + H]$^+$ Calcd. for $C_{20}H_{19}F_2O_3^+$ 345.1297; Found 345.1300.

IR (ATR): 1768, 1683, 1448, 1299, 1263, 1208, 1099, 698 cm^{-1}.

$[\alpha]_D^{20}$ = +46.5 (c = 0.5, $CHCl_3$).

Chiral HPLC: (Chiralpak IC, 1 % *i*PrOH/hexane, 1.0 mL/min, 210 nm): tR (minor) 9.34 min, tR (major) 11.43 min, 97:3 *er*.

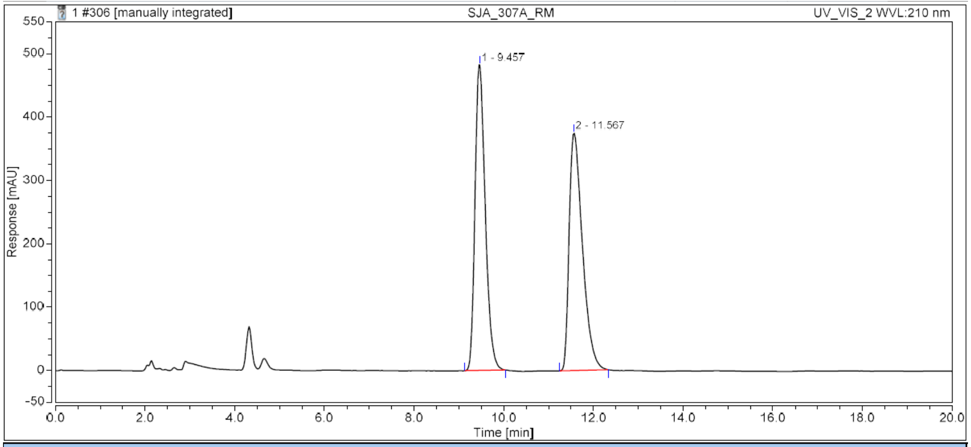

Integration Results

No.	Peak Name	Retention Time min	Area mAU*min	Height mAU	Relative Area %	Relative Height %	Amount n.a.
1		9.457	127.613	482.791	49.82	56.27	n.a.
2		11.567	128.527	375.234	50.18	43.73	n.a.
**Total:**			**256.140**	**858.025**	**100.00**	**100.00**	

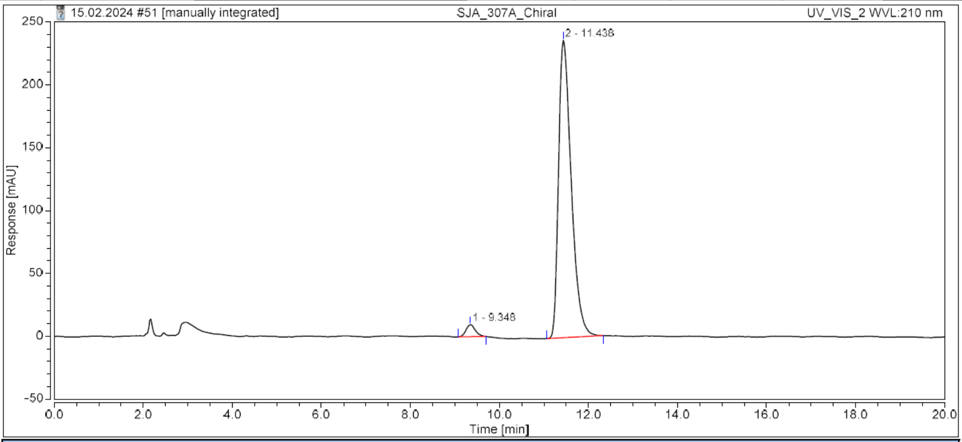

Integration Results

No.	Peak Name	Retention Time min	Area mAU*min	Height mAU	Relative Area %	Relative Height %	Amount n.a.
1		9.348	2.310	9.639	2.88	3.91	n.a.
2		11.438	78.032	236.737	97.12	96.09	n.a.
**Total:**			**80.342**	**246.376**	**100.00**	**100.00**	

Prop-2-yn-1-yl (*S*)-2,2-difluoro-5-oxo-4,5-diphenylpentanoate (7d)

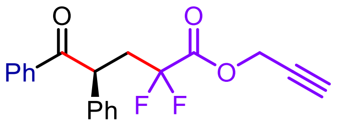

The title compound was synthesized according to the general procedure (**GP 9**), and was obtained after silica gel column chromatography (*n*-pentane : ethylacetate 20:1) as a colorless oil (75 % Yield, 26 mg, 96 % ee). $\mathbf{R_f}$ = 0.22 (*n*-pentane : ethylacetate 20:1).

^{1}H NMR (400 MHz, Chloroform-*d*): δ = 7.97 – 7.92 (m, 2H), 7.52 – 7.46 (m, 1H), 7.42 – 7.36 (m, 2H), 7.30 (d, *J* = 4.4 Hz, 4H), 7.25 – 7.19 (m, 1H), 4.94 (dd, *J* = 7.9, 5.3 Hz, 1H), 4.71 – 4.53 (m, 2H), 3.35 – 3.19 (m, 1H), 2.66 – 2.50 (m, 2H) ppm.

^{13}C NMR (101 MHz, Chloroform-*d*): δ = 197.3, 163.1 (t, *J* = 33.5 Hz), 137.6, 135.8, 133.4, 129.4, 129.0, 128.7, 128.4, 127.9, 115.3 (t, *J* = 251.4 Hz), 76.5, 75.9, 54.1, 47.0 (t, *J* = 3.8 Hz), 38.2 (t, *J* = 23.3 Hz) ppm.

^{19}F NMR (376 MHz, Chloroform-*d*): δ = -103.68 (dt, *J* = 262.0, 16.2 Hz), -104.57j (dt, J = 262.0, 16.2 Hz) ppm.

HRMS (ESI/QTOF): m/z: $[M + Na]^+$ Calcd. for $C_{20}H_{16}F_2NaO_3^+$ 365.0960; Found 365.0962.

IR (ATR): 3294, 1773, 1682, 1597, 1208, 1187, 698 cm^{-1}.

$[\alpha]_D^{20}$ = +44.4 (c = 0.6, $CHCl_3$).

Chiral HPLC: (Chiralpak IC, 1 % *i*PrOH/hexane, 1.0 mL/min, 210 nm): tR (minor) 11.0 min, tR (major) 12.72 min, 98:2 *er.*

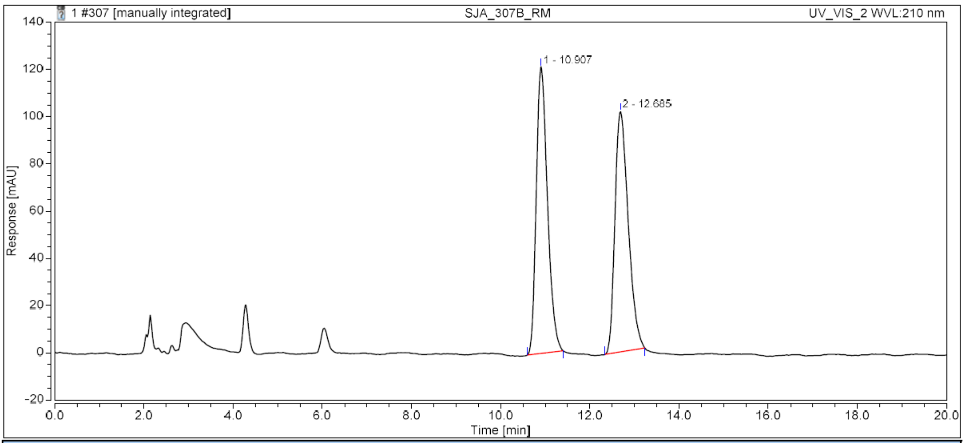

Integration Results

No.	Peak Name	Retention Time min	Area mAU*min	Height mAU	Relative Area %	Relative Height %	Amount n.a.
1		10.907	35.336	121.379	50.17	54.35	n.a.
2		12.685	35.095	101.952	49.83	45.65	n.a.
**Total:**			**70.431**	**223.331**	**100.00**	**100.00**	

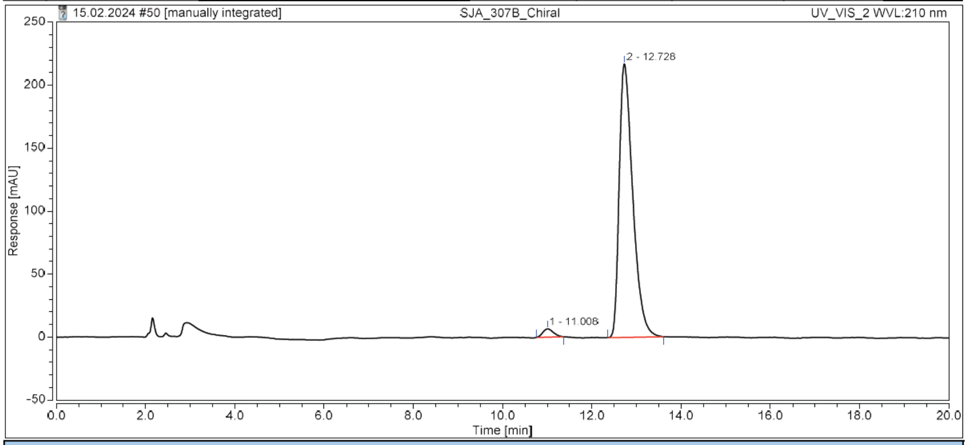

Integration Results

No.	Peak Name	Retention Time min	Area mAU*min	Height mAU	Relative Area %	Relative Height %	Amount n.a.
1		11.008	1.795	6.520	2.30	2.92	n.a.
2		12.728	76.248	216.952	97.70	97.08	n.a.
**Total:**			**78.044**	**223.472**	**100.00**	**100.00**	

(S)-*N*,*N*-diethyl-2,2-difluoro-5-oxo-4,5-diphenylpentanamide (7e)

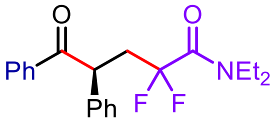

The title compound was synthesized according to the general procedure (**GP 9**), and was obtained after silica gel column chromatography (*n*-pentane : ethylacetate 4:1) as a colorless oil (62 % Yield, 22 mg, 94 % ee). $\mathbf{R_f}$ = 0.24 (*n*-pentane : ethylacetate 4:1).

^{1}H NMR (400 MHz, $CDCl_3$): δ = 7.99 – 7.94 (m, 2H), 7.51 – 7.45 (m, 1H), 7.41 – 7.35 (m, 2H), 7.34 – 7.24 (m, 4H), 7.22 – 7.16 (m, 1H), 5.06 (dd, *J* = 9.0, 3.5 Hz, 1H), 3.52 – 3.38 (m, 3H), 3.34 (q, *J* = 7.0 Hz, 2H), 2.61 – 2.45 (m, 1H), 1.17 – 1.08 (m, 6H) ppm.

^{13}C NMR (101 MHz, $CDCl_3$): δ = 198.1, 162.6 (t, *J* = 28.6 Hz), 138.8, 136.4, 133.1, 129.2, 128.9, 128.6, 128.3, 127.5, 118.7 (t, *J* = 254.8 Hz), 46.9 (t, *J* = 3.5 Hz), 42.0 (t, *J* = 6.1 Hz), 41.6, 38.7 (t, *J* = 23.0 Hz), 14.3, 12.4 ppm.

^{19}F NMR (376 MHz, $CDCl_3$): δ = -97.64 – -98.53 (m), -98.56 – -99.43 (m) ppm.

HRMS (ESI/QTOF): m/z: $[M + H]^+$ Calcd. for $C_{21}H_{24}F_2NO_2^+$ 360.1770; Found 360.1778.

IR (ATR): 1683, 1657, 1096, 794, 665 cm^{-1}.

$[\alpha]_D^{20}$ = +51.2 (c = 0.4, $CHCl_3$).

Chiral HPLC: (Chiralpak IC, 5 % *i*PrOH/hexane, 1.0 mL/min, 210 nm): tR (minor) 15.98 min, tR (major) 19.41 min, 97:3 *er.*

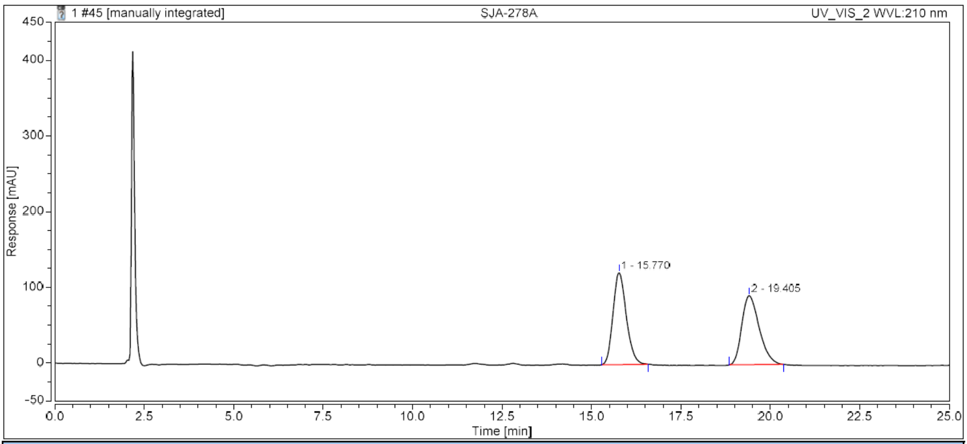

Integration Results							
No.	Peak Name	Retention Time min	Area mAU*min	Height mAU	Relative Area %	Relative Height %	Amount n.a.
1		15.770	52.275	121.218	50.39	57.06	n.a.
2		19.405	51.456	91.205	49.61	42.94	n.a.
**Total:**			**103.731**	**212.423**	**100.00**	**100.00**	

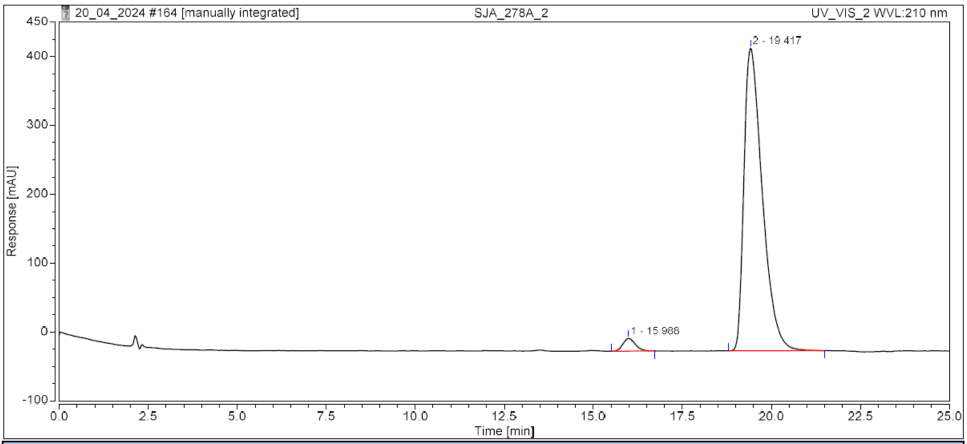

Integration Results							
No.	Peak Name	Retention Time min	Area mAU*min	Height mAU	Relative Area %	Relative Height %	Amount n.a.
1		15.988	7.427	18.535	2.75	4.04	n.a.
2		19.417	262.661	439.690	97.25	95.96	n.a.
**Total:**			**270.088**	**458.225**	**100.00**	**100.00**	

(*S*)-2,2-difluoro-1-morpholino-4,5-diphenylpentane-1,5-dione (7f)

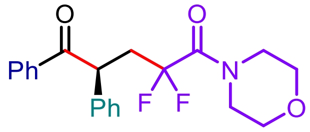

The title compound was synthesized according to the general procedure (**GP 9**), and was obtained after silica gel column chromatography (*n*-pentane : ethylacetate 4:1) as a colorless oil (61 % Yield, 23 mg, 97 % ee). **R_f** = 0.20 (*n*-pentane : ethylacetate 4:1).

^{1}H NMR (400 MHz, Chloroform-*d*): δ = 7.94 – 7.90 (m, 2H), 7.48 – 7.42 (m, 1H), 7.38 – 7.32 (m, 2H), 7.30 – 7.22 (m, 4H), 7.19 – 7.14 (m, 1H), 4.99 (dd, *J* = 8.8, 3.6 Hz, 1H), 3.71 – 3.58 (m, 6H), 3.57 – 3.52 (m, 2H), 3.49 – 3.33 (m, 1H), 2.57 – 2.41 (m, 1H) ppm.

^{13}C NMR (101 MHz, Chloroform-*d*): δ = 197.83, 161.7 (t, *J* = 28.9 Hz), 138.5, 136.1, 133.1, 129.2, 128.8, 128.6, 128.1, 127.5, 118.4 (t, *J* = 254.3 Hz), 66.6, 46.7 (t, *J* = 3.5 Hz), 46.5 (t, *J* = 6.0 Hz), 43.3, 38.4 (t, *J* = 22.6 Hz) ppm.

^{19}F NMR (376 MHz, Chloroform-*d*): δ = -97.48 (ddd, *J* = 276.0, 20.6, 14.6 Hz), -98.47 (ddd, *J* = 275.9, 20.5, 15.9 Hz) ppm.

HRMS (ESI/QTOF): m/z: $[M + Na]^+$Calcd for $C_{21}H_{21}F_2NNaO_3^+$ 396.1382; Found 396.1390.

IR (ATR): 1667, 1652, 1263, 1093, 792, 665 cm^{-1}.

$[\alpha]_D^{20}$ = +50.2 (c = 0.5, $CHCl_3$).

Chiral HPLC: (Chiralpak IC, 10 % *i*PrOH/hexane, 1.0 mL/min, 210 nm): tR (major) 14.57 min, tR (minor) 18.80 min, 98.5:1.5 *er.*

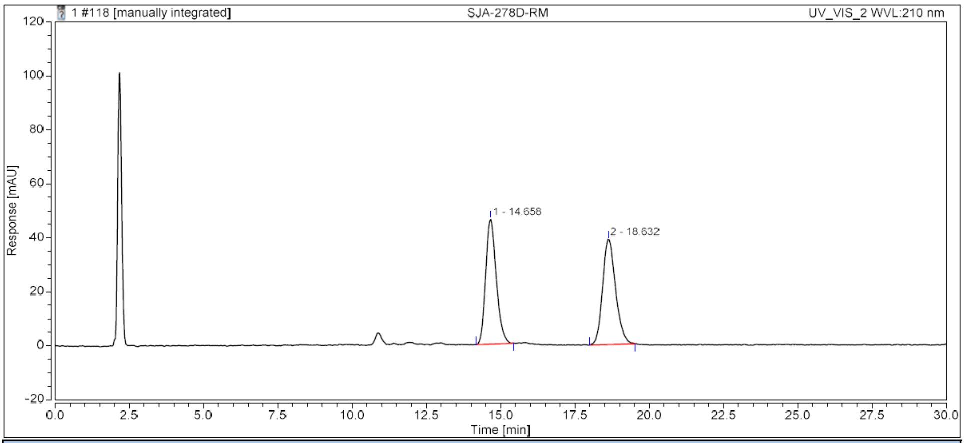

Integration Results							
No.	Peak Name	Retention Time min	Area mAU*min	Height mAU	Relative Area %	Relative Height %	Amount n.a.
1		14.658	19.128	46.293	49.24	54.27	n.a.
2		18.632	19.718	39.005	50.76	45.73	n.a.
**Total:**			**38.846**	**85.298**	**100.00**	**100.00**	

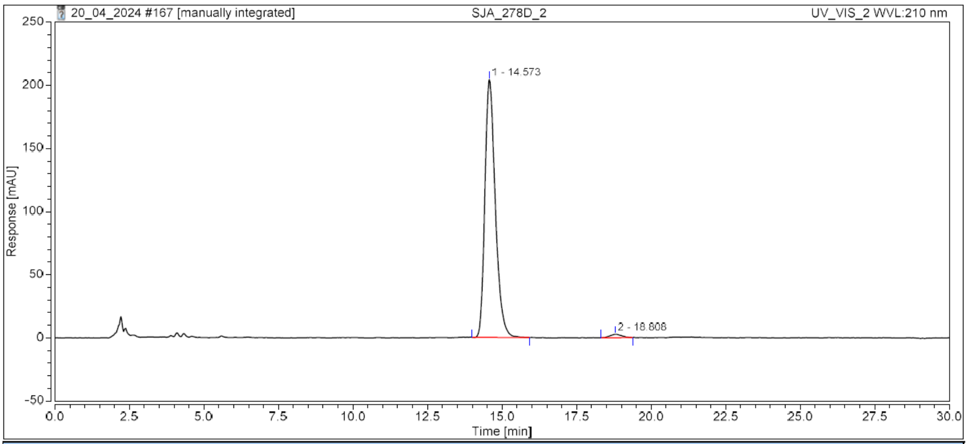

Integration Results							
No.	Peak Name	Retention Time min	Area mAU*min	Height mAU	Relative Area %	Relative Height %	Amount n.a.
1		14.573	82.758	204.044	98.56	98.71	n.a.
2		18.808	1.207	2.669	1.44	1.29	n.a.
**Total:**			**83.965**	**206.713**	**100.00**	**100.00**	

(*S*)-*N*-(*tert*-butyl)-2,2-difluoro-5-oxo-4,5-diphenylpentanamide (7g)

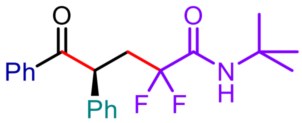

The title compound was synthesized according to the general procedure (**GP 9**), and was obtained after silica gel column chromatography (*n*-pentane : ethylacetate 4:1) as a colorless oil (63 % Yield, 23 mg, 92 % ee). **R_f** = 0.24 (*n*-pentane : ethylacetate 4:1).
^{1}H NMR (400 MHz, Chloroform-*d*): δ = 7.93 – 7.86 (m, 2H), 7.44 – 7.39 (m, 1H), 7.34 – 7.29 (m, 2H), 7.25 – 7.17 (m, 4H), 7.15 – 7.10 (m, 1H), 5.97 (s, 1H), 4.96 (dd, *J* = 8.7, 4.1 Hz, 1H), 3.29 – 3.10 (m, 1H), 2.54 – 2.37 (m, 1H), 1.27 (s, 9H) ppm.
^{13}C NMR (101 MHz, Chloroform-*d*): δ = 197.7, 163.0 (t, *J* = 27.4 Hz), 138.4, 136.2, 133.2, 129.2, 128.9, 128.7, 128.2, 127.6, 117.0 (t, *J* = 254.1 Hz), 51.9, 46.8 (t, *J* = 3.4 Hz), 37.8 (t, *J* = 23.2 Hz), 28.4 ppm.
^{19}F NMR (376 MHz, Chloroform-*d*): δ = -103.39 – -105.00 (m) ppm.
HRMS (ESI/QTOF): m/z: [M + Na]$^+$ Calcd. for $C_{21}H_{23}F_2NNaO_2^+$ 382.1589; Found 382.1591.
IR (ATR): 1687, 1597, 1263, 1258, 1071, 855 cm^{-1}.
$[\alpha]_D^{20}$ = +30.2 (c = 1, $CHCl_3$).
Chiral HPLC: (Chiralpak IC, 5 % *i*PrOH/hexane, 1.0 mL/min, 210 nm): tR (minor) 9.69 min, tR (major) 17.30 min, 96:4 er.

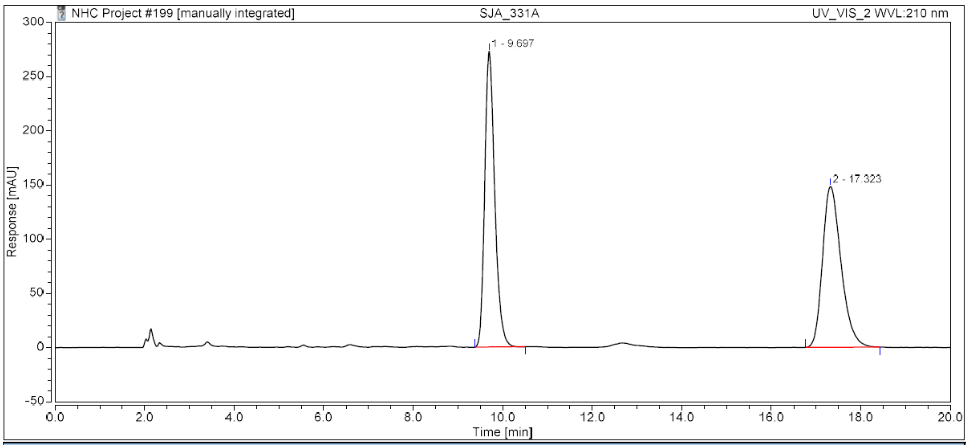

Integration Results							
No.	Peak Name	Retention Time min	Area mAU*min	Height mAU	Relative Area %	Relative Height %	Amount n.a.
1		9.697	70.762	272.899	49.90	64.76	n.a.
2		17.323	71.055	148.503	50.10	35.24	n.a.
**Total:**			**141.817**	**421.401**	**100.00**	**100.00**	

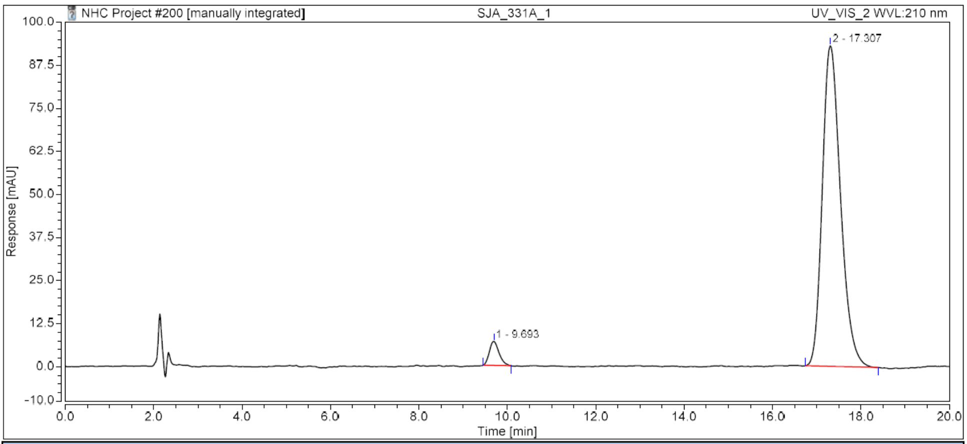

Integration Results							
No.	Peak Name	Retention Time min	Area mAU*min	Height mAU	Relative Area %	Relative Height %	Amount n.a.
1		9.693	1.761	7.008	3.84	7.00	n.a.
2		17.307	44.123	93.126	96.16	93.00	n.a.
Total:			45.884	100.134	100.00	100.00	

(*S*)-2,2-difluoro-1-(isoindolin-2-yl)-4,5-diphenylpentane-1,5-dione (7h)

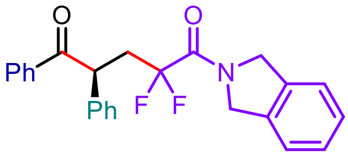

The title compound was synthesized according to the general procedure (**GP 9**), and was obtained after silica gel column chromatography (*n*-pentane : ethylacetate 4:1) as a colorless viscous oil (65 % Yield, 28 mg, 94 % ee). $\mathbf{R_f}$ = 0.22 (*n*-pentane : ethylacetate 4:1).

^{1}**H NMR** (400 MHz, Chloroform-*d*): δ = 7.99 – 7.95 (m, 2H), 7.51 – 7.45 (m, 1H), 7.39 (dd, *J* = 8.3, 6.9 Hz, 2H), 7.34 – 7.16 (m, 9H), 5.09 (dd, *J* = 8.9, 3.7 Hz, 1H), 5.02 (s, 2H), 4.80 (s, 2H), 3.55 – 3.38 (m, 1H), 2.70 – 2.52 (m, 1H) ppm.

^{13}**C NMR** (101 MHz, Chloroform-*d*): δ = 197.7, 162.1 (t, *J* = 30.1 Hz), 138.2, 136.2 (t, *J* = 2.2 Hz), 136.0, 134.4, 132.9, 128.9, 128.6, 128.4, 127.9, 127.6, 127.5, 127.3, 122.5, 122.3, 117.9 (t, *J* = 253.3 Hz), 53.6, 52.2 (t, *J* = 6.9 Hz), 46.6 (t, *J* = 3.7 Hz), 37.7 (t, *J* = 22.9 Hz) ppm.

^{19}**F NMR** (376 MHz, $CDCl_3$): δ = -101.56 (dt, *J* = 274.6, 17.1 Hz), -102.5 (dt, *J* = 274.6, 17.1 Hz) ppm.

HRMS (ESI/QTOF): m/z: $[M + Na]^+$ Calcd for $C_{25}H_{21}F_2NNaO_2^+$ 428.1433; Found 428.1438.

$[\alpha]_D^{20}$ = +27.1 (c = 1, $CHCl_3$).

IR (ATR): 1665, 1596, 1580, 1448, 1230, 1089, 1002, 950, 860, 587 cm^{-1}.

Chiral HPLC: (Chiralpak IB, 5 % *i*PrOH/hexane, 1.0 mL/min, 210 nm): tR (major) 6.89 min, tR (minor) 15.08 min, 97:3 *er.*

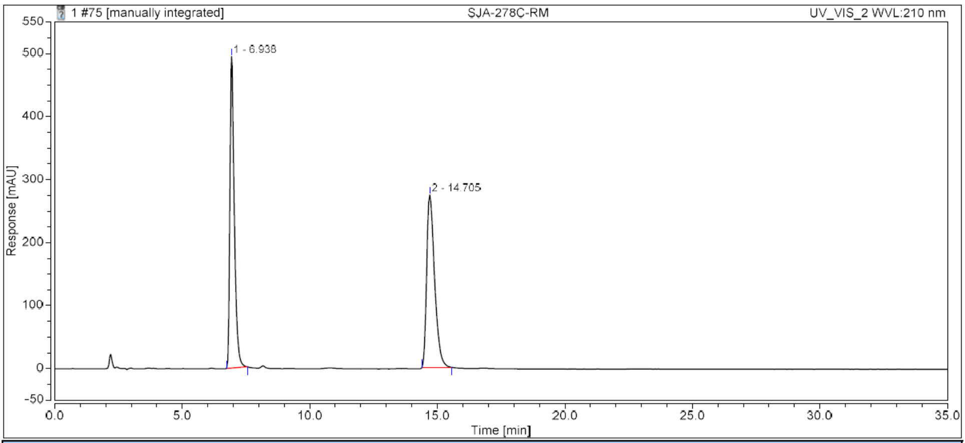

Integration Results

No.	Peak Name	Retention Time min	Area mAU*min	Height mAU	Relative Area %	Relative Height %	Amount n.a.
1		6.938	98.746	494.396	50.21	64.37	n.a.
2		14.705	97.926	273.702	49.79	35.63	n.a.
**Total:**			**196.673**	**768.098**	**100.00**	**100.00**	

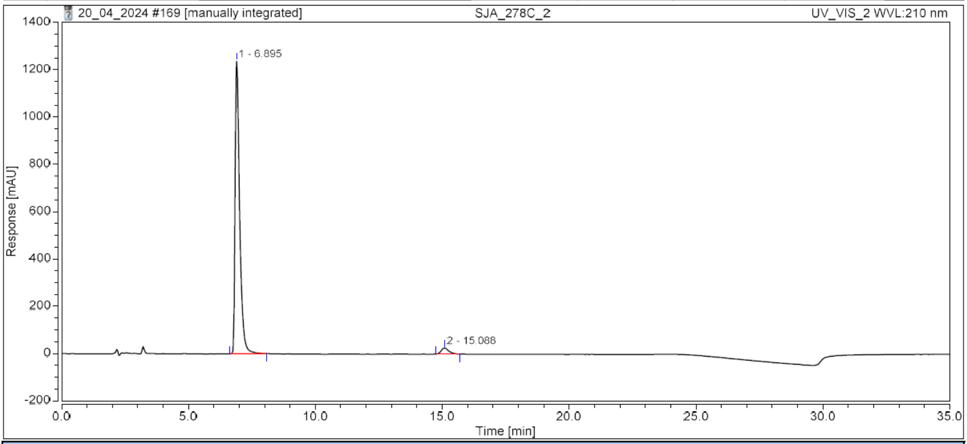

Integration Results

No.	Peak Name	Retention Time min	Area mAU*min	Height mAU	Relative Area %	Relative Height %	Amount n.a.
1		6.895	256.298	1235.902	96.76	98.02	n.a.
2		15.088	8.581	24.926	3.24	1.98	n.a.
**Total:**			**264.879**	**1260.828**	**100.00**	**100.00**	

(*S*)-4,4-difluoro-1,2-diphenyl-4-(phenylsulfonyl)butan-1-one (7i)

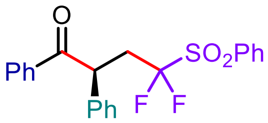

The title compound was synthesized according to the general procedure (**GP 9**), and was obtained after silica gel column chromatography (*n*-pentane : ethylacetate 4:1) as a colorless oil (60 % Yield, 24 mg, 92 % ee). R_f = 0.20 (*n*-pentane : ethylacetate 4:1).

^{1}**H NMR** (400 MHz, $CDCl_3$): δ = 7.99 – 7.93 (m, 4H), 7.78 – 7.71 (m, 1H), 7.62 – 7.56 (m, 2H), 7.53 – 7.46 (m, 1H), 7.43 – 7.37 (m, 2H), 7.36 – 7.27 (m, 4H), 7.25 – 7.20 (m, 1H), 5.16 (dd, *J* = 8.3, 4.7 Hz, 1H), 3.69 – 3.52 (m, 1H), 2.88 – 2.72 (m, 1H) ppm.

^{13}**C NMR** (101 MHz, $CDCl_3$): δ = 196.8, 137.7, 135.8, 135.5, 133.4, 132.3, 130.9, 129.47, 129.46, 129.0, 128.7, 128.2, 127.9, 123.8 (t, *J* = 287.3 Hz), 46.4, 33.3 (t, *J* = 19.1 Hz) ppm.

^{19}**F NMR** (376 MHz, $CDCl_3$): δ = -100.4 (ddd, *J* = 229.2, 22.8, 10.3 Hz), -101.8 (ddd, *J* = 231.1, 24.6, 15.3 Hz) ppm.

HRMS (ESI/QTOF): m/z: $[M + Na]^+$ Calcd. for $C_{22}H_{18}F_2NaO_3S^+$ 423.0837; Found 423.0839.

IR (ATR): 1684, 1448, 1344, 1162, 757, 604 cm^{-1}.

$[\alpha]_D^{20}$ = -21.2 (c = 0.7, $CHCl_3$).

Chiral HPLC: (Chiralpak IC, 5 % *i*PrOH/hexane, 1.0 mL/min, 210 nm): tR (major) 13.38 min, tR (minor) 14.95 min, 96:4 *er.*

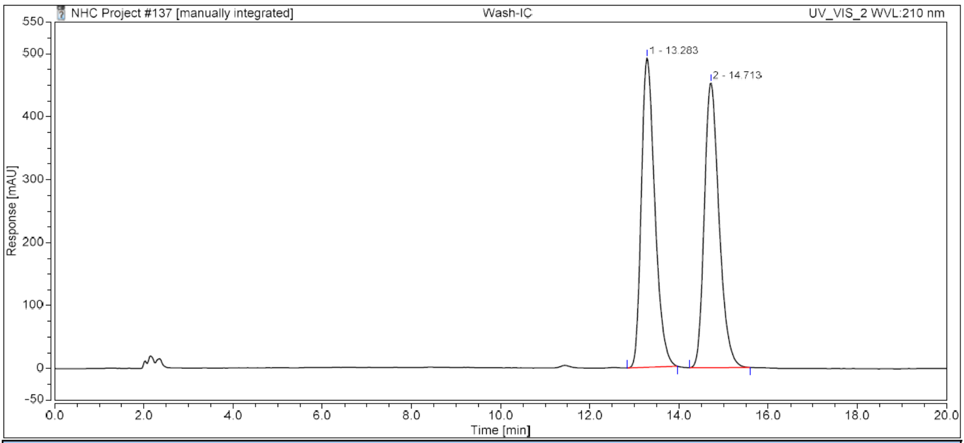

Integration Results							
No.	Peak Name	Retention Time min	Area mAU*min	Height mAU	Relative Area %	Relative Height %	Amount n.a.
1		13.283	172.411	491.871	49.87	52.07	n.a.
2		14.713	173.323	452.776	50.13	47.93	n.a.
Total:			345.734	944.647	100.00	100.00	

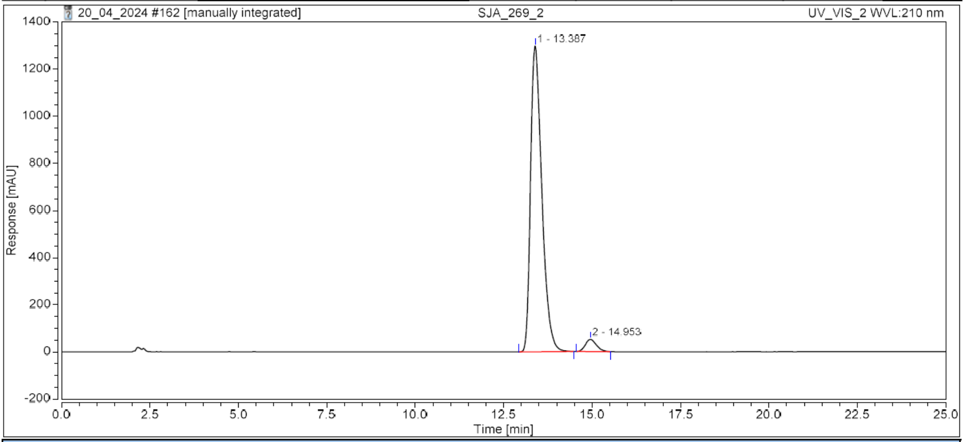

Integration Results							
No.	Peak Name	Retention Time min	Area mAU*min	Height mAU	Relative Area %	Relative Height %	Amount n.a.
1		13.387	480.094	1297.827	96.17	96.15	n.a.
2		14.953	19.127	52.030	3.83	3.85	n.a.
Total:			499.221	1349.857	100.00	100.00	

(*S*)-4-bromo-4,4-difluoro-1,2-diphenylbutan-1-one (7j)

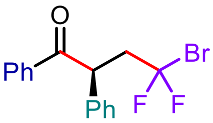

The title compound was synthesized according to the general procedure (**GP 9**), and was obtained after silica gel column chromatography (*n*-pentane : ethylacetate 40:1) as a colorless solid (87 % Yield, 29 mg, 86 % ee). **R$_f$** = 0.40 (*n*-pentane : ethylacetate 40:1).

MP: 116-119 C

^{1}H NMR (400 MHz, Chloroform-*d*): δ = 8.01 – 7.95 (m, 2H), 7.54 – 7.48 (m, 1H), 7.45 – 7.38 (m, 2H), 7.34 – 7.28 (m, 4H), 7.27 – 7.20 (m, 1H), 5.00 (dd, *J* = 7.7, 4.5 Hz, 1H), 3.70 (tdd, *J* = 15.1, 12.9, 7.7 Hz, 1H), 2.89 – 2.74 (tdd, *J* = 15.2, 13.1, 4.5 Hz, 1H) ppm.

^{13}C NMR (101 MHz, Chloroform-*d*): δ = 196.8, 137.5, 135.9, 133.5, 129.4, 128.9, 128.8, 128.2, 127.9, 121.7 (t, *J* = 306.0 Hz) 48.8 (t, *J* = 2.6 Hz), 47.6 (t, *J* = 21.0 Hz) ppm.

^{19}F NMR (377 MHz, Chloroform-*d*): δ = -42.70 (ddd, *J* = 156.6, 15.5, 13.2 Hz), -43.45 (ddd, *J* = 156.2, 15.3, 13.0 Hz).

HRMS (ESI/QTOF): m/z: $[M + H]^+$ Calcd. for $C_{16}H_{14}BrF_2O^+$ 339.0191; Found 339.0179.

IR (ATR): 1684, 1597, 1494, 1448, 1322, 1272, 1231, 1018, 696 cm^{-1}

$[\alpha]_D^{20}$ = +27.2 (c = 0.7, $CHCl_3$).

Chiral HPLC: (Chiralpak IB, 0.1 % *i*PrOH/hexane, 1.0 mL/min, 254 nm): tR (minor) 8.36 min, tR (major) 11.71 min, 93:7 *er.*

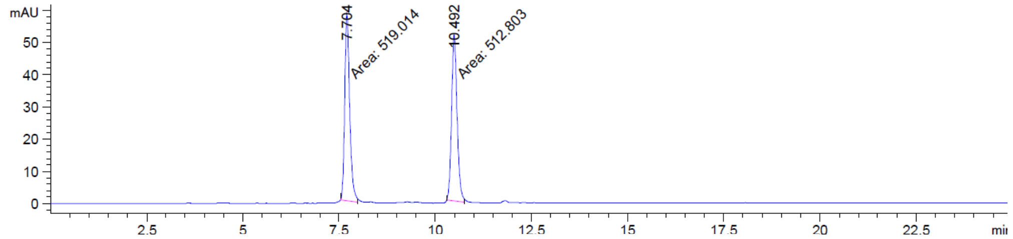

Peak #	RetTime [min]	Type	Width [min]	Area [mAU*s]	Height [mAU]	Area %
1	7.704	MM	0.1493	519.01373	57.94661	50.3010
2	10.492	MM	0.1659	512.80298	51.51036	49.6990

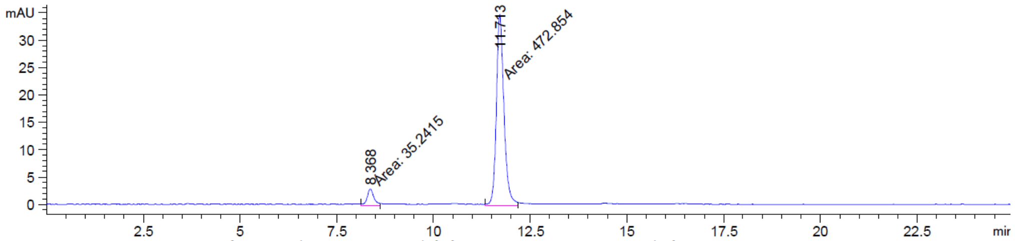

Peak #	RetTime [min]	Type	Width [min]	Area [mAU*s]	Height [mAU]	Area %
1	8.368	MM	0.1970	35.24149	2.98177	6.9360
2	11.713	MM	0.2260	472.85406	34.87809	93.0640

(*S*)-4,4,4-Trifluoro-1,2-diphenylbutan-1-one (7k)

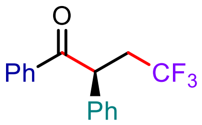

The title compound was synthesized according to the general procedure (**GP 9**), using CF_3I (10% solution in THF, 0.2 mmol, 2 eq.) as a trifluoromethyl radical precursor, and was obtained after silica gel column chromatography (*n*-pentane : ethylacetate 50:1) as a colorless oil (54 % Yield, 15 mg, 78 % ee). **R_f** = 0.34 (*n*-pentane : ethylacetate 50:1).

^{1}H NMR (400 MHz, Chloroform-*d*): δ = 7.98 – 7.89 (m, 2H), 7.51 – 7.46 (m, 1H), 7.41 – 7.34 (m, 2H), 7.32 – 7.27 (m, 4H), 7.26 – 7.19 (m, 1H), 4.89 (dd, *J* = 7.7, 5.4 Hz, 1H), 3.30 (dqd, *J* = 15.1, 10.8, 7.7 Hz, 1H), 2.53 (dqd, *J* = 15.1, 10.8, 5.4 Hz, 1H) ppm.

^{13}C NMR (101 MHz, Chloroform-*d*): δ = 196.8, 137.5, 135.8, 133.5, 129.4, 128.9, 128.8, 128.1, 127.9, 126.5 (q, *J* = 277.2 Hz), 47.3 (q, *J* = 2.4 Hz), 37.5 (q, *J* = 28.2 Hz) ppm.

^{19}F NMR (376 MHz, Chloroform-*d*): δ = -64.60 (t, *J* = 10.8 Hz) ppm.

HRMS (Sicrit plasma/LTQ-Orbitrap): m/z: $[M + H]^+$ Calcd for $C_{16}H_{14}F_3O^+$ 279.0991; Found 279.0990.

IR (ATR): 1684, 1287, 1106, 1074, 696 cm^{-1}.

$[\alpha]_D^{20}$ = +49.8 (c = 0.5, CHCl3).

Chiral HPLC: (Chiralpak IA, 0.1 % iPrOH/hexane, 1.0 mL/min, 254 nm): tR (major) 8.80 min, tR (minor) 9.68 min, 89:11 er.

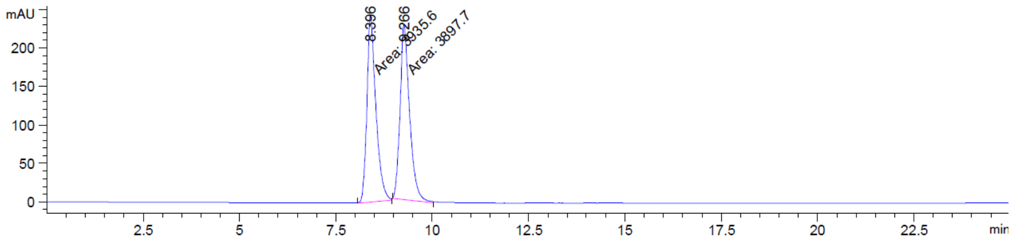

Peak #	RetTime [min]	Type	Width [min]	Area [mAU*s]	Height [mAU]	Area %
1	8.396	MM	0.2700	3935.60132	242.92004	50.2419
2	9.266	MM	0.2857	3897.69800	227.37007	49.7581

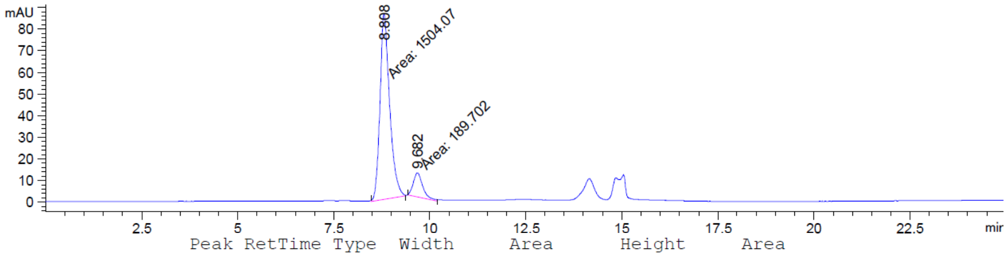

Peak #	RetTime [min]	Type	Width [min]	Area [mAU*s]	Height [mAU]	Area %
1	8.808	MM	0.2920	1504.06909	85.85258	88.8000
2	9.682	MM	0.2837	189.70155	11.14492	11.2000

(*S*)-4,4,5,5,6,6,7,7,7-Nonafluoro-1,2-diphenylheptan-1-one (7l)

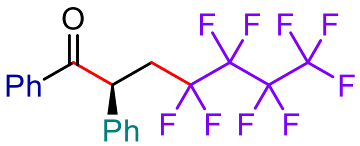

The title compound was synthesized according to the general procedure (**GP 9**), using perfluorobutyl iodide (0.2 mmol, 2 eq.) as a perfluorobutyl radical precursor, and was obtained after silica gel column chromatography (*n*-pentane : ethylacetate 80:1) as a colorless solid (57 % Yield, 24 mg, 80% ee). **R_f** = 0.40 (*n*-pentane : ethylacetate 80:1). **MP:** 68 – 70 °C

^{1}H NMR (400 MHz, Chloroform-*d*): δ = 8.01 – 7.94 (m, 2H), 7.56 – 7.48 (m, 1H), 7.45 – 7.39 (m, 2H), 7.35 – 7.31 (m, 4H), 7.29 – 7.22 (m, 1H), 5.04 (dd, *J* = 8.4, 4.1 Hz, 1H), 3.58 – 3.33 (m, 1H), 2.60 – 2.26 (m, 1H) ppm.

^{13}C NMR (101 MHz, Chloroform-*d*): δ = 196.8, 137.8, 135.7, 133.5, 129.5, 128.9, 128.8, 128.2, 128.0, 122.95 – 105.33 (m), 45.7, 34.4 (t, *J* = 20.8 Hz) ppm.

^{19}F NMR (376 MHz, Chloroform-*d*): δ = -81.03 (tt, *J* = 9.5, 3.2 Hz), -111.50 – -114.34 (m), -124.38 (tq, *J* = 9.9, 5.5 Hz), -125.90 (qd, *J* = 12.4, 11.1, 4.4 Hz) ppm.

HRMS (ESI/QTOF): m/z: $[M + H]^+$ Calcd for $C_{19}H_{14}F_9O^+$ 429.0895; Found 429.0905.

IR (ATR): 1685, 1448, 1232, 1133, 698 cm^{-1}.

$[\alpha]_D^{20}$ = +56.4 (c = 0.5, CHCl3).

Chiral HPLC: (Chiralpak IB, 0.1 % iPrOH/hexane, 1.0 mL/min, 254 nm): tR (major) 7.57 min, tR (minor) 8.18 min, 90:10 er.

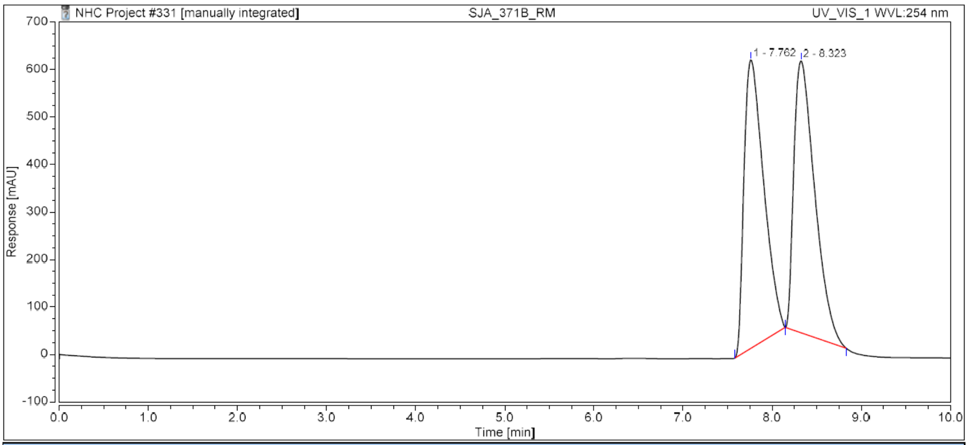

Integration Results

No.	Peak Name	Retention Time min	Area mAU*min	Height mAU	Relative Area %	Relative Height %	Amount n.a.
1		7.762	154.704	607.360	49.85	51.45	n.a.
2		8.323	155.645	573.130	50.15	48.55	n.a.
**Total:**			**310.350**	**1180.490**	**100.00**	**100.00**	

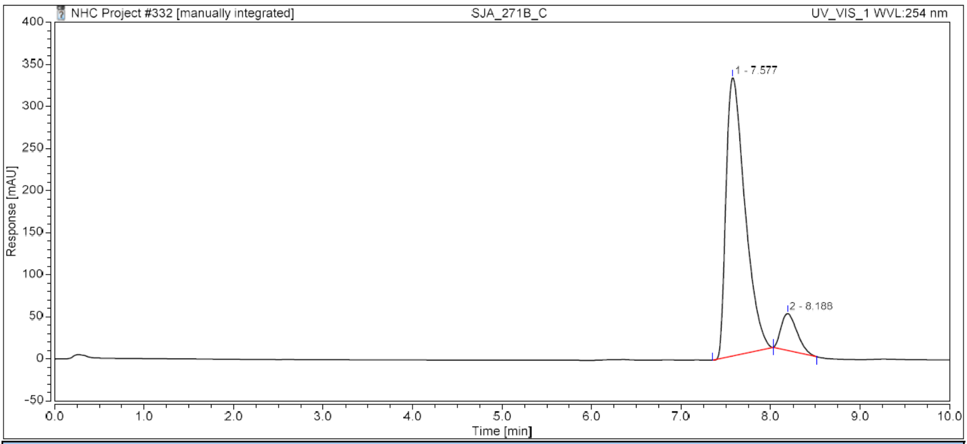

Integration Results							
No.	Peak Name	Retention Time min	Area mAU*min	Height mAU	Relative Area %	Relative Height %	Amount n.a.
1		7.577	81.025	331.045	89.96	88.34	n.a.
2		8.188	9.043	43.703	10.04	11.66	n.a.
**Total:**			**90.069**	**374.749**	**100.00**	**100.00**	

(*S*)-4,4,4-trichloro-1,2-diphenylbutan-1-one (7m)

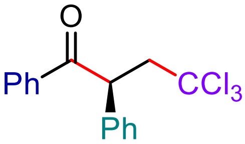

The title compound was synthesized according to the general procedure (**GP 9**), and was obtained after silica gel column chromatography (*n*-pentane : ethylacetate 40:1) as a colorless solid (56 % Yield, 18 mg, 84 % ee). $\mathbf{R_f}$ = 0.32 (*n*-pentane : ethylacetate 40:1).

MP: 101 – 102 °C

^{1}**H NMR** (400 MHz, Chloroform-*d*): δ = 8.04 – 7.99 (m, 2H), 7.55 – 7.49 (m, 1H), 7.45 – 7.39 (m, 2H), 7.37 – 7.28 (m, 4H), 7.27 – 7.20 (m, 1H), 5.15 (dd, *J* = 7.9, 2.5 Hz, 1H), 4.27 (dd, *J* = 14.9, 7.9 Hz, 1H), 3.04 (dd, *J* = 14.9, 2.5 Hz, 1H) ppm.

^{13}**C NMR** (101 MHz, Chloroform-*d*): δ = 197.4, 138.0, 136.3, 133.4, 129.5, 129.0, 128.8, 128.3, 127.8, 98.7, 57.5, 51.2 ppm.

HRMS (Sicrit plasma/LTQ-Orbitrap): m/z: [M + H]$^+$ Calcd for $C_{16}H_{14}Cl_3O^+$ 327.0105; Found 327.0106.

$[\alpha]_D^{20}$ = +41.2 (c = 0.5, $CHCl_3$).

Chiral HPLC: (Chiralpak IA, 0.5 % *i*PrOH/hexane, 1.0 mL/min, 210 nm): tR (major) 5.57 min, tR (minor) 7.22 min, 92:8 *er.*

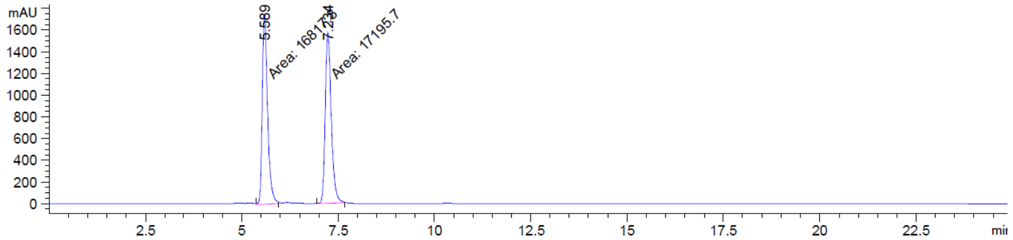

Peak #	RetTime [min]	Type	Width [min]	Area [mAU*s]	Height [mAU]	Area %
1	5.589	MM	0.1598	1.68178e4	1754.19592	49.4445
2	7.234	MM	0.1820	1.71957e4	1574.67468	50.5555

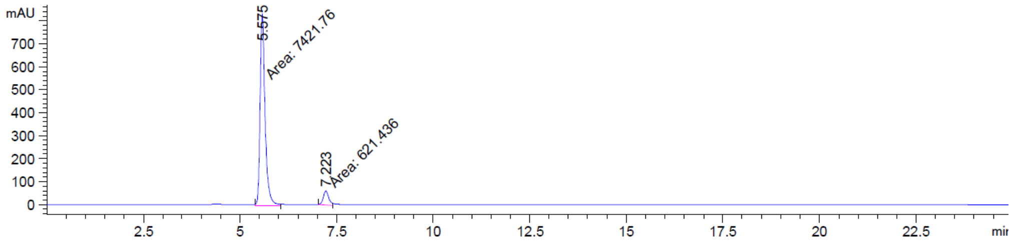

Peak #	RetTime [min]	Type	Width [min]	Area [mAU*s]	Height [mAU]	Area %
1	5.575	MM	0.1487	7421.76367	831.99316	92.2738
2	7.223	MM	0.1681	621.43585	61.60125	7.7262

Ethyl (*S*)-2,2-difluoro-4-(4-(4-((1-isopropoxy-2-methyl-1-oxopropan-2-yl)oxy)benzoyl)phenyl)-5-oxo-5-phenylpentanoate (8a)

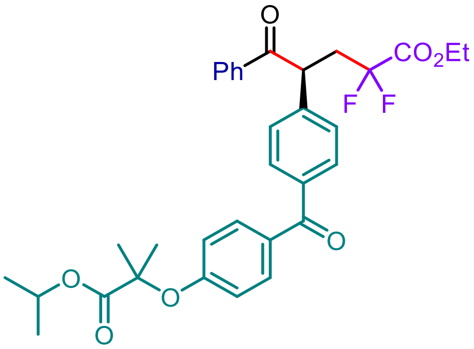

The title compound was synthesized according to the general procedure (**GP 9**), and was obtained after silica gel column chromatography (*n*-pentane : ethylacetate 4:1) as a colorless solid (78 % Yield, 45 mg, 94 % ee). **R$_f$** = 0.22 (*n*-pentane : ethylacetate 4:1).

MP: 181-182 C.

^{1}H NMR (400 MHz, Chloroform-*d*): δ = 7.99 – 7.93 (m, 2H), 7.73 – 7.64 (m, 4H), 7.56 – 7.49 (m, 1H), 7.45 – 7.37 (m, 4H), 6.86 – 6.80 (m, 2H), 5.14 – 5.00 (m, 2H), 4.22 (dq, *J* = 10.7, 7.2 Hz, 1H), 4.13 (dq, *J* = 10.8, 7.1 Hz, 1H), 3.40 – 3.24 (m, 1H), 2.63 – 2.47 (m, 1H), 1.65 (s, 6H), 1.25 (t, *J* = 7.1 Hz, 3H), 1.19 (d, *J* = 6.3 Hz, 6H) ppm.

^{13}C NMR (101 MHz, Chloroform-*d*): δ = 196.9, 194.8, 173.2, 163.8 (t, *J* = 32.4 Hz), 159.8, 142.0, 137.6, 135.7, 133.6, 132.1, 130.7, 130.3, 128.99, 128.90, 128.2, 117.3, 115.2 (t, *J* = 251.1 Hz), 79.5, 69.4, 63.1, 46.8 (t, *J* = 3.8 Hz), 38.1 (t, *J* = 23.3 Hz), 25.5, 25.4, 21.6, 13.9 ppm.

^{19}F NMR (376 MHz, Chloroform-*d*): δ = -103.9 (dt, *J* = 260.0, 16.6 Hz), -104.8 (dt, *J* = 260.0, 16.6 Hz) ppm.

HRMS (ESI/QTOF): m/z: $[M + H]^+$ Calcd. for $C_{33}H_{35}F_2O_7^+$ 581.2345; Found 581.2353.

IR (ATR): 1766, 1729, 1685, 1653, 1589, 1504, 1175, 1146, 1099, 929, 735 cm^{-1}.

$[\alpha]_D^{20}$ = +41.3 (c = 0.5, $CHCl_3$).

Chiral HPLC: (Chiralpak IC, 15 % *i*PrOH/hexane, 1.0 mL/min, 210 nm): tR (minor) 16.55 min, tR (major) 19.15 min, 97:3 *er.*

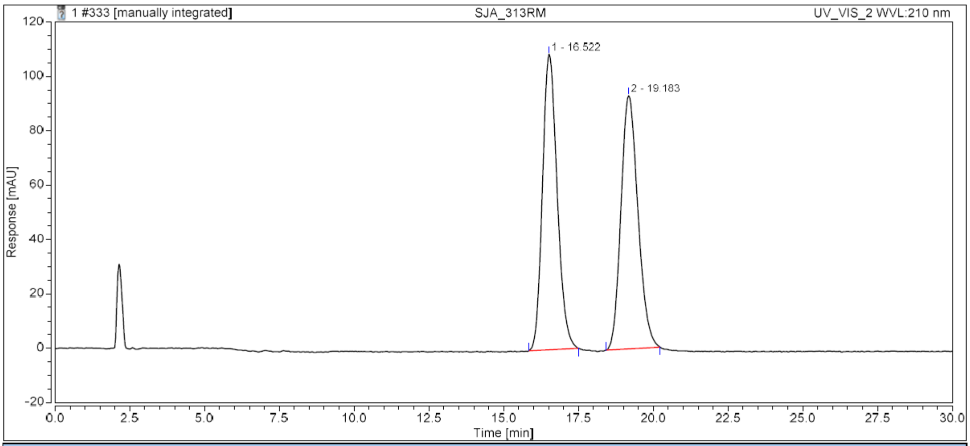

Integration Results

No.	Peak Name	Retention Time min	Area mAU*min	Height mAU	Relative Area %	Relative Height %	Amount n.a.
1		16.522	61.900	108.792	50.26	53.87	n.a.
2		19.183	61.272	93.159	49.74	46.13	n.a.
**Total:**			**123.172**	**201.951**	**100.00**	**100.00**	

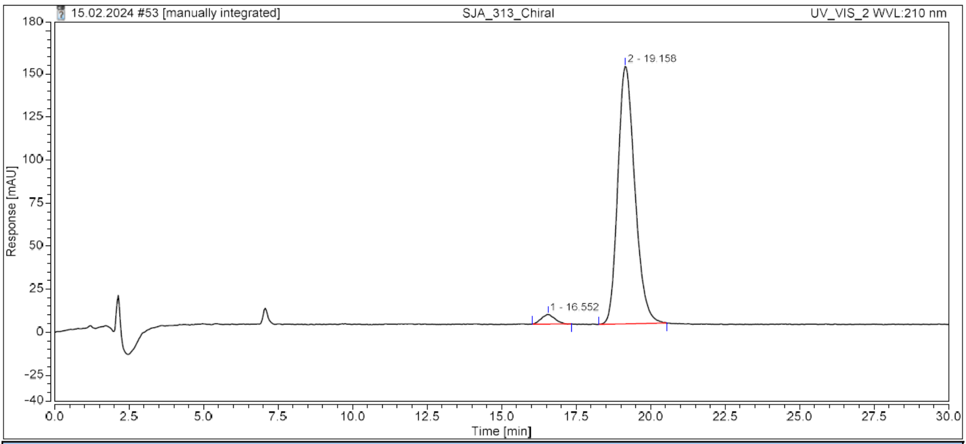

Integration Results

No.	Peak Name	Retention Time min	Area mAU*min	Height mAU	Relative Area %	Relative Height %	Amount n.a.
1		16.552	2.866	5.572	2.81	3.59	n.a.
2		19.158	99.080	149.661	97.19	96.41	n.a.
**Total:**			**101.946**	**155.233**	**100.00**	**100.00**	

Ethyl (*S*)-2,2-difluoro-4-((8*R*,9*S*,13*S*,14*S*)-13-methyl-17-oxo-7,8,9,11,12,13,14,15,16,17-decahydro-6*H*-cyclopenta[a]phenanthren-3-yl)-5-oxo-5-phenylpentanoate (8b)

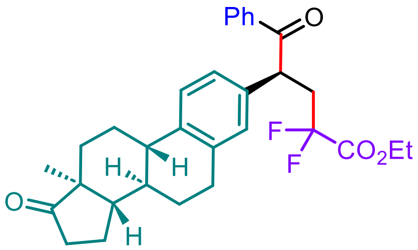

The title compound was synthesized according to the general procedure (**GP 9**), and was obtained after silica gel column chromatography (*n*-pentane : ethylacetate 4:1) as a colorless solid (52 % Yield, 26 mg, 90 % de). $\mathbf{R_f}$ = 0.20 (*n*-pentane : ethylacetate 4:1).
MP: 196-187 C.
^{1}H NMR (400 MHz, Chloroform-*d*): δ = 8.00 – 7.94 (m, 2H), 7.52 – 7.47 (m, 1H), 7.44 – 7.37 (m, 2H), 7.22 – 7.16 (m, 1H), 7.11 – 7.04 (m, 1H), 7.01 – 6.97 (m, 1H), 4.90 (dd, *J* = 8.5, 4.4 Hz, 1H), 4.23 – 4.14 (m, 1H), 4.13 – 4.02 (m, 1H), 3.37 – 3.19 (m, 1H), 2.91 – 2.79 (m, 2H), 2.56 – 2.40 (m, 2H), 2.40 – 2.29 (m, 1H), 2.26 – 1.87 (m, 5H), 1.66 – 1.31 (m, 6H), 1.22 (td, *J* = 7.2, 1.2 Hz, 3H), 0.87 (d, *J* = 2.3 Hz, 3H) ppm.
^{13}C NMR (101 MHz, Chloroform-*d*): δ = 220.7, 197.3 (d, *J* = 2.8 Hz), 163.8 (td, *J* = 32.5, 2.4 Hz), 139.2, 137.4, 135.9, 135.2 (d, *J* = 4.5 Hz), 133.2, 128.9, 128.6, 128.4 (d, *J* = 6.9 Hz), 126.2 (d, *J* = 2.6 Hz), 125.6 (d, *J* = 1.4 Hz), 115.3 (t, *J* = 250.7 Hz), 62.9, 50.4, 47.9, 46.2 (t, *J* = 3.6 Hz), 44.2, 38.3 (t, *J* = 22.8 Hz), 37.9, 35.8, 31.5, 29.3 (d, *J* = 5.1 Hz), 26.3 (d, *J* = 2.0 Hz), 25.6, 21.5, 13.8, 13.7 ppm.
^{19}F NMR (376 MHz, Chloroform-*d*): δ = -103.64 – -105.28 (m) ppm.
HRMS (ESI/QTOF): *m/z*: $[M + Na]^+$ Calcd. for $C_{31}H_{34}F_2NaO_4^+$: 531.2317; Found 531.2318.
IR (ATR): 1767, 1738, 1683, 1596, 1579, 1190, 1095, 661 cm^{-1}.
$[\alpha]_D^{20}$ = +32.4 (c = 0.6, $CHCl_3$).
Chiral HPLC: (Chiralpak IB, 3 % *i*PrOH/hexane, 1.0 mL/min, 210 nm): tR (minor) 10.66 min, tR (major) 11.58 min, 95:5 *dr.*

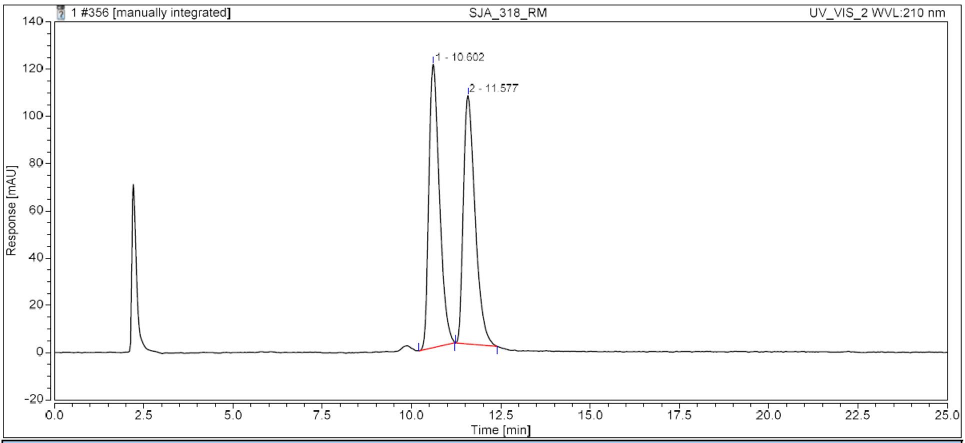

Integration Results							
No.	Peak Name	Retention Time min	Area mAU*min	Height mAU	Relative Area %	Relative Height %	Amount n.a.
1		10.602	41.312	119.918	50.79	53.26	n.a.
2		11.577	40.035	105.235	49.21	46.74	n.a.
Total:			**81.347**	**225.153**	**100.00**	**100.00**	

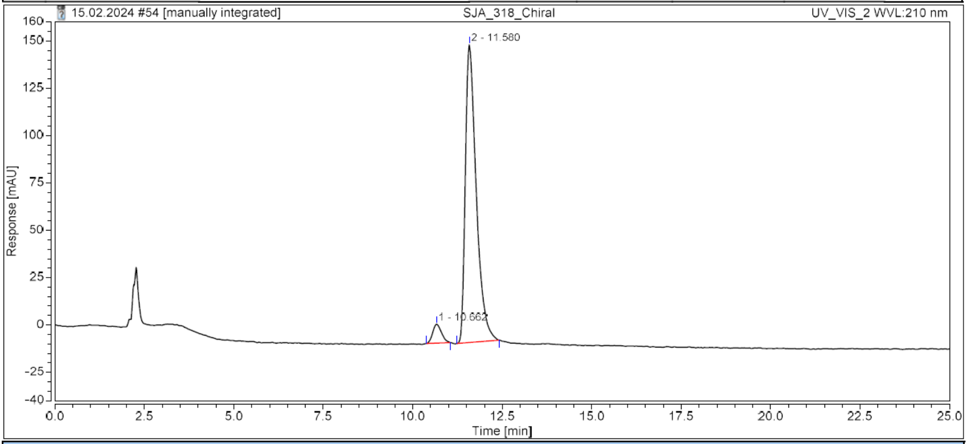

Integration Results							
No.	Peak Name	Retention Time min	Area mAU*min	Height mAU	Relative Area %	Relative Height %	Amount n.a.
1		10.662	2.824	9.986	4.85	5.97	n.a.
2		11.580	55.399	157.238	95.15	94.03	n.a.
Total:			**58.223**	**167.224**	**100.00**	**100.00**	

Ethyl (*S*)-4-((*R*)-2,8-dimethyl-2-((4*R*,8*R*)-4,8,12-trimethyltridecyl)chroman-6-yl)-2,2-difluoro-5-oxo-5-phenylpentanoate (8c)

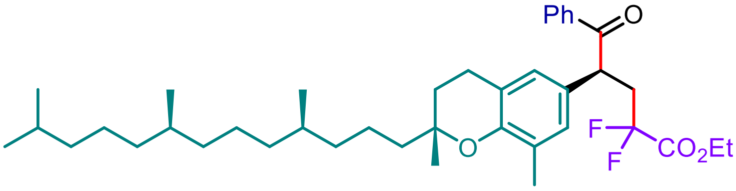

The title compound was synthesized according to the general procedure (**GP 9**), and was obtained after silica gel column chromatography (*n*-pentane : ethylacetate 20:1) as a colorless gel (73 % Yield, 47 mg, 92 % de). $\mathbf{R_f}$ = 0.24 (*n*-pentane : ethylacetate 20:1).

^{1}H NMR (400 MHz, Chloroform-*d*): δ = 8.00 – 7.95 (m, 2H), 7.52 – 7.46 (m, 1H), 7.44 – 7.37 (m, 2H), 6.87 – 6.82 (m, 1H), 6.79 – 6.75 (m, 1H), 4.80 (dd, *J* = 8.4, 4.5 Hz, 1H), 4.16 (dq, *J* = 10.7, 7.2 Hz, 1H), 4.04 (dq, *J* = 10.7, 7.2 Hz, 1H), 3.30 – 3.14 (m, 1H), 2.74 – 2.59 (m, 2H), 2.55 – 2.39 (m, 1H), 2.09 (s, 3H), 1.80 – 1.63 (m, 2H), 1.56 – 1.47 (m, 3H), 1.42 – 1.29 (m, 4H), 1.28 – 1.18 (m, 13H), 1.17 – 1.10 (m, 3H), 1.10 – 0.97 (m, 4H), 0.86 (d, *J* = 6.6 Hz, 6H), 0.83 (d, *J* = 6.3 Hz, 6H) ppm.

^{13}C NMR (101 MHz, Chloroform-*d*): δ = 197.7, 164.0 (t, *J* = 32.6 Hz), 151.7, 136.2, 133.1, 129.0, 128.6, 128.1 (d, *J* = 3.8 Hz), 127.6, 127.1, 126.6 (d, *J* = 3.4 Hz), 121.2, 115.5 (t, *J* = 250.3 Hz), 76.3, 62.9, 46.1 (t, *J* = 4.0 Hz), 40.4 (d, *J* = 5.1 Hz), 39.5, 38.6 (t, *J* = 23.3 Hz), 37.58, 37.56, 37.4, 32.9, 32.8, 31.1, 28.1, 24.9, 24.5, 24.3 (d, *J* = 4.0 Hz), 22.8, 22.7, 22.3, 21.1, 19.8, 19.7, 16.2, 13.8 ppm.

^{19}F NMR (376 MHz, Chloroform-*d*): δ = -104.0 (dt, *J* = 258.4, 16.6 Hz), -104.8 (dt, *J* = 258.4, 16.6 Hz) ppm.
HRMS (ESI/QTOF): m/z: [M + Na]$^+$ Calcd for $C_{40}H_{58}F_2NaO_4^+$ 663.4195; Found 663.4205
IR (ATR): 2926, 1769, 1683, 1597, 1579, 1197, 1002, 854 cm^{-1}.
$[\alpha]_D^{20}$ = +12.4 (c = 0.5, $CHCl_3$).
Chiral HPLC: (Chiralpak IC, 1 % *i*PrOH/hexane, 1.0 mL/min, 210 nm): tR (minor) 5.24 min, tR (major) 6.00 min, 96:4 *dr.*

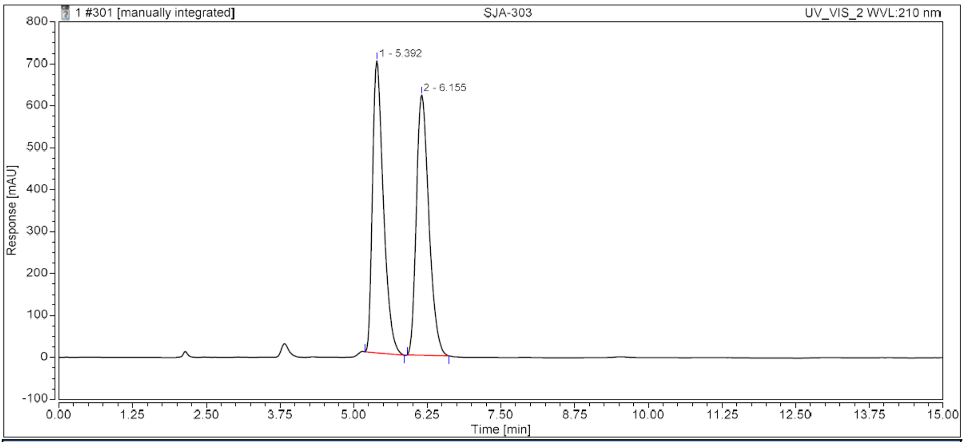

Integration Results							
No.	Peak Name	Retention Time min	Area mAU*min	Height mAU	Relative Area %	Relative Height %	Amount n.a.
1		5.392	149.630	697.177	49.38	52.88	n.a.
2		6.155	153.362	621.342	50.62	47.12	n.a.
**Total:**			**302.992**	**1318.520**	**100.00**	**100.00**	

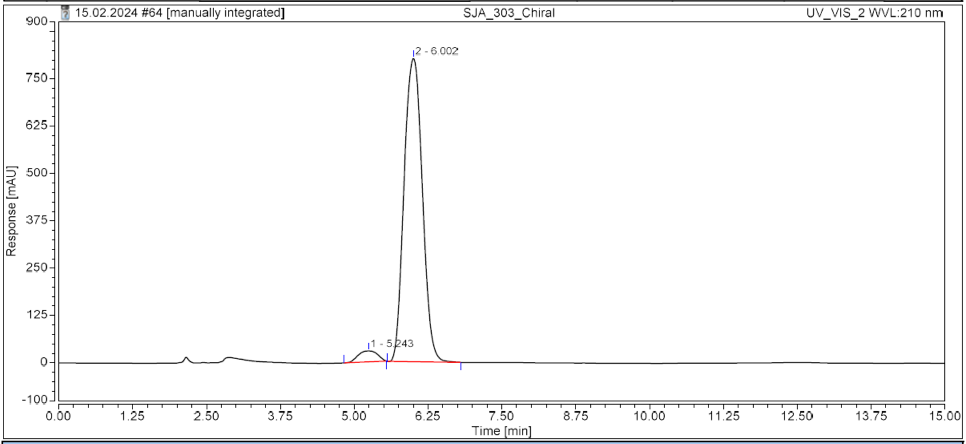

Integration Results							
No.	Peak Name	Retention Time min	Area mAU*min	Height mAU	Relative Area %	Relative Height %	Amount n.a.
1		5.243	11.033	28.839	3.70	3.48	n.a.
2		6.002	287.372	800.985	96.30	96.52	n.a.
**Total:**			**298.406**	**829.824**	**100.00**	**100.00**	

(3*S*,8*S*,9*S*,10*R*,13*R*,14*S*,17*R*)-10,13-dimethyl-17-((*R*)-6-methylheptan-2-yl)-2,3,4,7,8,9,10,11,12,13,14,15,16,17-tetradecahydro-1*H*-cyclopenta[a]phenanthren-3-yl (*S*)-2,2-difluoro-5-oxo-4,5-diphenylpentanoate (9a)

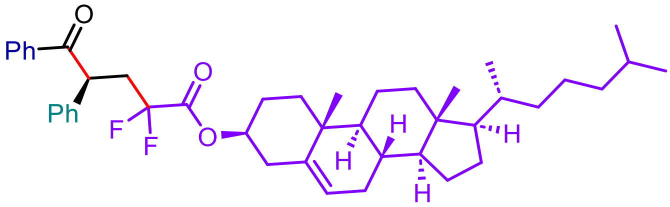

The title compound was synthesized according to the general procedure (**GP 9**), and was obtained after silica gel column chromatography (*n*-pentane : ethylacetate 20:1) as a colorless solid (64 % Yield, 43 mg, 94 % de). **R$_f$** = 0.26 (*n*-pentane : ethylacetate 20:1).

MP: 162-163 C

^{1}H NMR (400 MHz, Chloroform-*d*): δ = 7.99 – 7.92 (m, 2H), 7.52 – 7.46 (m, 1H), 7.42 – 7.36 (m, 2H), 7.32 – 7.25 (m, 4H), 7.25 – 7.18 (m, 1H), 5.42 – 5.30 (m, 1H), 5.00 – 4.93 (m, 1H), 4.64 – 4.52 (m, 1H), 3.40 – 3.19 (m, 1H), 2.59 – 2.43 (m, 1H), 2.39 – 2.31 (m, 1H), 2.30 – 2.15 (m, 1H), 2.06 – 1.62 (m, 6H), 1.56 – 0.84 (m, 32H), 0.68 (s, 3H) ppm.

^{13}C NMR (101 MHz, Chloroform-*d*): δ = 197.3, 163.2 (t, *J* = 32.4 Hz), 138.9 (d, *J* = 1.4 Hz), 138.0, 135.9, 133.2, 129.2, 128.8, 128.6, 128.2, 127.6 (d, *J* = 2.2 Hz), 123.2 (d, *J* = 6.7 Hz), 115.3 (t, *J* = 250.8 Hz), 77.2, 56.6, 56.1, 49.9, 46.8 (q, *J* = 3.5 Hz), 42.3, 39.7, 39.5, 38.1 (t, *J* = 23.2 Hz), 37.5 (d, *J* = 12.2 Hz), 36.8, 36.5, 36.2, 35.8, 31.9, 31.8, 28.2, 28.0, 27.2 (d, *J* = 8.7 Hz), 24.3, 23.8, 22.8, 22.5, 21.0, 19.2, 18.7, 11.8 ppm.

^{19}F NMR (376 MHz, Chloroform-*d*): δ = -104.32 (td, *J* = 16.7, 9.9 Hz) ppm.

HRMS (ESI/QTOF): *m/z*: $[M + Na]^+$ Calcd. for $C_{44}H_{58}F_2NaO_3^+$: 463.2055; Found 463.2063.

IR (ATR): 2937, 2906, 2867, 1761, 1685, 1467, 1448, 1209, 1101, 698 cm^{-1}.

$[\alpha]_D^{20}$ = +19.2 (c = 1.0, $CHCl_3$).

Chiral HPLC: (Chiralpak IC, 1 % *i*PrOH/hexane, 1.0 mL/min, 210 nm): tR (minor) 6.61 min, tR (major) 7.69 min, 97:3 dr.

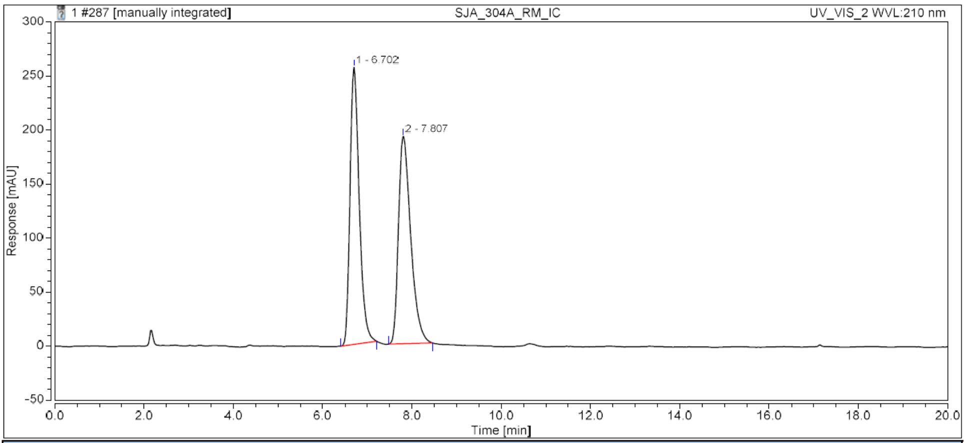

Integration Results

No.	Peak Name	Retention Time min	Area mAU*min	Height mAU	Relative Area %	Relative Height %	Amount n.a.
1		6.702	61.158	256.374	50.10	57.18	n.a.
2		7.807	60.907	191.951	49.90	42.82	n.a.
**Total:**			**122.066**	**448.325**	**100.00**	**100.00**	

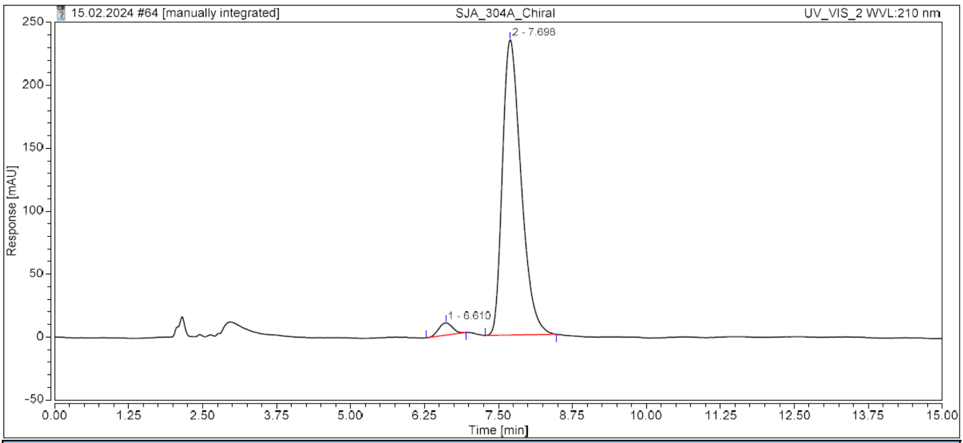

Integration Results

No.	Peak Name	Retention Time min	Area mAU*min	Height mAU	Relative Area %	Relative Height %	Amount n.a.
1		6.610	2.608	9.737	2.95	3.99	n.a.
2		7.698	85.784	234.588	97.05	96.01	n.a.
**Total:**			**88.392**	**244.325**	**100.00**	**100.00**	

(*E*)-3,7-dimethylocta-2,6-dien-1-yl (*S*)-2,2-difluoro-5-oxo-4,5-diphenylpentanoate (9b)

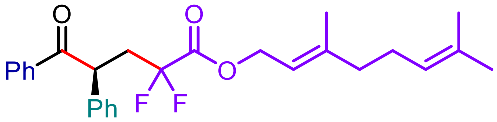

The title compound was synthesized according to the general procedure (**GP 9**), and was obtained after silica gel column chromatography (*n*-pentane : ethylacetate 20:1) as a colorless oil (66 % Yield, 29 mg, 96 % ee). **R$_f$** = 0.22 (*n*-pentane : ethylacetate 20:1).

^{1}H NMR (400 MHz, Chloroform-*d*): δ = 7.99 – 7.92 (m, 2H), 7.52 – 7.46 (m, 1H), 7.42 – 7.36 (m, 2H), 7.31 – 7.26 (m, 4H), 7.25 – 7.18 (m, 1H), 5.28 – 5.21 (m, 1H), 5.09 – 5.03 (m, 1H), 4.96 (dd, *J* = 8.0, 4.9 Hz, 1H), 4.64 (dd, *J* = 12.2, 7.3 Hz, 1H), 4.52 (dd, *J* = 12.2, 7.2 Hz, 1H), 3.37 – 3.20 (m, 1H), 2.61 – 2.46 (m, 1H), 2.13 – 1.98 (m, 4H), 1.70 – 1.66 (m, 6H), 1.60 (s, 3H) ppm.

^{13}C NMR (101 MHz, Chloroform-*d*): δ = 197.4, 163.9 (t, *J* = 32.5 Hz), 144.2, 138.0, 136.0, 133.3, 132.1, 129.3, 128.9, 128.7, 128.3, 127.7, 123.6, 116.8, 115.4 (t, *J* = 250.9 Hz), 63.7, 46.9 (t, *J* = 3.8 Hz), 39.6, 38.3 (t, *J* = 23.3 Hz), 26.2, 25.7, 17.8, 16.6 ppm.

^{19}F NMR (376 MHz, Chloroform-*d*): δ = -104.21 (td, *J* = 16.6, 9.8 Hz) ppm.

HRMS (ESI/QTOF): *m/z*: $[M + Na]^+$ Calcd for $C_{27}H_{30}F_2NaO_3^+$ 463.2055; Found 463.2057.

IR (ATR): 1798, 1765, 1684, 1649, 1291, 1263, 968 cm^{-1}.

$[\alpha]_D^{20}$ = +39.6 (c = 0.8, $CHCl_3$).

Chiral HPLC: (Chiralpak IC, 1 % *i*PrOH/hexane, 1.0 mL/min, 210 nm): tR (minor) 8.04 min, tR (major) 9.49 min, 98:2 er.

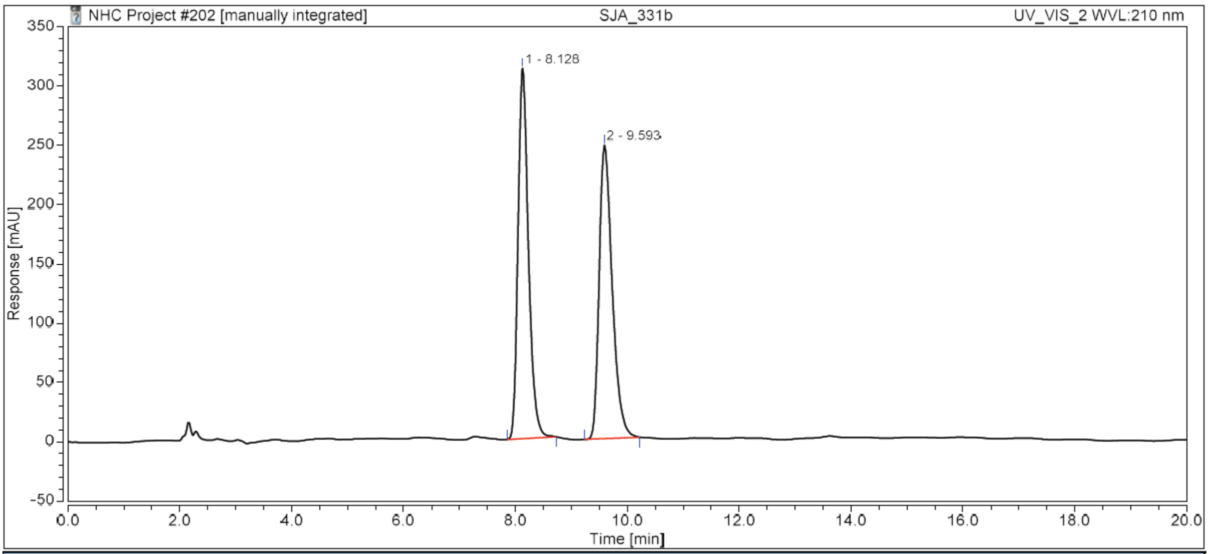

Integration Results

No.	Peak Name	Retention Time min	Area mAU*min	Height mAU	Relative Area %	Relative Height %	Amount n.a.
1		8.128	67.110	312.460	50.20	55.79	n.a.
2		9.593	66.588	247.651	49.80	44.21	n.a.
**Total:**			**133.699**	**560.111**	**100.00**	**100.00**	

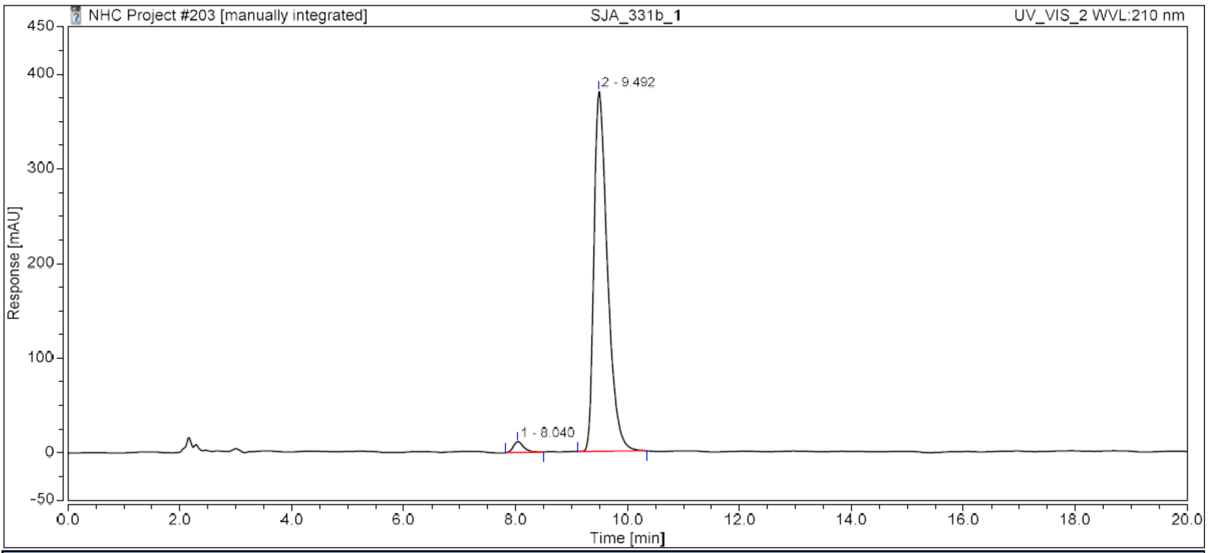

Integration Results							
No.	Peak Name	Retention Time min	Area mAU*min	Height mAU	Relative Area %	Relative Height %	Amount n.a.
1		8.040	2.462	11.506	2.27	2.94	n.a.
2		9.492	106.112	380.021	97.73	97.06	n.a.
**Total:**			**108.574**	**391.526**	**100.00**	**100.00**	

Reaction with linchpin aldehyde

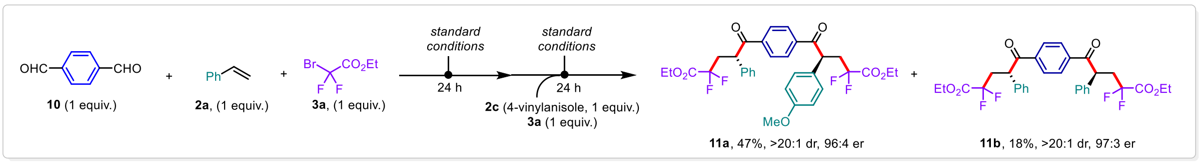

In a nitrogen-filled glove box, an oven-dried 5 mL reaction tube was charged with chiral thiazolium salt **NHC 7** (5 mol%) and 1 mL of dry, degassed methyl *tert*-butyl ether. Terephthalaldehyde **10** (0.1 mmol, 1 eq.), styrene **2a** (0.1 mmol, 1.0 eq.), ethyl difluorobromoacetate **3a** (0.1 mmol, 1 eq.), and Cs_2CO_3 (0.12 mmol, 1.2 eq.) were then added, and the tube was sealed with a crimper. The reaction tube was removed from the glove box, stirred at 60 °C for 24 hours, cooled to room temperature, and returned to the glove box. A second portion of **NHC 7** (5 mol%) was added, followed by 4-vinyl anisole **2c** (0.1 mmol, 1 eq.), ethyl difluorobromoacetate **3a** (0.1 mmol, 1 eq.), and Cs_2CO_3 (0.12 mmol, 1.2 eq.). The tube was again sealed with a crimper, removed from the glove box, and stirred at 60 °C for another 24 hours. The reaction mixture was then filtered through a 4 cm silica gel pad, and the solvent was evaporated under reduced pressure. The crude product mixture was purified by silica gel column chromatography using a pentane/ethyl acetate mixture as the eluent, yielding **11a** as the major product (47%) and **11b** as the minor product (18%), both with high diastereoselectivity and enantioselectivity.

Ethyl (*S*)-5-(4-((*S*)-5-ethoxy-4,4-difluoro-2-(4-methoxyphenyl)-5-oxopentanoyl)phenyl)-2,2-difluoro-5-oxo-4-phenylpentanoate (11a)

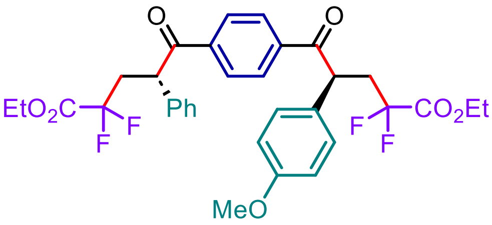

11a was obtained as a coloreless oil in 47% yield (29 mg), d.r. >20:1, 96:4 er. $\mathbf{R_f}$ = 0.22 (*n*-pentane : ethylacetate 9:1).

^{1}H NMR (800 MHz, Chloroform-*d*): δ = 7.95 – 7.90 (m, 4H), 7.29 – 7.26 (m, 3H), 7.24 – 7.20 (m, 2H), 7.15 – 7.11 (m, 2H), 6.82 – 6.77 (m, 2H), 4.88 (dd, *J* = 8.1, 4.8 Hz, 1H), 4.82 (dd, *J* = 8.0, 5.0 Hz, 1H), 4.19 – 4.13 (m, 2H), 4.09 – 4.04 (m, 2H), 3.73 (s, 3H), 3.29 – 3.14 (m, 2H), 2.54 – 2.43 (m, 2H), 1.23 – 1.20 (m, 6H) ppm.
^{13}C NMR (201 MHz, Chloroform-*d*): δ = 196.6, 196.5, 163.76 (t, *J* = 32.6 Hz), 163.72 (t, *J* = 32.2 Hz), 159.2, 139.0, 138.9, 137.1, 129.4, 129.3, 128.9, 128.8, 128.2, 127.9, 115.17 (t, *J* = 250.7 Hz), 115.11 (t, *J* = 250.8 Hz), 114.7, 63.01, 62.9, 55.2, 47.3 (t, *J* = 3.7 Hz), 46.4 (t, *J* = 3.6 Hz), 38.0 (t, *J* = 23.3 Hz), 29.7, 13.7 ppm.
^{19}F NMR (376 MHz, Chloroform-*d*): δ = -103.63 – 104.46 (m), -104.47 – 105.28 (m) ppm.
HRMS (ESI/QTOF): *m/z*: $[M + Na]^+$ Calcd for $C_{33}H_{32}F_4NaO_7^+$ 639.1976; Found 639.1981.
IR (ATR): 1764, 1686, 1511, 1375, 1031, 774 cm^{-1}.
$[\alpha]_D^{20}$ = +19.6 (c = 0.5, $CHCl_3$).
Chiral HPLC: (Chiralpak IA, 10 % *i*PrOH/hexane, 1.0 mL/min, 254 nm): tR (minor) 12.75 min, tR (major) 13.85 min, 96:4 er.

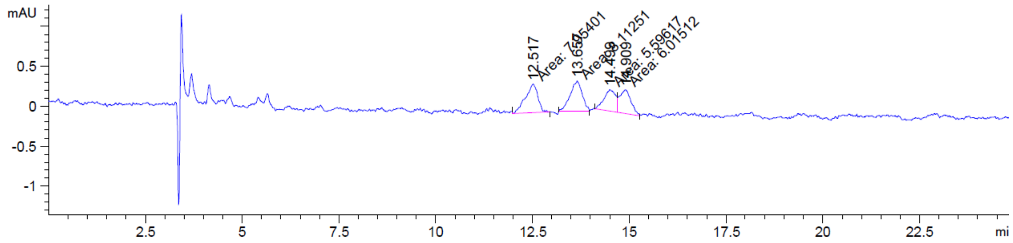

Peak #	RetTime [min]	Type	Width [min]	Area [mAU*s]	Height [mAU]	Area %
1	12.517	MM	0.3723	7.95401	3.56066e-1	28.7378
2	13.657	MM	0.3585	8.11251	3.77188e-1	29.3105
3	14.499	MF	0.3465	5.59617	2.69200e-1	20.2190
4	14.909	FM	0.3337	6.01512	3.00397e-1	21.7326

To reconfirm the identity of the diastereomeric peaks in the HPLC trace of the racemic sample, we performed a control experiment by adding 20 µL of a solution of the enantiopure sample into the racemic mixture. We observed a change in the ratio between the first two peaks, while the ratio of the third and fourth peaks remained the same, confirming that the first and second peaks are enantiomeric.

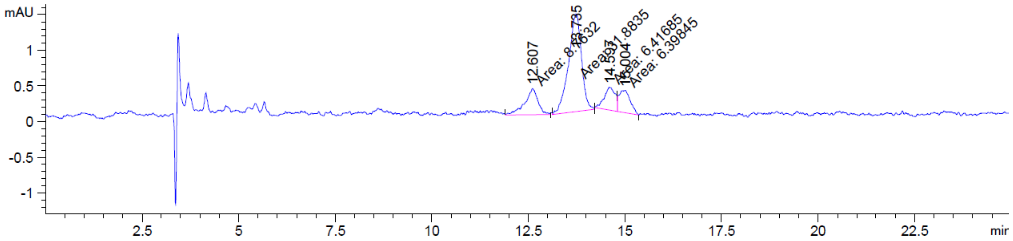

Peak #	RetTime [min]	Type	Width [min]	Area [mAU*s]	Height [mAU]	Area %
1	12.607	MM	0.3959	8.76320	3.68957e-1	16.3915
2	13.735	MM	0.3888	31.88350	1.36692	59.6377
3	14.597	MF	0.3336	6.41685	3.20581e-1	12.0026
4	15.004	FM	0.3372	6.39845	3.16220e-1	11.9682

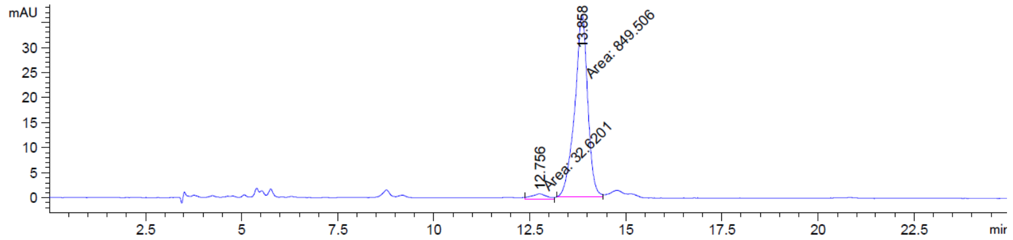

Peak #	RetTime [min]	Type	Width [min]	Area [mAU*s]	Height [mAU]	Area %
1	12.756	MM	0.4868	32.62011	1.11671	3.6979
2	13.858	MM	0.3890	849.50562	36.39898	96.3021

Diethyl 5,5'-(1,4-phenylene)(4*S*,4'*S*)-bis(2,2-difluoro-5-oxo-4-phenylpentanoate) (11b)

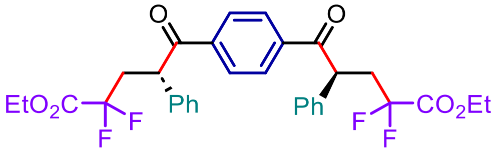

11b was obtained as a colorless oil in 18% yield (10.5 mg), with a d.r. >20:1 and 97:3 e.r. $\mathbf{R_f}$ = 0.28 (n-pentane/ethyl acetate 9:1).

^{1}H NMR (400 MHz, Chloroform-*d*): δ = 7.93 (s, 4H), 7.32 – 7.18 (m, 10H), 4.88 (dd, *J* = 8.1, 4.8 Hz, 2H), 4.16 (dq, *J* = 10.8, 7.1 Hz, 2H), 4.06 (dq, *J* = 10.8, 7.1 Hz, 2H), 3.24 (tdd, *J* = 16.9, 15.0, 8.1 Hz, 2H), 2.49 (tdd, *J* = 17.0, 15.1, 4.8 Hz, 2H), 1.22 (t, *J* = 7.2 Hz, 6H) ppm.

^{13}C NMR (101 MHz, Chloroform-*d*): δ = 196.5, 163.7 (t, *J* = 32.4 Hz), 139.0, 137.1, 129.4, 128.9, 128.2, 127.9, 115.1 (t, *J* = 250.8 Hz), 63.0, 47.3 (t, *J* = 3.8 Hz), 38.0 (t, *J* = 23.4 Hz), 13.7 ppm.

^{19}F NMR (376 MHz, Chloroform-*d*): δ = -104.0 (dt, *J* = 258.7, 16.7 Hz), -104.8 (dt, *J* = 258.7, 16.7 Hz) ppm.

HRMS (ESI/QTOF): *m/z*: [M + Na]$^+$ Calcd for $C_{32}H_{30}F_4NaO_6^+$ 609.1871; Found 609.1867.

IR (ATR): 1760, 1684, 1301, 1260, 1095, 1070, 698 cm^{-1}.

$[\alpha]_D^{20}$ = +24.3 (c = 0.5, $CHCl_3$).

Chiral HPLC: (Chiralpak IC, 5 % *i*PrOH/hexane, 1.0 mL/min, 210 nm): tR (minor) 17.25 min, tR (major) 33.02 min, 97:3 er.

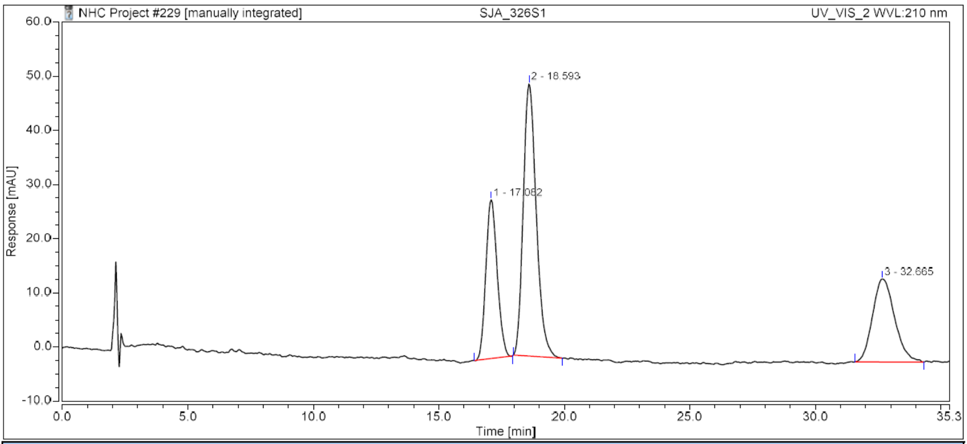

Integration Results

No.	Peak Name	Retention Time min	Area mAU*min	Height mAU	Relative Area %	Relative Height %	Amount n.a.
1		17.082	15.340	29.332	25.10	30.88	n.a.
2		18.593	30.175	50.327	49.37	52.98	n.a.
3		32.665	15.605	15.341	25.53	16.15	n.a.
**Total:**			**61.119**	**95.001**	**100.00**	**100.00**	

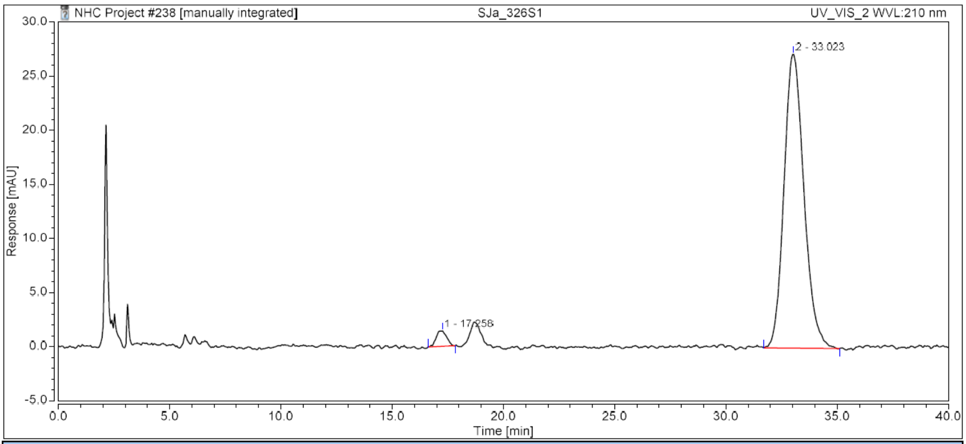

Integration Results

No.	Peak Name	Retention Time min	Area mAU*min	Height mAU	Relative Area %	Relative Height %	Amount n.a.
1		17.258	0.776	1.421	2.67	4.97	n.a.
2		33.023	28.244	27.182	97.33	95.03	n.a.
**Total:**			**29.020**	**28.604**	**100.00**	**100.00**	

Reaction with linchpin olefin

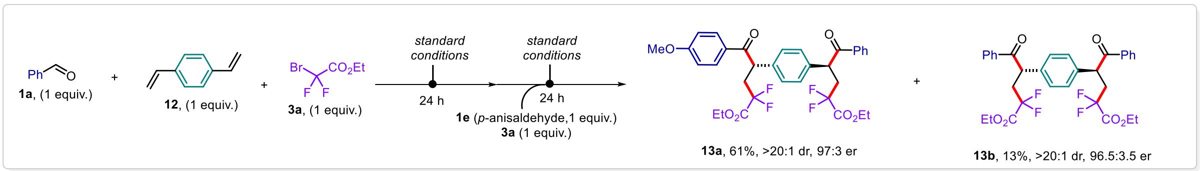

In a nitrogen-filled glove box, an oven-dried 5 mL reaction tube was charged with chiral thiazolium salt **NHC 7** (5 mol%) and 1 mL of dry, degassed methyl *tert*-butyl ether. Benzaldehyde **1a** (0.1 mmol, 1 eq.), 1,4-divinylbenzene **13** (0.1 mmol, 1.0 eq.), ethyl difluorobromoacetate **3a** (0.1 mmol, 1 eq.), and Cs_2CO_3 (0.12 mmol, 1.2 eq.) were then added, and the tube was sealed with a crimper. The reaction tube was removed from the glove box, stirred at 60 °C for 24 hours, cooled to room temperature, and returned to the glove box. A second portion of **NHC 7** (5 mol%) was added, followed by *p*-anisaldehyde **2c** (0.1 mmol, 1 eq.), ethyl difluorobromoacetate **3a** (0.1 mmol, 1 eq.), and Cs_2CO_3 (0.12 mmol, 1.2 eq.). The tube was again sealed with a crimper, removed from the glove box, and stirred at 60 °C for another 24 hours. The reaction mixture was then filtered through a 4 cm silica gel pad, and the solvent was evaporated under reduced pressure. The crude product mixture was purified by silica gel column chromatography using a pentane/ethyl acetate mixture as the eluent, yielding **13a** as the major product (47%) and **13b** as the minor product (18%), both with high diastereoselectivity and enantioselectivity.

Ethyl (*S*)-4-(4-((*S*)-5-ethoxy-4,4-difluoro-1,5-dioxo-1-phenylpentan-2-yl)phenyl)-2,2-difluoro-5-(4-methoxyphenyl)-5-oxopentanoate (13a)

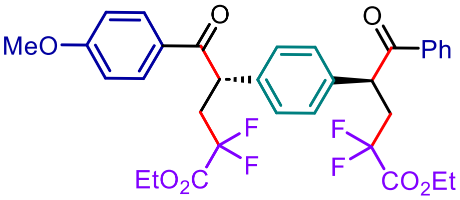

13a was obtained as a colorless oil in 61% yield (37 mg), with a d.r. >20:1 and 97:3 e.r. **R$_f$** = 0.22 (n-pentane/ethyl acetate 9:1).

^{1}H NMR (400 MHz, Chloroform-*d*): δ = 7.94 – 7.87 (m, 4H), 7.54 – 7.45 (m, 1H), 7.42 – 7.35 (m, 2H), 7.22 (s, 4H), 6.88 – 6.82 (m, 2H), 4.89 (dd, *J* = 7.9, 5.1 Hz, 1H), 4.84 (dd, *J* = 7.8, 5.3 Hz, 1H), 4.06 – 3.94 (m, 2H), 3.89 – 3.77 (m, 5H), 3.24 – 3.06 (m, 2H), 2.53 – 2.36 (m, 2H), 1.15 – 1.08 (m, 6H) ppm.

^{13}C NMR (101 MHz, Chloroform-*d*): δ = 197.1, 195.6, 163.8, 163.73 (t, *J* = 32.6 Hz), 163.71 (t, *J* = 32.9 Hz), 137.7, 137.1, 135.7, 133.5, 131.3, 129.1, 128.9, 128.8, 128.6, 115.3 (t, *J* = 250.4 Hz), 115.2 (t, *J* = 250.6 Hz), 114.0, 62.9, 62.8, 55.6, 46.3 (t, *J* = 3.8 Hz), 45.9 (t, *J* = 3.9 Hz), 38.2 (t, *J* = 23.3 Hz), 31.6, 29.8, 13.8 ppm.

^{19}F NMR (377 MHz, Chloroform-*d*): δ = -103.88 (dtd, *J* = 259.4, 16.3, 6.6 Hz), -105.03 (dtd, *J* = 259.4, 16.6, 3.1 Hz).

HRMS (ESI/QTOF): *m/z*: $[M + Na]^+$ Calcd for $C_{33}H_{32}F_4NaO_7^+$ 639.1976; Found 639.1982.

IR (ATR): 1767, 1679, 1599, 1332, 1307, 1212, 1088 cm^{-1}.

$[\alpha]_D^{20}$ = +32.3 (c = 0.5, $CHCl_3$).

Chiral HPLC: (Chiralpak IF, 10 % *i*PrOH/hexane, 1.0 mL/min, 254 nm): tR (minor) 20.78 min, tR (major) 22.08 min, 97:3 er.

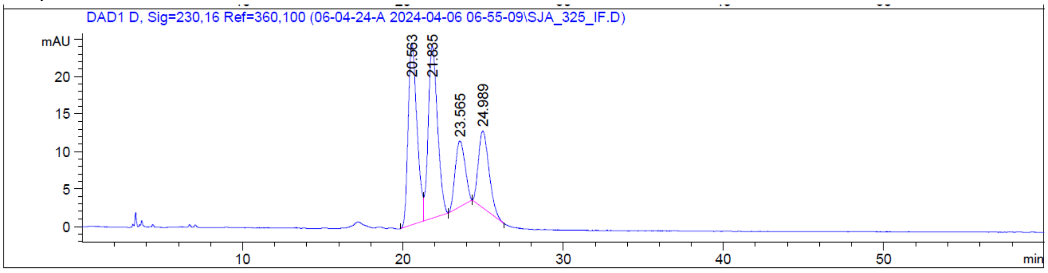

Peak #	RetTime [min]	Type	Width [min]	Area [mAU*s]	Height [mAU]	Area %
1	20.563	BV	0.5837	926.02783	24.23875	34.2777
2	21.835	VB	0.6088	927.30817	22.97744	34.3251
3	23.565	BB	0.6342	376.10696	8.77266	13.9219
4	24.989	BB	0.6341	472.10101	10.23572	17.4752

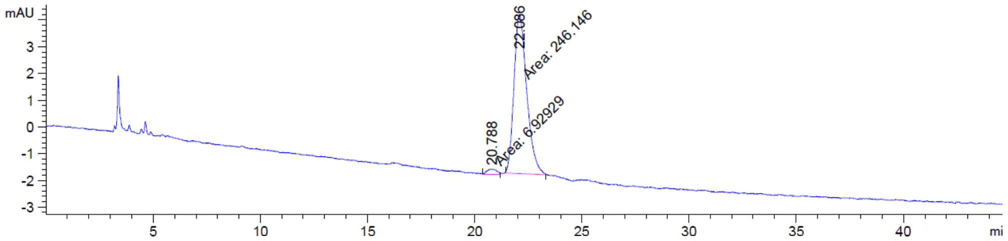

Peak #	RetTime [min]	Type	Width [min]	Area [mAU*s]	Height [mAU]	Area %
1	20.788	MM	0.5494	6.92929	2.10200e-1	2.7380
2	22.086	MM	0.6900	246.14597	5.94572	97.2620

Diethyl 4,4'-(1,4-phenylene)(4*S*,4'*S*)-bis(2,2-difluoro-5-oxo-5-phenylpentanoate) (13b)

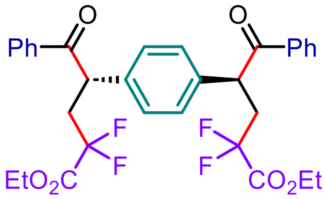

13b was obtained as a colorless oil in 13% yield (8 mg), with a d.r. >20:1 and 96.5:3.5 e.r. $\mathbf{R_f}$ = 0.28 (n-pentane : ethylacetate 9:1).

^{1}H NMR (400 MHz, Chloroform-*d*): δ = 7.95 – 7.88 (m, 4H), 7.54 – 7.47 (m, 2H), 7.43 – 7.36 (m, 4H), 7.23 (s, 4H), 4.90 (dd, *J* = 8.1, 4.8 Hz, 2H), 4.03 (dq, *J* = 10.8, 7.1 Hz, 2H), 3.93 (dq, *J* = 10.8, 7.2 Hz, 2H), 3.34 – 3.09 (m, 2H), 2.53 – 2.36 (m, 2H), 1.14 (t, *J* = 7.2 Hz, 6H) ppm.

^{13}C NMR (101 MHz, Chloroform-*d*): δ = 197.0, 163.6 (t, *J* = 32.4 Hz), 137.2, 135.6, 133.3, 129.0, 128.8, 128.6, 115.1 (t, *J* = 250.8 Hz), 62.8, 46.1 (t, *J* = 3.8 Hz), 38.1 (t, *J* = 23.4 Hz), 13.6 ppm.

^{19}F NMR (376 MHz, Chloroform-*d*):-104.02 (dt, *J* = 259.7, 16.3 Hz), -104.97 (dt, *J* = 259.6, 16.6 Hz).

HRMS (ESI/QTOF): *m/z*: $[M + Na]^+$ Calcd for $C_{32}H_{30}F_4NaO_6^+$ 609.1871; Found 609.1887.

$[\alpha]_D^{20}$ = +41.6 (c = 0.5, $CHCl_3$).

IR (ATR): 1762, 1680, 1297, 1085, 1019, 850, 688 cm^{-1}.

Chiral HPLC: (Chiralpak IC, 6 % *i*PrOH/hexane, 1.0 mL/min, 210 nm): tR (minor) 13.06 min, tR (major) 22.53 min, 96.5:3.5 er.

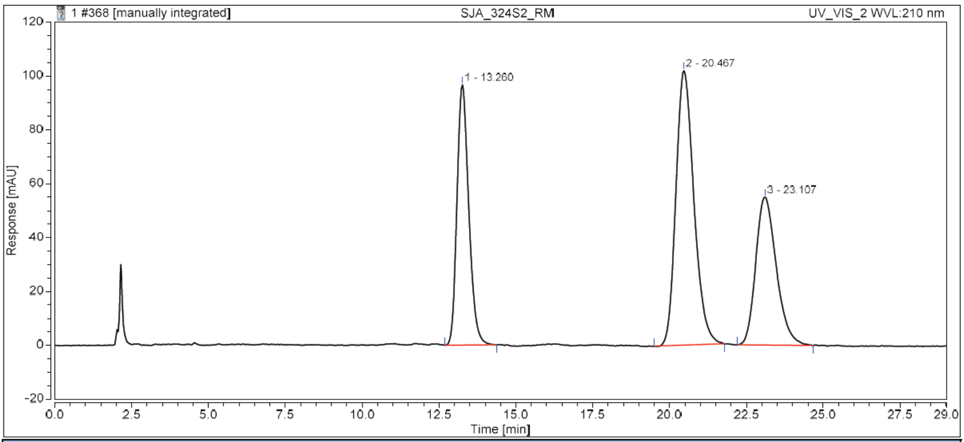

Integration Results

No.	Peak Name	Retention Time min	Area mAU*min	Height mAU	Relative Area %	Relative Height %	Amount n.a.
1		13.260	42.875	96.733	27.73	38.13	n.a.
2		20.467	69.019	101.993	44.64	40.20	n.a.
3		23.107	42.701	54.998	27.62	21.68	n.a.
**Total:**			**154.596**	**253.725**	**100.00**	**100.00**	

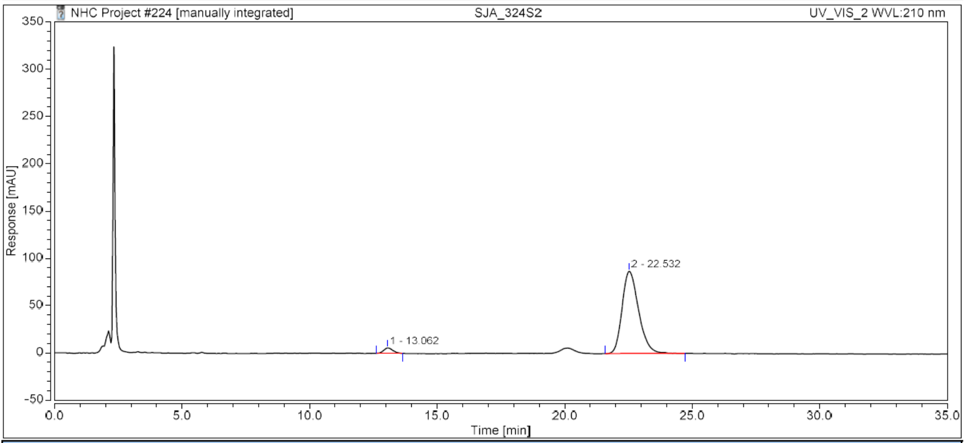

Integration Results

No.	Peak Name	Retention Time min	Area mAU*min	Height mAU	Relative Area %	Relative Height %	Amount n.a.
1		13.062	2.320	5.555	3.43	5.99	n.a.
2		22.532	65.242	87.174	96.57	94.01	n.a.
**Total:**			**67.562**	**92.729**	**100.00**	**100.00**	

Scale up experiment

In a nitrogen-filled glove box, an oven-dried 50 mL screw cap reaction tube was charged with chiral thiazolium salt NHC7 (90 mg, 5 mol%). Then, 20 mL of dry and degassed methyl *tert*-butyl ether (MTBE) was added. Subsequently, aldehyde **1d** (463 mg, 2 mmol, 1 eq.), styrene **2a** (312 mg, 3 mmol, 1.5 eq.), difluoroalkyl bromide **3a** (807 mg, 4 mmol, 2 eq.), and Cs_2CO_3 (977.5 mg, 3 mmol, 1.5 eq.) were added to the reaction tube, which was then screwed tightly. The reaction tube was removed from the glove box and stirred at 60 °C for 24 hours. The reaction mixture was then filtered through a short pad of silica gel (4 cm), and the solvent was evaporated under reduced pressure. The crude product mixture was purified by silica gel column chromatography using *n*-pentane : ethylacetate (30:1) mixture as eluent to obtain chiral ketone **4d** as colorless solid in 81% yield (742 mg) and 97:3 er.

(1*S*,2*S*)-4,4-difluoro-1-(4-iodophenyl)-2-phenylpentane-1,5-diol (14)*

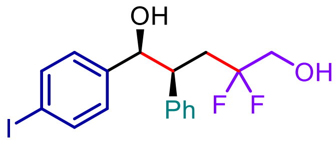

A suspension of $NaBH_4$ (0.1 mmol, 1 eq.) in THF was stirred at room temperature, and a solution of enantiopure ester **4d** (45.8 mg, 0.1 mmol, 96% ee) in dry THF (0.35 mL) was added dropwise over 5 minutes. The reaction mixture was then stirred at room temperature for 2 hours. It was hydrolyzed

with water, acidified with 2 N HCl, and extracted with ether. The organic phase was washed with sodium bicarbonate solution, followed by brine, and dried over sodium sulfate. After evaporation of the solvent under reduced pressure, the crude mixture was purified by silica gel column chromatography to obtain diol **14** as a colorless solid (86% yield, d.r. >20:1, 96:4 e.r.). **R$_f$** = 0.20 (*n*-pentane : ethylacetate 4:1)

** The absolute configuration of this compound was determined based on the absolute configuration of **4d** and the X-ray crystallography of the racemic sample of **14**.*

MP: 171-172 °C

^{1}H NMR (400 MHz, Chloroform-*d*): δ = 7.64 – 7.59 (m, 2H), 7.33 – 7.23 (m, 3H), 7.18 – 7.13 (m, 2H), 6.94 – 6.89 (m, 2H), 4.82 (dd, *J* = 6.8, 2.4 Hz, 1H), 3.56 – 3.30 (m, 2H), 3.26 – 3.18 (m, 1H), 2.51 – 2.20 (m, 2H), 1.95 – 1.90 (m, 1H), 1.63 (t, *J* = 7.1 Hz, 1H) ppm.

^{13}C NMR (101 MHz, Chloroform-*d*): δ = 141.3, 139.1, 137.3, 128.9, 128.69, 128.61, 127.5, 122.9 (t, *J* = 242.9 Hz), 93.4, 64.3 (d, *J* = 30.8 Hz), 64.0 (d, *J* = 31.1 Hz), 35.3 (t, *J* = 23.7 Hz) ppm.

^{19}F NMR (376 MHz, Chloroform-*d*) δ -103.61 (dddt, *J* = 251.4, 18.3, 15.9, 12.9 Hz), -107.28 (dddt, *J* = 251.7, 19.4, 15.8, 11.8 Hz) ppm.

HRMS (ESI/QTOF): *m/z*: $[M + Na]^+$ Calcd for $C_{17}H_{17}F_2INaO_2^+$ 441.0134; Found 441.0146.

IR (ATR): 3401, 3391, 1701, 1611, 1113, 1060, 676 cm^{-1}.

$[\alpha]_D^{20}$ = -59.7 (c = 0.5, $CHCl_3$).

Chiral HPLC: (Chiralpak IC, 5 % *i*PrOH/hexane, 1.0 mL/min, 254 nm): tR (minor) 11.86 min, tR (major) 13.44 min, 96:4 er.

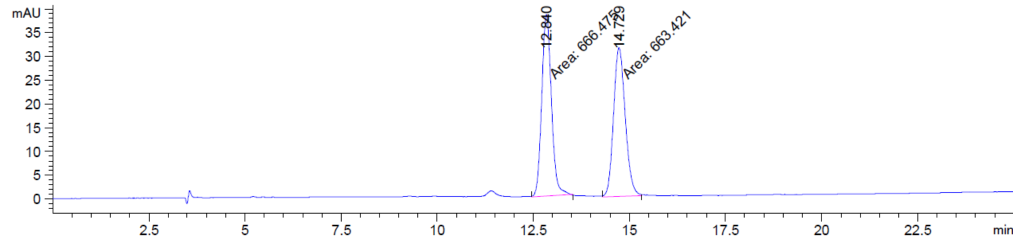

Peak #	RetTime [min]	Type	Width [min]	Area [mAU*s]	Height [mAU]	Area %
1	12.840	MM	0.2909	666.47473	38.18108	50.1148
2	14.729	MM	0.3527	663.42108	31.34701	49.8852

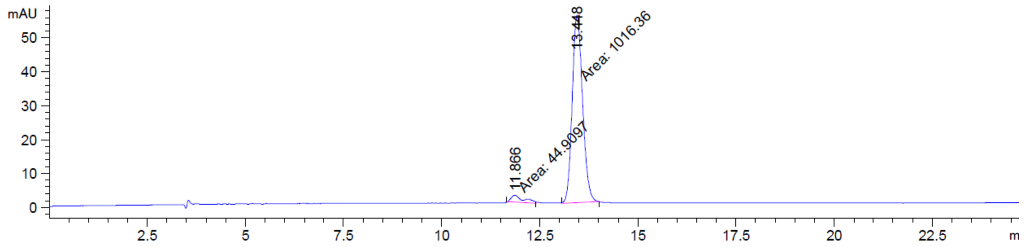

Peak #	RetTime [min]	Type	Width [min]	Area [mAU*s]	Height [mAU]	Area %
1	11.866	MM	0.3660	44.90971	2.04492	4.2317
2	13.448	MM	0.3065	1016.36304	55.27602	95.7683

Supplementary Experiments for Revision

(*S*)-4,4-Dimethyl-1,2-diphenylpentan-1-one (27)

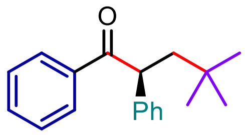

The title compound was synthesized according to the general procedure (**GP 9**) using redox active ester **24** as the radical precursor, and was obtained after silica gel column chromatography (*n*-pentane : ethylacetate 40:1 – 20:1) as a colorless solid (11 % Yield, 2.9 mg, 92 % ee). $\mathbf{R_f}$ = 0.36 (*n*-pentane : ethylacetate 40:1).

Note: To improve the yield, we conducted the reaction under conditions similar to those reported by Ohmiya et al.[22] *in the racemic version of this molecule. Benzaldehyde (0.1 mmol, 1 eq.), styrene (0.2 mmol, 2 eq.), redox-active ester* ***24****(0.15 mmol, 1.5 eq.), 5 mol% NHC 7, and 10 mol%* Cs_2CO_3 *were used in 0.2 mL of solvent at 80 °C for 6 hours.*

In TBME (0.2 mL), the product was obtained in 16% yield with a 92:8 enantiomeric ratio.

In DMSO (0.2 mL), the product was obtained in 72% yield with a 50:50 enantiomeric ratio.

^{1}**H NMR** (400 MHz, Chloroform-*d*): δ = 8.04 – 7.97 (m, 2H), 7.53 – 7.47 (m, 1H), 7.44 – 7.39 (m, 2H), 7.34 – 7.30 (m, 2H), 7.30 – 7.24 (m, 2H), 7.20 – 7.15 (m, 1H), 4.73 (dd, *J* = 8.9, 3.3 Hz, 1H), 2.63 (dd, *J* = 14.0, 8.9 Hz, 1H), 1.59 (dd, *J* = 14.1, 3.4 Hz, 1H), 0.89 (s, 9H) ppm.

^{13}**C NMR** (101 MHz, Chloroform-*d*): δ = 200.1, 141.2, 137.1, 132.9, 129.0, 128.7, 128.7, 128.2, 126.8, 49.8, 47.7, 31.3, 29.9 ppm.

HRMS (ESI/QTOF): m/z: $[M + Na]^+$ Calcd for $C_{19}H_{22}NaO^+$ 289.1563; Found 289.1569.

IR (ATR): 2953, 1673, 1596, 1447, 1178, 697 cm^{-1}.

$[\alpha]_D^{20}$ = +47.2 (c = 0.1, $CHCl_3$).

Chiral HPLC: (Chiralpak IC, 0.3 % *i*PrOH/hexane, 1.0 mL/min, 210 nm): tR (minor) 4.63 min, tR (major) 5.74 min, 96:4 *er*.

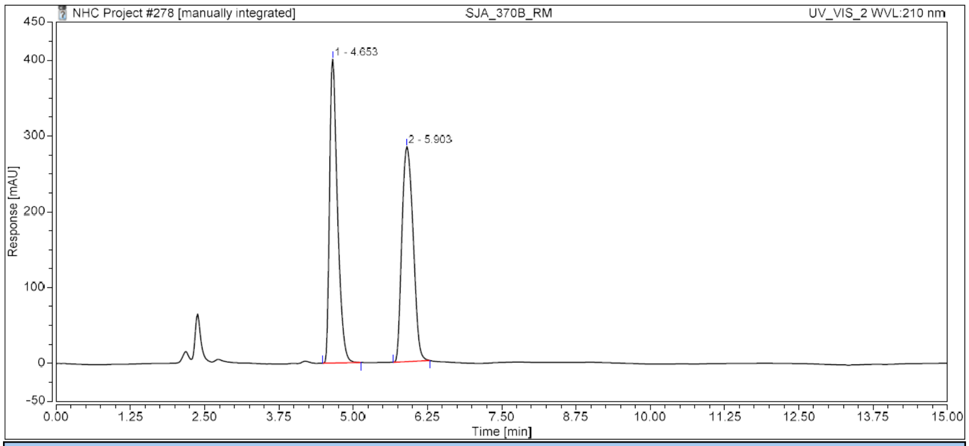

Integration Results

No.	Peak Name	Retention Time min	Area mAU*min	Height mAU	Relative Area %	Relative Height %	Amount n.a.
1		4.653	61.952	400.652	49.87	58.55	n.a.
2		5.903	62.281	283.618	50.13	41.45	n.a.
**Total:**			**124.233**	**684.271**	**100.00**	**100.00**	

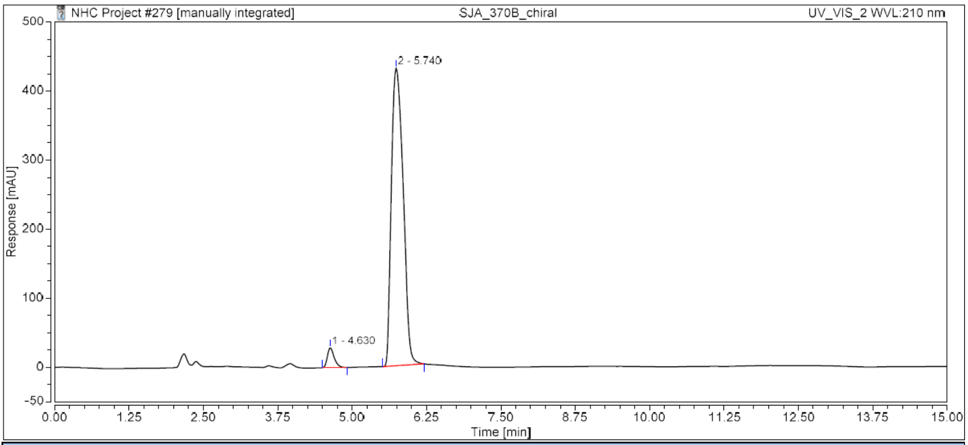

Integration Results							
No.	Peak Name	Retention Time min	Area mAU*min	Height mAU	Relative Area %	Relative Height %	Amount n.a.
1		4.630	3.816	28.772	3.74	6.25	n.a.
2		5.740	98.247	431.546	96.26	93.75	n.a.
Total:			102.063	460.318	100.00	100.00	

(*S*)-1,2-Diphenylpropan-1-one (28)

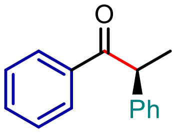

The title compound was synthesized according to the general procedure (**GP 9**) using redox active ester **25** as the radical precursor in the abscence of styrene. Product **28** was obtained after silica gel column chromatography (*n*-pentane : ethylacetate 40:1 – 20:1) as a colorless liquid (7 % Yield, 1,5 mg, 96:4 er). $\mathbf{R_f}$ = 0.34 (*n*-pentane : ethylacetate 40:1).

Note: To improve the yield, we conducted the reaction under conditions similar to those reported by Ohmiya et al.[23] *in the racemic version of this molecule. Benzaldehyde (0.15 mmol, 1.5 eq.), redox-active ester* ***25*** *(0.1 mmol, 1.0 eq.), 10 mol% NHC 7, and 25mol%* Cs_2CO_3 *were used in 0.2 mL of solvent at 60 °C for 6 hours.*

In TBME (0.2 mL), the product was obtained in 12% yield with a 96:4 enantiomeric ratio.

In DMSO (0.2 mL), the product was obtained in 61% yield with a 50:50 enantiomeric ratio.

^{1}**H NMR** (400 MHz, Chloroform-*d*): δ = 0 7.97 – 7.93 (m, 1H), 7.50 – 7.44 (m, 1H), 7.41 – 7.35 (m, 1H), 7.31 – 7.27 (m, 2H), 7.24 – 7.17 (m, 1H), 4.69 (q, *J* = 6.9 Hz, 1H), 1.54 (d, *J* = 6.9 Hz, 2H) ppm.

^{13}**C NMR** (101 MHz, Chloroform-*d*): δ = 200.4, 141.6, 136.6, 132.9, 129.1, 128.9, 128.6, 127.9, 127.0, 48.0, 19.6 ppm.

HRMS (ESI/QTOF): m/z: $[M + Na]^+$ Calcd for $C_{15}H_{14}NaO^+$ 233.0937; Found 233.0939.

IR (ATR): 1681, 1596, 1491, 1221, 951, 697 cm^{-1}.

The spectral data is in accordance with the literature.[24]

Chiral HPLC: (Chiralpak IC, 1 % *i*PrOH/hexane, 1.0 mL/min, 254 nm): tR (major) 6.51 min, tR (minor) 7.84 min, 96:4 *er*.

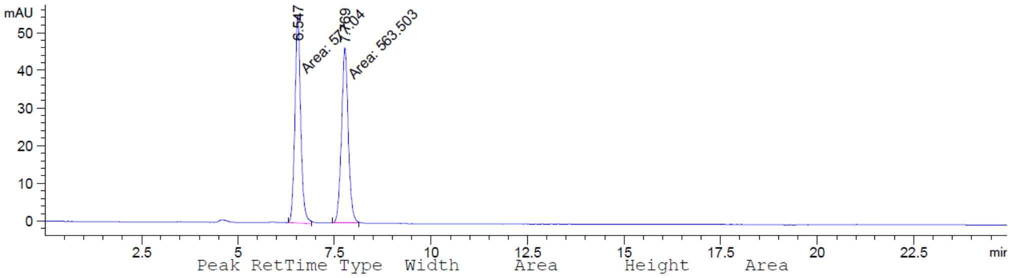

Peak #	RetTime [min]	Type	Width [min]	Area [mAU*s]	Height [mAU]	Area %
1	6.547	MM	0.1743	577.03967	55.16570	50.5934
2	7.769	MM	0.2022	563.50305	46.45218	49.4066

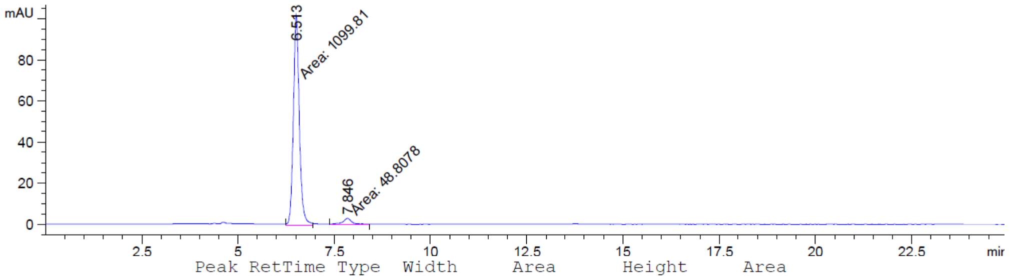

Peak #	RetTime [min]	Type	Width [min]	Area [mAU*s]	Height [mAU]	Area %
1	6.513	MM	0.1794	1099.81201	102.19647	95.7507
2	7.846	MM	0.2798	48.80782	2.90738	4.2493

*A control experiment, where enantiopure ketone **28** (92% ee) was treated with 5 mol% Cs_2CO_3 in DMSO at 40 °C for 60 minutes, demonstrated complete racemization, explaining the loss of enantioselectivity in DMSO.*

Methyl (*S*)-2-benzyl-3-oxo-3-phenylpropanoate (29)

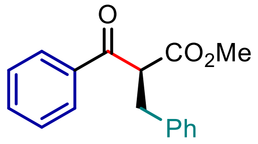

To synthesize this compound, we initially used Katritzky Salt **26** as a radical precursor and conducted the reaction under our optimized conditions, with slight modifications. The reaction conditions were as follows: benzaldehyde (0.1 mmol, 1 eq.), Katritzky Salt **26** (0.2 mmol, 2 eq.), NHC 7 (5 mol%), and Cs_2CO_3 (15 mol%) in 1 mL of TBME under a nitrogen atmosphere, stirred at 60 °C for 24 hours. However, no product formation was observed under these conditions, and partial decomposition of Katritzky Salt xx was detected.

Next, we carried out the reaction in DMSO and DCM, following the literature procedure for the racemic reaction by Hong et al.,[16] using our chiral NHC 7. The reaction conditions were as follows: benzaldehyde (0.15 mmol, 1.5 eq.), Katritzky Salt **26** (0.1 mmol, 1 eq.), NHC 7 (10 mol%), and Cs_2CO_3 (25 mol%) in 1 mL DMSO or DCM, stirred at room temperature for 24 hours.

In DMSO, the product was obtained after silica gel column chromatography (*n*-pentane : ethylacetate 20:1 – 9:1) as a colorless oil with a 57% (15 mg) yield as a racemic mixture (50:50 er). **R_f** = 0.22 (*n*-pentane : ethylacetate 20:1). *No enantioselectivity was observed, which we believe is due to the epimerization of the enantiopure product xx in DMSO in the presence of a catalytic amount of Cs_2CO_3, as the α-proton of the β-keto ester is acidic.*

A similar observation was made in DCM, where the product was obtained with a 32% (8.5 mg) yield as a racemic mixture (50:50 er).

^{1}H NMR (400 MHz, Chloroform-*d*): δ = 8.01 – 7.86 (m, 2H), 7.59 – 7.52 (m, 1H), 7.47 – 7.40 (m, 2H), 7.28 – 7.14 (m, 5H), 4.65 (t, *J* = 7.3 Hz, 1H), 3.64 (s, 3H), 3.39 – 3.26 (m, 2H) ppm.

^{13}C NMR (101 MHz, Chloroform-*d*): δ = 194.4, 169.7, 138.3, 136.1, 133.6, 128.9, 128.7, 128.6, 128.5, 126.6, 55.9, 52.5, 34.8 ppm.

HRMS (ESI/QTOF): m/z: $[M + Na]^+$ Calcd for $C_{17}H_{16}NaO_3^+$ 291.0992; Found 291.1000.

IR (ATR): 1738, 1683, 1448, 1230, 1151, 697 cm^{-1}.

$[\alpha]_D^{20}$ = 0 (c = 0.5, $CHCl_3$).

Chiral HPLC: (Chiralpak IC, 3 % *i*PrOH/hexane, 1.0 mL/min, 254 nm): tR (1st) 18.6 min, tR (2nd) 21.4 min, 50:50 *er.*

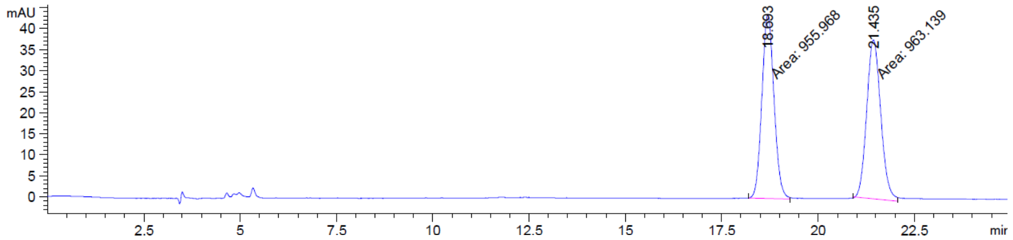

Peak #	RetTime [min]	Type	Width [min]	Area [mAU*s]	Height [mAU]	Area %
1	18.693	MM	0.3639	955.96783	43.78780	49.8132
2	21.435	MM	0.4241	963.13928	37.84989	50.1868

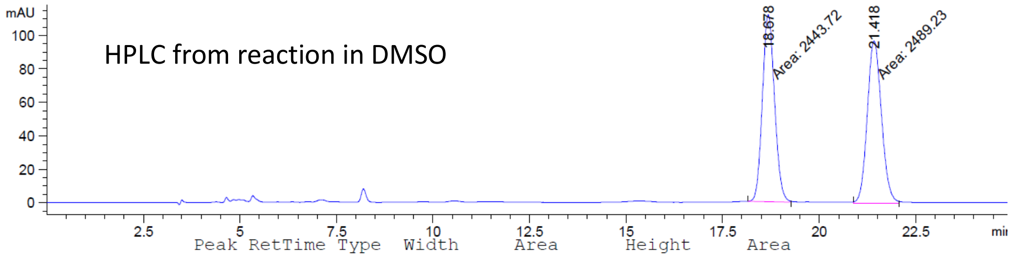

Peak #	RetTime [min]	Type	Width [min]	Area [mAU*s]	Height [mAU]	Area %
1	18.678	MM	0.3640	2443.71777	111.89515	49.5387
2	21.418	MM	0.4268	2489.22607	97.20039	50.4613

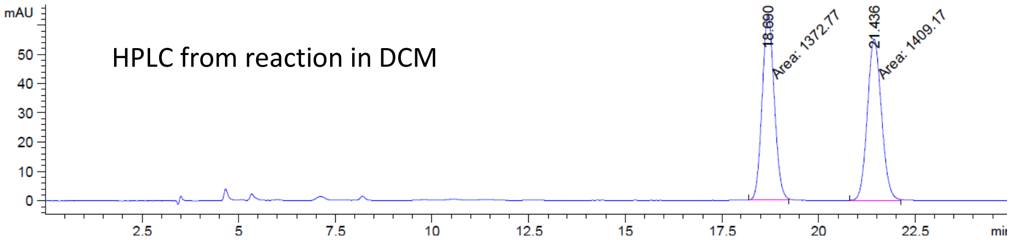

Peak #	RetTime [min]	Type	Width [min]	Area [mAU*s]	Height [mAU]	Area %
1	18.690	MM	0.3612	1372.76624	63.34964	49.3458
2	21.436	MM	0.4254	1409.16626	55.21007	50.6542

Control Experiment with Radical-Clock-Type Olefin

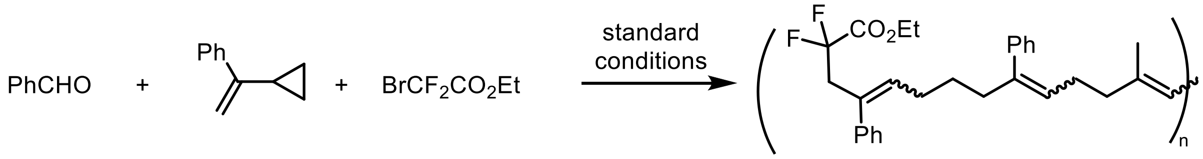

The reaction was performed according to the general procedure (**GP 9**). No dicarbofunctionalization product was observed by mass spectrometry or NMR spectroscopy. A product (5 mg) was isolated, and ^{1}H NMR analysis suggested it was likely a cyclopropane ring-opening radical polymerization product, supporting the involvement of a radical pathway.

X-ray Christallographic data

Methyl (*S*)-4-(5-ethoxy-4,4-difluoro-5-oxo-2-phenylpentanoyl)benzoate (4f)

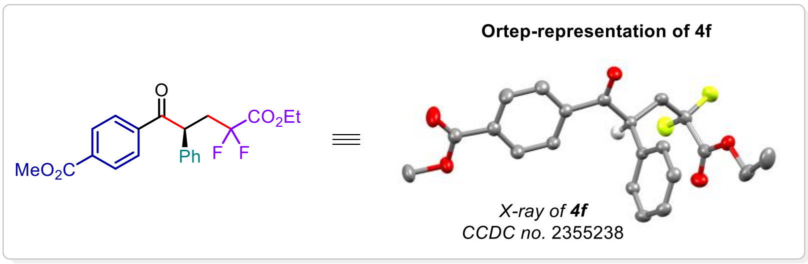

Experimental: Single clear pale colourless needle-shaped crystals of **4f** were used as supplied. A suitable crystal with dimensions 0.49 × 0.04 × 0.04 mm^3 was selected and mounted on a XtaLAB Synergy R, DW system, HyPix-Arc 150 diffractometer. The crystal was kept at a steady T = 140.00(10) K during data collection. The structure was solved with the ShelXT (Sheldrick, 2015)[25] solution program using dual methods and by using Olex2 1.5 (Dolomanov et al., 2009)[26,27] as the graphical interface. The model was refined with ShelXL 2019/3 (Sheldrick, 2015) using full matrix least squares minimisation on $\boldsymbol{F^2}$**.**

Crystal Data. $C_{21}H_{20}F_2O_5$, M_r = 390.37, monoclinic, $P2_1$ (No. 4), a = 5.80743(6) Å, b = 39.0780(5) Å, c = 16.7030(2) Å, β = 91.6904(11)°, $\alpha = \gamma$ = 90°, V = 3788.98(8) $Å^3$, T = 140.00(10) K, Z = 8, Z' = 4, μ(Cu K_α) = 0.933, 65547 reflections measured, 14751 unique (R_{int} = 0.0386) which were used in all calculations. The final wR_2 was 0.0956 (all data) and R_1 was 0.0380 (I≥2 σ(I)).

Compound	4f
Formula	$C_{21}H_{20}F_2O_5$
$D_{calc.}$/ g cm^{-3}	1.369
μ/mm^{-1}	0.933
Formula Weight	390.37
Colour	clear pale colourless
Shape	needle
Size/mm^3	0.49×0.04×0.04
T/K	140.00(10)
Crystal System	monoclinic
Flack Parameter	-0.05(4)
Hooft Parameter	-0.05(4)
Space Group	$P2_1$
a/Å	5.80743(6)
b/Å	39.0780(5)
c/Å	16.7030(2)
α/°	90
β/°	91.6904(11)
γ/°	90
V/Å^3	3788.98(8)
Z	8
Z'	4
Wavelength/Å	1.54184
Radiation type	Cu K$_\alpha$
Θ_{min}/°	2.261
Θ_{max}/°	74.241
Measured Refl's.	65547
Indep't Refl's	14751
Refl's I≥2 σ(I)	12101
R_{int}	0.0386
Parameters	1058
Restraints	107
Largest Peak	0.188
Deepest Hole	-0.192
GooF	1.018
wR_2 (all data)	0.0956
wR_2	0.0893
R_1 (all data)	0.0515
R_1	0.0380
CCDC number	**2355238**

Structure Quality Indicators

**Reflections:**	d min (CuKα) 2Θ=148.5° 0.80	I/σ(I) 28.0	Rint m=4.45 3.86%	Full 135.4° 97% to 148.5° 100
**Refinement:**	Shift 0.001	Max Peak 0.2	Min Peak -0.2	GooF 1.018
				Hooft -.05(4)

A clear pale colourless needle-shaped crystal with dimensions 0.49 × 0.04 × 0.04 mm^3 was mounted. Data were collected using a XtaLAB Synergy R, DW system, HyPix-Arc 150 diffractometer operating at T = 140.00(10) K.

Data were measured using ω scans with Cu K_α radiation. The diffraction pattern was indexed and the total number of runs and images was based on the strategy calculation from the program CrysAlisPro system (CCD 43.119a 64-bit (release 08-04-2024)). The maximum resolution that was achieved was Θ = 74.241° (0.80 Å).

The unit cell was refined using CrysAlisPro 1.171.43.119a (Rigaku OD, 2024) on 21362 reflections, 33% of the observed reflections.

Data reduction, scaling and absorption corrections were performed using CrysAlisPro 1.171.43.119a (Rigaku OD, 2024).[28] The final completeness is 100.00 % out to 74.241° in Θ. A gaussian absorption correction was performed using CrysAlisPro 1.171.43.119a (Rigaku Oxford Diffraction, 2024). The numerical absorption correction was based on gaussian integration over a multifaceted crystal model. The empirical absorption correction was done using spherical harmonics, implemented in SCALE3 ABSPACK scaling algorithm. The absorption coefficient μ of this crystal is 0.933 mm^{-1} at this wavelength (λ = 1.54184Å) and the minimum and maximum transmissions are 0.755 and 1.000.

The structure was solved and the space group $P2_1$ (# 4) determined by the ShelXT (Sheldrick, 2015) structure solution program using dual methods and refined by full matrix least squares minimisation on $\boldsymbol{F}^2$ using version 2019/3 of ShelXL (Sheldrick, 2015). All non-hydrogen atoms were refined anisotropically. Hydrogen atom positions were calculated geometrically and refined using the riding model.

The value of Z' is 4. The moiety formula is C21 H20 F2 O5.

The Flack parameter was refined to -0.05(4). Determination of absolute structure using Bayesian statistics on Bijvoet differences using the Olex2 results in -0.05(4). The chiral atoms in this structure are: C8(S), C29(S), C50(S), C71(S). Note: The Flack parameter is used to determine chirality of the crystal studied, the value should be near 0, a value of 1 means that the stereochemistry is wrong and the model should be inverted. A value of 0.5 means that the crystal consists of a racemic mixture of the two enantiomers.

Ortep-represention of (*S*)-**4f** (thermal ellipsoids set at 50% probability). CCDC **2355238** contains the crystallographic data for (*S*)-**4f**. These data can be obtained free of charge from The Cambridge Crystallographic Data Centre via www.ccdc.cam.ac.uk/data_request/cif.

Datablock: sja-285f

Bond precision: C-C = 0.0041 A Wavelength=1.54184

Cell:	a=5.80743(6)	b=39.0780(5)	c=16.7030(2)
	alpha=90	beta=91.6904(11)	gamma=90
Temperature:	140 K		

	Calculated	Reported
Volume	3788.98(8)	3788.98(8)
Space group	P 21	P 1 21 1
Hall group	P 2yb	P 2yb
Moiety formula	C21 H20 F2 O5	C21 H20 F2 O5
Sum formula	C21 H20 F2 O5	C21 H20 F2 O5
Mr	390.37	390.37
Dx,g cm-3	1.369	1.369
Z	8	8
Mu (mm-1)	0.933	0.933
F000	1632.0	1632.0
F000'	1638.00	
h,k,lmax	7,48,20	7,48,20
Nref	15451[7845]	14751
Tmin,Tmax	0.954,0.966	0.755,1.000
Tmin'	0.635	

Correction method= # Reported T Limits: Tmin=0.755 Tmax=1.000
AbsCorr = GAUSSIAN

Data completeness= 1.88/0.95 Theta(max)= 74.241

R(reflections)= 0.0380(12101) wR2(reflections)= 0.0956(14751)

S = 1.018 Npar= 1058

4,4-Difluoro-1-(4-iodophenyl)-2-phenylpentane-1,5-diol [(*rac*)-14]

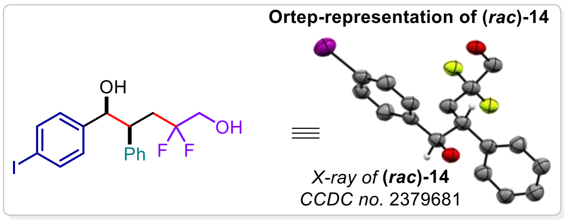

Experimental. Single clear pale colourless irregular-shaped crystals of **(*rac*)-14** were used as supplied. A suitable crystal with dimensions 0.52 × 0.15 × 0.11 mm^3 was selected and mounted on a SuperNova, Dual, Cu at home/near, Atlas diffractometer. The crystal was kept at a steady T = 200.00(10) K during data collection. The structure was solved with the ShelXT (Sheldrick, 2015)[25] solution program using iterative methods and by using Olex2 1.5 (Dolomanov et al., 2009)[26,27] as the graphical interface. The model was refined with olex2.refine 1.5 (Bourhis et al., 2015) using full matrix least squares minimisation on F^2.

Crystal Data. $C_{17}H_{17}F_2IO_2$, M_r = 418.225, orthorhombic, $Pca2_1$ (No. 29), a = 10.9409(13) Å, b = 38.4254(18) Å, c = 7.7715(4) Å, $\alpha = \beta = \gamma$ = 90°, V = 3267.2(5) $Å^3$, T = 200.00(10) K, Z = 8, Z' = 2, μ(Cu K_α) = 15.635, 15471 reflections measured, 4547 unique (R_{int} = 0.0650) which were used in all calculations. The final wR_2 was 0.1667 (all data) and R_1 was 0.0602 (I≥2 σ(I)).

**Compound**	**(*rac*)-14**
Formula	$C_{17}H_{17}F_2IO_2$
$D_{calc.}$/ g cm^{-3}	1.700
μ/mm^{-1}	15.635
Formula Weight	418.225
Colour	clear pale colourless
Shape	irregular
Size/mm^3	0.52×0.15×0.11
T/K	200.00(10)
Crystal System	orthorhombic
Flack Parameter	0.006(6)
Hooft Parameter	0.006(6)
Space Group	$Pca2_1$
a/Å	10.9409(13)
b/Å	38.4254(18)
c/Å	7.7715(4)
α/°	90
β/°	90
γ/°	90
V/Å^3	3267.2(5)
Z	8
Z'	2
Wavelength/Å	1.54184
Radiation type	Cu K_α
Θ_{min}/°	3.45
Θ_{max}/°	72.23
Measured Refl's.	15471
Indep't Refl's	4547
Refl's I≥2 σ(I)	4062
R_{int}	0.0650
Parameters	452
Restraints	331
Largest Peak	1.0220
Deepest Hole	-0.9055
GooF	1.0634
wR_2 (all data)	0.1667
wR_2	0.1634
R_1 (all data)	0.0660
R_1	0.0602
CCDC number	**2379681**

Structure Quality Indicators

**Reflections:**	d min (CuKα) 2Θ=144.5° 0.81	I/σ(I) 27.5	Rint m=3.40 6.50%	Full 135.4° 98% to 144.5° 99.8
**Refinement:**	Shift -0.001	Max Peak 1.0	Min Peak -0.9	GooF 1.063
	Hooft .006(6)			

A clear pale colourless irregular-shaped crystal with dimensions 0.52 × 0.15 × 0.11 mm^3 was mounted. Data were collected using a SuperNova, Dual, Cu at home/near, Atlas diffractometer operating at T = 200.00(10) K.

Data were measured using ω scans with Cu K_{α} radiation. The diffraction pattern was indexed and the total number of runs and images was based on the strategy calculation from the program CrysAlisPro system (CCD 43.112a 64-bit (release 01-03-2024)). The maximum resolution that was achieved was Θ = 72.23° (0.81 Å).

The unit cell was refined using CrysAlisPro 1.171.43.115a (Rigaku OD, 2024) on 2300 reflections, 15% of the observed reflections.

Data reduction, scaling and absorption corrections were performed using CrysAlisPro 1.171.43.115a (Rigaku OD, 2024).[28] The final completeness is 99.78 % out to 72.23° in Θ. An analytical absorption correction was performed using CrysAlisPro 1.171.43.115a (Rigaku Oxford Diffraction, 2024). The analytical numeric absorption correction was done using a multifaceted crystal model based on expressions derived by R.C. Clark & J.S. Reid. (Clark, R. C. & Reid, J. S. (1995). Acta Cryst. A51, 887-897). The empirical absorption correction was done using spherical harmonics, implemented in SCALE3 ABSPACK scaling algorithm. The absorption coefficient μ of this crystal is 15.635 mm^{-1} at this wavelength (λ = 1.54184Å) and the minimum and maximum transmissions are 0.069 and 0.387.

The structure was solved and the space group $Pca2_1$ (# 29) determined by the ShelXT (Sheldrick, 2015) structure solution program using iterative methods and refined by full matrix least squares minimisation on $\boldsymbol{F}^2$ using version of olex2.refine 1.5 (Bourhis et al., 2015). All non-hydrogen atoms were refined anisotropically. Hydrogen atom positions were calculated geometrically and refined using the riding model.

_twin_special_details: Component 2 rotated by 177.1232° around [0.08 0.25 0.96] (reciprocal) or [0.04 0.01 1.00] (direct)

The value of Z' is 2. This means that there are two independent molecules in the asymmetric unit. The moiety formula is C17 H17 F2 I O2.

The Flack parameter was refined to 0.006(6). Determination of absolute structure using Bayesian statistics on Bijvoet differences using the Olex2 results in 0.006(6). The chiral atoms in this structure are: C1(S), C8(S), C18(R), C25(R). Note: The Flack parameter is used to determine chirality of the crystal studied, the value should be near 0, a value of 1 means that the stereochemistry is wrong and the model should be inverted. A value of 0.5 means that the crystal consists of a racemic mixture of the two enantiomers.
Ortep-represention of (*rac*)-**14** (thermal ellipsoids set at 50% probability). CCDC 2379681 contains the crystallographic data for (*rac*)-**14**. These data can be obtained free of charge from The Cambridge Crystallographic Data Centre via www.ccdc.cam.ac.uk/data_request/cif.

Datablock: sja-315-racemic-1

Bond precision:	C-C = 0.0172 A		Wavelength=1.54184
Cell:	a=10.9409(13)	b=38.4254(18)	c=7.7715(4)
	alpha=90	beta=90	gamma=90
Temperature:	200 K		

	Calculated	Reported
Volume	3267.2(4)	3267.2(5)
Space group	P c a 21	P c a 21
Hall group	P 2c -2ac	P 2c -2ac
Moiety formula	C17 H17 F2 I O2	C17 H17 F2 I O2
Sum formula	C17 H17 F2 I O2	C17 H17 F2 I O2
Mr	418.21	418.23
Dx,g cm-3	1.700	1.700
Z	8	8
Mu (mm-1)	15.633	15.635
F000	1648.0	1654.8
F000'	1650.67	
h,k,lmax	13,47,9	13,47,9
Nref	6477[3502]	4547
Tmin,Tmax	0.114,0.171	0.069,0.387
Tmin'	0.000	

Correction method= # Reported T Limits: Tmin=0.069 Tmax=0.387
AbsCorr = ANALYTICAL

Data completeness= 1.30/0.70 Theta(max)= 72.230

R(reflections)= 0.0602(4062) wR2(reflections)= 0.1667(4547)

S = 1.063 Npar= 452

Geometry optimization of the active carbenes and the ketyl radical intermediates

Geometry optimizations were carried out using the Gaussian 09 program.[29] For active NHCs, structure optimizations were performed with the B3LYP[30] functional and the def2-SVP[31] basis set, while all ketyl radicals were optimized using the (U)B3LYP functional[30] and the def2-SVP[31] basis set for all atoms. To ensure accurate treatment of dispersion interactions, a refinement step was performed using Grimme's D3 dispersion correction[32] (B3LYP-D3 and UB3LYP-D3) on the pre-optimized geometries.

Optimized geometry analysis of ketyl radical intermediates and comparison of enantioselectivities in corresponding reaction outcomes

Selected ketyl radical intermediates

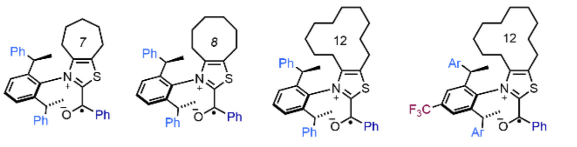

radical 15 radical 16 radical 17 radical 18

Ar = 3,5-di-*tert*-butylphenyl

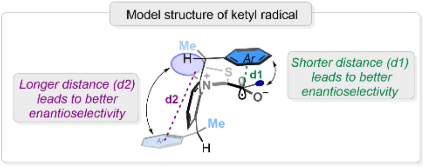

Comparison Chart

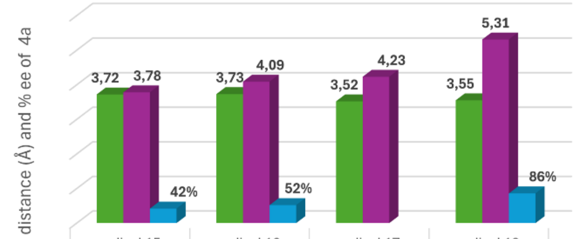

	radical 15	radical 16	radical 17	radical 18
d1 (Å)	3,72	3,73	3,52	3,55
d2 (Å)	3,78	4,09	4,23	5,31
% ee 4a	42%	52%		86%

d1 (Å) d2 (Å) % ee 4a

Optimized 3D structures different ketyl radical intermediates

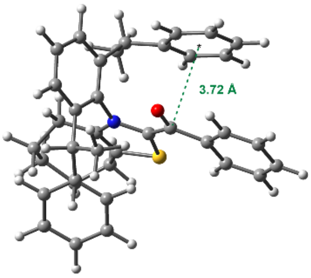

(d1) of radical 15

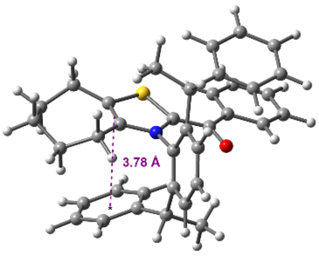

(d2) of radical 15

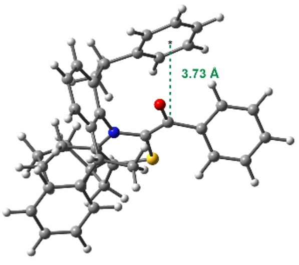

(d1) of radical 16

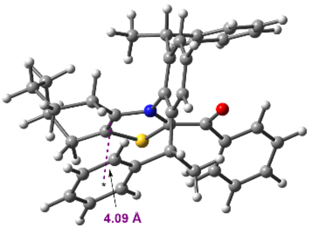

(d2) of radical 16

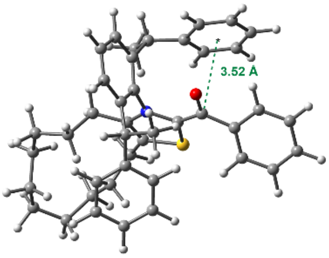

(d1) of radical 17

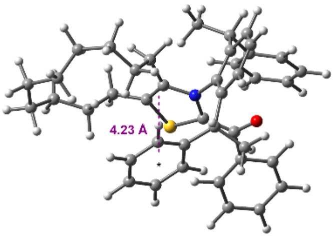

(d2) of radical 17

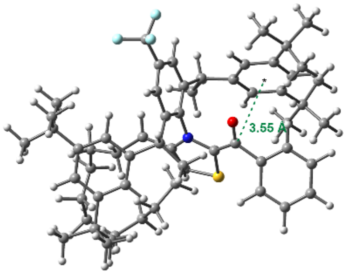

(d1) of radical 18

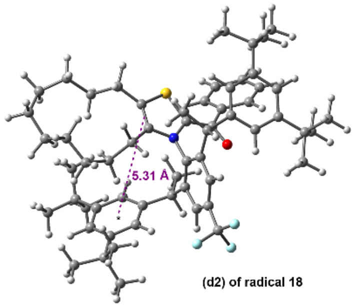

(d2) of radical 18

Three-dimensional arrangements of NHC7’ and its corresponding ketyl radical intermediate 18

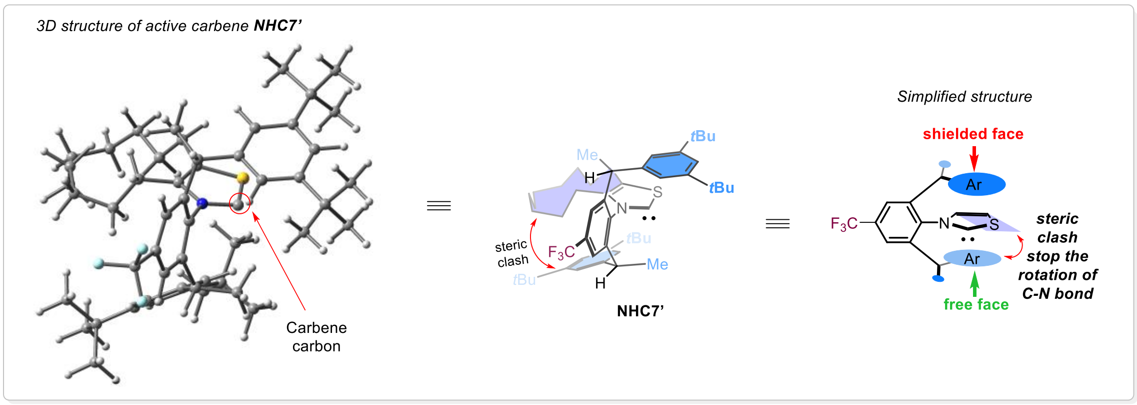

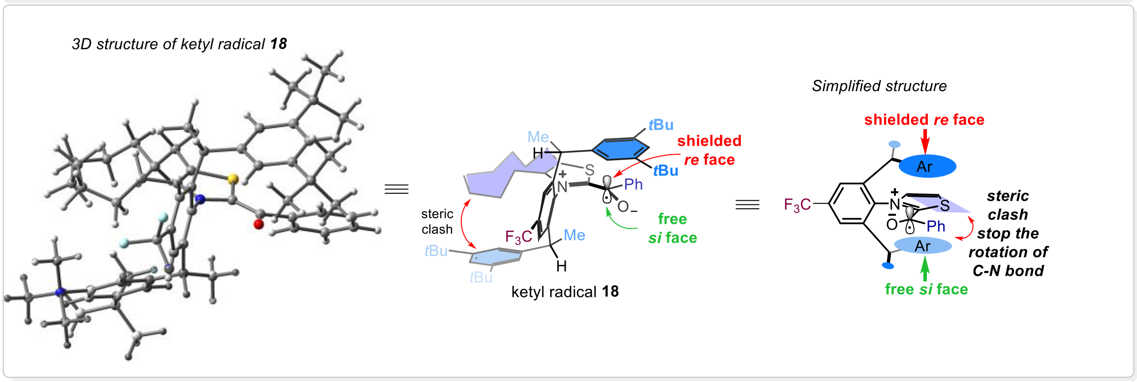

Stationary state coordinates

NHC1' active carbene

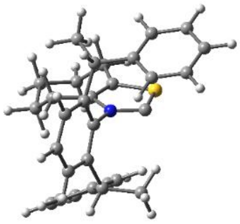

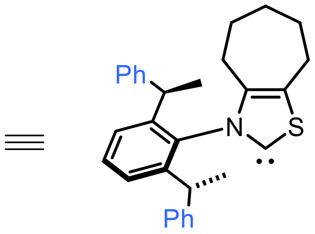

Charge: 0; Spin: 1

C	-1.95721200	2.36580600	2.11137100
C	-1.69528000	1.17780500	1.40927100
C	-0.60699500	1.17246400	0.49886800
C	0.10811400	2.35933900	0.20170800
C	-0.20787600	3.51525500	0.93640300
C	-1.20792600	3.51990800	1.90317500
H	-2.78319300	2.38149600	2.82722200
H	0.34157600	4.43640300	0.72568100
H	-1.42585300	4.42964200	2.46809900
C	0.63270900	-1.01511000	0.49992800
C	-0.76877700	-0.39053600	-1.34106700
N	-0.23467300	-0.07817500	-0.13929700
C	1.10427000	2.55441300	-0.95538900
H	1.47430100	3.58088800	-0.80539700
C	-2.68307100	0.01971300	1.64590900
H	-3.41812400	0.45422700	2.34438800
C	-2.11166600	-1.19542800	2.39125500
H	-1.43543600	-1.79912700	1.77152700
H	-1.56266800	-0.86843700	3.28678900
H	-2.93030500	-1.85058100	2.72704800
C	0.36400100	2.57455100	-2.30825500
H	1.06629500	2.80380500	-3.12543600
H	-0.14136100	1.62050600	-2.51684600
H	-0.41030900	3.35713200	-2.29341900
C	-3.50854000	-0.33153300	0.39980600
C	-3.73554300	-1.65227500	-0.01061300
C	-4.12940800	0.69999300	-0.32615200
C	-4.54887100	-1.93441800	-1.11401100
H	-3.27150800	-2.48355000	0.52152000
C	-4.94086500	0.42308200	-1.42566500
H	-3.96698800	1.73937900	-0.02945700
C	-5.15473400	-0.89984800	-1.82694900
H	-4.70317900	-2.97348000	-1.41723100
H	-5.40664600	1.24501500	-1.97593800
H	-5.78734200	-1.11990500	-2.69076700
C	2.38189000	1.71183200	-0.95057500
C	2.62237200	0.67963500	-1.86921100
C	3.41942500	2.06334500	-0.06762200
C	3.86069400	0.02869100	-1.91093700
H	1.84023900	0.37941600	-2.56774100
C	4.65765600	1.41915900	-0.10750600

H	3.25842400	2.87415900	0.64961700
C	4.88581100	0.39857400	-1.03692000
H	4.02500400	-0.76762500	-2.64188500
H	5.45060700	1.72205000	0.58159400
H	5.85693300	-0.10128600	-1.08060500
S	-0.18722100	-1.96032800	-1.71624100
C	0.75746900	-2.15038000	-0.24181800
C	1.48616200	-3.42824300	0.07779500
C	2.93169000	-3.27521700	0.57259800
H	0.90767100	-3.99155400	0.83580300
H	1.48442700	-4.06337500	-0.82274200
C	3.08743900	-2.59627000	1.93834200
H	3.51644400	-2.72028800	-0.18115900
H	3.37312000	-4.28516400	0.62999100
C	2.75301100	-1.09978000	1.97608300
H	2.47404800	-3.13342700	2.68720300
H	4.13409600	-2.71985500	2.26590800
H	3.09419200	-0.69714300	2.94536100
H	3.33449000	-0.57408700	1.20352600
C	1.26904800	-0.72551600	1.83599300
H	1.15977400	0.34730600	2.04921400
H	0.69953500	-1.24414000	2.62817600

NHC4' active carbene

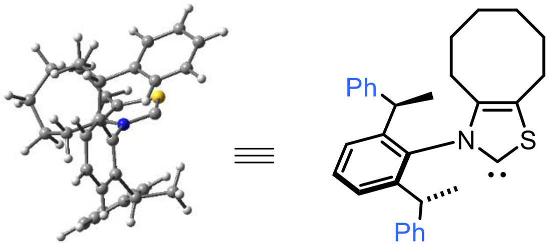

Charge: 0; Spin: 1

C	2.03049800	-1.88568200	2.46636700
C	1.70196100	-0.88130400	1.54055600
C	0.65267100	-1.14379800	0.62441300
C	0.04448900	-2.42205600	0.54907800
C	0.42434600	-3.38553700	1.49872000
C	1.38509100	-3.11867600	2.46904500
H	2.82493000	-1.69146000	3.19167300
H	-0.04130000	-4.37376300	1.45896500
H	1.65314900	-3.88078500	3.20495800
C	-0.86262800	0.79840000	0.12144000
C	0.79866400	0.07669100	-1.45816600
N	0.19962600	-0.08202500	-0.25762600
C	-0.89564400	-2.91082400	-0.56915000
H	-1.18539200	-3.92198900	-0.24108400
C	2.59004100	0.37545500	1.55650200
H	3.26696800	0.20156700	2.41012600
C	1.87642600	1.69299000	1.88443800
H	1.22151000	2.03914100	1.07452900
H	1.27038600	1.58332500	2.79628100

H	2.61603100	2.48513300	2.07734400
C	-0.11667100	-3.10555300	-1.88434600
H	-0.76788000	-3.54724500	-2.65527900
H	0.30512300	-2.16252400	-2.26115500
H	0.72688100	-3.79237800	-1.71573200
C	3.52578300	0.44992500	0.33889700
C	3.70562300	1.62306400	-0.40688200
C	4.29266500	-0.67536000	-0.00969800
C	4.61722000	1.67077900	-1.46732200
H	3.12637700	2.51800800	-0.17718800
C	5.20228400	-0.63211000	-1.06617000
H	4.17099400	-1.60495400	0.55210700
C	5.36952600	0.54496200	-1.80296700
H	4.73260000	2.59722000	-2.03634700
H	5.78309600	-1.52371400	-1.31747700
H	6.07946400	0.58130000	-2.63329300
C	-2.23276800	-2.18272600	-0.72597600
C	-2.55309300	-1.39971900	-1.84387000
C	-3.22619300	-2.37188200	0.25270000
C	-3.82073900	-0.82086500	-1.97720400
H	-1.80863300	-1.23016100	-2.62293700
C	-4.49106700	-1.79485500	0.12580400
H	-3.00480800	-2.99452100	1.12521400
C	-4.79486300	-1.01446700	-0.99543800
H	-4.04563100	-0.21722500	-2.86063900
H	-5.24655400	-1.96493800	0.89766600
H	-5.78719000	-0.56931100	-1.10555000
S	0.01311800	1.41302900	-2.19061600
C	-1.10981000	1.71075600	-0.86222200
C	-2.14282900	2.80443300	-0.96568500
H	-3.13963800	2.37613700	-0.78346400
H	-2.16929400	3.15545500	-2.00982300
C	-2.80474200	1.41259400	1.74927200
H	-3.33833800	0.89470900	2.56342400
H	-3.48306300	1.34701100	0.88689300
C	-1.53457000	0.59608300	1.45820400
H	-1.79576700	-0.46961400	1.53365500
H	-0.79635300	0.76387400	2.26184000
C	-2.59928200	2.87382700	2.19778100
H	-3.58355900	3.37663400	2.20951800
H	-2.26244600	2.86107800	3.24983000
C	-1.59491600	3.73075100	1.41349900
H	-1.48877800	4.69578600	1.93892600
H	-0.59979700	3.26068200	1.46894300
C	-1.92291200	4.03035100	-0.05299300
H	-1.10020700	4.63743000	-0.46896800
H	-2.82750200	4.66192100	-0.11148500

NHC13' active carbene

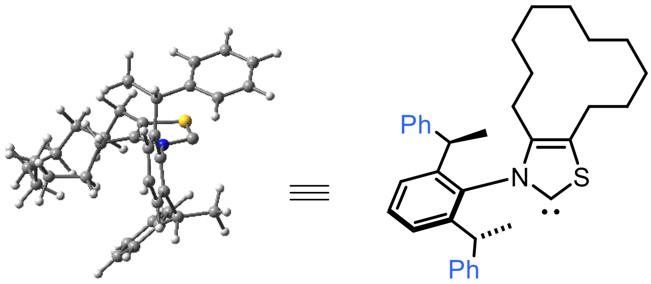

Charge: 0; Spin: 1

C	-2.99154600	1.59200300	2.52556300
C	-2.52176900	0.61772300	1.62980300
C	-1.65264200	1.03694200	0.58772500
C	-1.39283000	2.41210100	0.35539400
C	-1.89892000	3.33420100	1.28951400
H	-3.64825300	1.28163200	3.34254500
H	-1.69868700	4.39749600	1.13487500
C	0.11360000	-0.69712500	0.20489700
C	-1.58915200	-0.31213000	-1.43071400
N	-1.03107600	0.02180800	-0.24479600
C	-0.75947400	3.03881800	-0.90546700
H	-0.72523500	4.11133900	-0.66065300
C	-3.08585800	-0.80464100	1.82578700
H	-3.83461800	-0.66544700	2.62373400
C	-2.10911700	-1.84511300	2.39552600
H	-1.34720200	-2.16261600	1.67185300
H	-1.59706900	-1.44553100	3.28343500
H	-2.66330200	-2.74250400	2.71131700
C	-1.71943300	2.91811400	-2.10788400
H	-1.30389400	3.44847700	-2.97953100
H	-1.91262900	1.86918000	-2.37453000
H	-2.68862900	3.37691000	-1.85682900
C	-3.90588100	-1.29786500	0.62600900
C	-3.75116700	-2.57646100	0.07380000
C	-4.90871400	-0.46713600	0.09582000
H	-2.98341600	-3.25226100	0.45255500
H	-5.04830600	0.53618100	0.50697000
C	-5.55370900	-2.17266700	-1.49694100
H	-6.18785500	-2.51002100	-2.32084200
C	0.69488000	2.70528700	-1.24412200
C	1.06307700	1.76950700	-2.22225700
C	1.71810200	3.44665700	-0.62627400
H	0.29642100	1.18978300	-2.73734100
H	1.45430700	4.19897400	0.12348000
C	3.41178700	2.31364900	-1.93199000
H	4.45982600	2.16701500	-2.20590900
S	-0.64616400	-1.60950700	-2.04687700
C	0.46411500	-1.67248200	-0.68381500
C	1.59082200	-2.66411200	-0.56689100
H	1.59418100	-3.06911400	0.46160200
H	1.39385200	-3.52717600	-1.22454200

C	0.81674500	-0.40867100	1.50627600
H	0.08307900	-0.13522200	2.27740500
H	1.25576900	-1.36294900	1.83921500
C	2.98185700	-2.07737300	-0.86870200
H	3.04984900	-1.82946100	-1.94206800
H	3.06942400	-1.11717000	-0.34640700
C	4.15051400	-2.98997000	-0.47389900
H	3.96211600	-3.43016000	0.52290800
H	4.18319400	-3.84755200	-1.16907000
C	5.52307400	-2.28860600	-0.46771200
H	5.57240500	-1.59144200	-1.32491300
H	6.01620700	-2.26795500	1.64569300
C	5.94137100	-1.54086000	0.81548300
H	6.97294200	-1.18630100	0.64837900
H	6.30241900	-3.04543900	-0.66212000
C	1.92448700	0.67185300	1.48129200
H	2.40302500	0.70328200	0.49153800
H	1.46020300	1.66090700	1.60814900
C	2.98943100	0.46067200	2.57205000
H	3.55755000	1.39803500	2.70733700
H	2.48118500	0.27839000	3.53608300
C	3.98097500	-0.68394000	2.29382200
H	4.46839400	-0.98030300	3.23945000
H	3.42732800	-1.58048300	1.96697800
C	5.08440700	-0.34115600	1.27726100
H	4.65713600	0.16997600	0.39723600
H	5.74562000	0.40734700	1.74831300
C	-5.72251500	-0.89520400	-0.95206700
H	-6.49166900	-0.22719500	-1.34886100
C	-4.56553700	-3.00970600	-0.97889700
H	-4.41900700	-4.00945900	-1.39648700
C	3.06039600	3.25622800	-0.96018900
H	3.83331600	3.85325900	-0.46874700
C	2.40680300	1.57263400	-2.55933100
H	2.66569600	0.83967400	-3.32814400
C	-2.66000200	2.93581400	2.38303400
H	-3.02857700	3.67543400	3.09812300

NHC7' active carbene

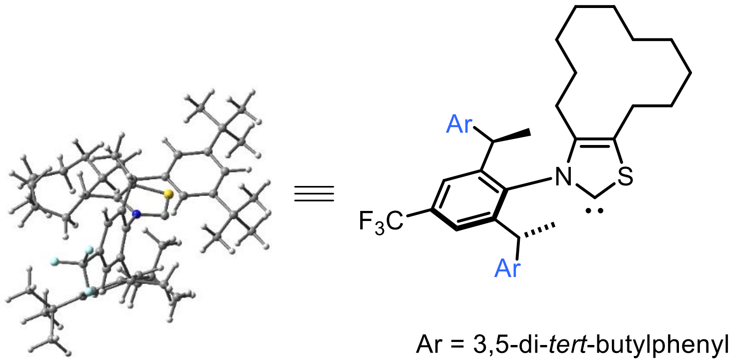

Ar = 3,5-di-*tert*-butylphenyl

Charge: 0; Spin: 1

C	1.90121700	2.96573600	-1.74160900
C	1.77241200	1.61200700	-1.39874700
C	0.82432900	1.26621200	-0.39812700
C	0.16510000	2.26423500	0.36554400
C	0.34093000	3.60104300	-0.02904700
C	1.16334900	3.95368200	-1.09521700
H	2.61403300	3.25218100	-2.51696600
H	-0.16250400	4.38835400	0.53463900
C	-0.28722400	-0.86571400	-1.07546200
C	1.14342800	-0.79828800	0.84218900
N	0.54112500	-0.13769300	-0.17216000
C	-0.57249500	2.07935100	1.70832400
H	-0.91446400	3.09978000	1.93951500
C	2.76296100	0.65306200	-2.09420600
H	3.44455700	1.34476900	-2.61661400
C	2.17606900	-0.22018400	-3.21443100
H	1.54818200	-1.03829500	-2.83846500
H	1.57417000	0.38945500	-3.90450300
H	2.99303700	-0.66983600	-3.79910000
C	0.44096800	1.73449300	2.81970000
H	-0.05718700	1.74352900	3.80183200
H	0.91216600	0.75541100	2.65410600
H	1.24249900	2.48954900	2.83795600
C	3.67209300	-0.09873100	-1.11153900
C	3.90466700	-1.47436200	-1.20838400
C	4.35175300	0.63569500	-0.12778600
C	4.78886000	-2.13127400	-0.33650400
H	3.38365400	-2.04915000	-1.96977200
C	5.23849900	0.02416500	0.76479600
H	4.16801100	1.70814200	-0.06337100
C	5.43663700	-1.36129800	0.63809200
H	6.12081200	-1.85798400	1.32880000
C	-1.87387100	1.26779000	1.77285200
C	-1.98067200	0.05675100	2.46767300
C	-3.04882600	1.86563900	1.28897600
C	-3.22873900	-0.54676200	2.70838600
H	-1.07523500	-0.40372000	2.86358400
C	-4.31366300	1.29796300	1.49298200
H	-2.96616900	2.82984500	0.77906400

C	-4.37467700	0.08668900	2.20354300
H	-5.34682800	-0.35901200	2.39497000
S	0.67354400	-2.44101700	0.67583700
C	-0.31245700	-2.19377600	-0.76045100
C	-1.00963900	-3.30523800	-1.50085300
H	-0.78872800	-3.19449600	-2.57854100
H	-0.57127300	-4.27356200	-1.20756400
C	-1.01266900	-0.23044000	-2.23254100
H	-0.40262600	0.57356400	-2.66602500
H	-1.08100000	-1.00245800	-3.01553200
C	-2.53694000	-3.34195200	-1.31399000
H	-2.77191200	-3.63368500	-0.27640300
H	-2.92193000	-2.32152300	-1.42166700
C	-3.26872600	-4.27245500	-2.28935700
H	-2.88431100	-4.11792100	-3.31466900
H	-3.01846600	-5.31841500	-2.03824300
C	-4.80064700	-4.10406500	-2.29012900
H	-5.14421600	-3.95414900	-1.24956400
H	-5.16799100	-3.22415900	-4.24058100
C	-5.40286800	-2.99939600	-3.18339200
H	-6.49914800	-3.09654800	-3.10441400
H	-5.25520500	-5.05649300	-2.61278600
C	-2.42763900	0.33185300	-1.95859900
H	-2.92771400	-0.25557900	-1.17376300
H	-2.33508000	1.34580000	-1.54102100
C	-3.29522900	0.37248600	-3.22992100
H	-4.14970500	1.05074500	-3.06451000
H	-2.70624200	0.83080900	-4.04444400
C	-3.82798900	-0.99475300	-3.69660100
H	-4.13955300	-0.91890400	-4.75321900
H	-3.00658700	-1.73125300	-3.69662400
C	-5.02218200	-1.53151400	-2.88715100
H	-4.84481300	-1.40901800	-1.80441000
H	-5.88967500	-0.88581900	-3.10979700
C	5.05407700	-3.65010400	-0.41193800
C	5.97596200	0.80475500	1.87202200
C	7.50264400	0.61534600	1.71787600
H	8.04055100	1.16999700	2.50460500
H	7.79866100	-0.44171300	1.79746300
H	7.85075600	0.98732900	0.74087700
C	5.67593900	2.31379800	1.82151600
H	5.98507100	2.76201400	0.86402500
H	4.60574400	2.52566000	1.96913400
H	6.22820600	2.83074500	2.62227600
C	5.52592600	0.26983500	3.25159400
H	4.43903900	0.38979700	3.38215100
H	5.75912300	-0.79911100	3.37189000
H	6.03406700	0.81692700	4.06329100
C	6.56076100	-3.90267800	-0.65033600
H	7.18109000	-3.48730500	0.15828300
H	6.76681800	-4.98467300	-0.70553000

H	6.89324300	-3.44383100	-1.59527300
C	4.27162100	-4.32479300	-1.55360600
H	3.18404600	-4.20768100	-1.42924300
H	4.55081200	-3.92019000	-2.53933800
H	4.48889100	-5.40470300	-1.56661800
C	4.62484400	-4.31100700	0.91864000
H	3.55340900	-4.14511400	1.11100100
H	4.80474000	-5.39868500	0.88566100
H	5.18413500	-3.90651700	1.77580500
C	-3.31430100	-1.82034200	3.57880600
C	-5.58127200	2.04423800	1.02161700
C	-2.76349700	-1.50010300	4.98925100
H	-3.34118200	-0.69162900	5.46519800
H	-2.82415400	-2.38985200	5.63786600
H	-1.70994100	-1.18433800	4.95482100
C	-4.75923000	-2.32896200	3.73606700
H	-4.76640500	-3.24059200	4.35417900
H	-5.40781300	-1.59044100	4.23254100
H	-5.21261400	-2.58499100	2.76540600
C	-2.47581000	-2.95510300	2.95332500
H	-2.86995700	-3.23430400	1.96345300
H	-1.42232700	-2.67113900	2.82150700
H	-2.50514700	-3.85168200	3.59447000
C	-6.86871300	1.24492000	1.29553000
H	-7.02498100	1.06782300	2.37086000
H	-7.74112100	1.80644700	0.92554400
H	-6.86223900	0.26905000	0.78511300
C	-5.68189800	3.39046300	1.77768000
H	-4.81293100	4.03633100	1.57977500
H	-6.58565800	3.94172800	1.46862000
H	-5.73696000	3.22842100	2.86593300
C	-5.50603900	2.31701900	-0.49651900
H	-4.61839000	2.90790300	-0.76812100
H	-5.46920500	1.37385600	-1.06322300
H	-6.39375700	2.87865100	-0.83124000
C	1.25491000	5.39117300	-1.54126100
F	0.31207400	5.68254200	-2.45989800
F	2.44499400	5.66960100	-2.10223000
F	1.07926100	6.24711800	-0.51887200

Ketyl radical 15

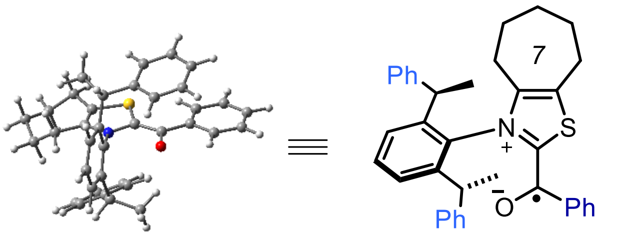

Charge: 0; Spin: 2

C	0.01289200	3.83610900	-1.31883600
C	-0.09148300	2.74226200	-0.44605600
C	0.49584200	1.51084800	-0.84890100
C	1.11386400	1.37092600	-2.11640900
C	1.19519100	2.51468400	-2.93118000
C	0.66600900	3.73837700	-2.54386000
H	-0.42680700	4.79091800	-1.01930400
H	1.68305100	2.42670800	-3.90577200
H	0.75122200	4.61019500	-3.19712500
C	1.67590900	0.39783200	1.02403900
N	0.58684900	0.43995300	0.12991500
C	1.60849300	0.08487600	-2.81696100
H	2.40148400	0.45134400	-3.48817100
C	-0.88040700	3.00906600	0.85337500
H	-1.14497000	4.07537100	0.76325200
C	-0.06996500	2.93206500	2.15595400
H	0.20950800	1.90877400	2.43856300
H	0.84894500	3.53002900	2.06621300
H	-0.65717400	3.35558400	2.98528200
C	0.48998800	-0.44999900	-3.74221200
H	0.84899300	-1.32655100	-4.30415400
H	-0.41121700	-0.71547900	-3.17583500
H	0.19501600	0.32648100	-4.46413900
C	-2.24315600	2.30491500	0.92194100
C	-2.66174500	1.56898300	2.03947600
C	-3.15495700	2.47524400	-0.13408600
C	-3.95008000	1.02447300	2.10395100
H	-1.98346800	1.40455800	2.87768600
C	-4.44066800	1.93897700	-0.07161000
H	-2.84800600	3.02873900	-1.02456400
C	-4.84643000	1.21013000	1.05135200
H	-4.24910800	0.44996500	2.98466700
H	-5.12869900	2.08479500	-0.90847900
H	-5.85120500	0.78306500	1.09967700
C	2.28902700	-1.03501300	-2.03038800
C	1.60115700	-2.16710900	-1.56276300
C	3.68754100	-1.01747600	-1.89140300
C	2.28220600	-3.22507200	-0.95420500
H	0.52023800	-2.23491400	-1.68639800
C	4.37599000	-2.07809200	-1.29519900
H	4.25142100	-0.16382600	-2.28007200
C	3.67294100	-3.18735100	-0.81724800

H	1.71949300	-4.09141100	-0.59698400
H	5.46595700	-2.04175800	-1.21529000
H	4.20604700	-4.02352600	-0.35721400
S	0.02817800	-1.41681100	1.81201600
C	1.53626700	-0.53917400	2.00192500
C	2.36497300	-0.81301200	3.22961700
C	3.88362400	-0.89669600	3.03246000
H	2.14666800	-0.03645600	3.98908100
H	2.01673200	-1.76014600	3.67299900
C	4.55124400	0.41296600	2.60313000
H	4.11256400	-1.68683900	2.29672300
H	4.32422700	-1.22220800	3.99034800
C	4.22958500	0.86761400	1.17589400
H	4.28808600	1.21381100	3.32042800
H	5.64468400	0.28709400	2.68355000
H	4.91802100	1.68900000	0.91342700
H	4.44680600	0.04623900	0.47622600
C	2.80905600	1.39097100	0.91202800
H	2.78970800	1.83196500	-0.09259200
H	2.60783100	2.22918800	1.60285900
C	-0.40408100	-0.51225900	0.37551200
C	-1.53916100	-0.70561400	-0.48795600
C	-2.58076900	-1.77615300	-0.28540000
C	-3.63338500	-1.77722800	-1.22640700
C	-2.60277200	-2.77030000	0.71208900
C	-4.65632300	-2.72052700	-1.17291900
H	-3.61680300	-1.00806300	-1.99873100
C	-3.62950400	-3.71809700	0.76589500
H	-1.82127800	-2.83818400	1.46597400
C	-4.66223800	-3.70026800	-0.17330300
H	-5.45728900	-2.69283100	-1.91686500
H	-3.61572900	-4.47771300	1.55216500
H	-5.46336100	-4.44272700	-0.12834900
O	-1.65605400	0.04710500	-1.48359300

Ketyl radical 16

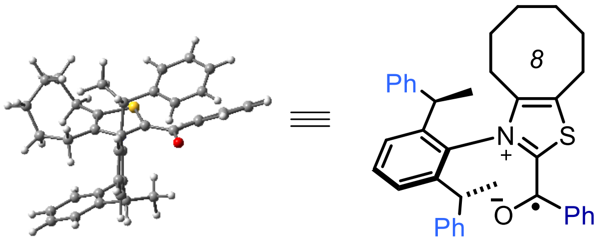

Charge: 0; Spin: 2

C	-0.05718200	3.56393500	-1.45101600
C	-0.18495800	2.49367100	-0.54975600
C	0.36202600	1.24062800	-0.93295000
C	0.95169700	1.05176600	-2.21069200
C	1.02265200	2.16133000	-3.06899900
C	0.54439500	3.41200400	-2.69520200
H	-0.45054200	4.54208900	-1.16296700
H	1.46273400	2.03113400	-4.06083700
H	0.62677500	4.26190200	-3.37706600
C	1.48826400	-0.02451500	0.89777300
N	0.39022000	0.15334200	0.02968400
C	1.48131500	-0.27417200	-2.80288000
H	1.72797600	0.00550100	-3.83901800
C	-0.97201900	2.81417900	0.73865500
H	-1.03551700	3.91457900	0.72199700
C	-0.27887100	2.49463000	2.06842700
H	-0.19854400	1.42015700	2.27453700
H	0.73192200	2.92674900	2.08780800
H	-0.84285500	2.94807900	2.89791200
C	0.42617700	-1.38273200	-2.93806400
H	0.81169000	-2.17804800	-3.59581200
H	0.15811500	-1.84335100	-1.97984900
H	-0.49955500	-0.98011800	-3.37016900
C	-2.44405600	2.36708800	0.69305400
C	-3.02971400	1.59390300	1.70511200
C	-3.27221100	2.82435600	-0.34610000
C	-4.39865700	1.29956500	1.68922800
H	-2.42448300	1.20793700	2.52616600
C	-4.63692200	2.53864200	-0.36392100
H	-2.84094600	3.41169300	-1.15993700
C	-5.21037900	1.77666600	0.65997600
H	-4.82795600	0.69306600	2.49100200
H	-5.25681400	2.91215300	-1.18340000
H	-6.28013700	1.55222600	0.65028800
C	2.82105700	-0.76038300	-2.23406600
C	2.95571700	-1.94480800	-1.49526900
C	3.98851800	-0.02645500	-2.51664900
C	4.21084000	-2.37834100	-1.05006600
H	2.07684400	-2.54744700	-1.26320000
C	5.24165300	-0.45296900	-2.07355100
H	3.91143400	0.89510100	-3.10122000
C	5.35858400	-1.63629900	-1.33551000

H	4.28835800	-3.30977700	-0.48252600
H	6.13199500	0.13424900	-2.31399000
H	6.33853300	-1.97957200	-0.99400400
S	-0.23454500	-1.84992200	1.51802900
C	1.32908800	-1.07363300	1.75592600
C	2.24147700	-1.65352800	2.80822500
H	3.23173100	-1.83512300	2.36686000
H	1.86356100	-2.65413800	3.07437800
C	3.91596200	0.66160200	1.61437000
H	4.72850500	1.23794500	1.14248500
H	4.21913100	-0.38711800	1.49177000
C	2.65310000	0.93212600	0.77986400
H	2.95029600	0.95161000	-0.27750900
H	2.28808400	1.95203300	0.98681400
C	3.87527400	1.06987600	3.10097200
H	4.78829800	0.68457000	3.59048900
H	3.95578200	2.17046200	3.15325800
C	2.64385300	0.66725200	3.92419600
H	2.74081700	1.11972500	4.92633100
H	1.74799900	1.13027400	3.48164900
C	2.38716700	-0.83148300	4.10775400
H	1.46804300	-0.95101900	4.70700100
H	3.20125400	-1.28219700	4.70316300
C	-0.64586500	-0.76953700	0.19913200
C	-1.83252700	-0.81126100	-0.61208300
C	-2.85675800	-1.90504900	-0.44772700
C	-4.18360800	-1.57802600	-0.78934400
C	-2.57544400	-3.23221200	-0.07612100
C	-5.19812400	-2.53100200	-0.71955000
H	-4.39289900	-0.55769000	-1.11224800
C	-3.59209700	-4.19105800	-0.01677700
H	-1.55248100	-3.54457100	0.13863300
C	-4.90843000	-3.84362500	-0.32784800
H	-6.22340600	-2.25104500	-0.97671000
H	-3.34767900	-5.21889400	0.26491100
H	-5.70294900	-4.59282000	-0.27634200
O	-2.03473700	0.07028400	-1.47225400

Ketyl radical 17

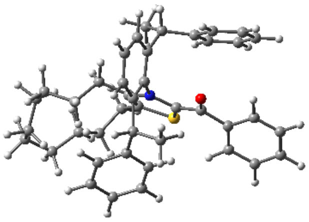

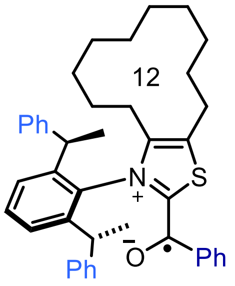

Charge: 0; Spin: 2

C	1.21359400	4.00300200	0.97888600
C	1.09628200	2.83308600	0.21403400
C	0.57174600	1.67051100	0.84887100
C	0.25814800	1.66497200	2.23213700
C	0.39026600	2.88160600	2.92947100
H	1.60335900	4.90616200	0.50253100
H	0.13688000	2.90134300	3.99282600
C	-0.97916300	0.51084800	-0.69318700
N	0.23446100	0.53017600	0.01529300
C	-0.05943300	0.47248000	3.16998000
H	-0.63298600	0.94450600	3.98279500
C	1.60703300	2.94862500	-1.24072400
H	1.94386800	3.99615300	-1.29361300
C	0.54954500	2.84277700	-2.35118400
H	0.13806600	1.83307400	-2.47458700
H	-0.28617900	3.53032100	-2.15431200
H	0.99909100	3.13571500	-3.31268400
C	1.25798500	-0.01419100	3.82147500
H	1.04757500	-0.81330400	4.54951300
H	1.97839600	-0.37423300	3.07673200
H	1.74421300	0.81811400	4.35253200
C	2.88532100	2.14874300	-1.52876200
C	2.97343000	1.19554200	-2.55213300
C	4.05042900	2.44643700	-0.80173800
H	2.09025500	0.92853700	-3.13385500
H	4.00192900	3.17337800	0.01311400
C	5.34101700	0.88687400	-2.13000900
H	6.29108500	0.40075500	-2.36598800
C	-0.95119900	-0.69300700	2.73925400
C	-0.44342200	-1.90190400	2.23659800
C	-2.32999700	-0.62996700	3.00685700
H	0.62742100	-2.01069900	2.06841100
H	-2.74625800	0.28124400	3.44573100
C	-2.65738700	-2.89587500	2.22047600
H	-3.31260200	-3.74877600	2.02518900
S	0.30750800	-1.59861500	-1.43963200
C	-1.10426000	-0.55988800	-1.53030500
C	-2.23967800	-0.89209300	-2.46045500
H	-2.52772500	0.02641800	-3.00294900
H	-1.88501300	-1.59101200	-3.23683200

C	-2.00457900	1.61442500	-0.58883300
H	-1.49697800	2.58091000	-0.47153000
H	-2.50476900	1.65733700	-1.56852900
C	-3.48678000	-1.46553100	-1.76360100
H	-3.25022400	-2.45954300	-1.34679600
H	-3.71136800	-0.83690500	-0.89361900
C	-4.72343300	-1.55816500	-2.66749300
H	-4.81122700	-0.64062300	-3.27832100
H	-4.57383700	-2.37721000	-3.39301800
C	-6.04368400	-1.78745300	-1.90626500
H	-5.85921500	-2.49141900	-1.07339700
H	-7.08122800	0.09417600	-2.21515900
C	-6.78321500	-0.54633300	-1.36397000
H	-7.73079400	-0.91133900	-0.93213100
H	-6.74807300	-2.30793200	-2.57759800
C	-3.08095700	1.49187100	0.51395800
H	-3.29708900	0.43333600	0.71356700
H	-2.67122800	1.89034100	1.45431400
C	-4.38369600	2.23254500	0.16237300
H	-4.96890000	2.39185700	1.08538000
H	-4.13293000	3.24241400	-0.20872200
C	-5.27274900	1.51409100	-0.86929300
H	-5.98928200	2.23744100	-1.29669100
H	-4.65597600	1.18690500	-1.72331900
C	-6.07341300	0.32670000	-0.30560300
H	-5.42965900	-0.31050600	0.32577900
H	-6.83235800	0.73763800	0.38318400
C	5.26524600	1.82997000	-1.09906400
H	6.15787900	2.08485900	-0.52152200
C	4.18892000	0.56689400	-2.84873000
H	4.23048500	-0.17529300	-3.65024800
C	-3.17720300	-1.71087900	2.74911600
H	-4.24353600	-1.63162900	2.97785100
C	-1.28550700	-2.98617000	1.97078400
H	-0.85971900	-3.91434200	1.58067400
C	0.84333400	4.04268200	2.31997700
H	0.92528400	4.97134700	2.89003500
C	1.06835500	-0.55654700	-0.24832300
C	2.36263100	-0.73986400	0.34249500
O	2.85115200	0.16035100	1.06258000
C	3.16397800	-1.99591500	0.12366300
C	4.56091000	-1.87710800	0.26494600
C	2.62927400	-3.27494000	-0.11759600
C	5.39343600	-2.98633600	0.13137800
H	4.96732500	-0.88937500	0.48490900
C	3.46473700	-4.38990100	-0.24177900
H	1.55039600	-3.42417400	-0.18030100
C	4.84956300	-4.25048500	-0.12726500
H	6.47571100	-2.86765200	0.23302500
H	3.02530700	-5.37517700	-0.41956900
H	5.50150600	-5.12212100	-0.22888300

Ketyl radical 18

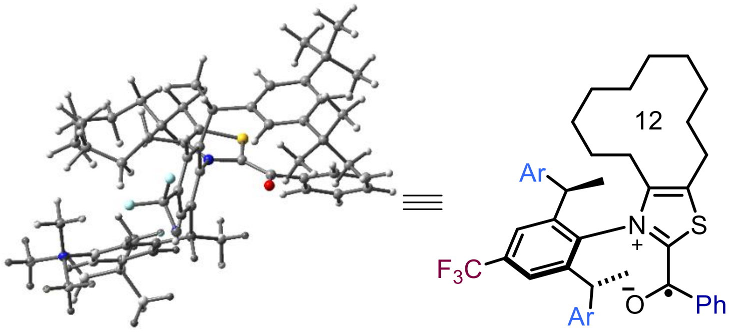

Ar = 3,5-di-*tert*-butylphenyl

Charge: 0; Spin: 2

C	-0.96705600	2.91915100	1.52200600
C	-1.04947000	1.53307700	1.31889700
C	-0.29377000	0.96865300	0.25179000
C	0.43828900	1.79256900	-0.64765500
C	0.40603700	3.17811500	-0.42629300
C	-0.24427500	3.74087800	0.66546400
H	-1.50728000	3.36738500	2.35695000
H	0.91152600	3.83762000	-1.13326400
C	0.58175600	-1.27281500	0.93559500
N	-0.25332500	-0.47748200	0.12754700
C	1.27393600	1.38507300	-1.88423700
H	1.24395300	2.30182500	-2.49652200
C	-2.03856600	0.81369400	2.26462700
H	-2.29146400	1.60410200	2.98956200
C	-1.48872300	-0.32060200	3.14062800
H	-1.28285500	-1.24681100	2.59046400
H	-0.56510200	-0.00531600	3.64594700
H	-2.22354300	-0.56003600	3.92420600
C	0.68607100	0.30989700	-2.80282400
H	1.23317100	0.30713500	-3.75770800
H	0.74416700	-0.70251600	-2.38244100
H	-0.36457800	0.53730900	-3.01729600
C	-3.39005300	0.49308700	1.60043400
C	-4.00651300	-0.75639700	1.72714100
C	-4.08038000	1.52152900	0.94334200
C	-5.29931500	-0.99279100	1.22762200
H	-3.47837800	-1.56039000	2.23323000
C	-5.36671100	1.33495700	0.42736600
H	-3.58964500	2.48676900	0.83238700
C	-5.95503300	0.06776200	0.58683700
H	-6.96219500	-0.09584300	0.19814500
C	2.77952900	1.20374500	-1.59424400
C	3.50641800	0.12806300	-2.11725500
C	3.48285000	2.18914000	-0.88288300
C	4.89884700	0.01979800	-1.95111500
H	2.98315000	-0.64221800	-2.68218800
C	4.86706000	2.12149900	-0.68062100
H	2.93457900	3.03736600	-0.47409500
C	5.55580300	1.02209700	-1.22289400
H	6.63127300	0.95392100	-1.08266700

S	-0.91734200	-2.92978300	-0.37029900
C	0.34419900	-2.61248600	0.81091700
C	0.93193700	-3.74755800	1.60602200
H	1.00066600	-3.42532700	2.66053600
H	0.21990300	-4.59060400	1.60567600
C	1.57572700	-0.69449200	1.91318200
H	1.25081300	0.30713700	2.22615100
H	1.51510000	-1.32339200	2.81588300
C	2.31845300	-4.24298300	1.15938200
H	2.24587500	-4.69396400	0.15447600
H	2.97557900	-3.37323700	1.04262100
C	2.95145600	-5.24497200	2.13764500
H	2.72604200	-4.94331300	3.17712500
H	2.46119400	-6.22570500	2.00788000
C	4.47520300	-5.41074600	1.98379400
H	4.73401800	-5.39028100	0.90869500
H	5.20104200	-4.52560500	3.82802000
C	5.37778300	-4.41507300	2.74175400
H	6.41721300	-4.74905800	2.58196900
H	4.75304800	-6.42047100	2.33149400
C	3.05408300	-0.61323100	1.46171900
H	3.27733300	-1.41623600	0.74390200
H	3.20558400	0.31909300	0.90385600
C	4.03974200	-0.67424400	2.64276000
H	5.00408700	-0.23765000	2.32946900
H	3.66695200	-0.01942700	3.45066100
C	4.29437600	-2.08732800	3.20012700
H	4.68877500	-2.01080800	4.22859100
H	3.33568800	-2.62332500	3.30199900
C	5.28822600	-2.91915900	2.36974700
H	5.06603100	-2.82080700	1.29287800
H	6.28485600	-2.46117300	2.49579000
C	-6.01416600	-2.35006400	1.40638000
C	-6.13050600	2.45675800	-0.30731500
C	-7.49988100	2.69142100	0.36976000
H	-8.05208000	3.49330300	-0.14782900
H	-8.13242400	1.79088500	0.35209000
H	-7.37387400	2.99090100	1.42266600
C	-5.35761700	3.78860300	-0.29732600
H	-5.17313700	4.14891000	0.72705400
H	-4.38813600	3.70271000	-0.81109700
H	-5.94220400	4.56215000	-0.82005000
C	-6.34434200	2.04149800	-1.78130200
H	-5.37706100	1.87745700	-2.28100000
H	-6.93183100	1.11352700	-1.86212400
H	-6.88714000	2.82862400	-2.33098400
C	-7.20768800	-2.16490500	2.37290100
H	-7.93244900	-1.43169400	1.98672300
H	-7.74051200	-3.11955900	2.51982500
H	-6.86712800	-1.80945000	3.35862500
C	-5.08056500	-3.42650400	1.99111200

H	-4.19467900	-3.58637400	1.35655200
H	-4.73451800	-3.16767800	3.00396000
H	-5.61773800	-4.38538200	2.06504500
C	-6.53564600	-2.86484700	0.04596000
H	-5.71132000	-2.99950800	-0.67084300
H	-7.04217200	-3.83580600	0.17431100
H	-7.26258200	-2.17392200	-0.40701600
C	5.65198400	-1.16156800	-2.60033700
C	5.58190500	3.25810400	0.08157600
C	5.48811700	-1.08030500	-4.13670300
H	5.89873400	-0.13589800	-4.52788300
H	6.02035200	-1.91252600	-4.62673500
H	4.43130600	-1.13574500	-4.43924800
C	7.15711800	-1.14643100	-2.27756400
H	7.64897000	-2.00959400	-2.75313700
H	7.65088100	-0.23798500	-2.65595600
H	7.34583000	-1.21277600	-1.19458800
C	5.06906100	-2.50131100	-2.09683900
H	5.18717000	-2.59669300	-1.00591400
H	3.99802000	-2.60114000	-2.32809200
H	5.59136700	-3.34988000	-2.56863800
C	7.05942300	2.93093100	0.36605400
H	7.64316200	2.80701200	-0.55912300
H	7.52058600	3.75388500	0.93446300
H	7.16746900	2.01199400	0.96397400
C	5.52493100	4.54619400	-0.77353900
H	4.48735900	4.85195400	-0.97755100
H	6.02387400	5.37975200	-0.25184700
H	6.02805200	4.39991500	-1.74264800
C	4.88060900	3.51738200	1.43528200
H	3.83724600	3.84419200	1.31160300
H	4.87935800	2.61101100	2.06188300
H	5.40628400	4.31179900	1.98990300
C	-0.11053200	5.21826100	0.92610800
F	1.09994700	5.50968400	1.45453000
F	-1.03388800	5.67238000	1.78901700
F	-0.21928100	5.94010600	-0.20332700
C	-1.14407700	-1.21307700	-0.66430300
C	-2.12537800	-0.65674800	-1.54936200
O	-2.35538300	0.56967600	-1.57925700
C	-2.88309500	-1.56395900	-2.48614200
C	-4.23411100	-1.26370300	-2.73664300
C	-2.28461800	-2.60985000	-3.21095100
C	-4.97335400	-2.00834100	-3.65569600
H	-4.69107900	-0.43584500	-2.19420000
C	-3.02287700	-3.34903600	-4.14068900
H	-1.22580700	-2.83824000	-3.07504800
C	-4.37111400	-3.05694700	-4.36082100
H	-6.02592100	-1.76766000	-3.82730900
H	-2.53633800	-4.15054500	-4.70302500
H	-4.94863700	-3.63704200	-5.08540100

References

1. Diesel, J.; Finogenova, A. M.; Cramer, N. Nickel-Catalyzed Enantioselective Pyridone C-H Functionalizations Enabled by a Bulky *N*-Heterocyclic Carbene Ligand. *J. Am. Chem. Soc*. **2018**, *140*, 4489–4493.
2. Braconi, E.; Cramer, N. A Chiral Naphthyridine Diimine Ligand Enables Nickel-Catalyzed Asymmetric Alkylidenecyclopropanations. *Angew. Chem. Int. Ed.* **2020**, *59*, 16425–16429.
3. Arienti, A.; Bigi, F.; Maggi, R.; Marzi, E.; Moggi, P.; Rastelli, M.; Sartori, G.; Tarantola, F. Regioselective Electrophilic Alkylation of Anilines with Phenylacetylene in the Presence of Montmorillonite KSF. *Tetrahedron* **1997**, 53, 3795–3804.
4. Pesch, J.; Harms, K.; Bach, T. Preparation of Axially Chiral *N*, *N'*-Diarylimidazolium and *N*-Arylthiazolium Salts and Evaluation of Their Catalytic Potential in the Benzoin and in the Intramolecular Stetter Reactions. *Eur. J. Org. Chem*. **2004**, 2025–2035.
5. Piel, I.; Pawelczyk, M. D.; Hirano, K.; Fröhlich, R.; Glorius, F. A Family of Thiazolium Salt Derived *N*-Heterocyclic Carbenes (NHCs) for Organocatalysis: Synthesis, Investigation and Application in Cross-Benzoin Condensation. *Eur. J. Org. Chem*. **2011**, 5475–5484.
6. Skvorcova, M.; Jirgensons, A. Amide-Group-Directed Protonolysis of Cyclopropane: An Approach to 2,2-Disubstituted Pyrrolidines. *Org. Lett.* **2017**, *19*, 2478–2481.
7. Andersen, T. L.; Frederiksen, M. W.; Domino, K.; Skrydstrup, T. Direct Access to *α,α*-Difluoroacylated Arenes by Palladium-Catalyzed Carbonylation of (Hetero)Aryl Boronic Acid Derivatives. *Angew. Chem. Int. Ed.* **2016**, *55*, 10396–10400.
8. Zhao, G.; Lim, S.; Musaev, D. G.; Ngai, M. Y. Expanding Reaction Profile of Allyl Carboxylates via 1,2-Radical Migration (RaM): Visible-Light-Induced Phosphine-Catalyzed 1,3-Carbobromination of Allyl Carboxylates. *J. Am. Chem. Soc.* **2023***, 145,* 8275−8284.
9. Itoh, T.; Sakabe, K.; Kudo, K.; Ohara, H.; Takagi, Y.; Kihara, H.; Zagatti, P.; Renou, M. Systematic Synthesis of Multifluorinated *α,α*-Difluoro-*γ*-Lactones through Intramolecular Radical Cyclization. *J. Org. Chem.* **1999**, *64*, 252–265.
10. Zhang, P.; Wang, S.; Li, W.; Xu, W.; Chen, B.; Wang, X. Preparation method of 2-halogenated-2, 2-difluoroacetic acid alkyne ester. US Patent US11242307B1, February 8, 2022.
11. Luan, Y. Y.; Li, J. Y.; Shi, W. Y.; Zhang, Z.; Jiao, R. Q.; Chen, X.; Liu, X. Y.; Liang, Y. M. Ruthenium-Catalyzed Difunctionalization of Vinyl Cyclopropanes for Double m-C(Sp2)-H/C-5(Sp3)-H Functionalization. *Org. Lett.* **2024**, 26, 3213–3217.
12. Joseph, E.; Smith, I.; Tunge, J. A. Cobalt-Catalyzed Decarboxylative Difluoroalkylation of Nitrophenylacetic Acid Salts. *Chem. Sci*. **2023**, *14*, 13902–13907.
13. Ismalaj, E.; Le Bars, D.; Billard, T. Direct Electrophilic (Benzenesulfonyl)Difluoromethylthiolation with a Shelf-Stable Reagent. *Angew. Chem. Int. Ed*. **2016**, *55*, 4790–4793.
14. Serafino, A.; Pierre, H.; Le Vaillant, F.; Boutet, J.; Guillamot, G.; Neuville, L.; Masson, G. Visible-Light-Driven Decarboxylative Borylation: Rapid Access to *α*- and *β*-Amino-Boronamides. *Org. Lett.* **2023**, *25*, 9249–9254.
15. Zhang, M.; Yu, M.; Wang, Z.; Liu, Y.; Wang, Q. Rapid Access to Aliphatic Sulfonamides. *Org. Lett.* **2022**, *24*, 3932–3937.
16. Kim, I.; Im, H.; Lee, H.; Hong, S. N-Heterocyclic Carbene-Catalyzed Deaminative Cross-Coupling of Aldehydes with Katritzky Pyridinium Salts. *Chem. Sci.* **2020**, *11*, 3192–3197.
17. Hoerrner, M. E.; Baker, K. M.; Basch, C. H.; Bampo, E. M.; Watson, M. P. Deaminative Arylation of Amino Acid-Derived Pyridinium Salts. *Org. Lett.* **2019**, *21*, 7356–7360.
18. Qi, X. K.; Zheng, M. J.; Yang, C.; Zhao, Y.; Guo, L.; Xia, W. Metal-Free Amino(Hetero)Arylation and Aminosulfonylation of Alkenes Enabled by Photoinduced Energy Transfer. *J. Am. Chem. Soc.* **2023**, *145*, 16630–16641.
19. Scheidt, F.; Neufeld, J.; Schäfer, M.; Thiehoff, C.; Gilmour, R. Catalytic Geminal Difluorination of Styrenes for the Construction of Fluorine-Rich Bioisosteres. *Org. Lett.* **2018**, *20*, 8073–8076.

20. Jang, Y. J.; An, H.; Choi, S.; Hong, J.; Lee, S. H.; Ahn, K. H.; You, Y.; Kang, E. J. Green-Light-Driven Fe(III)(Btz)3Photocatalysis in the Radical Cationic [4+2] Cycloaddition Reaction. *Org. Lett.* **2022**, *24*, 4479–4484.
21. Li, J. L.; Liu, Y. Q.; Zou, W. L.; Zeng, R.; Zhang, X.; Liu, Y.; Han, B.; He, Y.; Leng, H. J.; Li, Q. Z. Radical Acylfluoroalkylation of Olefins through *N*-Heterocyclic Carbene Organocatalysis. *Angew. Chem. Int. Ed*. **2020**, *59*, 1863–1870.
22. Ishii, T.; Ota, K.; Nagao, K.; Ohmiya, H. *N*-Heterocyclic Carbene-Catalyzed Radical Relay Enabling Vicinal Alkylacylation of Alkenes. *J. Am. Chem. Soc.* **2019**, *141*, 14073–14077.
23. Ishii, T.; Kakeno, Y.; Nagao, K.; Ohmiya, H. *N*-Heterocyclic Carbene-Catalyzed Decarboxylative Alkylation of Aldehydes. *J. Am. Chem. Soc.* **2019**, *141*, 3854–3858.
24. Wang, J.; Shen, X.; Chen, X.; Bao, Y.; He, J.; Lu, Z. Cobalt-Catalyzed Enantioconvergent Negishi Cross-Coupling of α-Bromoketones. *J. Am. Chem. Soc.* **2023**.
25. Sheldrick, G. M. SHELXT - Integrated Space-Group and Crystal-Structure Determination. *Acta Cryst.* **2015**, *71*, 3–8.
26. Dolomanov, O. V.; Bourhis, L. J.; Gildea, R. J.; Howard, J. A. K.; Puschmann, H. OLEX2: A Complete Structure Solution, Refinement and Analysis Program. *J. Appl. Cryst*. **2009**, *42*, 339–341.
27. Bourhis, L. J.; Dolomanov, O. V; Gildea, R. J.; Howard, J. A. K.; Puschmann, H. The Anatomy of a Comprehensive Constrained, Restrained Refinement Program for the Modern Computing Environment - Olex2 Dissected. *Acta. Cryst. A* **2015**, *71*, 59–75.
28. CrysAlisPro Software System, Rigaku Oxford Diffraction, **2024**.
29. Gaussian 09, Revision D.01, Frisch, M. J.; Trucks, G. W.; Schlegel, H. B.; Scuseria, G. E.; Robb, M.A.; Cheeseman, J. R.; Scalmani, G.; Barone, V.; Mennucci, B.; Petersson, G. A.; Nakatsuji, H.;Caricato, M.; Li, X.; Hratchian, H. P.; Izmaylov, A. F.; Bloino, J.; Zheng, G.; Sonnenberg, J. L.; Hada,M.; Ehara, M.; Toyota, K.; Fukuda, R.; Hasegawa, J.; Ishida, M.; Nakajima, T.; Honda, Y.; Kitao, O.;Nakai, H.; Vreven, T.; Montgomery, Jr., J. A.; Peralta, J. E.; Ogliaro, F.; Bearpark, M.; Heyd, J. J.;Brothers, E.; Kudin, K. N.; Staroverov, V. N.; Keith, T.; Kobayashi, R.; Normand, J.; Raghavachari,K.; Rendell, A.; Burant, J. C.; Iyengar, S. S.; Tomasi, J.; Cossi, M.; Rega, N.; Millam, J. M. ; Klene,M.; Knox, J. E.; Cross, J. B.; Bakken, V.; Adamo, C.; Jaramillo, J.; Gomperts, R.; Stratmann, R. E.;Yazyev, O.; Austin, A. J.; Cammi, R.; Pomelli, C.; Ochterski, J. W.; Martin, R. L.; Morokuma, K.;Zakrzewski, V. G.; Voth, G. A.; Salvador, P.; Dannenberg, J. J.; Dapprich, S.; Daniels, A. D.; Farkas,O.; Foresman, J. B.; Ortiz, J. V.; Cioslowski, J.; Fox, D. J. Gaussian, Inc., Wallingford CT, **2013**.
30. (a) Becke, A. D. Density-functional thermochemistry. III. The role of exact exchange. *J. Chem. Phys.* **1993**. 98, 5648-5652. (b) Lee, C.; Yang, W.; Parr, R. G. Development of the Colle-Salvetti correlation-energy formula into a functional of the electron density. *Phys. Rev. B* **1988**, *37*, 785–789.
31. Weigend, F.; Ahlrichs, R. Balanced Basis Sets of Split Valence, Triple Zeta Valence and Quadruple Zeta Valence Quality for H to Rn: Design and Assessment of Accuracy. *Phys. Chem. Chem. Phys*. **2005**, *7*, 3297–3305.
32. Grimme, S.; Antony, J.; Ehrlich, S.; Krieg, H. A Consistent and Accurate Ab Initio Parametrization of Density Functional Dispersion Correction (DFT-D) for the 94 Elements H-Pu. *J. Chem. Phys*. **2010**, *132*, 154104.

NMR spectra

2,6-Bis(1-(3,5-dimethylphenyl)vinyl)-4-(trifluoromethyl)aniline (ac)

^{1}**H NMR** (400 MHz, Chloroform-*d*):

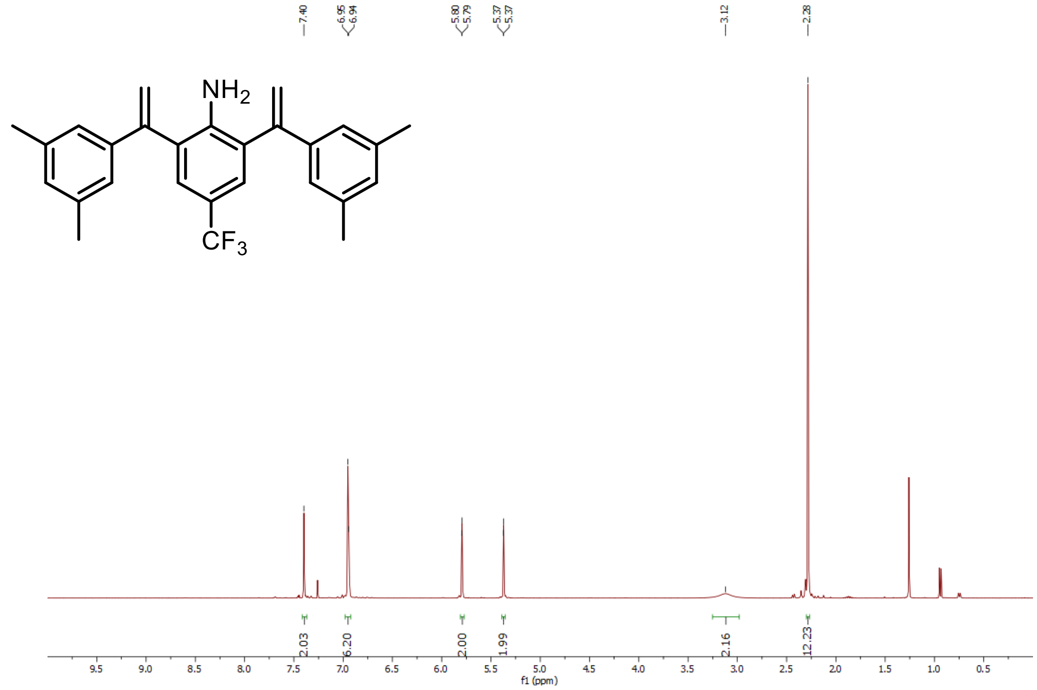

^{13}**C NMR** (101 MHz, Chloroform-*d*):

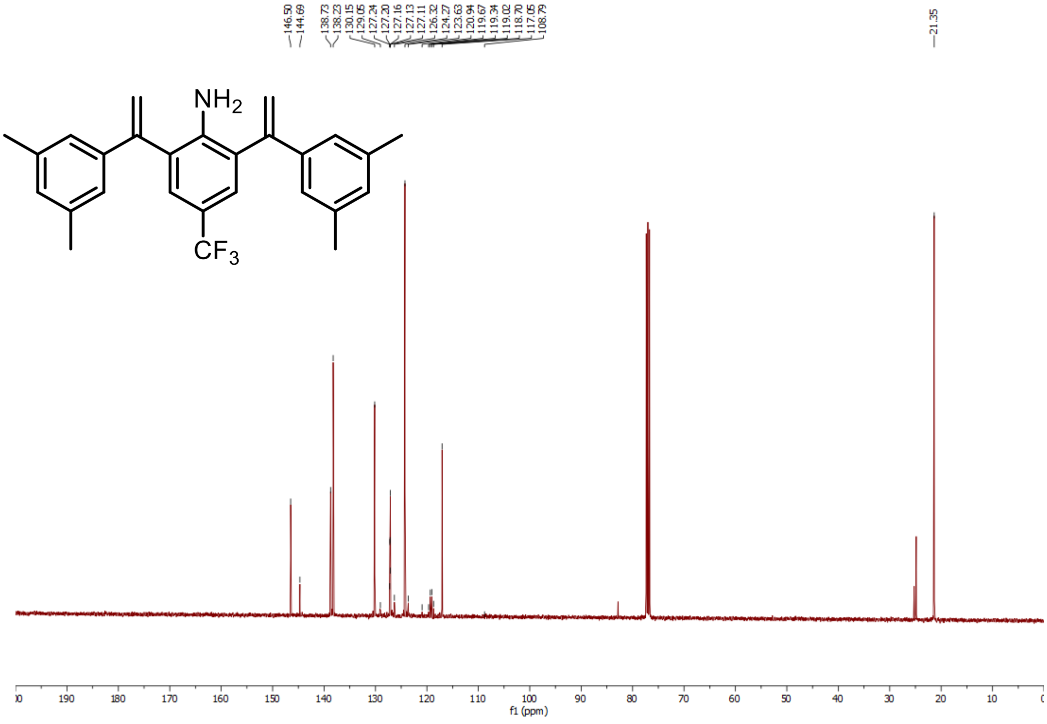

^{19}F NMR (376 MHz, Chloroform-*d*):

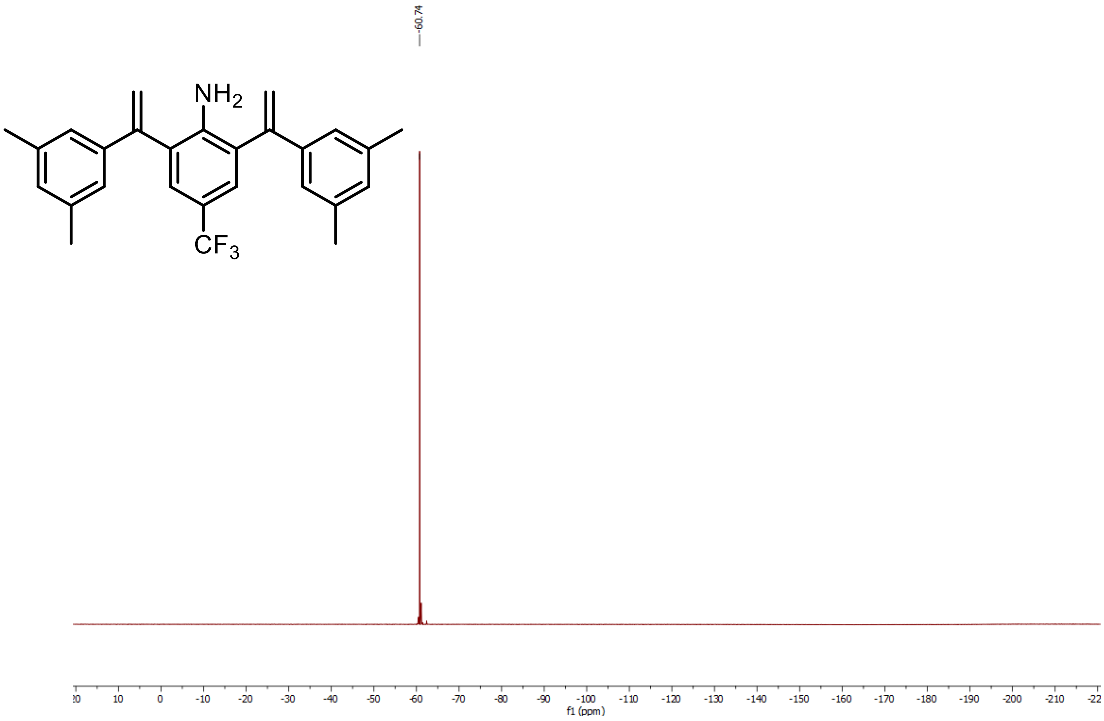

2,6-Bis(1-(3,5-di-*tert*-butylphenyl)vinyl)-4-(trifluoromethyl)aniline (ad)

^{1}H NMR (400 MHz, Chloroform-*d*):

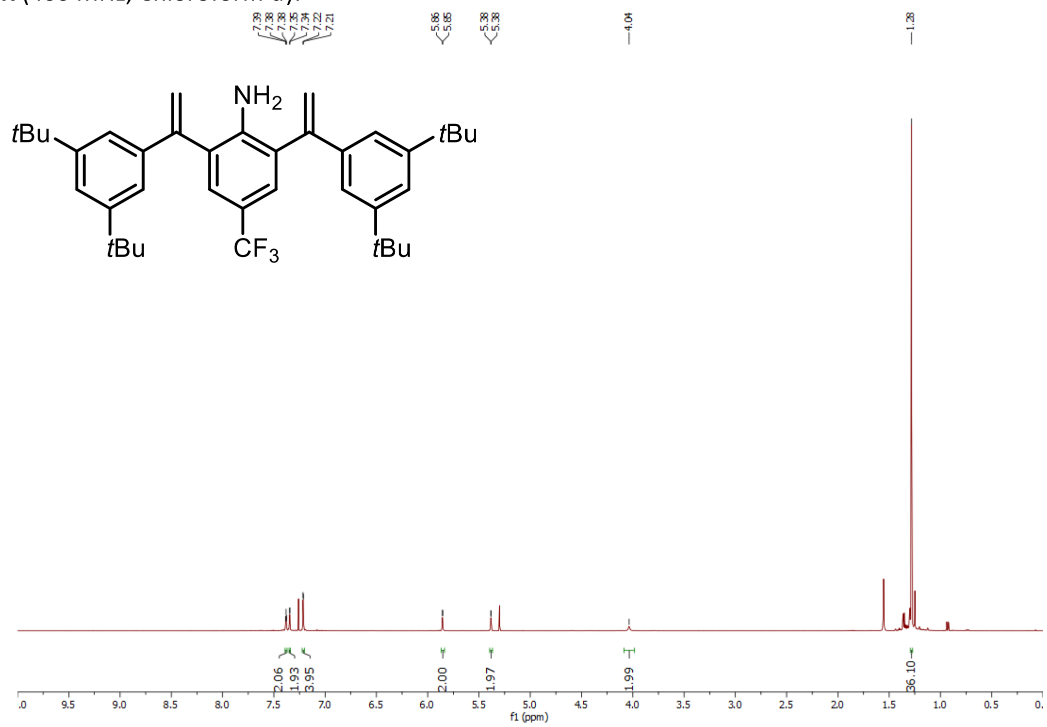

^{13}C NMR (101 MHz, Chloroform-*d*):

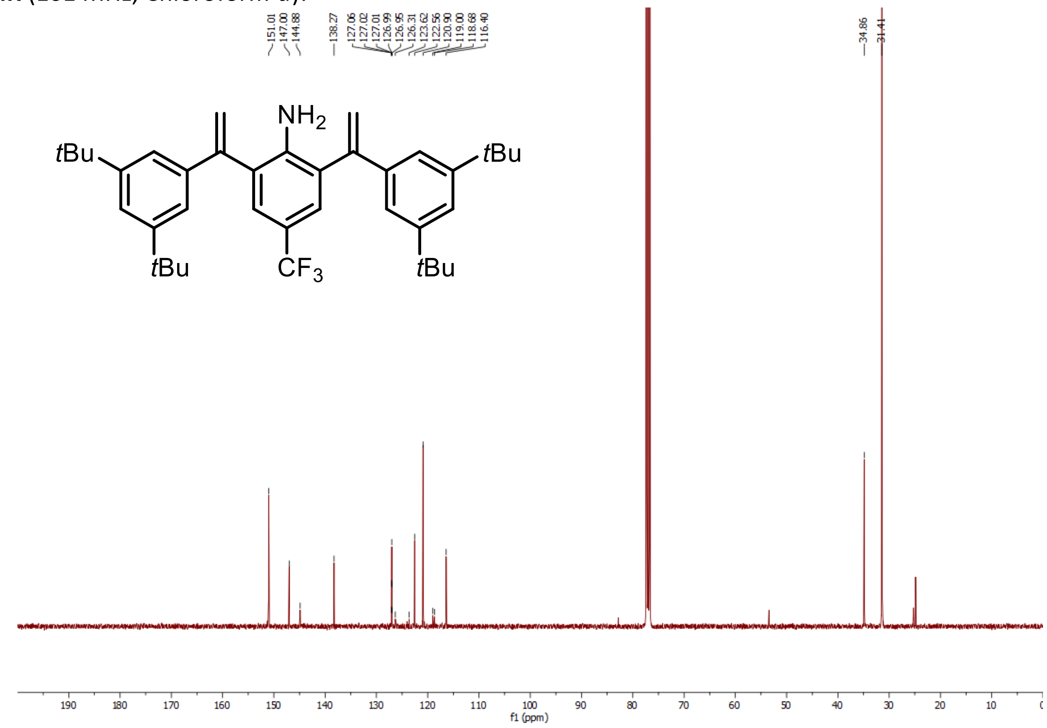

^{19}F NMR (376 MHz, Chloroform-*d*):

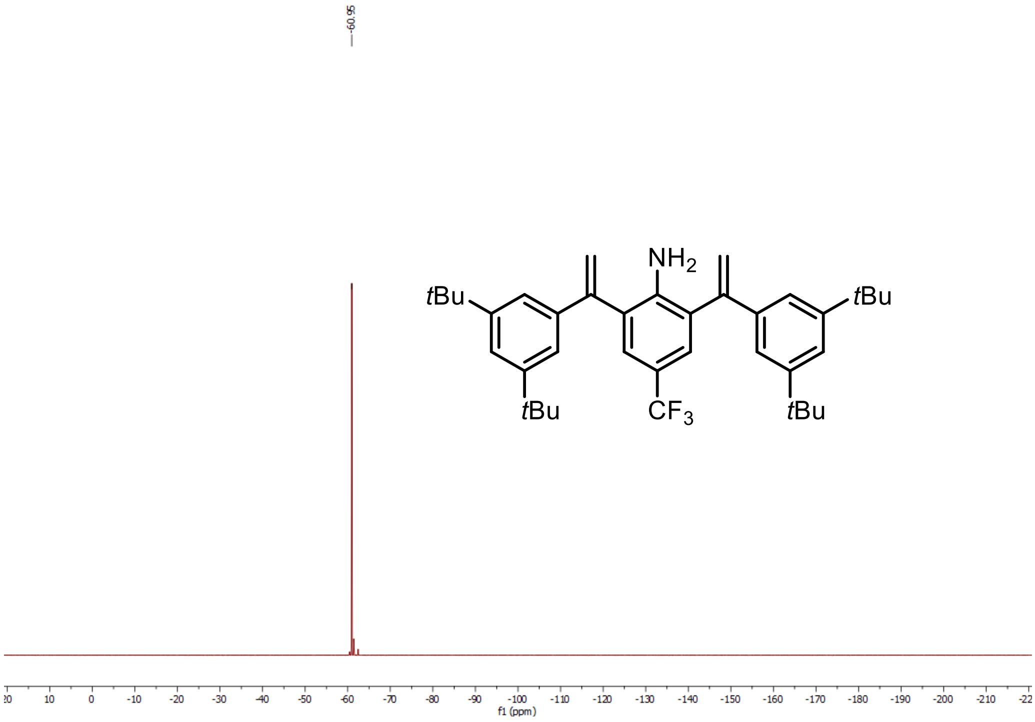

2,6-Bis((*R*)-1-(3,5-dimethylphenyl)ethyl)-4-(trifluoromethyl)aniline (be)

^{1}H NMR (400 MHz, Chloroform-*d*):

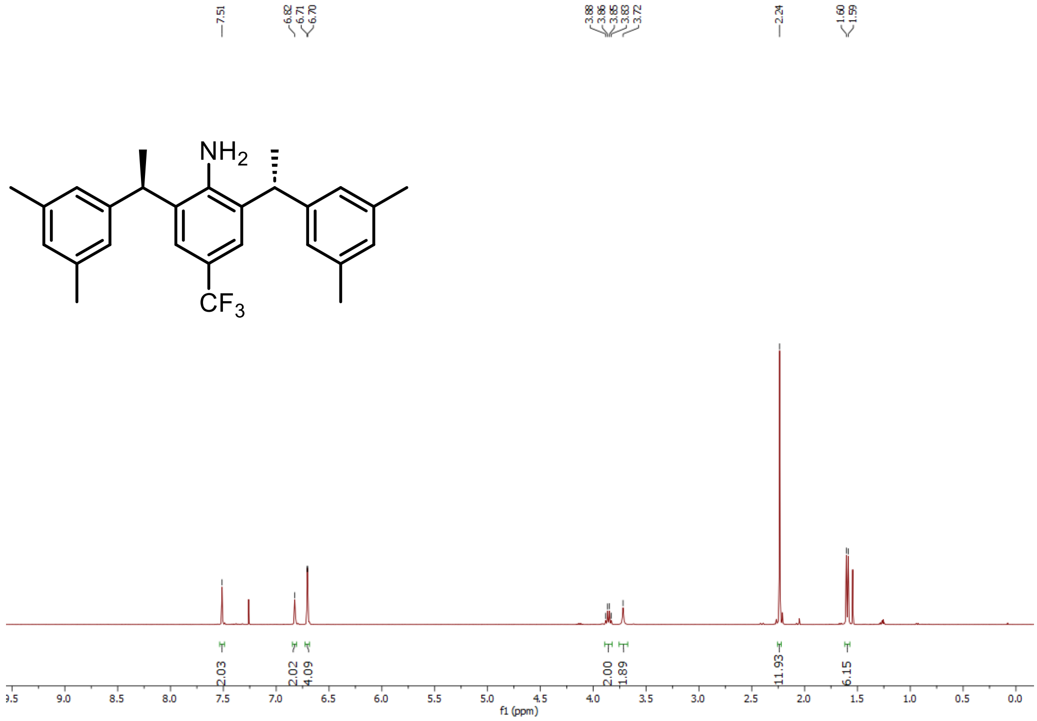

^{13}C NMR (101 MHz, Chloroform-*d*):

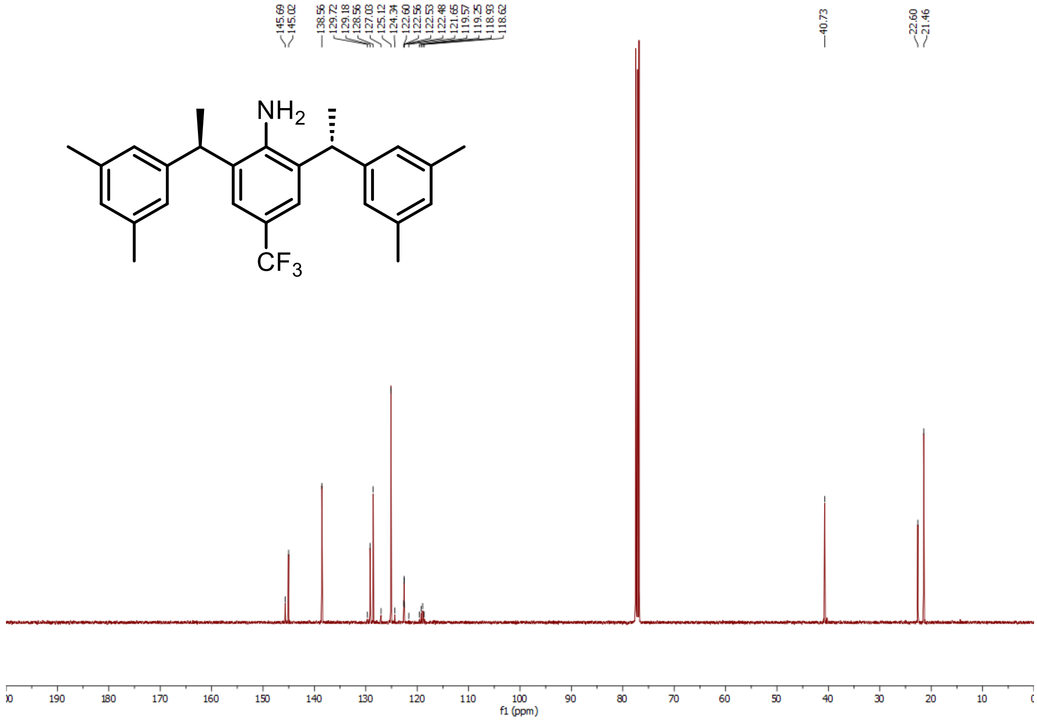

^{19}F NMR (376 MHz, Chloroform-*d*):

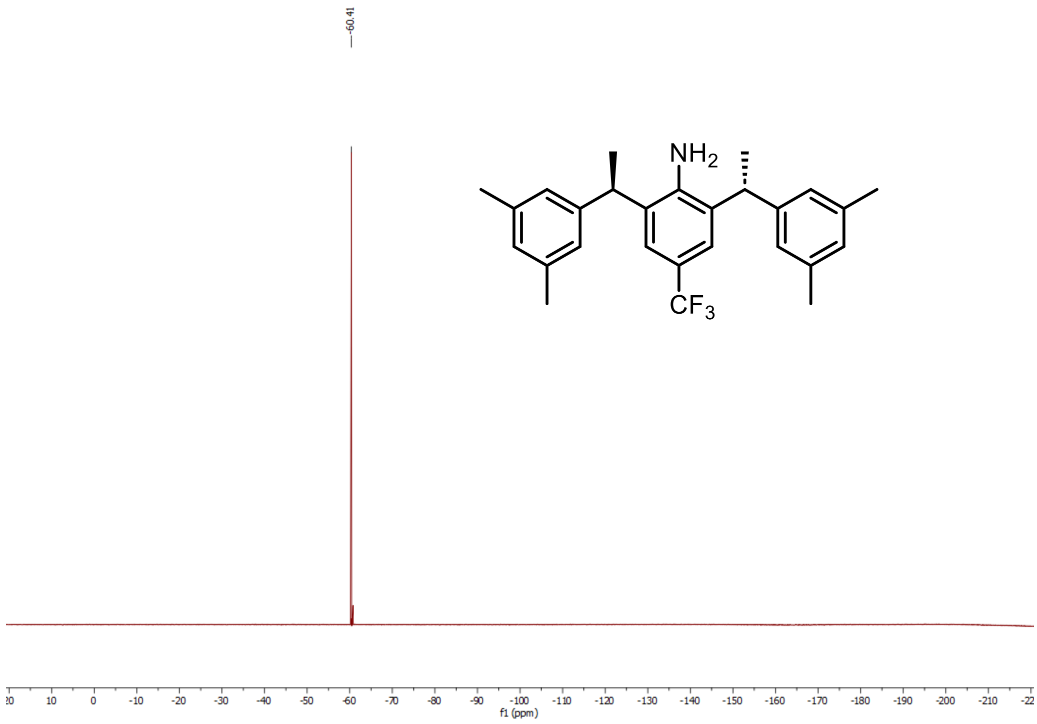

2,6-Bis((*R*)-1-(3,5-di-*tert*-butylphenyl)ethyl)-4-(trifluoromethyl)aniline (bf)

^{1}H NMR (400 MHz, Chloroform-*d*):

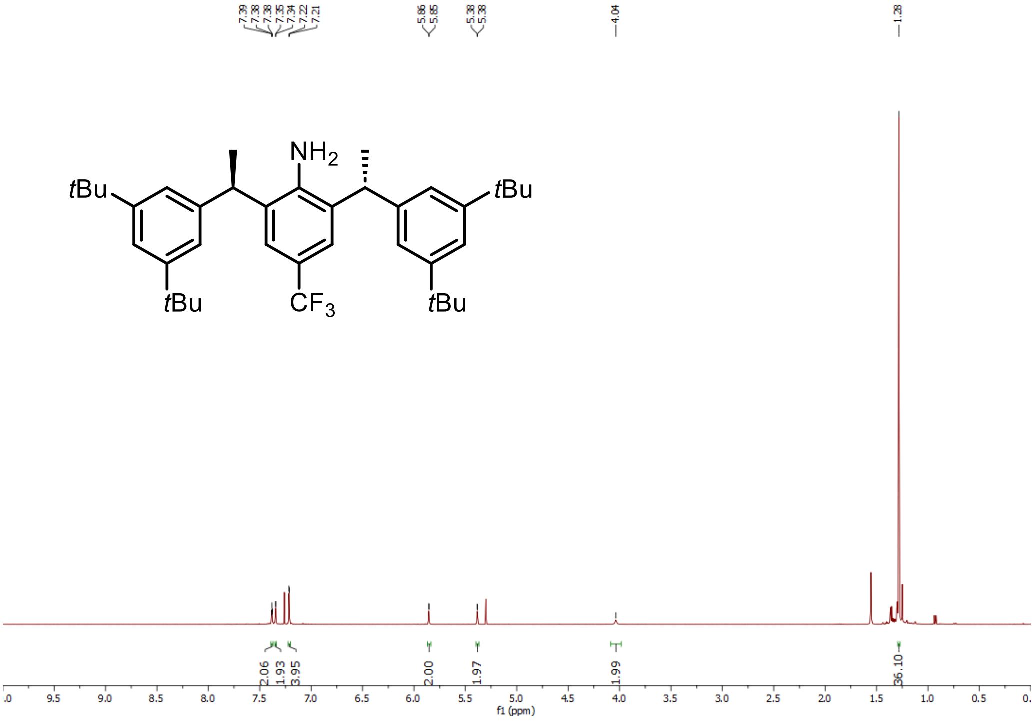

^{13}C NMR (101 MHz, Chloroform-*d*):

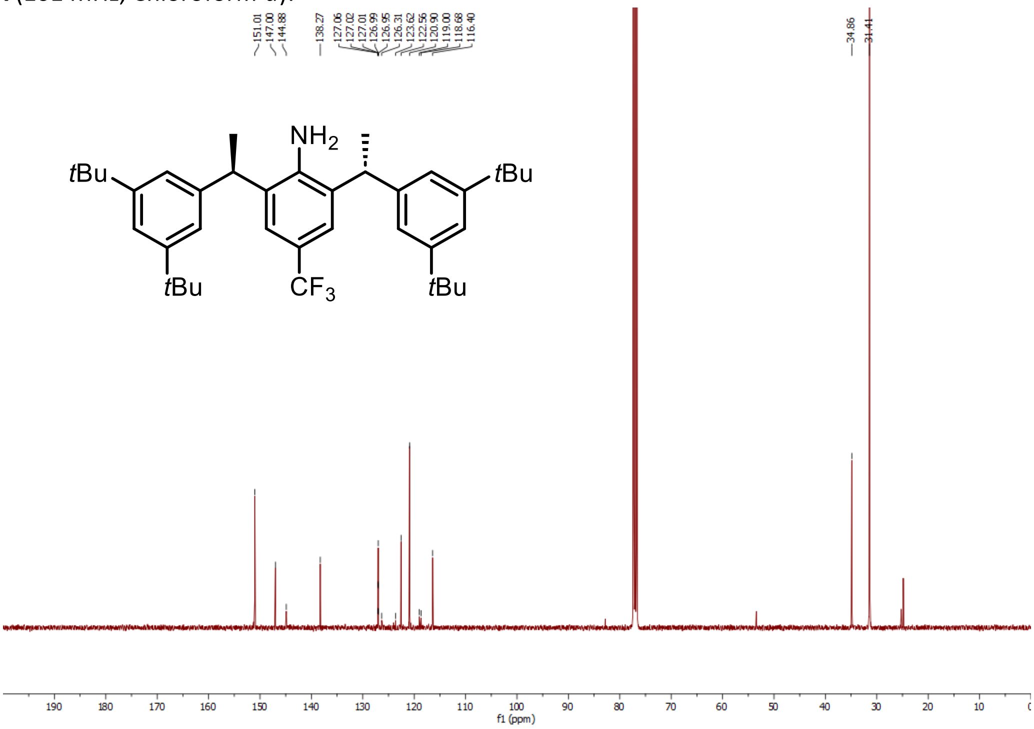

^{19}F NMR (376 MHz, Chloroform-*d*):

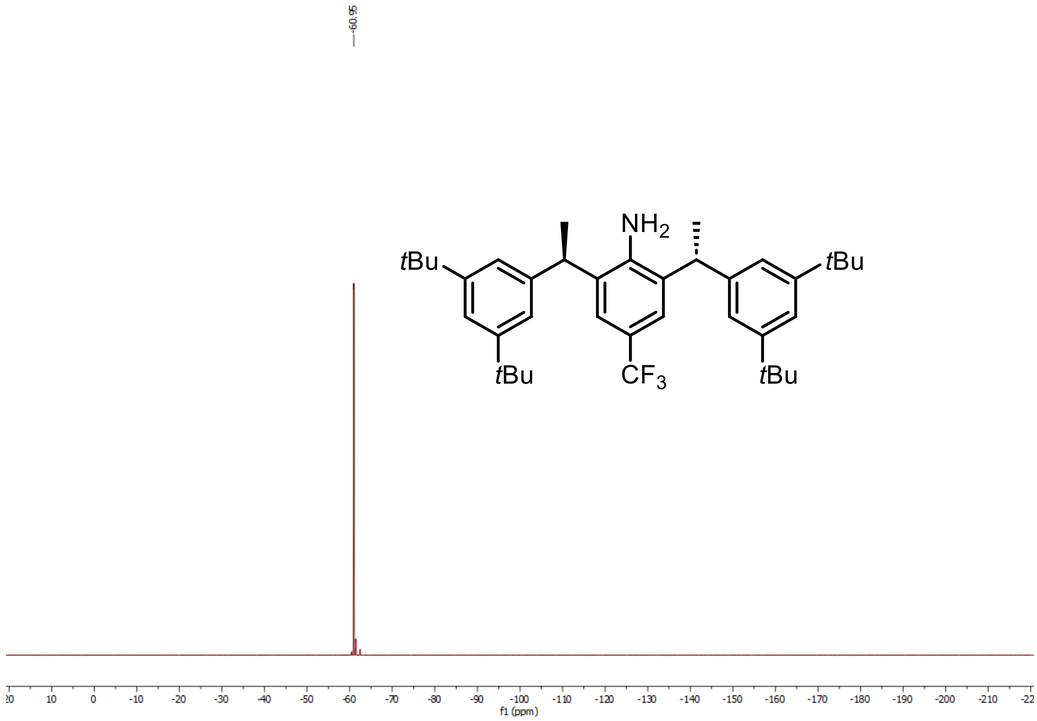

3-(2,6-Bis((*R*)-1-phenylethyl)phenyl)-5,6,7,8-tetrahydro-4*H*-cyclohepta[*d*]thiazol-3-ium perchlorate (NHC1)

^{1}**H NMR** (400 MHz, Chloroform-*d*):

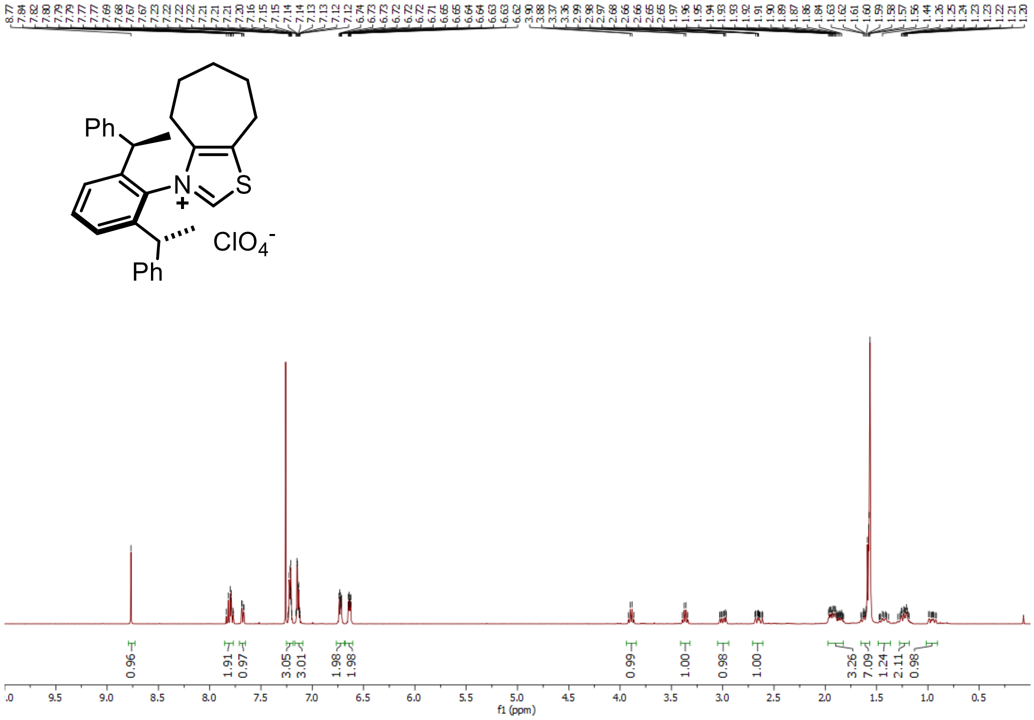

^{13}**C NMR** (101 MHz, Chloroform-*d*):

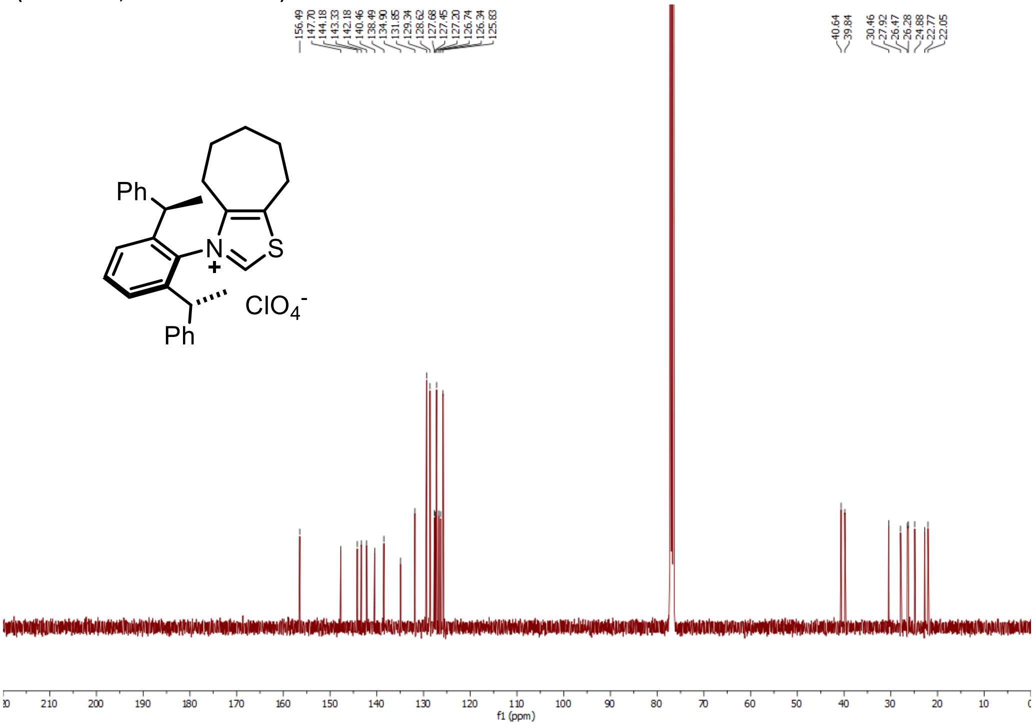

3-(4-Methoxy-2,6-bis((*R*)-1-phenylethyl)phenyl)-5,6,7,8-tetrahydro-4*H*-cyclohepta[*d*]thiazol-3-ium perchlorate (NHC2)

^{1}H NMR (400 MHz, Chloroform-*d*):

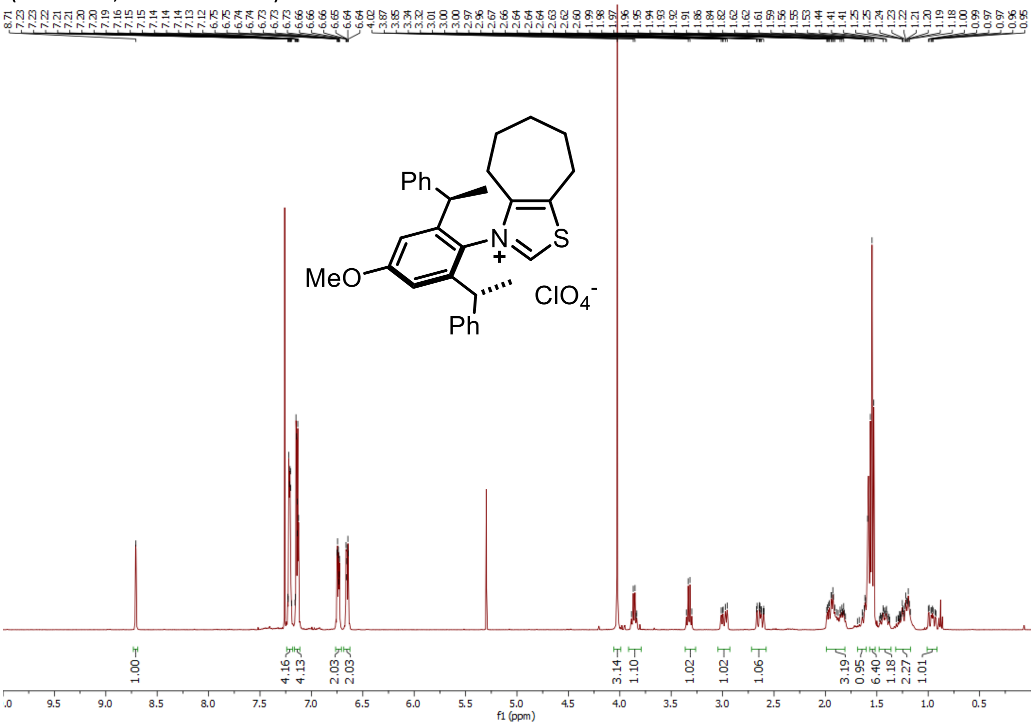

^{13}C NMR (101 MHz, Chloroform-*d*):

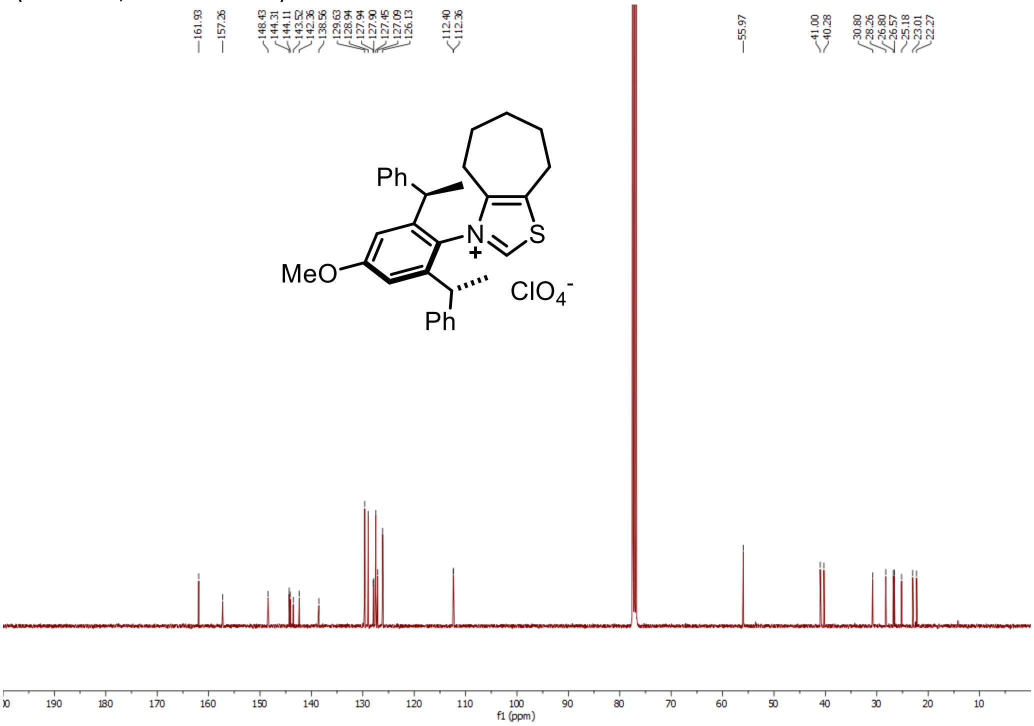

3-(2,6-Bis((*R*)-1-phenylethyl)-4-(trifluoromethyl)phenyl)-5,6,7,8-tetrahydro-*4H*-cyclohepta[*d*]thiazol-3-ium perchlorate (NHC3)

^{1}H NMR (400 MHz, Chloroform-*d*):

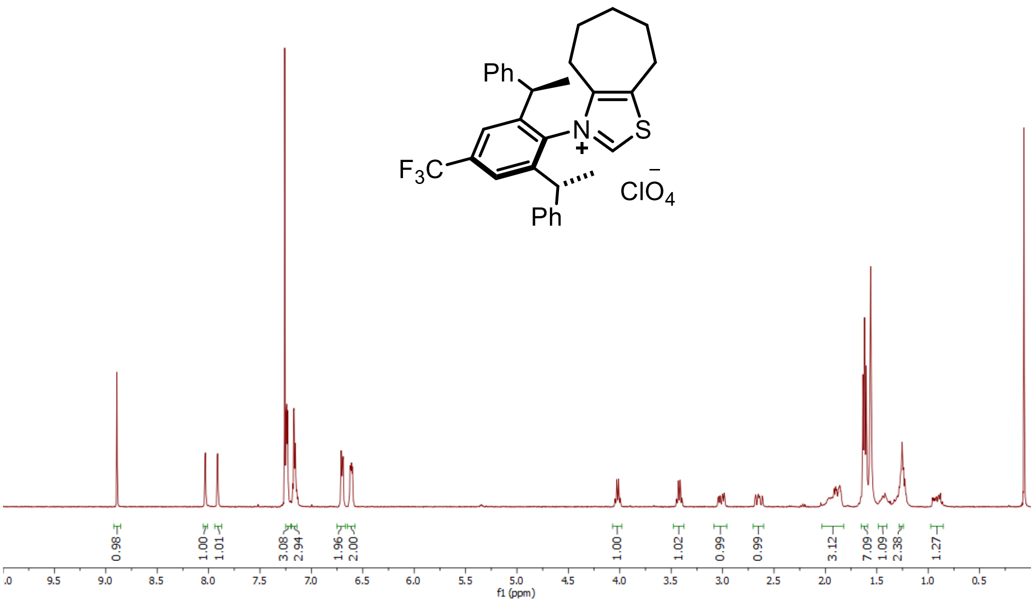

^{13}C NMR (101 MHz, Chloroform-*d*):

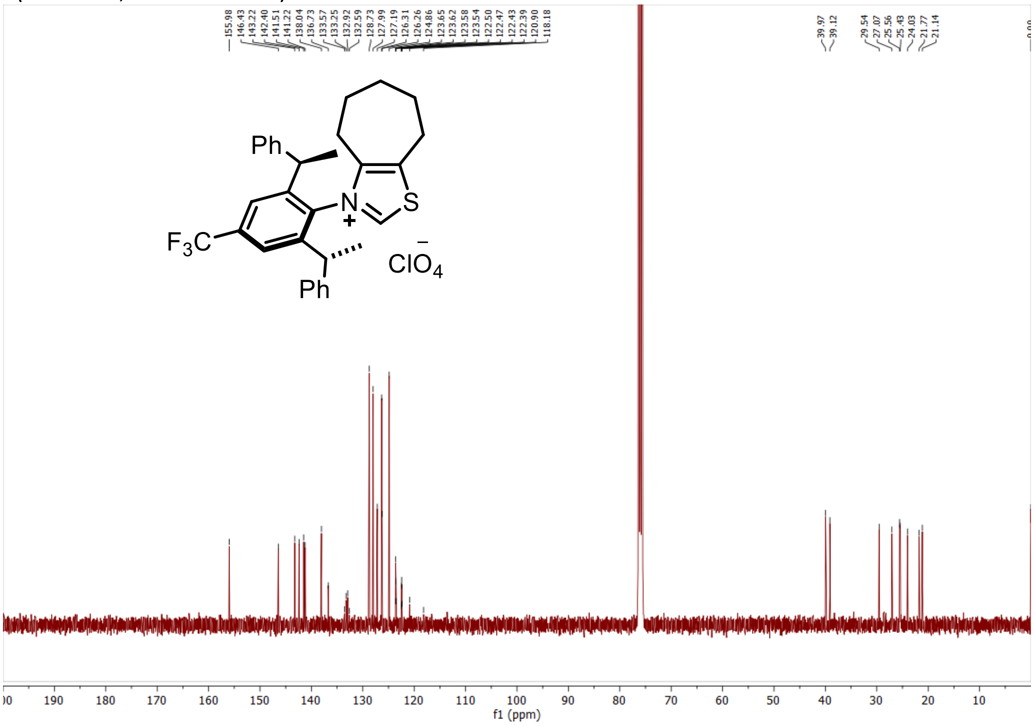

^{19}F NMR (376 MHz, Chloroform-*d*):

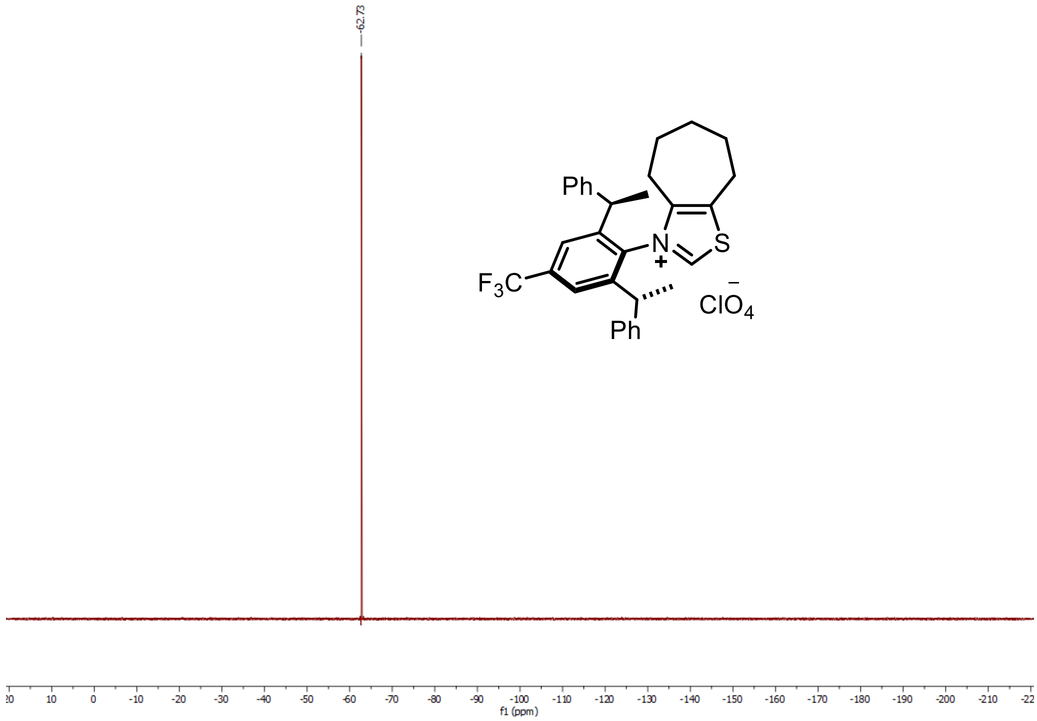

3-(2,6-Bis((*R*)-1-phenylethyl)phenyl)-4,5,6,7,8,9-hexahydrocycloocta[*d*]thiazol-3-ium perchlorate (NHC4)

^{1}H NMR (400 MHz, Chloroform-*d*):

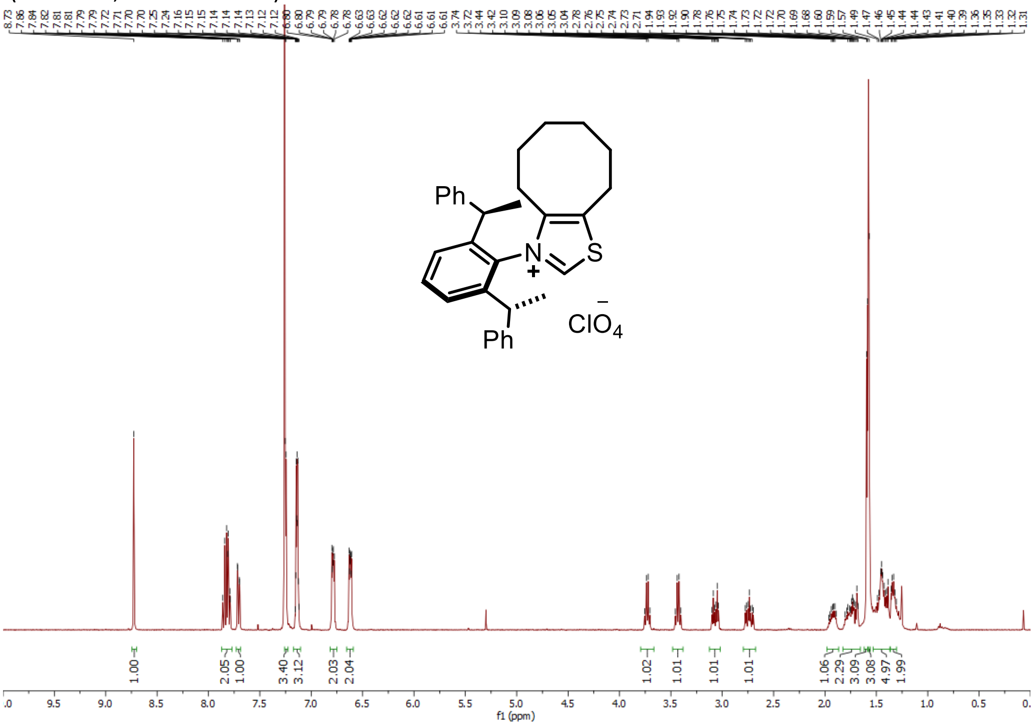

^{13}C NMR (101 MHz, $CDCl_3$):

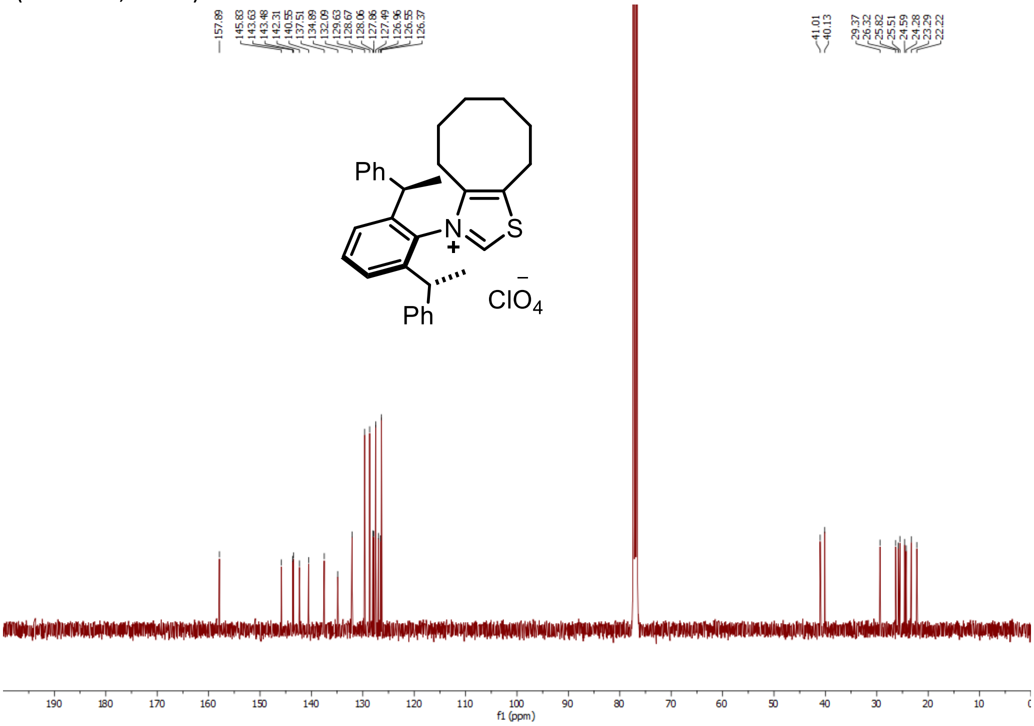

3-(2,6-Bis((*R*)-1-phenylethyl)-4-(trifluoromethyl)phenyl)-4,5,6,7,8,9,10,11,12,13-decahydrocyclododeca[d]thiazol-3-ium perchlorate (NHC5)

^{1}H NMR (400 MHz, Chloroform-*d*):

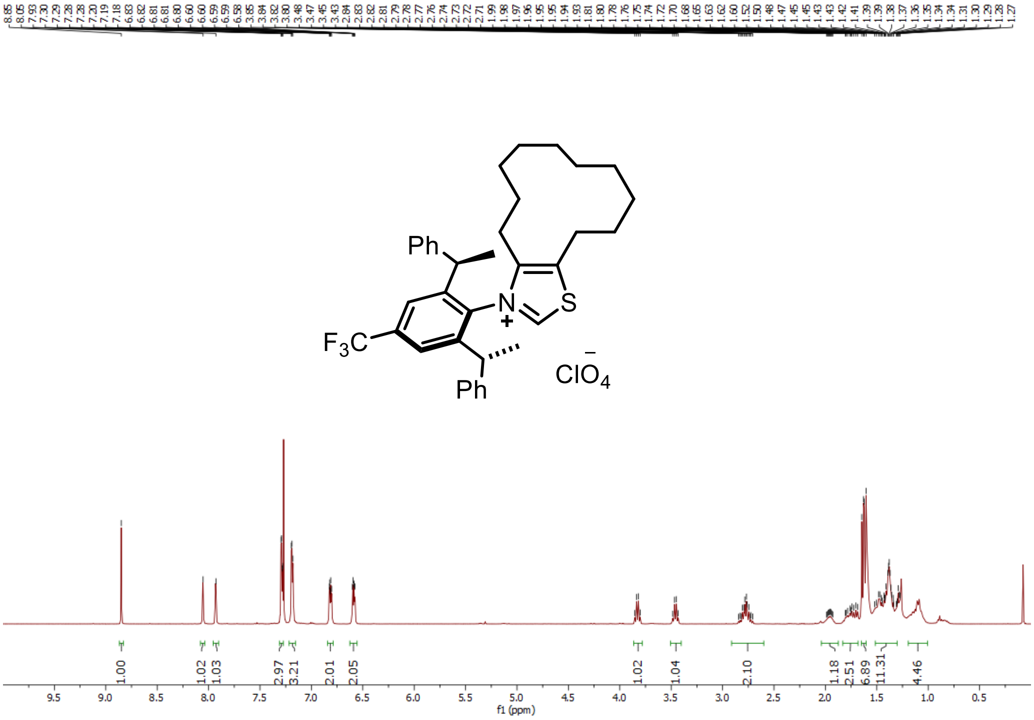

^{13}C NMR (101 MHz, Chloroform-*d*):

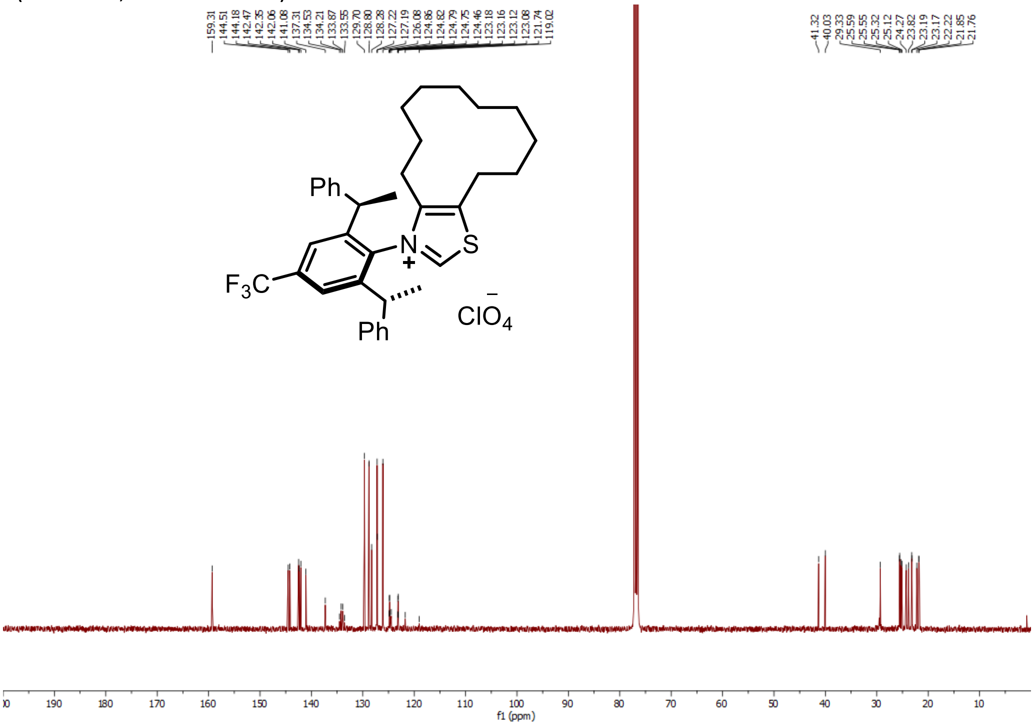

^{19}F NMR (376 MHz, Chloroform-*d*):

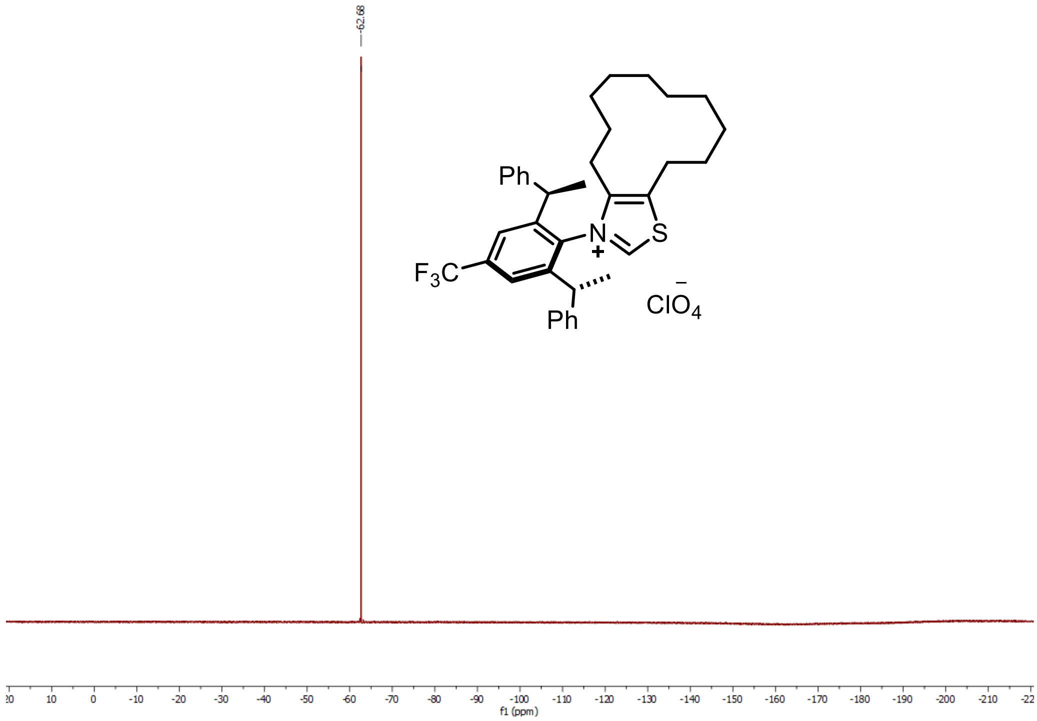

3-(2,6-bis((*R*)-1-(3,5-dimethylphenyl)ethyl)-4-(trifluoromethyl)phenyl)-4,5,6,7,8,9,10,11,12,13-decahydrocyclododeca[d]thiazol-3-ium perchlorate (NHC6)

^{1}H NMR (400 MHz, Chloroform-*d*):

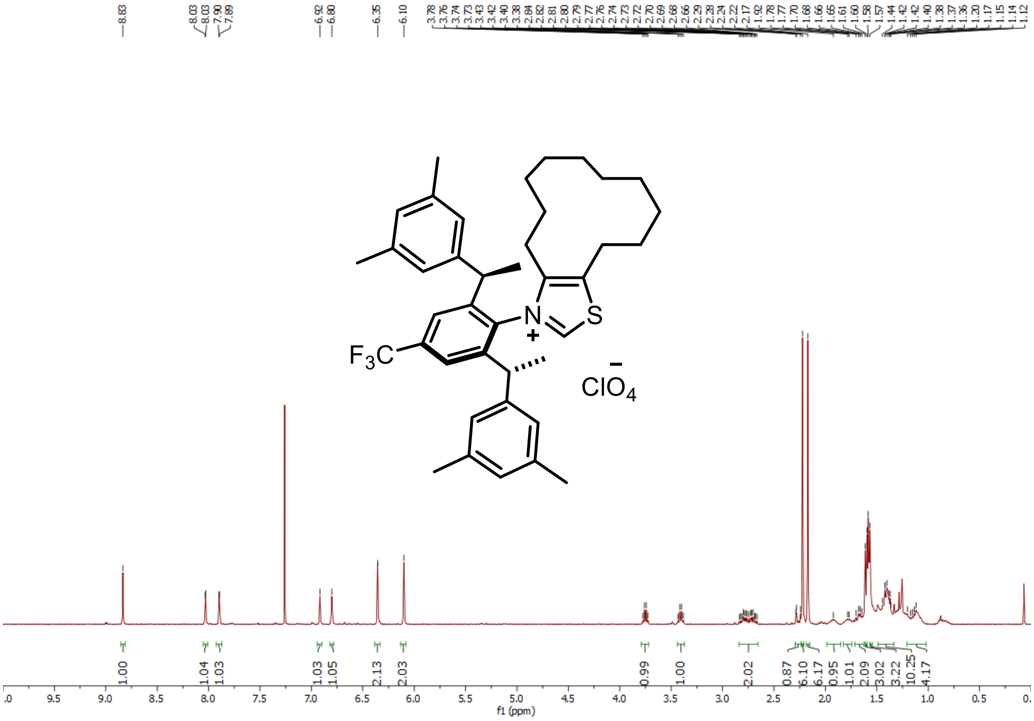

^{13}C NMR (101 MHz, Chloroform-*d*):

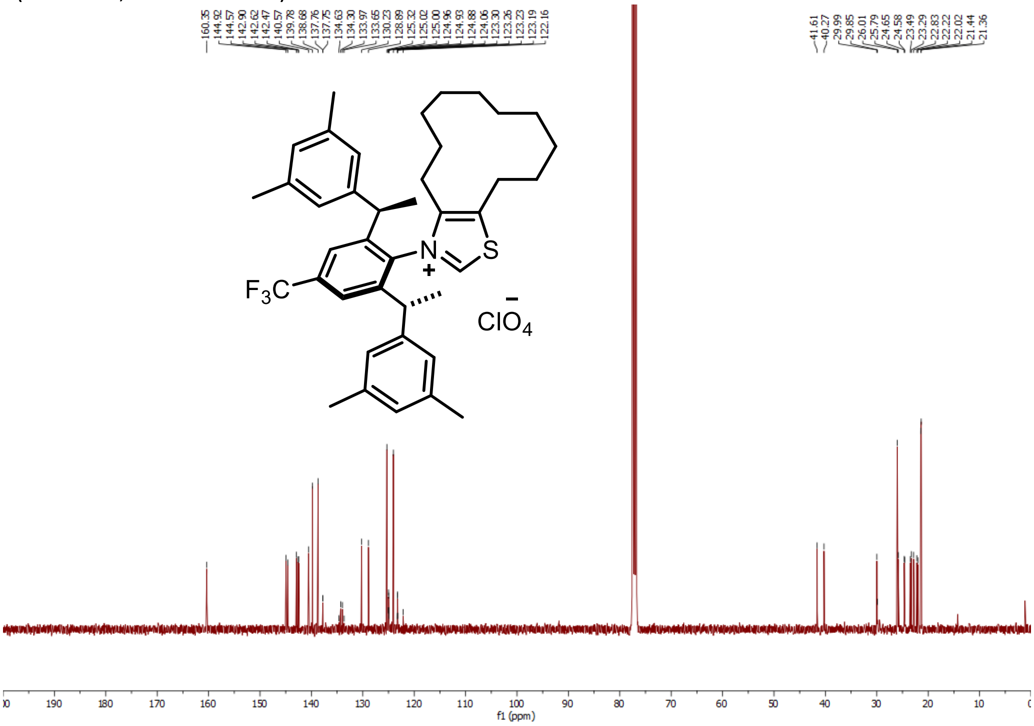

^{19}F NMR (376 MHz, Chloroform-*d*):

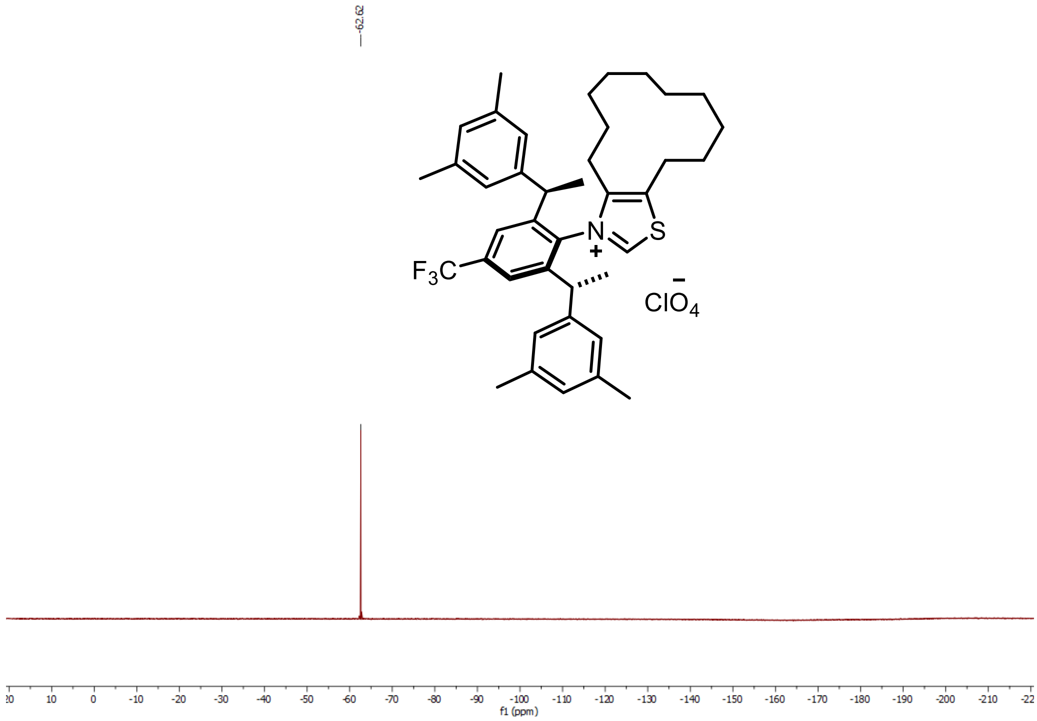

3-(2,6-Bis((*R*)-1-(3,5-di-*tert*-butylphenyl)ethyl)-4-(trifluoromethyl)phenyl)-4,5,6,7,8,9,10,11,12,13-decahydrocyclododeca[*d*]thiazol-3-ium perchlorate (NHC7)

^{1}H NMR (400 MHz, Chloroform-*d*):

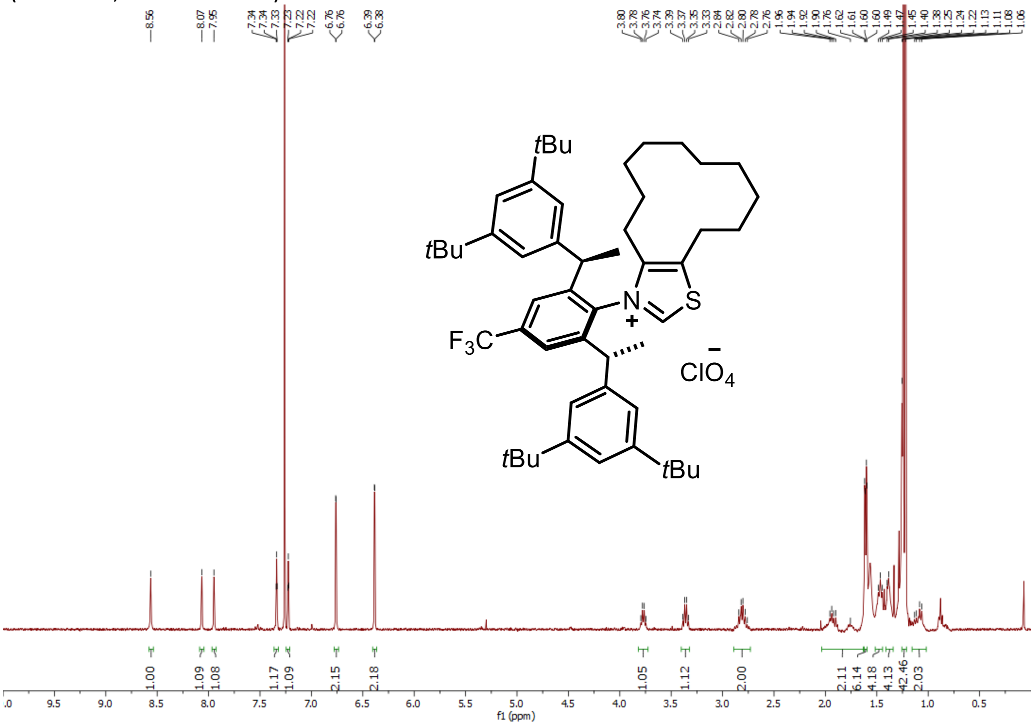

^{13}C NMR (101 MHz, Chloroform-*d*):

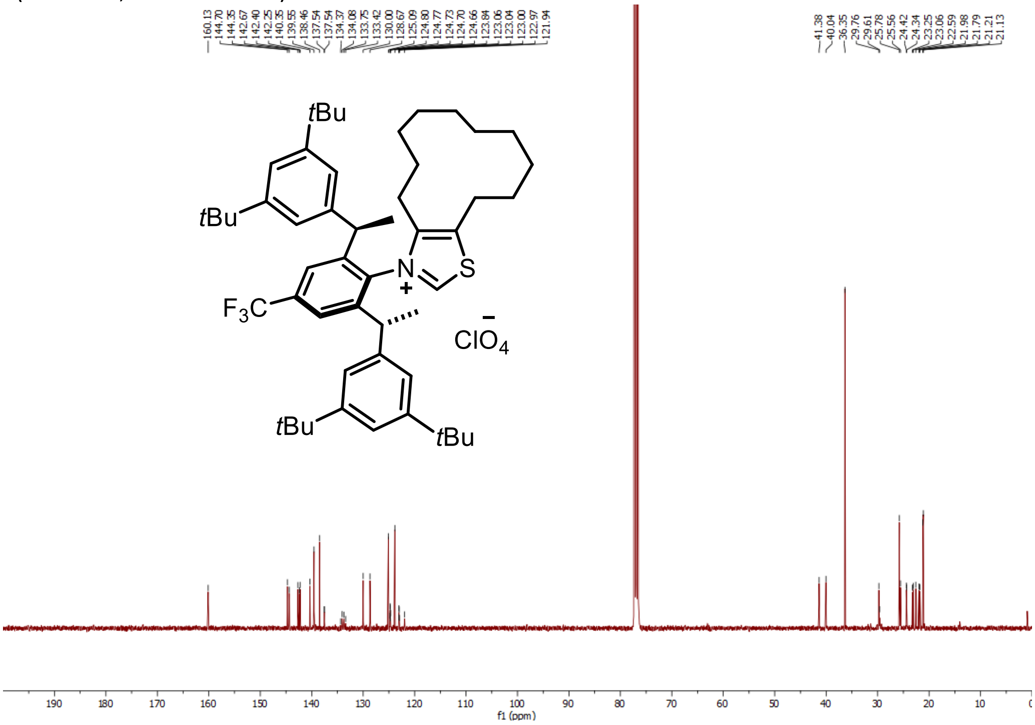

^{19}F NMR (376 MHz, Chloroform-*d*):

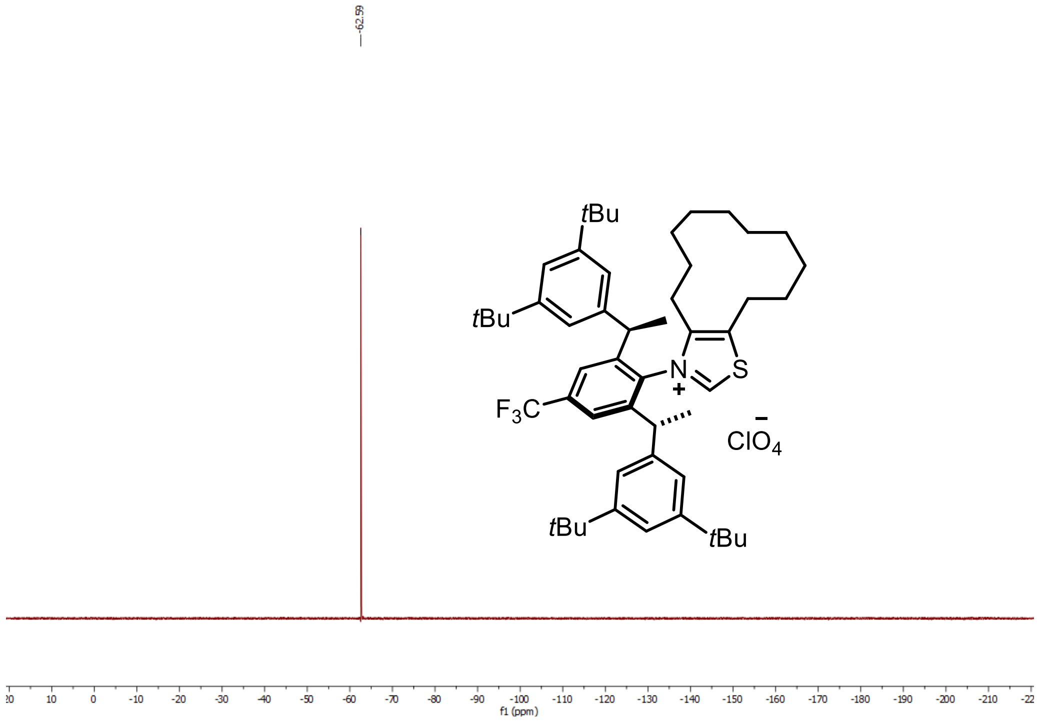

3-(4-Methyl-2,6-bis((*R*)-1-phenylethyl)phenyl)-5,6,7,8-tetrahydro-4H-cyclohepta[*d*]thiazol-3-ium perchlorate (NHC10)

^{1}H NMR (400 MHz, Chloroform-*d*):

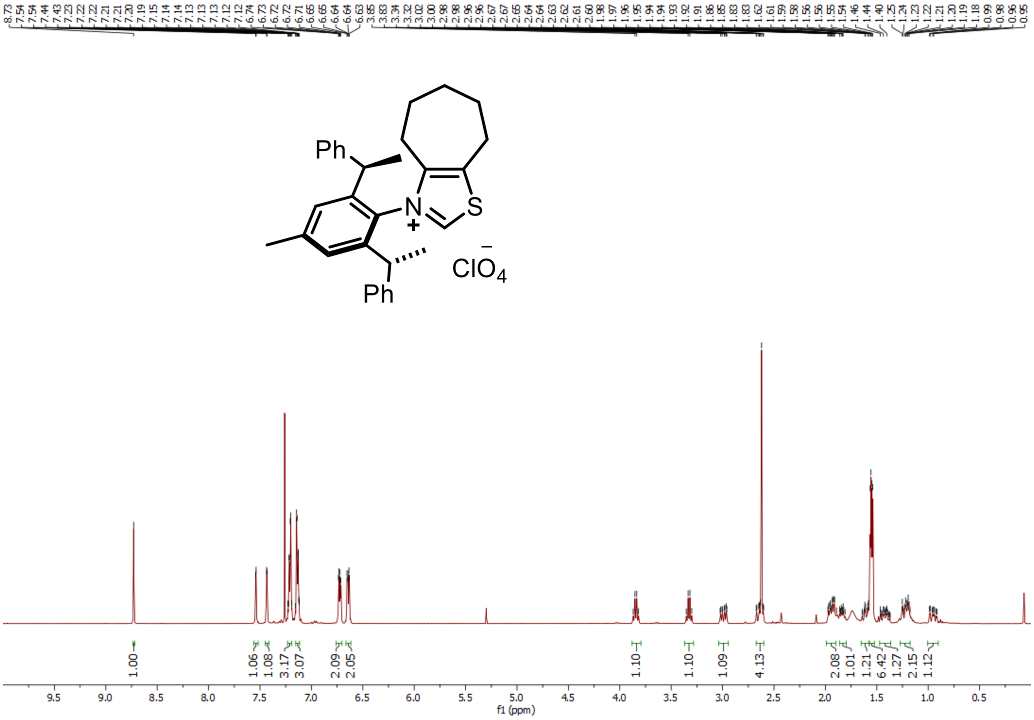

^{13}C NMR (101 MHz, Chloroform-*d*):

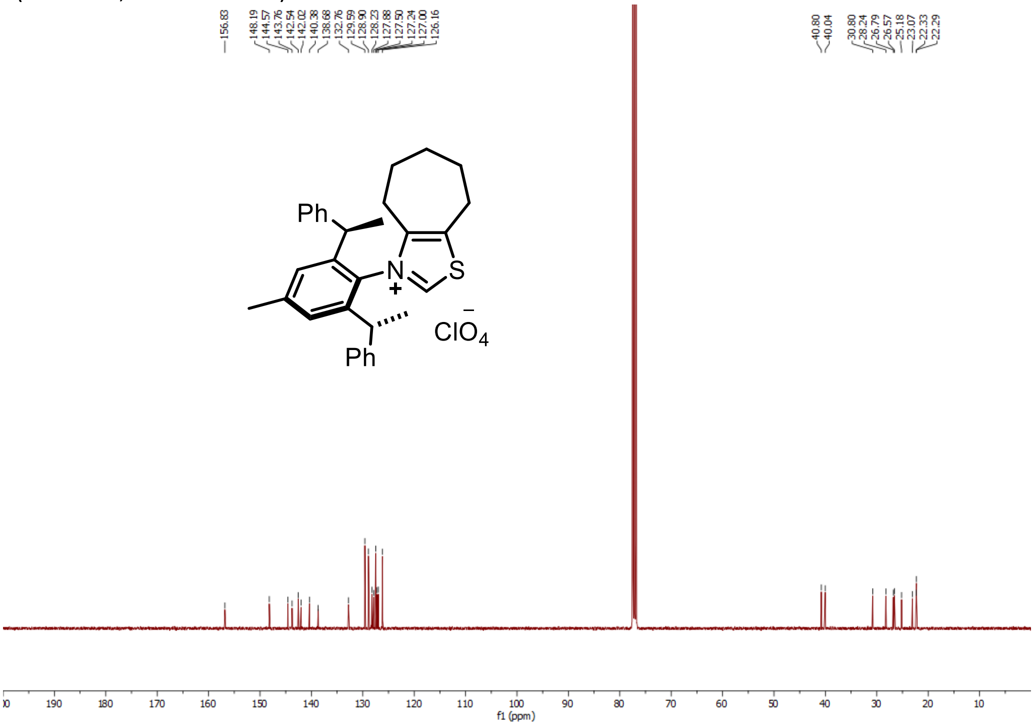

3-(2,6-Bis((*R*)-1-(3,5-dimethylphenyl)ethyl)phenyl)-5,6,7,8-tetrahydro-4*H*-cyclohepta[d]thiazol-3-ium perchlorate (NHC11)

[1]**H NMR** (400 MHz, Chloroform-*d*):

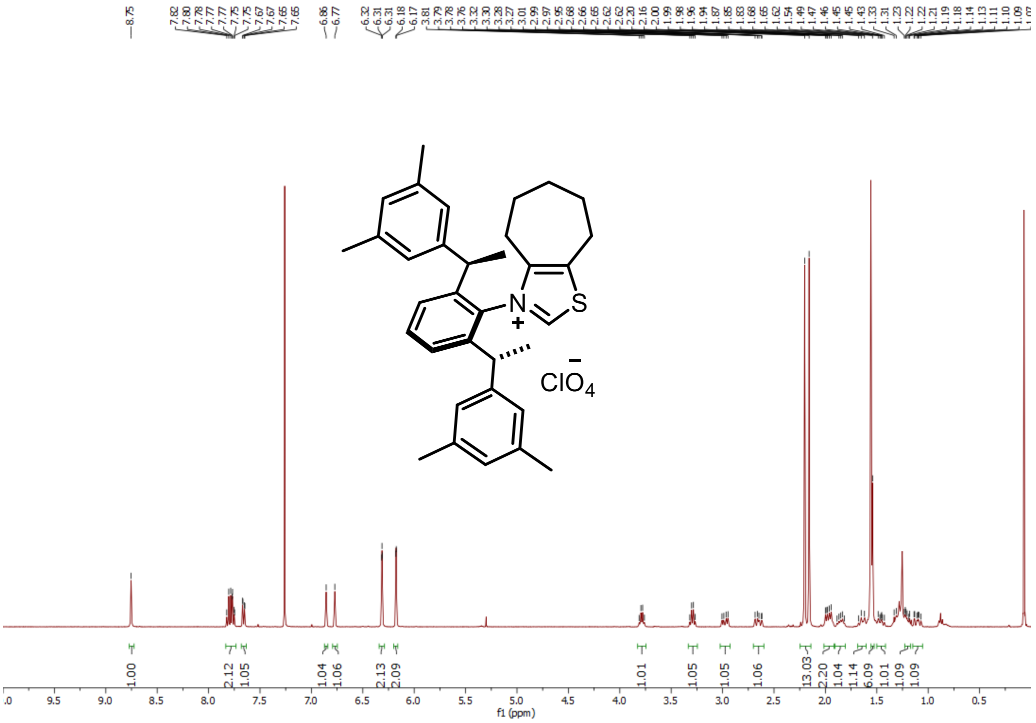

[13]**C NMR** (101 MHz, Chloroform-*d*):

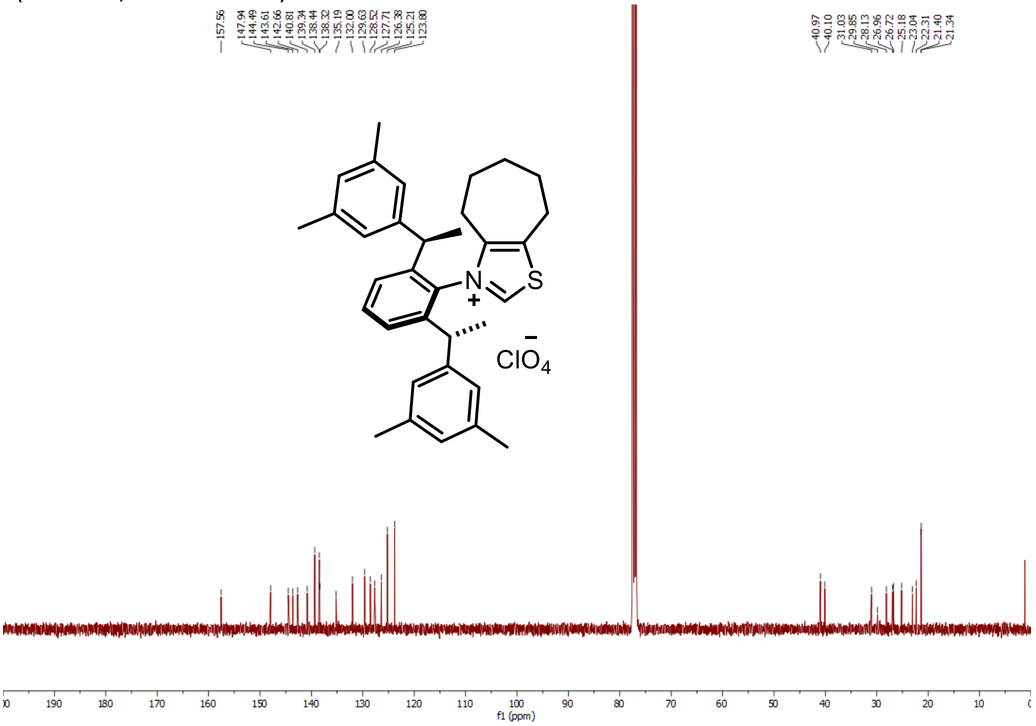

3-(4-Methyl-2,6-bis((*R*)-1-phenylethyl)phenyl)-4,5,6,7,8,9,10,11,12,13-decahydrocyclododeca[d]thiazol-3-ium perchlorate (NHC12)

^{1}**H NMR** (400 MHz, Chloroform-*d*):

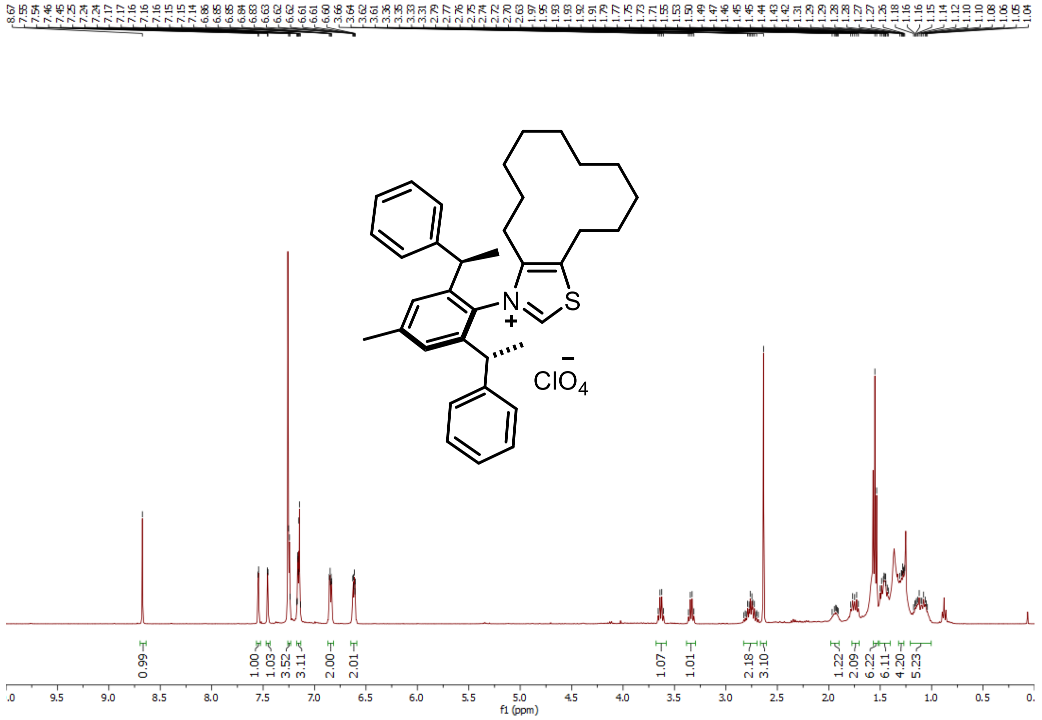

^{13}**C NMR** (101 MHz, Chloroform-*d*):

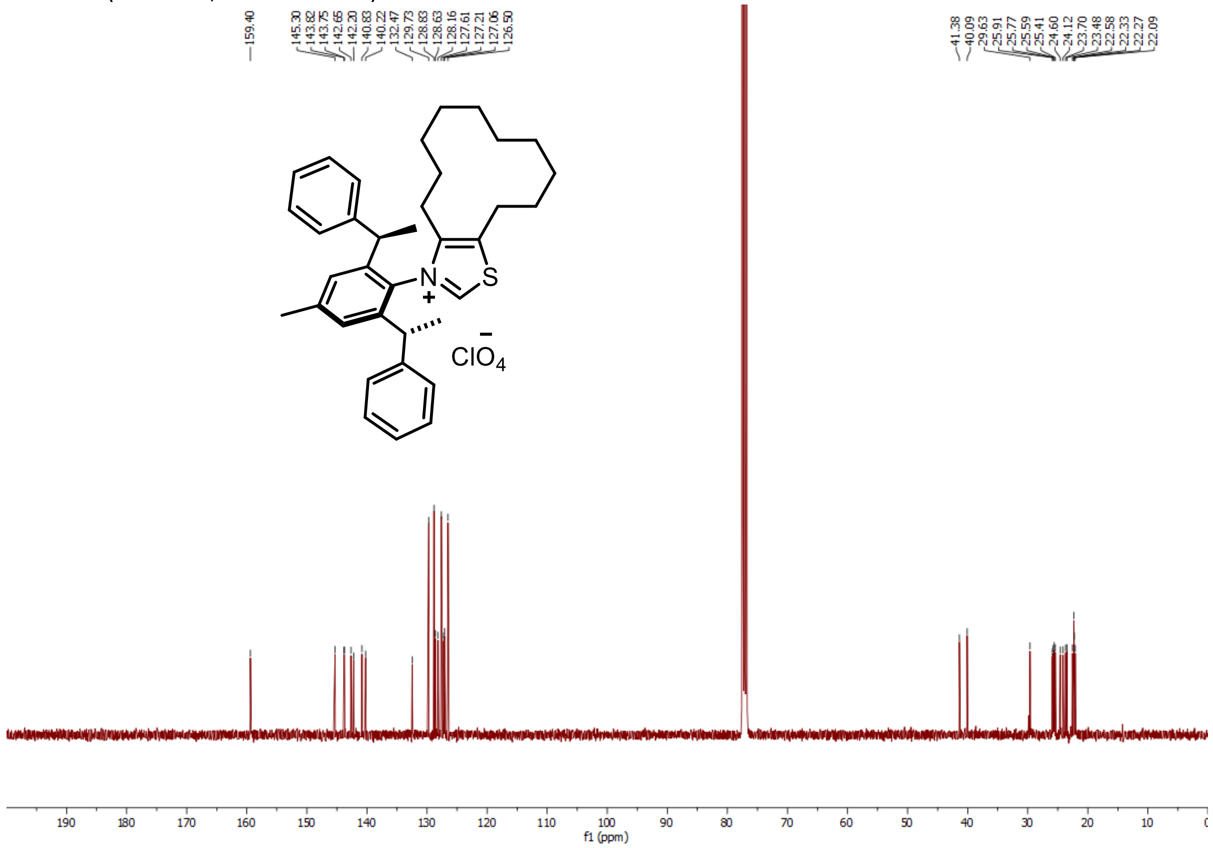

(2,2-Dimethylcyclopropyl)methanol

^{1}H NMR (400 MHz, Chloroform-*d*):

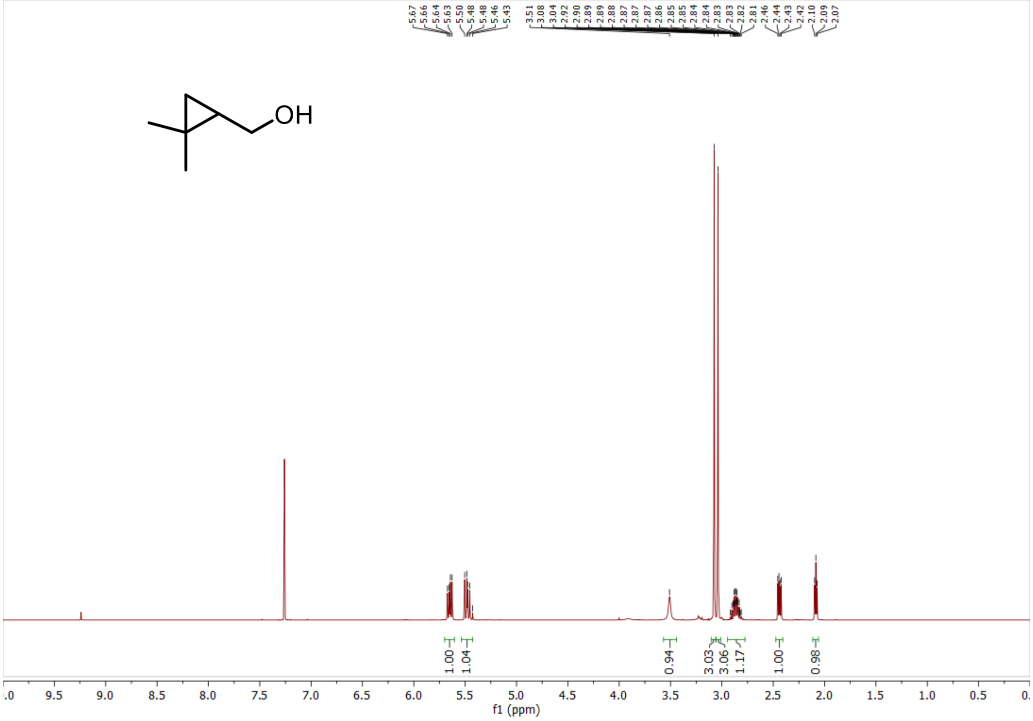

2,2-Dimethylcyclopropane-1-carbaldehyde (1y)

^{1}H NMR (400 MHz, Chloroform-*d*):

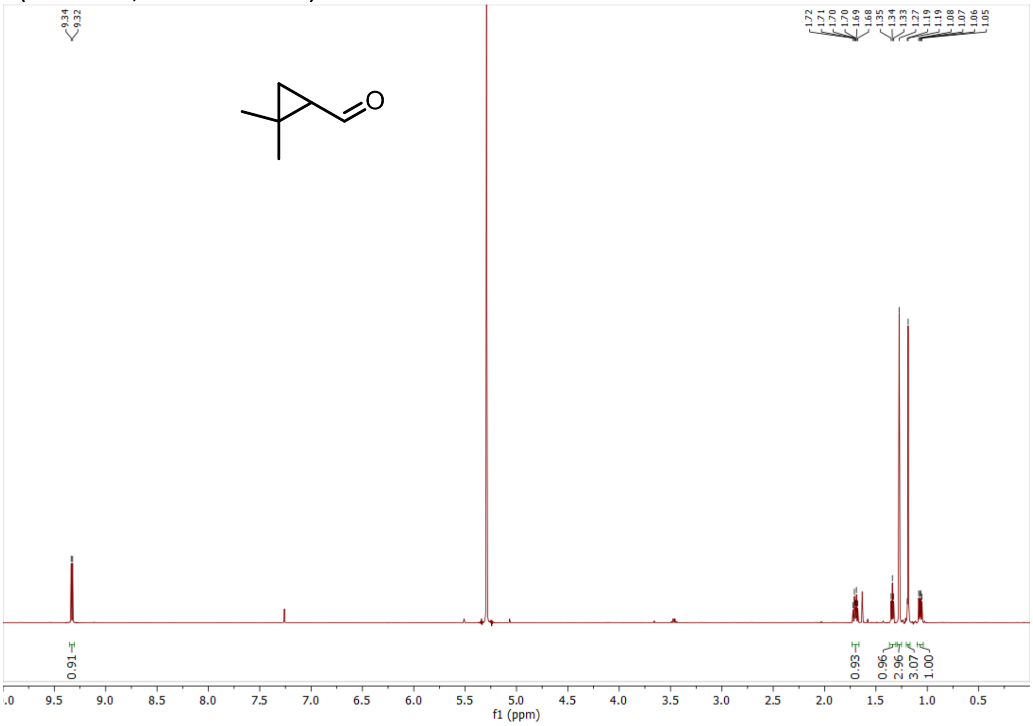

***tert*-Pentyl 2-bromo-2,2-difluoroacetate (3b)**

^{1}**H NMR** (400 MHz, Chloroform-*d*):

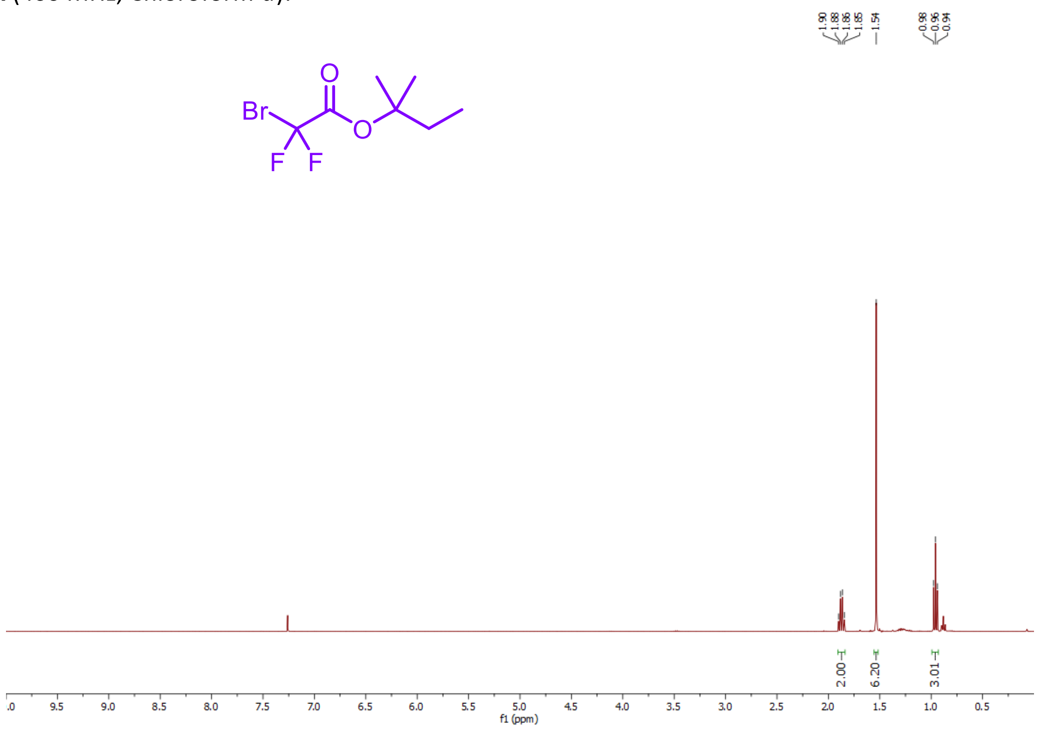

^{19}**F NMR** (376 MHz, Chloroform-*d*):

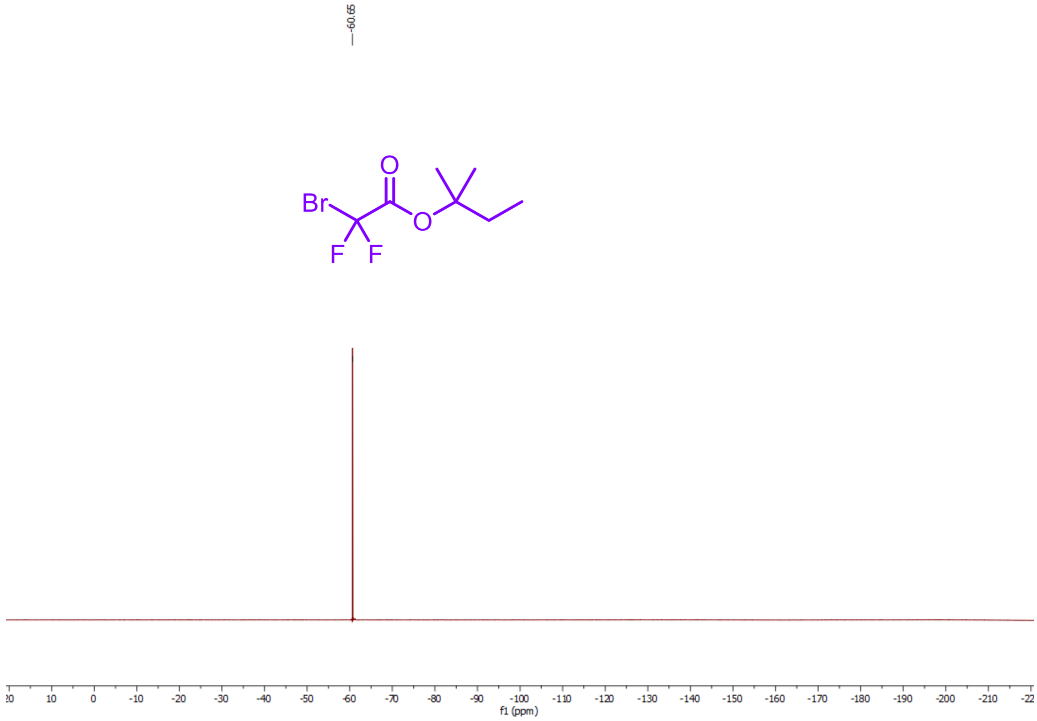

Cyclohexyl 2-bromo-2,2-difluoroacetate (3c)

^{1}H NMR (400 MHz, Chloroform-*d*):

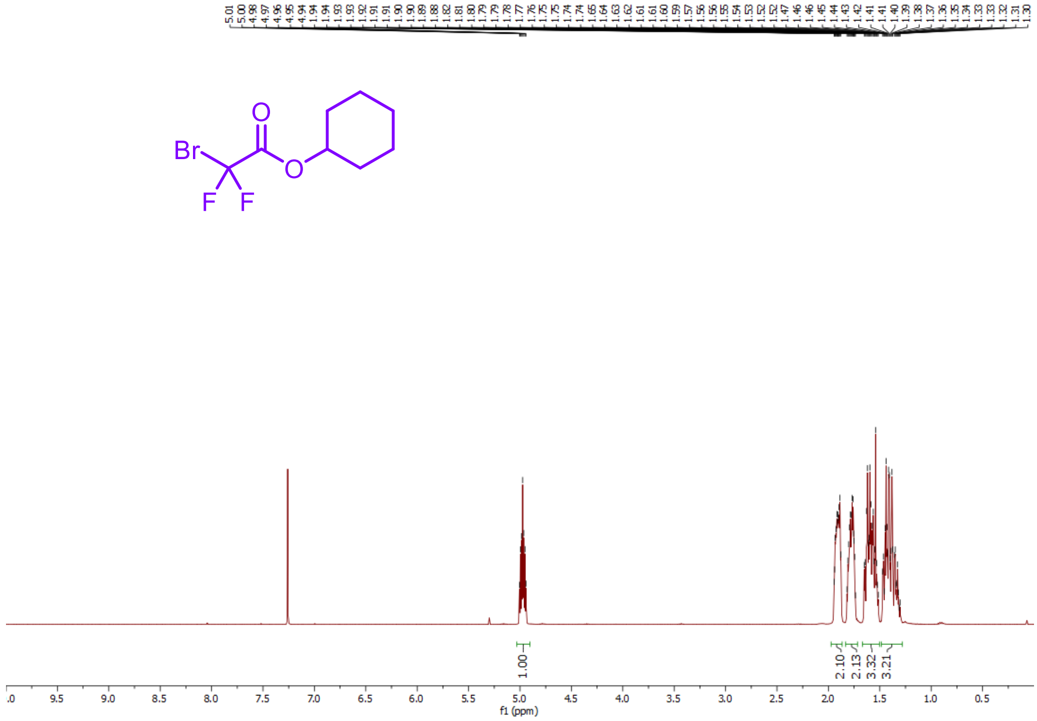

^{19}F NMR (376 MHz, Chloroform-*d*):

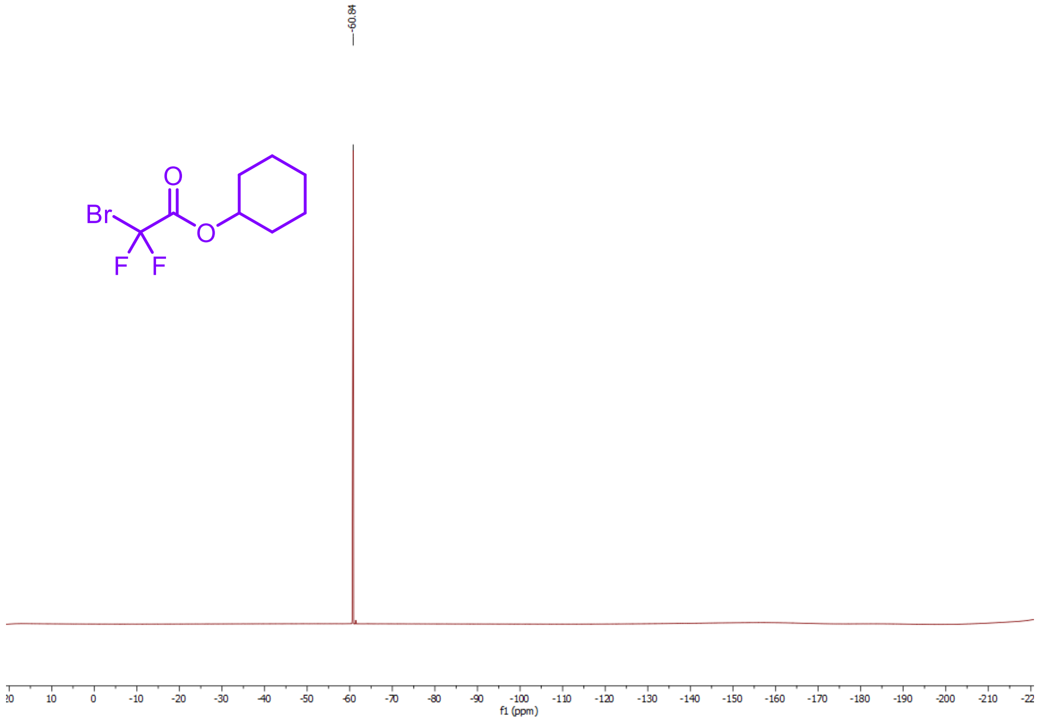

Allyl 2-bromo-2,2-difluoroacetate (3d)

^{1}H NMR (400 MHz, Chloroform-*d*):

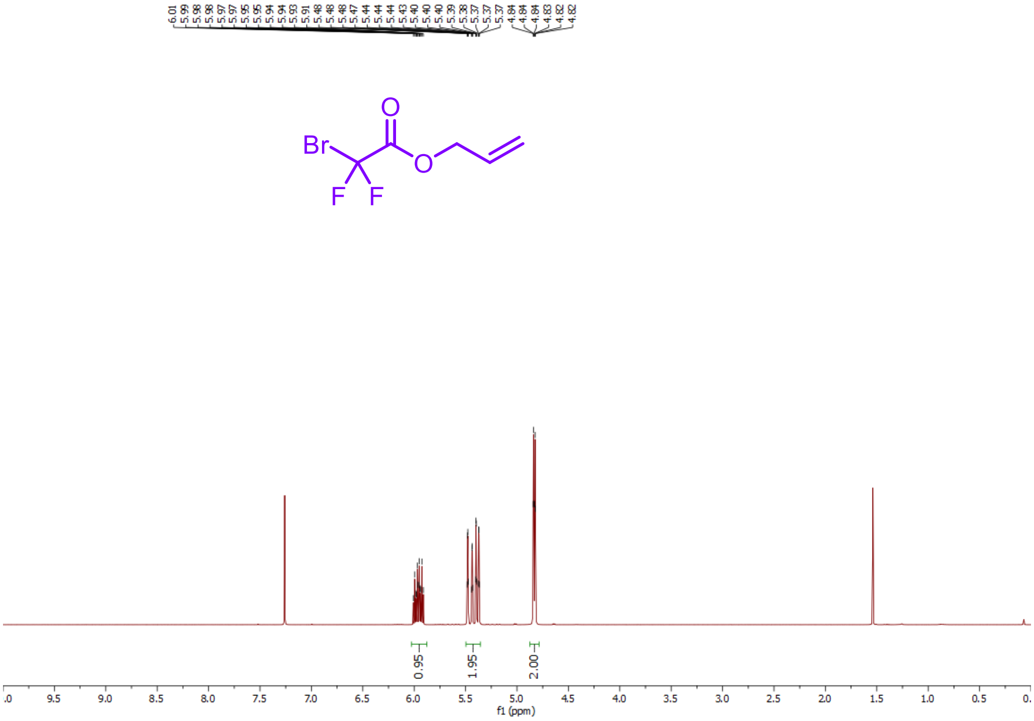

^{19}F NMR (376 MHz, Chloroform-*d*):

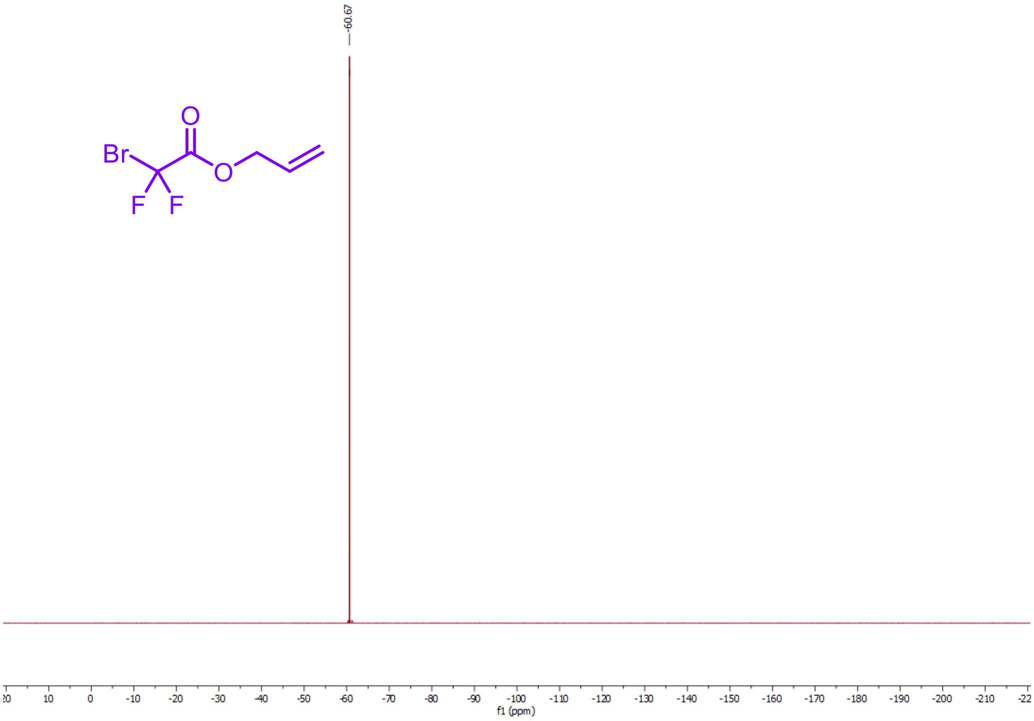

Prop-2-yn-1-yl 2-bromo-2,2-difluoroacetate (3e)

^{1}H NMR (400 MHz, Chloroform-*d*):

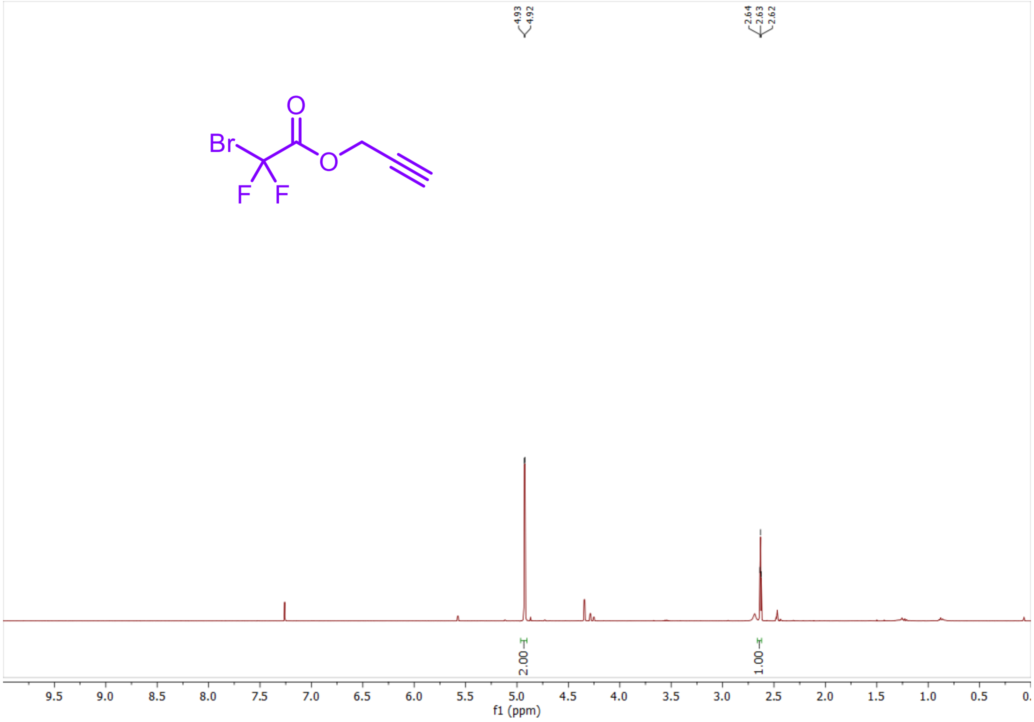

^{19}F NMR (376 MHz, Chloroform-*d*):

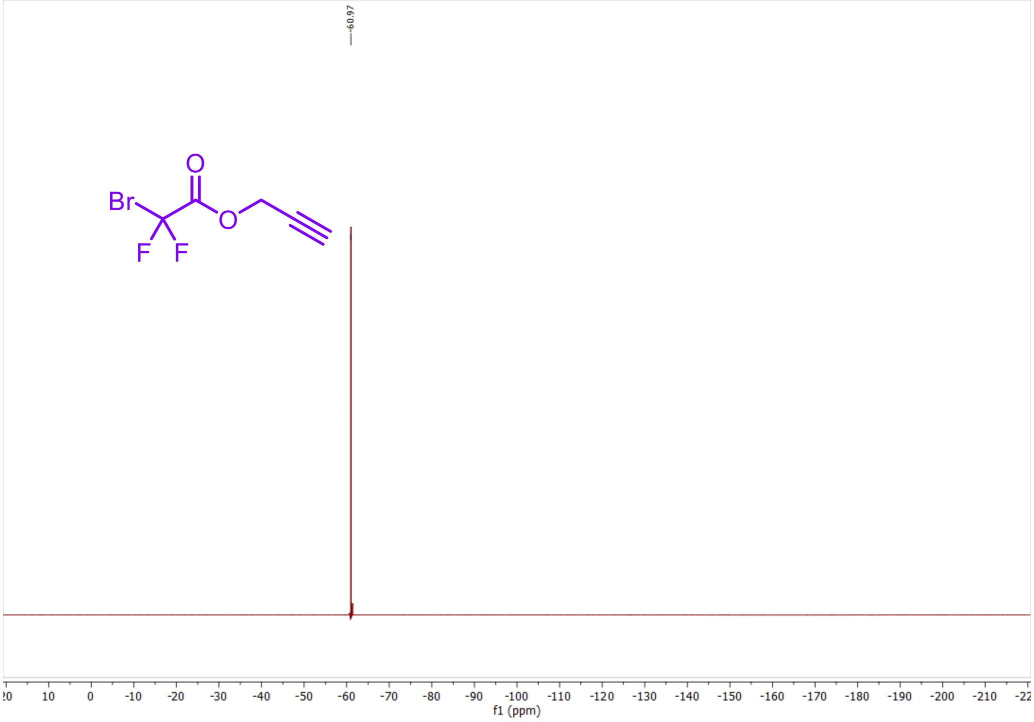

(*E*)-3,7-Dimethylocta-2,6-dien-1-yl 2-bromo-2,2-difluoroacetate (ca)

^{1}H NMR (400 MHz, Chloroform-*d*):

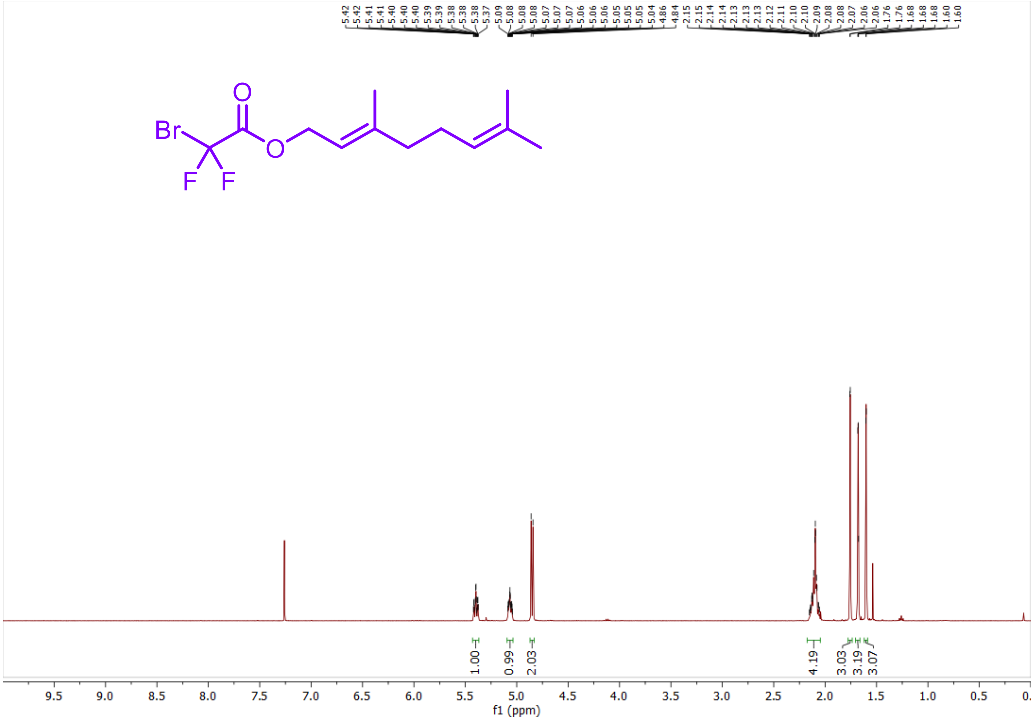

^{13}C NMR (101 MHz, Chloroform-d):

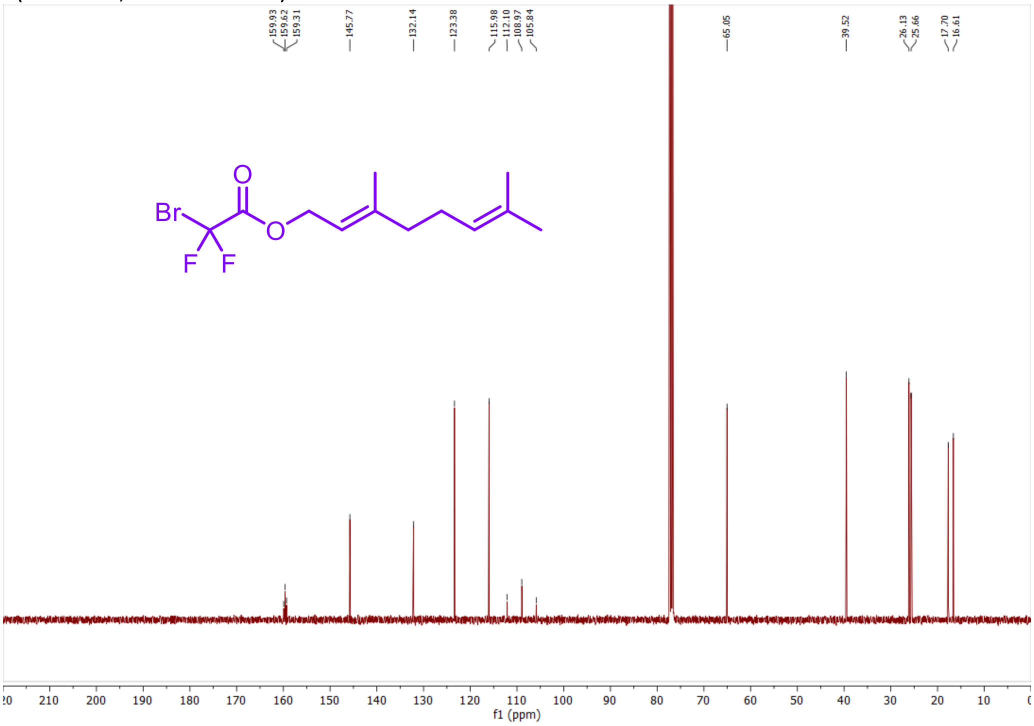

^{19}F NMR (376 MHz, Chloroform-*d*):

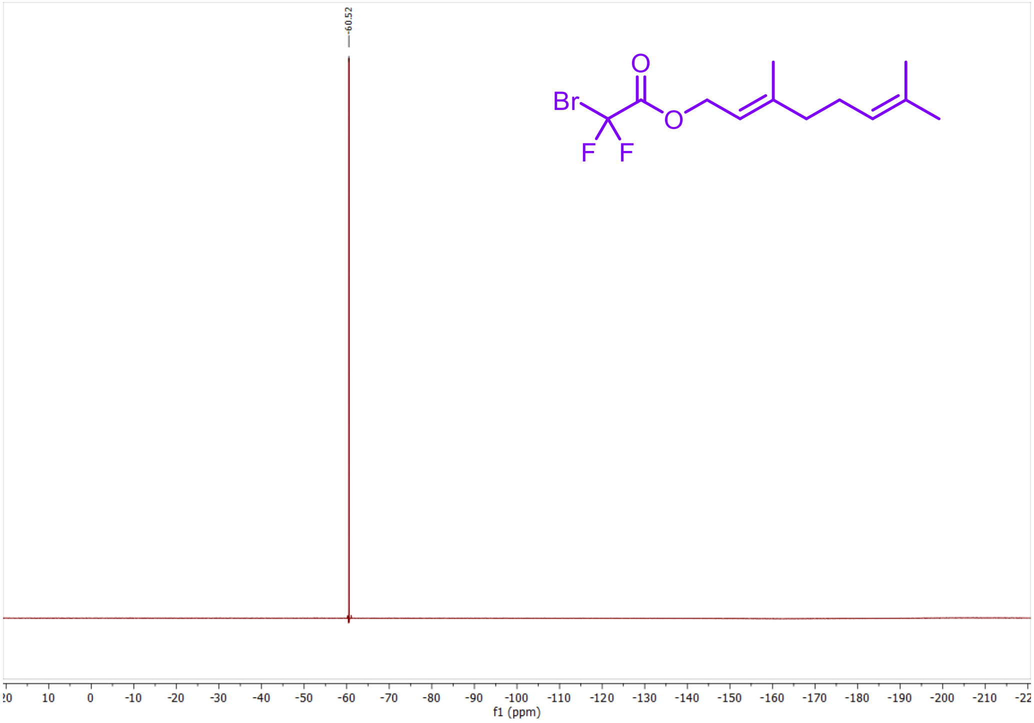

(3*S*,8*S*,9*S*,10R,13*R*,14*S*,17*R*)-10,13-dimethyl-17-((*R*)-6-methylheptan-2-yl)-2,3,4,7,8,9,10,11,12,13,14,15,16,17-tetradecahydro-1*H*-cyclopenta[a]phenanthren-3-yl 2-bromo-2,2-difluoroacetate (cb)

^{1}**H NMR** (400 MHz, Chloroform-d):

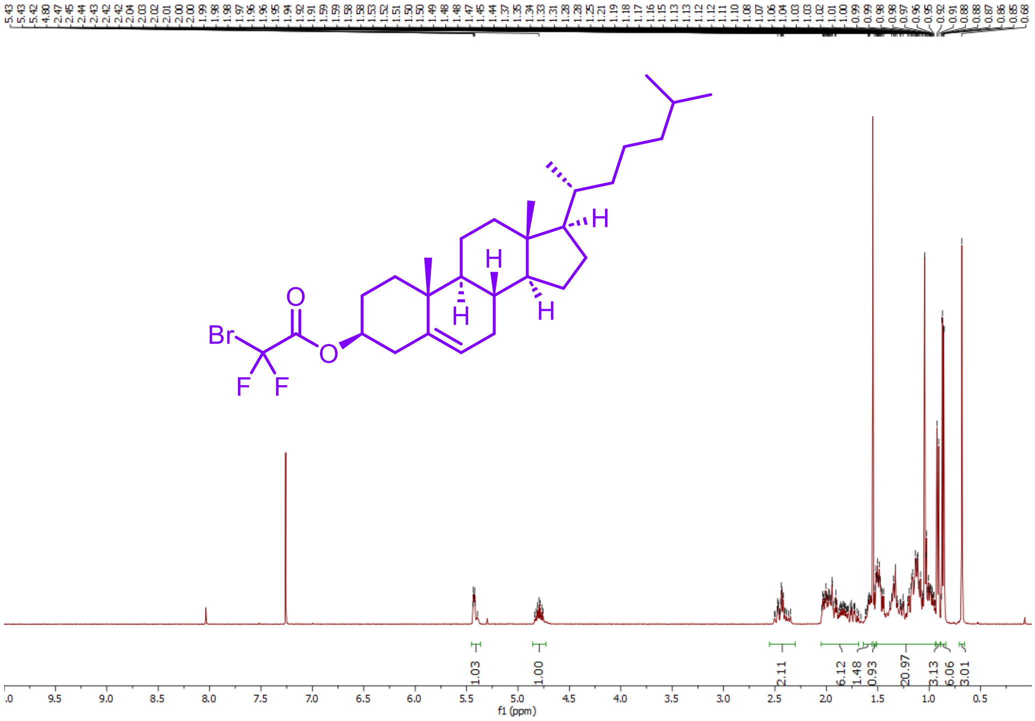

^{19}**F NMR** (376 MHz, Chloroform-*d*):

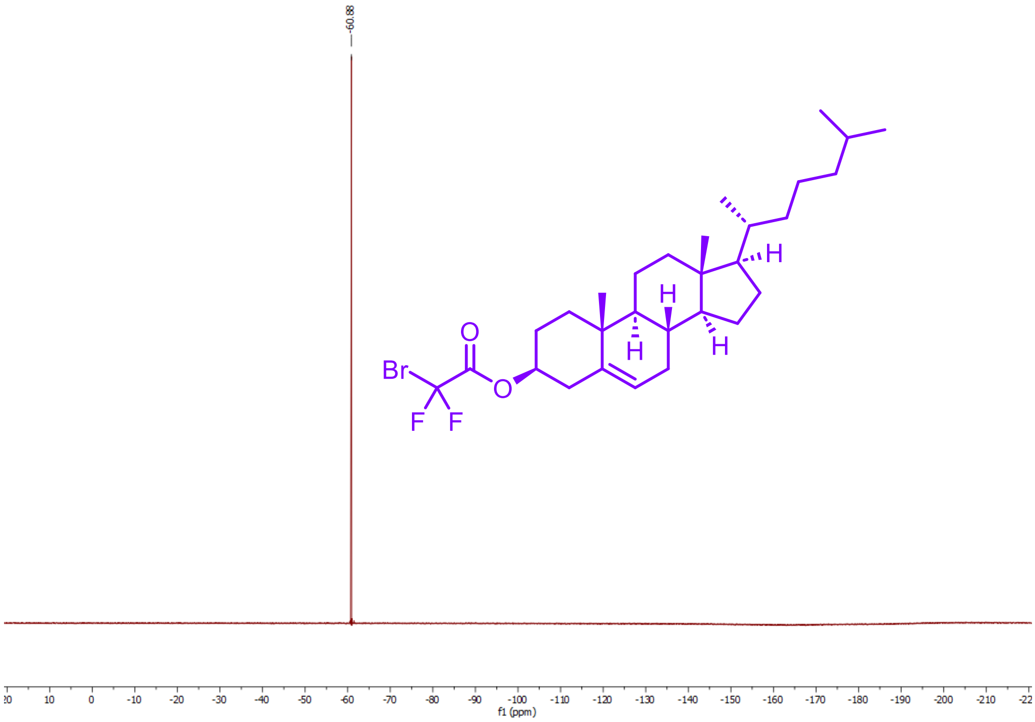

2-Bromo-*N*,*N*-diethyl-2,2-difluoroacetamide (3f)

^{1}H NMR (400 MHz, Chloroform-*d*):

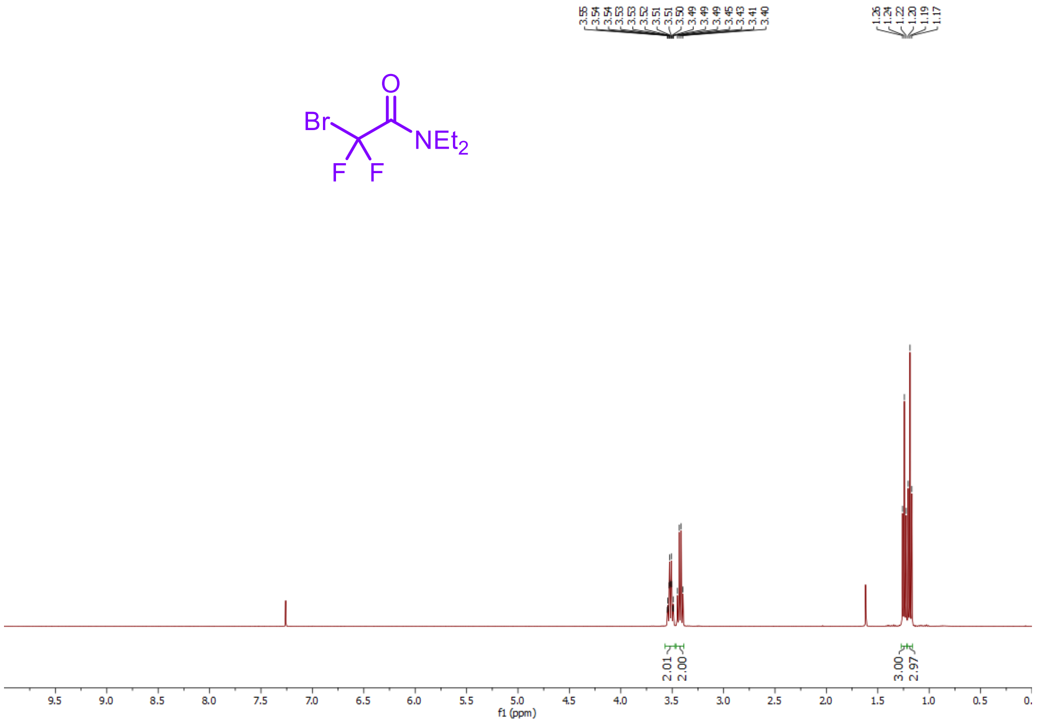

^{19}F NMR (376 MHz, Chloroform-*d*):

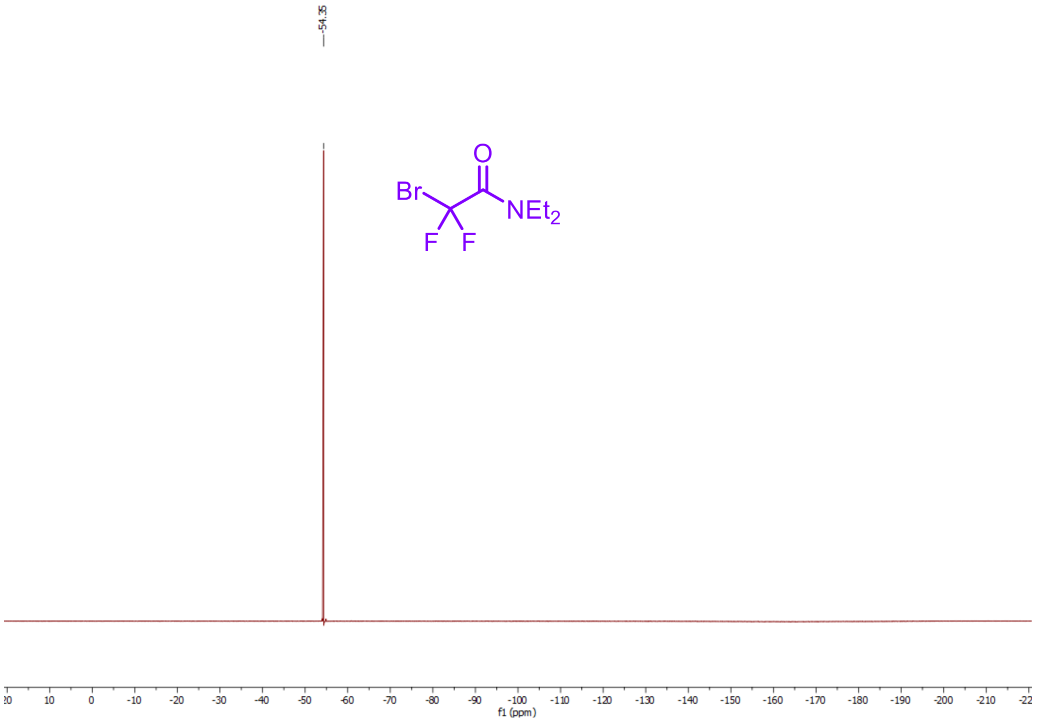

2-Bromo-2,2-difluoro-1-morpholinoethan-1-one (3g)

^{1}H NMR (400 MHz, Chloroform-*d*):

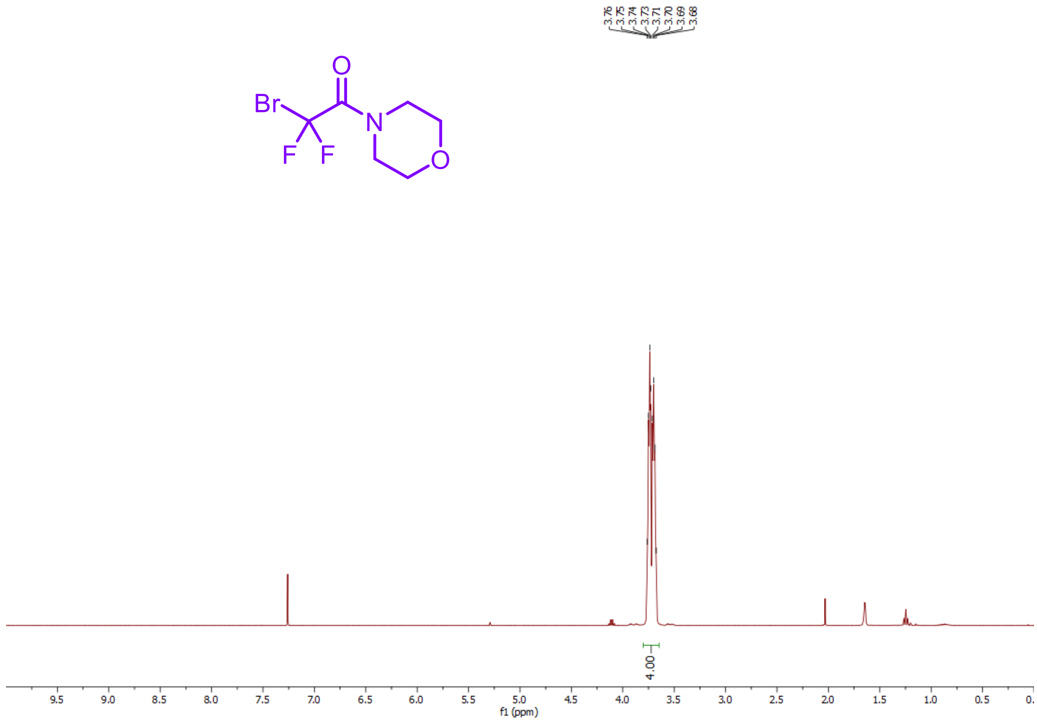

^{19}F NMR (376 MHz, Chloroform-*d*):

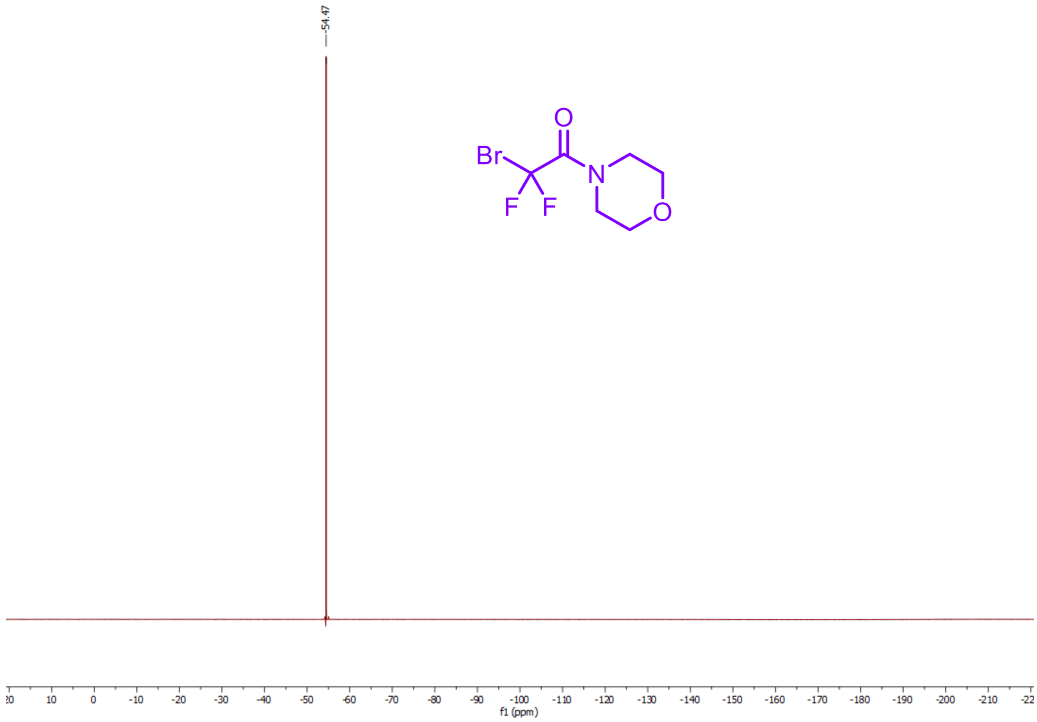

2-Bromo-2,2-difluoro-1-(isoindolin-2-yl)ethan-1-one (3h)

^{1}H NMR (400 MHz, Chloroform-*d*):

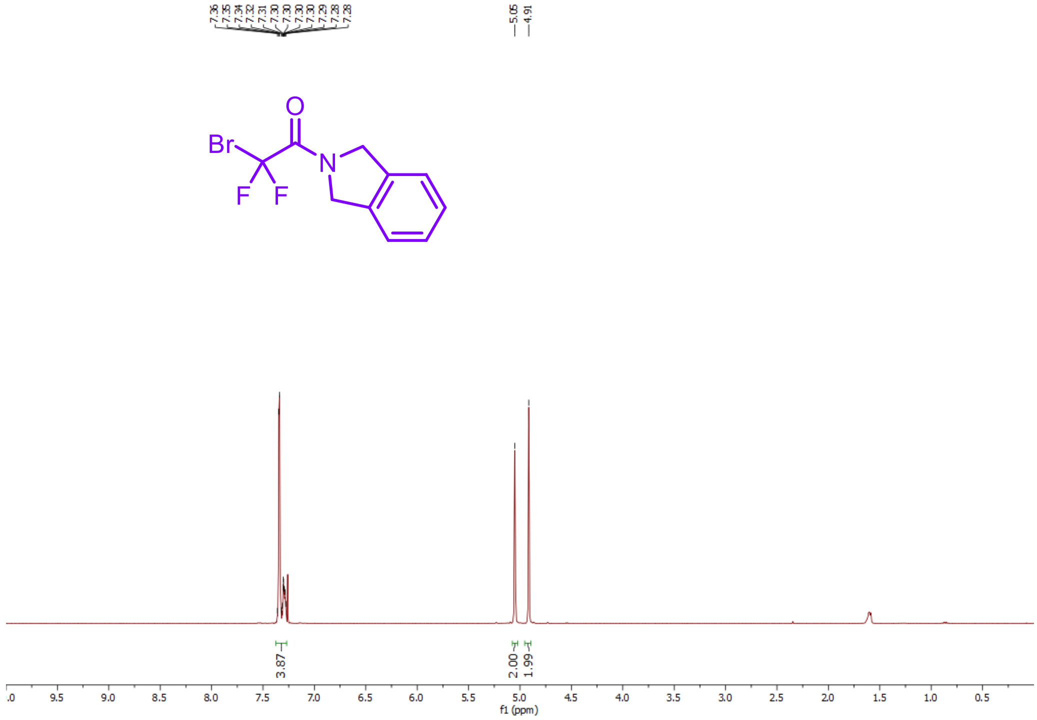

^{19}F NMR (376 MHz, Chloroform-*d*):

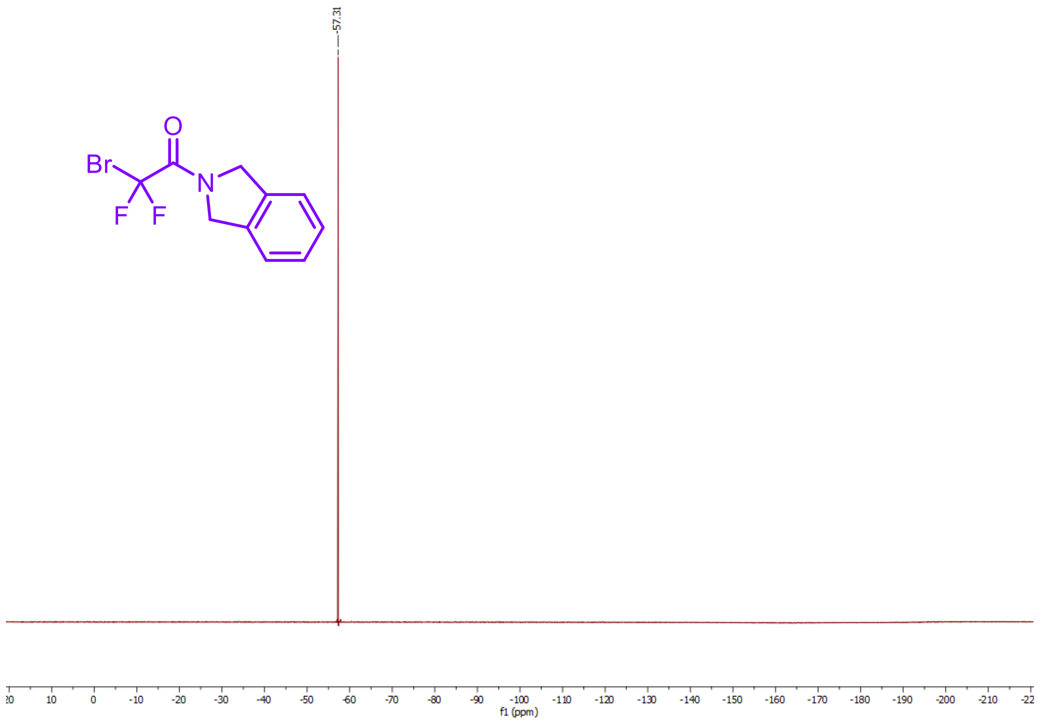

2-Bromo-*N*-(*tert*-butyl)-2,2-difluoroacetamide (3i)

^{1}H NMR (400 MHz, Chloroform-*d*):

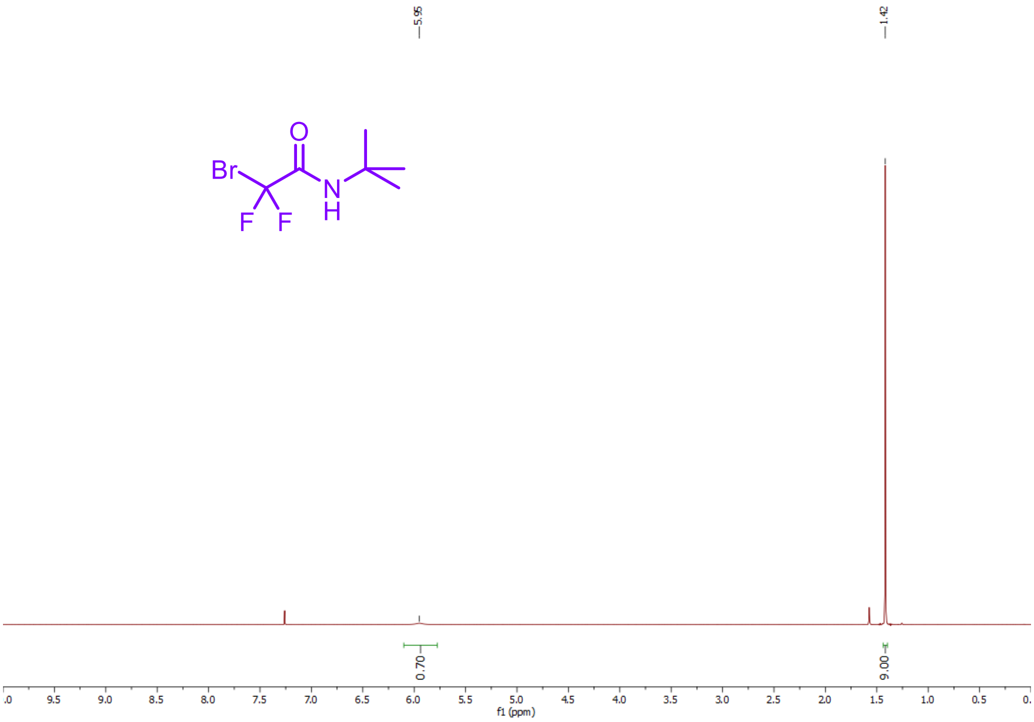

^{19}F NMR (376 MHz, Chloroform-*d*):

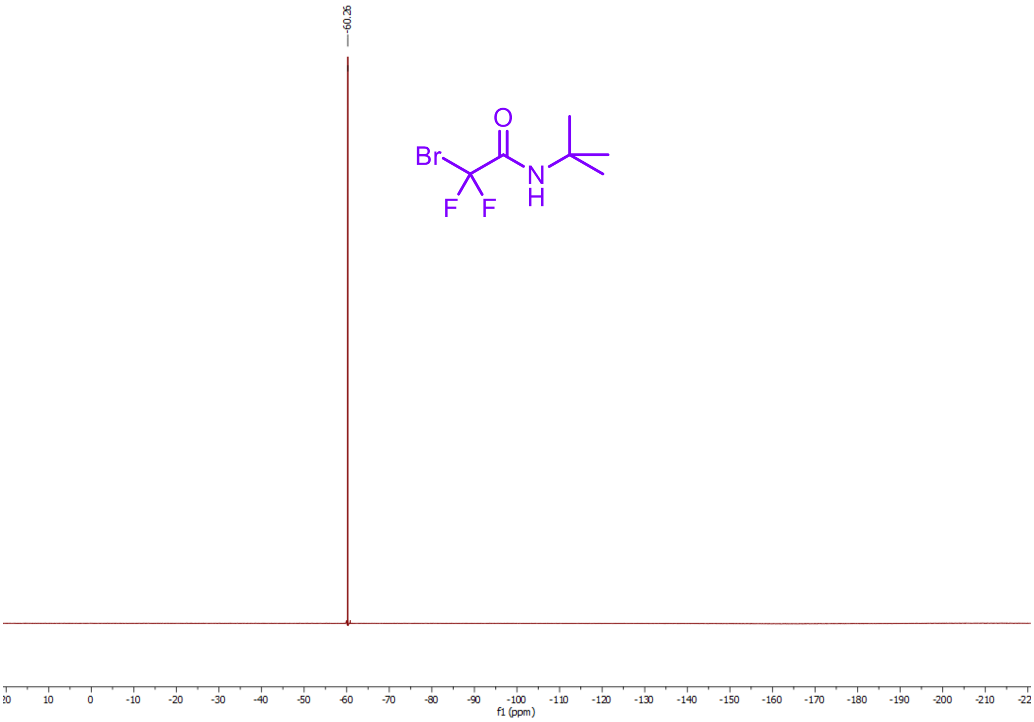

((Bromodifluoromethyl)sulfonyl)benzene (3j)

^{1}H NMR (400 MHz, Chloroform-*d*):

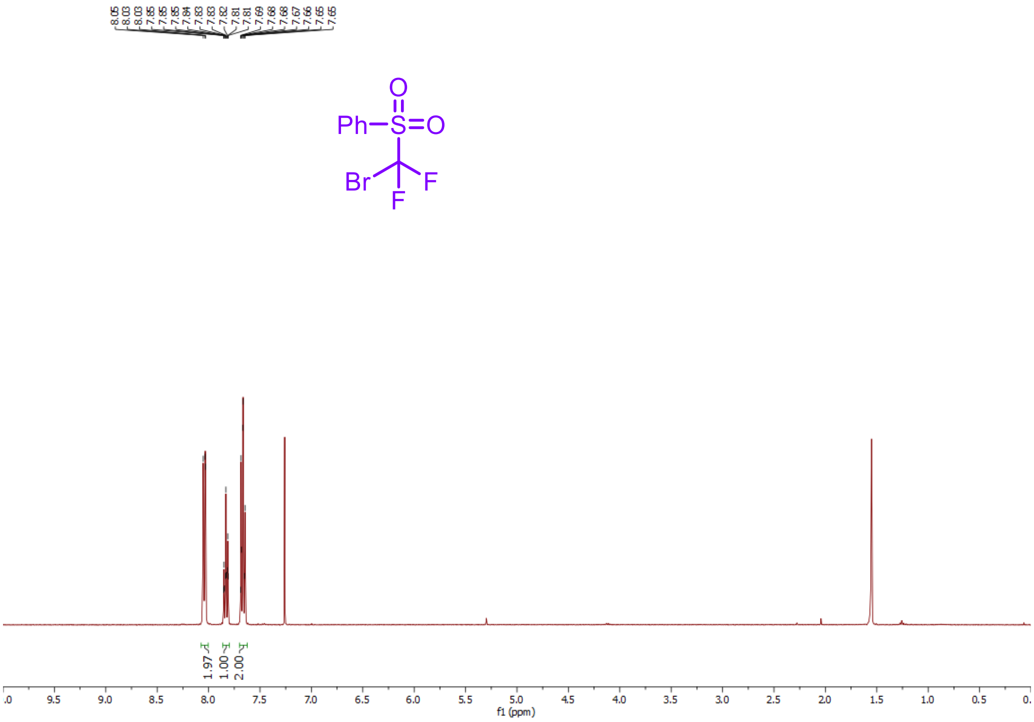

^{19}F NMR (376 MHz, Chloroform-*d*):

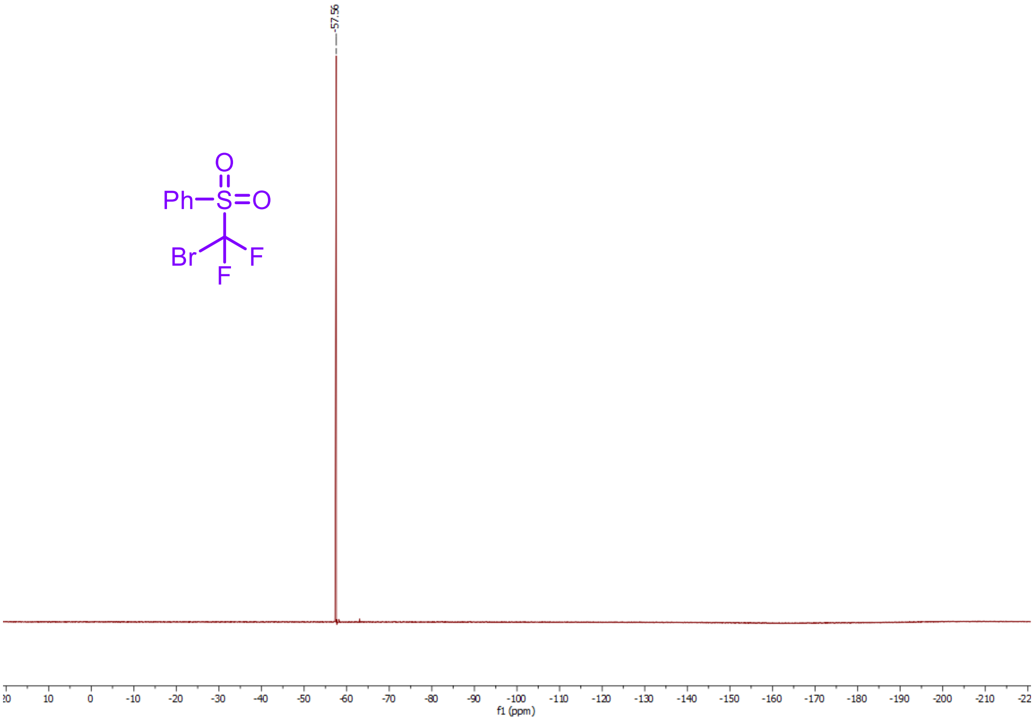

1,3-Dioxoisoindolin-2-yl pivalate (24)

^{1}H NMR (400 MHz, Chloroform-*d*):

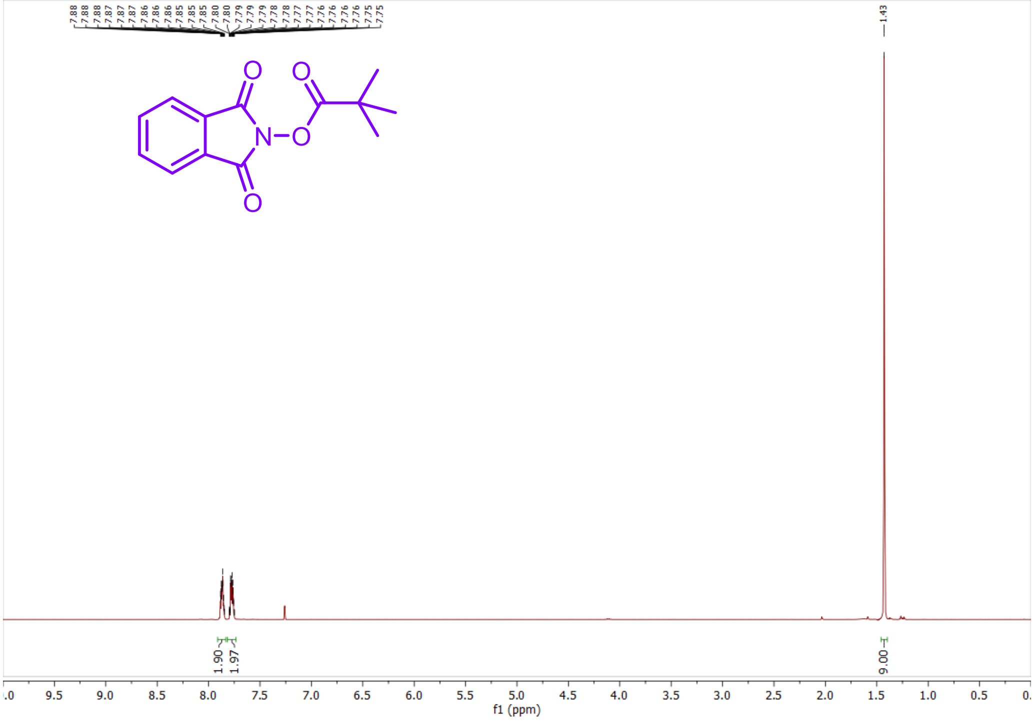

1,3-dioxoisoindolin-2-yl 2-phenylpropanoate (25)

^{1}H NMR (400 MHz, Chloroform-*d*):

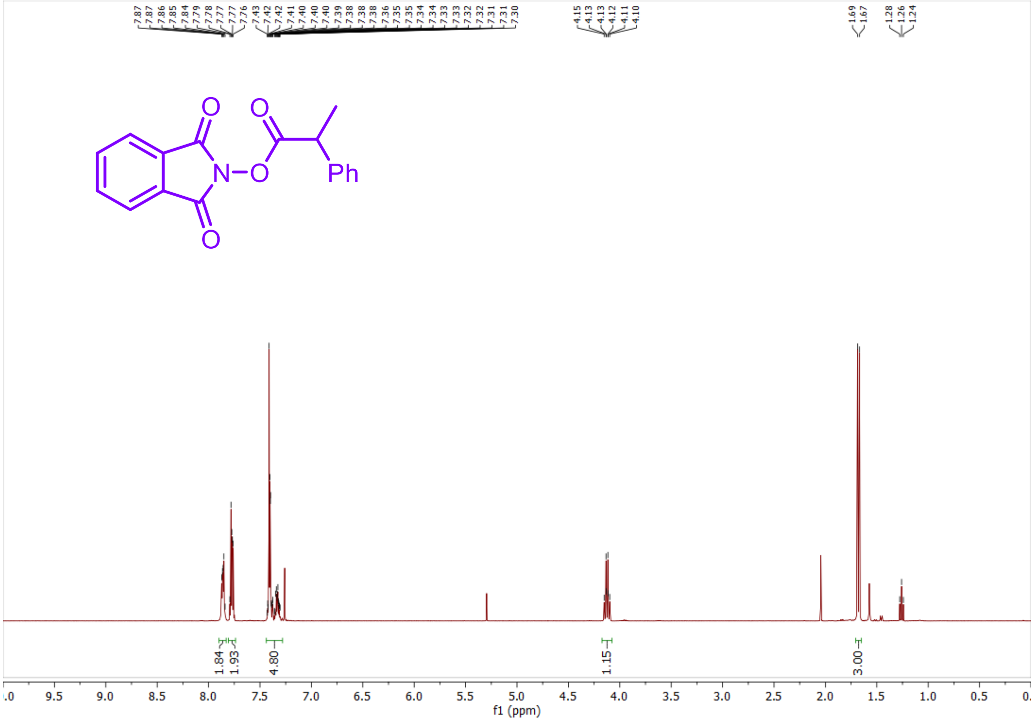

1-(1-Methoxy-1-oxo-3-phenylpropan-2-yl)-2,4,6-triphenylpyridin-1-ium tetrafluoroborate (26)

^{1}H NMR (400 MHz, Chloroform-*d*):

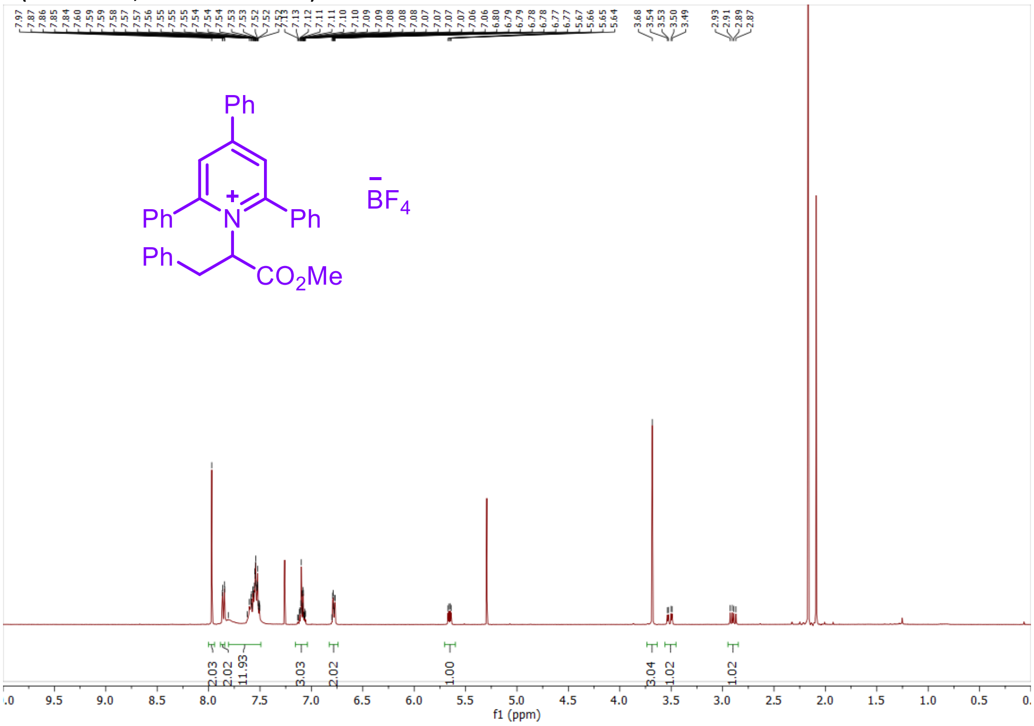

Isopropyl 2-methyl-2-(4-(4-vinylbenzoyl)phenoxy)propanoate (5a)

^{1}H NMR (400 MHz, Chloroform-*d*):

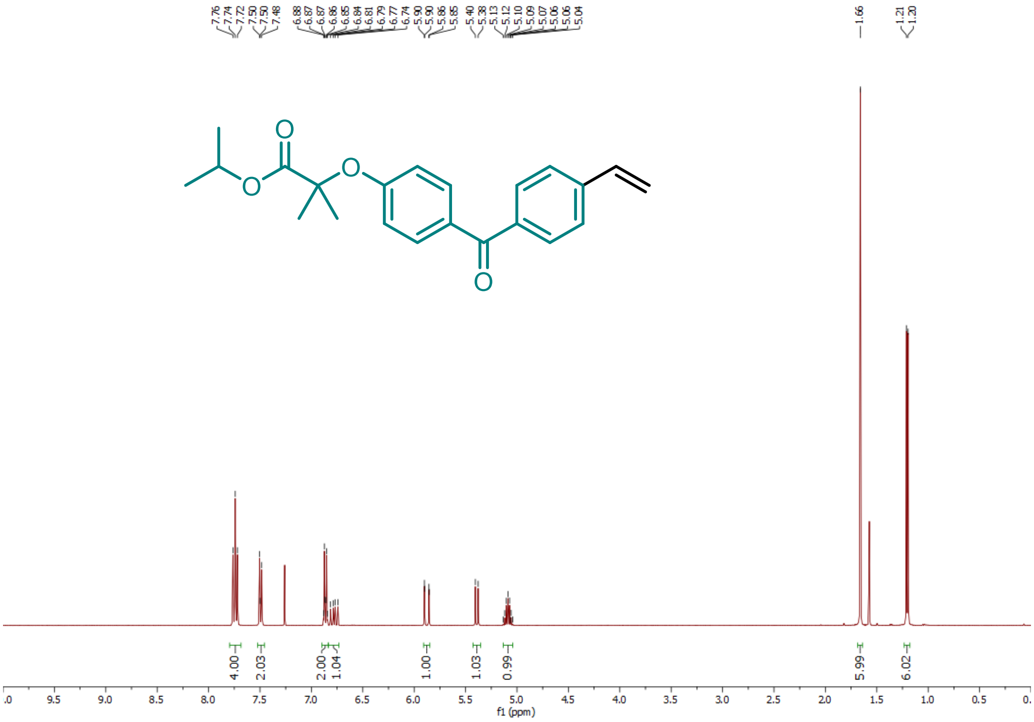

(8*R*,9*S*,13*S*,14*S*)-13-methyl-3-vinyl-6,7,8,9,11,12,13,14,15,16-decahydro-17*H*-cyclopenta[*a*]phenanthren-17-one (5b)

^{1}H NMR (400 MHz, Chloroform-*d*):

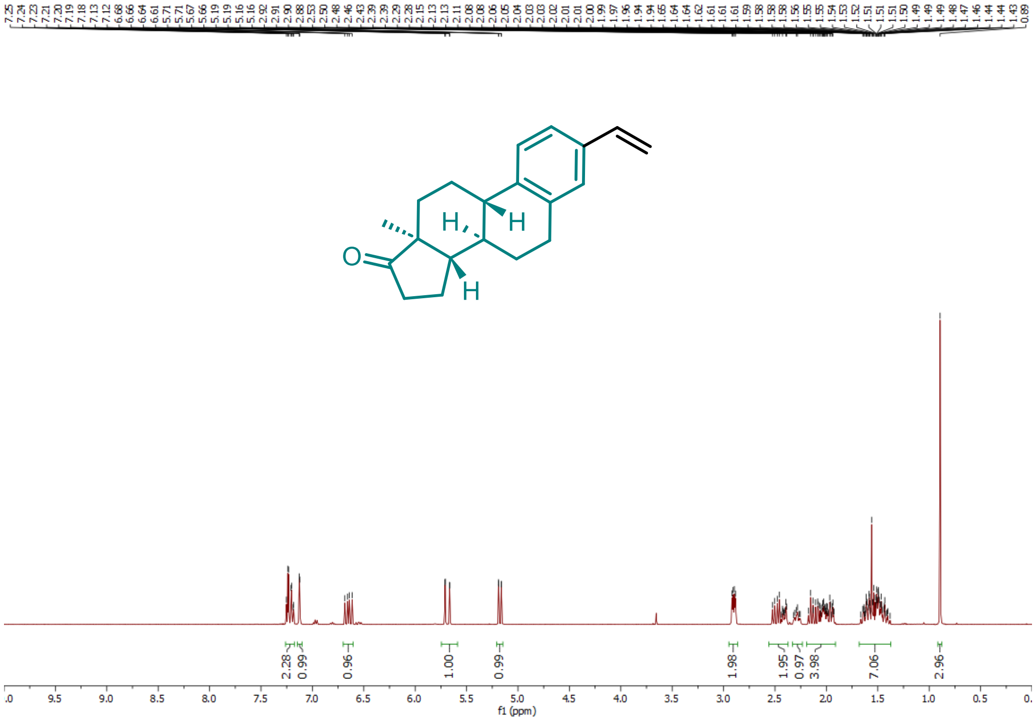

(*R*)-2,8-dimethyl-2-((4*R*,8*R*)-4,8,12-trimethyltridecyl)-6-vinylchromane (5c)

^{1}H NMR (400 MHz, Chloroform-*d*):

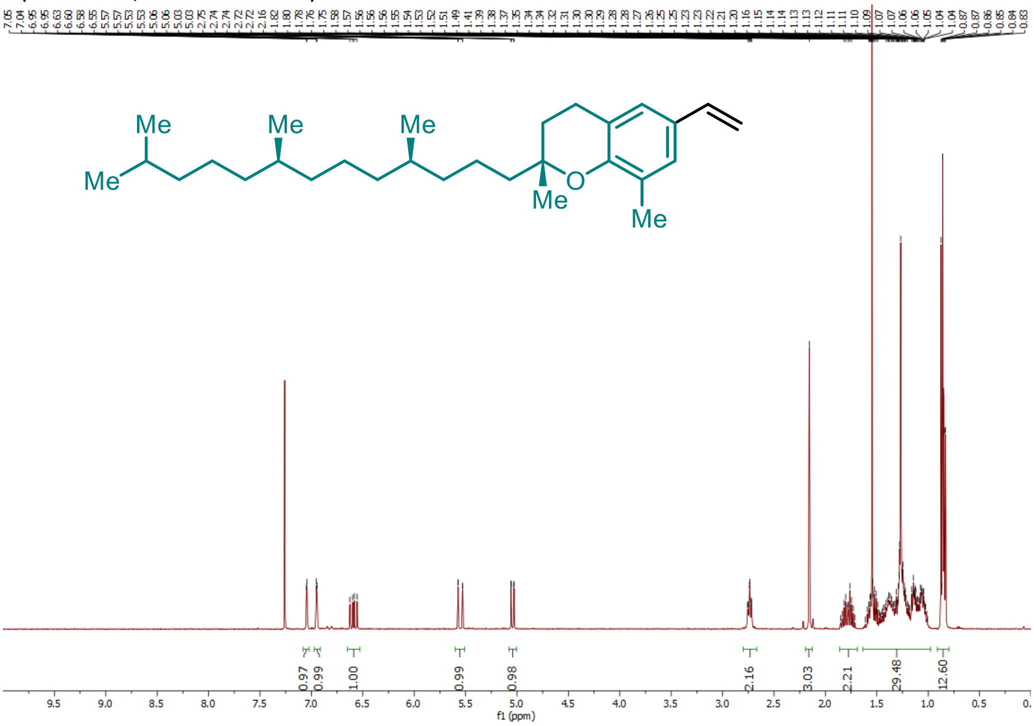

Ethyl (*S*)-2,2-difluoro-5-oxo-4,5-diphenylpentanoate (4a)

^{1}H NMR (400 MHz, Chloroform-*d*):

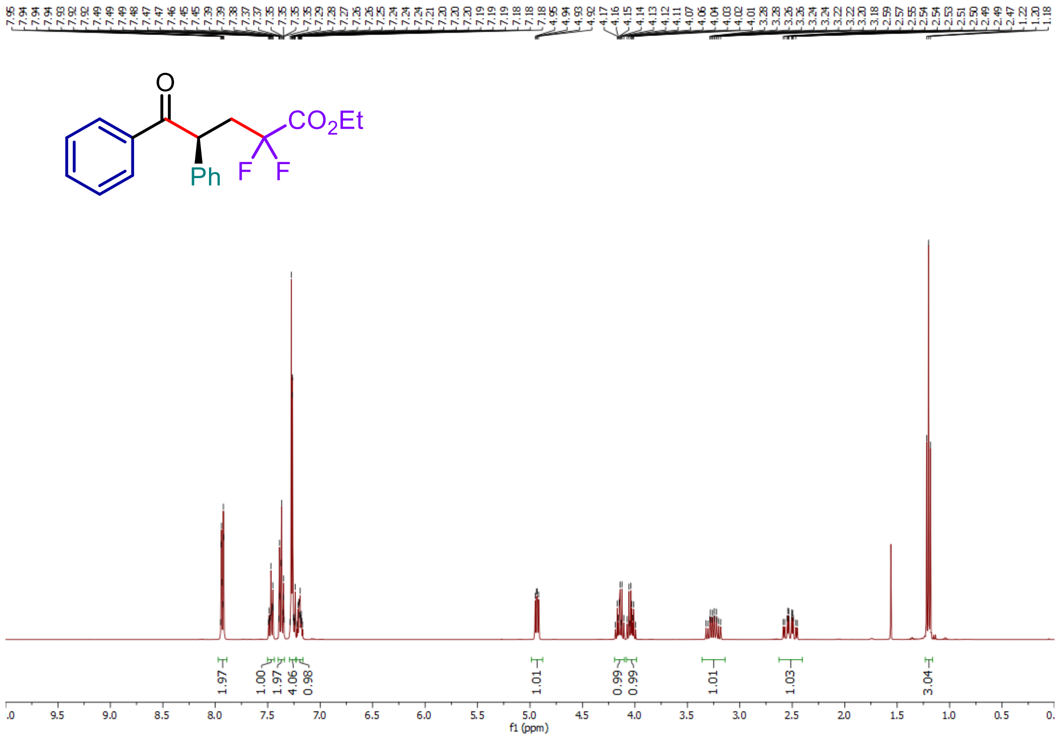

^{13}C NMR (101 MHz, Chloroform-*d*):

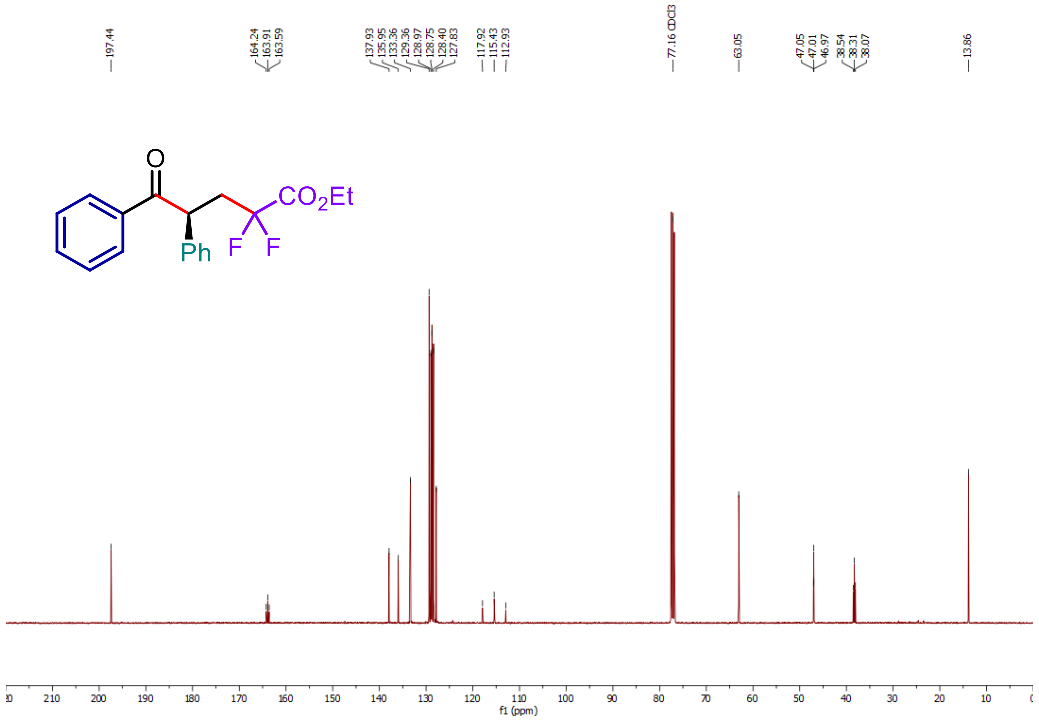

^{19}F NMR (376 MHz, $CDCl_3$):

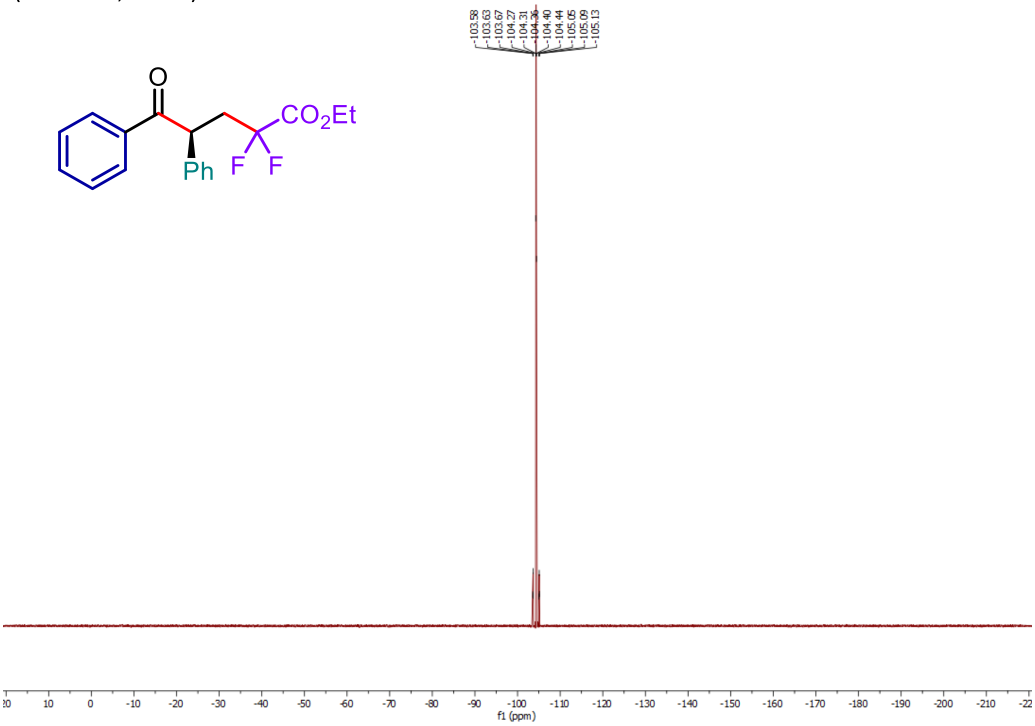

Ethyl (*S*)-2,2-difluoro-5-(4-fluorophenyl)-5-oxo-4-phenylpentanoate (4b)

^{1}H NMR (400 MHz, Chloroform-*d*):

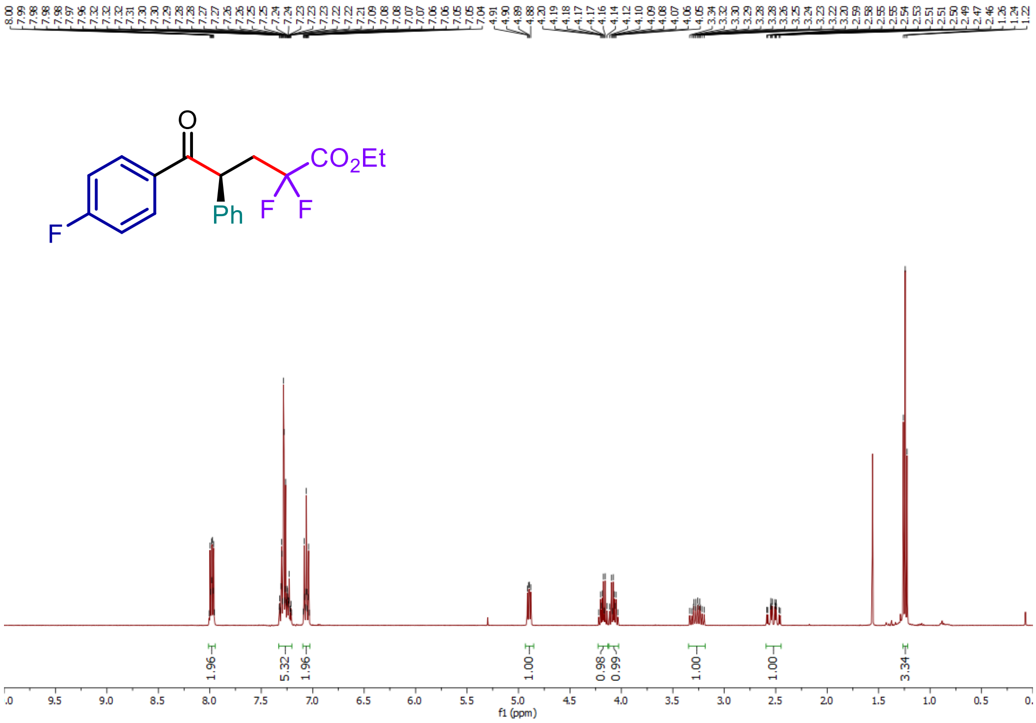

^{13}C NMR (101 MHz, Chloroform-*d*):

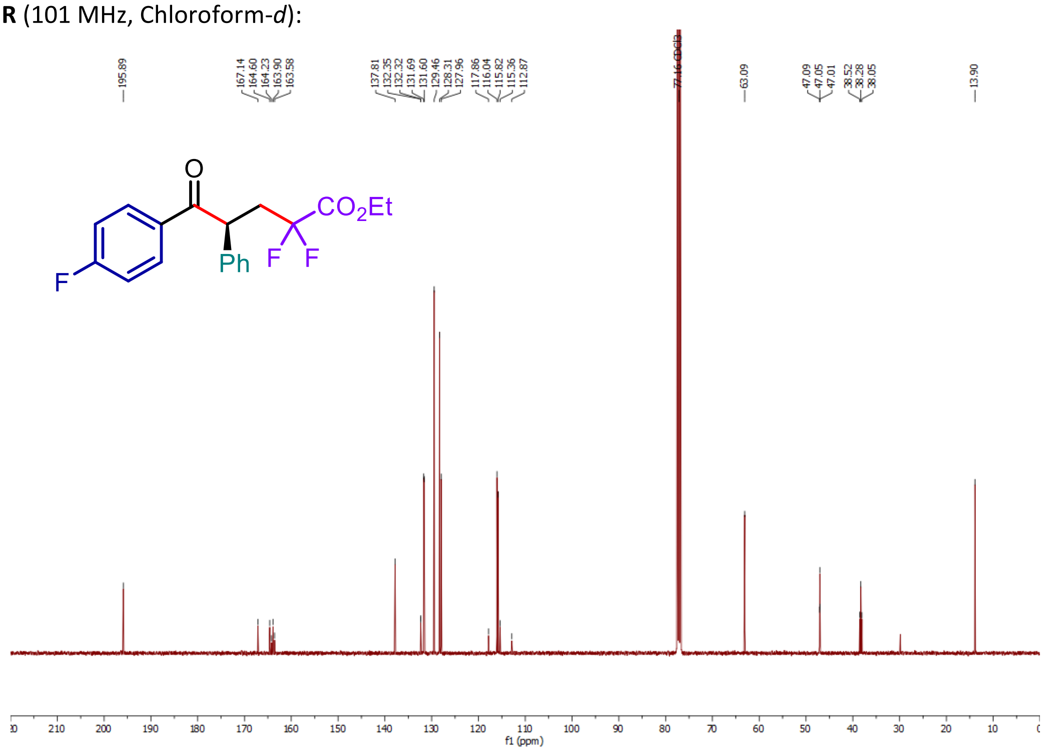

^{19}F NMR (376 MHz, Chloroform-*d*):

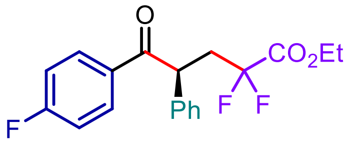

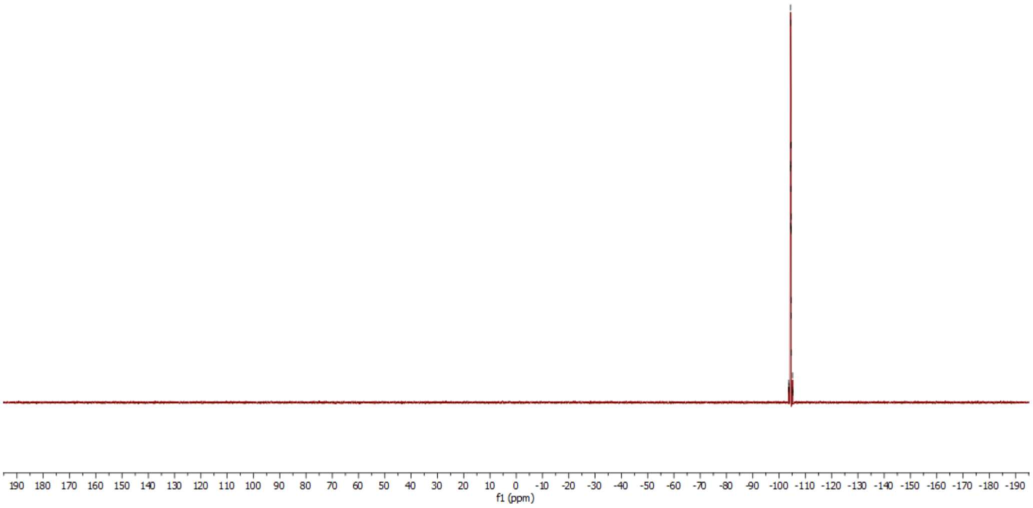

Ethyl (*S*)-5-(4-chlorophenyl)-2,2-difluoro-5-oxo-4-phenylpentanoate (4c)

^{1}H NMR (400 MHz, $CDCl_3$):

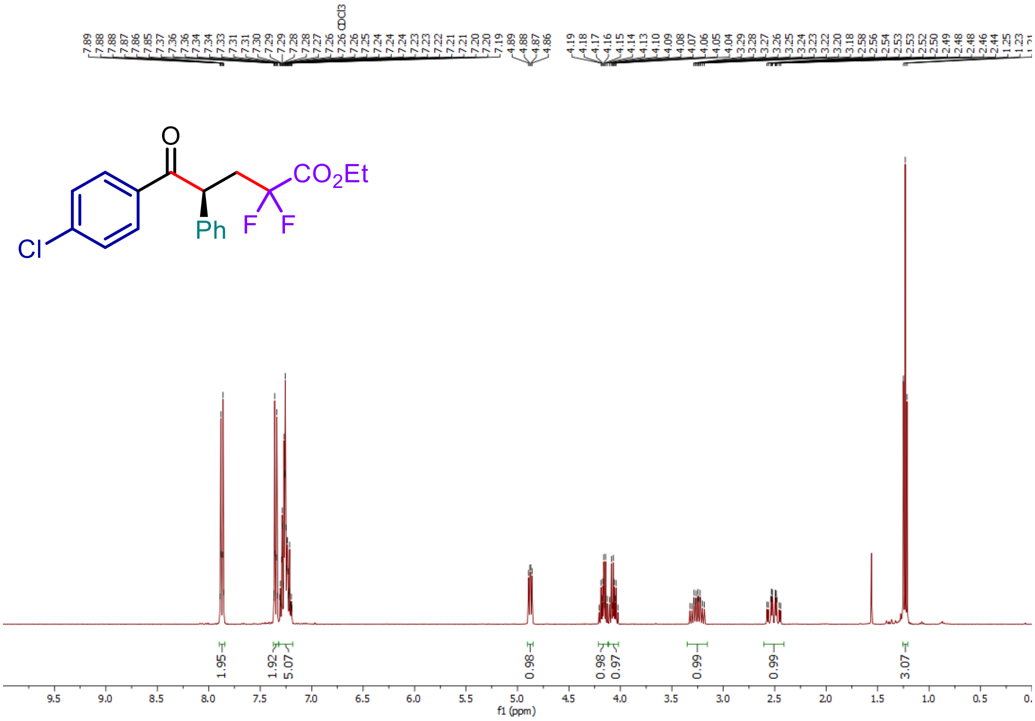

^{13}C NMR (101 MHz, $CDCl_3$):

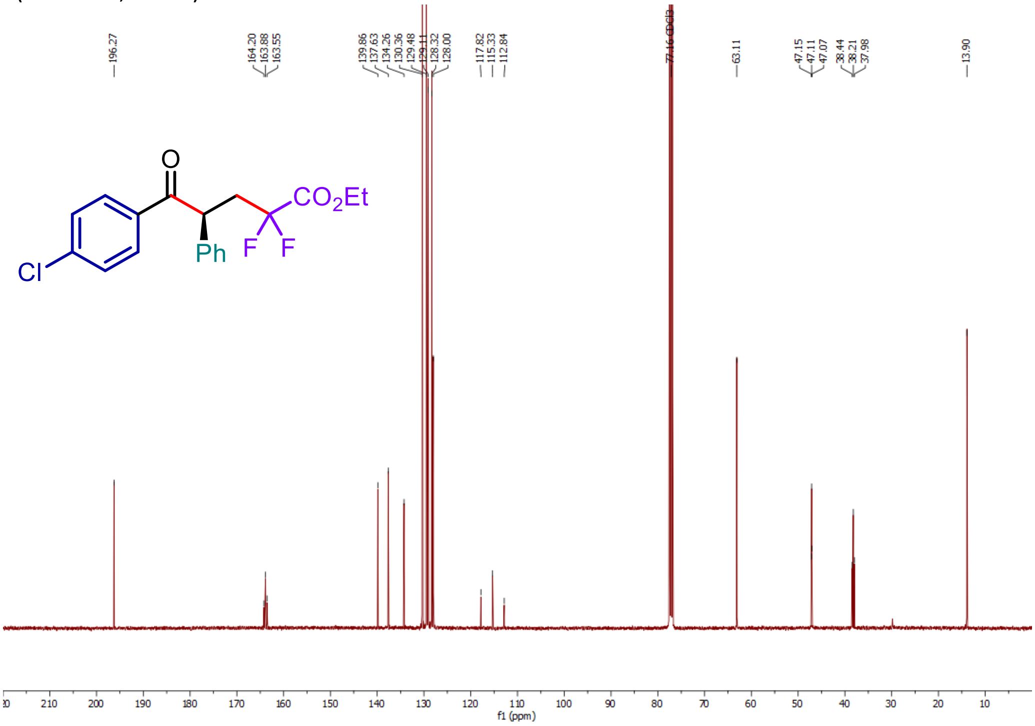

^{19}F NMR (376 MHz, $CDCl_3$):

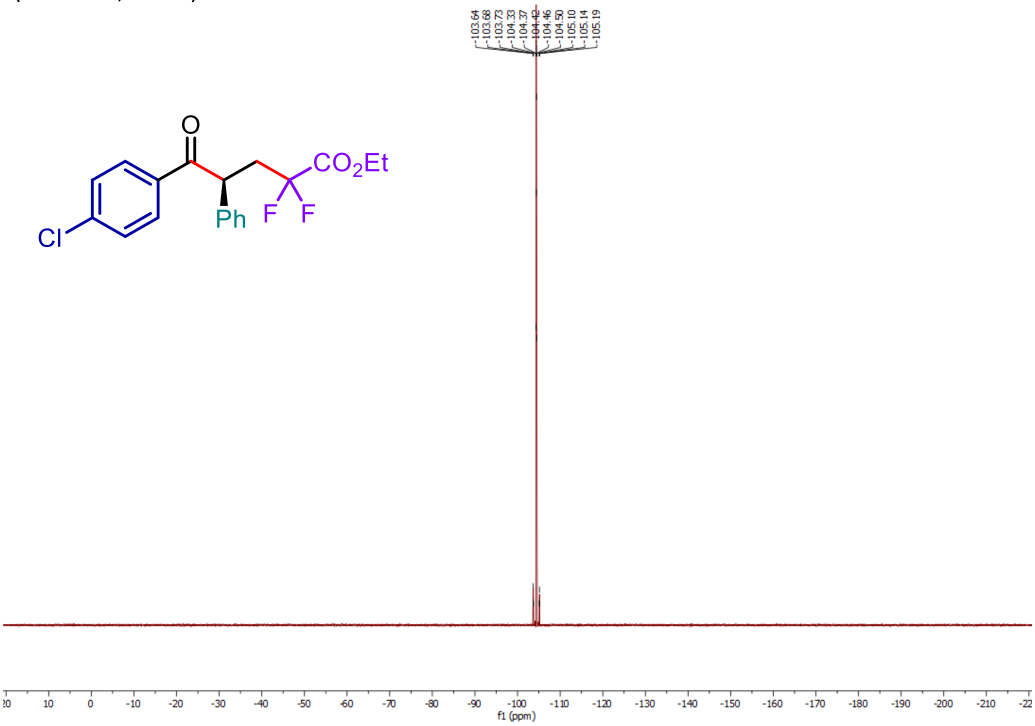

Ethyl (*S*)-2,2-difluoro-5-(4-iodophenyl)-5-oxo-4-phenylpentanoate (4d)

^{1}**H NMR** (400 MHz, Chloroform-*d*):

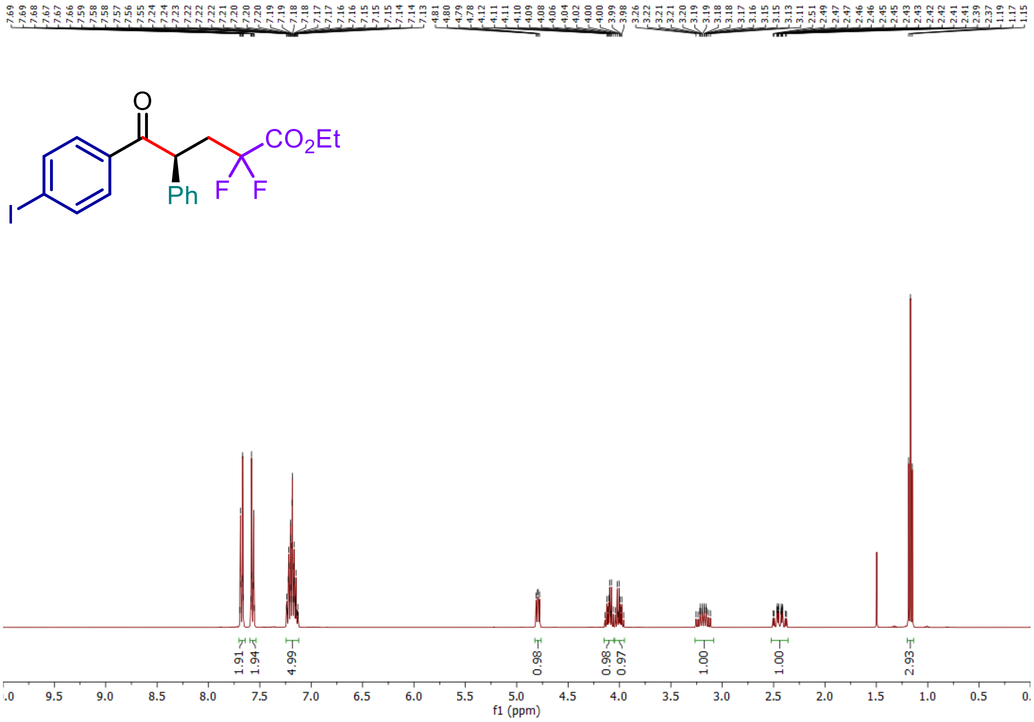

^{13}**C NMR** (101 MHz, Chloroform-*d*):

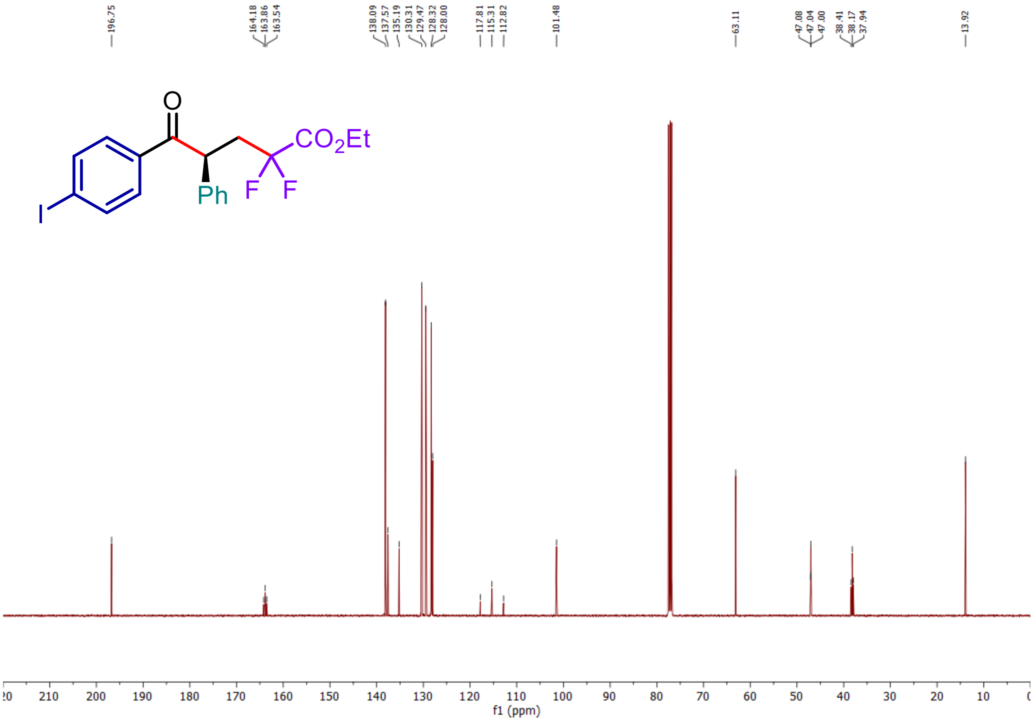

^{19}F NMR (376 MHz, Chloroform-*d*):

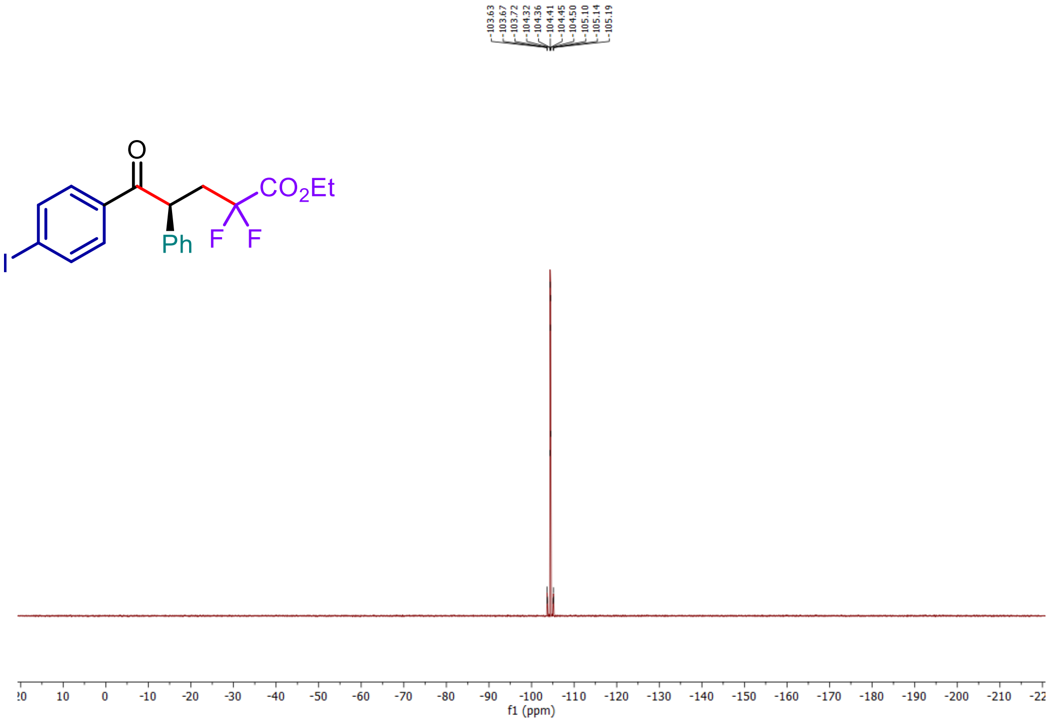

Ethyl (*S*)-2,2-difluoro-5-(4-methoxyphenyl)-5-oxo-4-phenylpentanoate (4e)

^{1}**H NMR** (400 MHz, Chloroform-*d*):

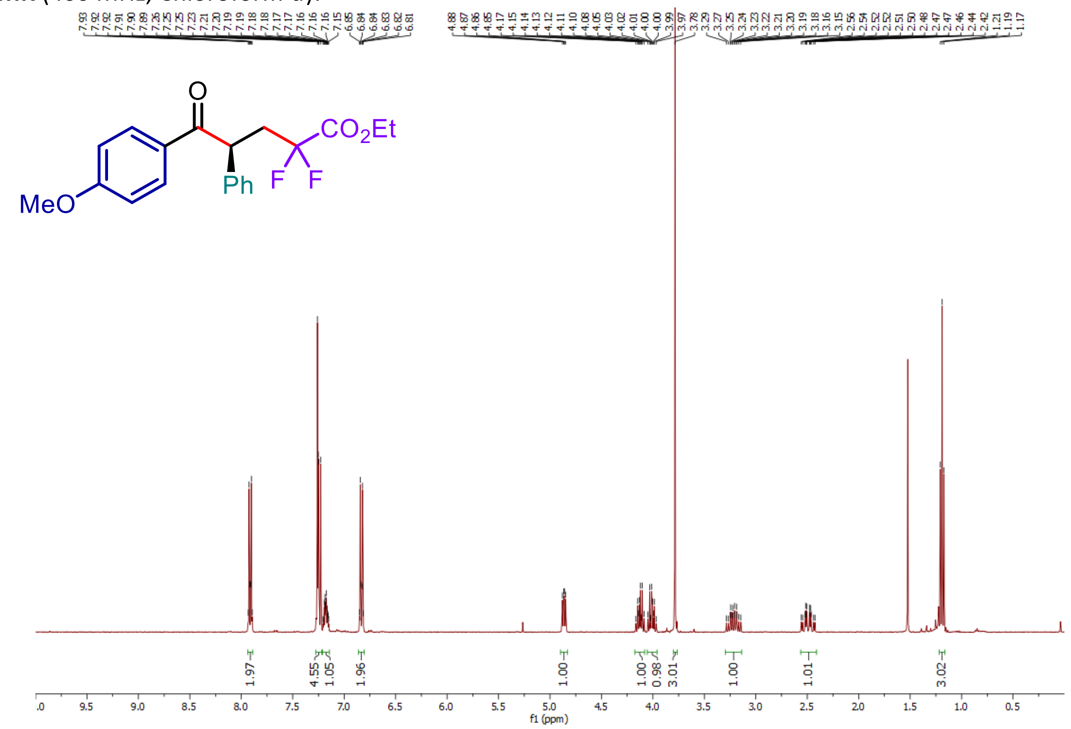

^{13}**C NMR** (101 MHz, Chloroform-*d*):

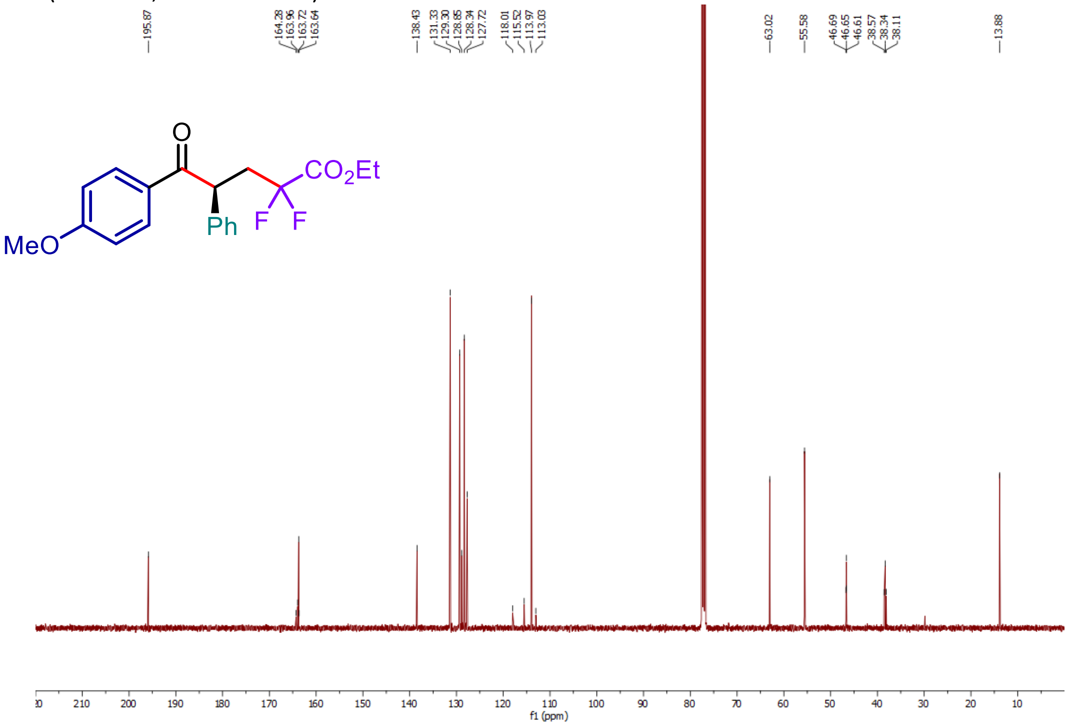

^{19}F NMR (376 MHz, Chloroform-*d*):

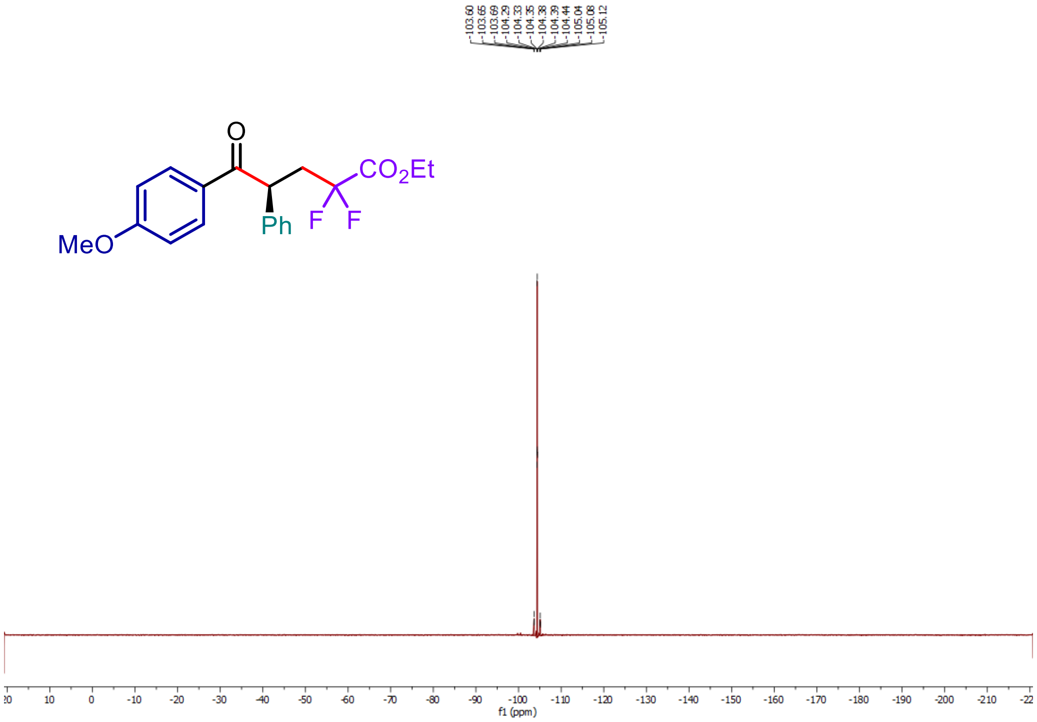

Methyl (*S*)-4-(5-ethoxy-4,4-difluoro-5-oxo-2-phenylpentanoyl)benzoate (4f)

^{1}H NMR (400 MHz, $CDCl_3$):

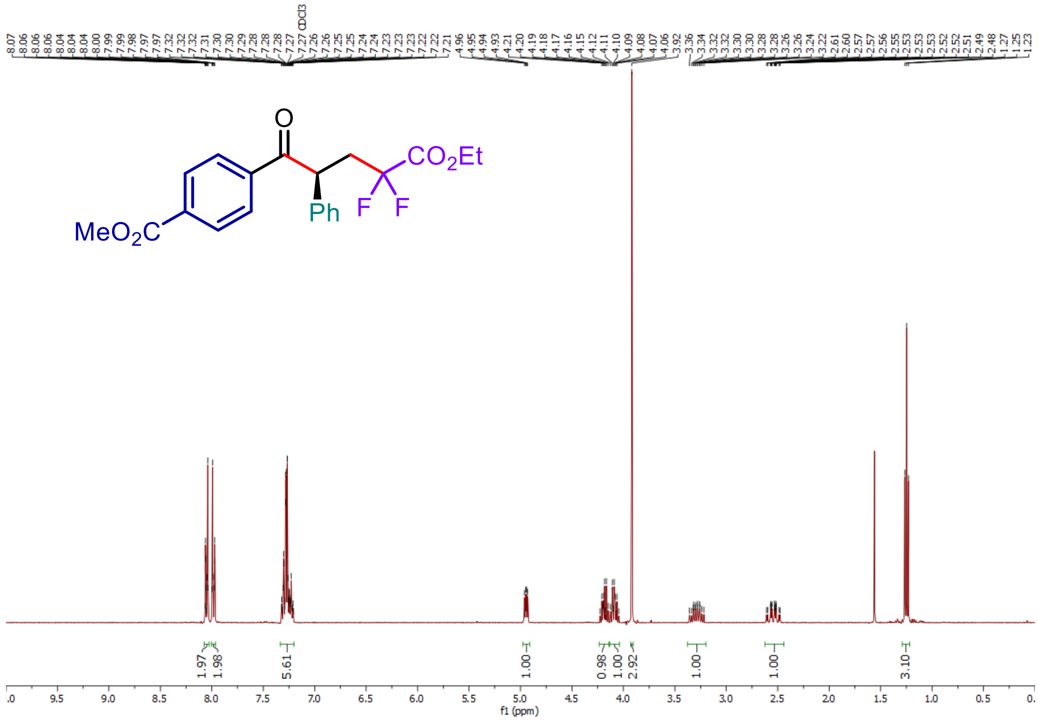

^{13}C NMR (101 MHz, $CDCl_3$):

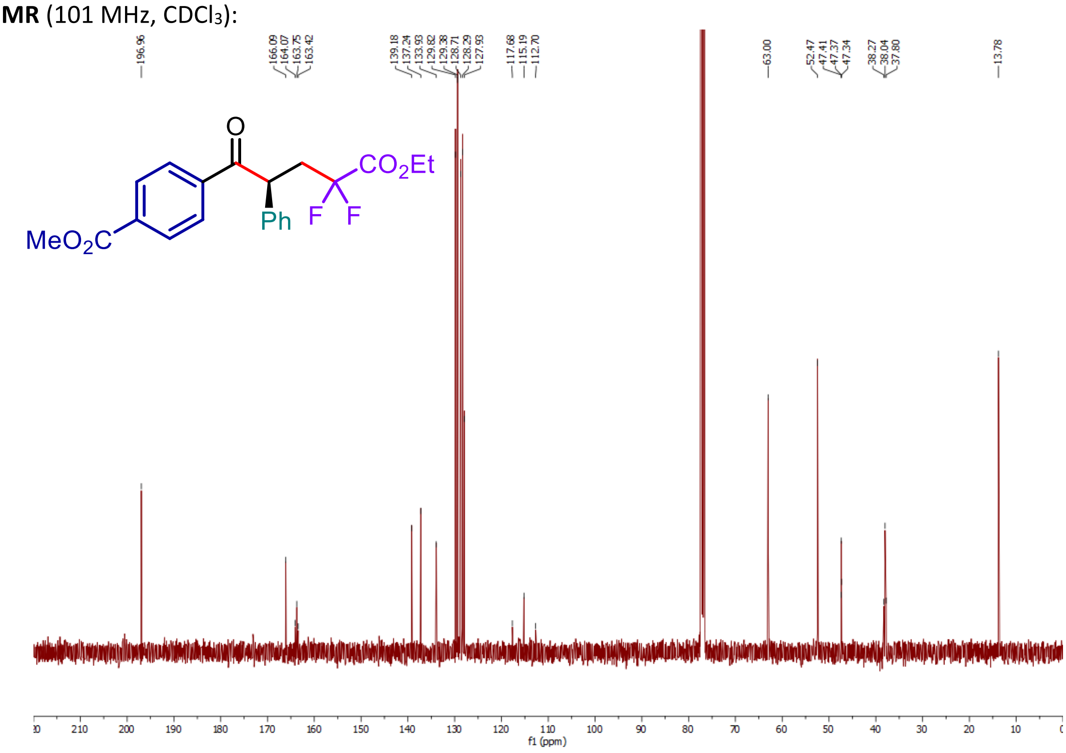

^{19}F NMR (376 MHz, $CDCl_3$):

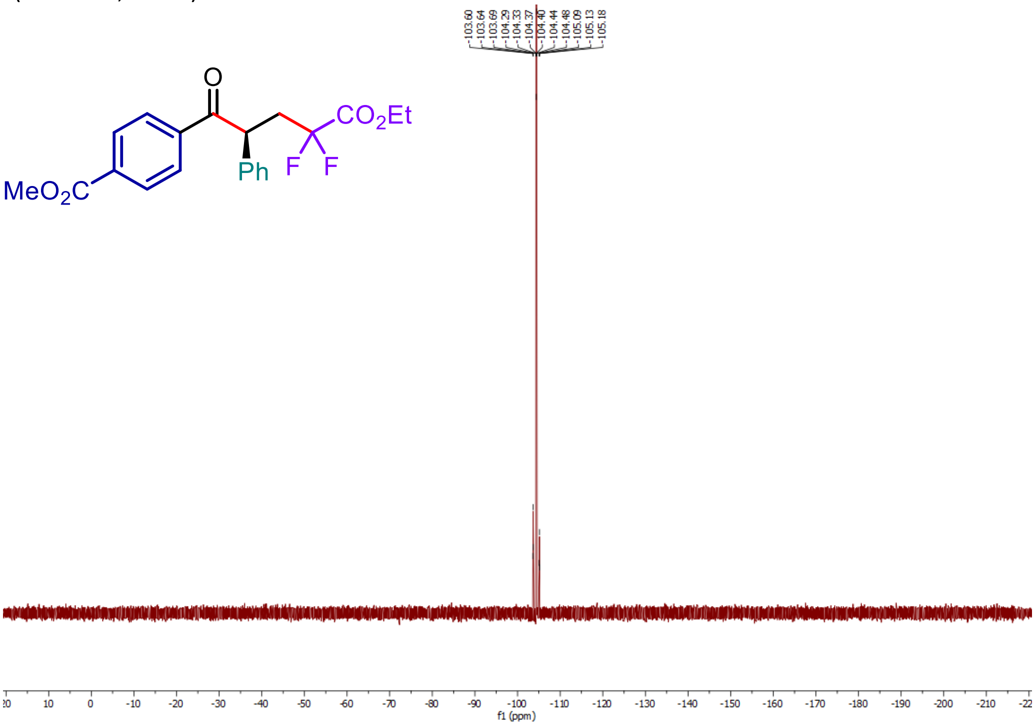

Ethyl (*S*)-2,2-difluoro-5-oxo-4-phenyl-5-(4-(trifluoromethoxy)phenyl)pentanoate (4g)

^{1}**H NMR** (400 MHz, Chloroform-*d*):

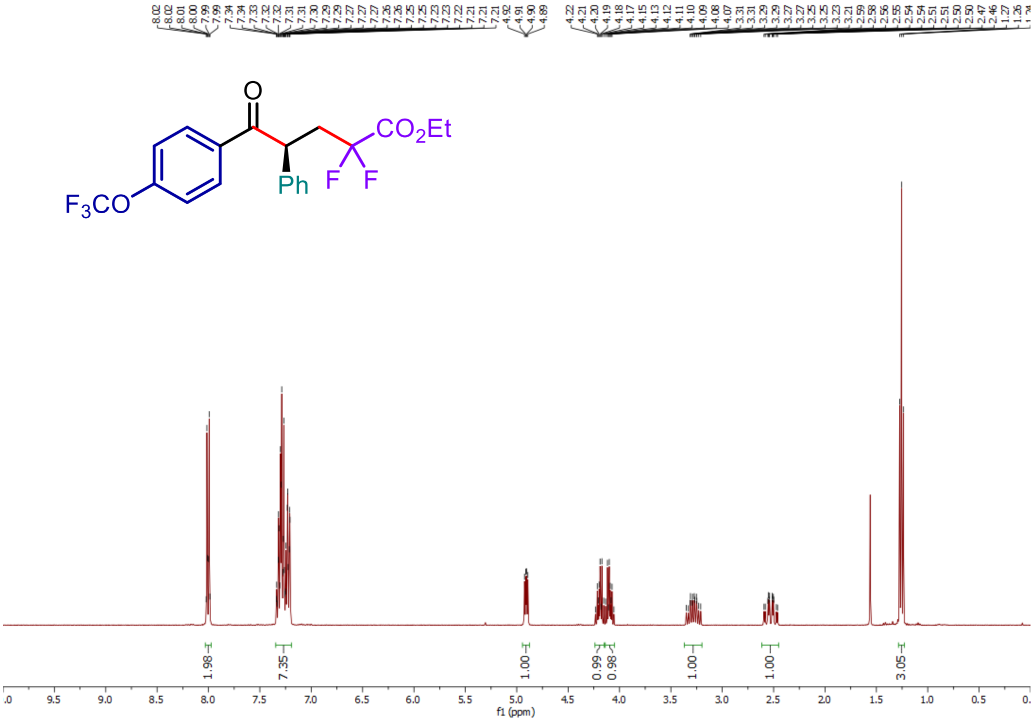

^{13}**C NMR** (101 MHz, Chloroform-*d*):

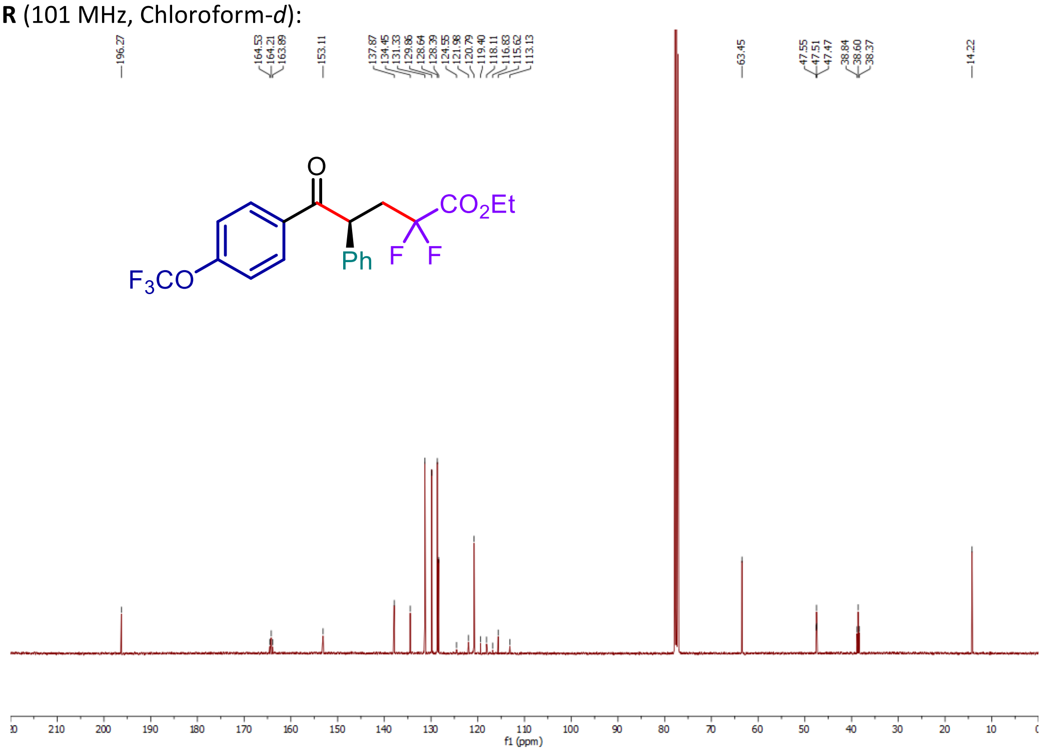

19F NMR (376 MHz, Chloroform-*d*):

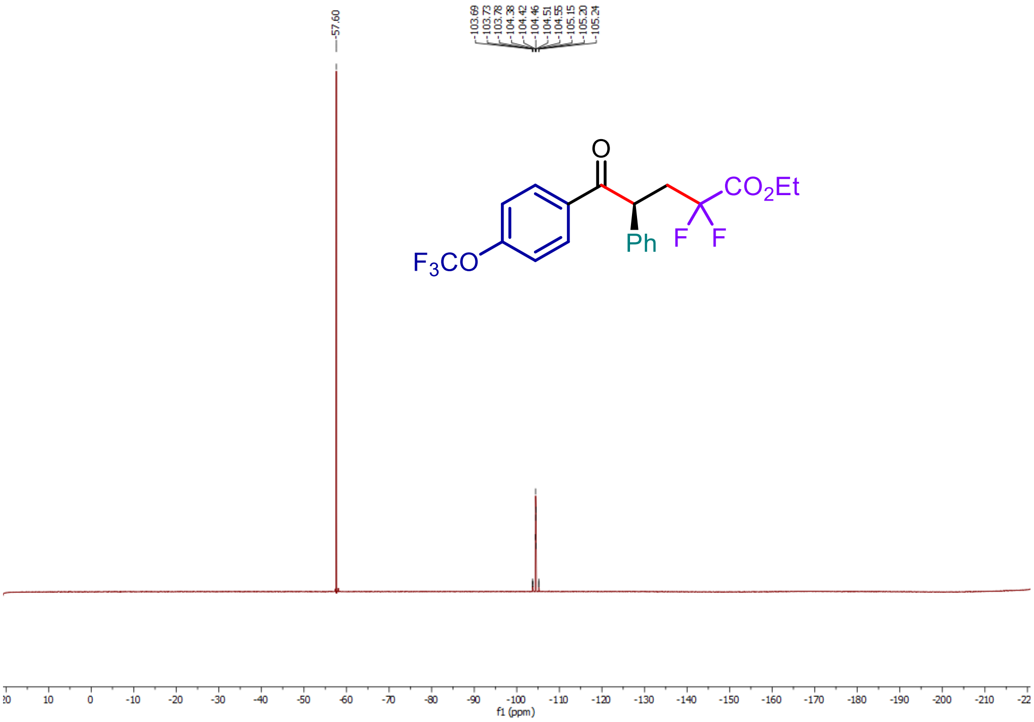

Ethyl (*S*)-2,2-difluoro-5-oxo-4-phenyl-5-(4-(trifluoromethyl)phenyl)pentanoate (4h)

^{1}H NMR (400 MHz, Chloroform-*d*):

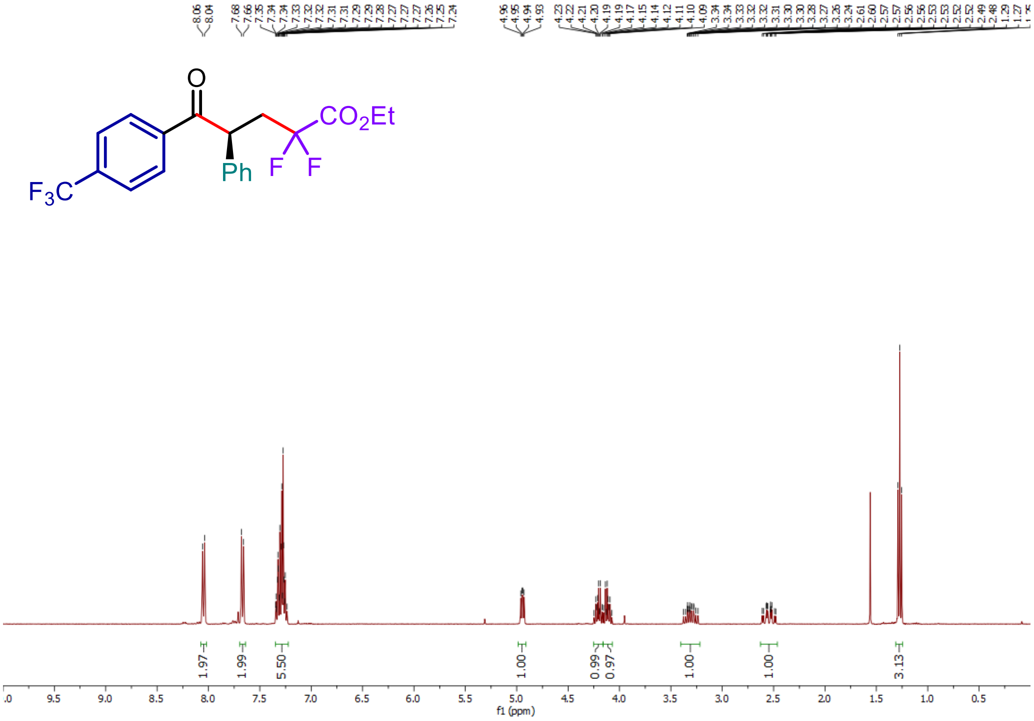

^{13}C NMR (101 MHz, Chloroform-*d*):

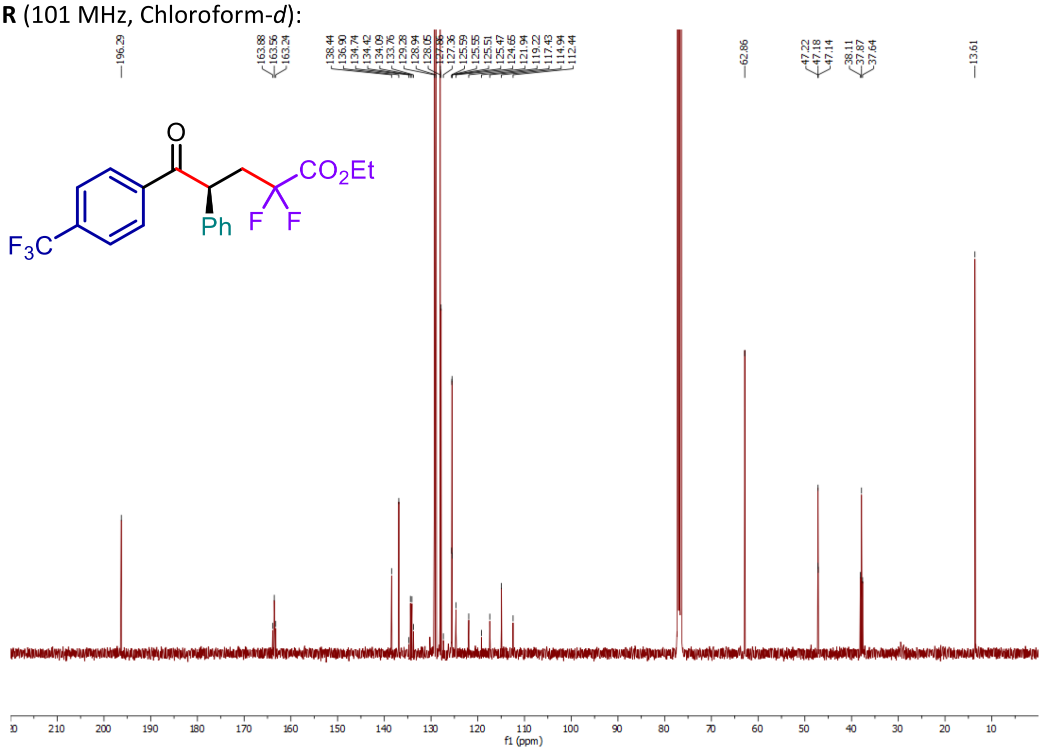

^{19}F NMR (376 MHz, Chloroform-*d*):

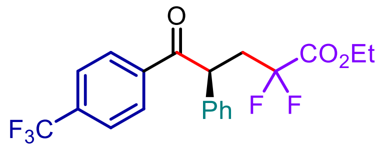

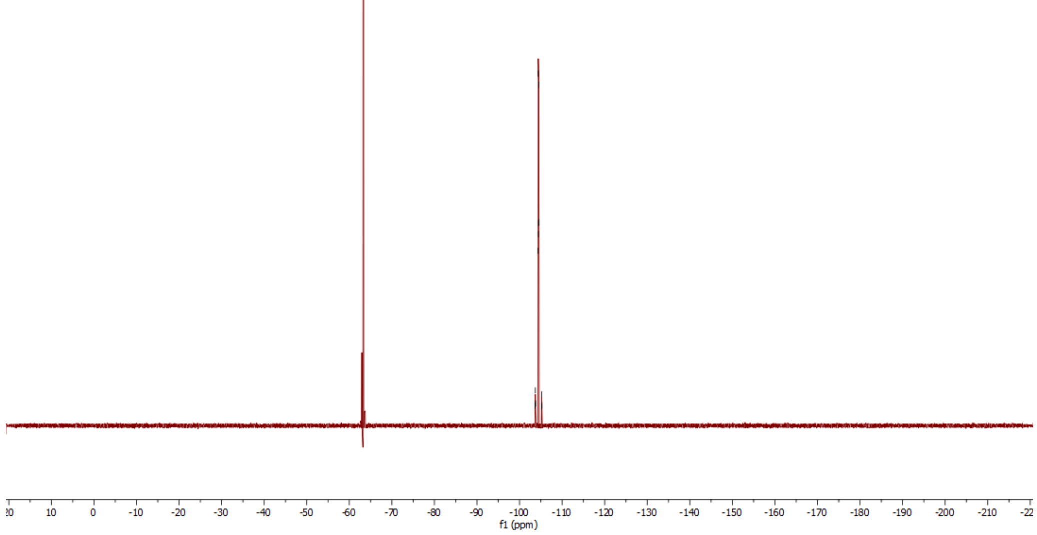

Ethyl (*S*)-2,2-difluoro-5-(4-nitrophenyl)-5-oxo-4-phenylpentanoate (4i)

^{1}H NMR (400 MHz, Chloroform-*d*):

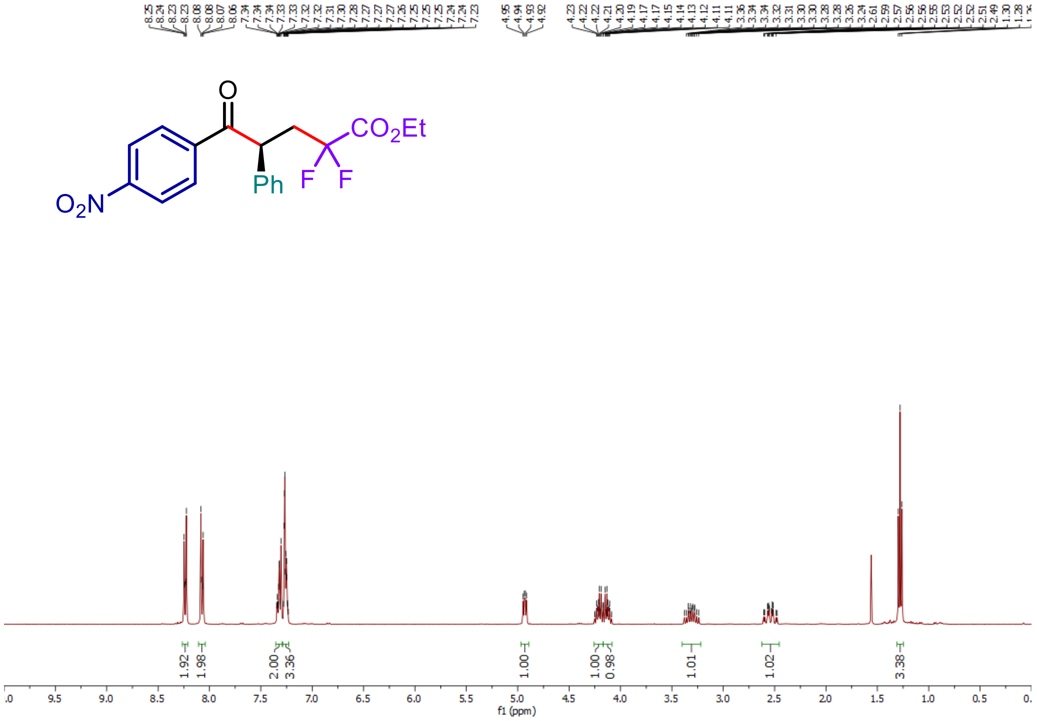

^{13}C NMR (101 MHz, Chloroform-*d*):

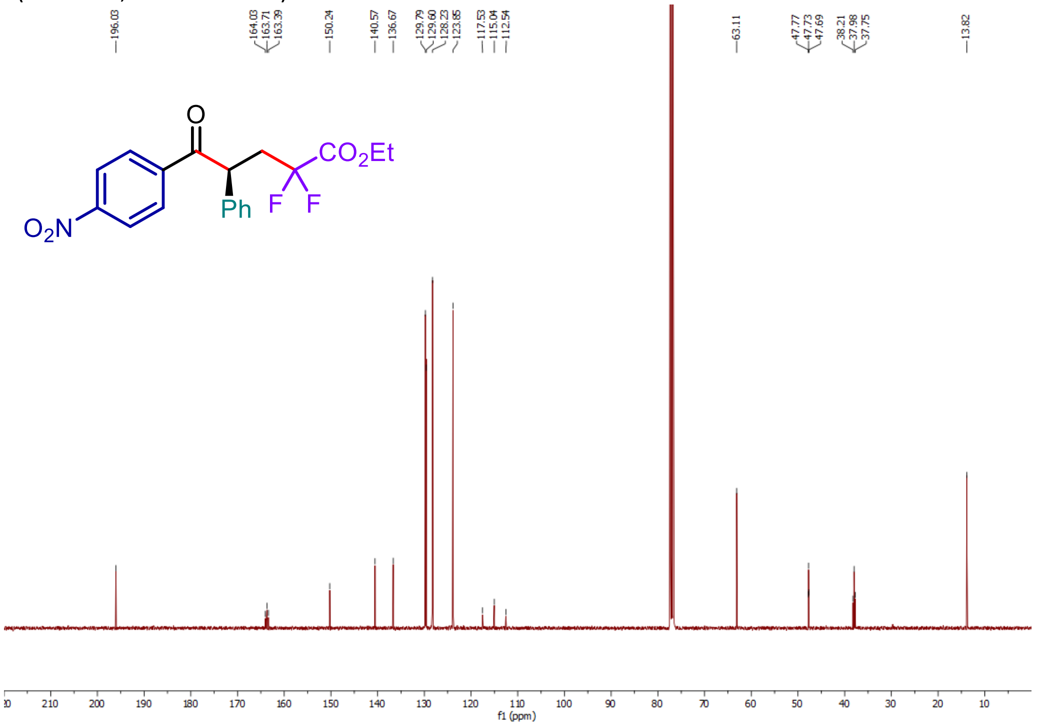

^{19}F NMR (376 MHz, Chloroform-*d*):

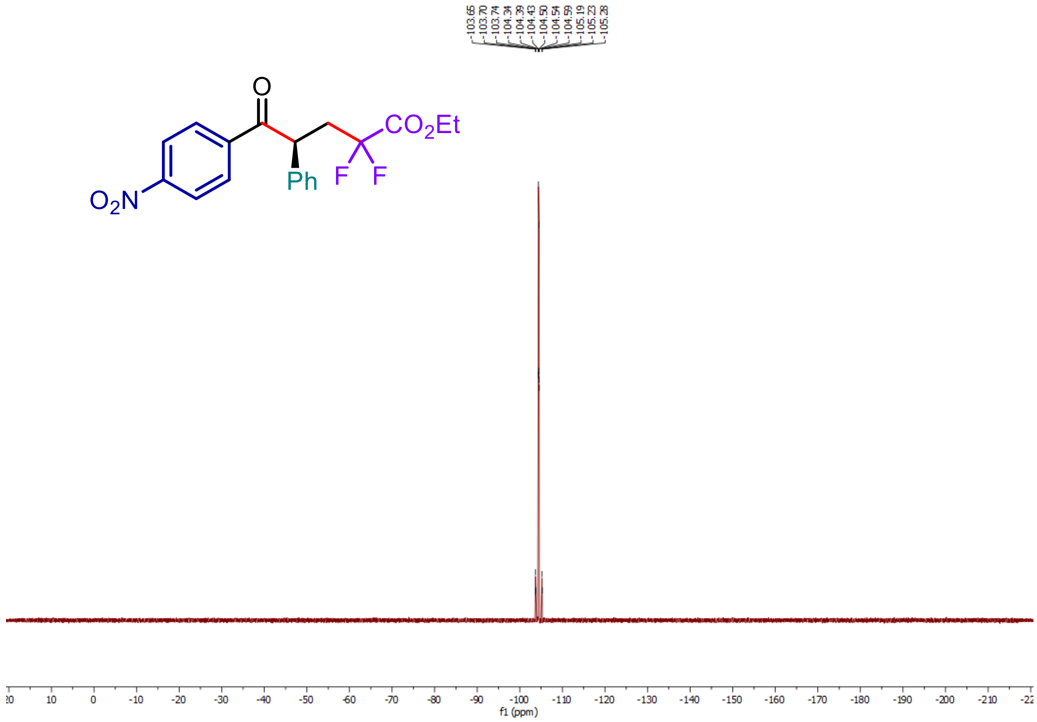

Ethyl (*S*)-2,2-difluoro-5-(3-fluorophenyl)-5-oxo-4-phenylpentanoate (4j)

^{1}H NMR (400 MHz, Chloroform-*d*):

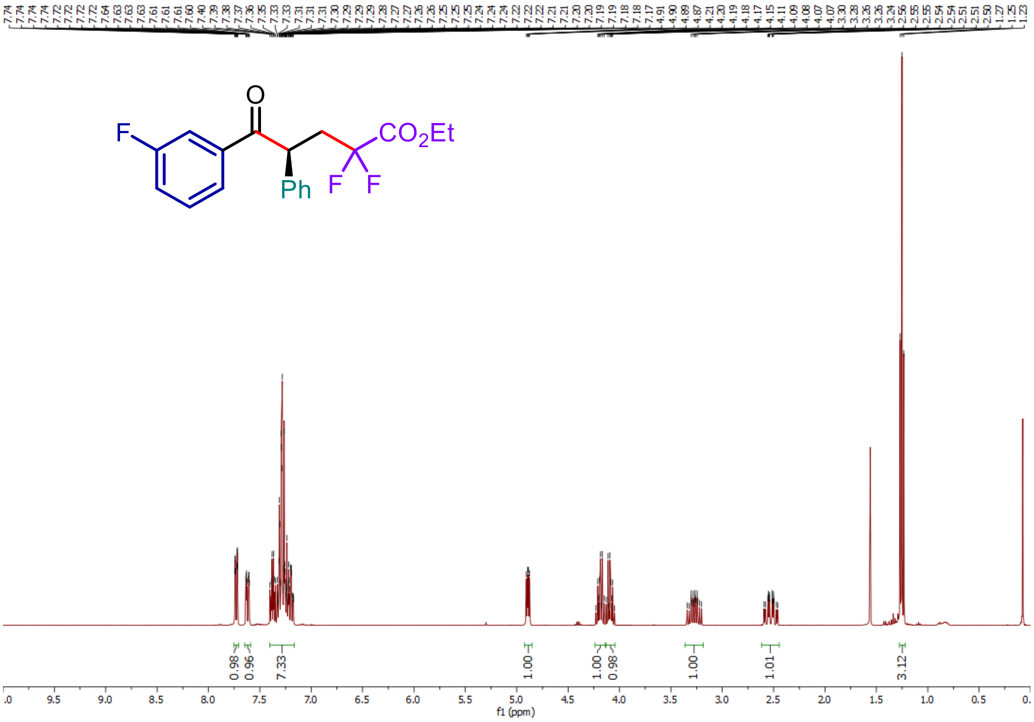

^{13}C NMR (101 MHz, Chloroform-*d*):

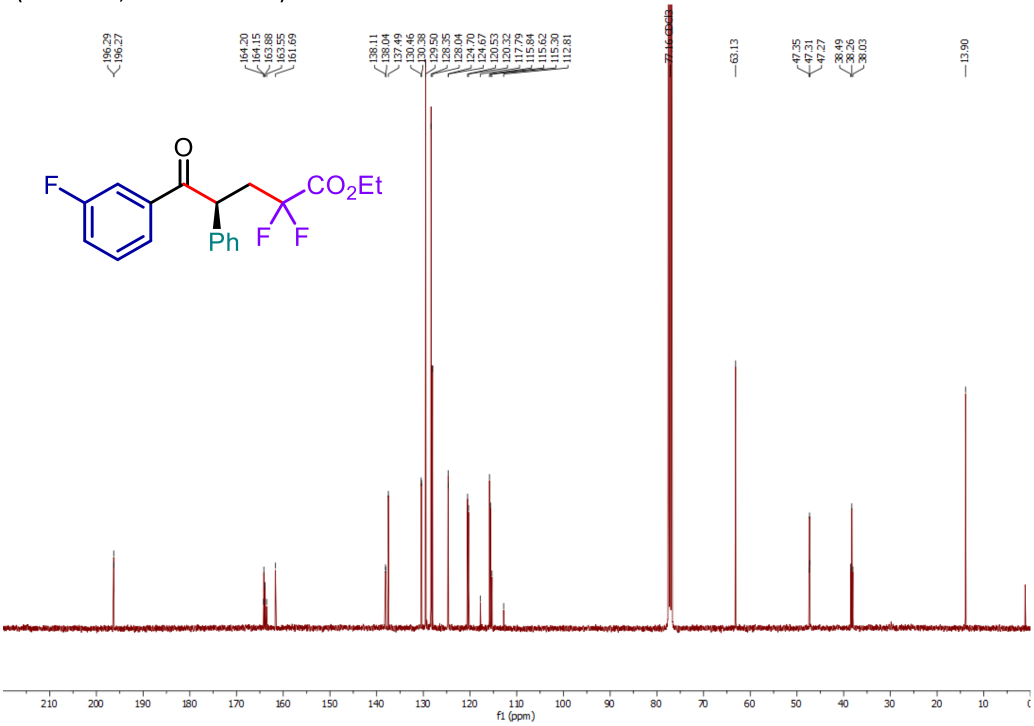

^{19}F NMR (376 MHz, Chloroform-*d*):

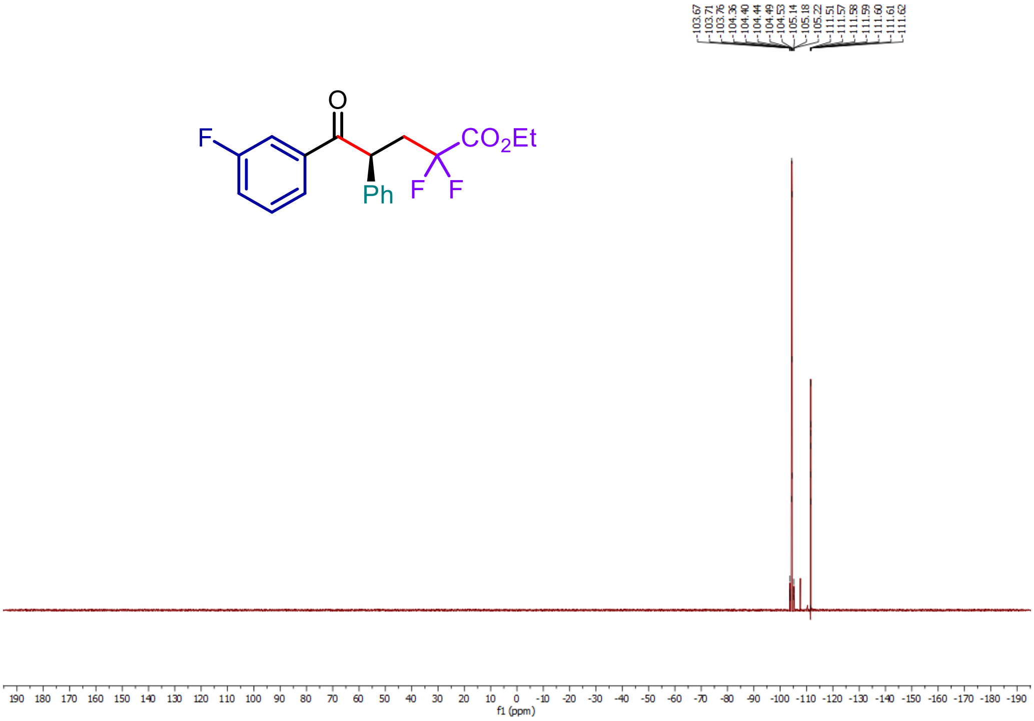

Ethyl (*S*)-2,2-difluoro-5-(3-methoxyphenyl)-5-oxo-4-phenylpentanoate (4k)

^{1}**H NMR** (400 MHz, $CDCl_3$):

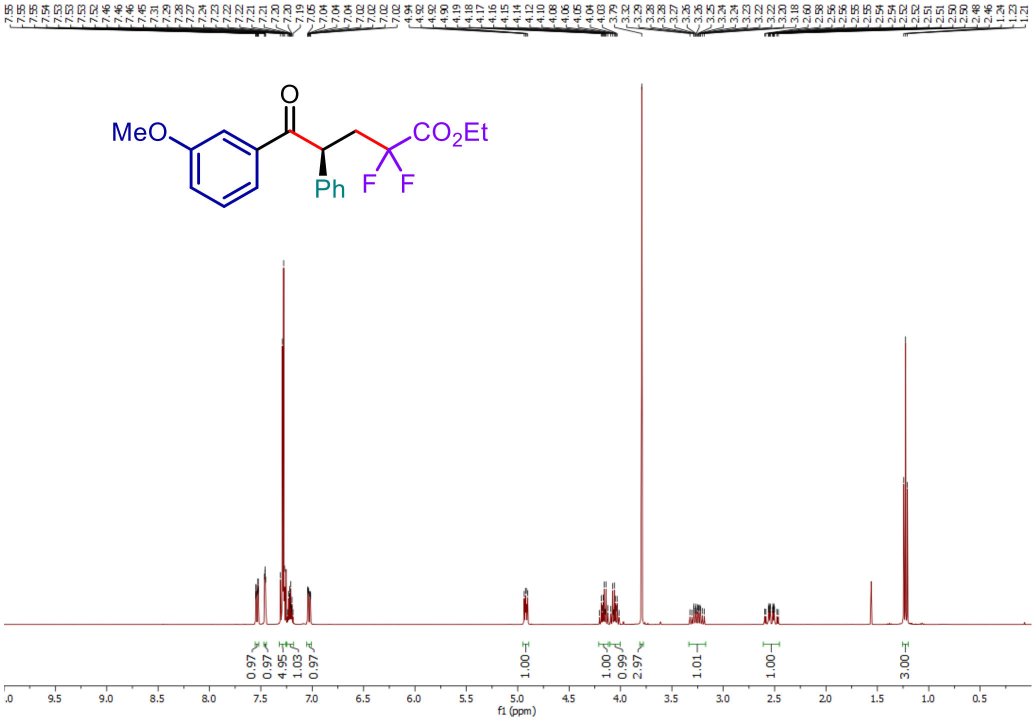

^{13}**C NMR** (101 MHz, $CDCl_3$):

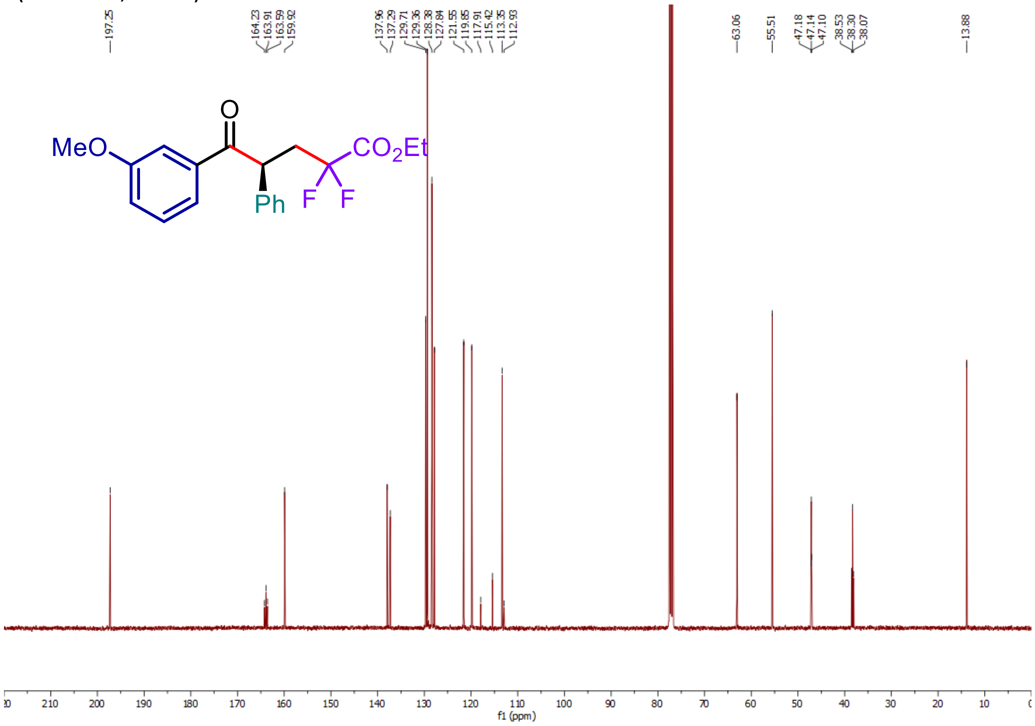

^{19}F NMR (376 MHz, $CDCl_3$):

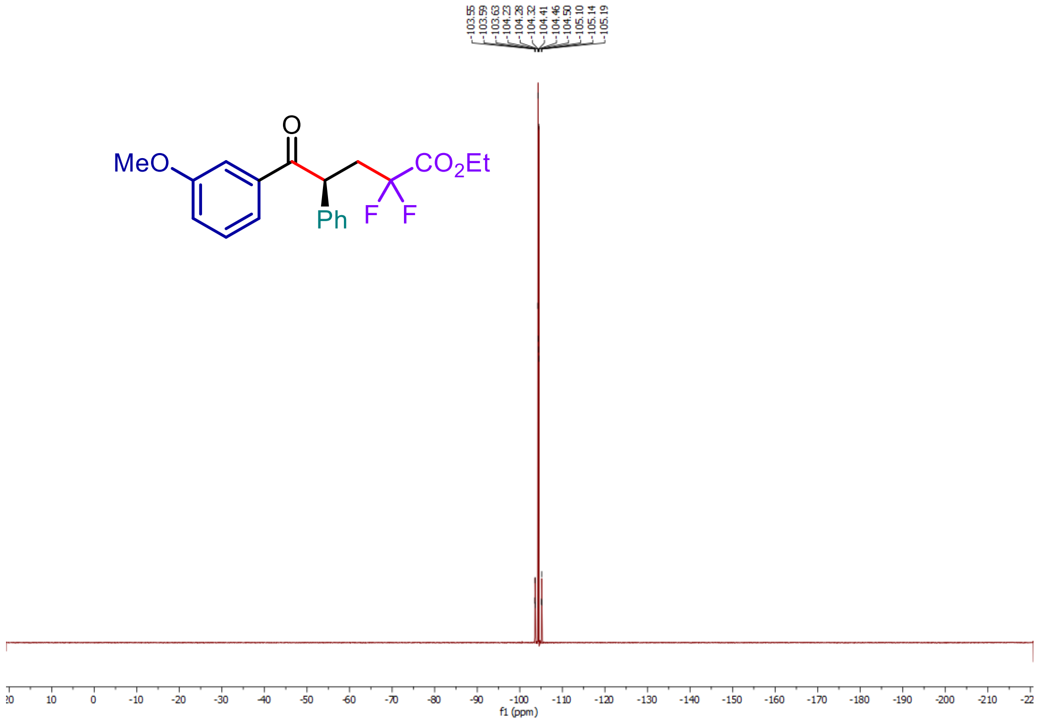

Ethyl (*S*)-5-(3,5-di-*tert*-butylphenyl)-2,2-difluoro-5-oxo-4-phenylpentanoate (4l)

^{1}H NMR (400 MHz, Chloroform-*d*):

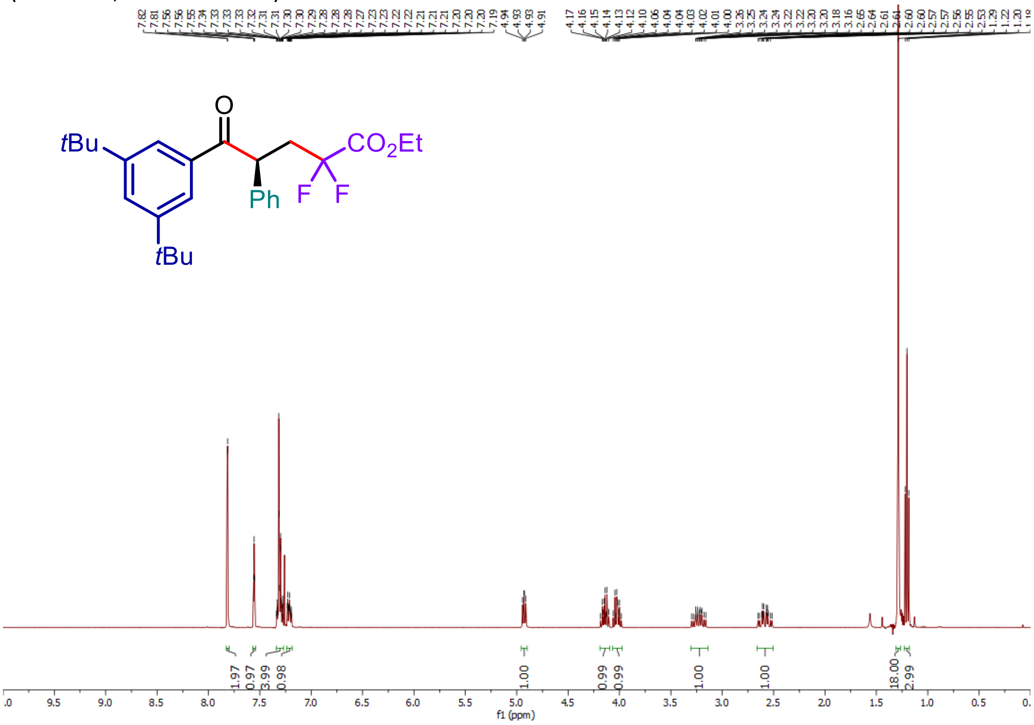

^{13}C NMR (101 MHz, Chloroform-*d*):

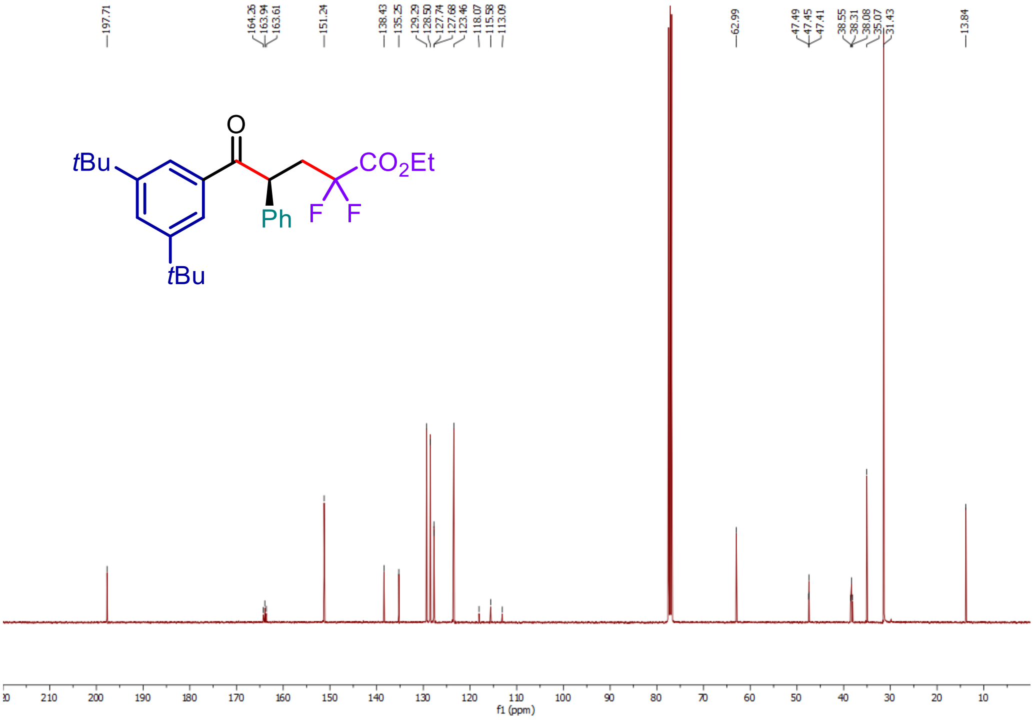

^{19}F NMR (376 MHz, CDCl₃):

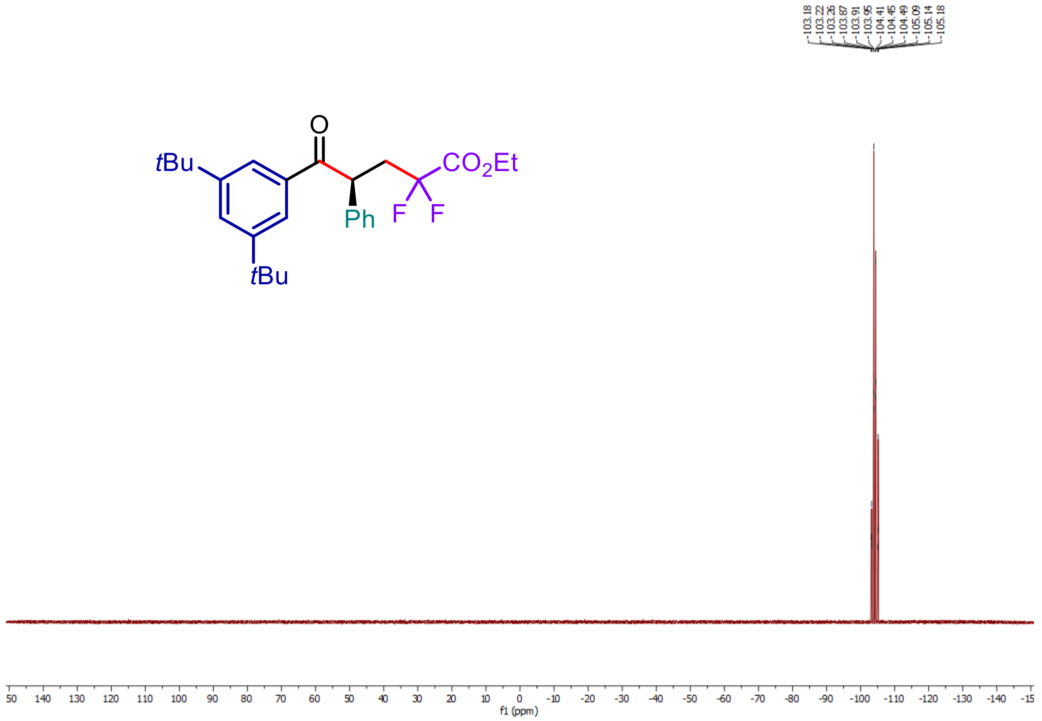

Ethyl (*S*)-2,2-difluoro-5-(2-fluorophenyl)-5-oxo-4-phenylpentanoate (4m)

^{1}H NMR (400 MHz, Chloroform-*d*):

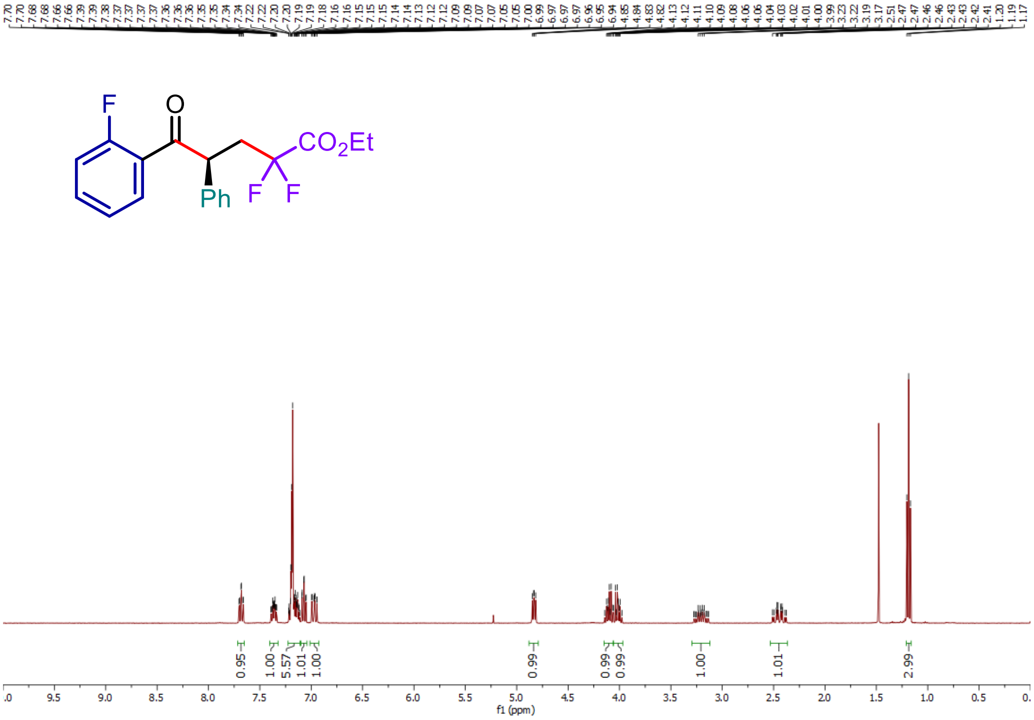

^{13}C NMR (101 MHz, Chloroform-*d*):

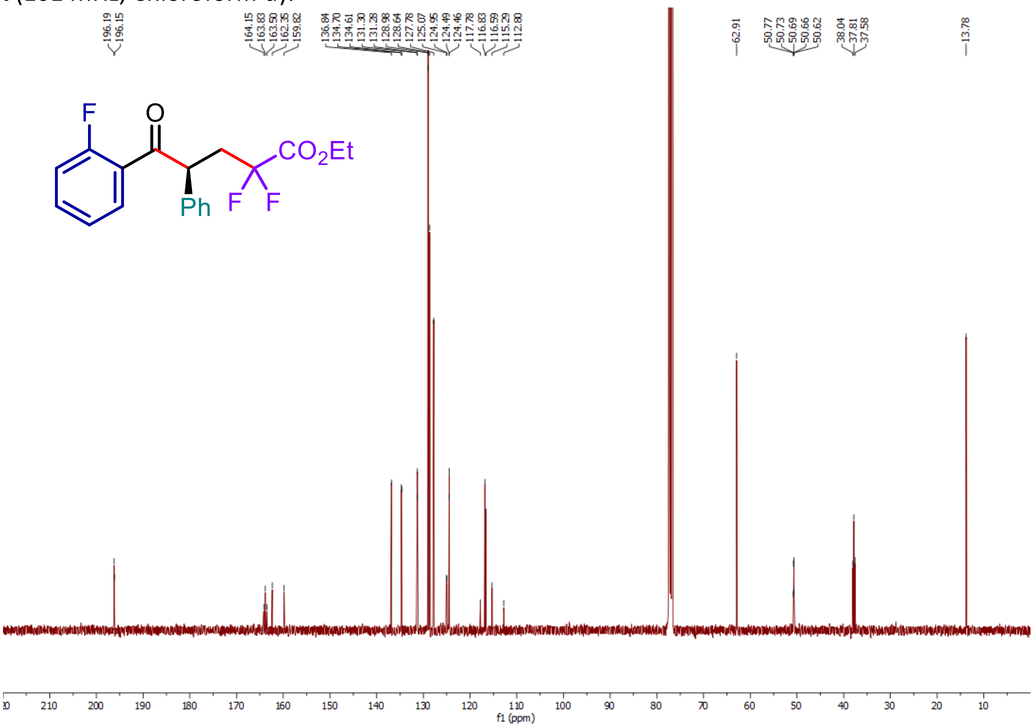

^{19}F NMR (376 MHz, Chloroform-*d*):

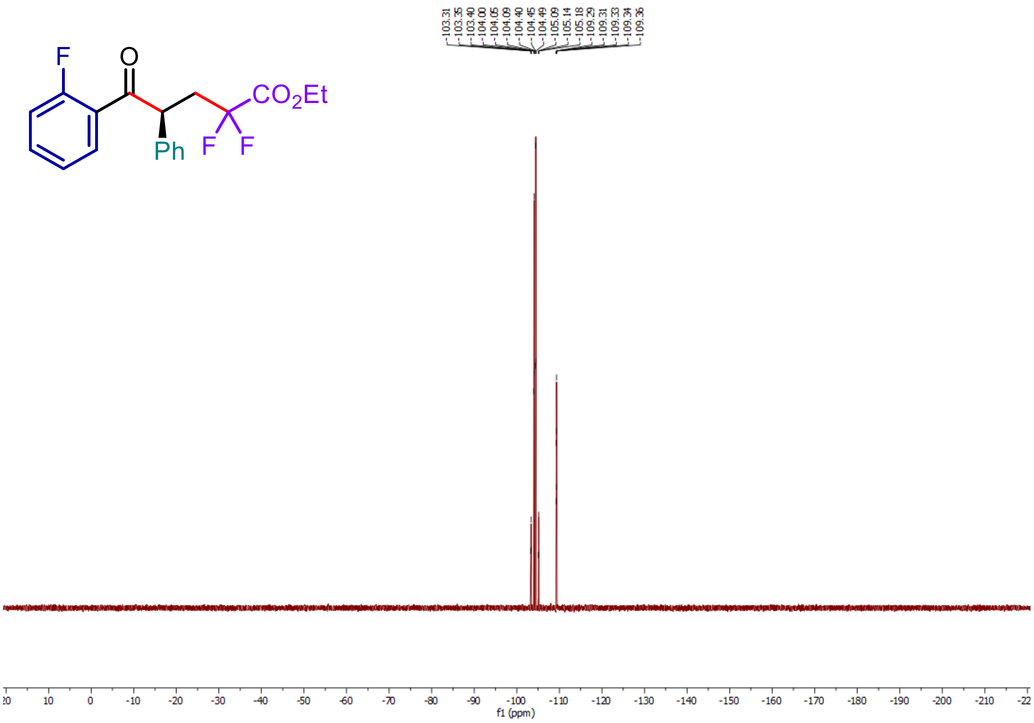

Ethyl (*S*)-2,2-difluoro-5-(2-hydroxyphenyl)-5-oxo-4-phenylpentanoate (4n)

^{1}H NMR (400 MHz, $CDCl_3$):

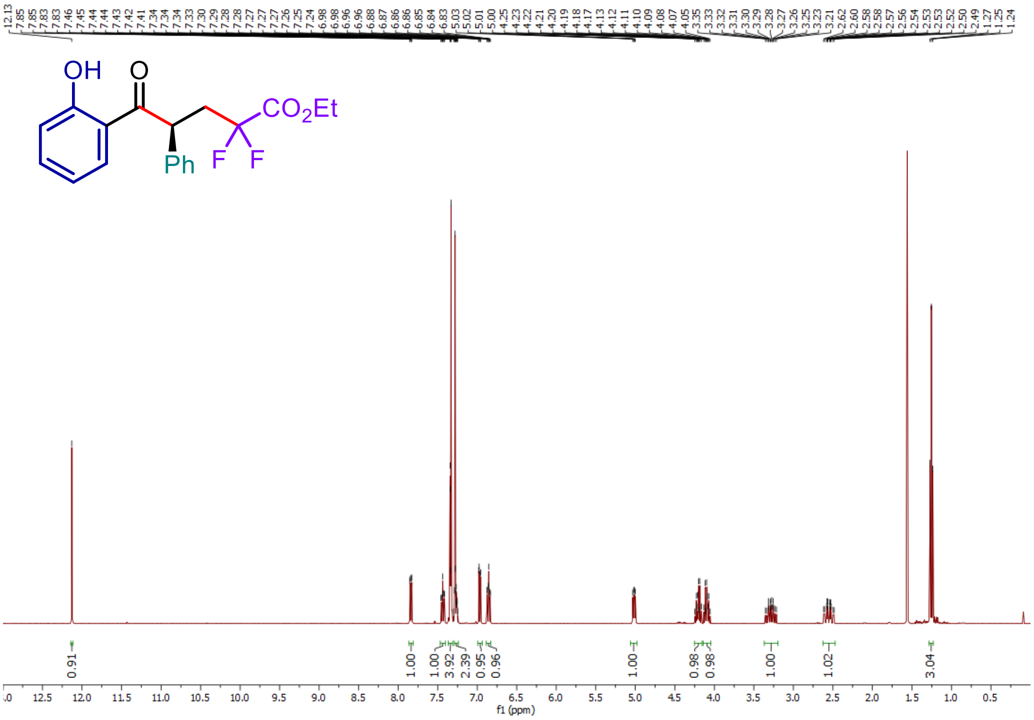

^{13}C NMR (101 MHz, $CDCl_3$):

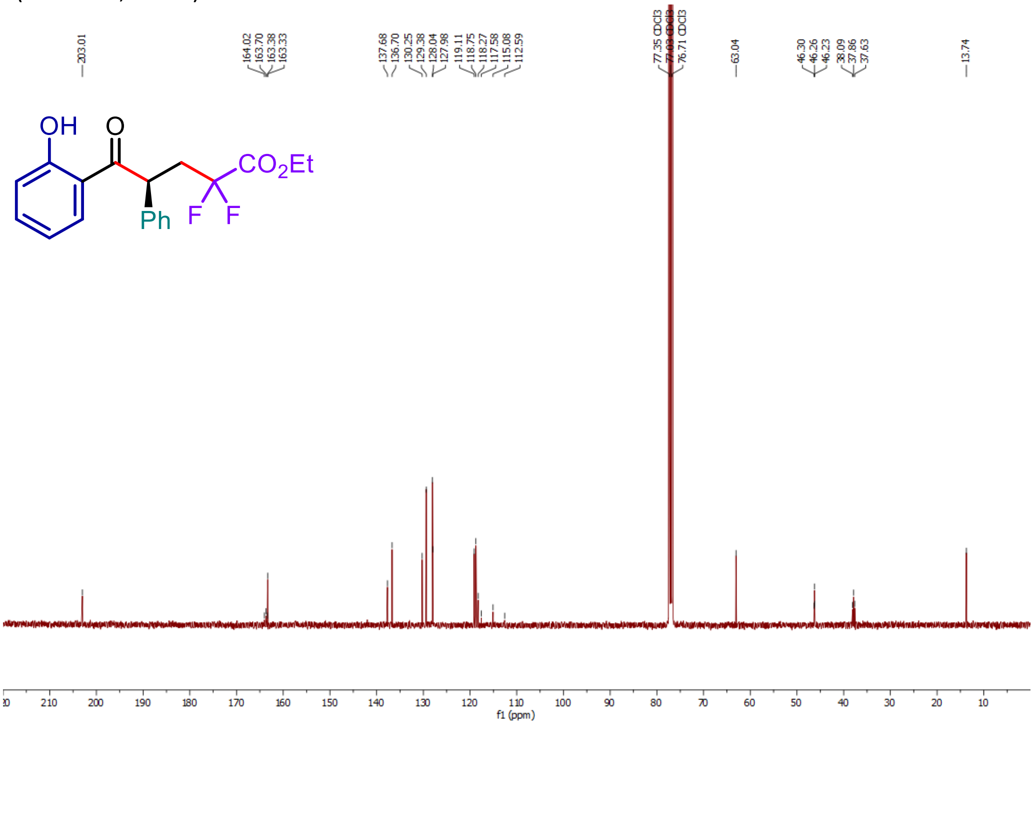

^{19}**F NMR** (376 MHz, $CDCl_3$):

-104.34
-104.36
-104.38
-104.41
-104.43
-104.45

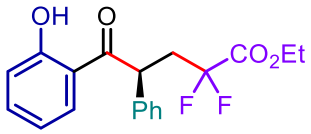

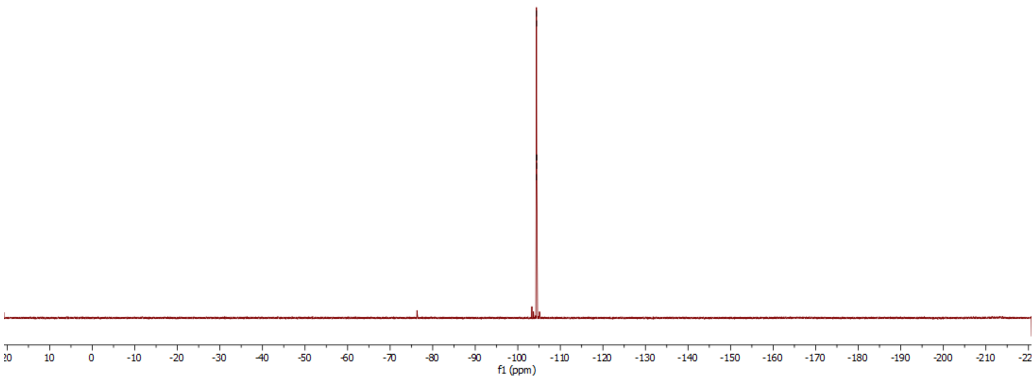

Ethyl (*S*)-2,2-difluoro-5-(naphthalen-2-yl)-5-oxo-4-phenylpentanoate (4o)

^{1}H NMR (400 MHz, Chloroform-*d*):

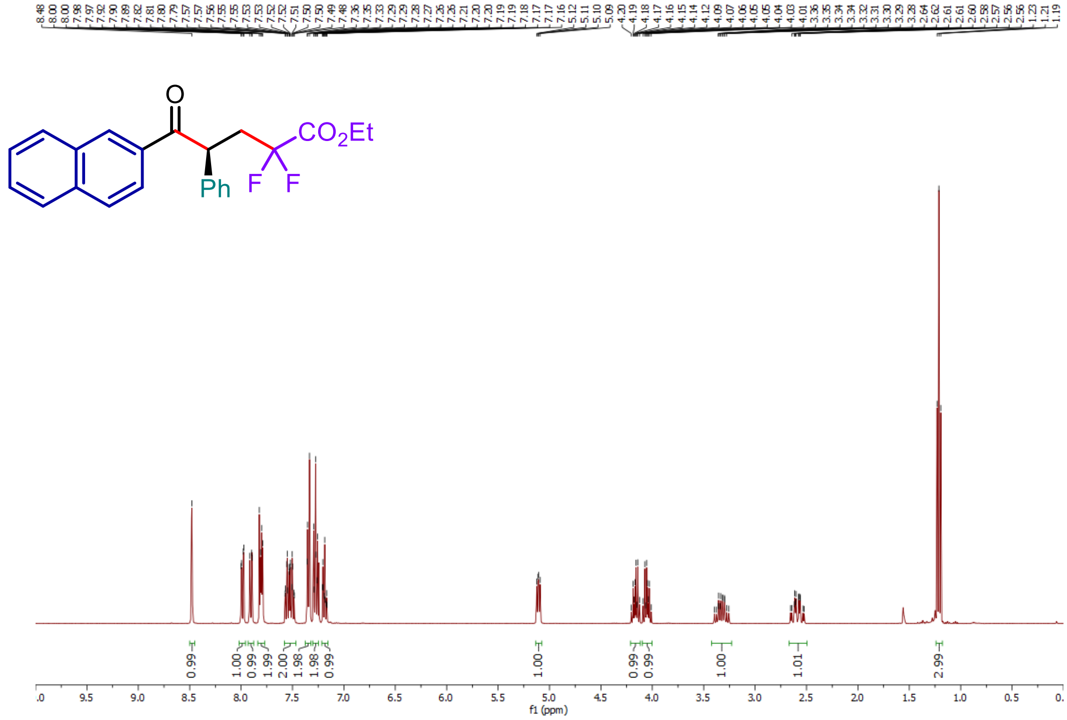

^{13}C NMR (101 MHz, Chloroform-*d*):

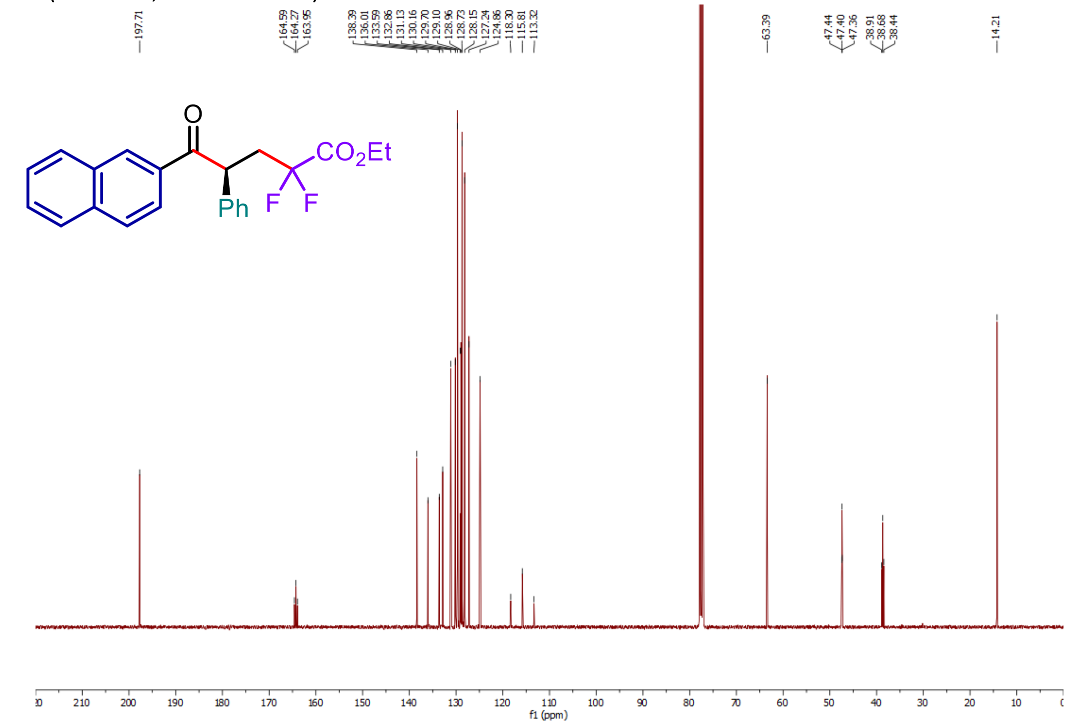

^{19}F NMR (376 MHz, $CDCl_3$):

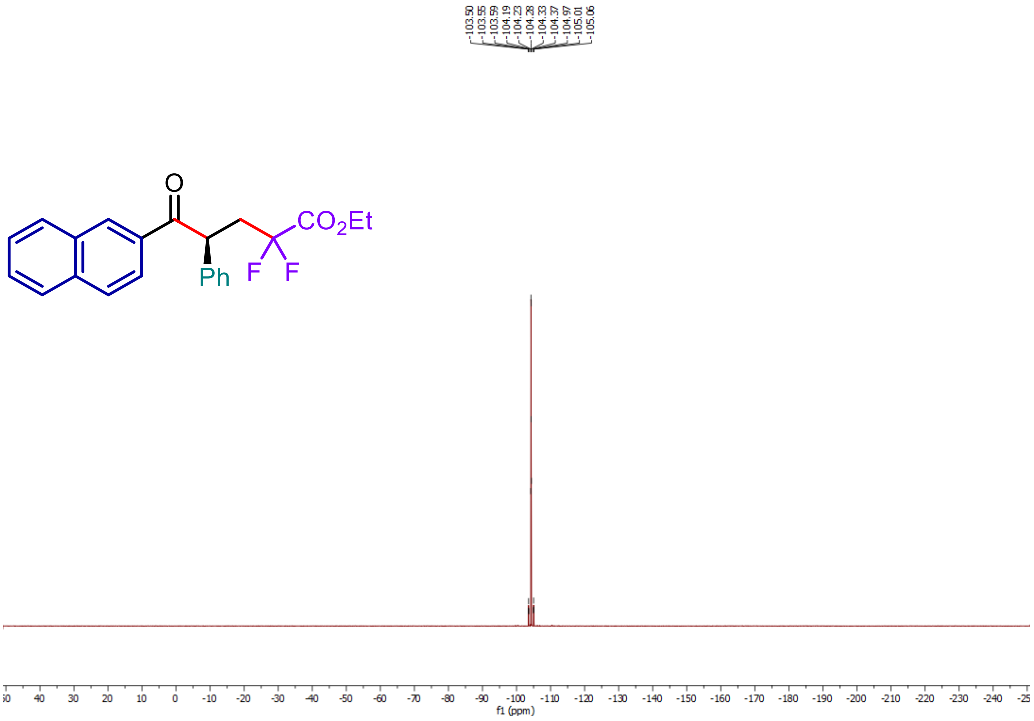

Ethyl (*S*)-2,2-difluoro-5-(naphthalen-1-yl)-5-oxo-4-phenylpentanoate (4p)

^{1}**H NMR** (400 MHz, Chloroform-*d*):

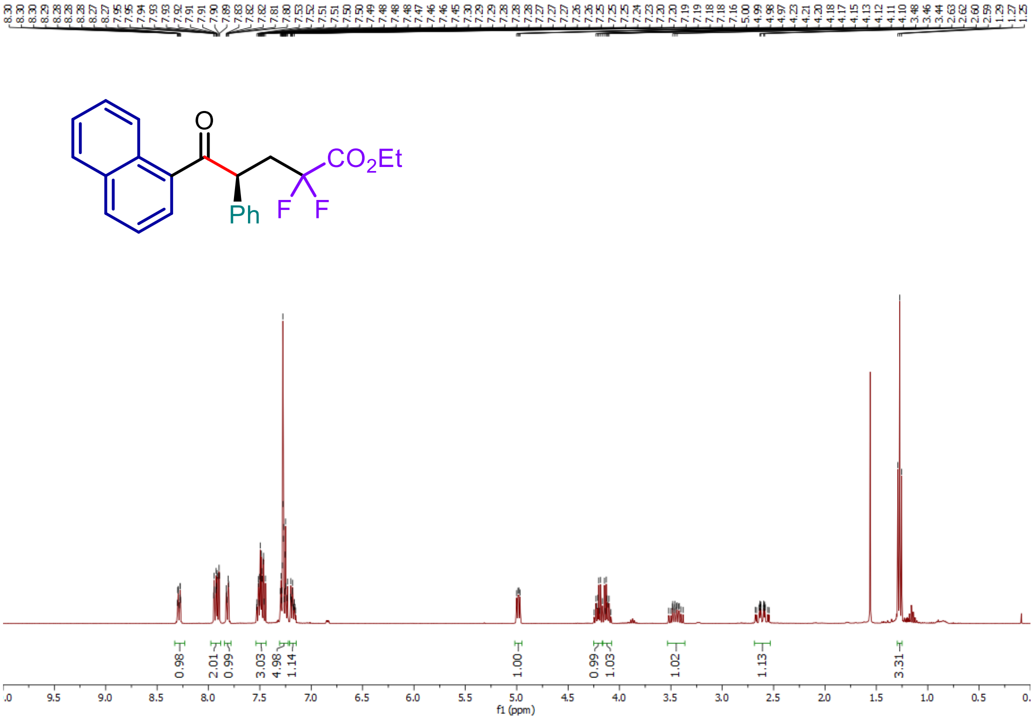

^{13}**C NMR** (101 MHz, Chloroform-*d*):

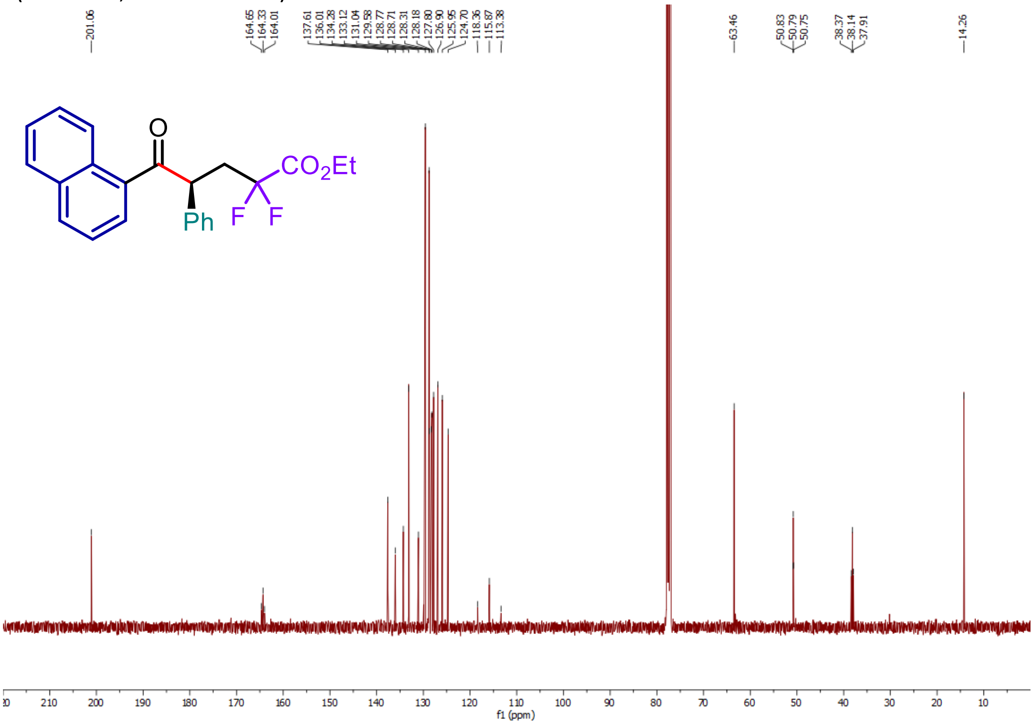

^{19}F NMR (376 MHz, $CDCl_3$):

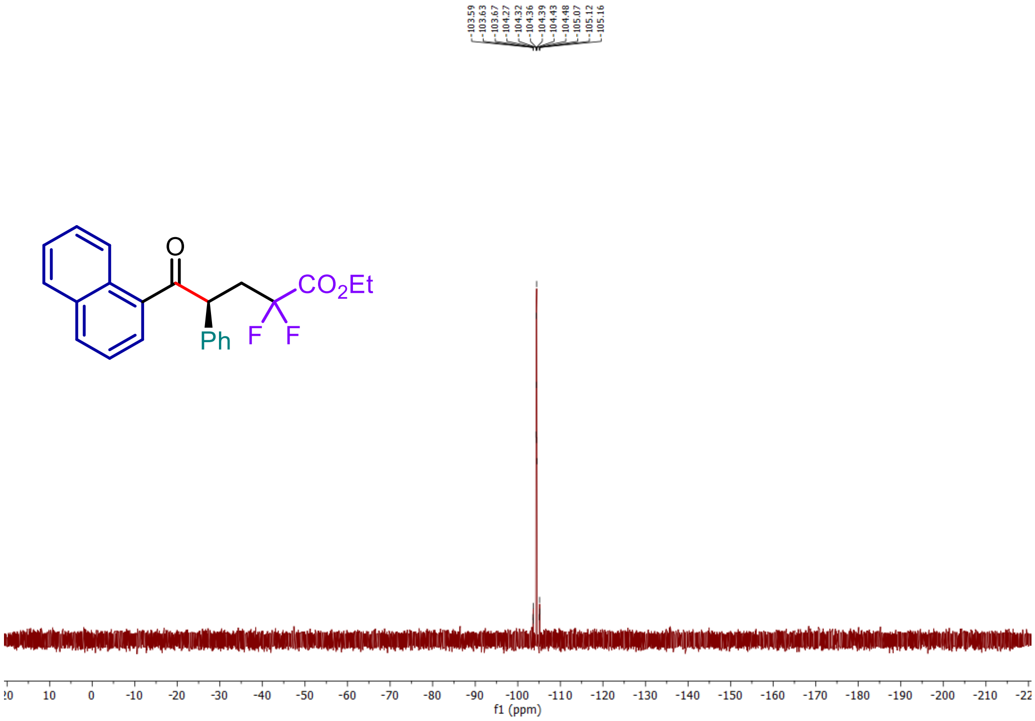

Ethyl (S)-2,2-difluoro-5-oxo-4-phenyl-5-(thiophen-2-yl)pentanoate (4q)

^{1}H NMR (400 MHz, $CDCl_3$):

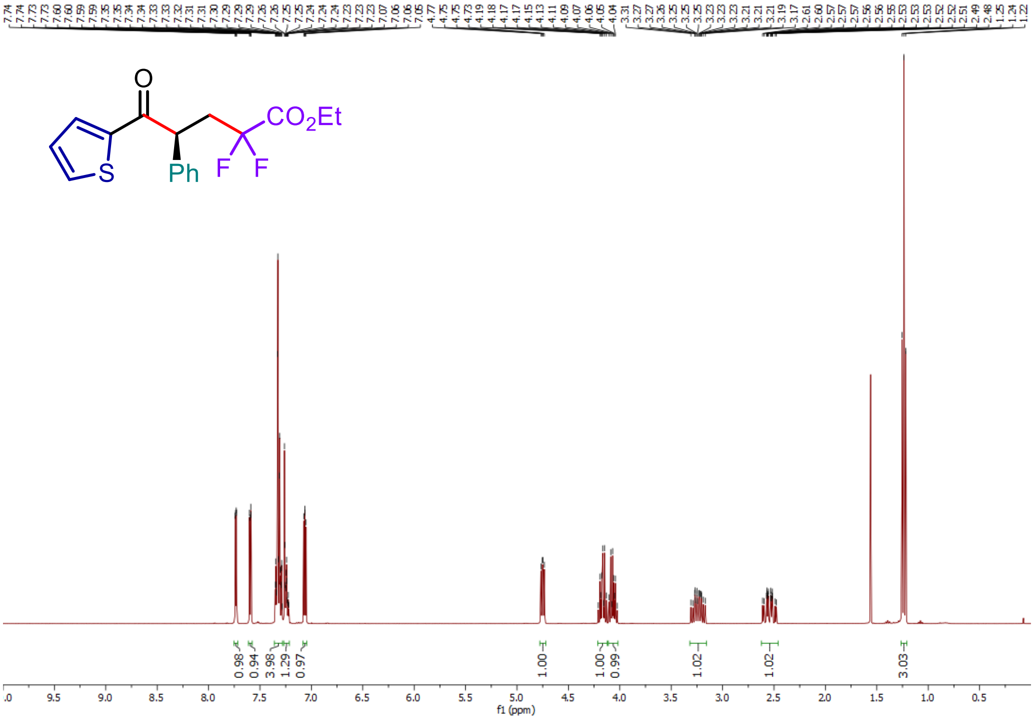

^{13}C NMR (101 MHz, $CDCl_3$):

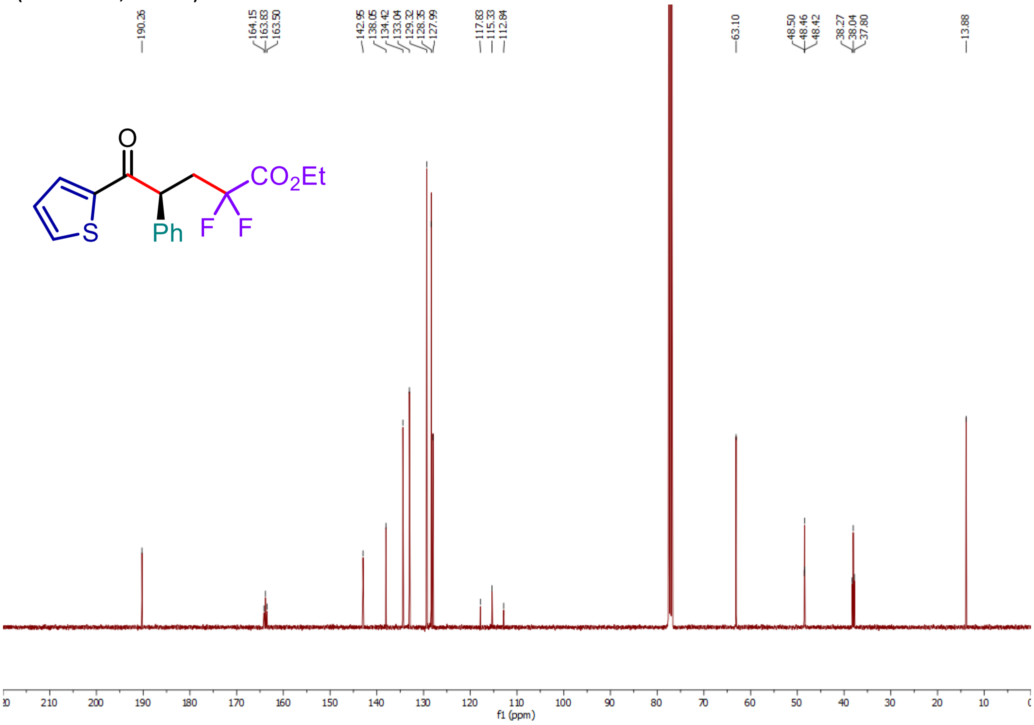

^{19}F NMR (376 MHz, $CDCl_3$):

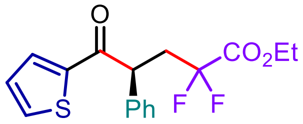

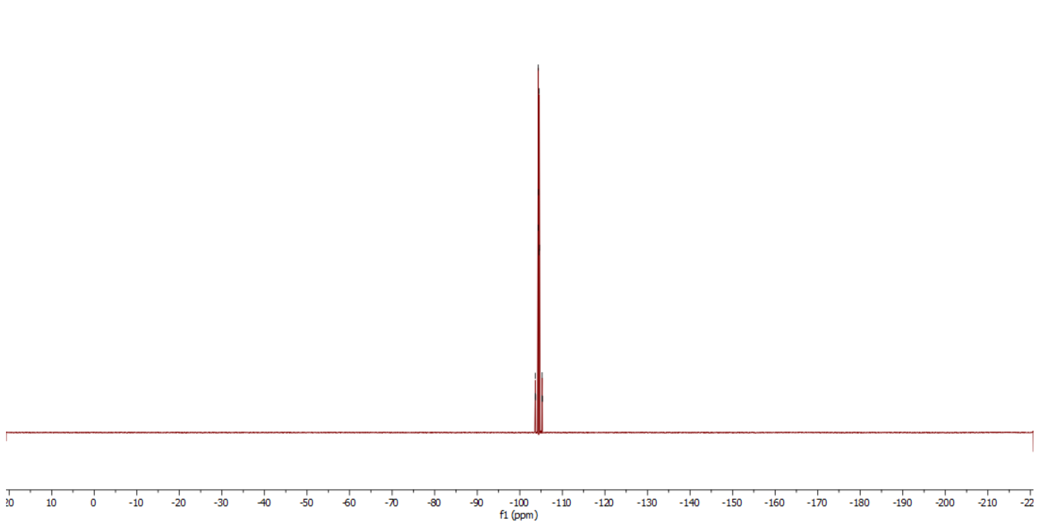

Ethyl (*S*)-2,2-difluoro-5-(furan-2-yl)-5-oxo-4-phenylpentanoate (4r)

^{1}H NMR (400 MHz, $CDCl_3$):

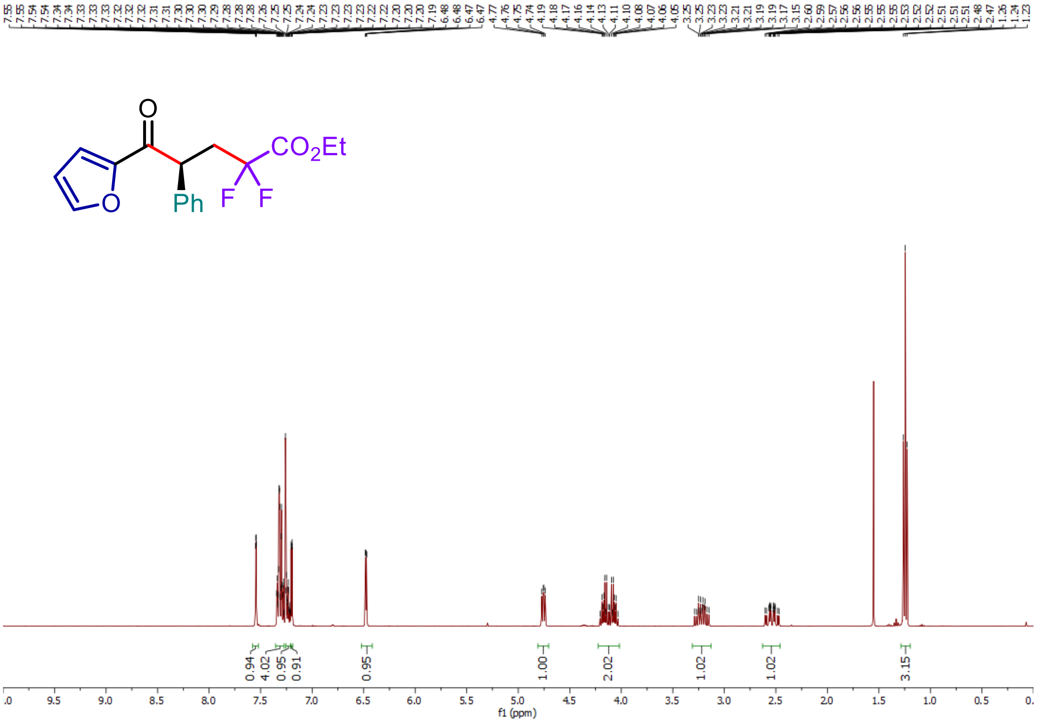

^{13}C NMR (101 MHz, $CDCl_3$):

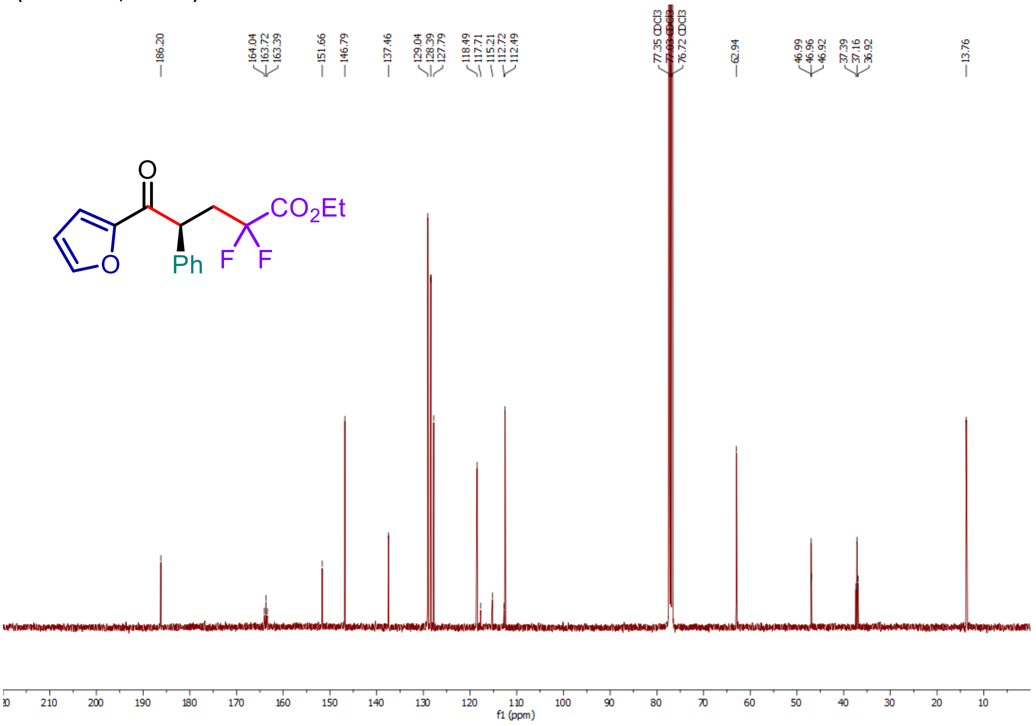

^{19}F NMR (376 MHz, $CDCl_3$):

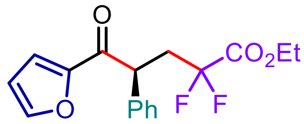

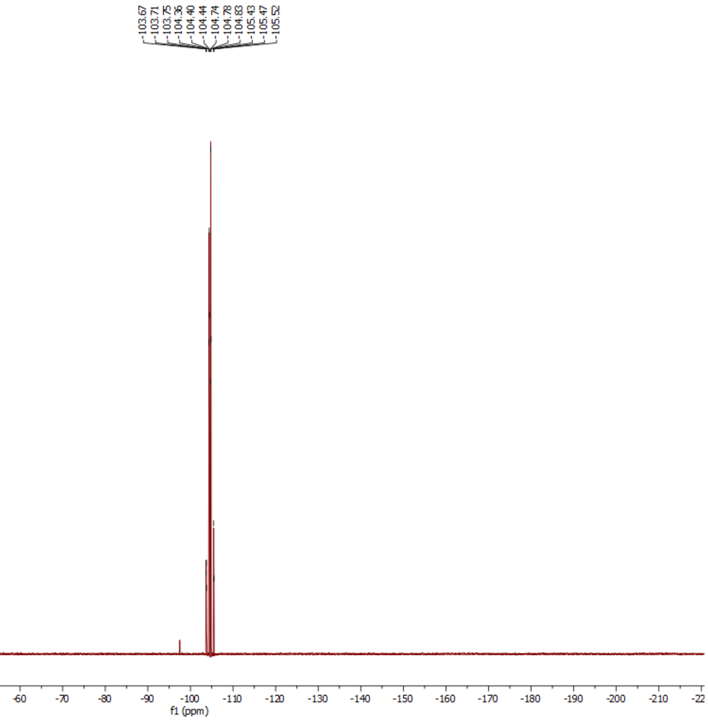

Ethyl (*S*)-2,2-difluoro-5-oxo-4-phenyl-5-(pyridin-3-yl)pentanoate (4s)

^{1}**H NMR** (400 MHz, $CDCl_3$):

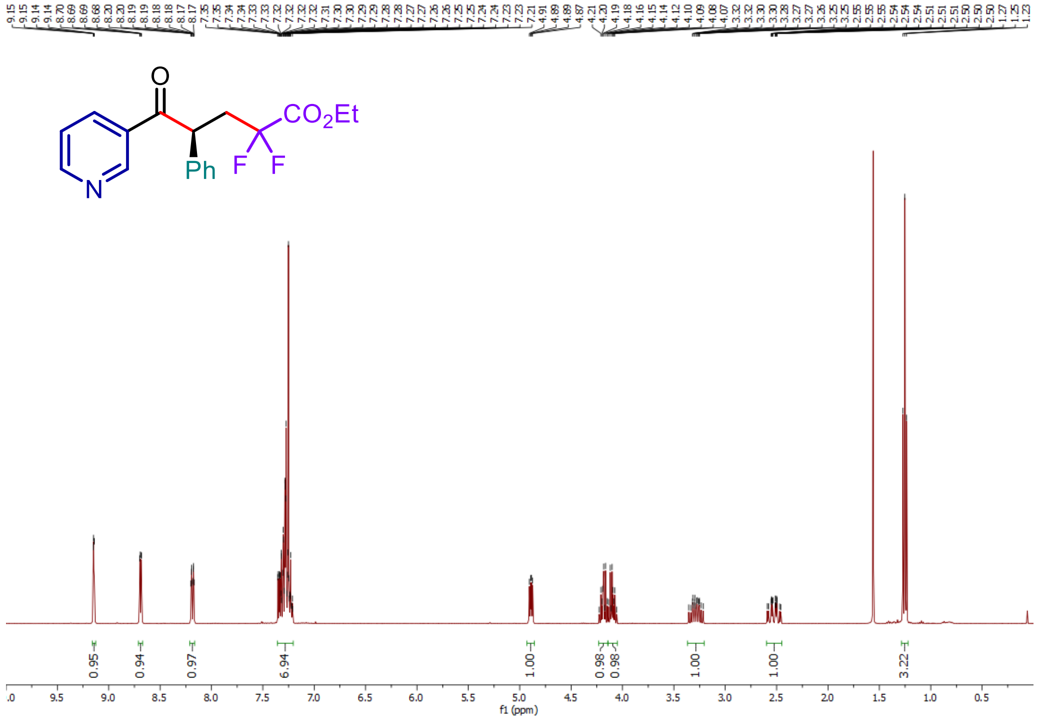

^{13}**C NMR** (101 MHz, $CDCl_3$):

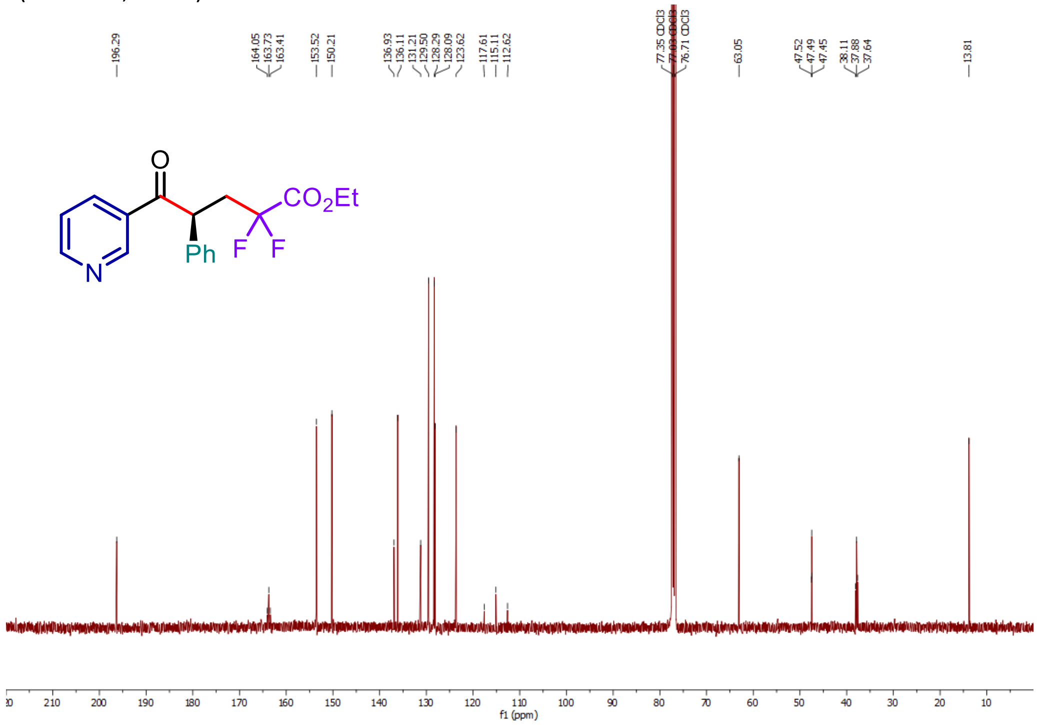

^{19}F NMR (376 MHz, $CDCl_3$):

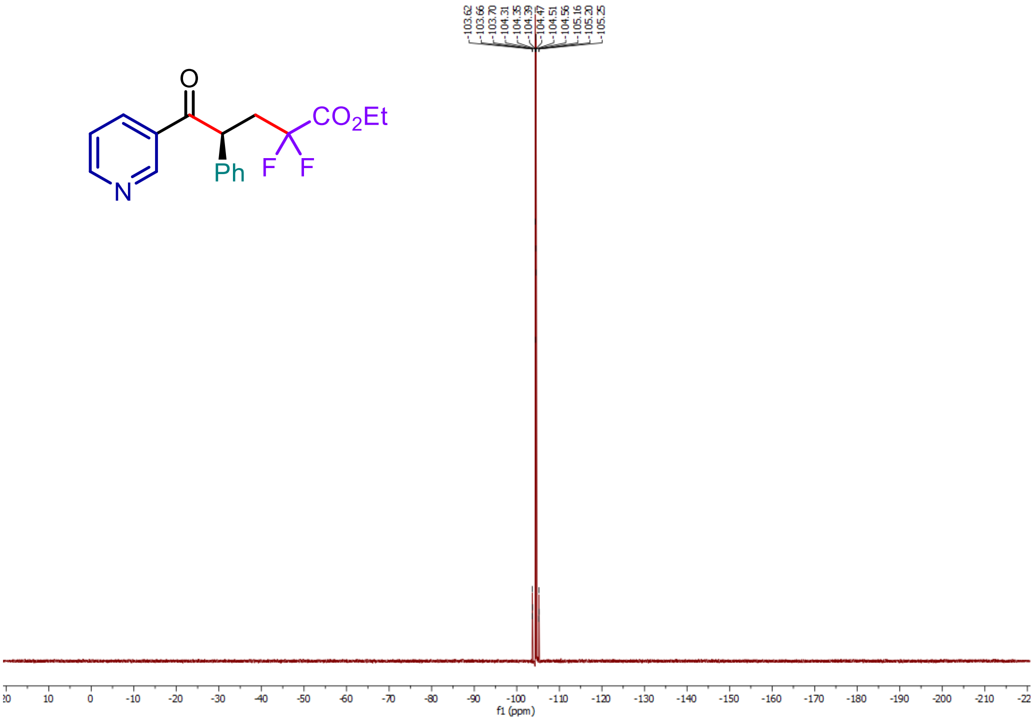

Ethyl (*S*)-5-(9-ethyl-9*H*-carbazol-3-yl)-2,2-difluoro-5-oxo-4-phenylpentanoate (4t)

^{1}H NMR (400 MHz, Chloroform-*d*):

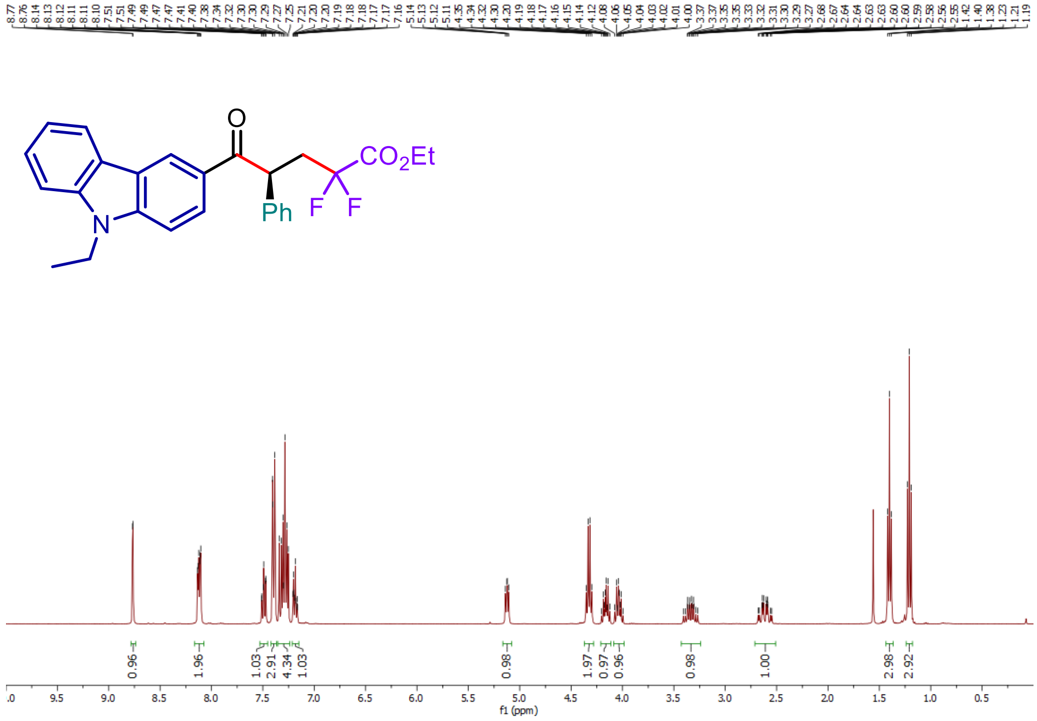

^{13}C NMR (101 MHz, Chloroform-*d*):

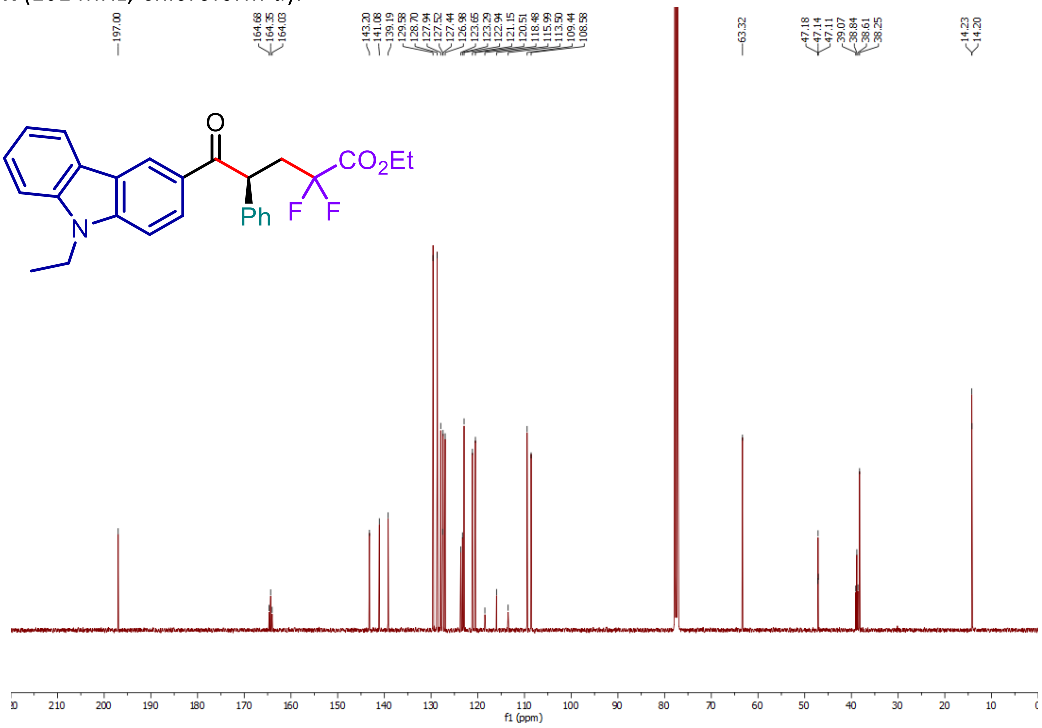

^{19}**F NMR** (376 MHz, $CDCl_3$):

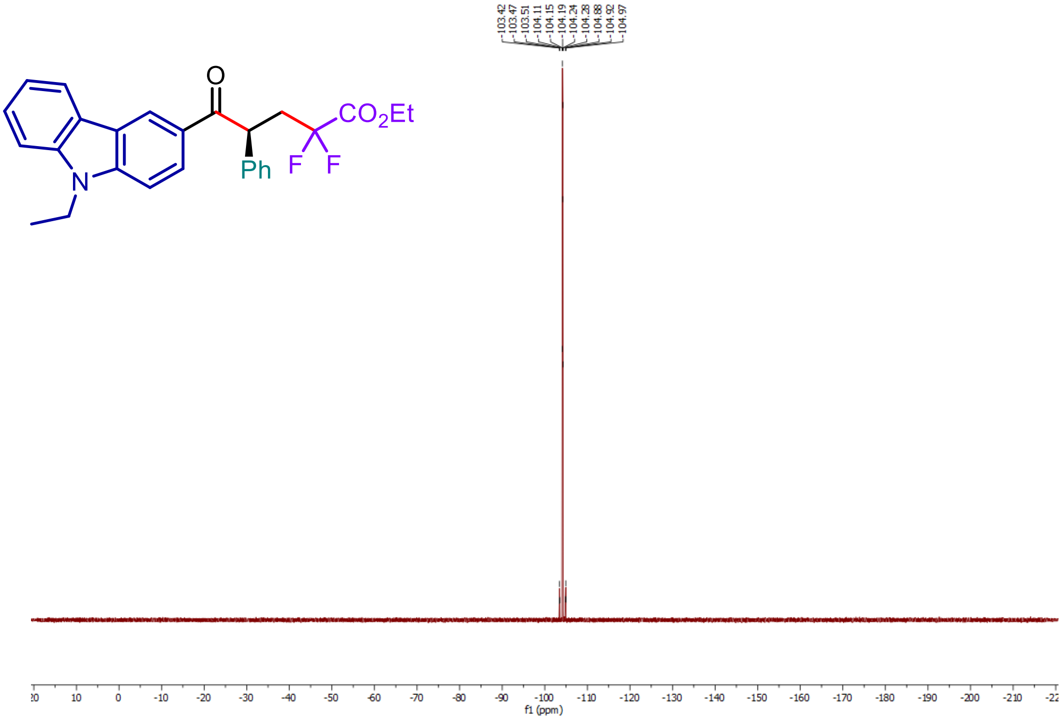

Ethyl (*S*)-2,2-difluoro-5-oxo-4-phenylhexanoate (4u)

^{1}H NMR (400 MHz, Chloroform-*d*):

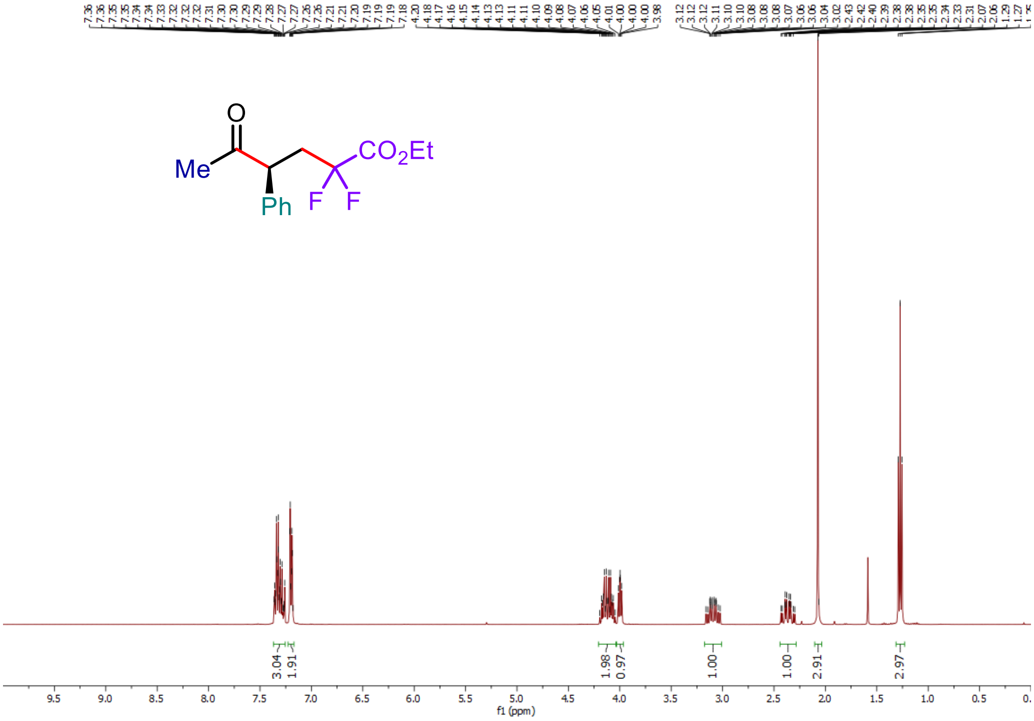

^{13}C NMR (101 MHz, Chloroform-*d*):

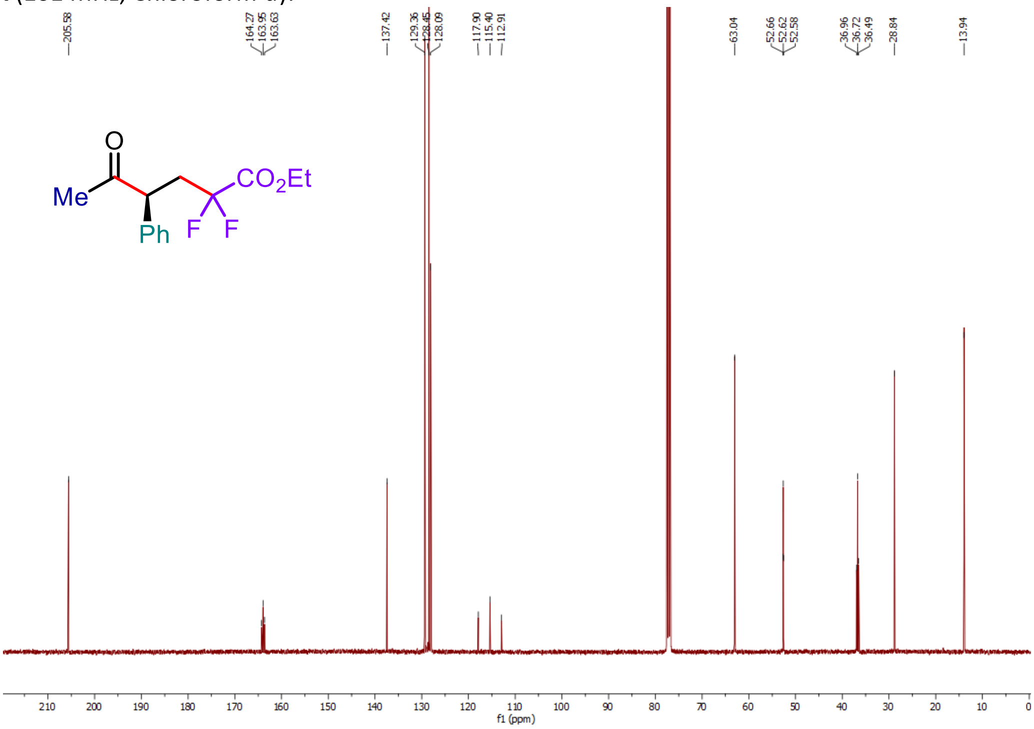

^{19}F NMR (377 MHz, Chloroform-*d*):

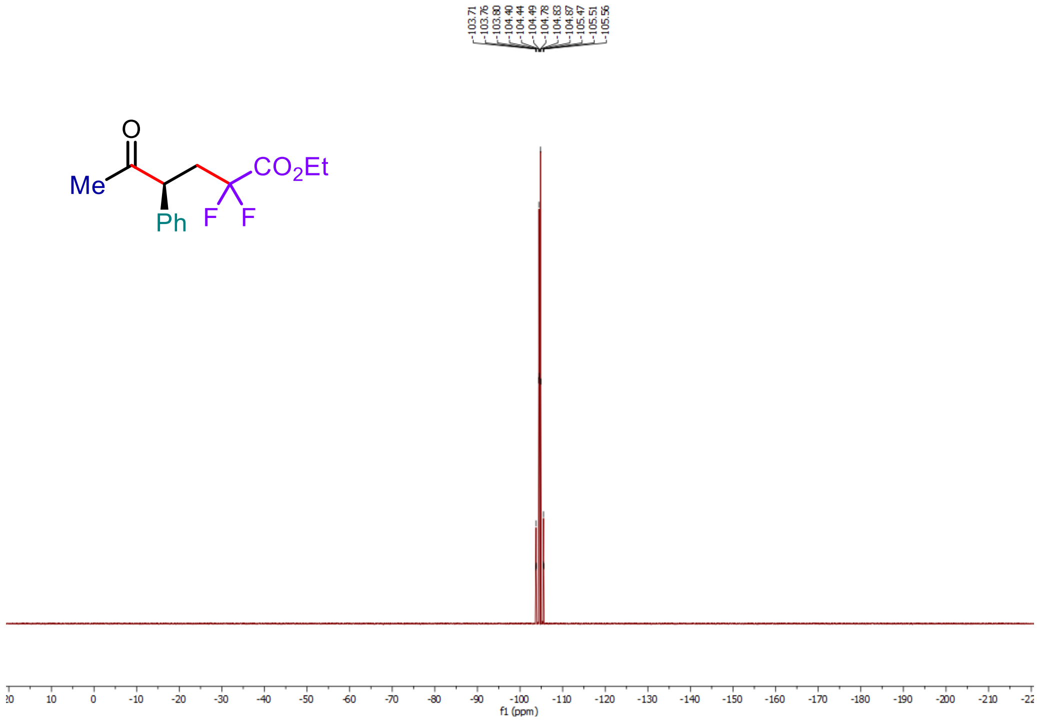

Ethyl (*S*)-2,2-difluoro-5-oxo-4-phenylheptanoate (4v)

^{1}H NMR (400 MHz, Chloroform-*d*):

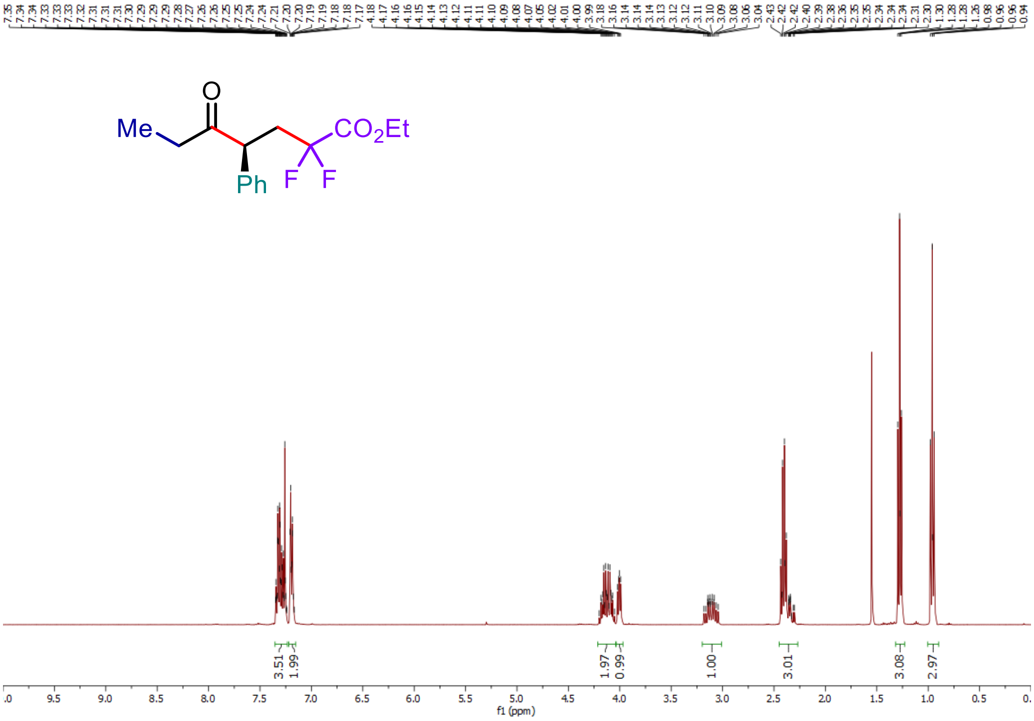

^{13}C NMR (101 MHz, Chloroform-*d*):

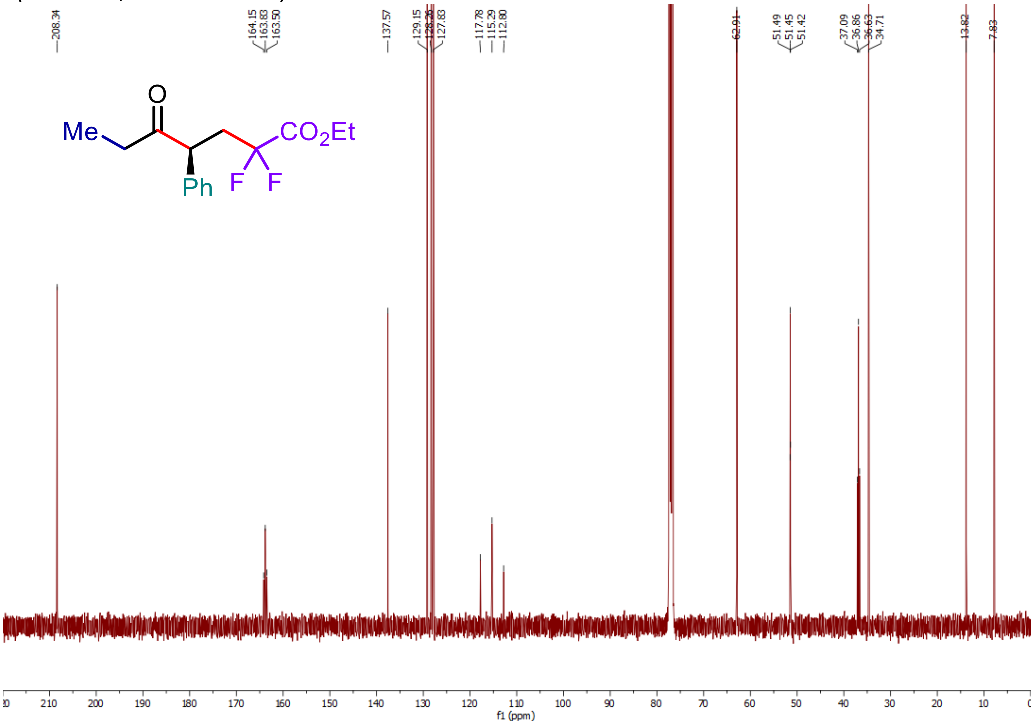

^{19}F NMR (376 MHz, Chloroform-*d*):

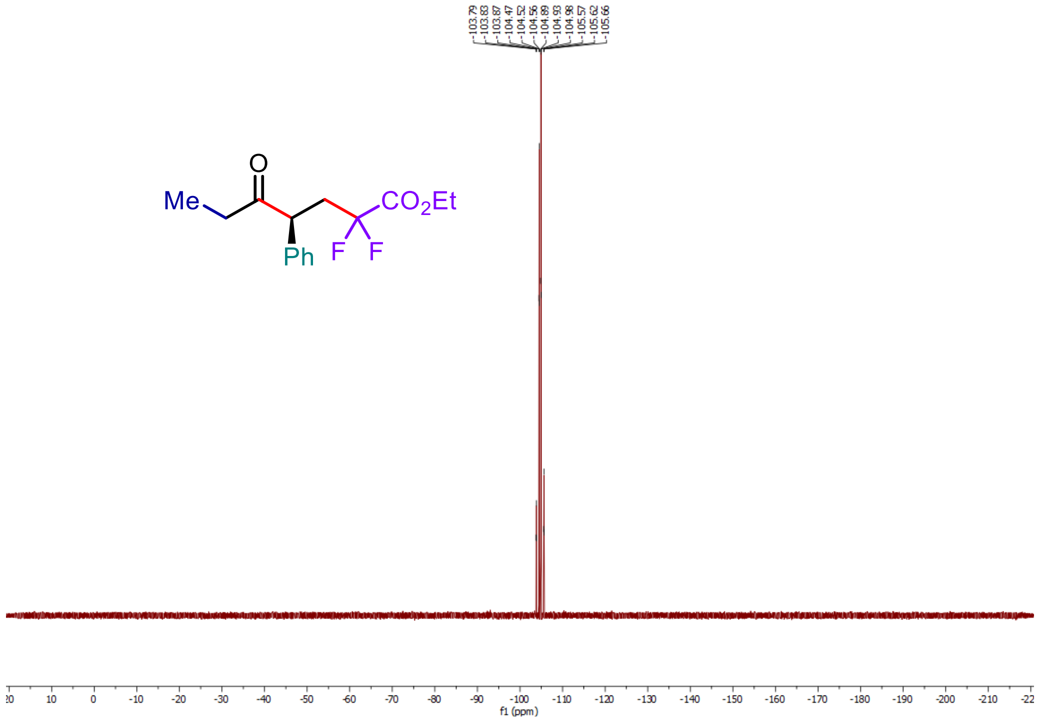

Ethyl (*S*)-2,2-difluoro-6-methyl-5-oxo-4-phenylheptanoate (4w)

^{1}H NMR (400 MHz, Chloroform-*d*):

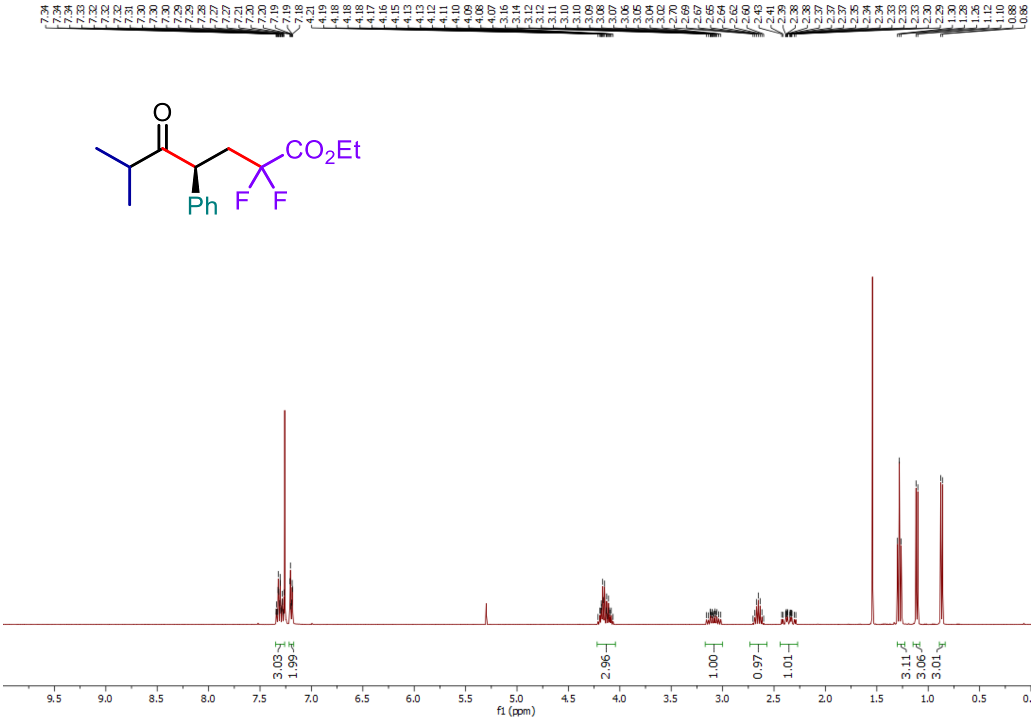

^{13}C NMR (101 MHz, Chloroform-*d*):

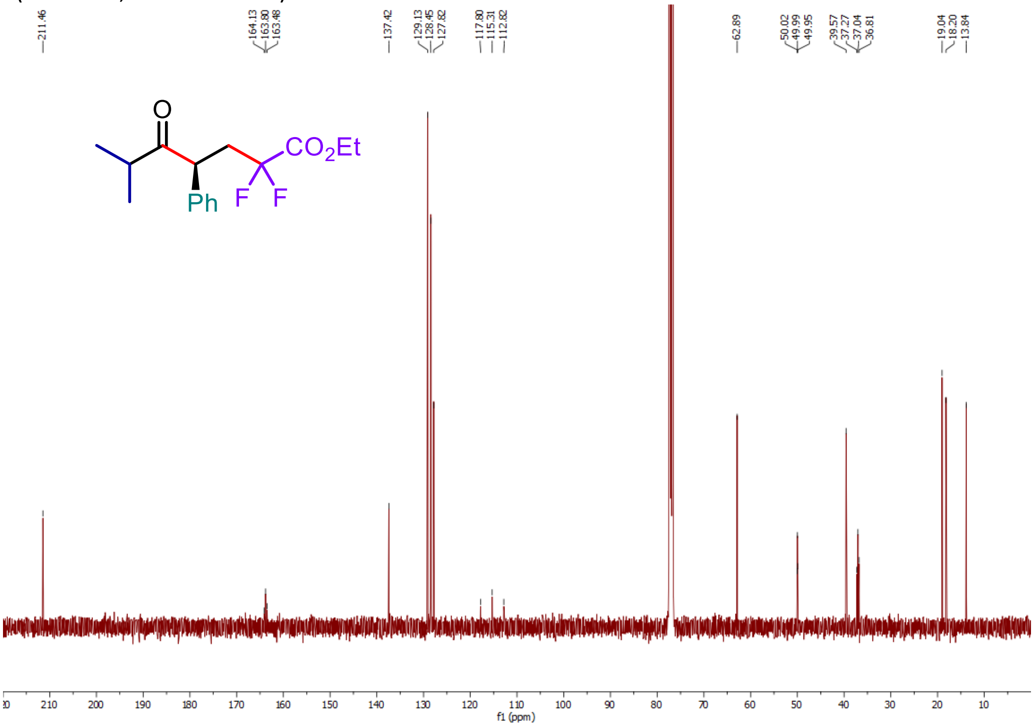

^{19}F NMR (376 MHz, Chloroform-*d*):

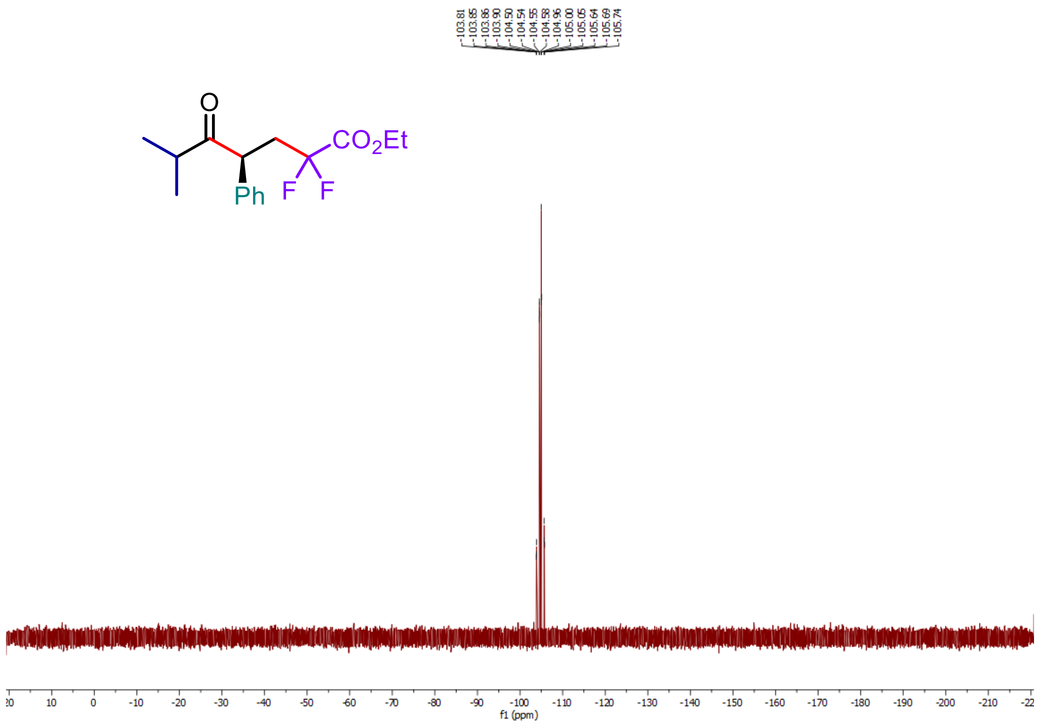

Ethyl (*S*)-5-cyclopropyl-2,2-difluoro-5-oxo-4-phenylpentanoate (4x)

^{1}H NMR (400 MHz, Chloroform-*d*):

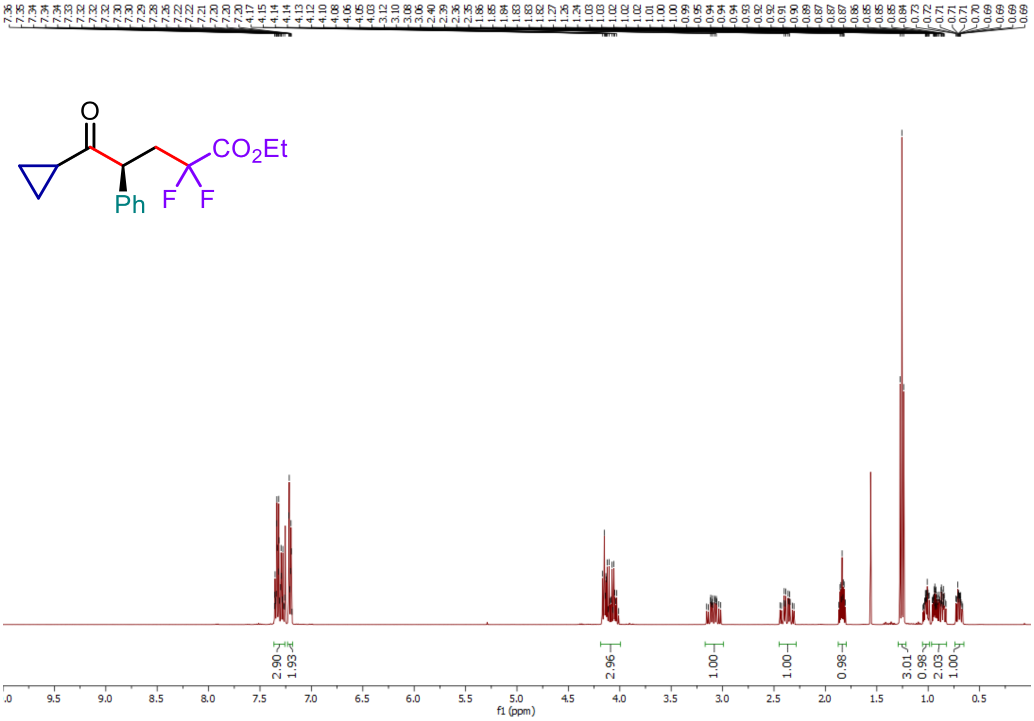

^{13}C NMR (101 MHz, Chloroform-*d*):

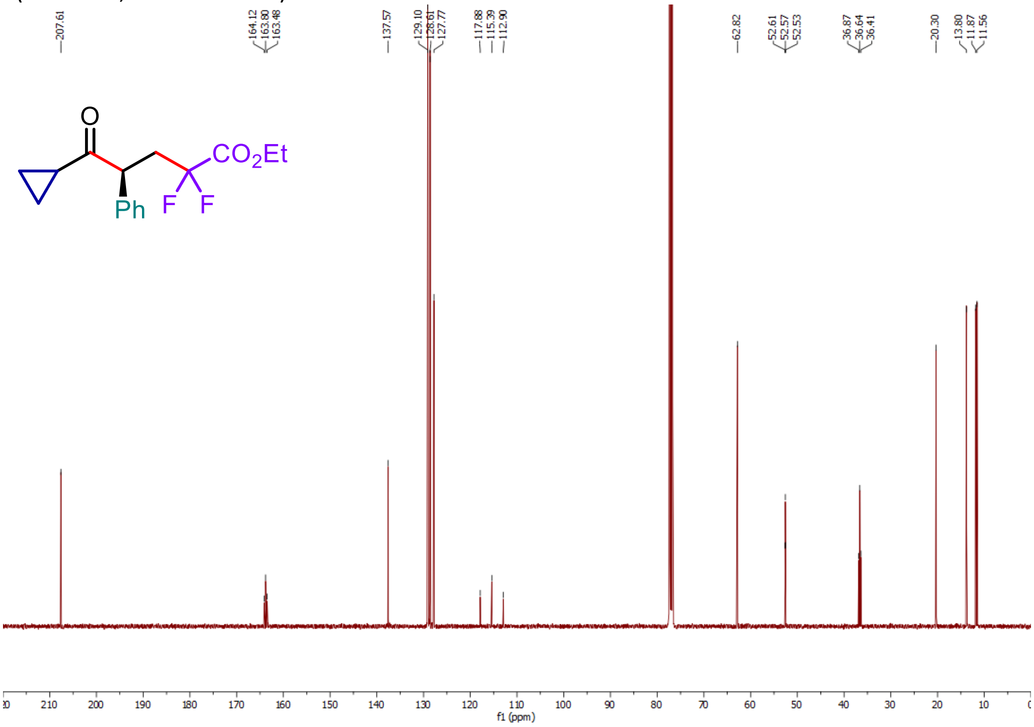

^{19}F NMR (376 MHz, Chloroform-*d*):

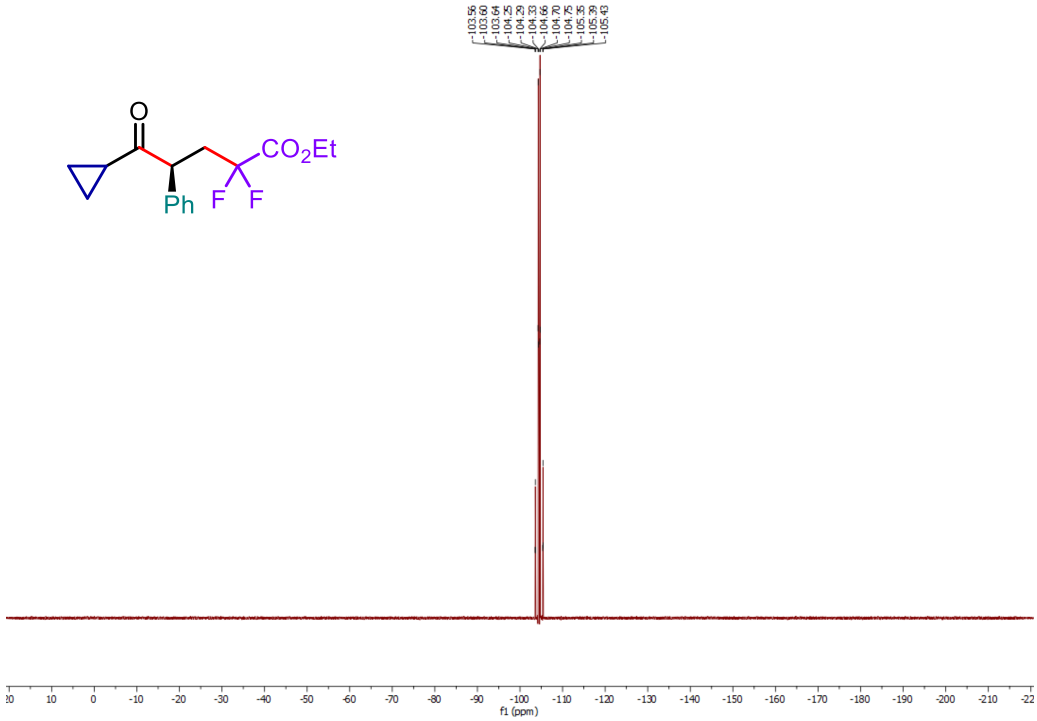

Ethyl (4*S*)-5-(2,2-dimethylcyclopropyl)-2,2-difluoro-5-oxo-4-phenylpentanoate (4y)

^{1}H NMR (400 MHz, Chloroform-*d*):

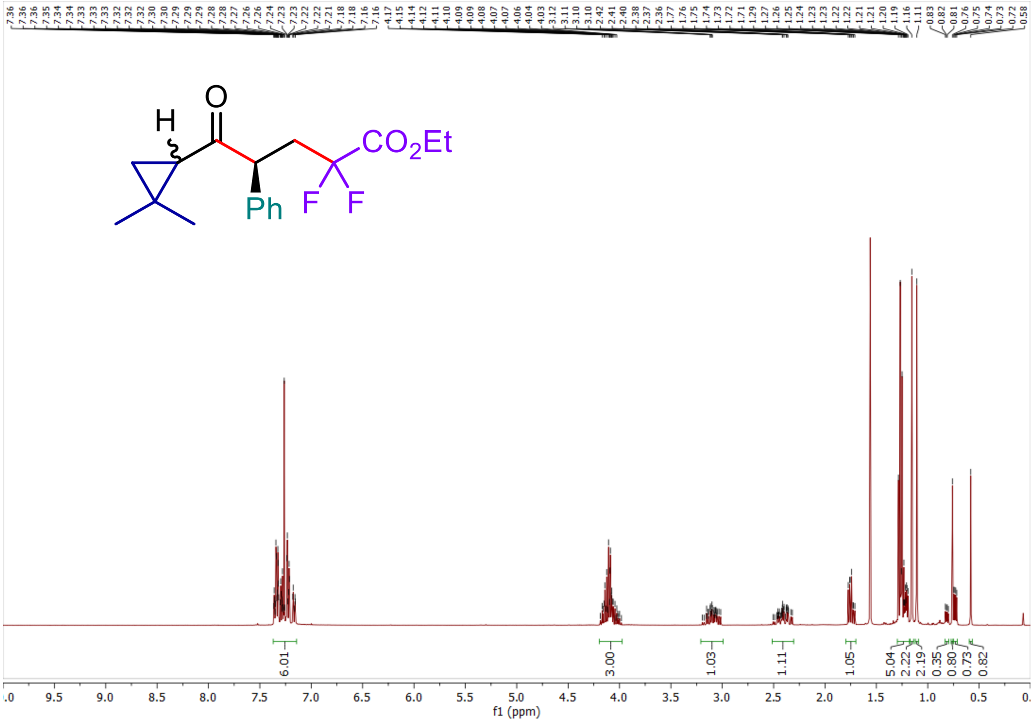

^{13}C NMR (101 MHz, Chloroform-*d*):

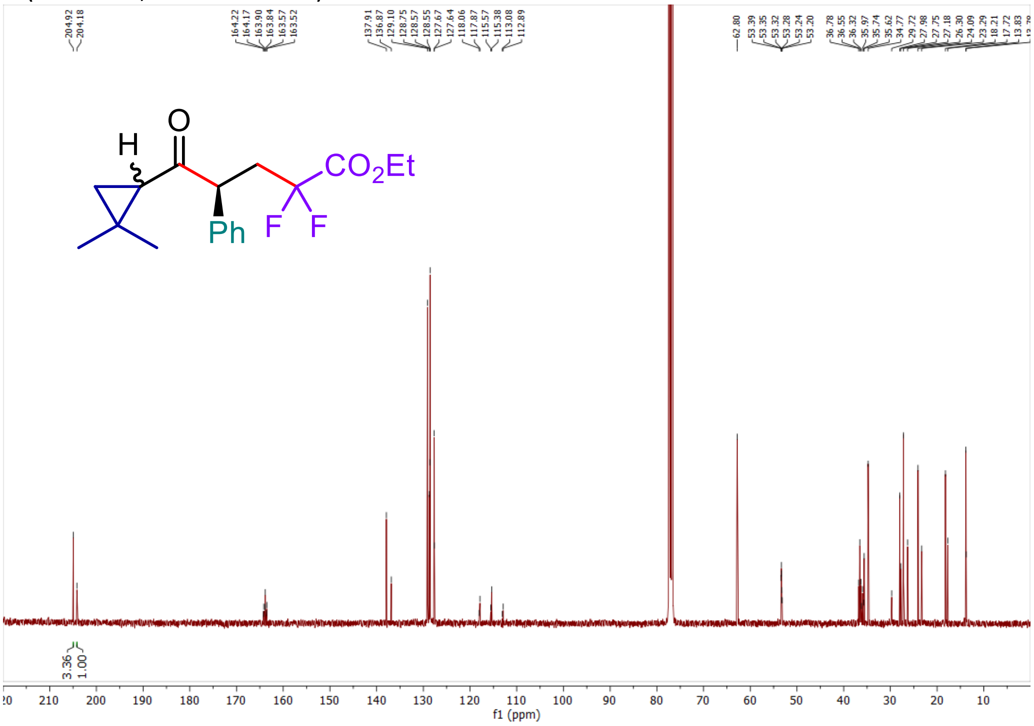

19F NMR (376 MHz, Chloroform-*d*):

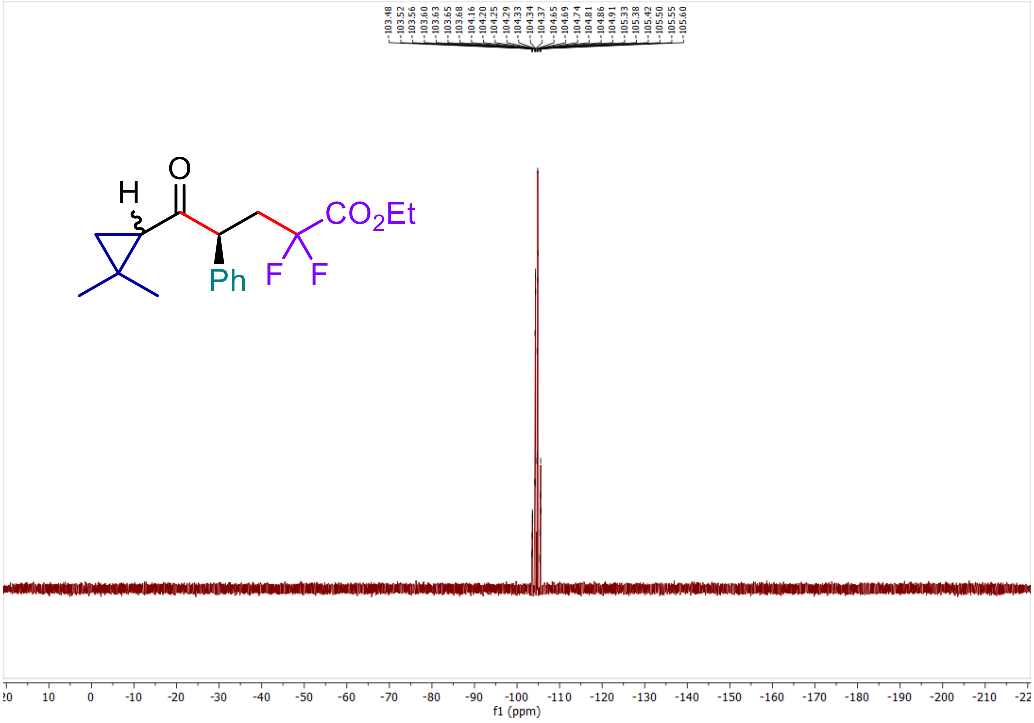

Ethyl (*S*)-2,2-difluoro-4-(4-fluorophenyl)-5-oxo-5-phenylpentanoate (6a)

^{1}H NMR (400 MHz, Chloroform-*d*):

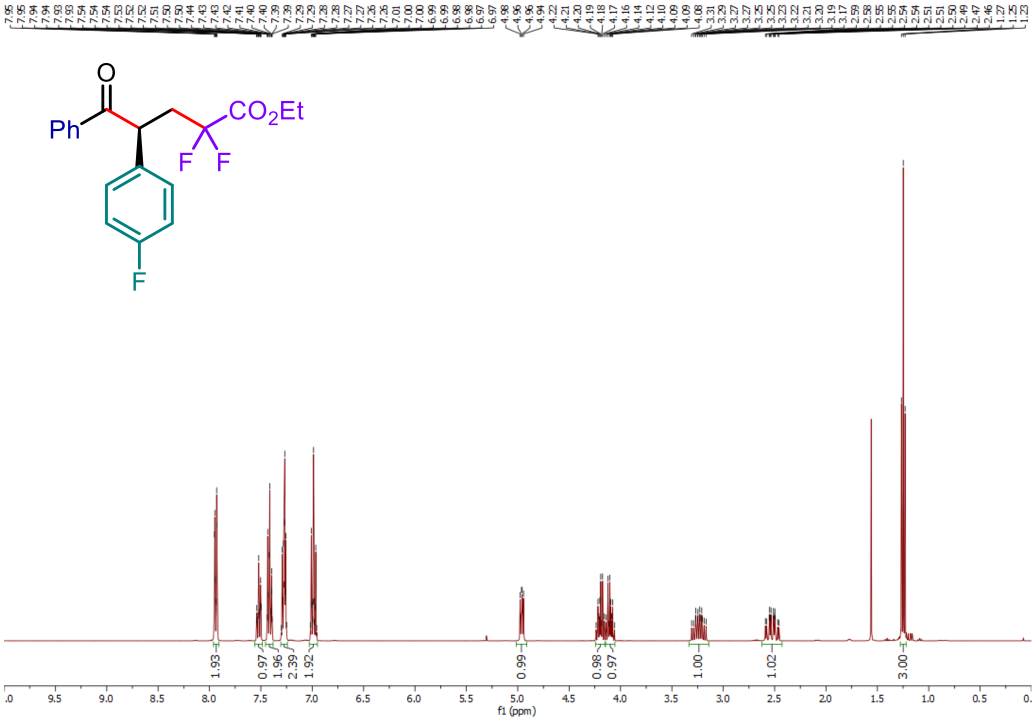

^{13}C NMR (101 MHz, Chloroform-*d*):

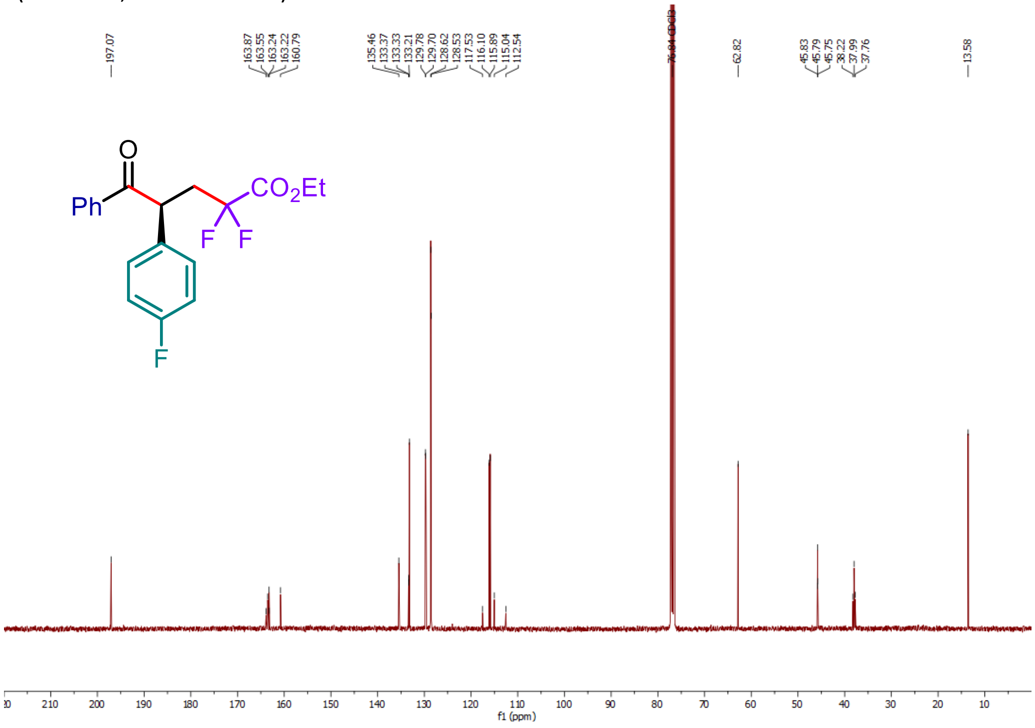

^{19}F NMR (376 MHz, Chloroform-*d*):

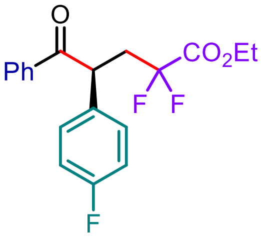

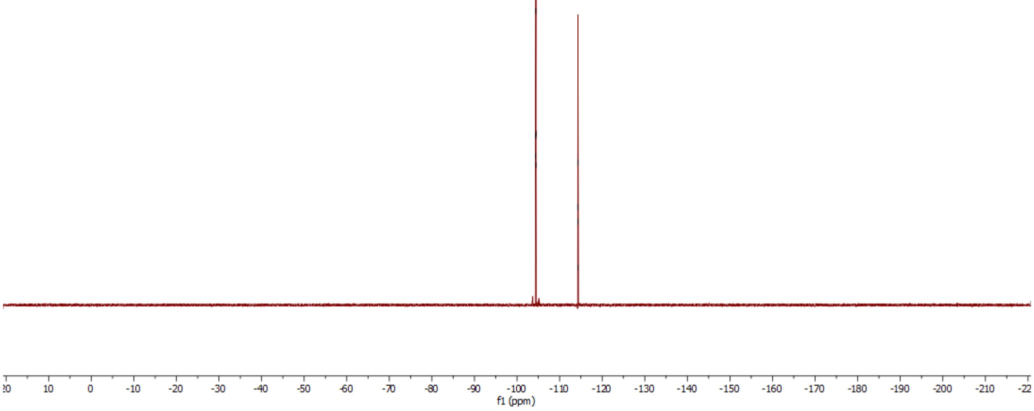

Ethyl (*S*)-2,2-difluoro-4-(4-methoxyphenyl)-5-oxo-5-phenylpentanoate (6b)

^{1}H NMR (400 MHz, Chloroform-*d*):

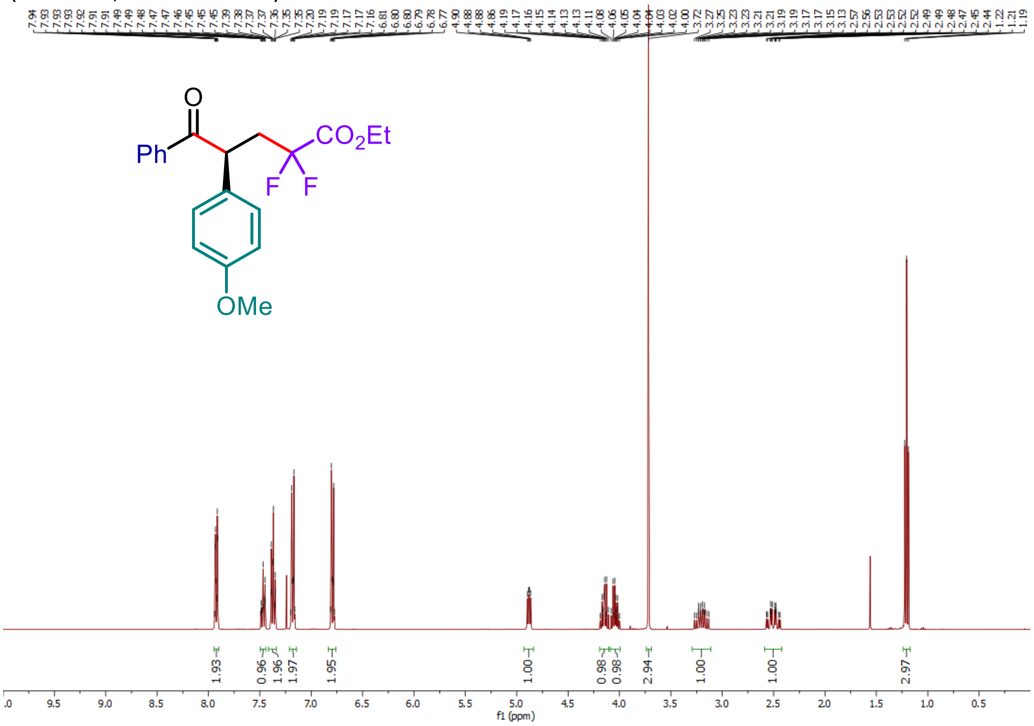

^{13}C NMR (101 MHz, Chloroform-*d*):

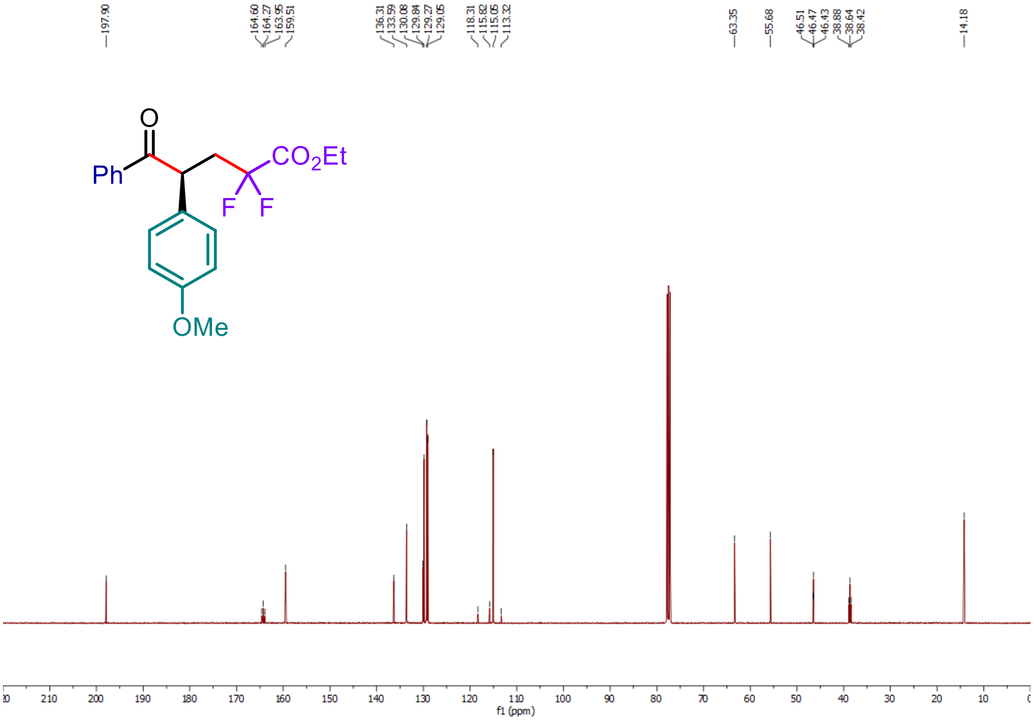

^{19}F NMR (376 MHz, Chloroform-*d*):

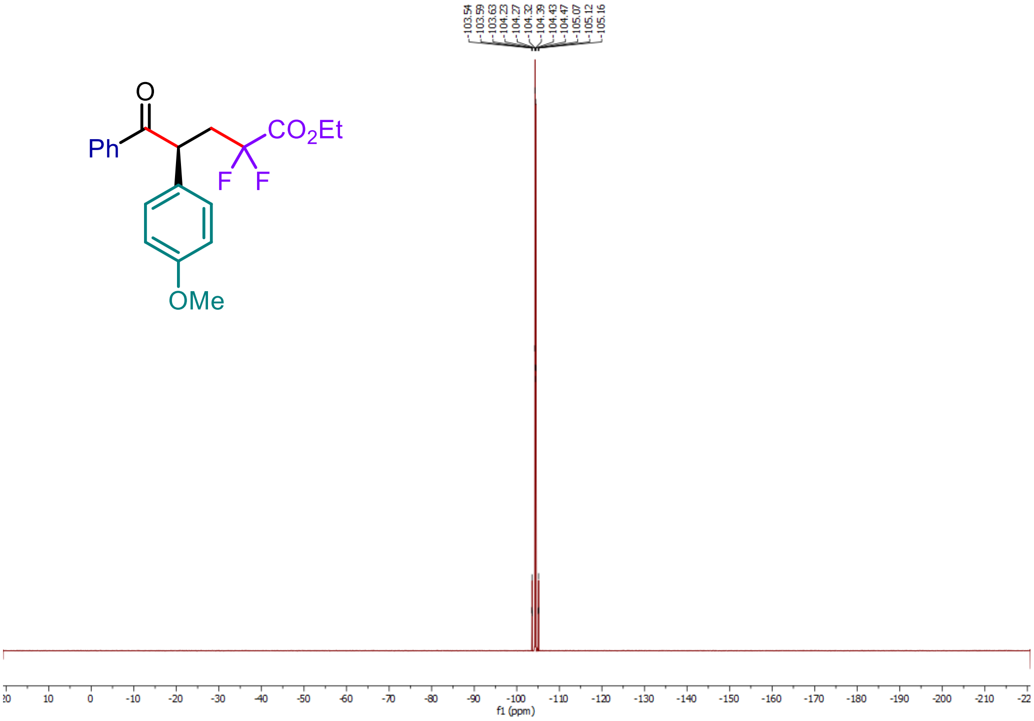

Ethyl (*S*)-4-(4-cyanophenyl)-2,2-difluoro-5-oxo-5-phenylpentanoate (6c)

^{1}H NMR (400 MHz, Chloroform-*d*):

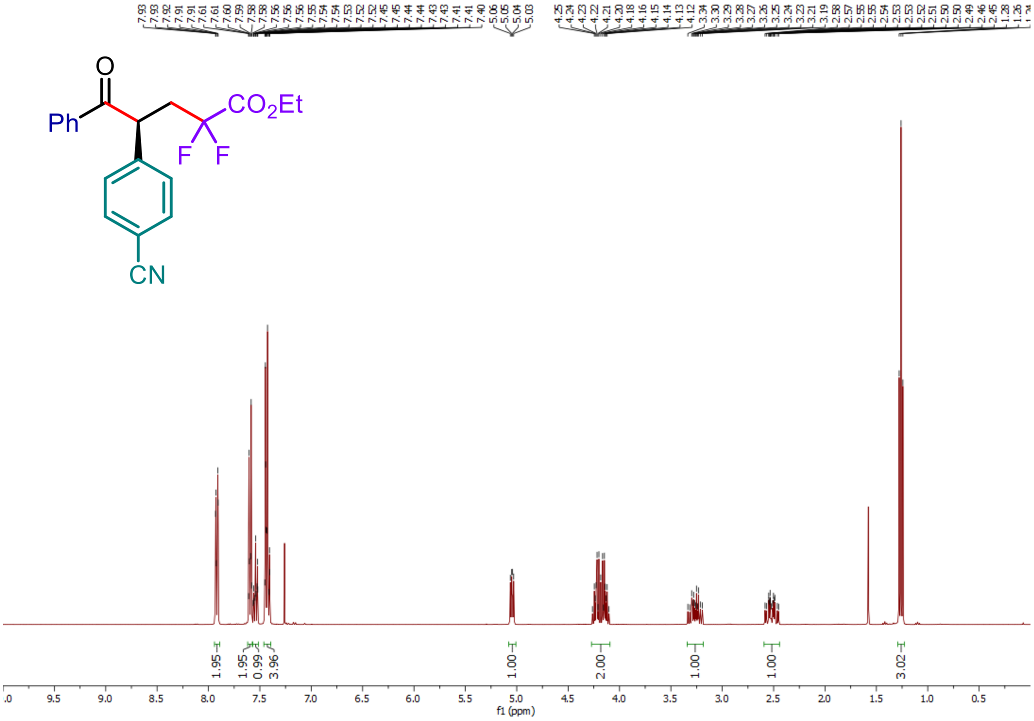

^{13}C NMR (101 MHz, Chloroform-*d*):

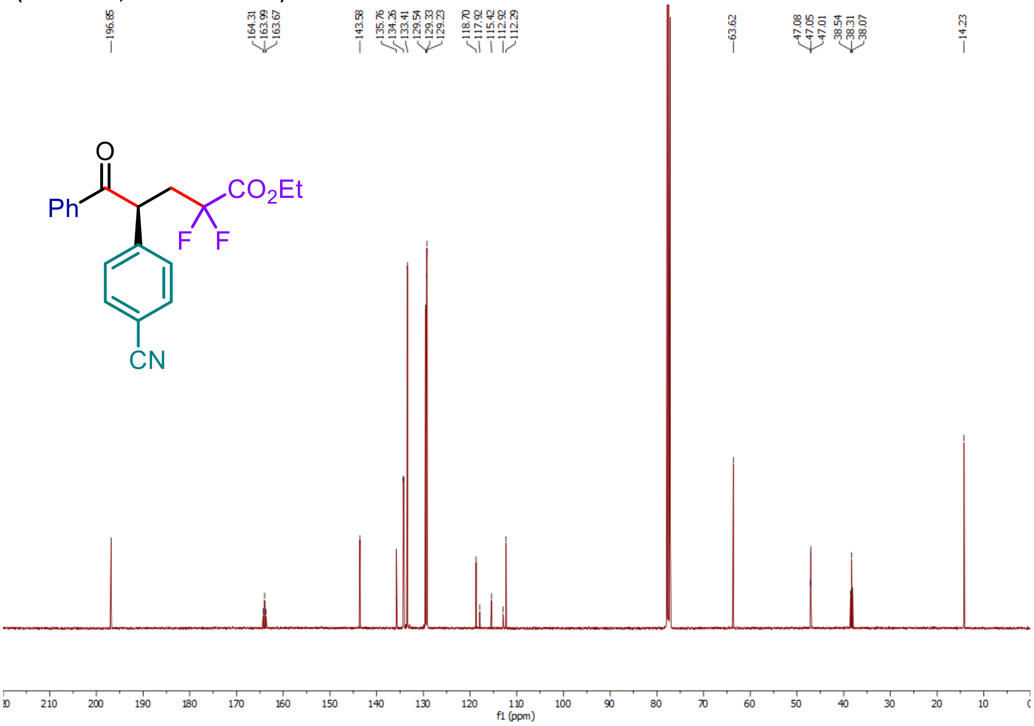

^{19}F NMR (376 MHz, Chloroform-*d*):

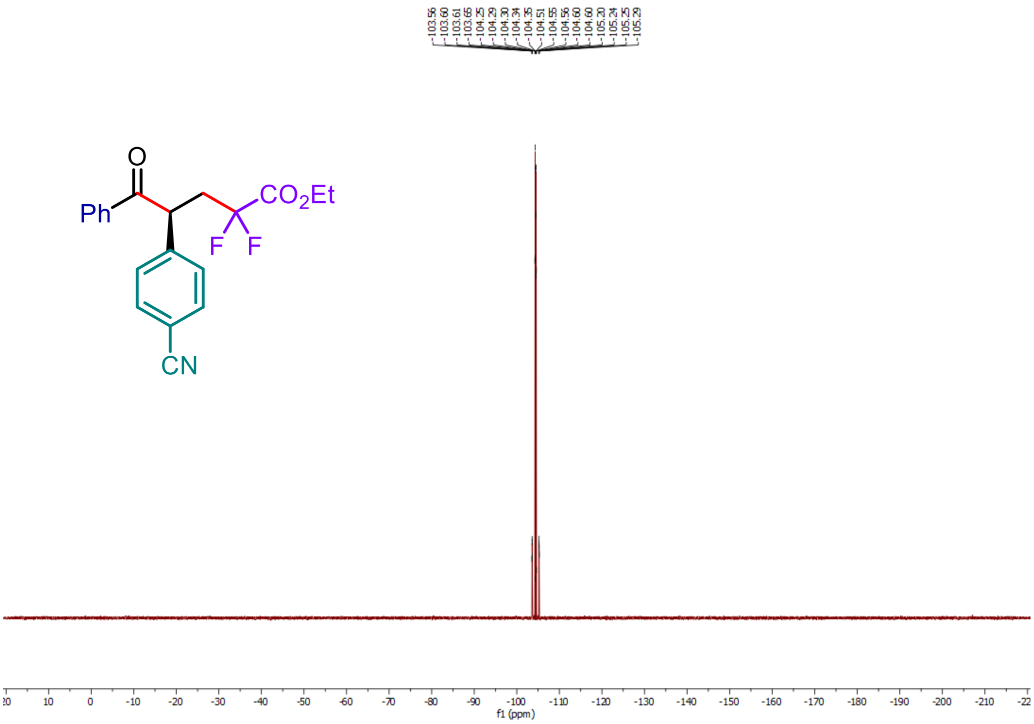

Ethyl (*S*)-2,2-difluoro-4-(4-nitrophenyl)-5-oxo-5-phenylpentanoate (6d)

^{1}H NMR (400 MHz, Chloroform-*d*):

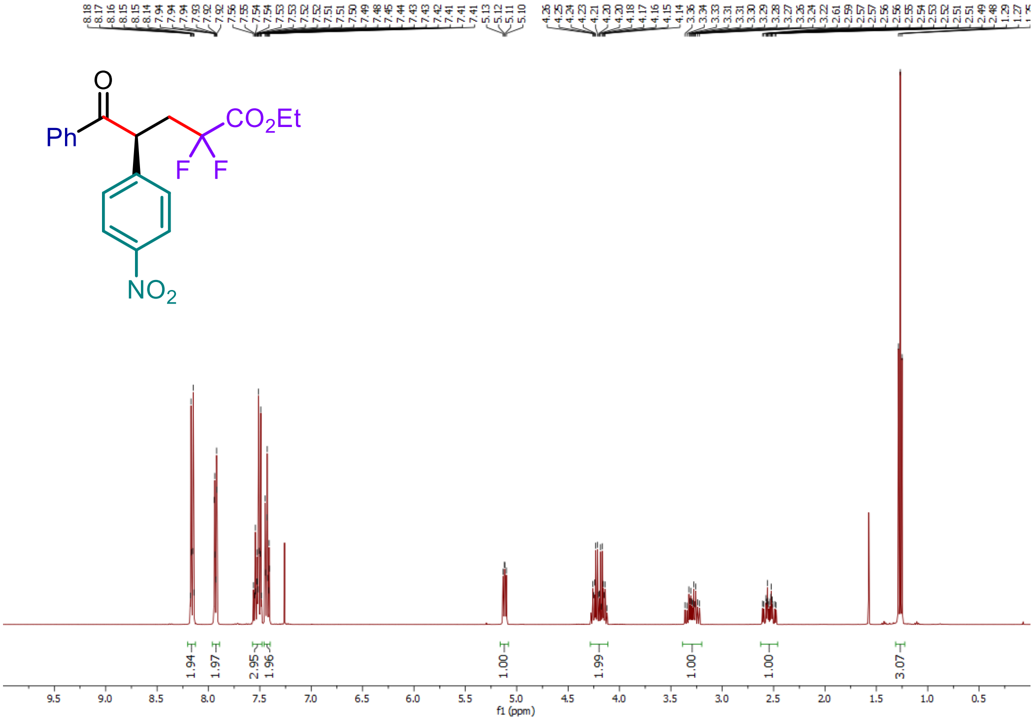

^{13}C NMR (101 MHz, Chloroform-*d*):

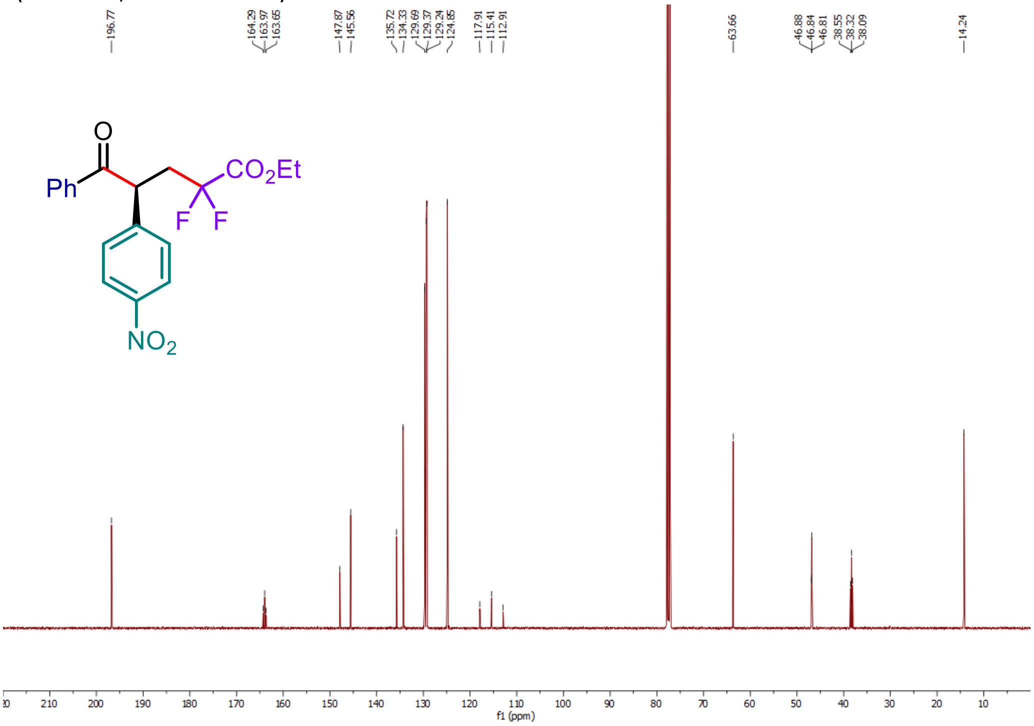

^{19}F NMR (376 MHz, Chloroform-*d*):

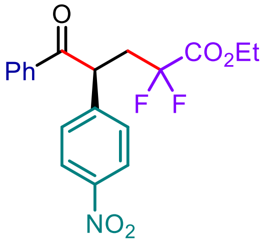

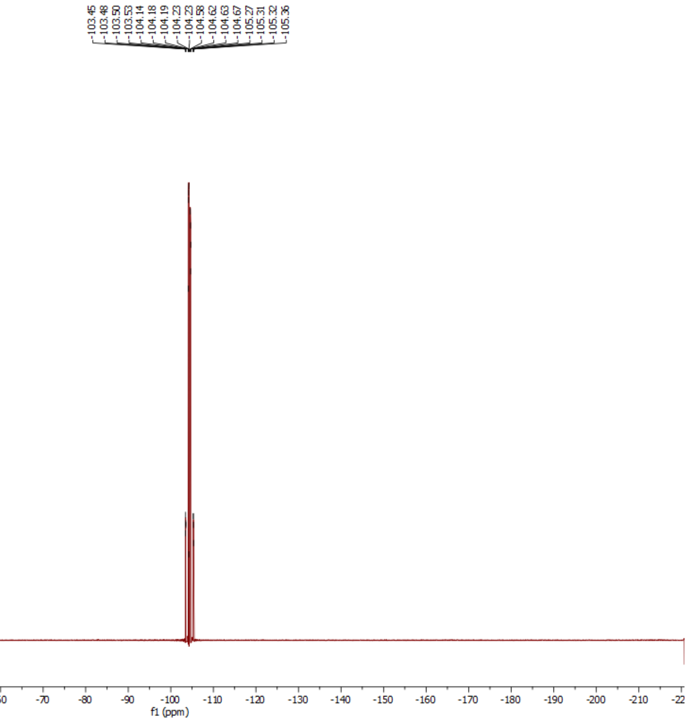

Ethyl (*S*)-2,2-difluoro-5-oxo-5-phenyl-4-(m-tolyl)pentanoate (6e)

^{1}H NMR (400 MHz, Chloroform-*d*):

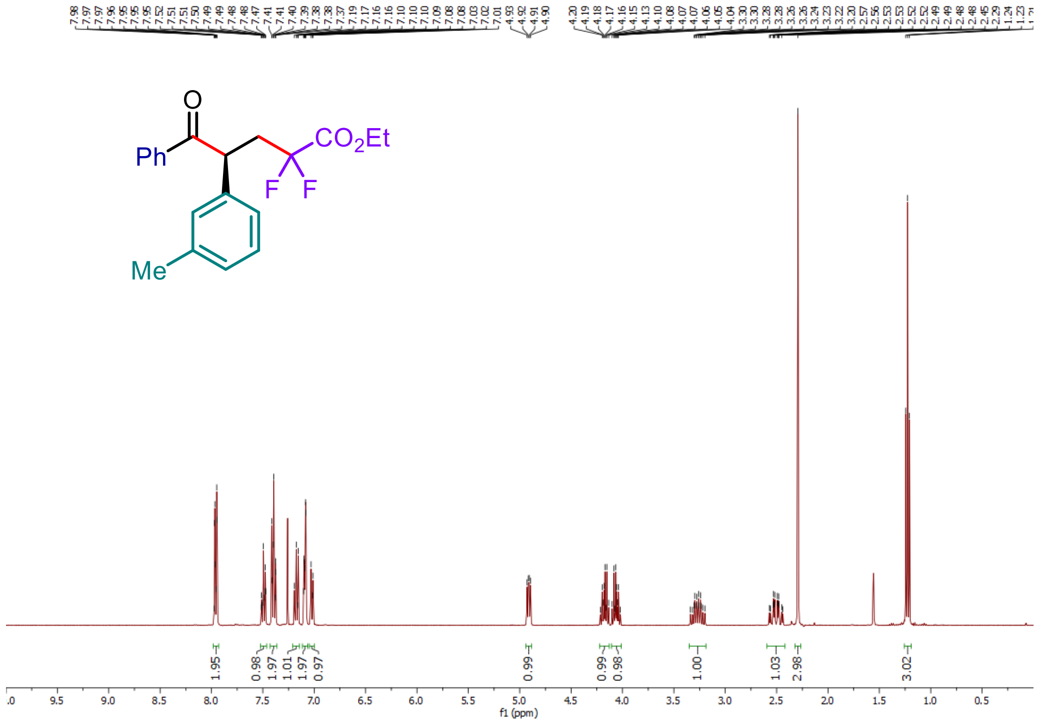

^{13}C NMR (101 MHz, Chloroform-*d*):

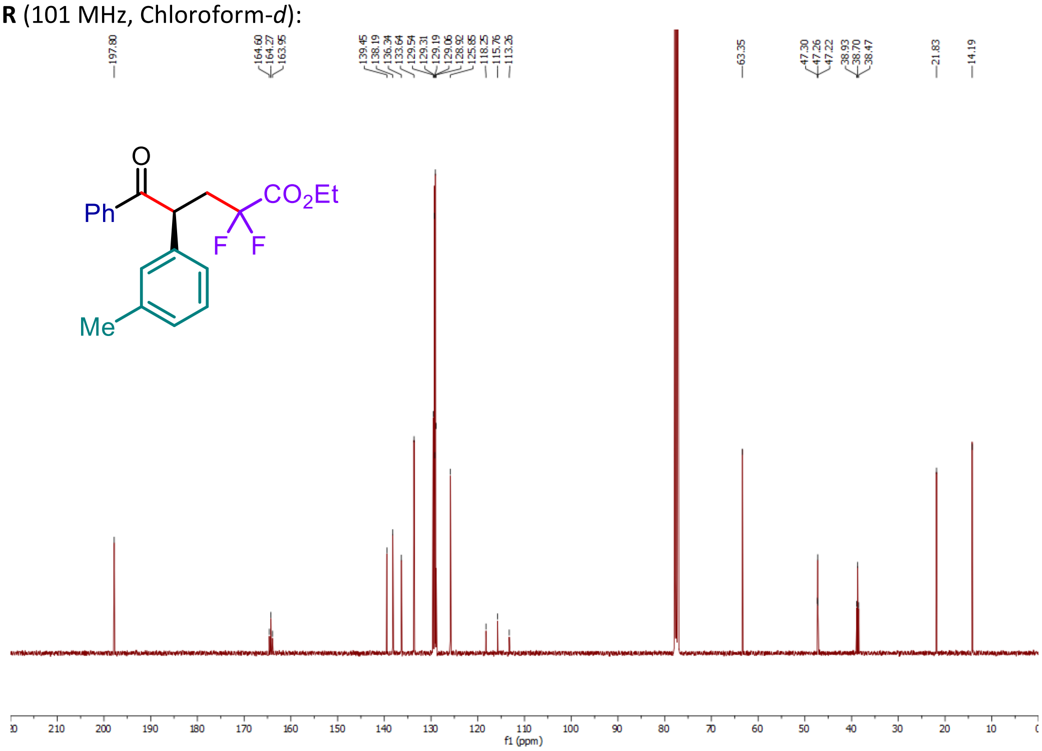

^{19}F NMR (376 MHz, Chloroform-*d*):

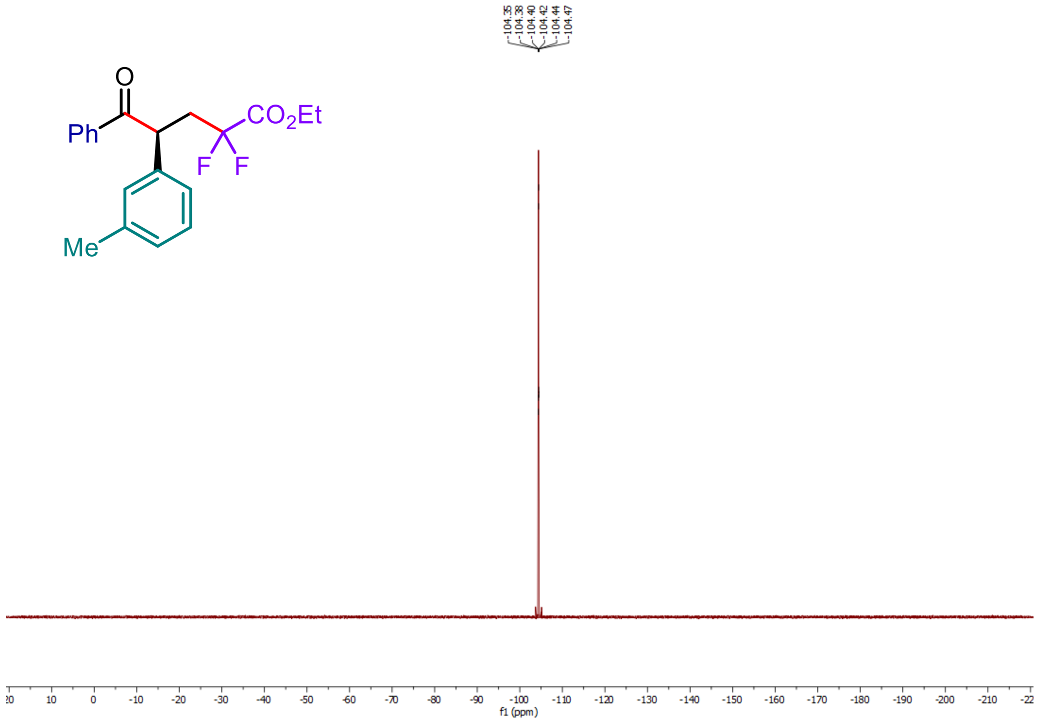

Ethyl (*S*)-2,2-difluoro-5-oxo-5-phenyl-4-(*o*-tolyl)pentanoate (6f)

^{1}H NMR (400 MHz, Chloroform-*d*):

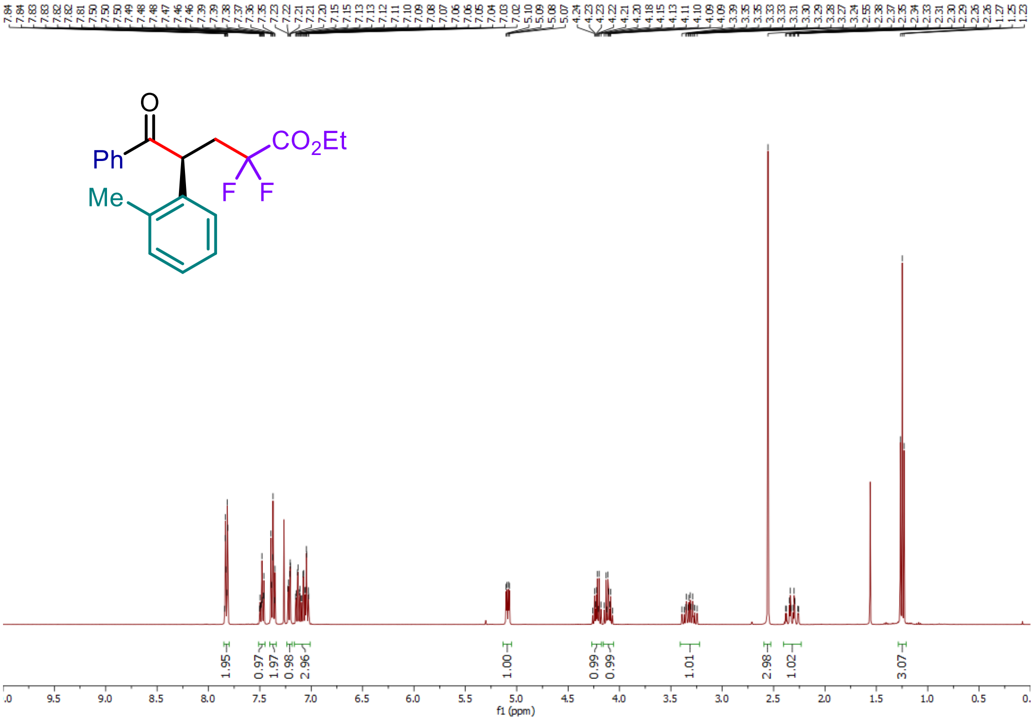

^{13}C NMR (101 MHz, Chloroform-*d*):

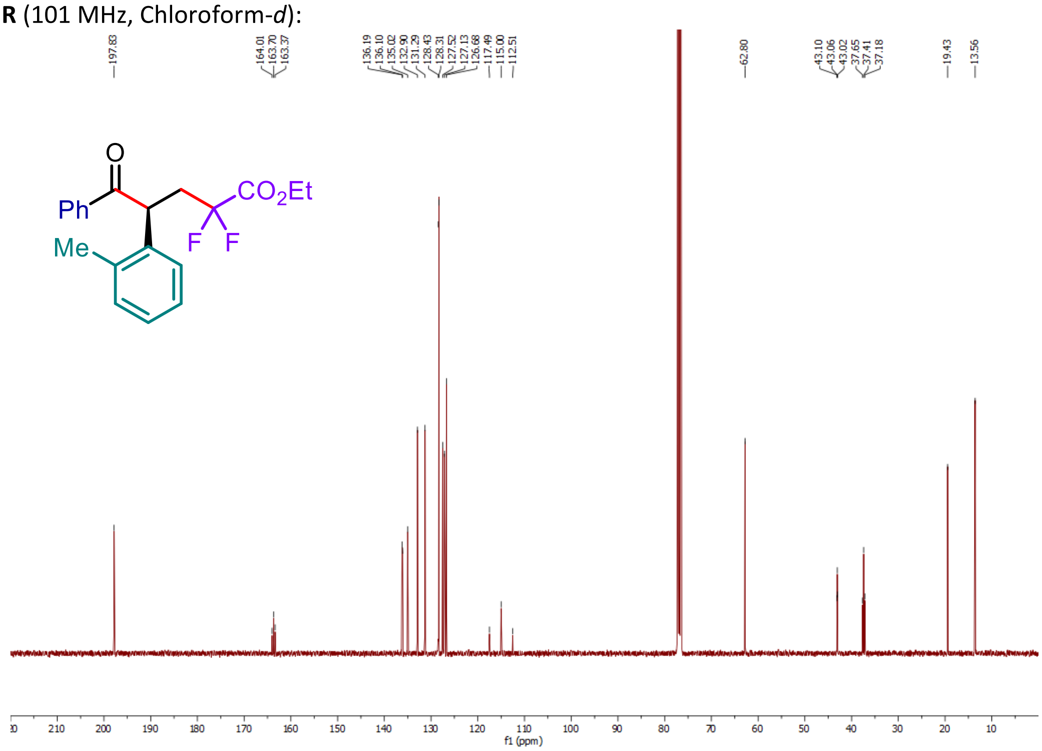

^{19}F NMR (376 MHz, Chloroform-*d*):

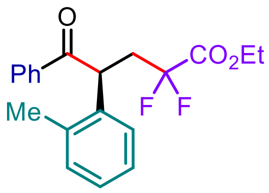

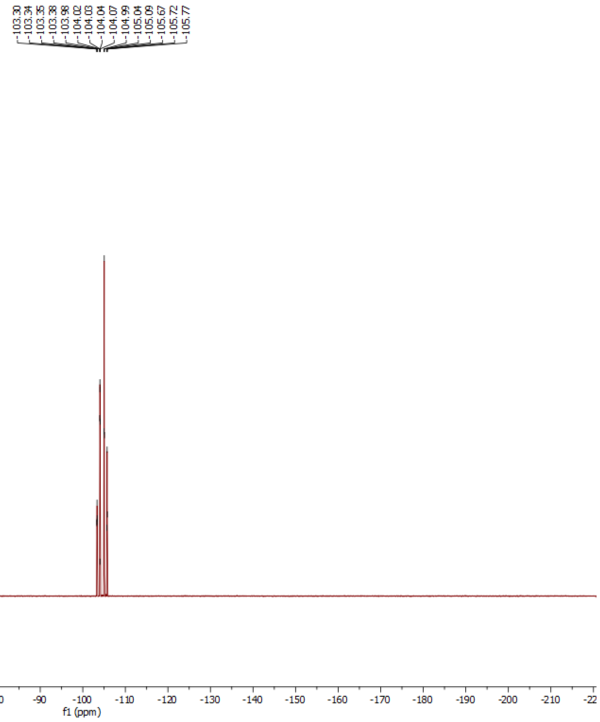

Ethyl (*S*)-2,2-difluoro-4-(2-fluorophenyl)-5-oxo-5-phenylpentanoate (6g)

^{1}H NMR (400 MHz, Chloroform-*d*):

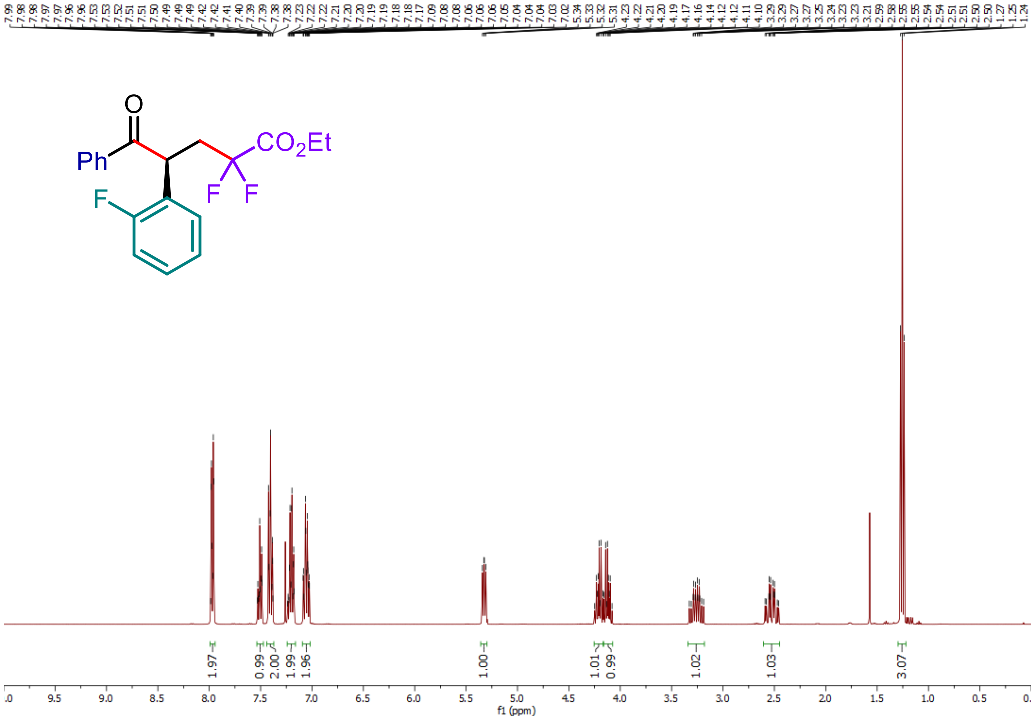

^{13}C NMR (101 MHz, Chloroform-*d*)

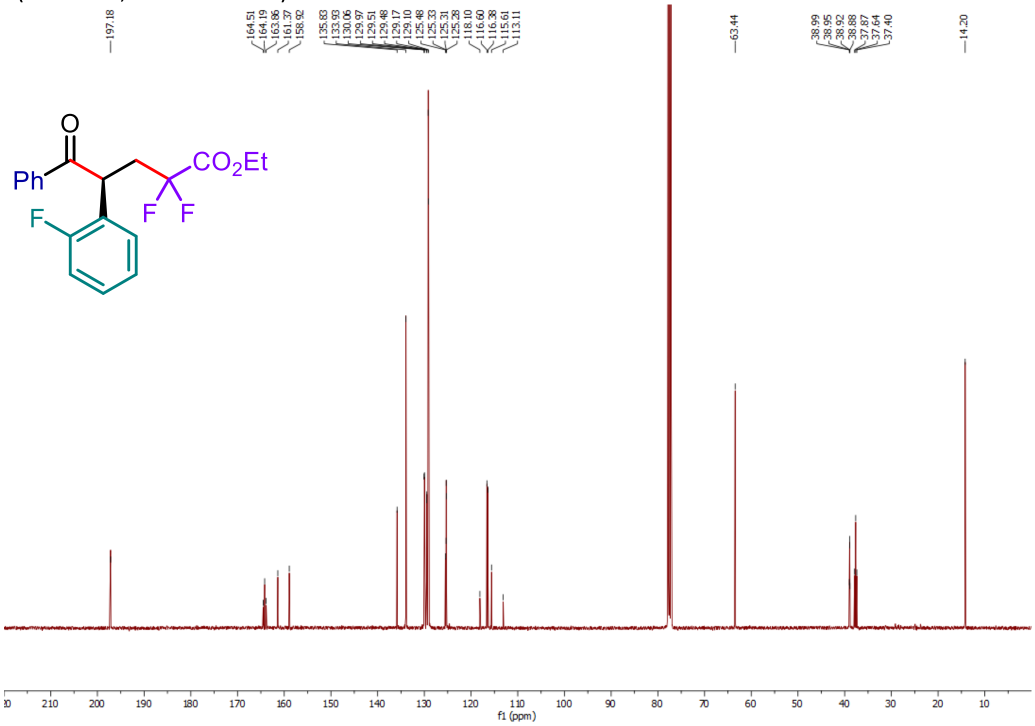

^{19}F NMR (376 MHz, Chloroform-*d*):

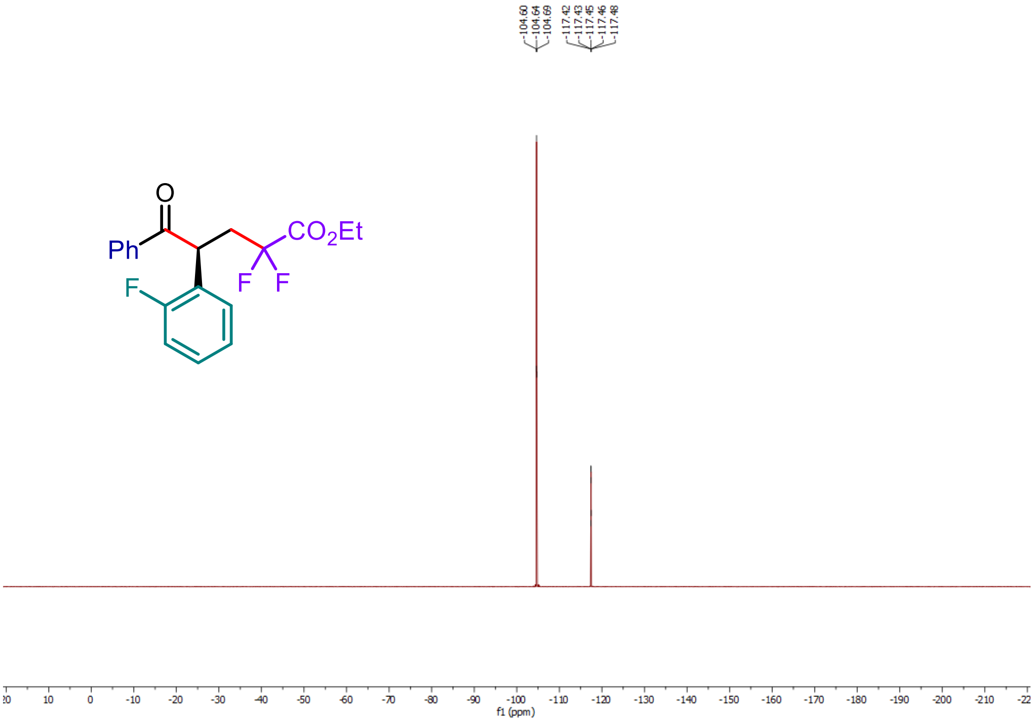

Ethyl (*S*)-2,2-difluoro-4-(naphthalen-2-yl)-5-oxo-5-phenylpentanoate (6h)

^{1}H NMR (400 MHz, Chloroform-*d*):

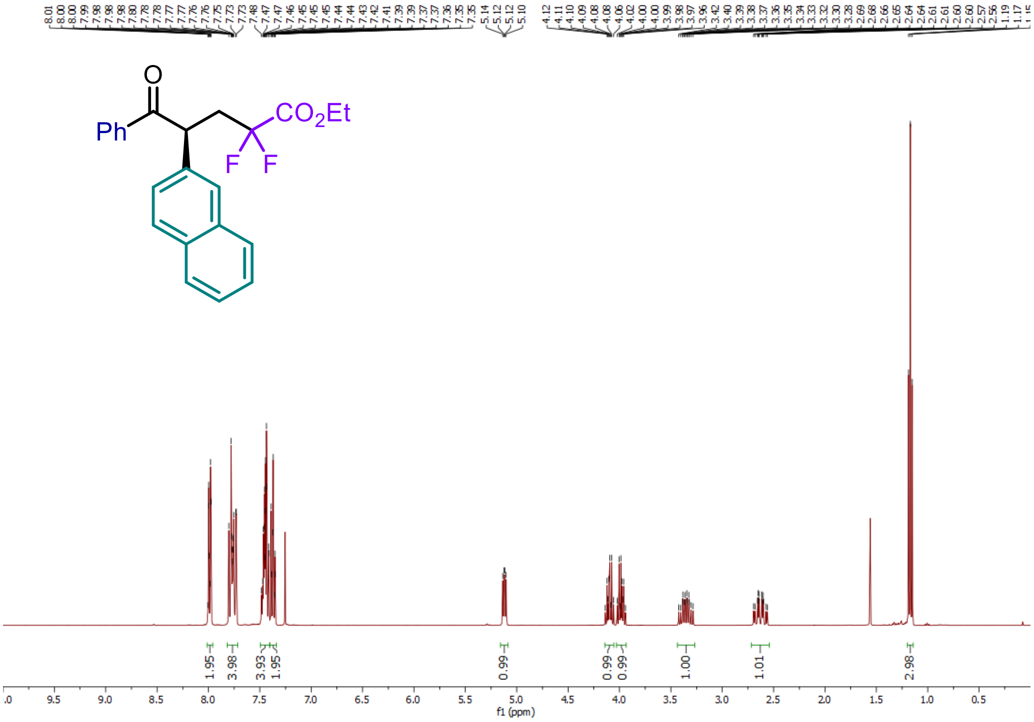

^{13}C NMR (101 MHz, Chloroform-*d*):

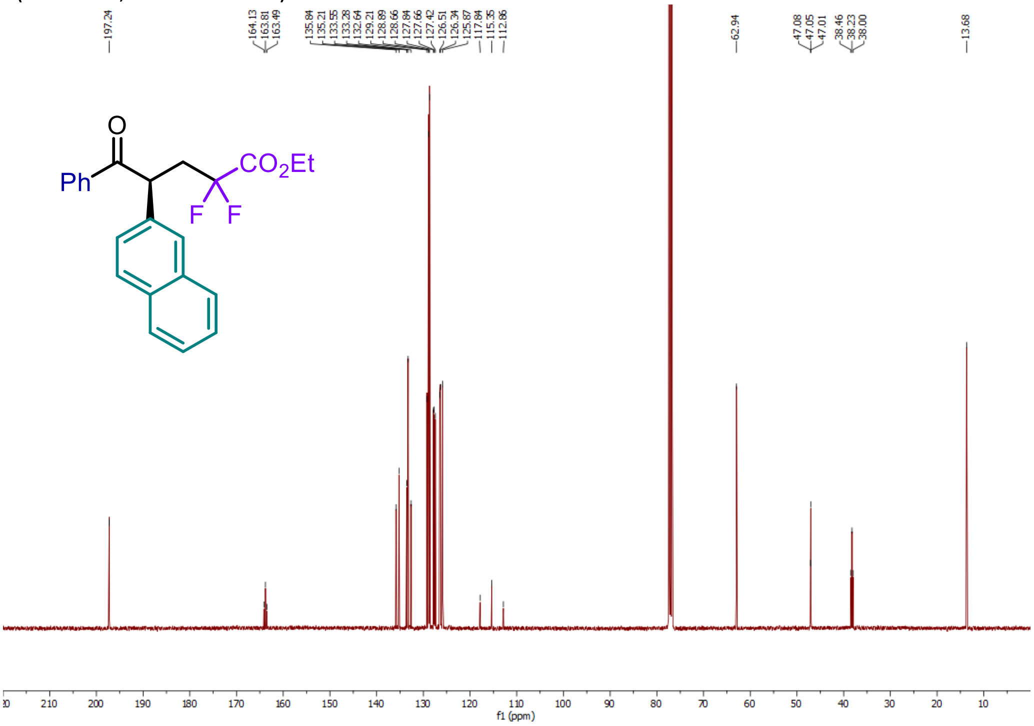

^{19}F NMR (376 MHz, Chloroform-*d*):

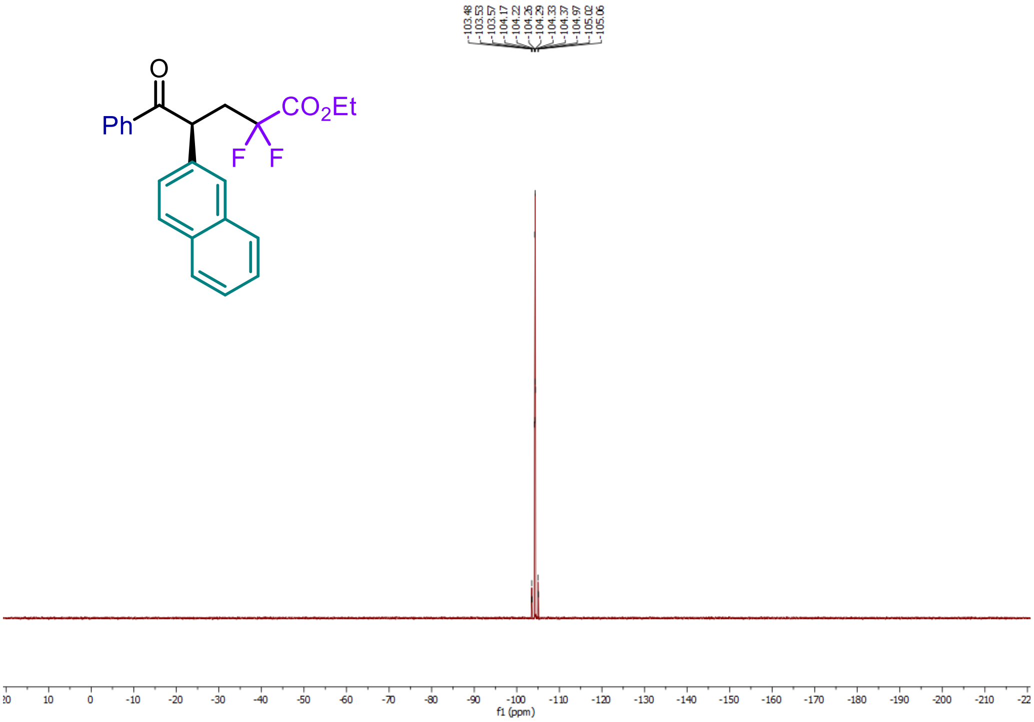

Ethyl (*S*)-2,2-difluoro-4-(2-ferrocenyl)-5-oxo-5-phenylpentanoate (6i)

^{1}H NMR (400 MHz, Chloroform-*d*):

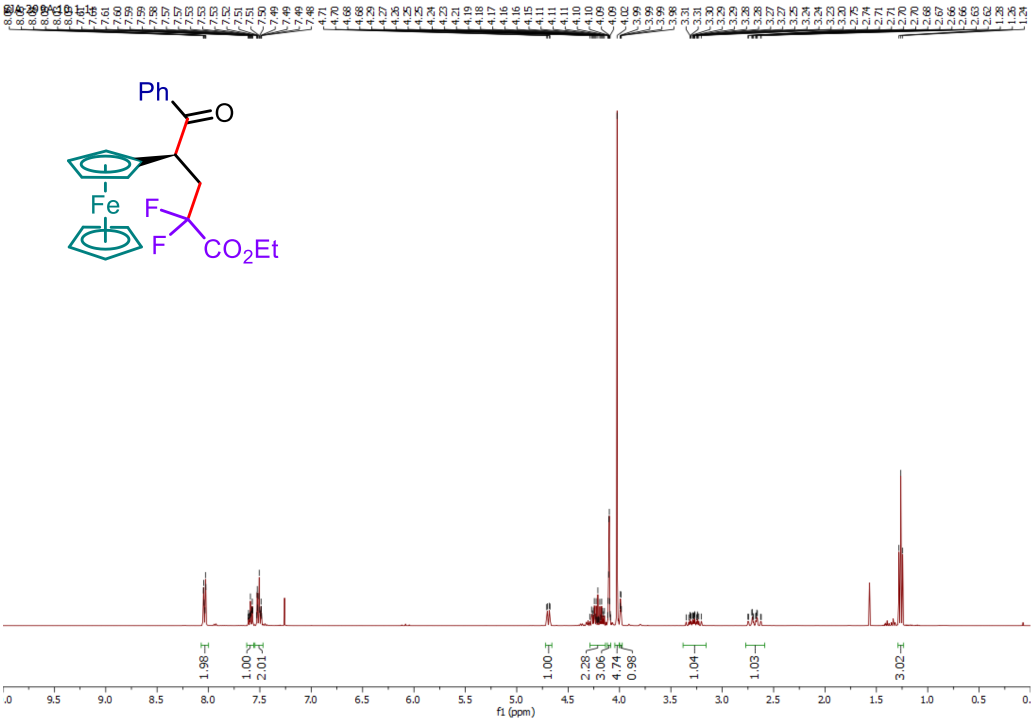

^{13}C NMR (101 MHz, Chloroform-*d*):

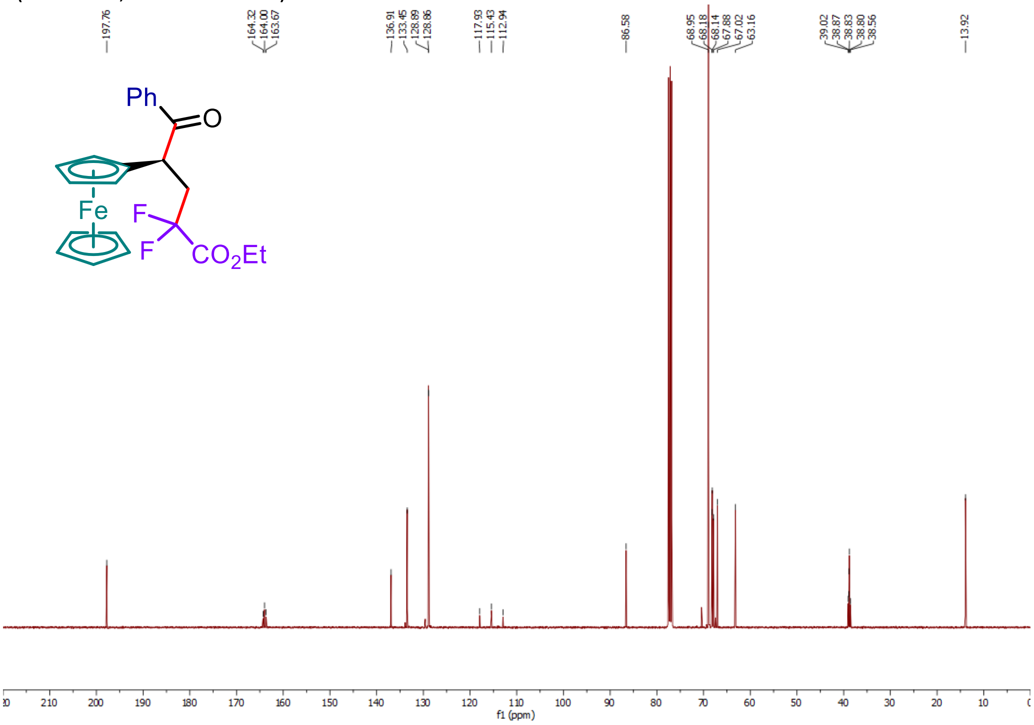

^{19}**F NMR** (376 MHz, Chloroform-*d*):

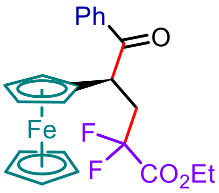

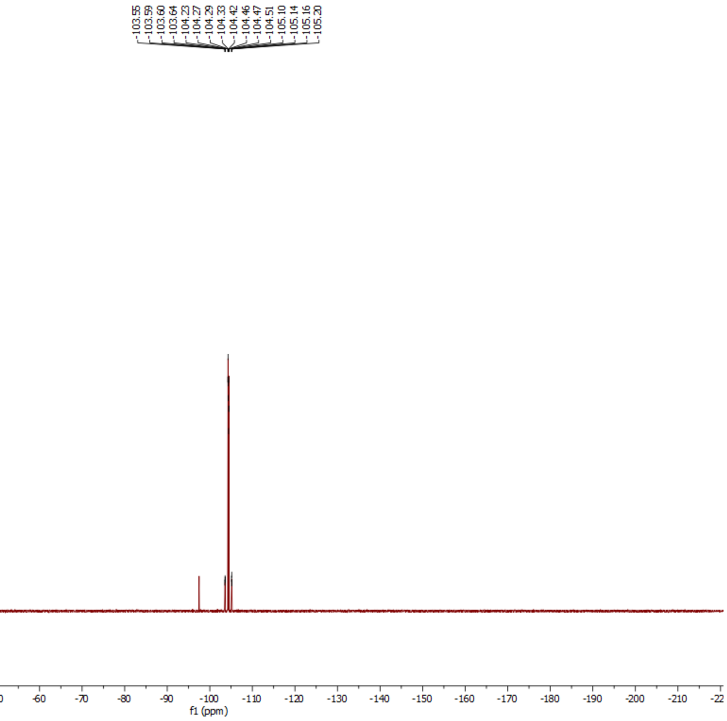

Ethyl (*R*)-2,2-difluoro-5-oxo-5-phenyl-4-(thiophen-2-yl)pentanoate (6j)

^{1}H NMR (400 MHz, Chloroform-*d*):

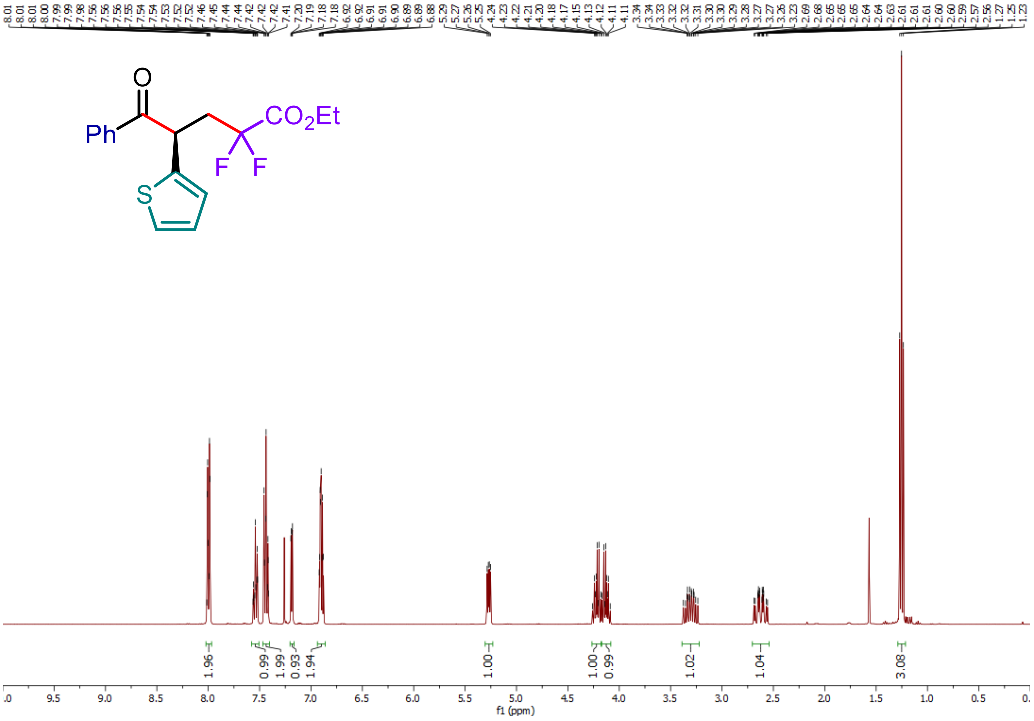

^{13}C NMR (101 MHz, Chloroform-*d*):

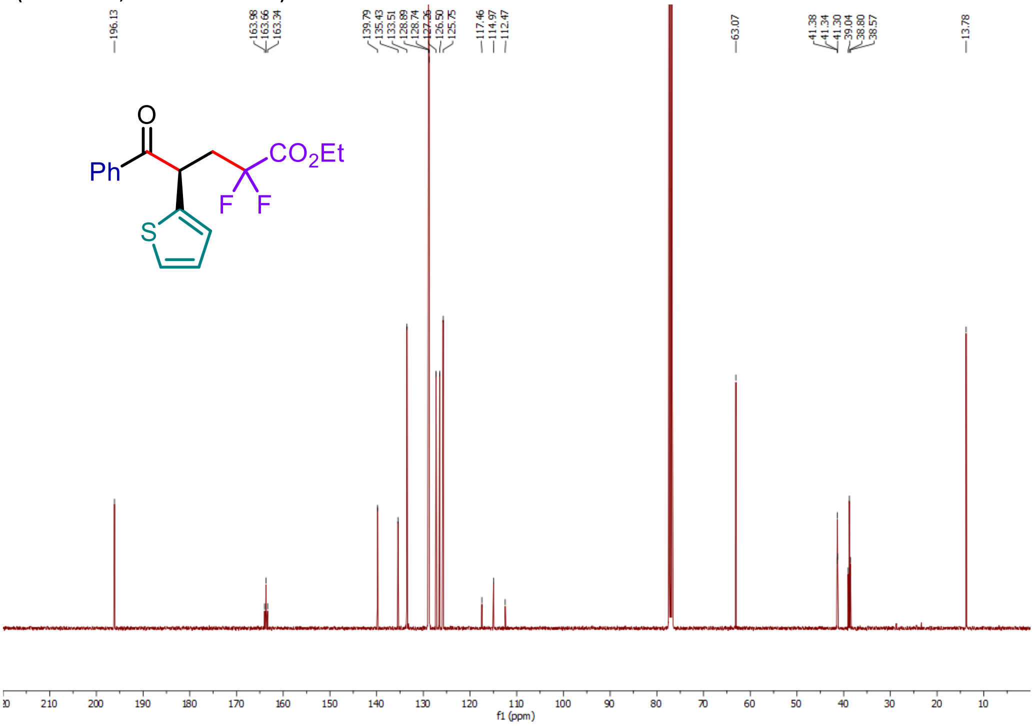

^{19}F NMR (376 MHz, Chloroform-*d*):

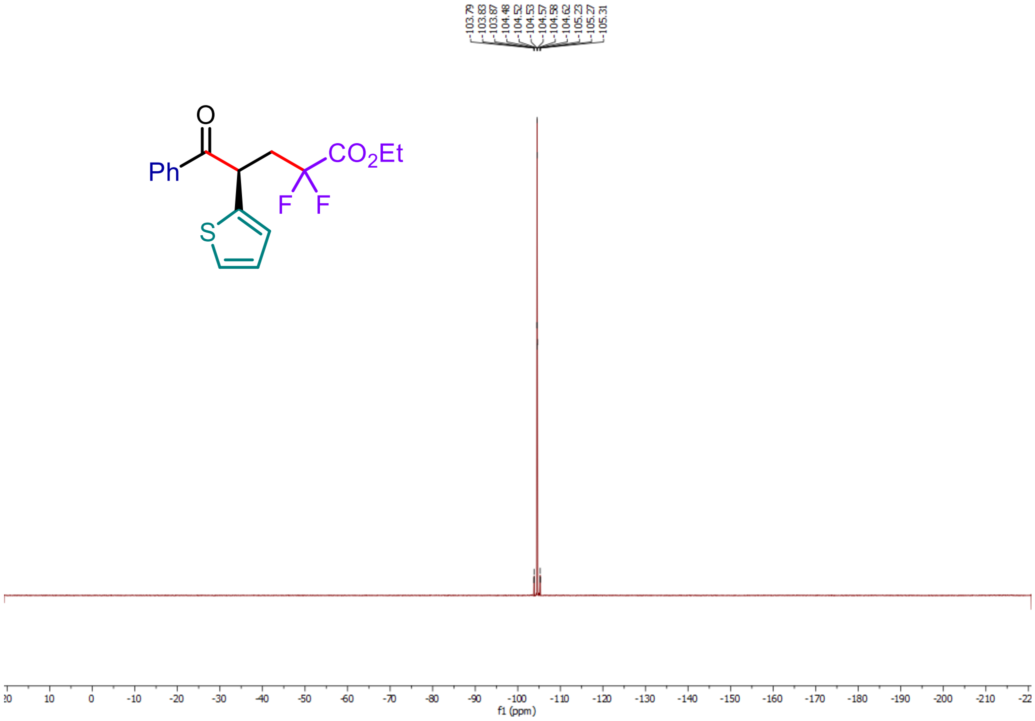

Ethyl (*S*)-2,2-difluoro-5-oxo-5-phenyl-4-(pyridin-3-yl)pentanoate (6k)

^{1}H NMR (400 MHz, Chloroform-*d*):

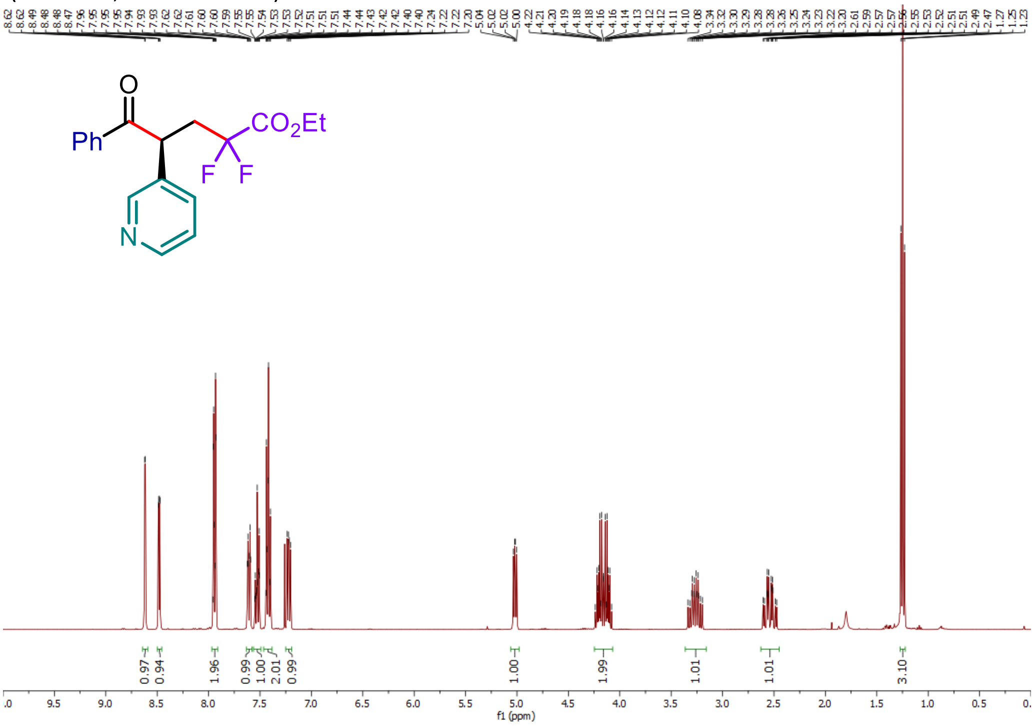

^{13}C NMR (101 MHz, Chloroform-*d*):

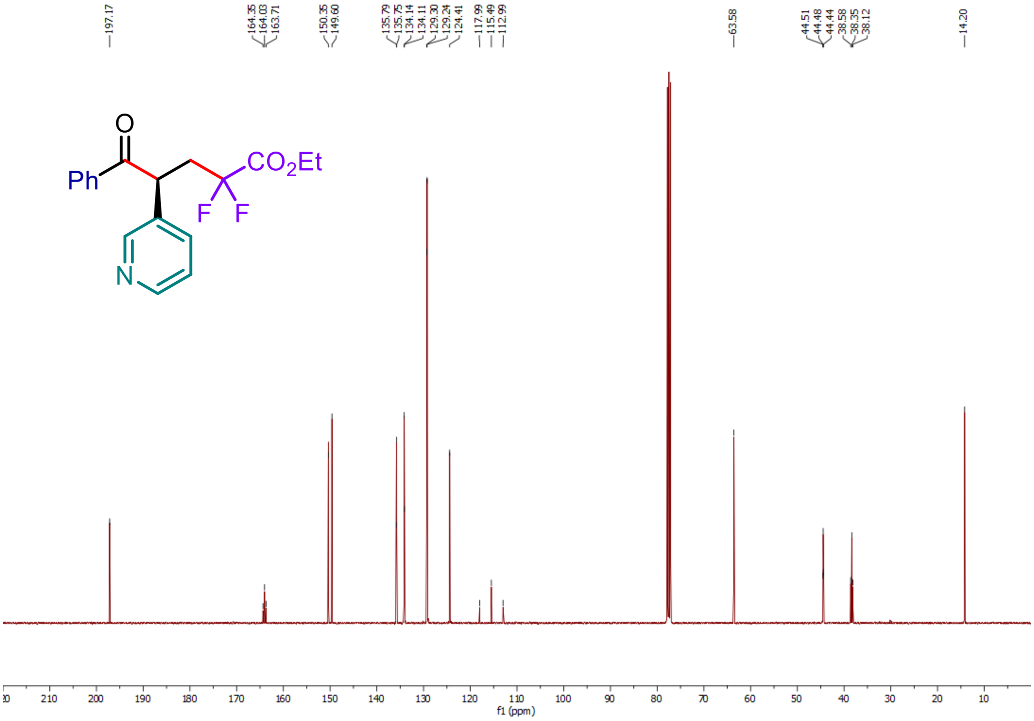

^{19}F NMR (376 MHz, Chloroform-*d*):

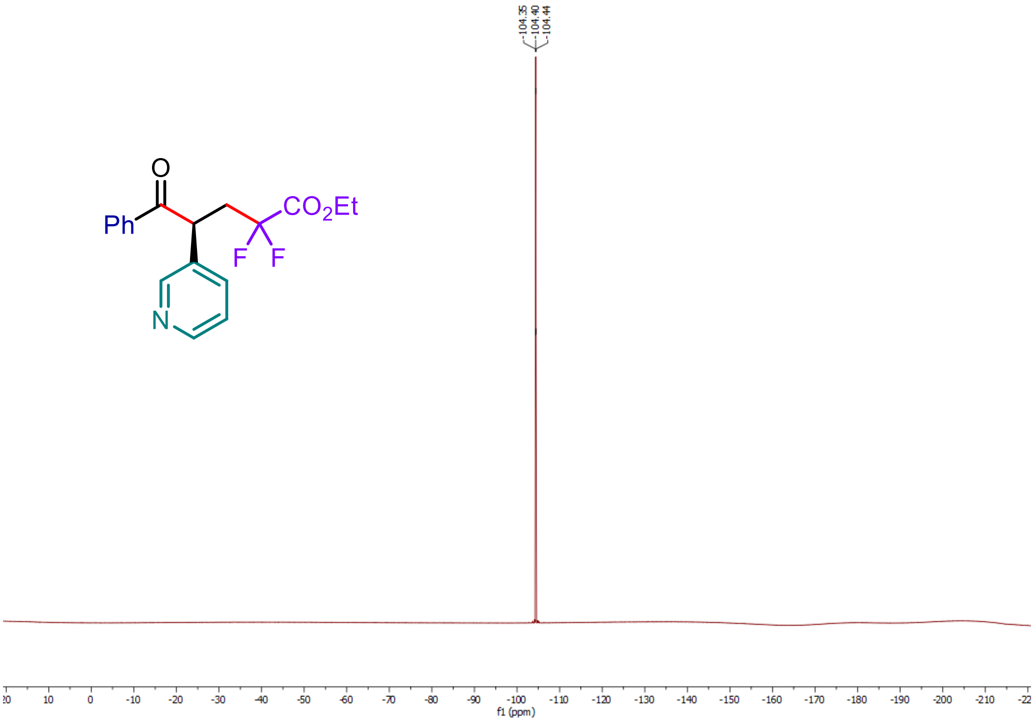

Ethyl (*S*)-2,2-difluoro-5-oxo-5-phenyl-4-(pyridin-4-yl)pentanoate (6l)

^{1}H NMR (400 MHz, Chloroform-*d*):

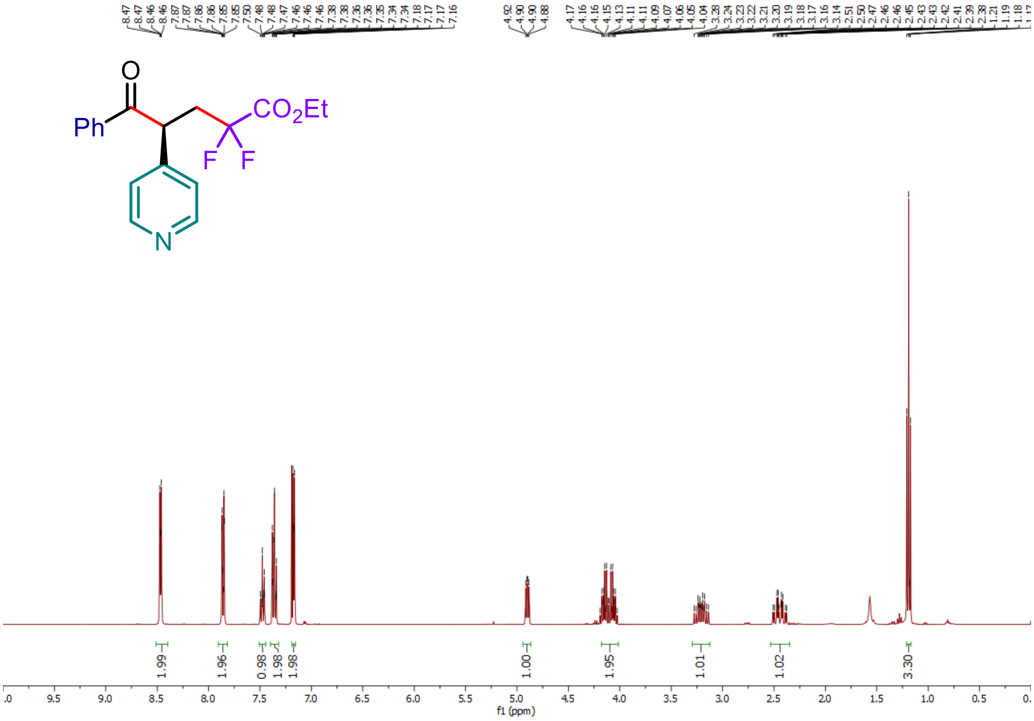

^{13}C NMR (101 MHz, Chloroform-*d*):

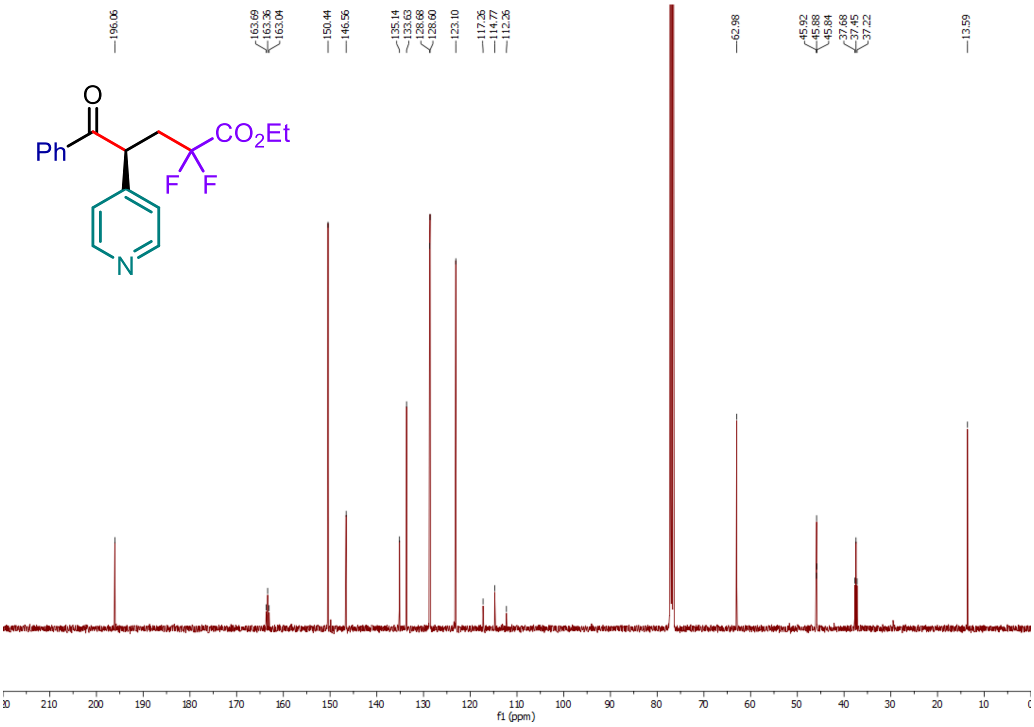

¹⁹F NMR (376 MHz, Chloroform-*d*):

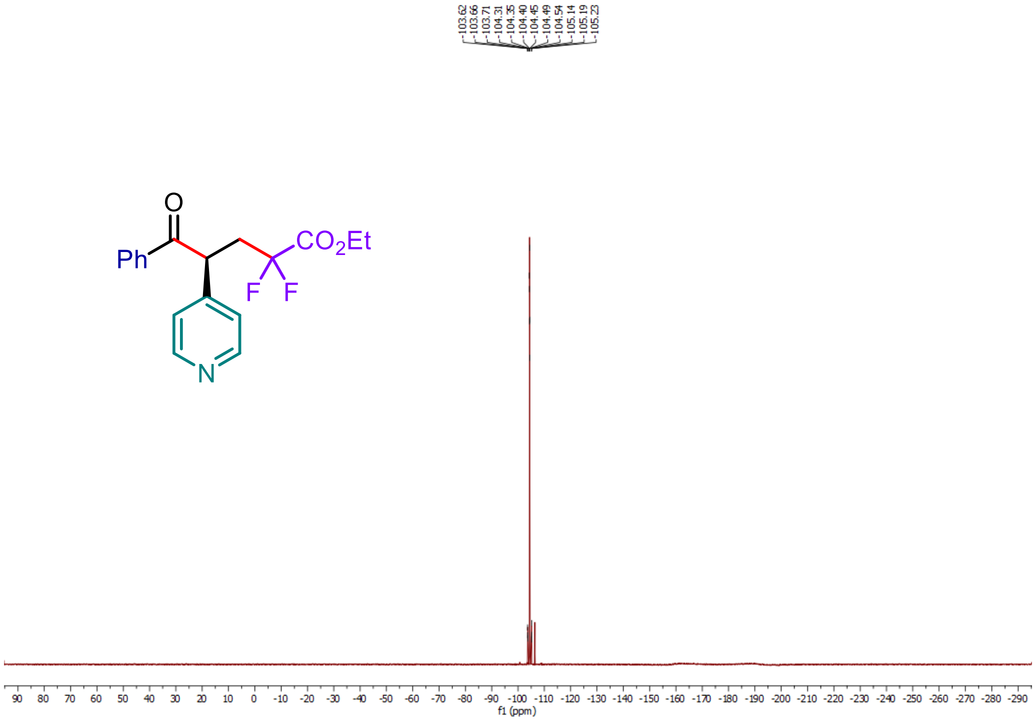

Ethyl (*S*)-4-(1-benzyl-1H-indol-3-yl)-2,2-difluoro-5-oxo-5-phenylpentanoate (6m)

^{1}H NMR (400 MHz, Chloroform-*d*):

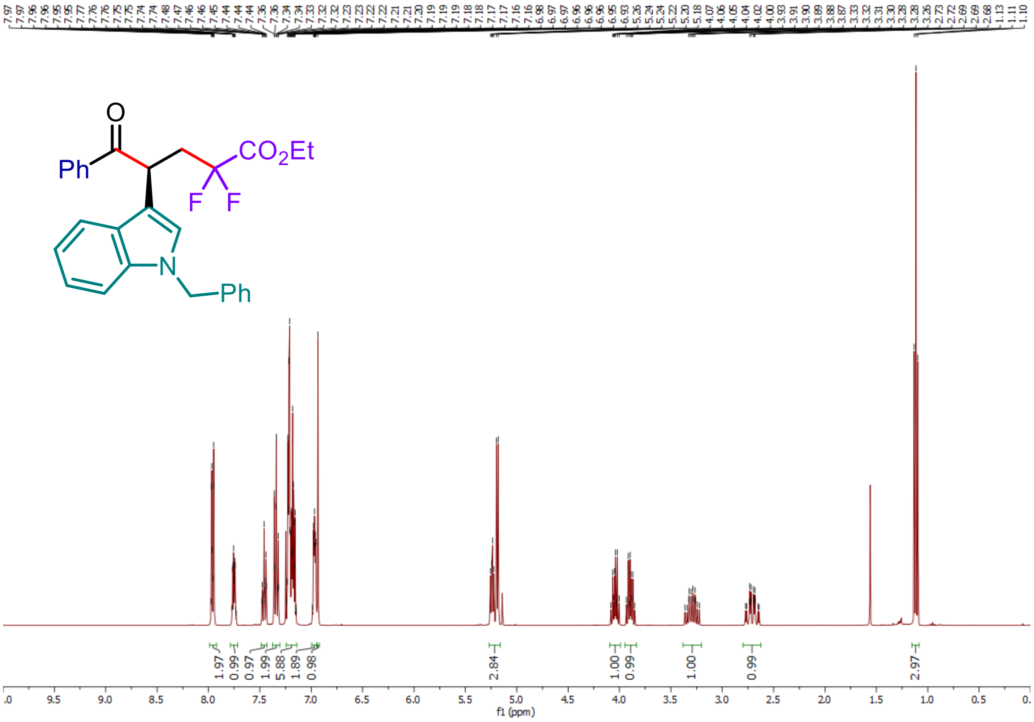

^{13}C NMR (101 MHz, Chloroform-*d*):

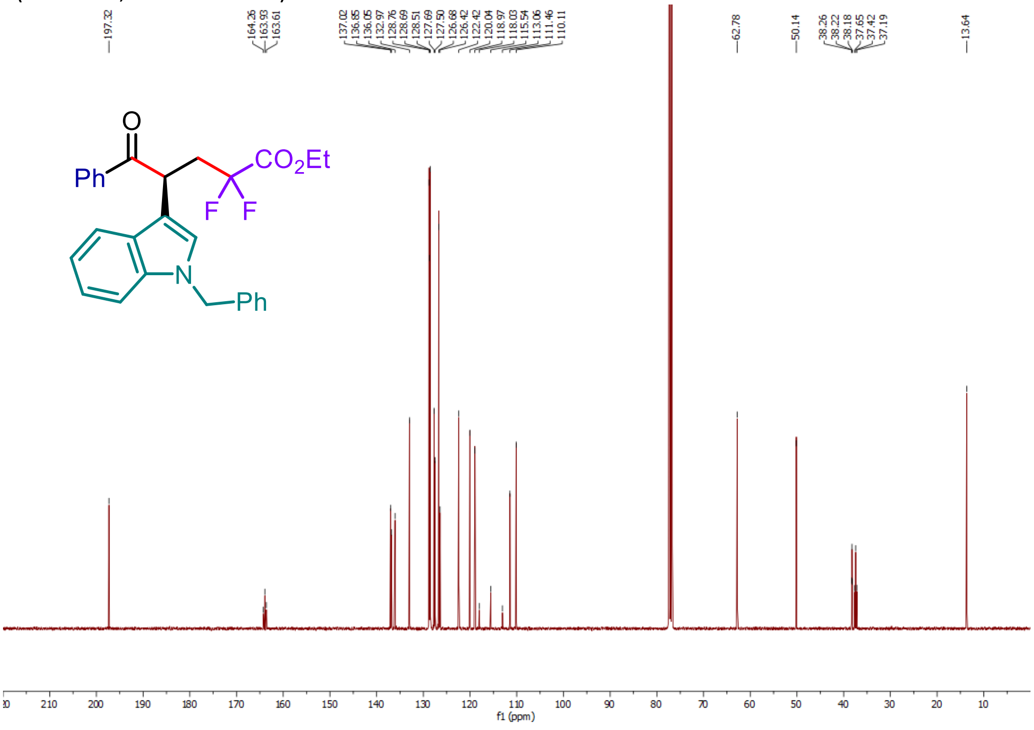

^{19}F NMR (376 MHz, Chloroform-*d*):

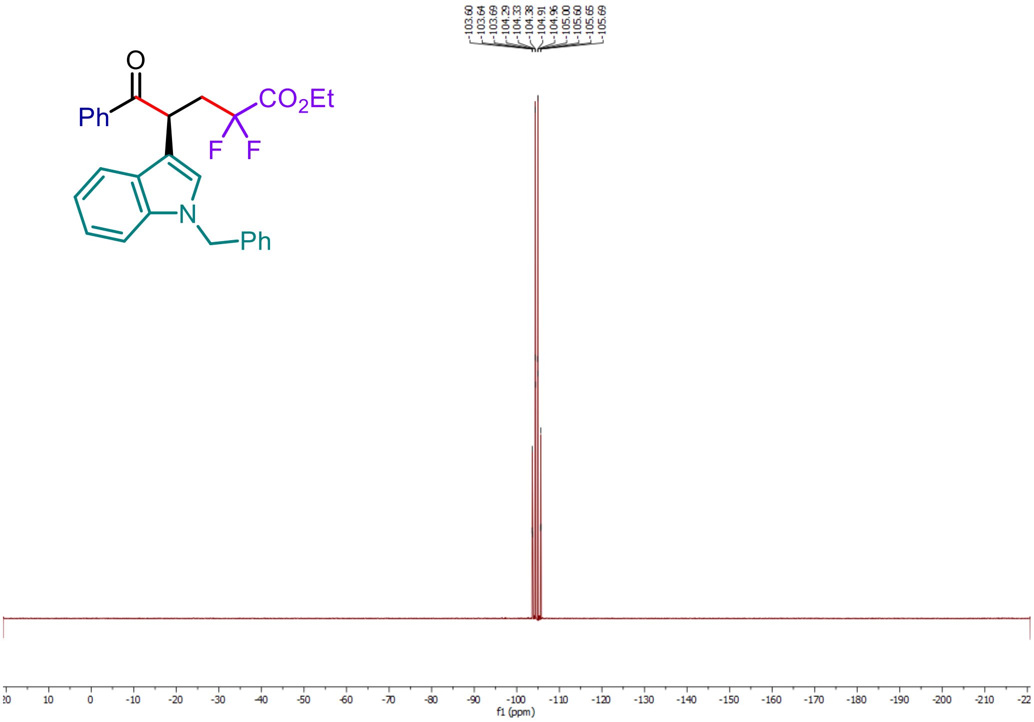

Ethyl (*R*)-2,2-difluoro-5-oxo-4-phenoxy-5-phenylpentanoate (6n)

^{1}H NMR (400 MHz, Chloroform-*d*):

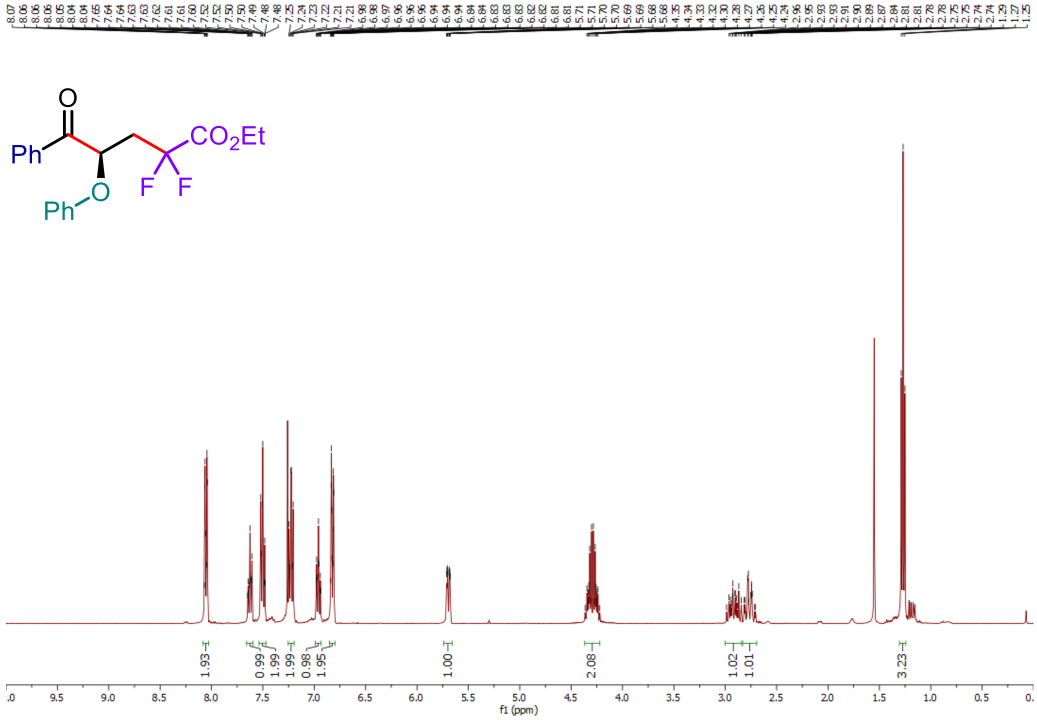

^{13}C NMR (101 MHz, Chloroform-*d*):

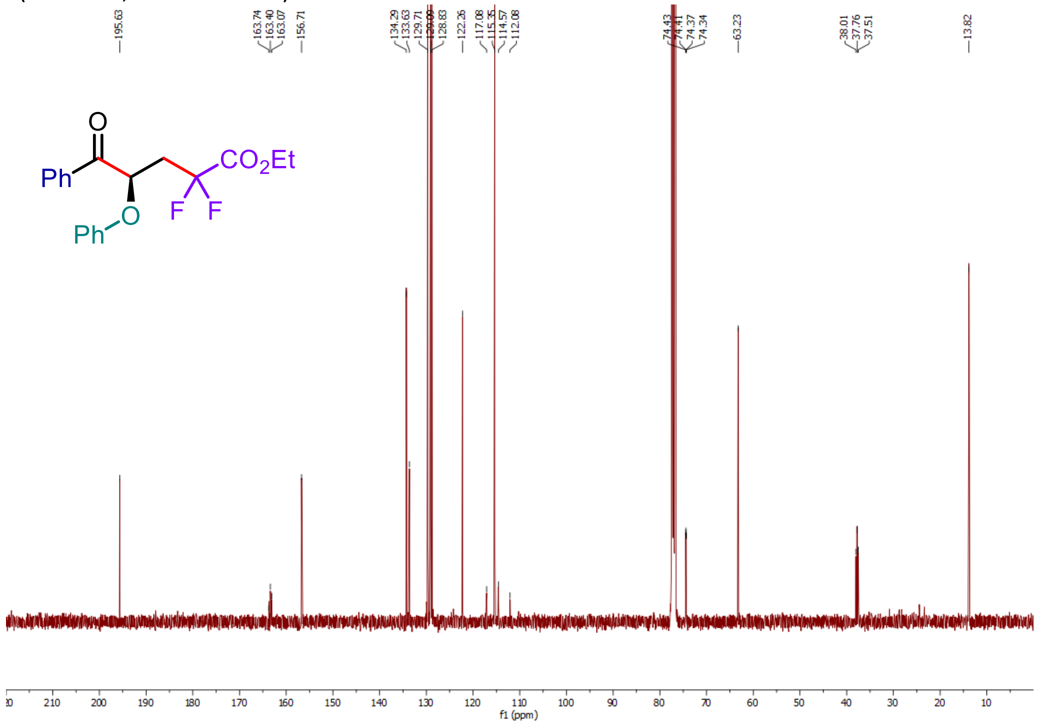

^{19}F NMR (376 MHz, Chloroform-*d*):

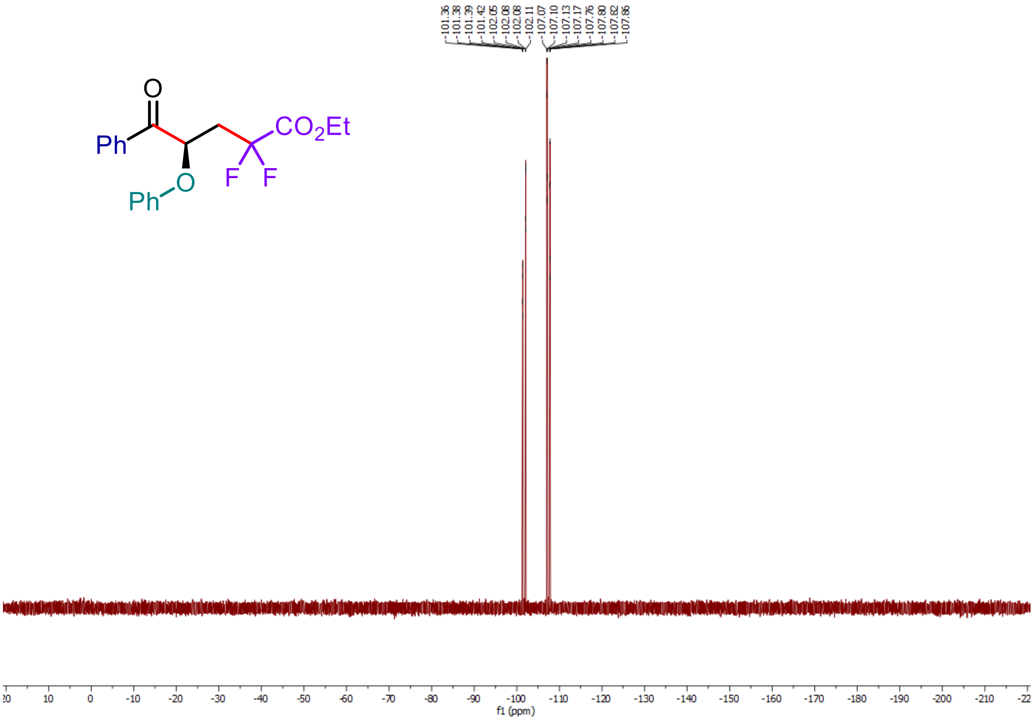

Ethyl (*R*)-2,2-difluoro-5-oxo-5-phenyl-4-(phenylthio)pentanoate (6o)

^{1}H NMR (400 MHz, Chloroform-*d*):

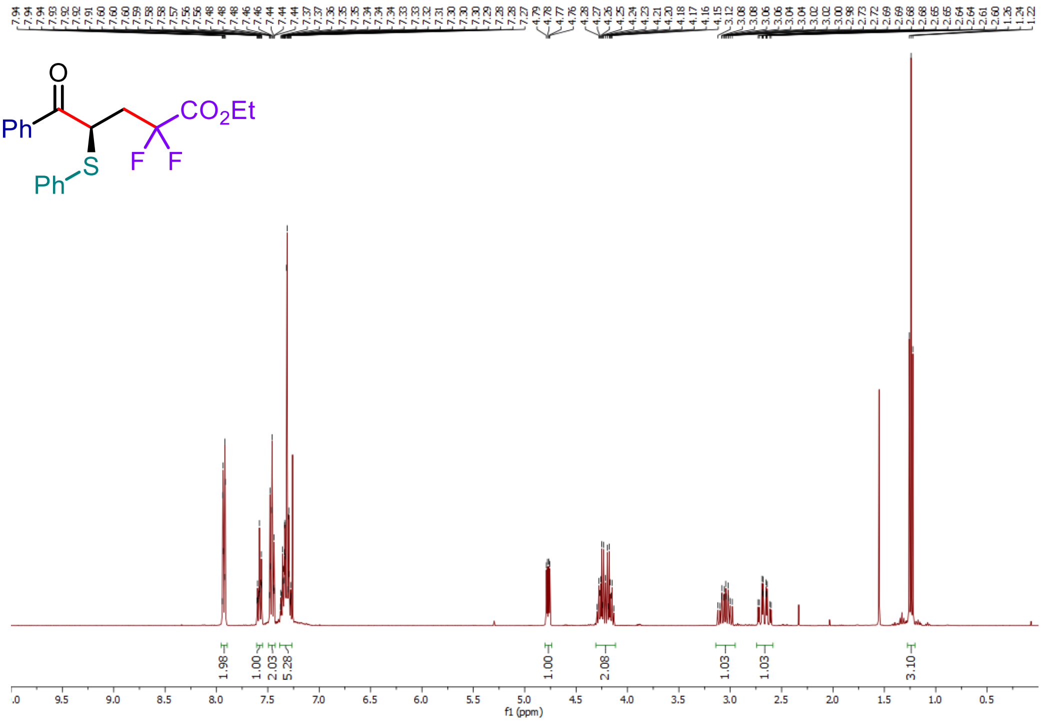

^{13}C NMR (101 MHz, Chloroform-*d*):

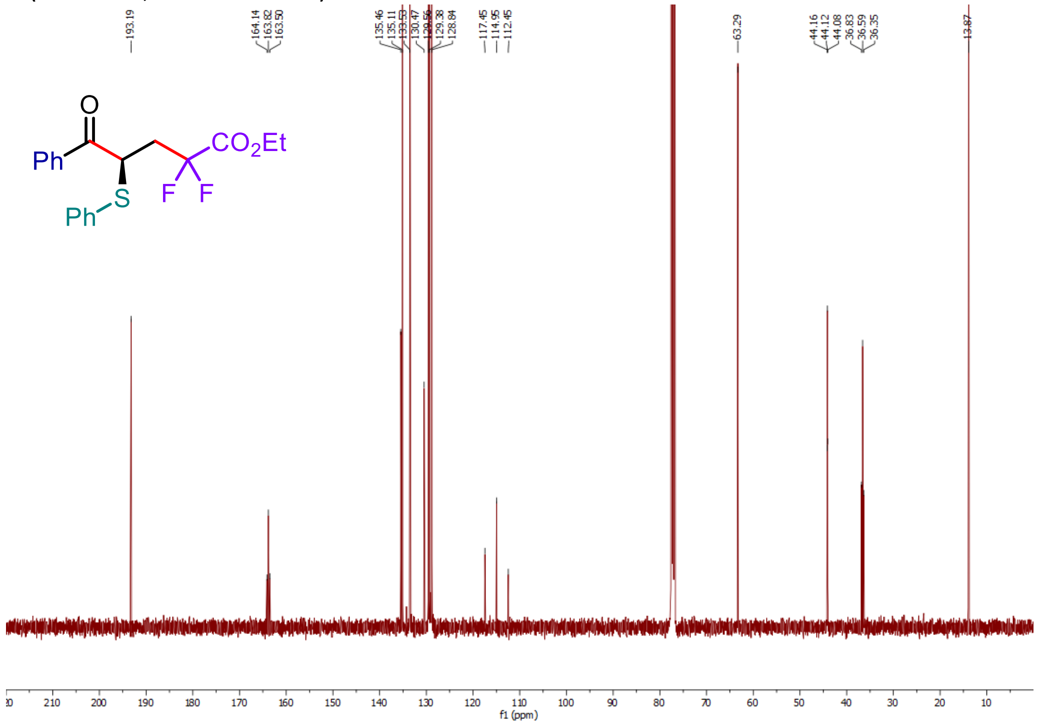

^{19}F NMR (376 MHz, Chloroform-*d*):

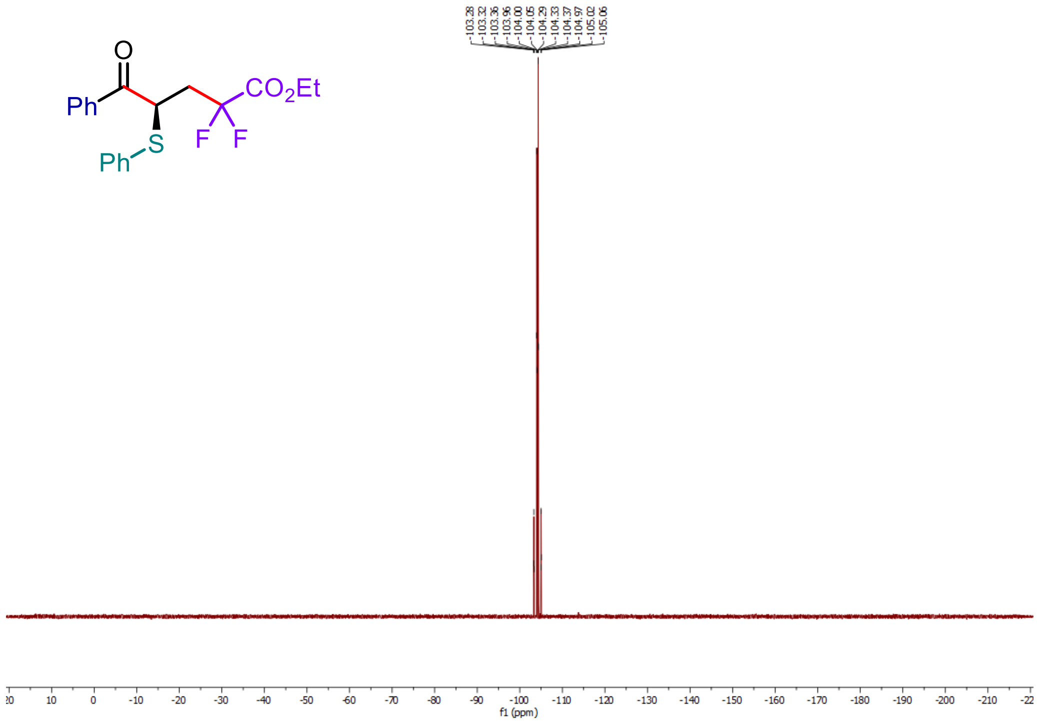

Ethyl (*R*)-4-(1,3-dioxoisoindolin-2-yl)-2,2-difluoro-5-oxo-5-phenylpentanoate (6p)

^{1}H NMR (400 MHz, Chloroform-*d*):

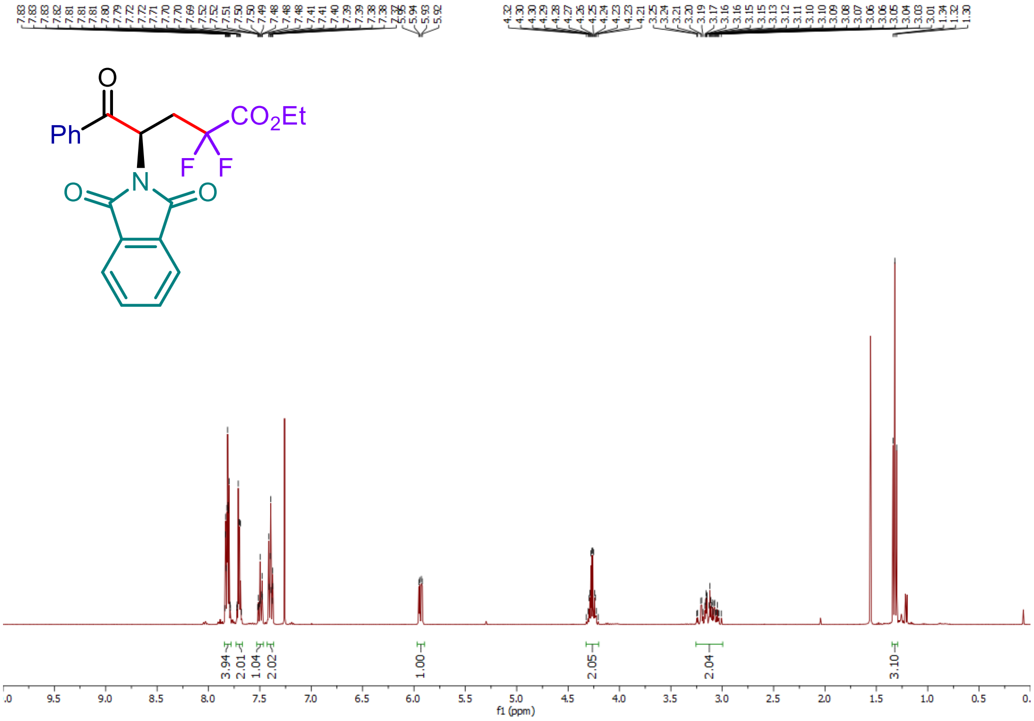

^{13}C NMR (101 MHz, Chloroform-*d*):

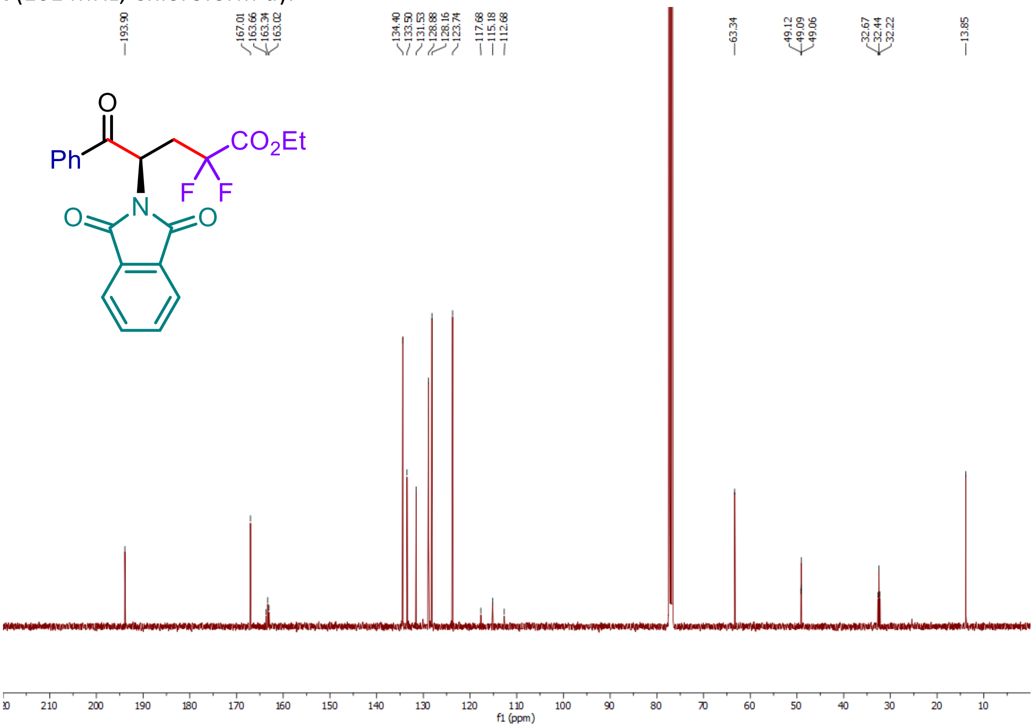

^{19}F NMR (376 MHz, Chloroform-*d*):

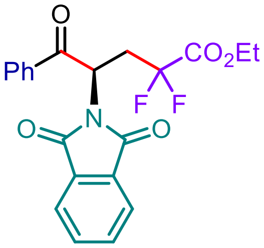

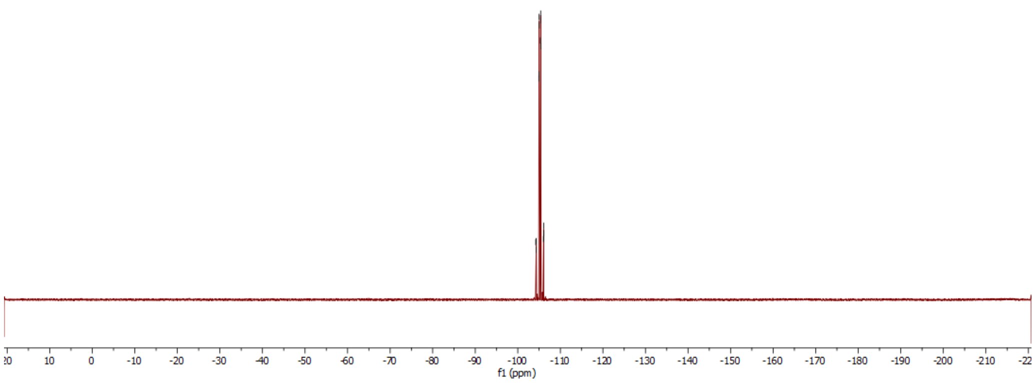

Ethyl (*R*)-4-acetoxy-2,2-difluoro-4-methyl-5-oxo-5-phenylpentanoate (6q)

^{1}H NMR (400 MHz, Chloroform-*d*):

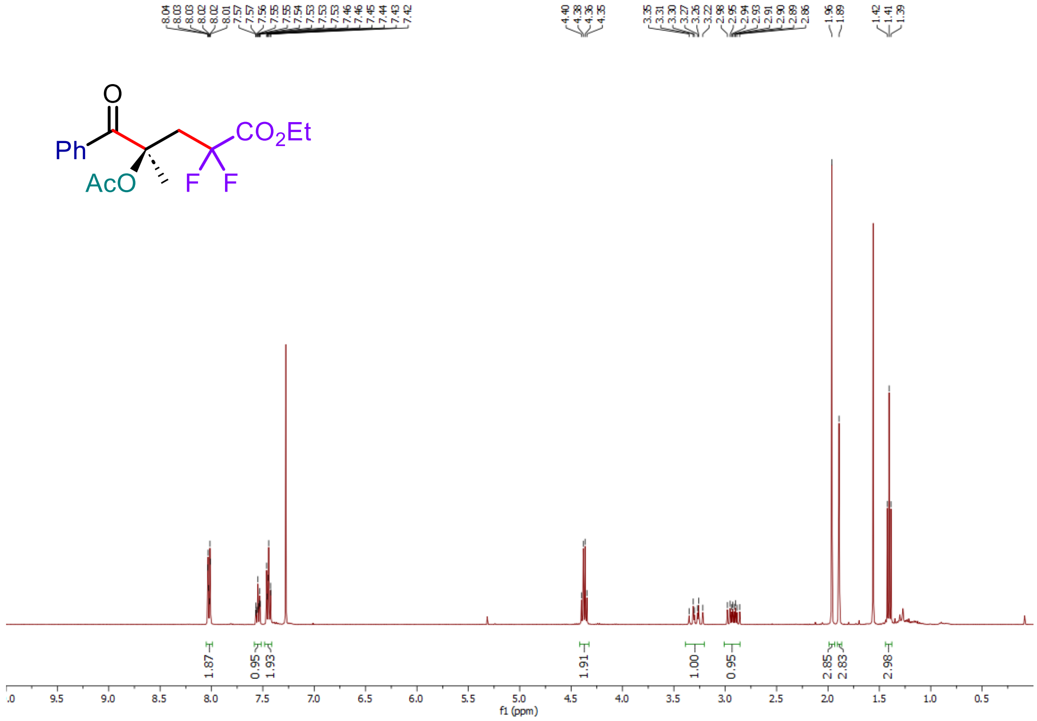

^{13}C NMR (101 MHz, Chloroform-*d*):

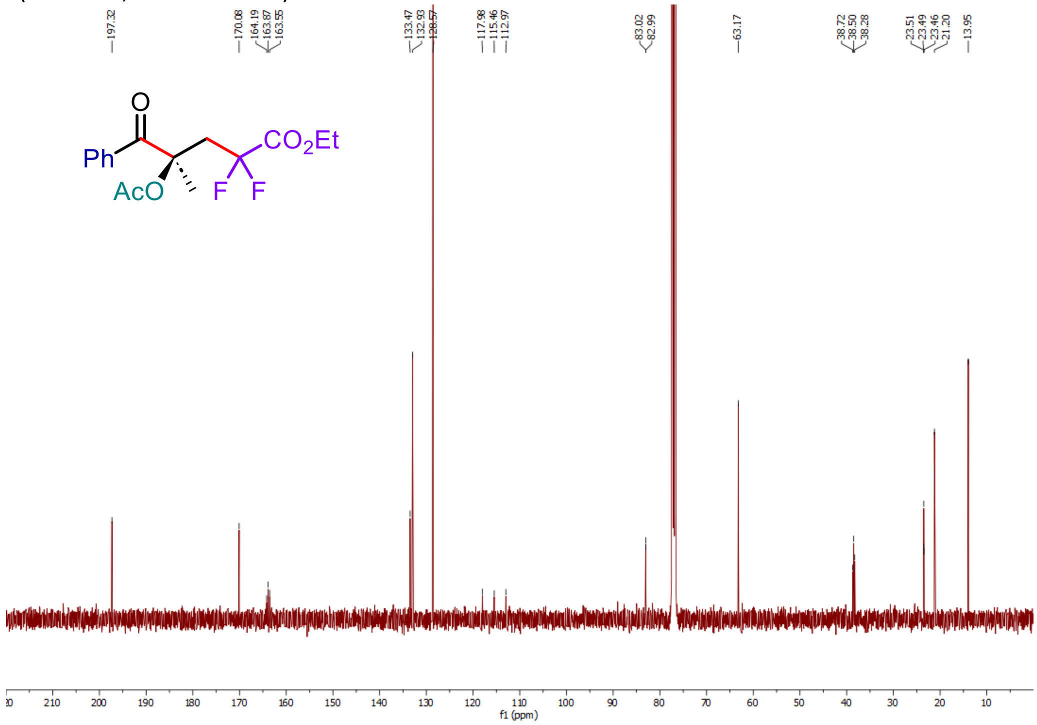

^{19}F NMR (376 MHz, Chloroform-*d*):

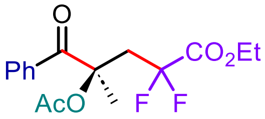

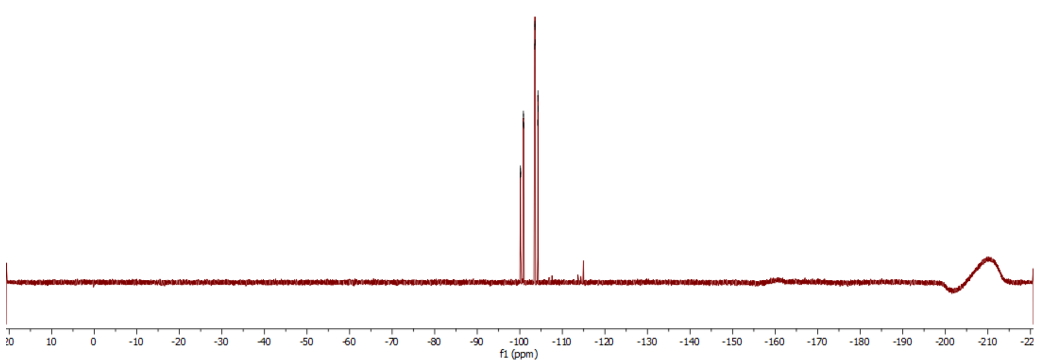

Ethyl (*R*)-2,2-difluoro-5-oxo-5-phenyl-4-((trimethylsilyl)methyl)pentanoate (6r)

^{1}H NMR (400 MHz, Chloroform-*d*):

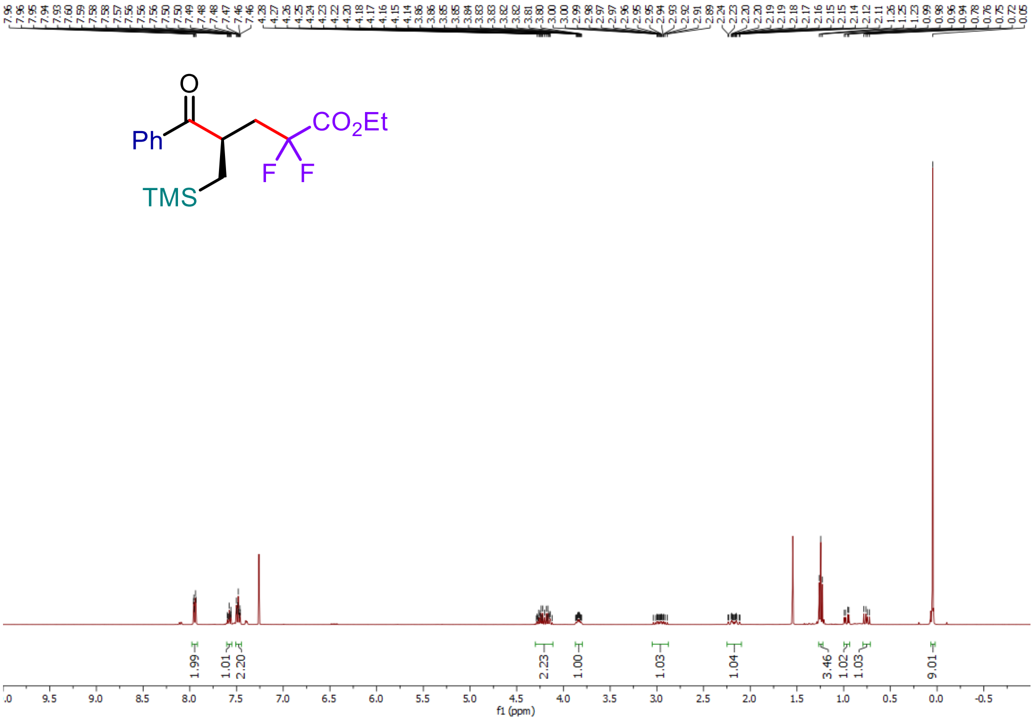

^{13}C NMR (101 MHz, Chloroform-*d*):

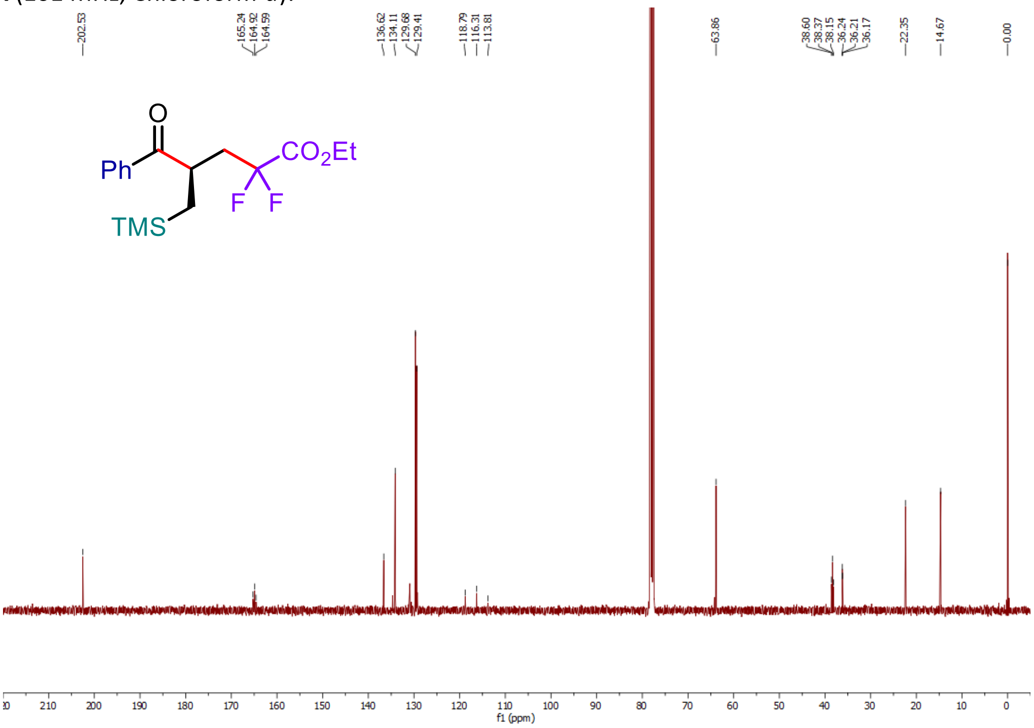

^{19}F NMR (376 MHz, Chloroform-*d*):

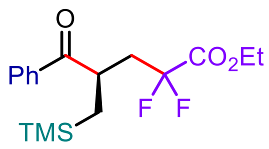

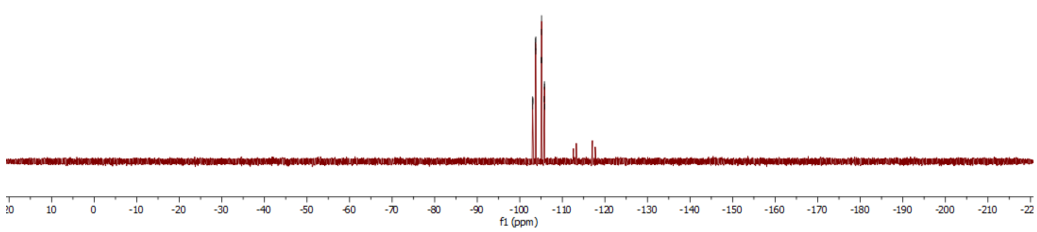

Ethyl (*R*)-4-benzoyl-2,2-difluoro-6,6-dimethylheptanoate (6s)

^{1}H NMR (400 MHz, Chloroform-*d*):

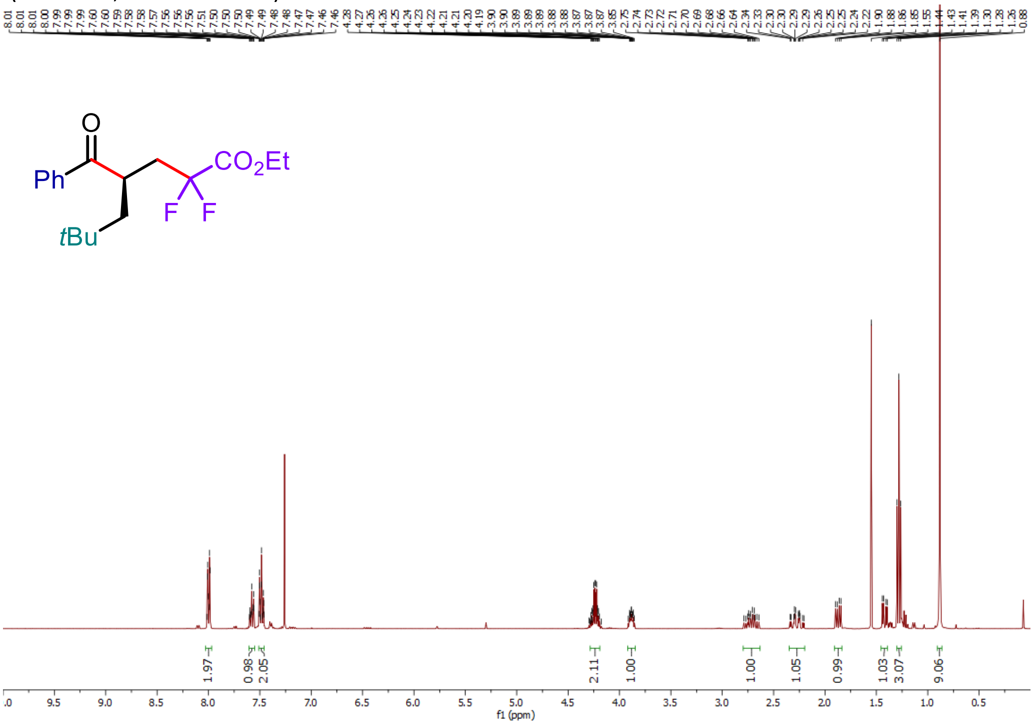

^{13}C NMR (101 MHz, Chloroform-*d*):

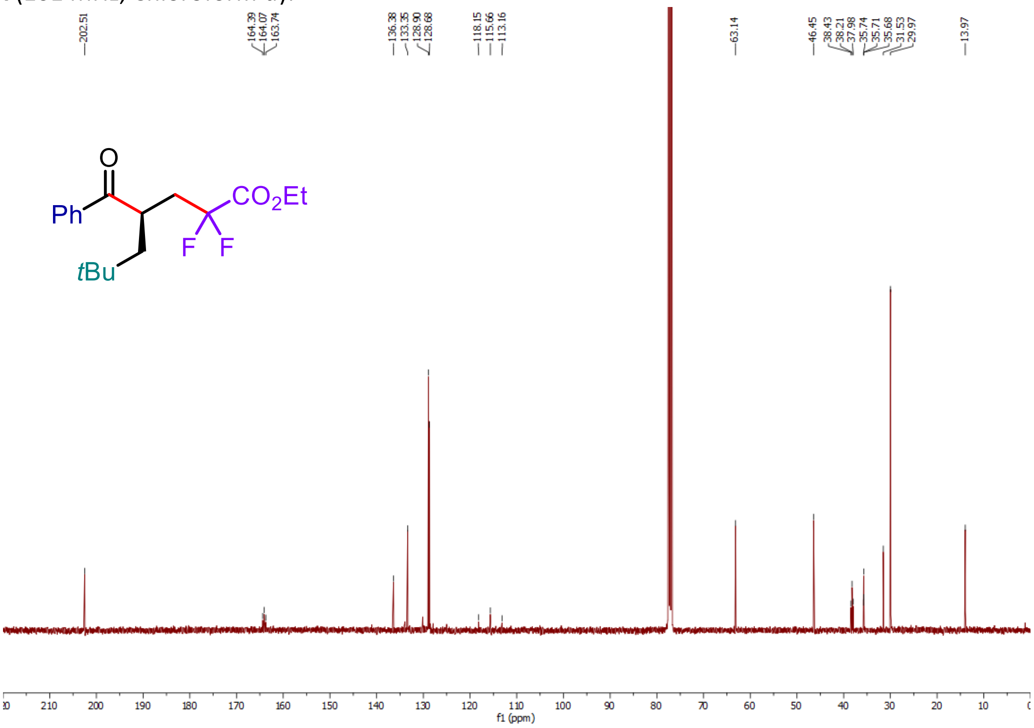

^{19}F NMR (376 MHz, Chloroform-*d*):

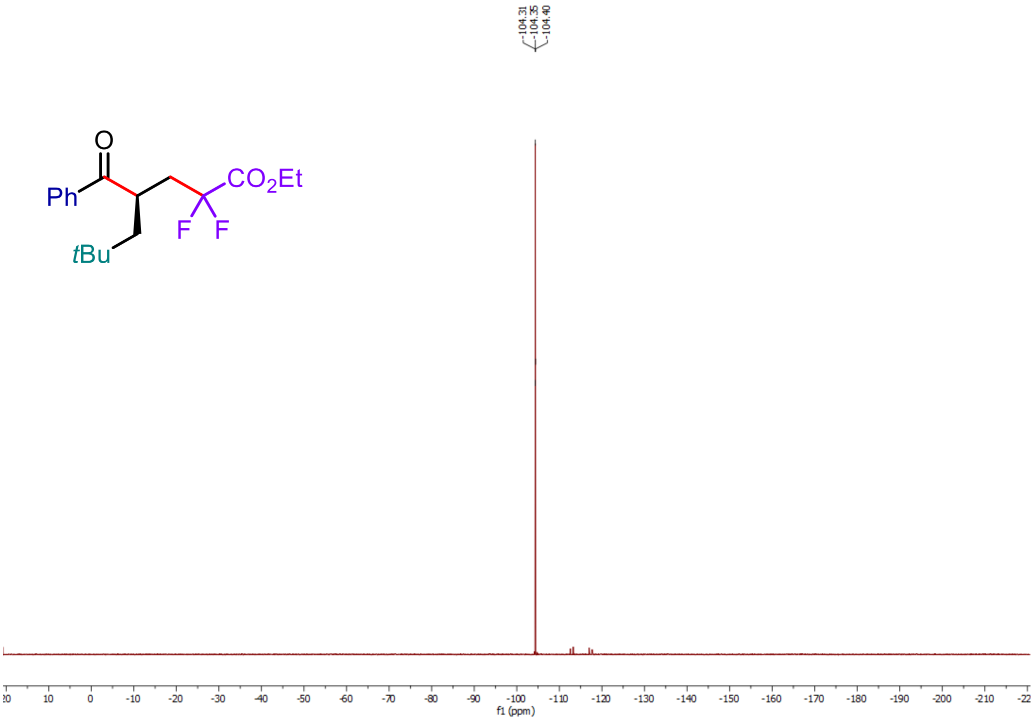

Ethyl (*R*)-4-benzyl-2,2-difluoro-5-oxo-5-phenylpentanoate (6t)

^{1}H NMR (400 MHz, Chloroform-*d*):

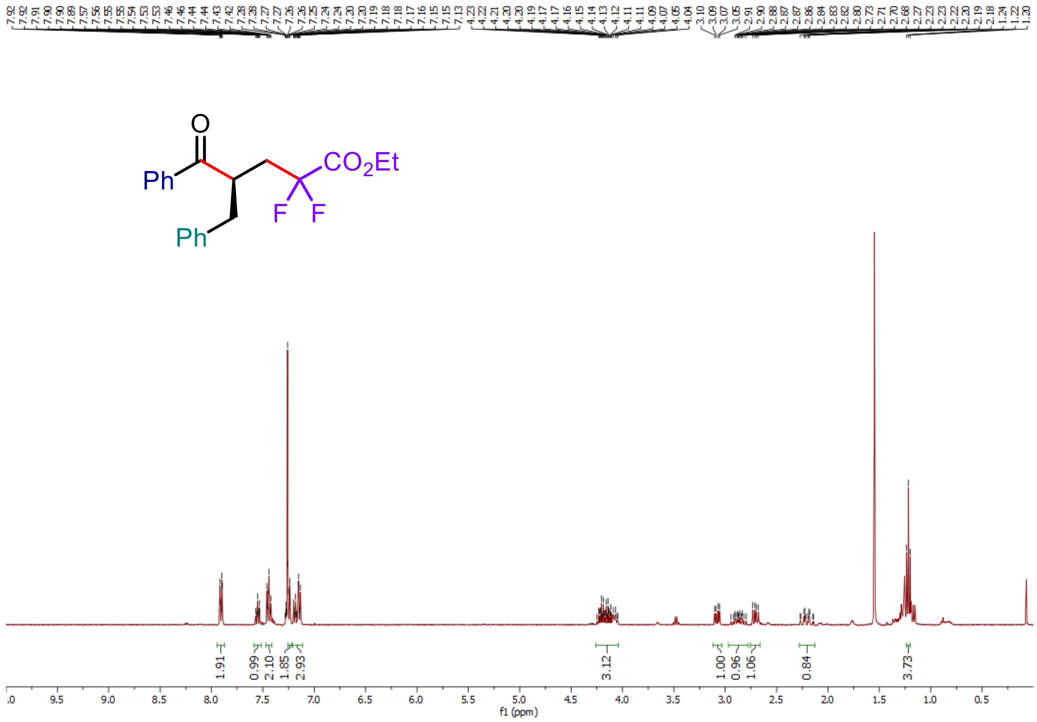

^{13}C NMR (101 MHz, Chloroform-*d*):

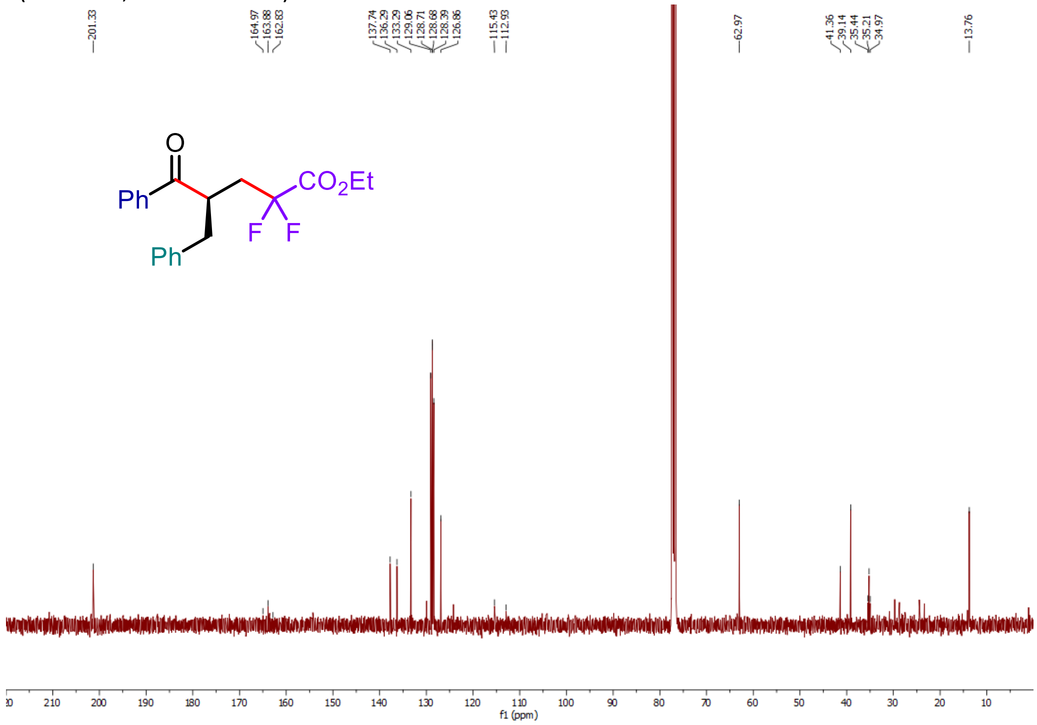

^{19}F NMR (376 MHz, Chloroform-*d*):

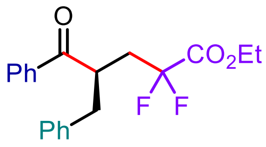

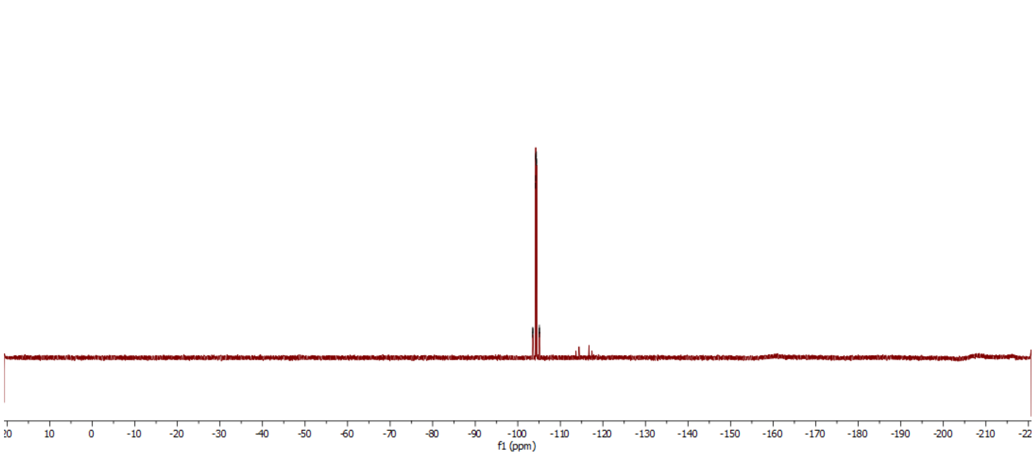

tert-Pentyl (_S_)-2,2-difluoro-5-oxo-4,5-diphenylpentanoate (7a)

^{1}H NMR (400 MHz, Chloroform-_d_):

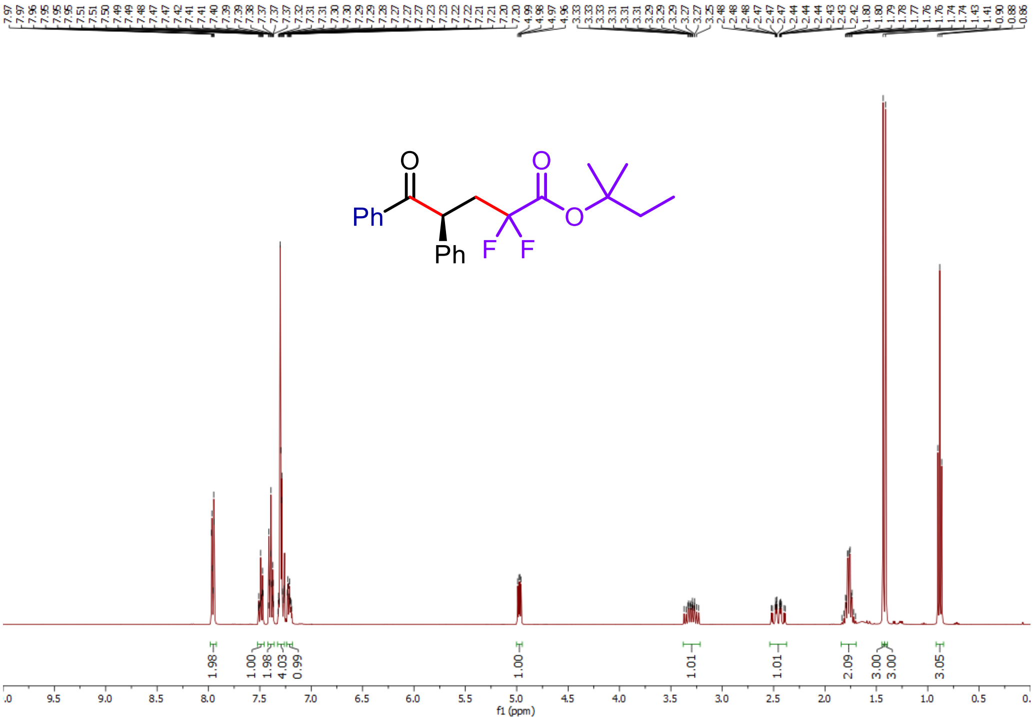

^{13}C NMR (101 MHz, Chloroform-_d_):

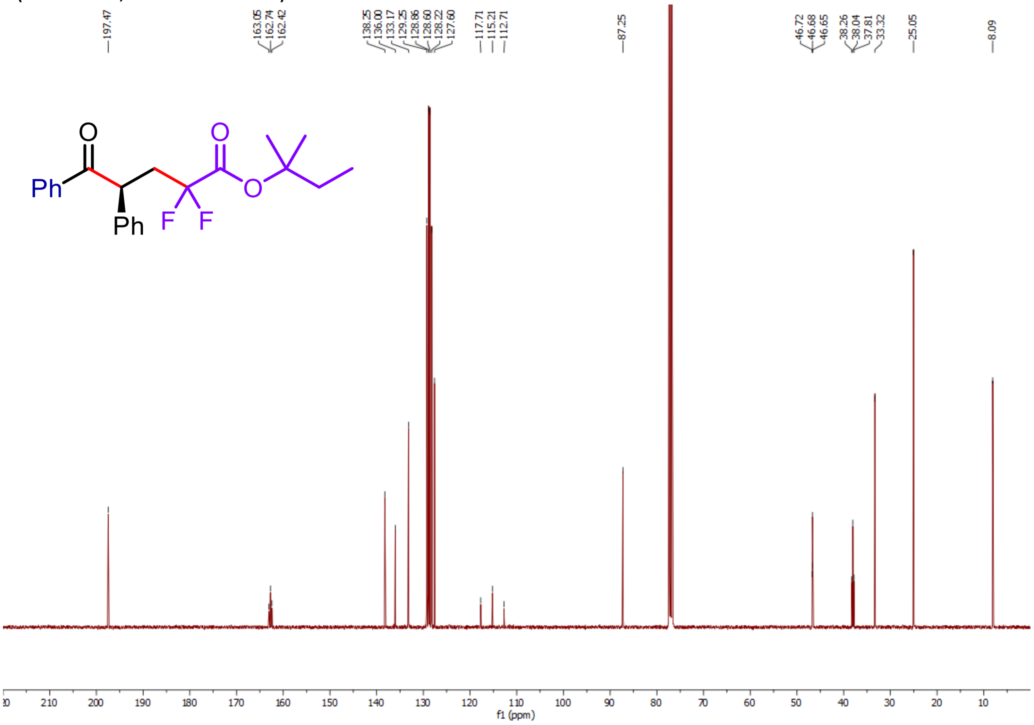

^{19}F NMR (376 MHz, Chloroform-*d*):

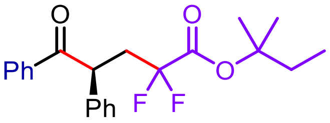

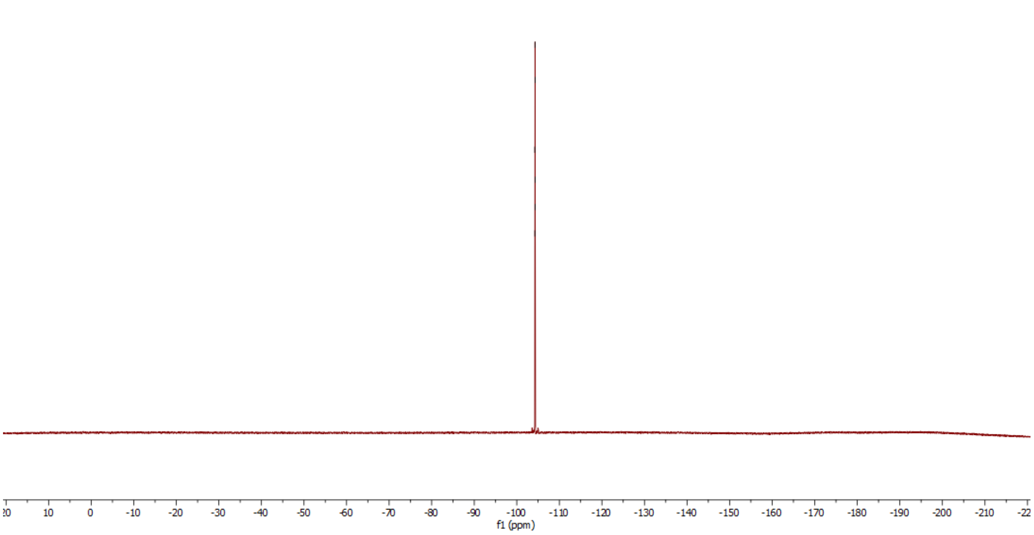

Cyclohexyl (*S*)-2,2-difluoro-5-oxo-4,5-diphenylpentanoate (7b)

^{1}H NMR (400 MHz, Chloroform-*d*):

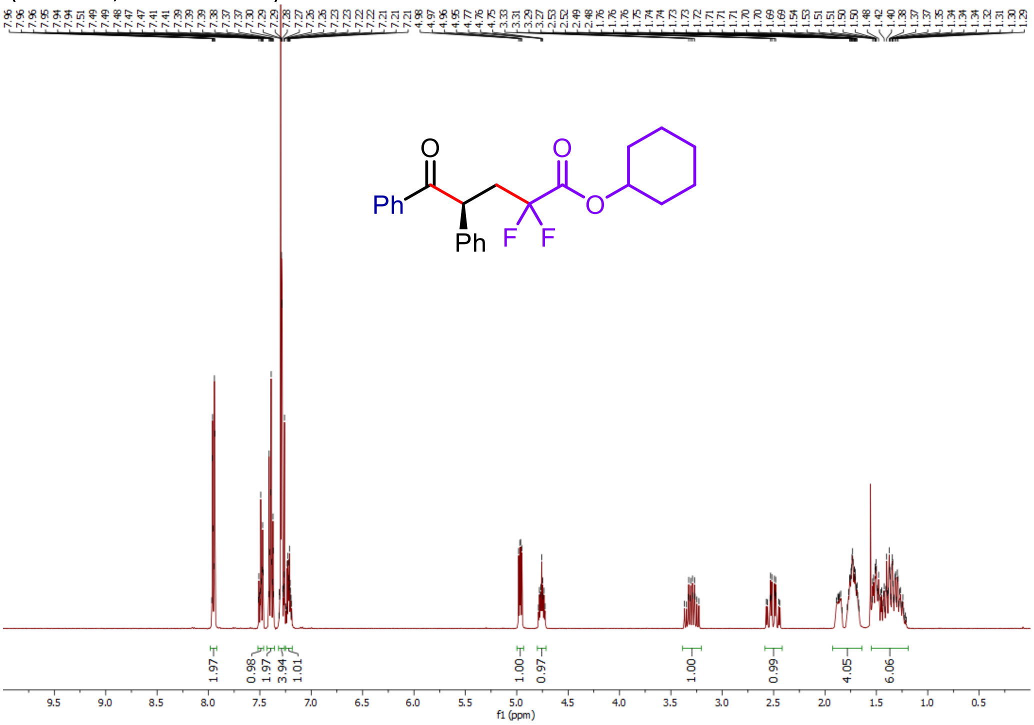

^{13}C NMR (101 MHz, Chloroform-*d*):

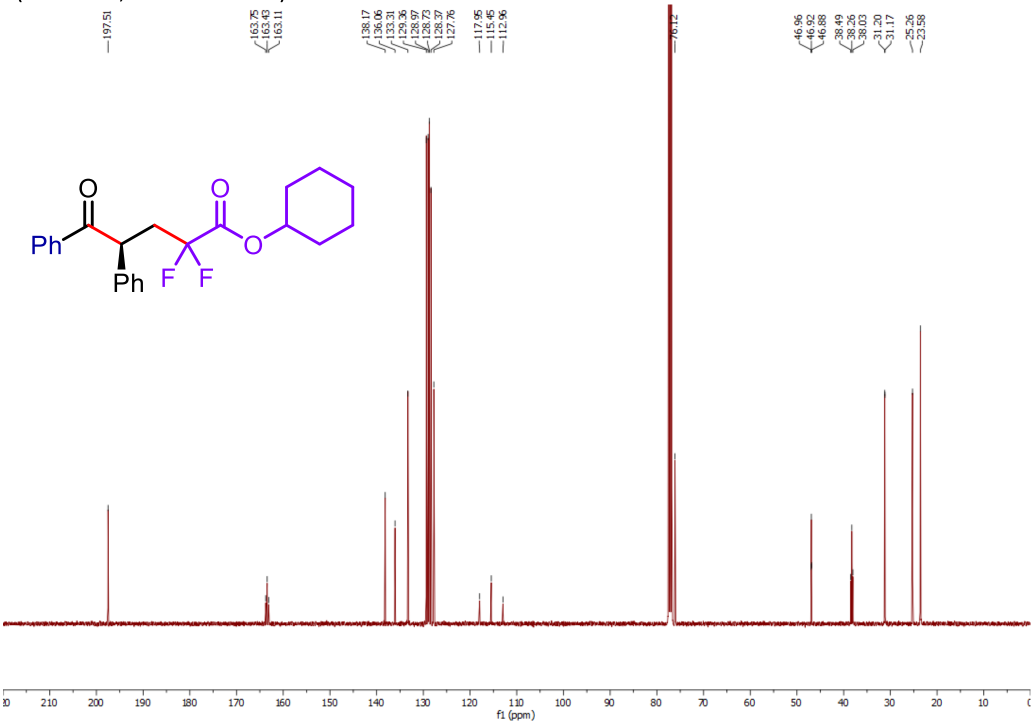

^{19}F NMR (376 MHz, Chloroform-*d*):

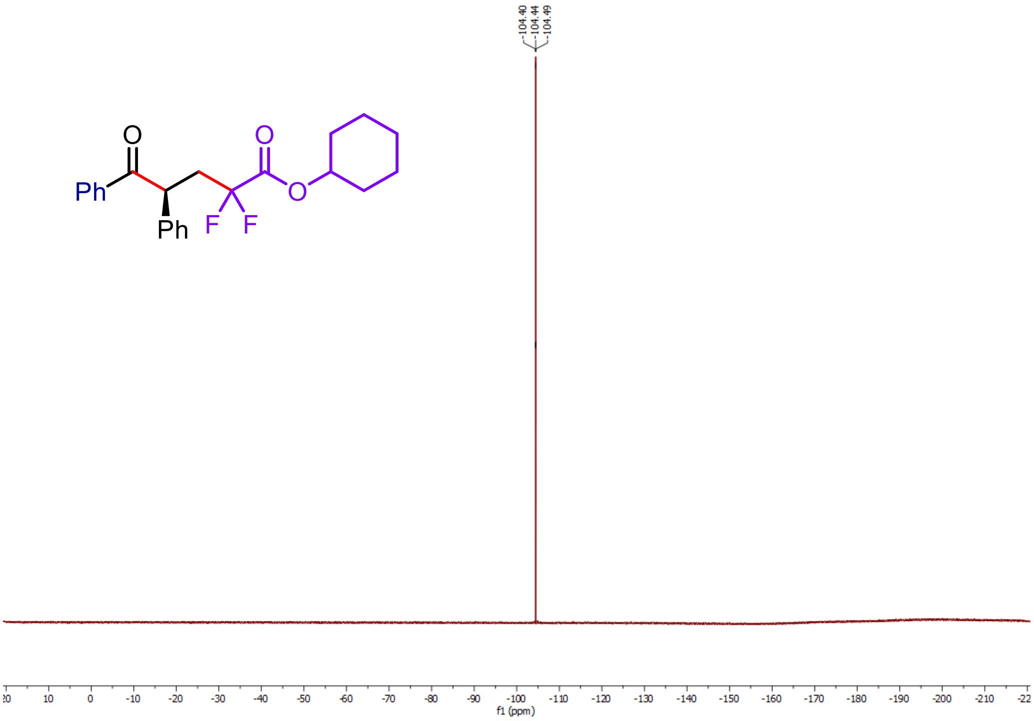

Allyl (*S*)-2,2-difluoro-5-oxo-4,5-diphenylpentanoate (7c)

^{1}H NMR (400 MHz, Chloroform-*d*):

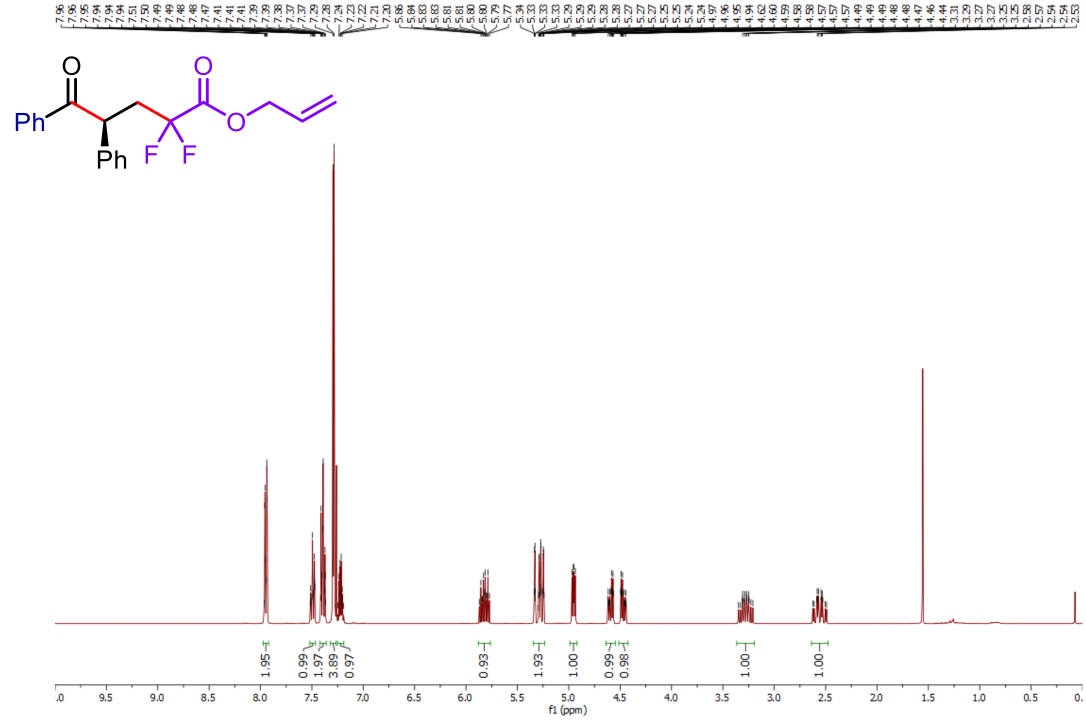

^{13}C NMR (101 MHz, Chloroform-*d*):

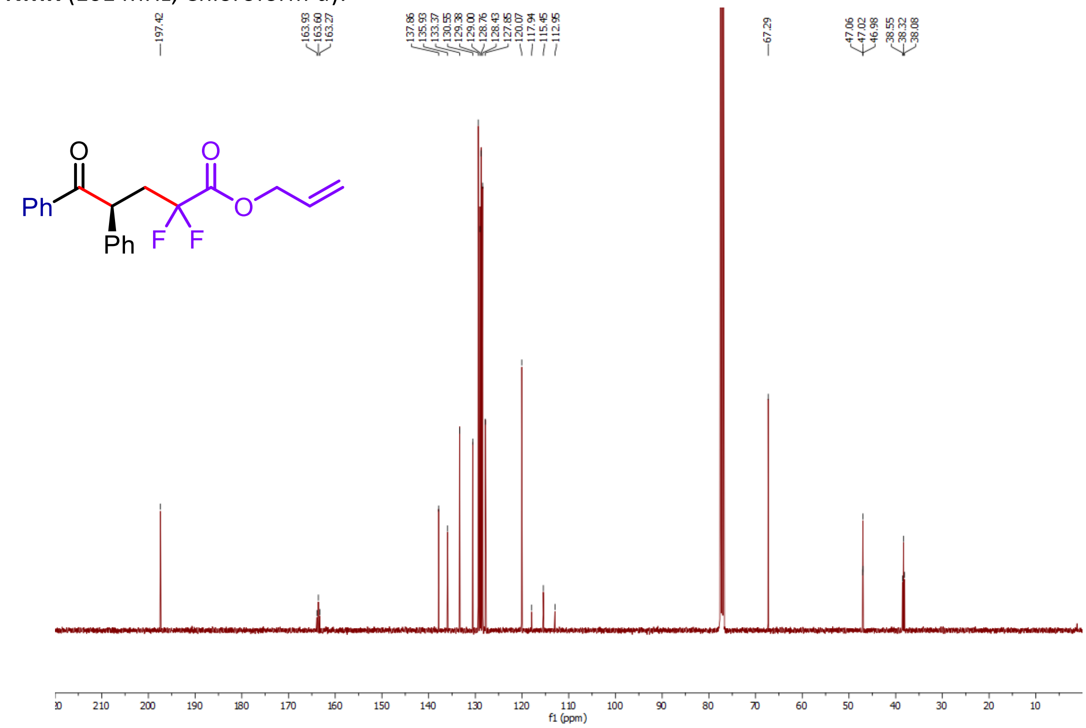

^{19}F NMR (376 MHz, Chloroform-*d*):

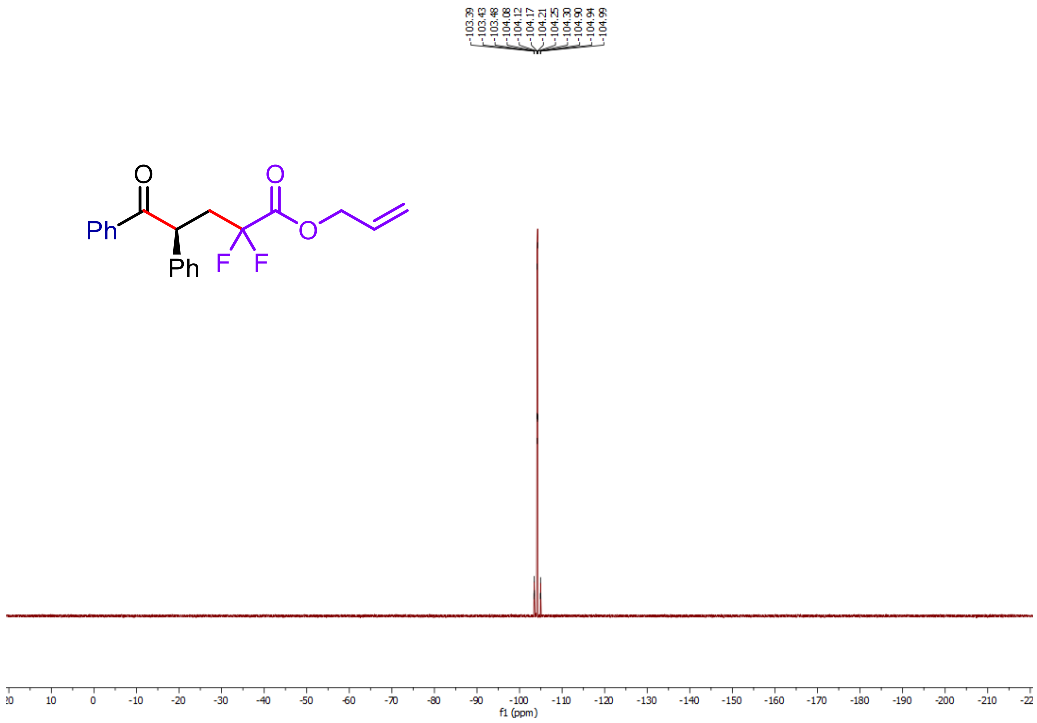

Prop-2-yn-1-yl (*S*)-2,2-difluoro-5-oxo-4,5-diphenylpentanoate (7d)

^{1}H NMR (400 MHz, Chloroform-*d*):

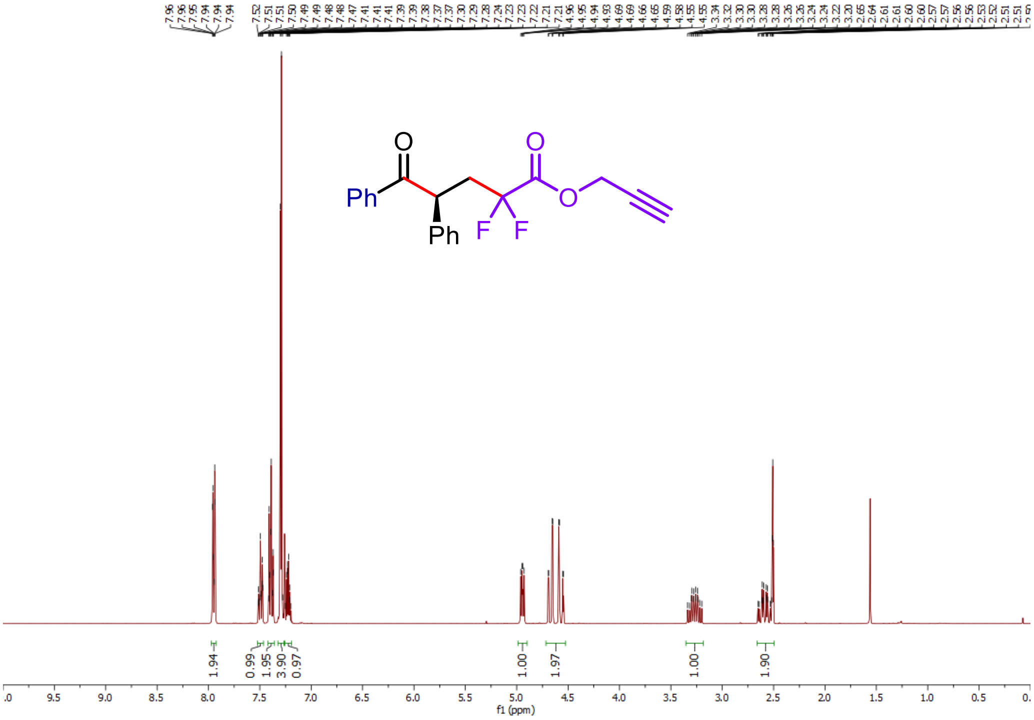

^{13}C NMR (101 MHz, Chloroform-*d*):

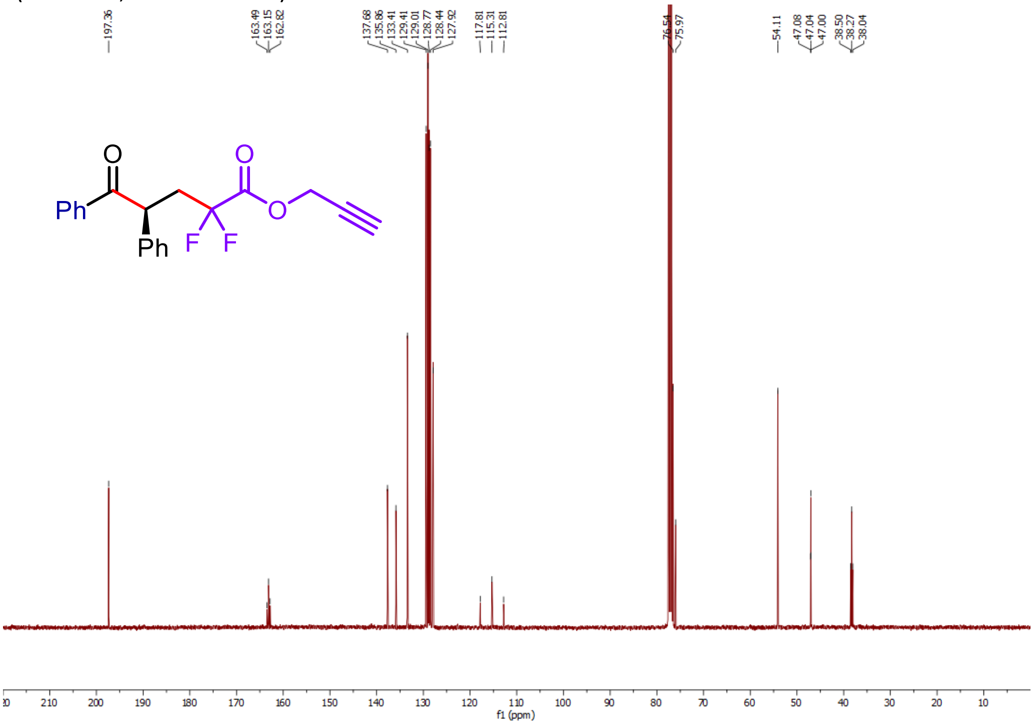

^{19}F NMR (376 MHz, Chloroform-*d*):

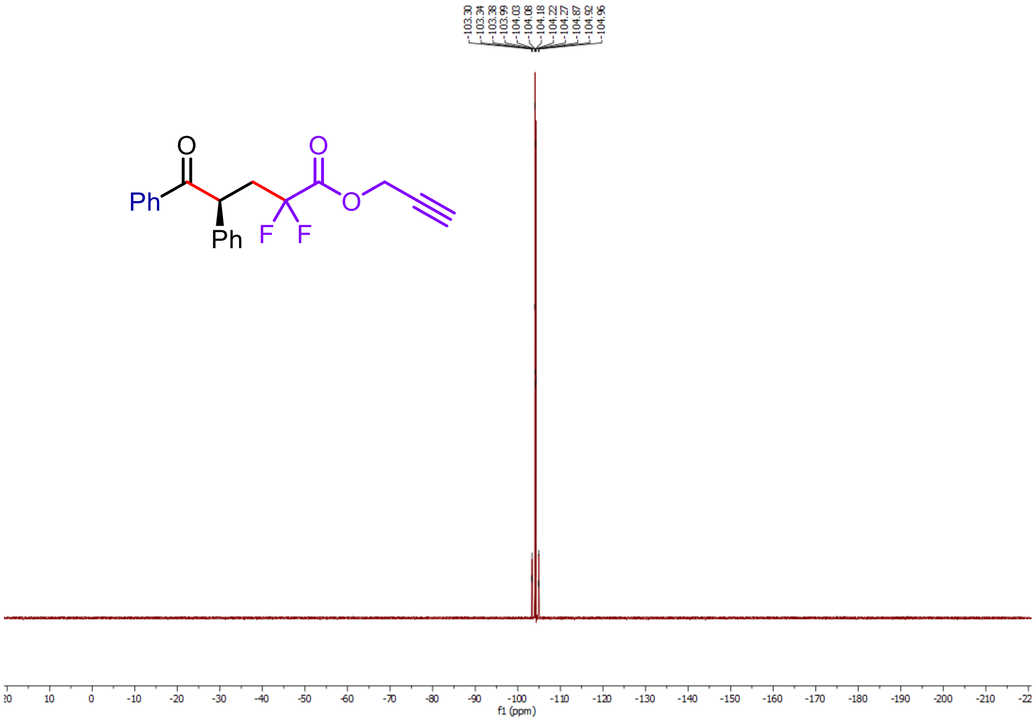

(*S*)-*N*,*N*-diethyl-2,2-difluoro-5-oxo-4,5-diphenylpentanamide (7e)

^{1}H NMR (400 MHz, $CDCl_3$):

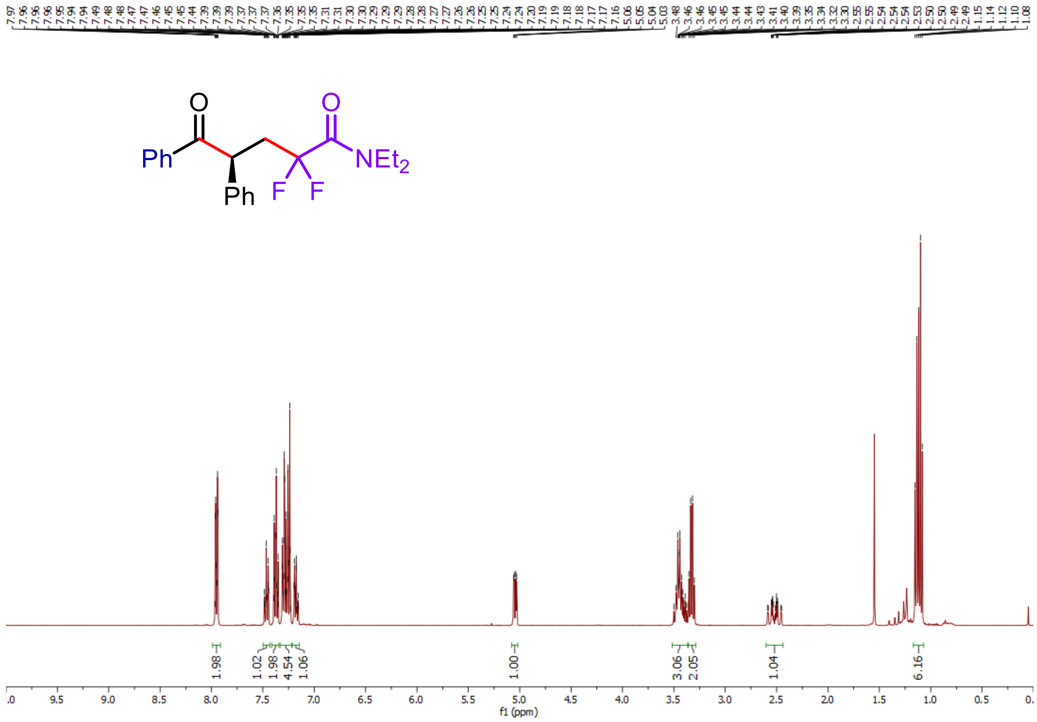

^{13}C NMR (101 MHz, $CDCl_3$):

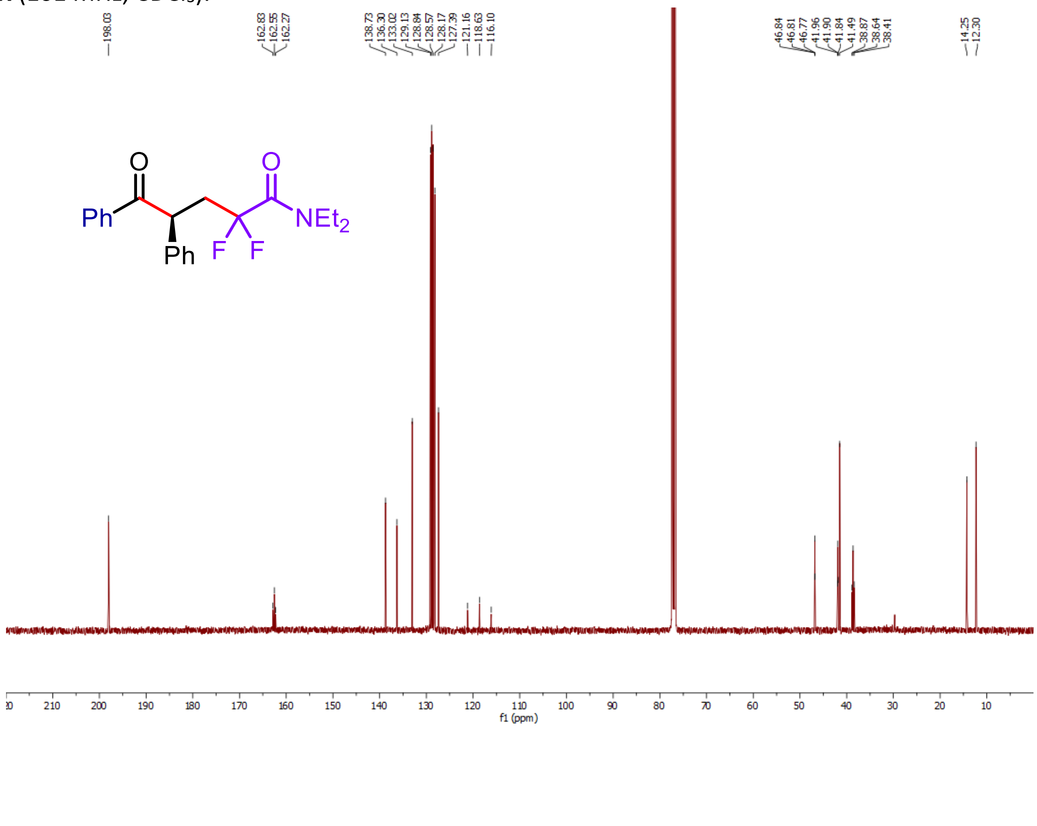

^{19}F NMR (376 MHz, $CDCl_3$):

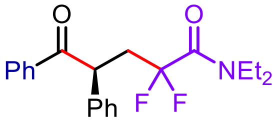

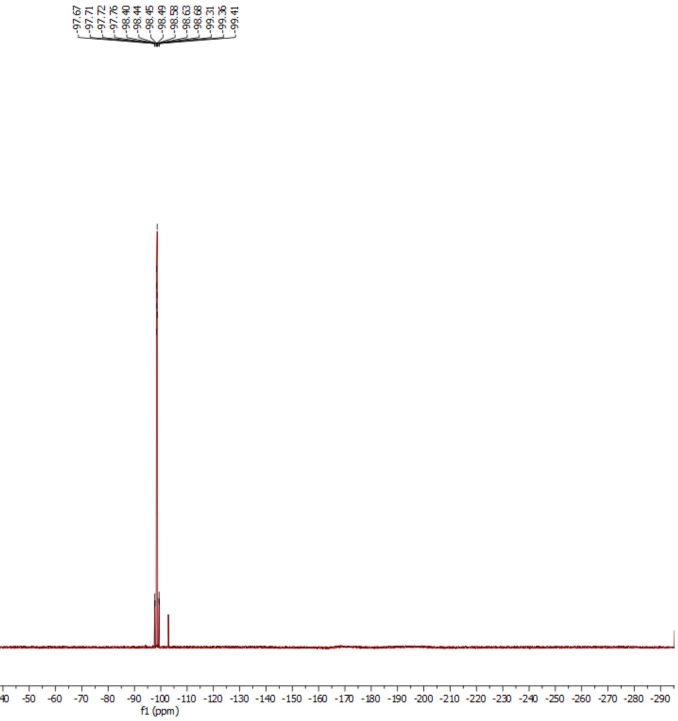

(*S*)-2,2-Difluoro-1-morpholino-4,5-diphenylpentane-1,5-dione (7f)

^{1}H NMR (400 MHz, Chloroform-*d*):

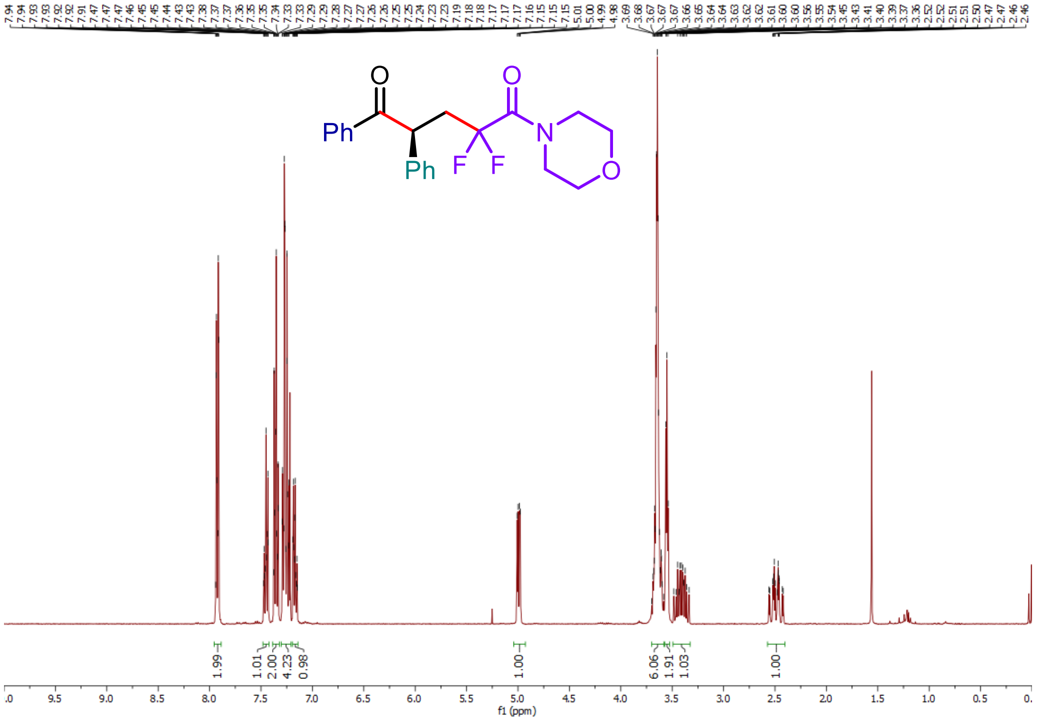

^{13}C NMR (101 MHz, Chloroform-*d*):

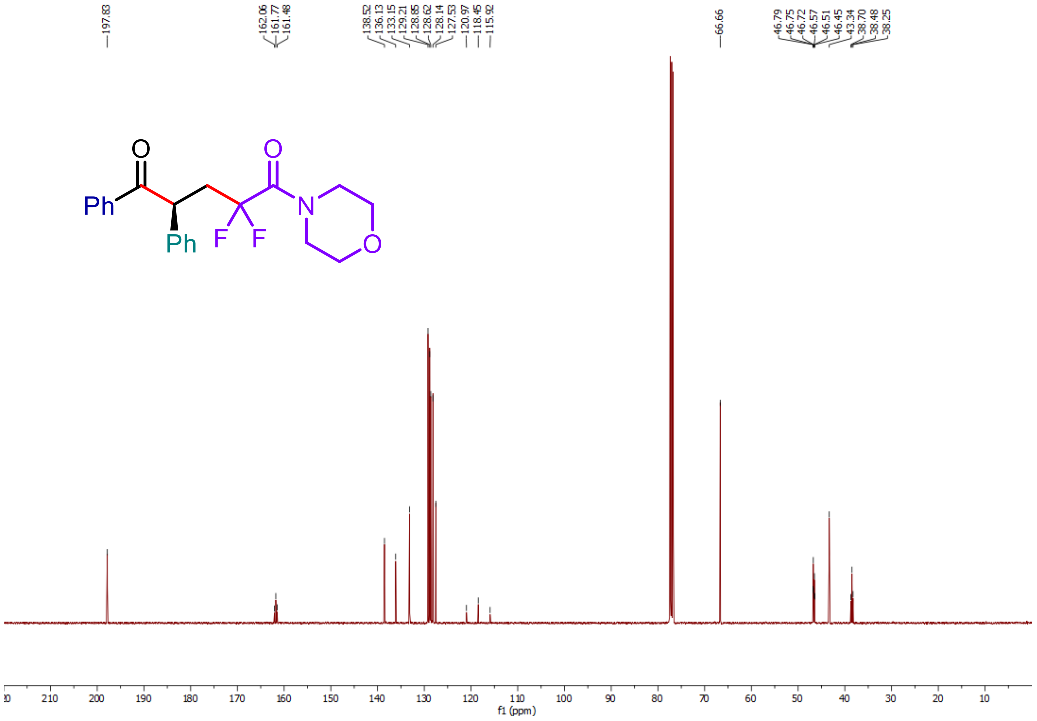

^{19}F NMR (376 MHz, Chloroform-*d*):

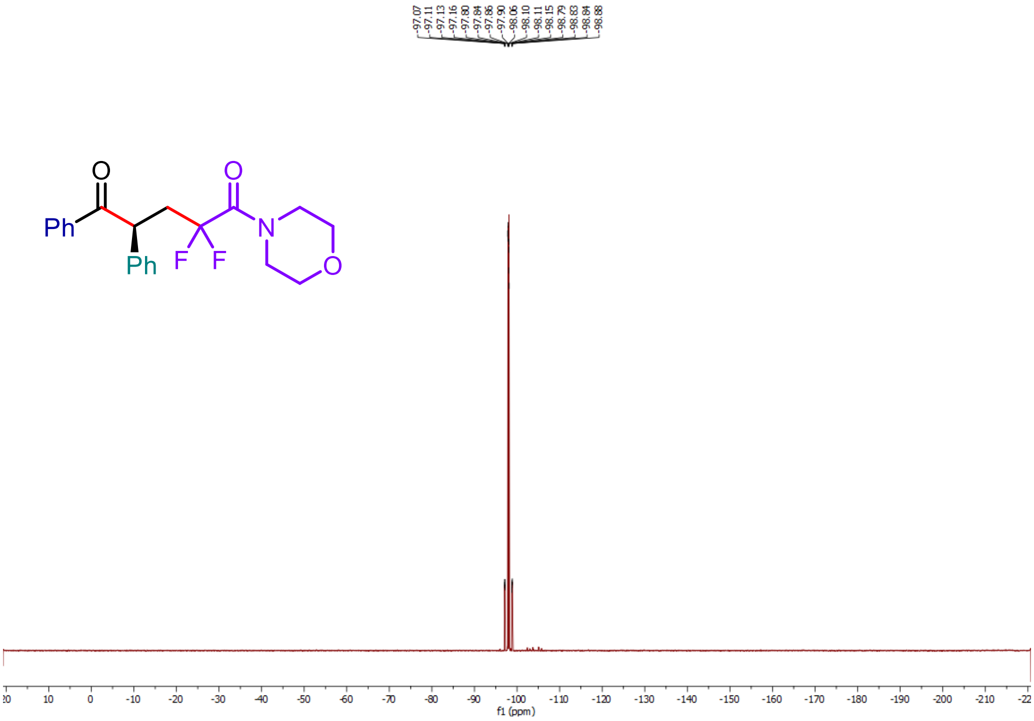

(*S*)-*N*-(*tert*-butyl)-2,2-difluoro-5-oxo-4,5-diphenylpentanamide (7g)

^{1}H NMR (400 MHz, Chloroform-*d*):

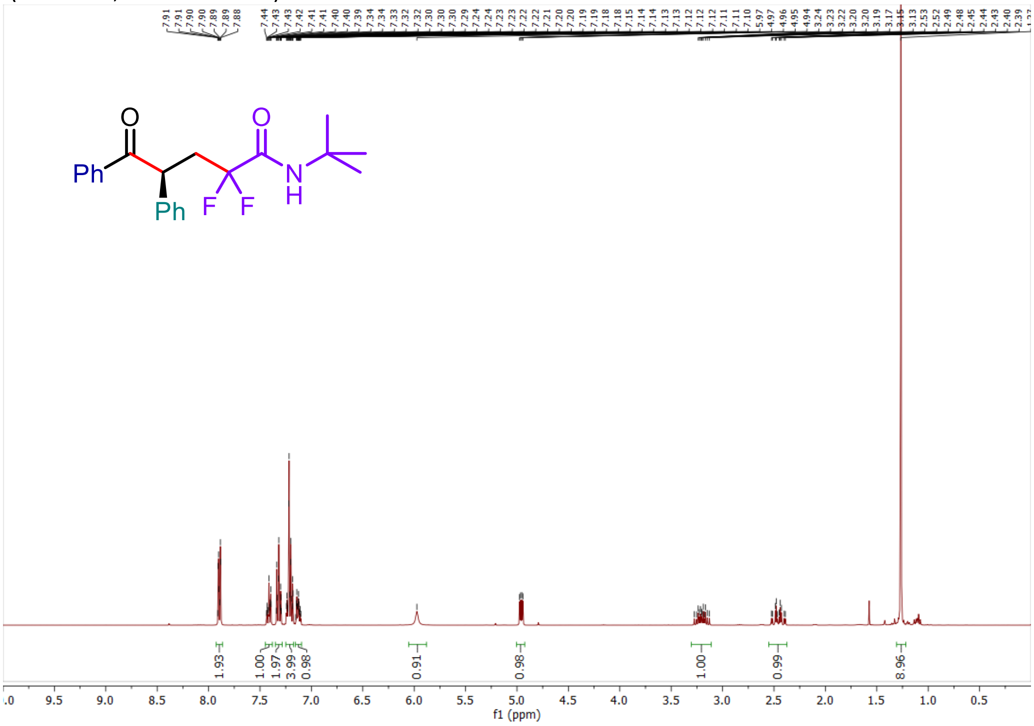

^{13}C NMR (101 MHz, Chloroform-*d*):

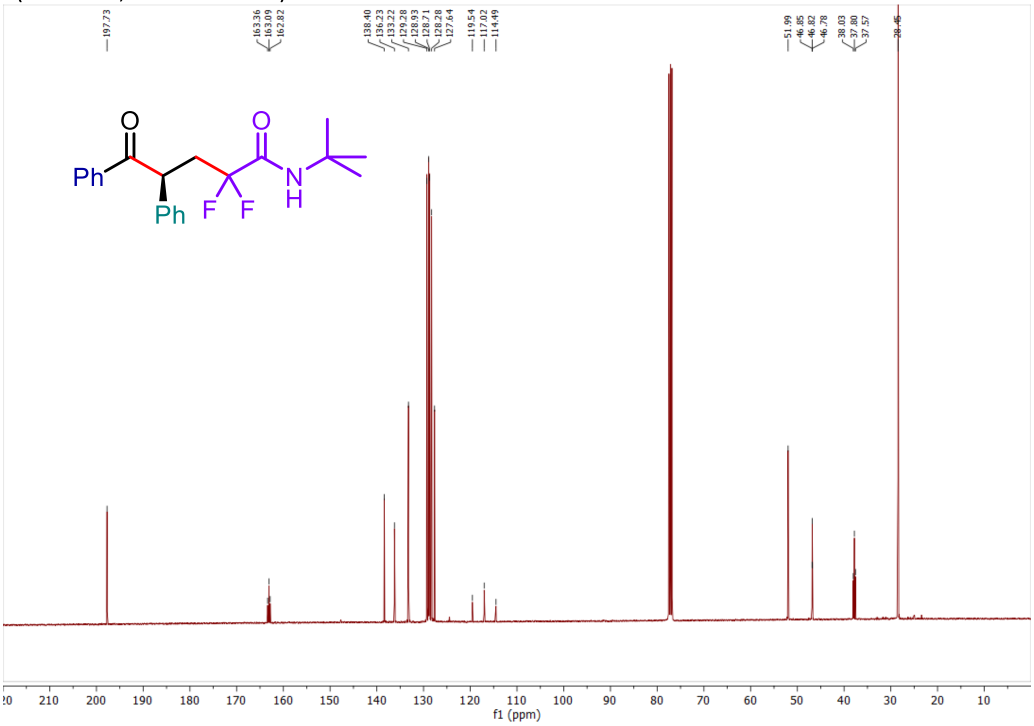

^{19}F NMR (376 MHz, Chloroform-*d*):

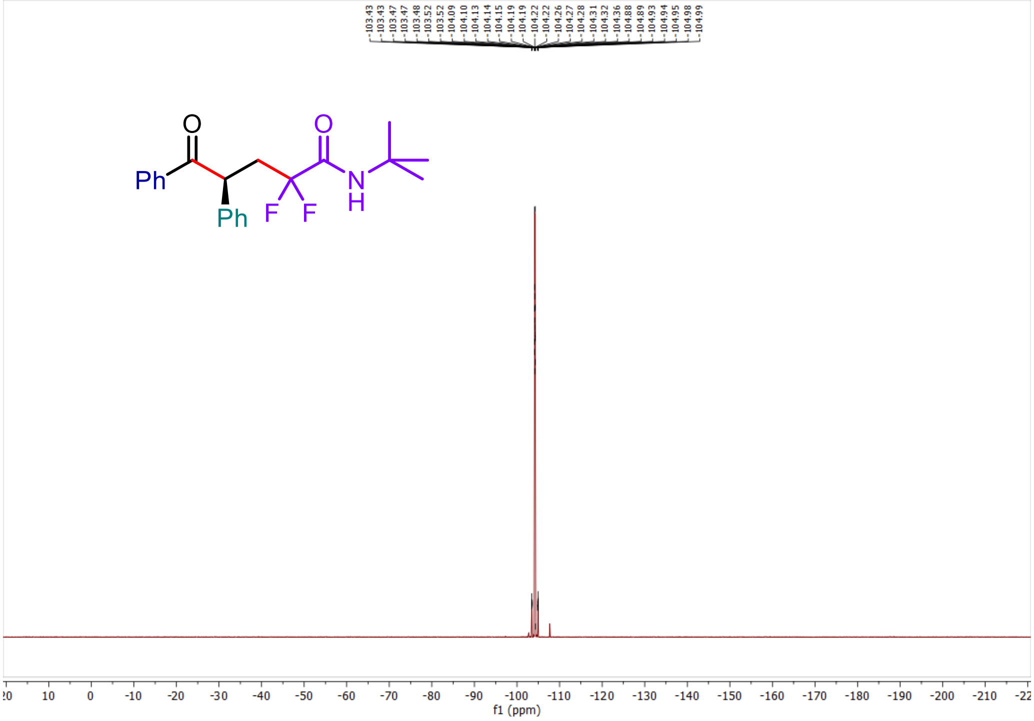

(*S*)-2,2-Difluoro-1-(isoindolin-2-yl)-4,5-diphenylpentane-1,5-dione (7h)

^{1}H NMR (400 MHz, Chloroform-*d*):

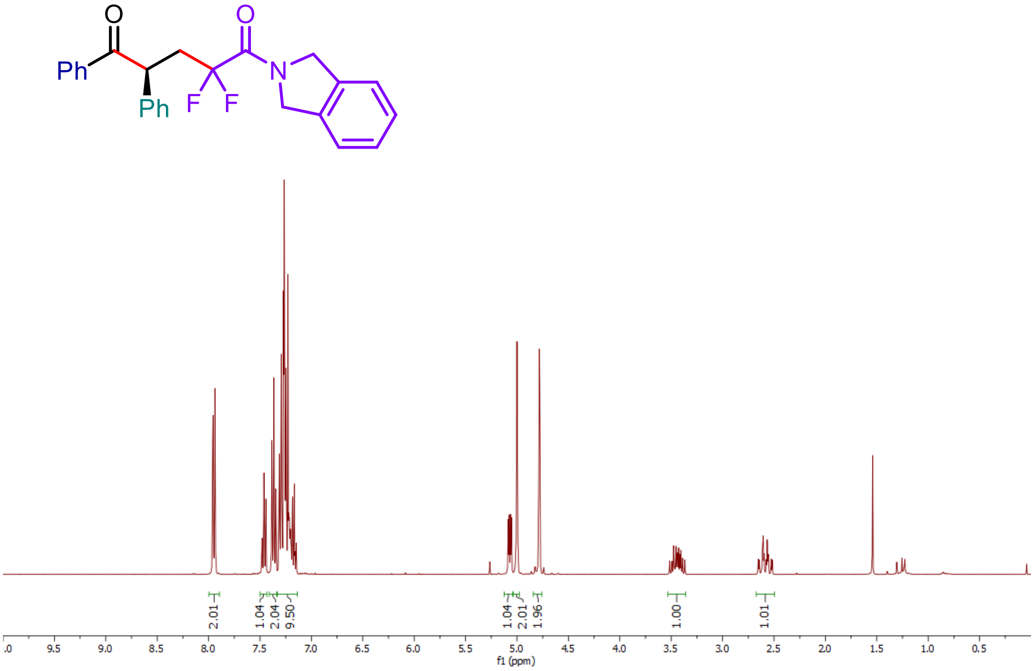

^{13}C NMR (101 MHz, Chloroform-*d*):

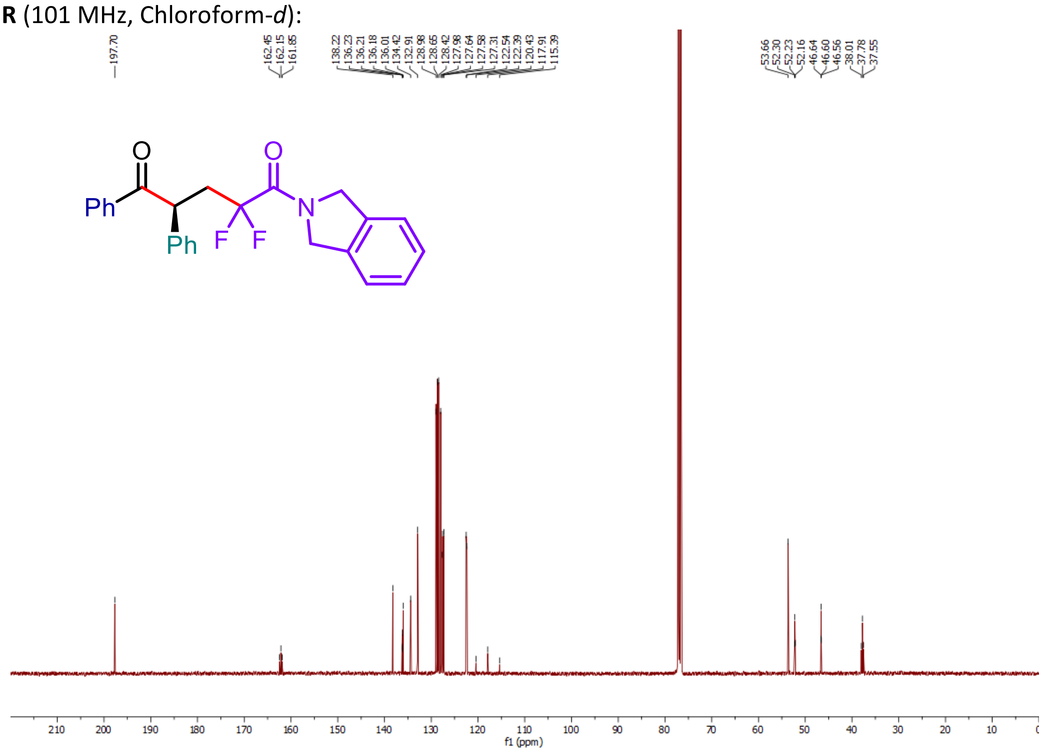

^{19}F NMR (376 MHz, $CDCl_3$):

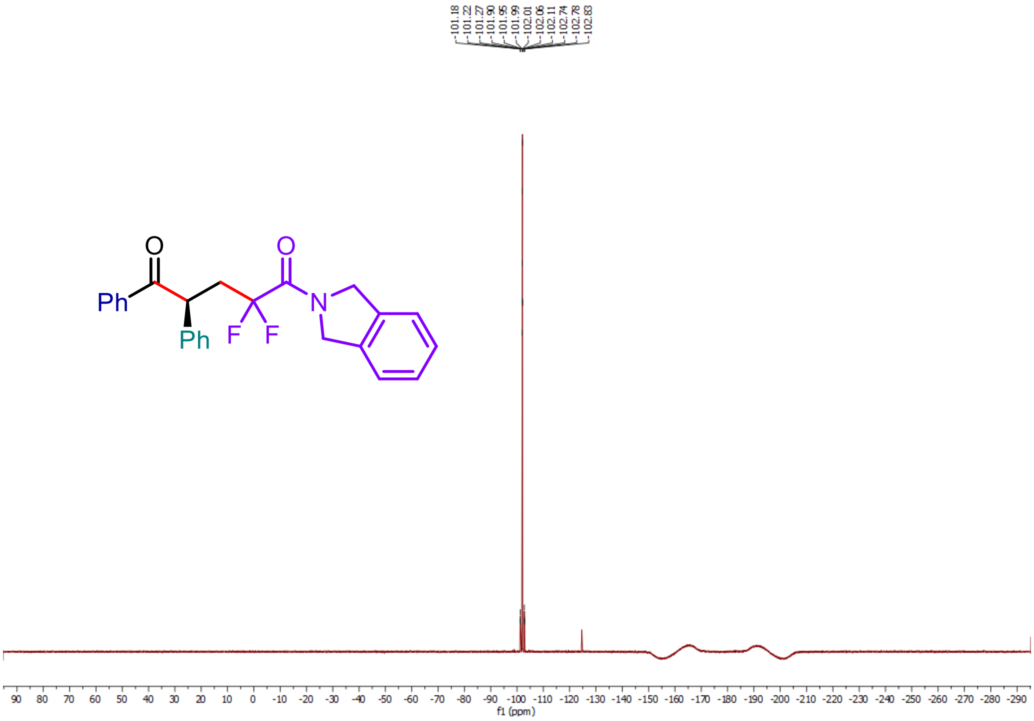

(*S*)-4,4-Difluoro-1,2-diphenyl-4-(phenylsulfonyl)butan-1-one (7i)

^{1}H NMR (400 MHz, $CDCl_3$):

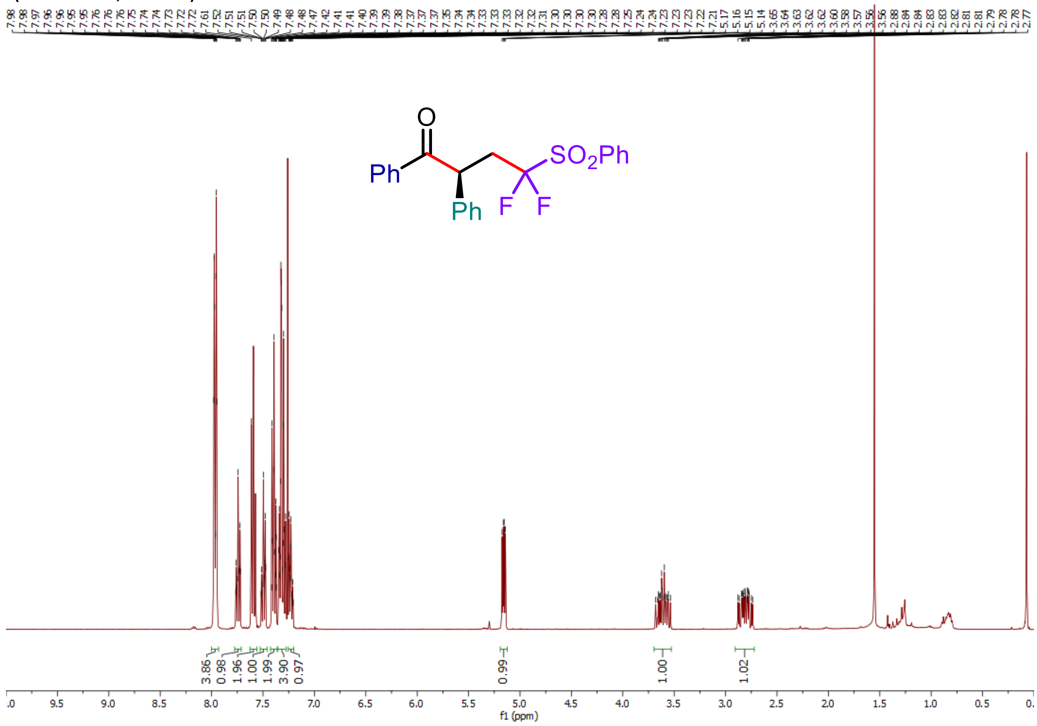

^{13}C NMR (101 MHz, $CDCl_3$):

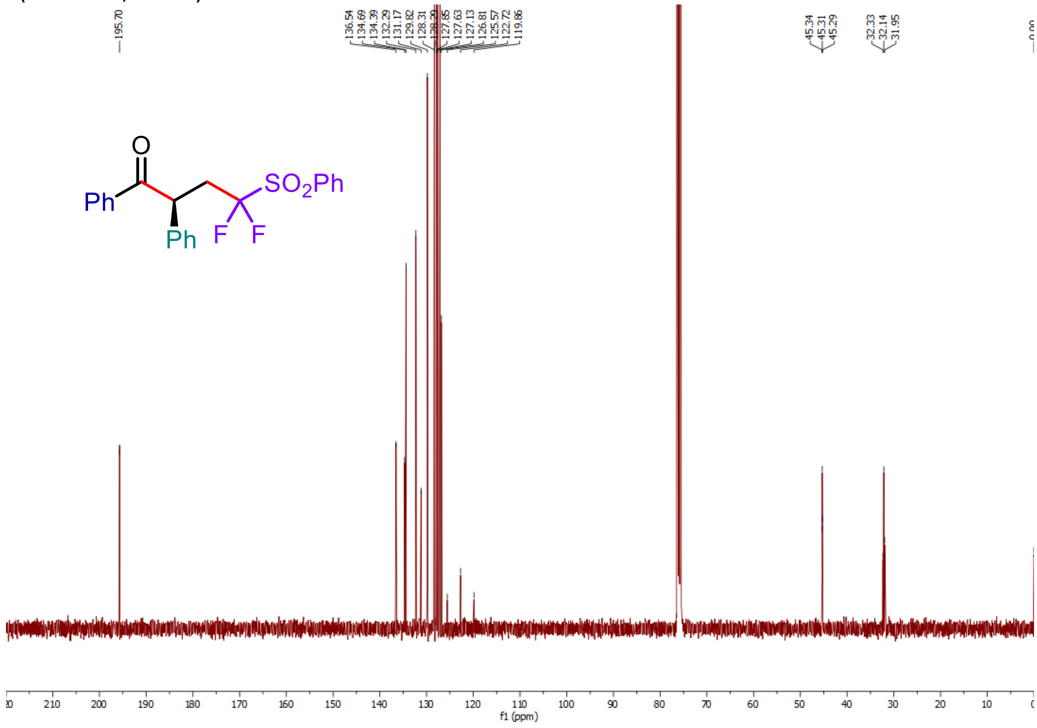

^{19}F NMR (376 MHz, $CDCl_3$):

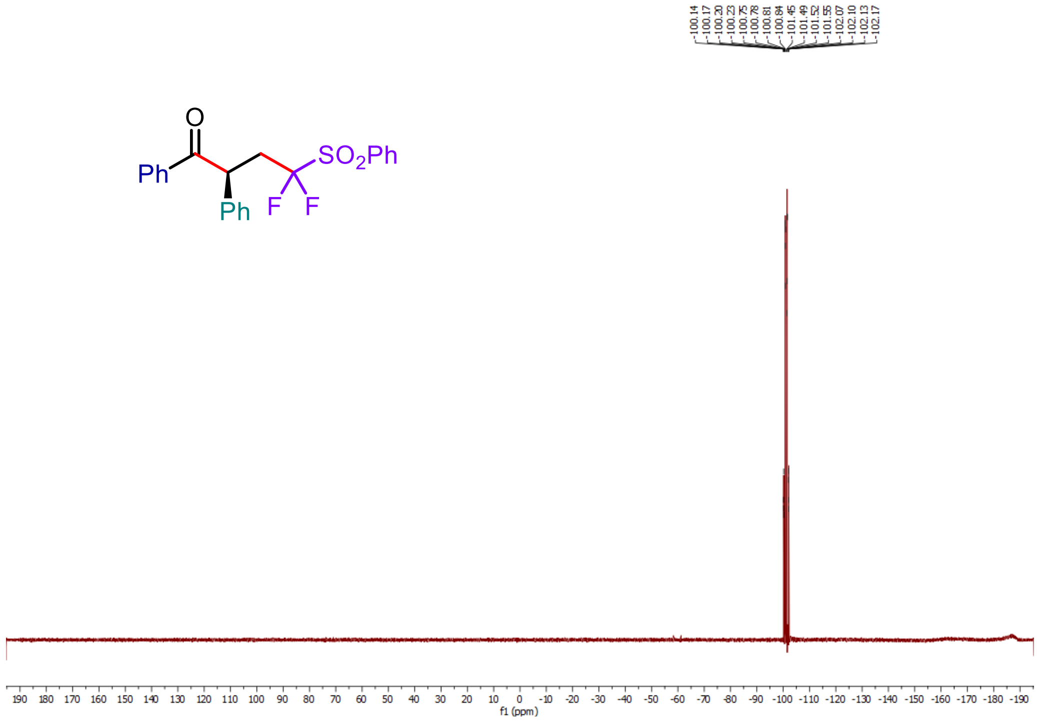

(*S*)-4-Bromo-4,4-difluoro-1,2-diphenylbutan-1-one (7j)

^{1}H NMR (400 MHz, Chloroform-*d*):

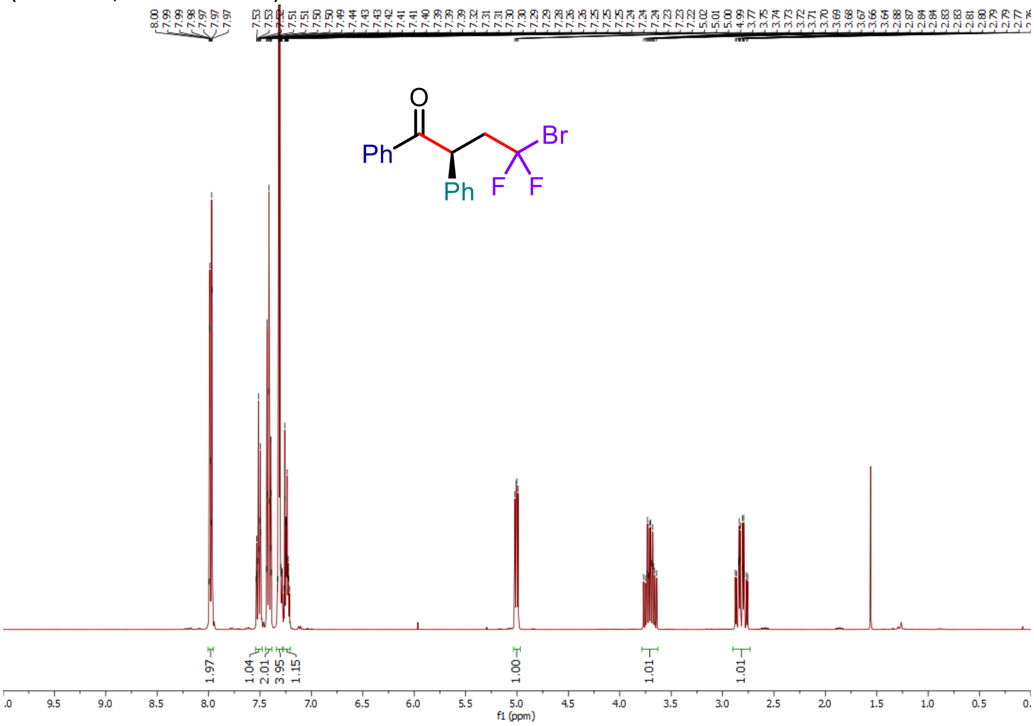

^{13}C NMR (101 MHz, Chloroform-*d*):

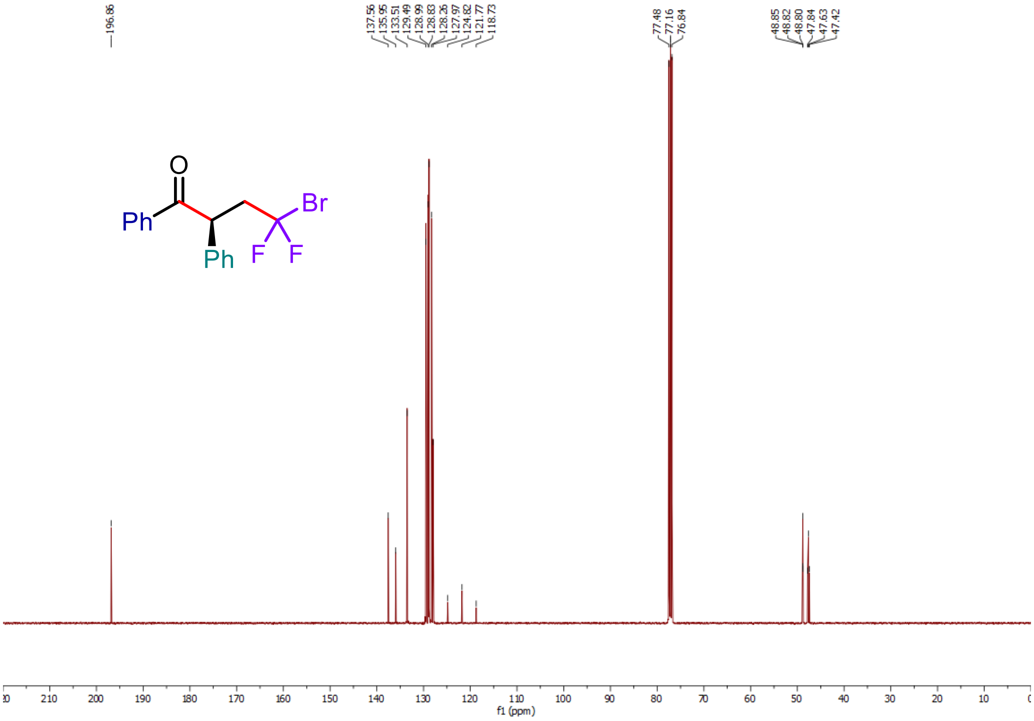

^{19}F NMR (377 MHz, Chloroform-*d*):

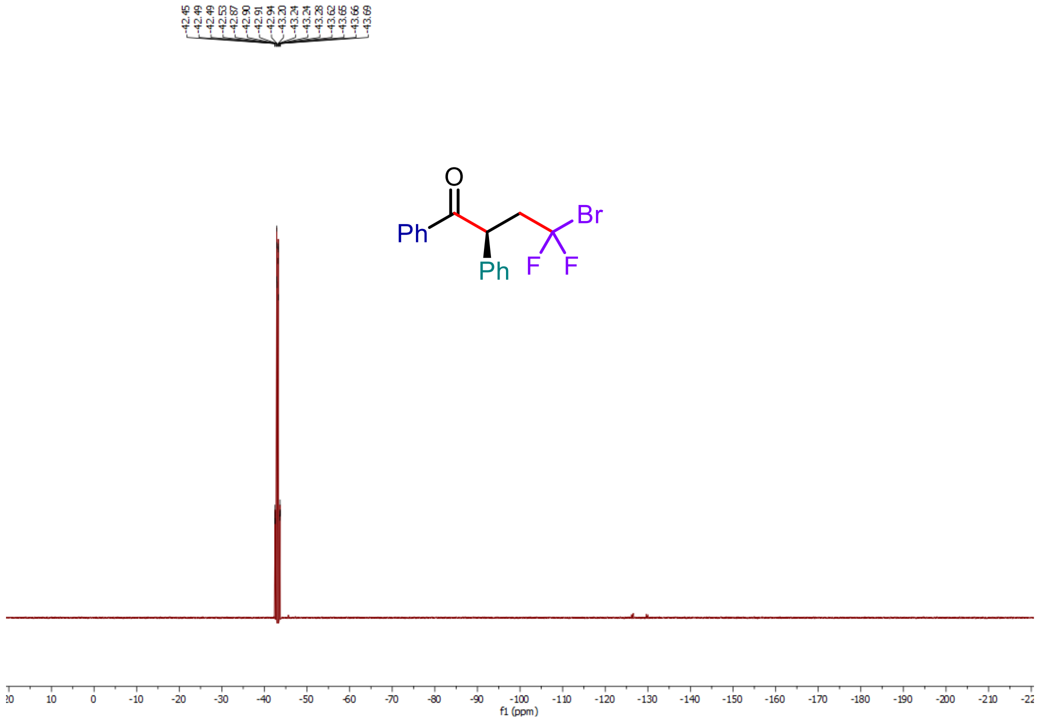

(*S*)-4,4,4-Trifluoro-1,2-diphenylbutan-1-one (7k)

^{1}H NMR (400 MHz, Chloroform-*d*):

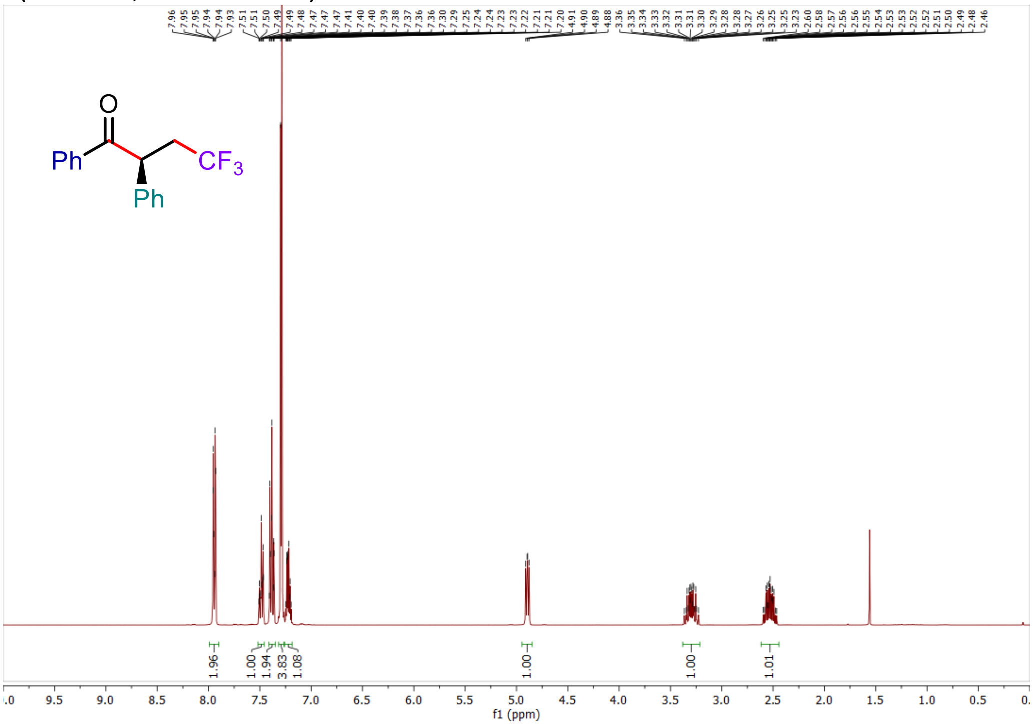

^{13}C NMR (101 MHz, Chloroform-*d*):

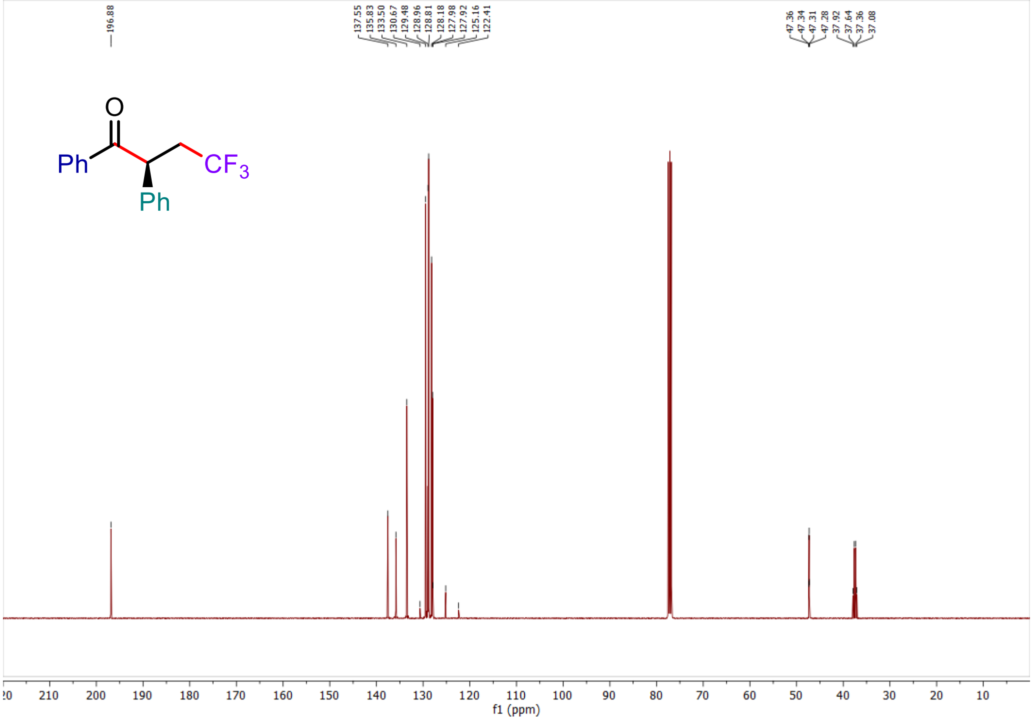

^{19}F NMR (376 MHz, Chloroform-*d*):

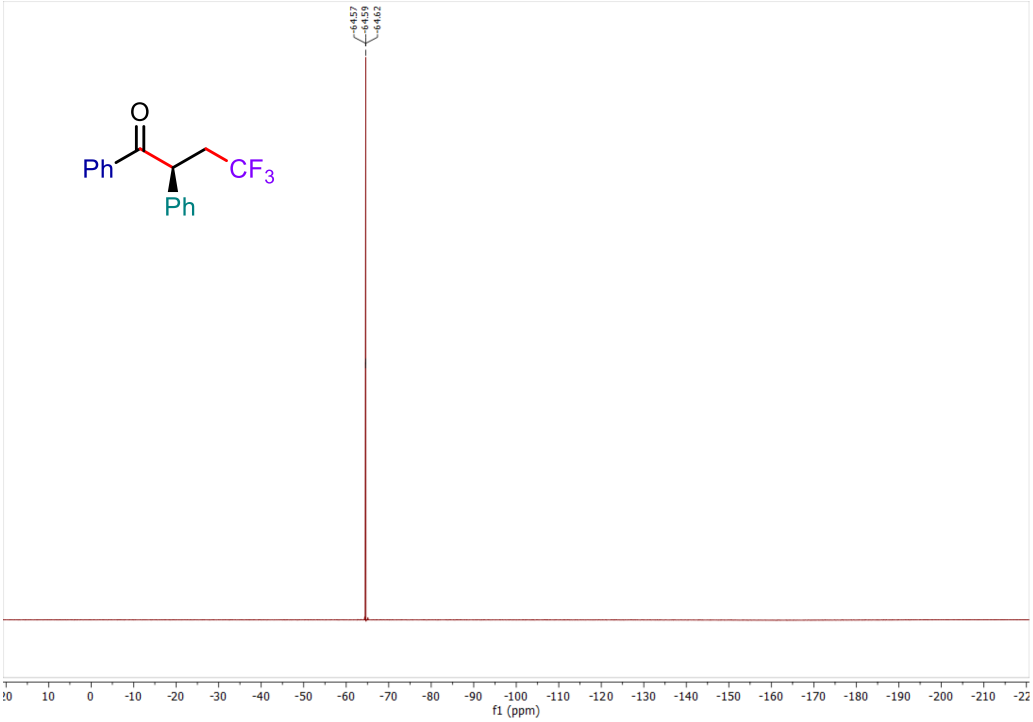

(*S*)-4,4,5,5,6,6,7,7,7-Nonafluoro-1,2-diphenylheptan-1-one (7l)

^{1}H NMR (400 MHz, Chloroform-*d*):

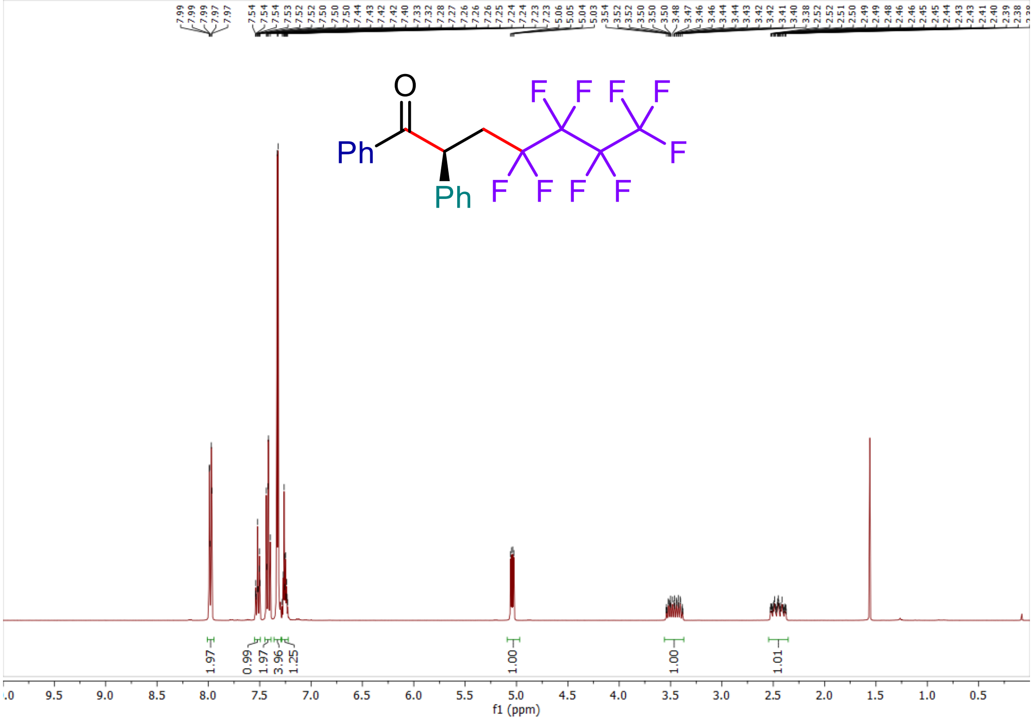

^{13}C NMR (101 MHz, Chloroform-*d*):

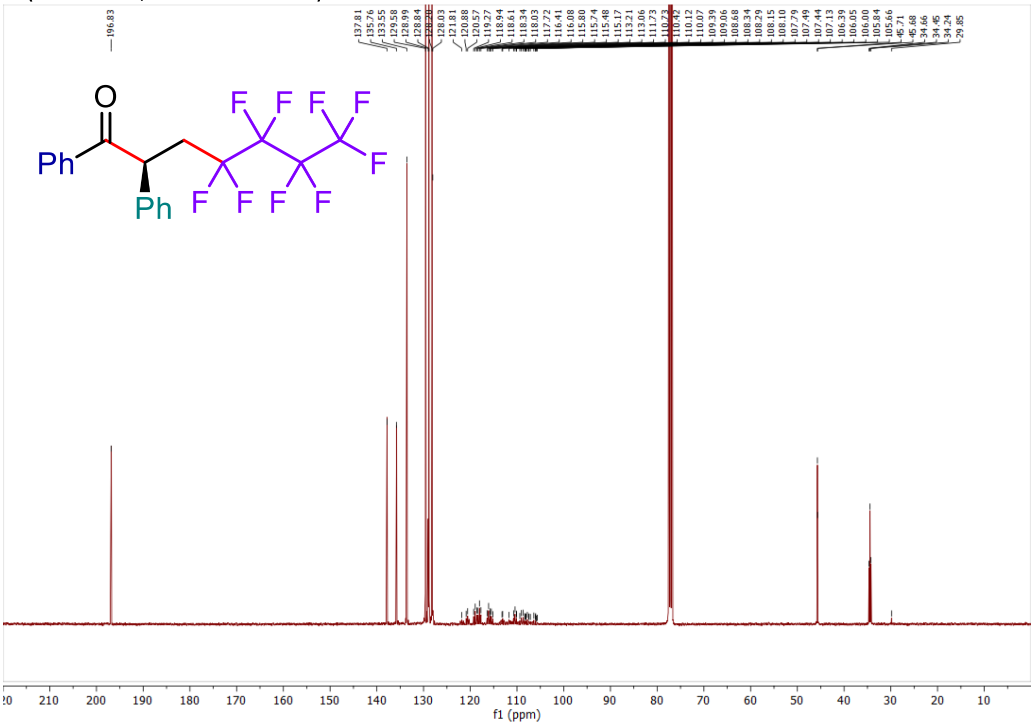

19F NMR (376 MHz, Chloroform-*d*):

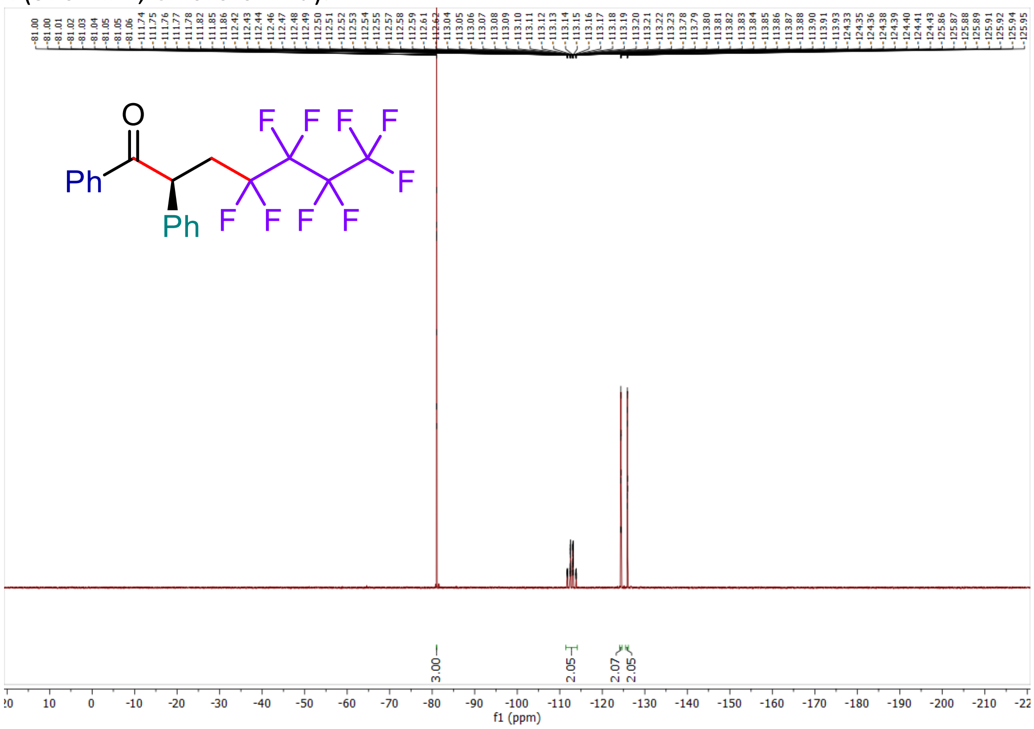

(*S*)-4,4,4-trichloro-1,2-diphenylbutan-1-one (7m)

^{1}H NMR (400 MHz, Chloroform-*d*):

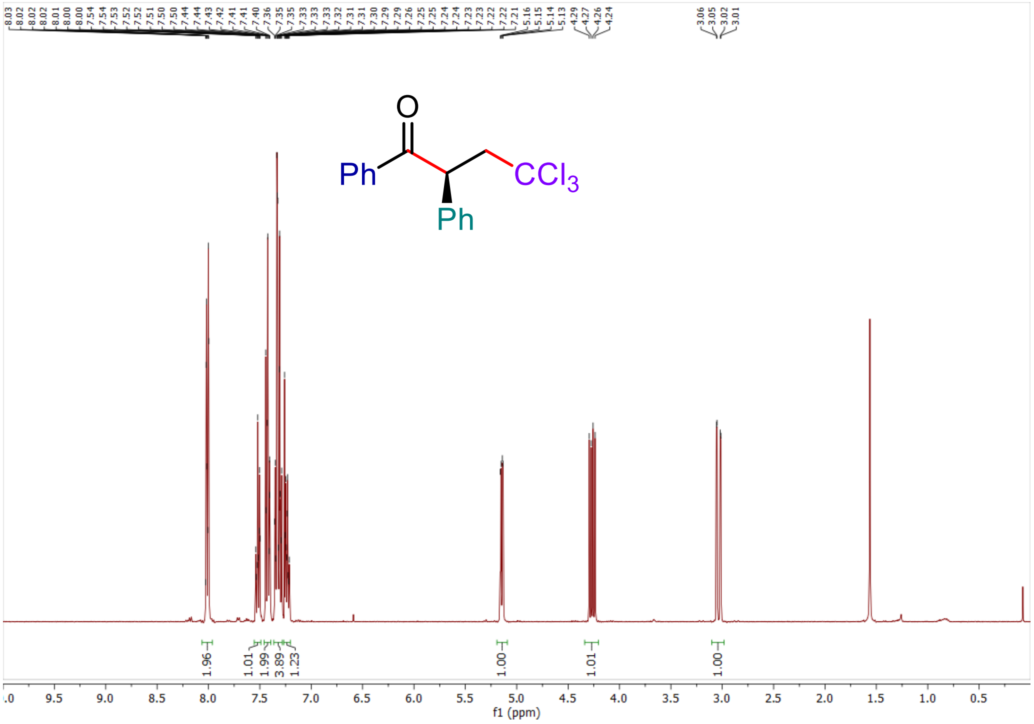

^{13}C NMR (101 MHz, Chloroform-*d*):

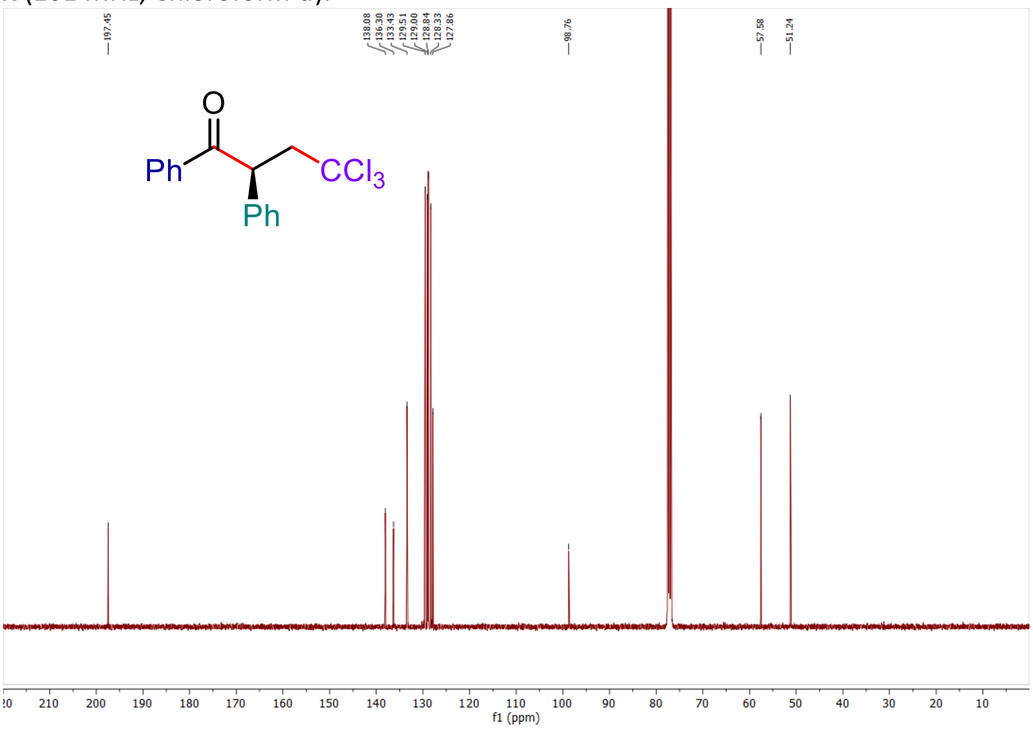

Ethyl (*S*)-2,2-difluoro-4-(4-(4-((1-isopropoxy-2-methyl-1-oxopropan-2-yl)oxy)benzoyl)phenyl)-5-oxo-5-phenylpentanoate (8a)

^{1}H NMR (400 MHz, Chloroform-*d*):

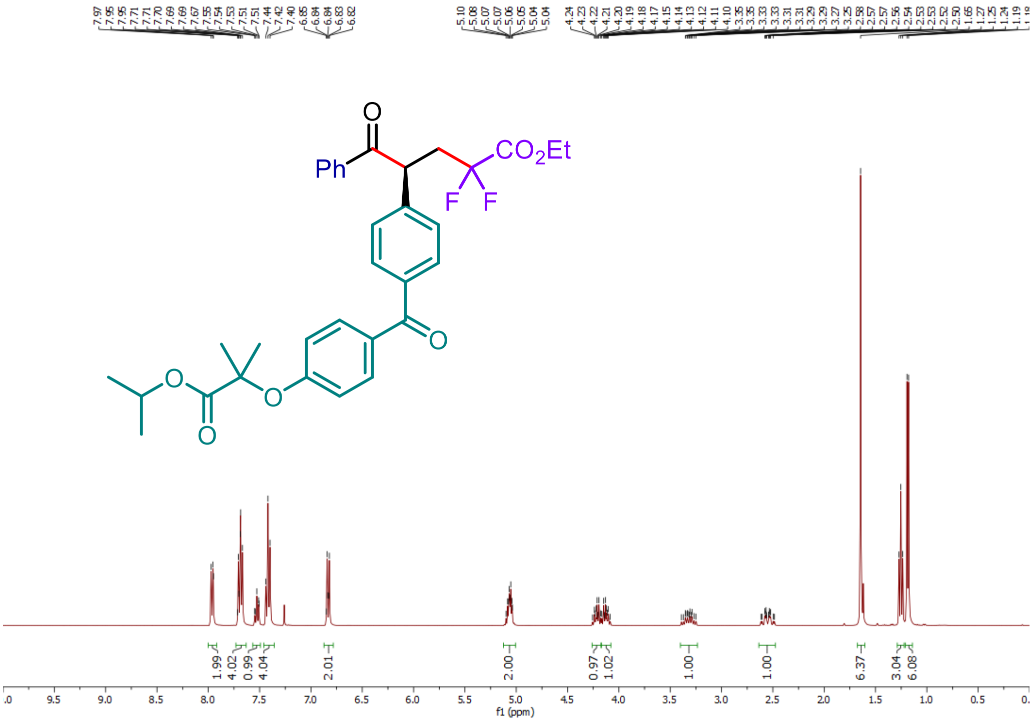

^{13}C NMR (101 MHz, Chloroform-*d*):

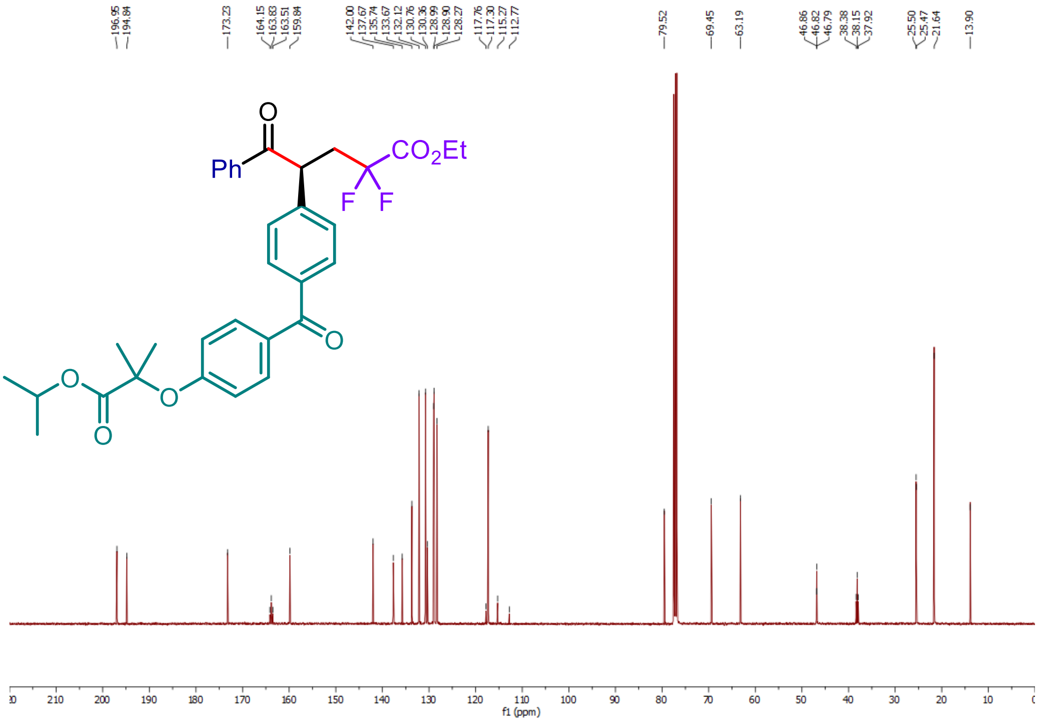

^{19}F NMR (376 MHz, Chloroform-*d*):

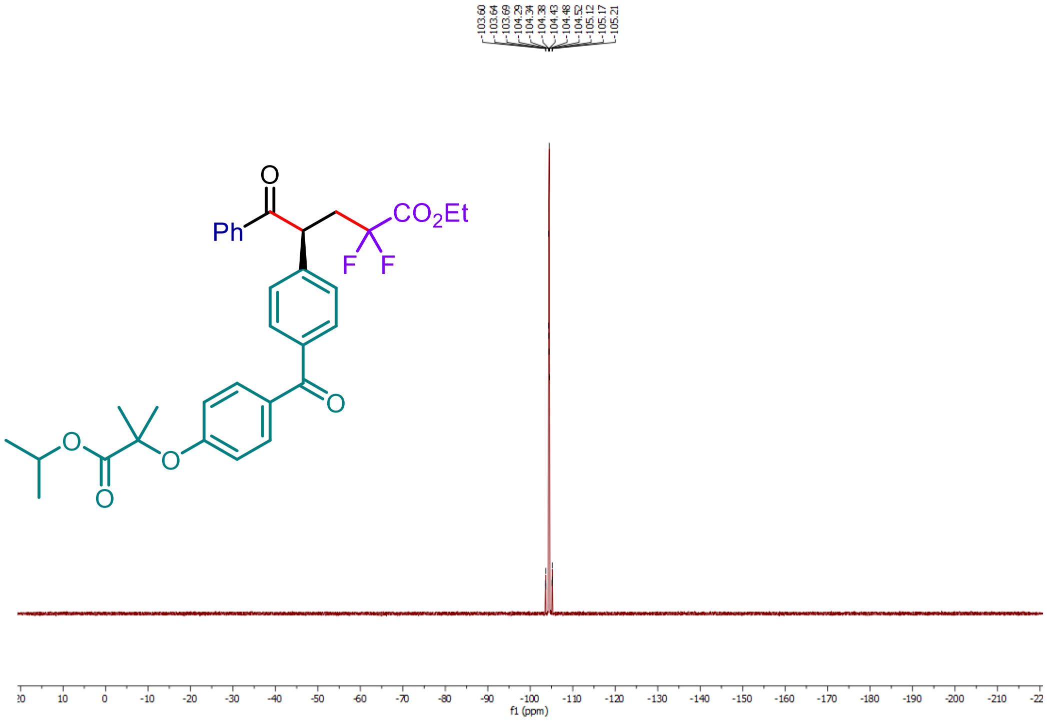

Ethyl (*S*)-2,2-difluoro-4-((8*R*,9*S*,13*S*,14*S*)-13-methyl-17-oxo-7,8,9,11,12,13,14,15,16,17-decahydro-6*H*-cyclopenta[*a*]phenanthren-3-yl)-5-oxo-5-phenylpentanoate (8b)

^{1}H NMR (400 MHz, Chloroform-*d*):

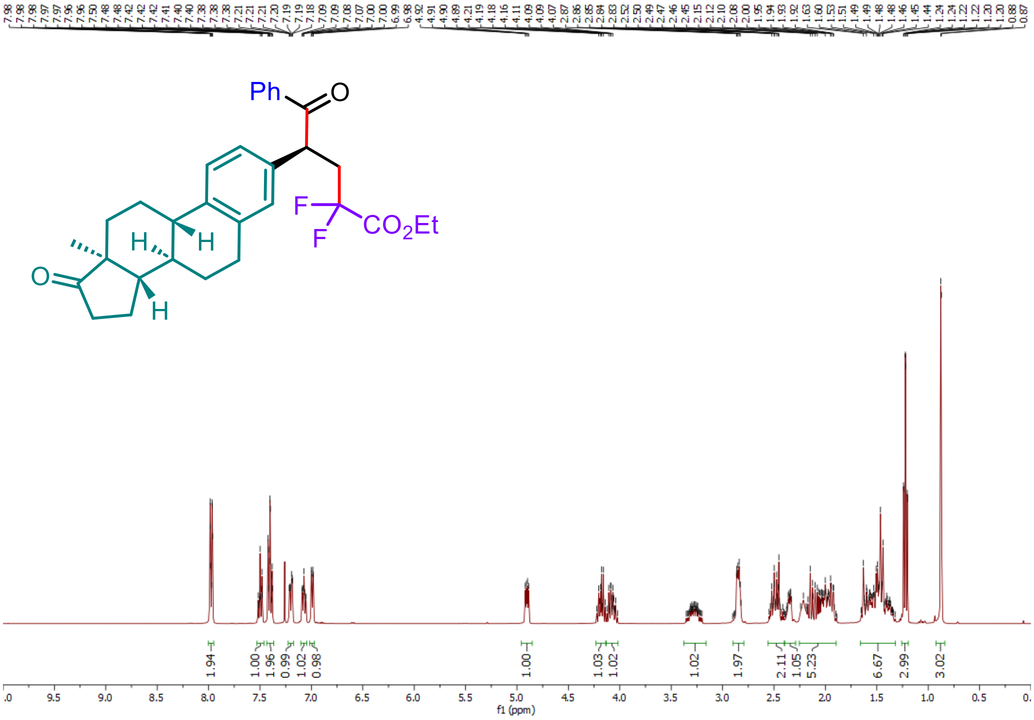

^{13}C NMR (101 MHz, Chloroform-*d*):

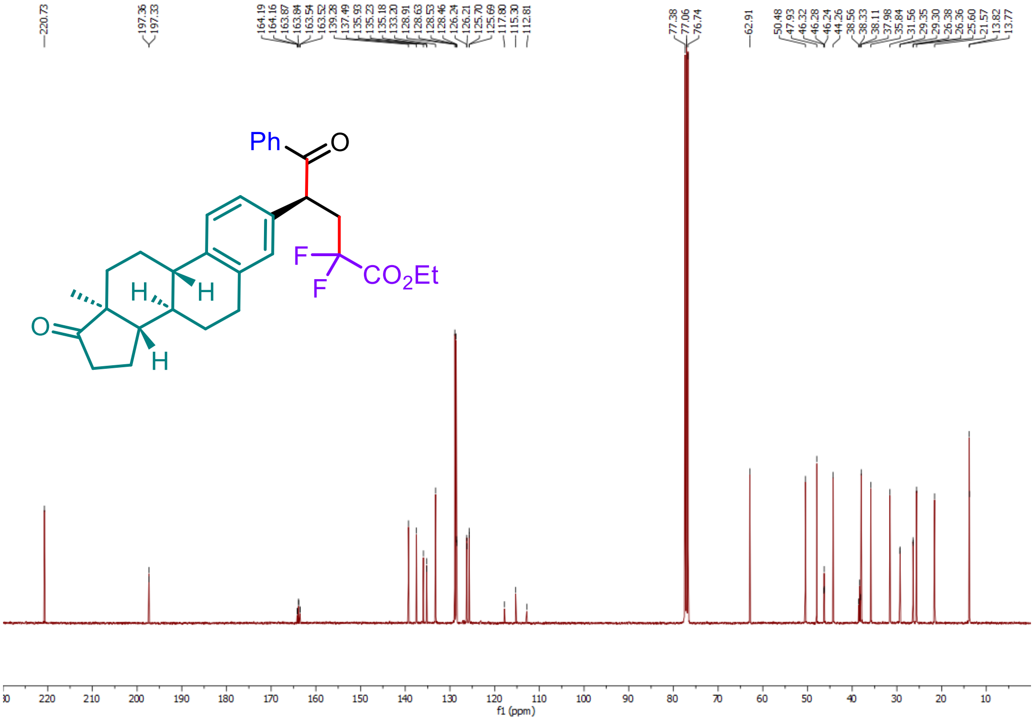

[19]**F NMR** (376 MHz, Chloroform-*d*):

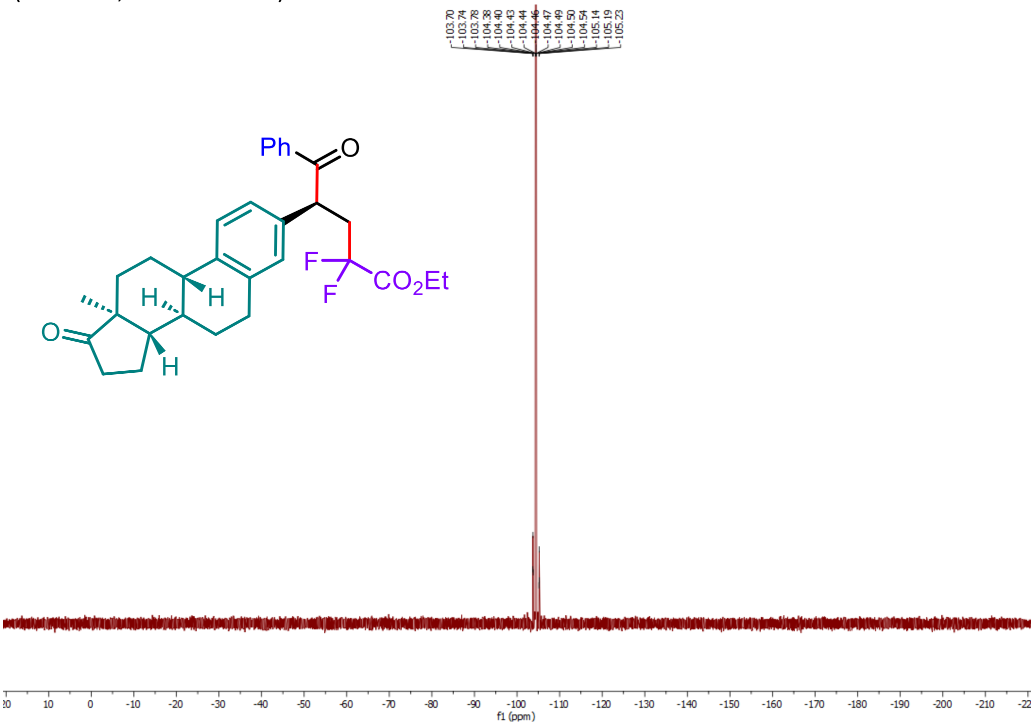

Ethyl (*S*)-4-((*R*)-2,8-dimethyl-2-((4*R*,8*R*)-4,8,12-trimethyltridecyl)chroman-6-yl)-2,2-difluoro-5-oxo-5-phenylpentanoate (8c)

^{1}H NMR (400 MHz, Chloroform-*d*):

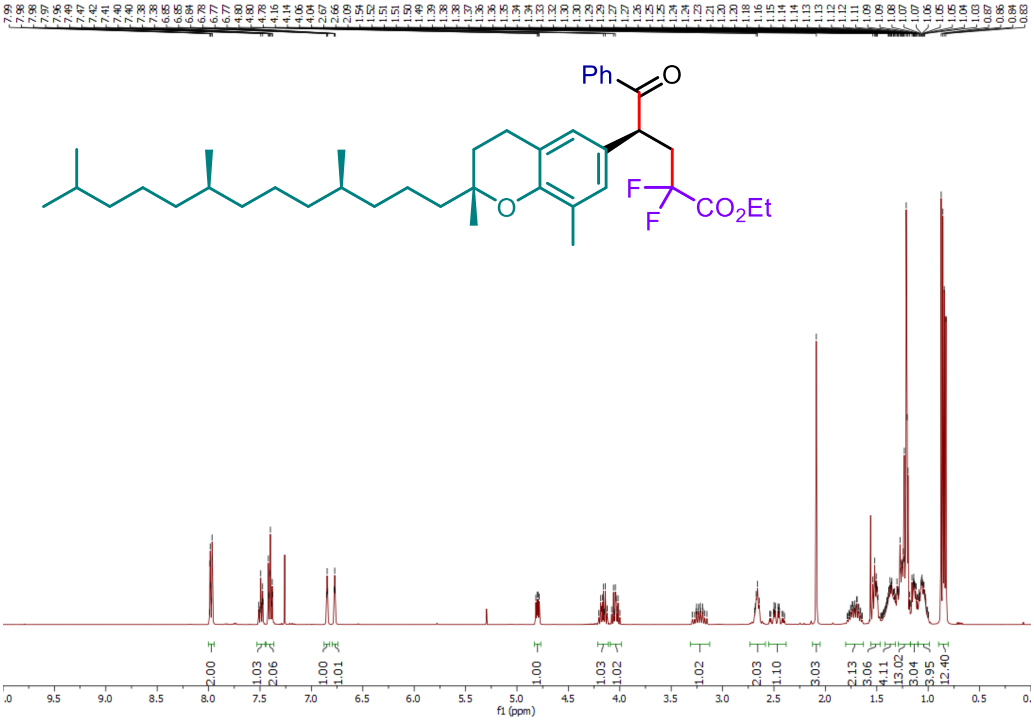

^{13}C NMR (101 MHz, Chloroform-*d*):

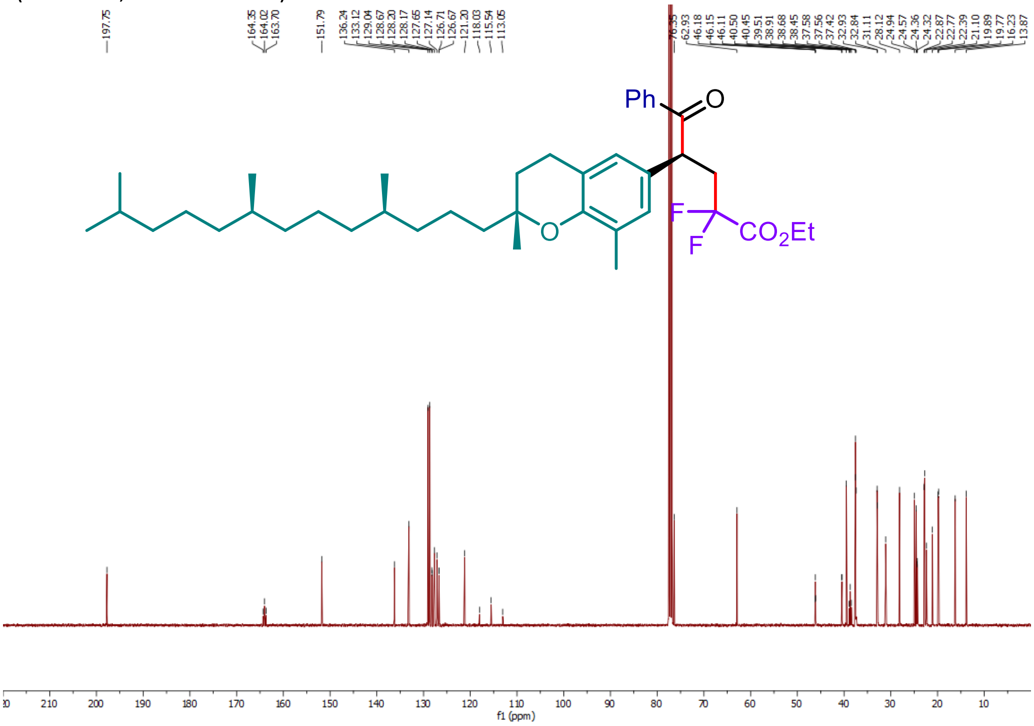

^{19}F NMR (376 MHz, Chloroform-*d*):

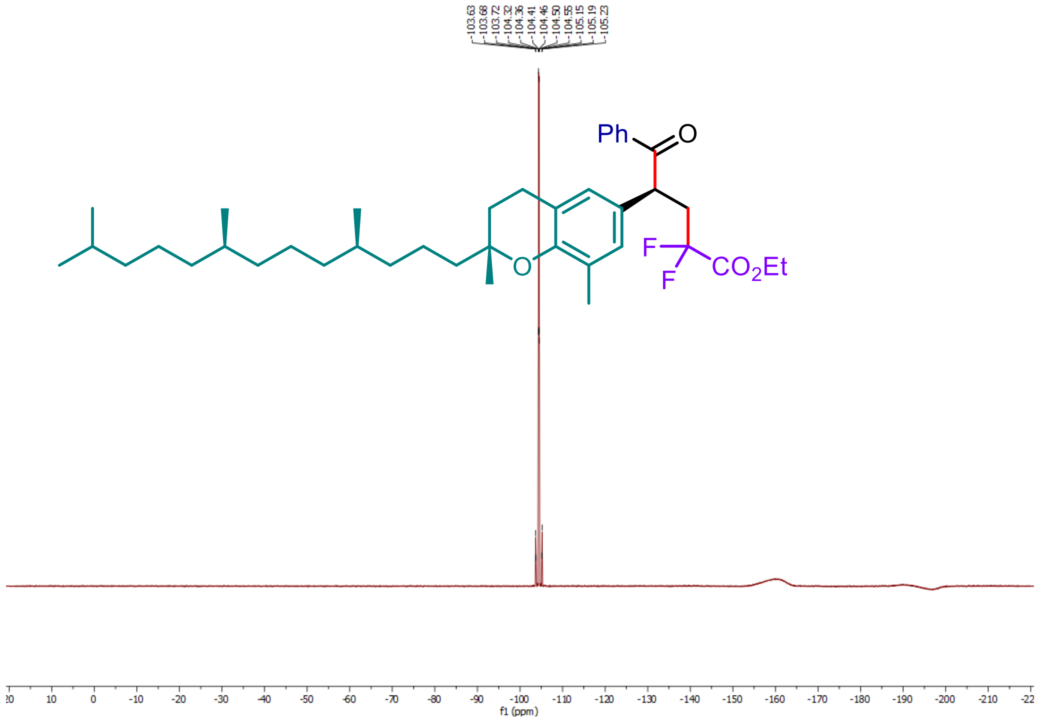

(3*S*,8*S*,9*S*,10*R*,13*R*,14*S*,17*R*)-10,13-dimethyl-17-((*R*)-6-methylheptan-2-yl)-2,3,4,7,8,9,10,11,12,13,14,15,16,17-tetradecahydro-1*H*-cyclopenta[a]phenanthren-3-yl (*S*)-2,2-difluoro-5-oxo-4,5-diphenylpentanoate (9a)

^{1}H NMR (400 MHz, Chloroform-*d*):

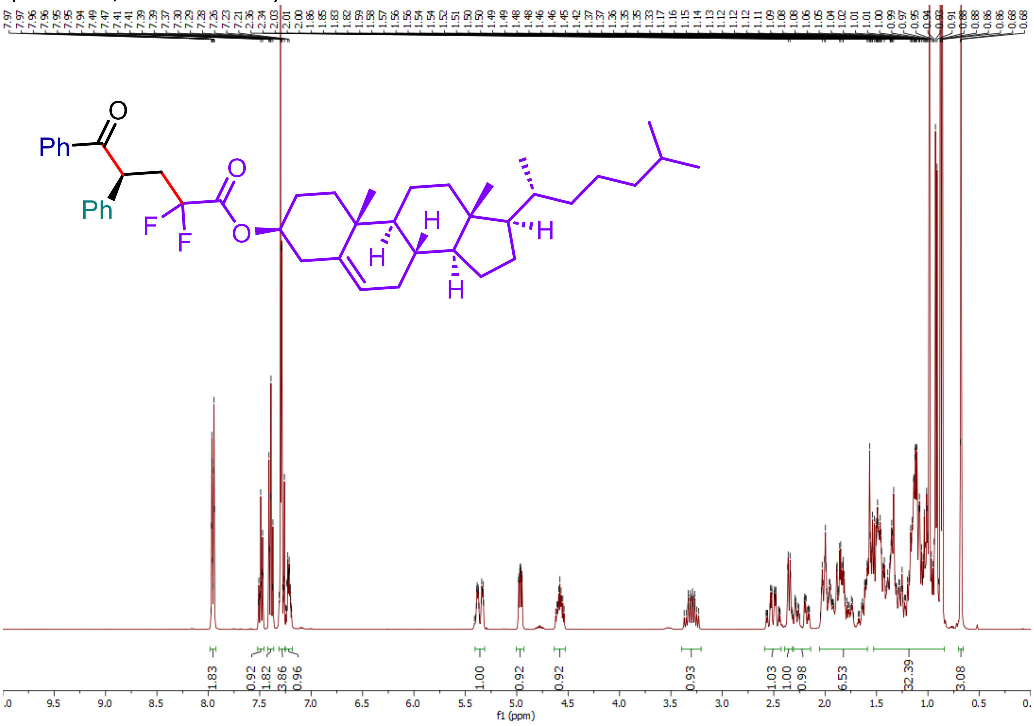

^{13}C NMR (101 MHz, Chloroform-*d*):

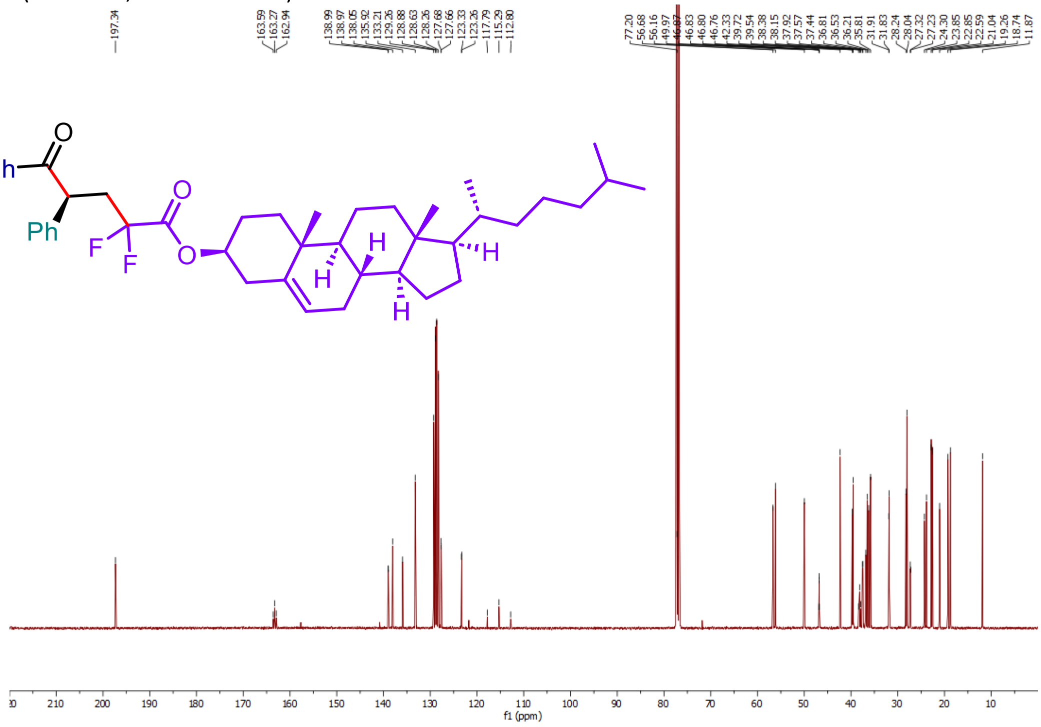

^{19}F NMR (376 MHz, Chloroform-*d*):

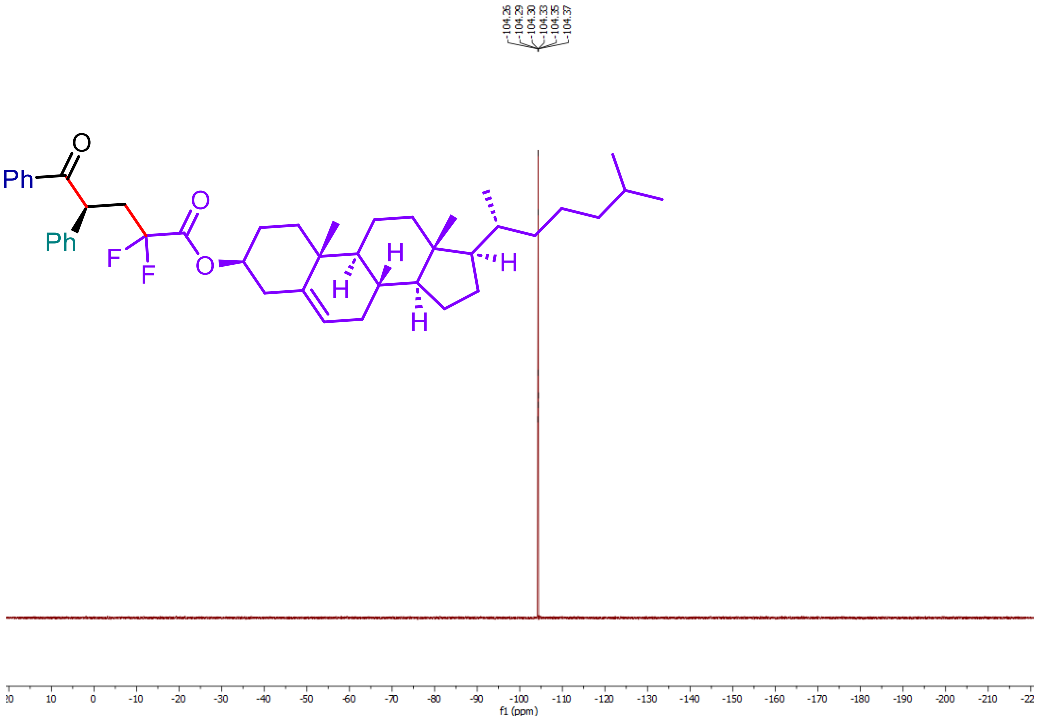

(*E*)-3,7-dimethylocta-2,6-dien-1-yl (*S*)-2,2-difluoro-5-oxo-4,5-diphenylpentanoate (9b)

^{1}H NMR (400 MHz, Chloroform-*d*):

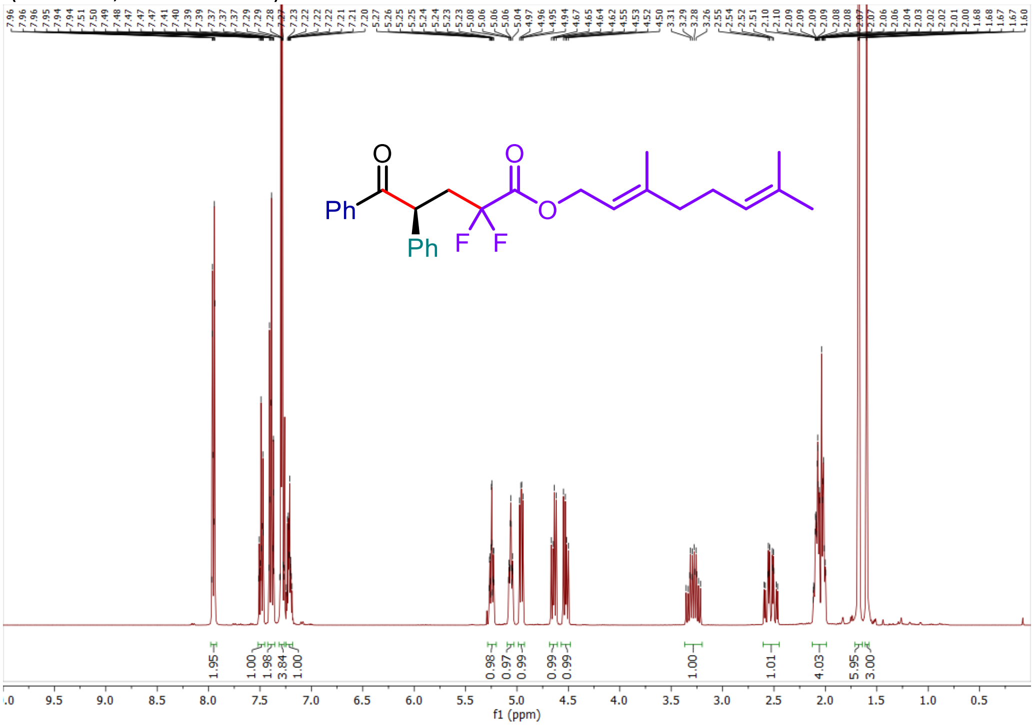

^{13}C NMR (101 MHz, Chloroform-*d*):

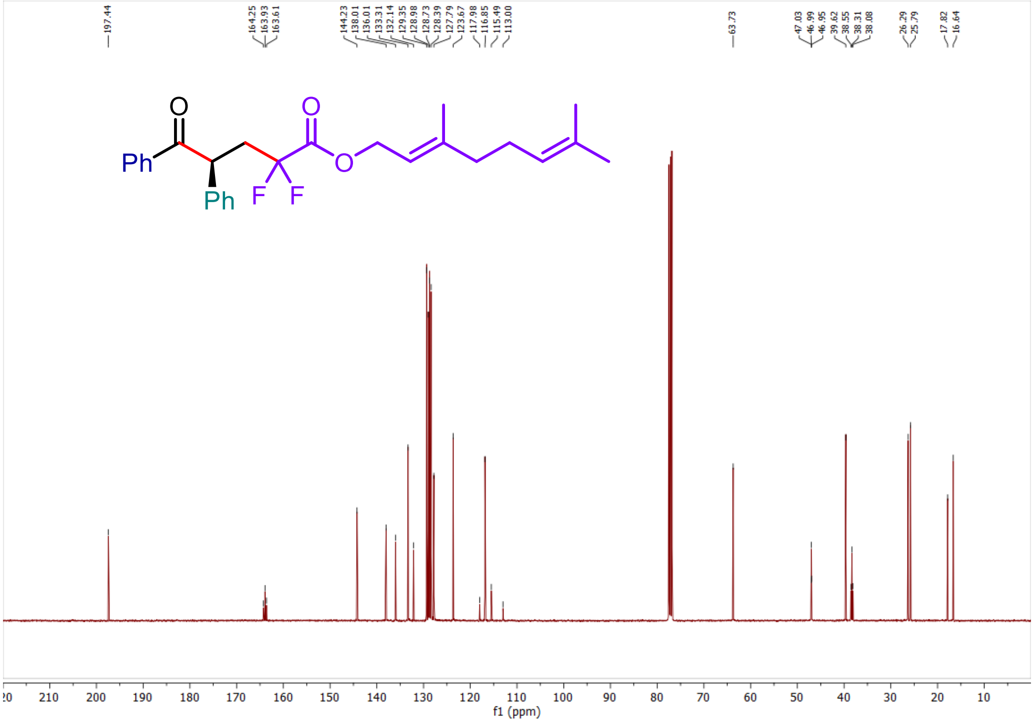

^{19}F NMR (376 MHz, Chloroform-*d*):

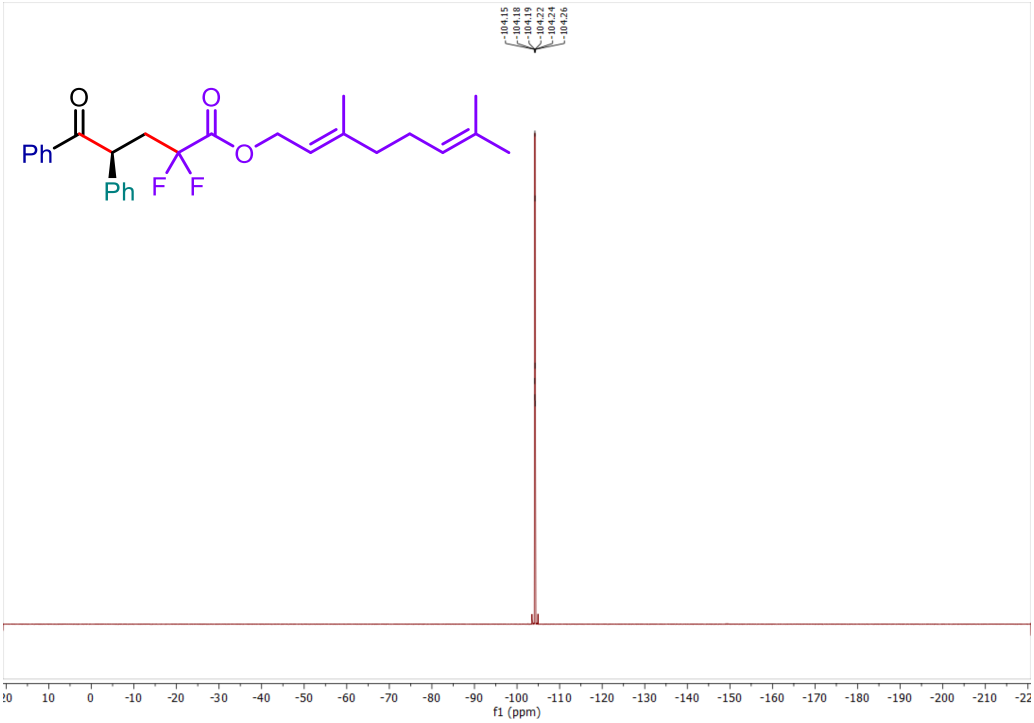

Ethyl (*S*)-5-(4-((S)-5-ethoxy-4,4-difluoro-2-(4-methoxyphenyl)-5-oxopentanoyl)phenyl)-2,2-difluoro-5-oxo-4-phenylpentanoate (11a)

^{1}H NMR (800 MHz, Chloroform-*d*):

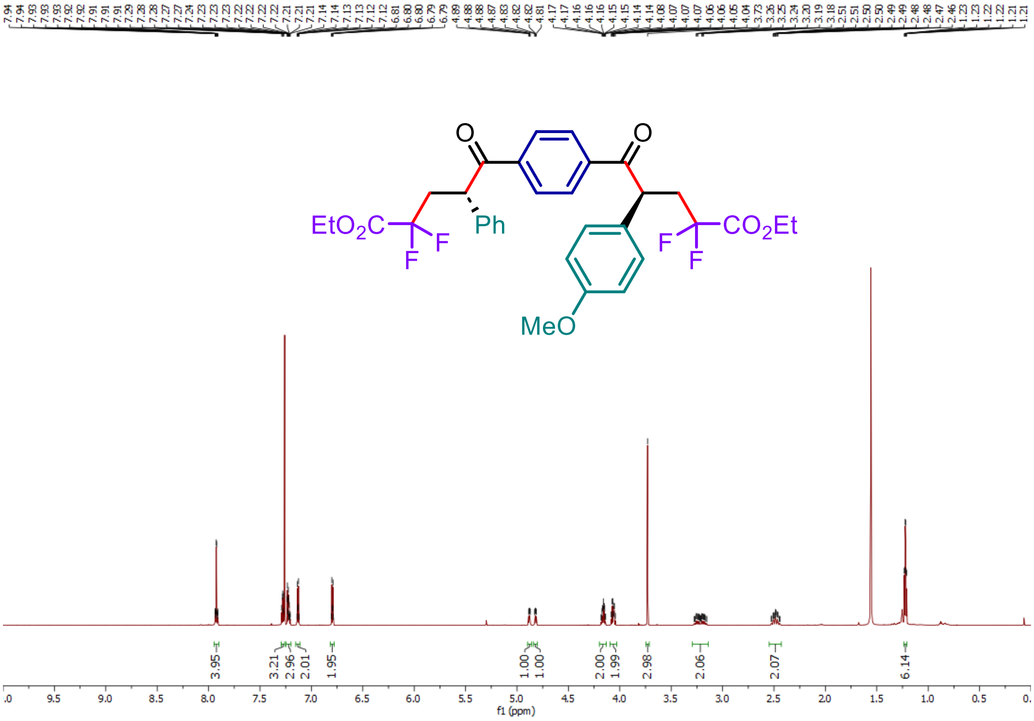

^{13}C NMR (201 MHz, Chloroform-*d*):

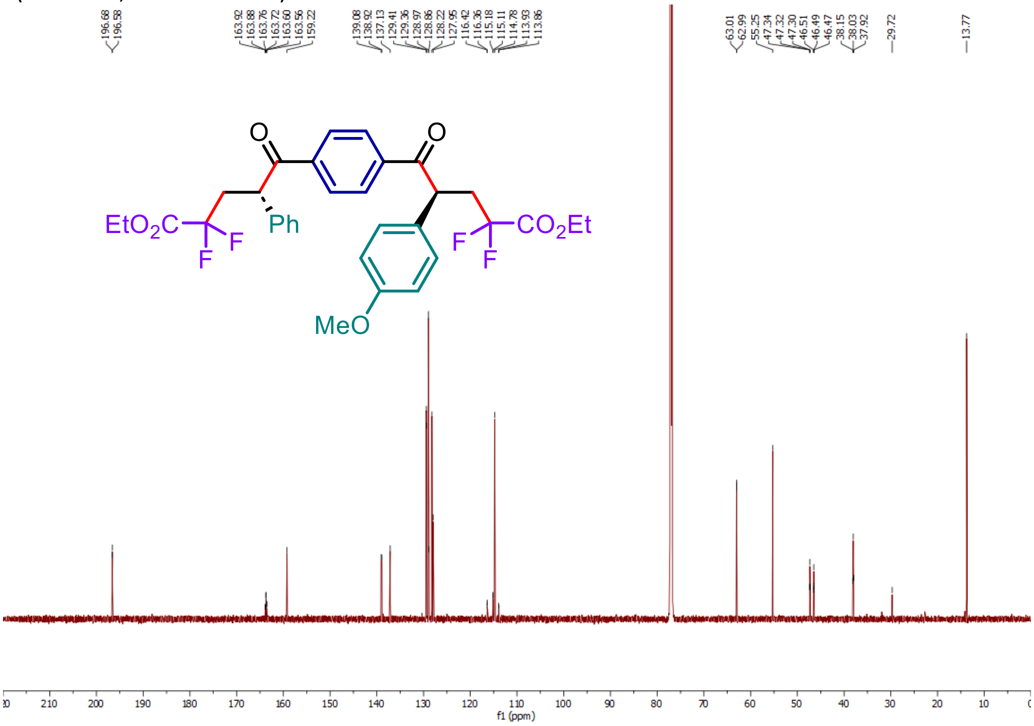

^{19}F NMR (376 MHz, Chloroform-*d*):

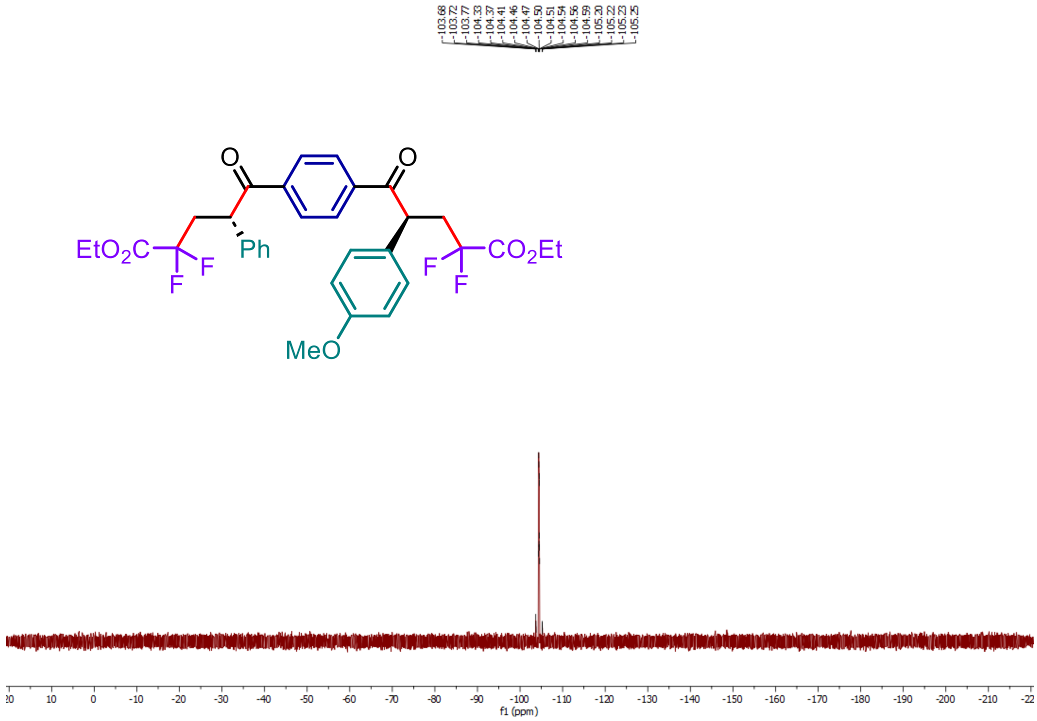

Diethyl 5,5'-(1,4-phenylene)(4*S*,4'*S*)-bis(2,2-difluoro-5-oxo-4-phenylpentanoate) (11b)

^{1}H NMR (400 MHz, Chloroform-*d*):

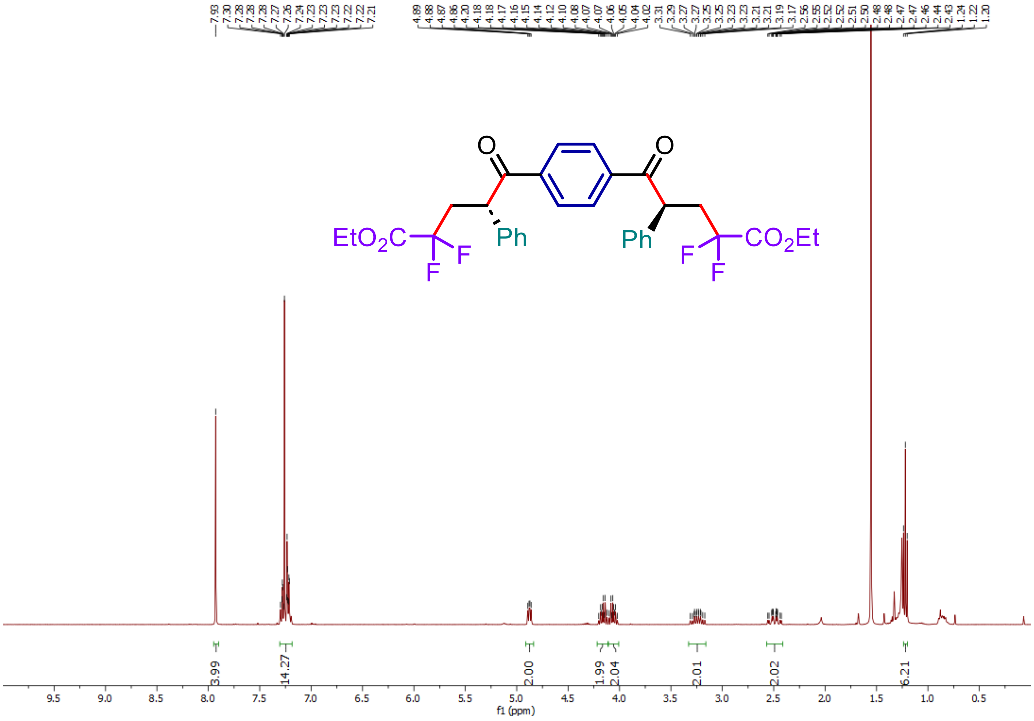

^{13}C NMR (101 MHz, Chloroform-*d*):

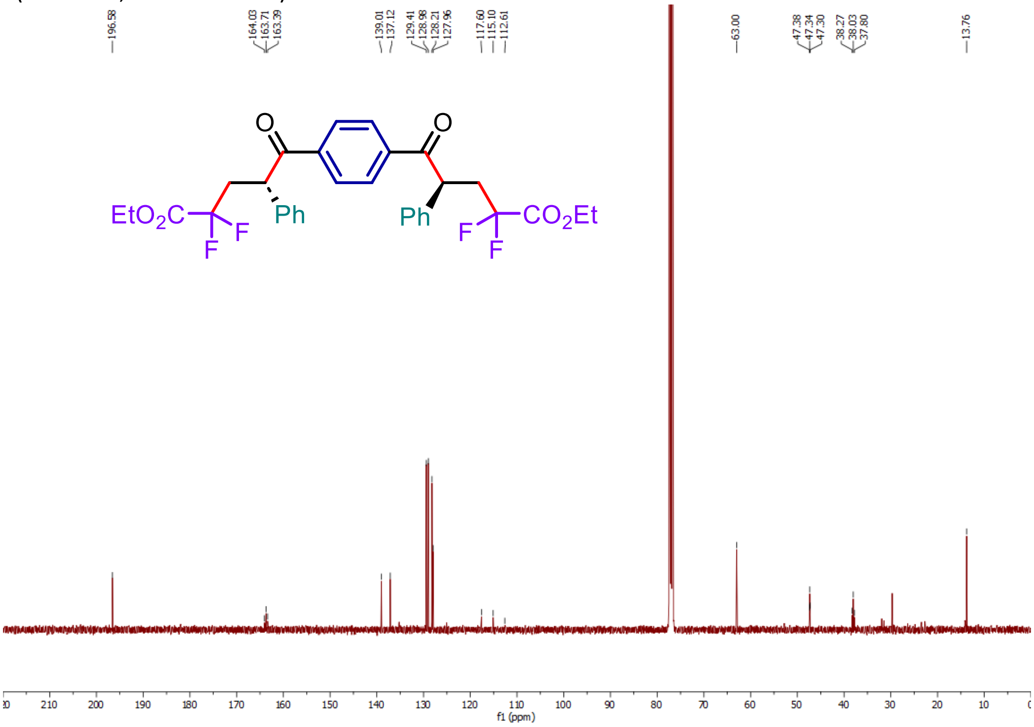

^{19}F NMR (376 MHz, Chloroform-*d*)

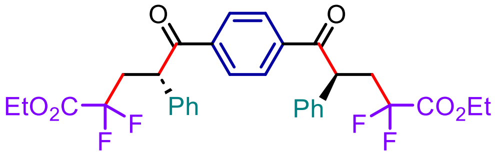

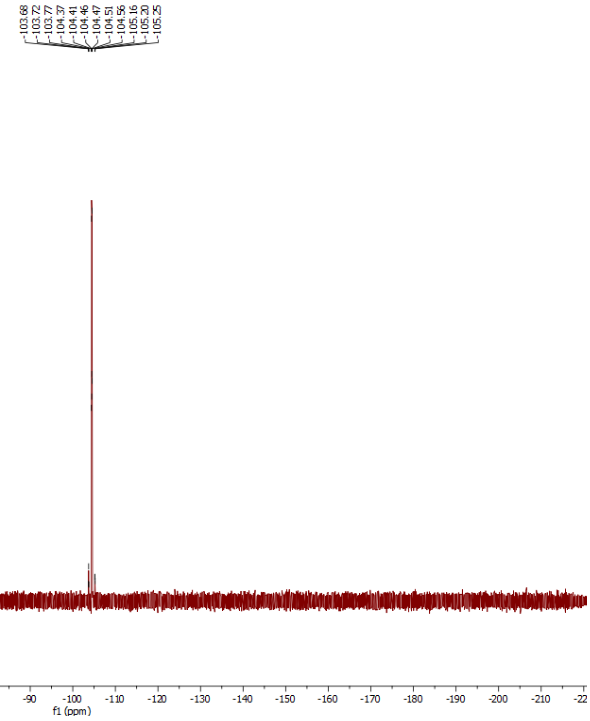

Ethyl (*S*)-4-(4-((*S*)-5-ethoxy-4,4-difluoro-1,5-dioxo-1-phenylpentan-2-yl)phenyl)-2,2-difluoro-5-(4-methoxyphenyl)-5-oxopentanoate (13a)

^{1}H NMR (400 MHz, Chloroform-*d*):

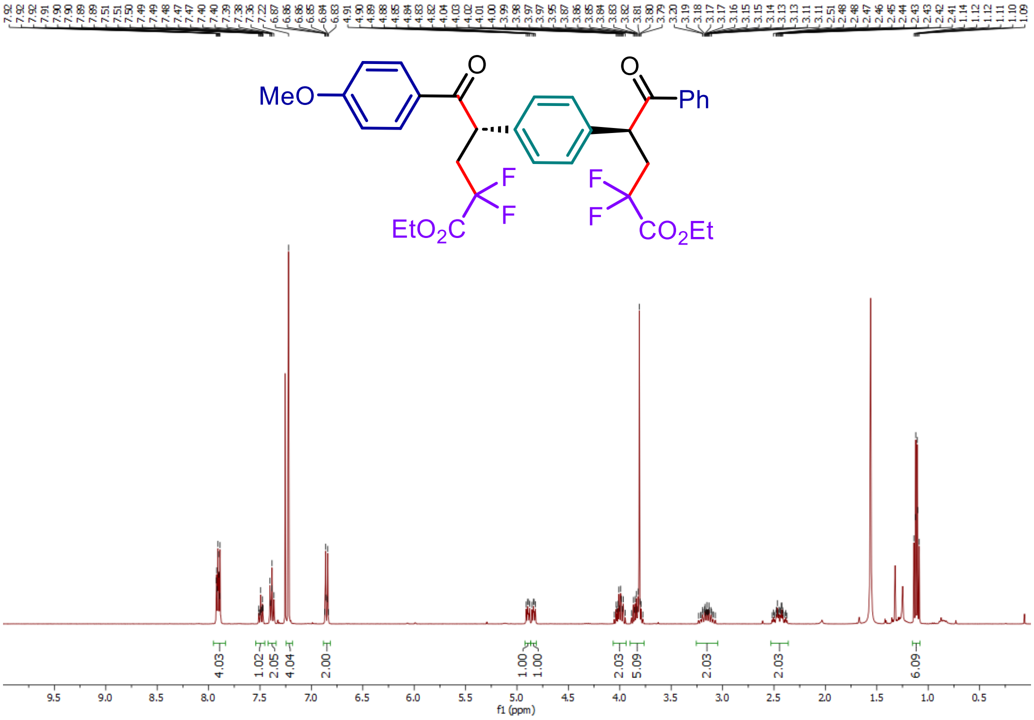

^{13}C NMR (101 MHz, Chloroform-*d*):

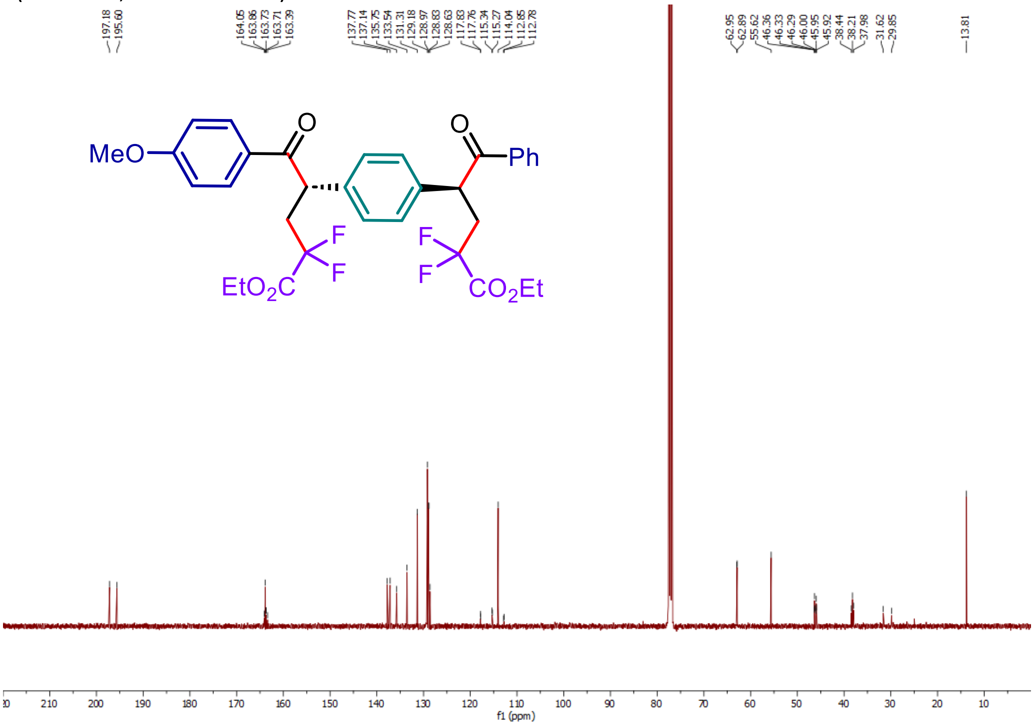

^{19}F NMR (377 MHz, Chloroform-*d*):

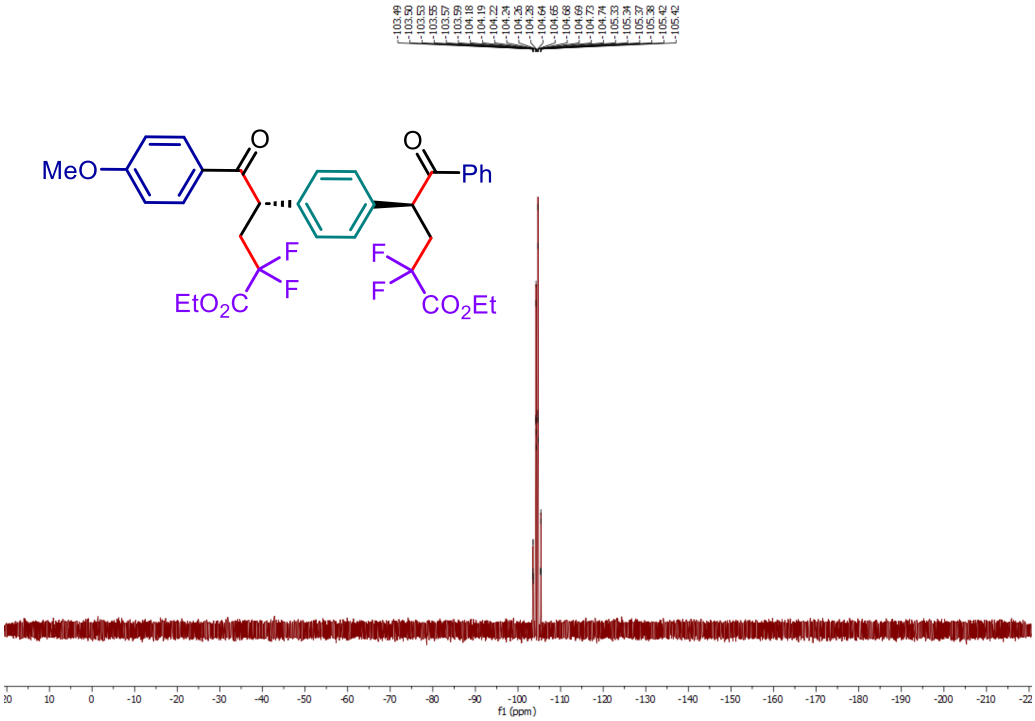

Diethyl 4,4'-(1,4-phenylene)(4*S*,4'*S*)-bis(2,2-difluoro-5-oxo-5-phenylpentanoate) (13b)

[1]**H NMR** (400 MHz, Chloroform-*d*):

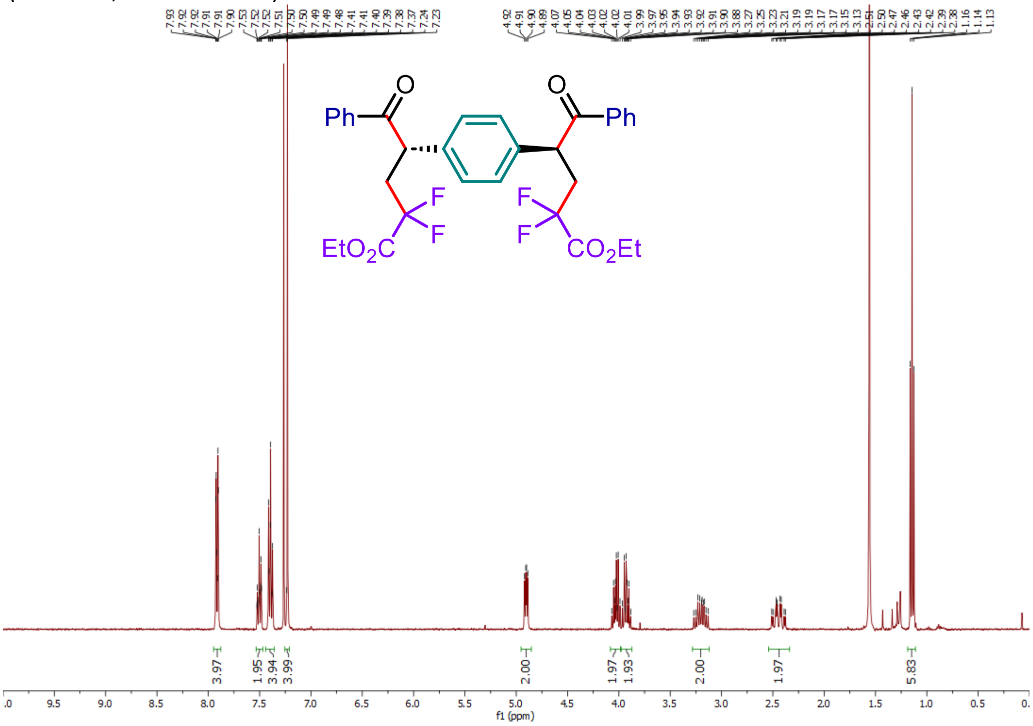

[13]**C NMR** (101 MHz, Chloroform-*d*):

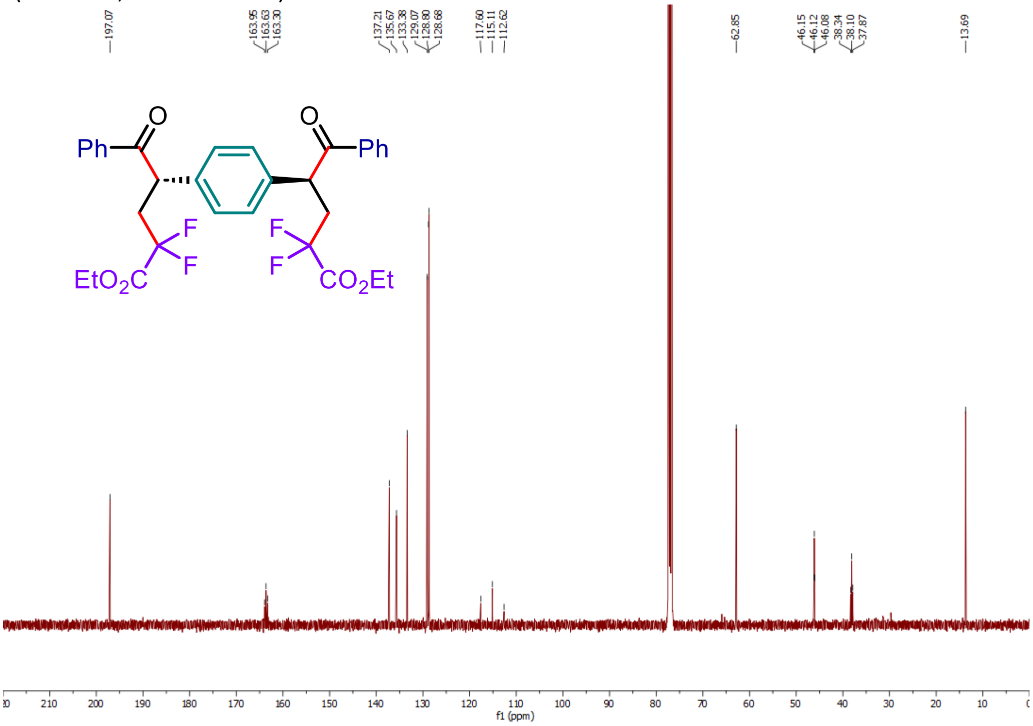

^{19}F NMR (376 MHz, Chloroform-*d*):-

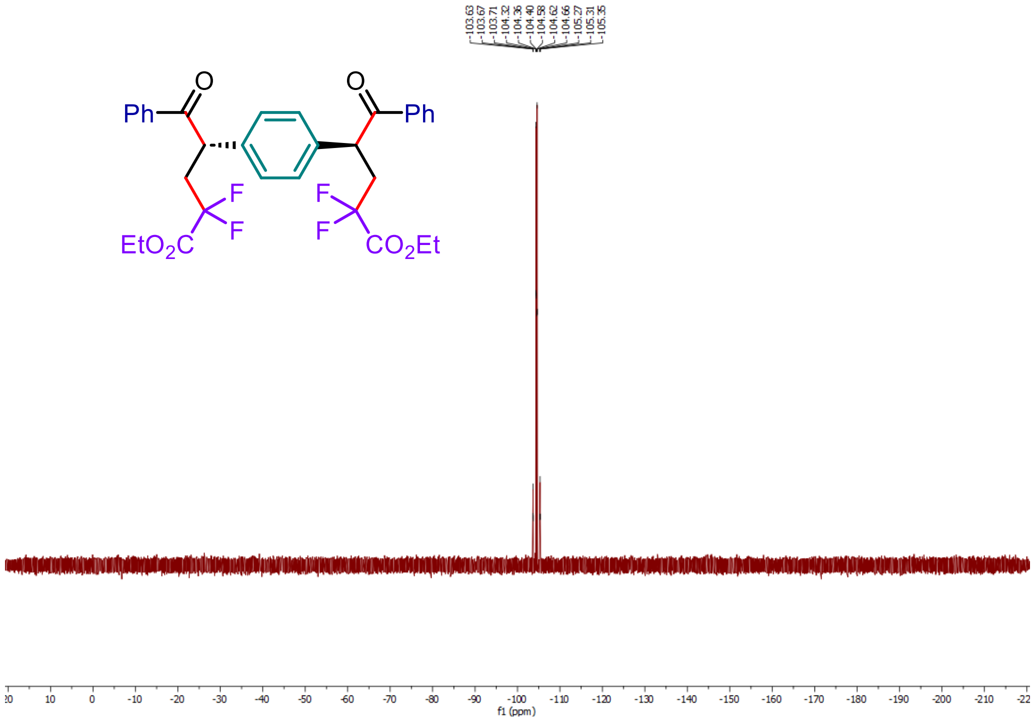

(1*S*,2*S*)-4,4-difluoro-1-(4-iodophenyl)-2-phenylpentane-1,5-diol (14)

^{1}H NMR (400 MHz, Chloroform-*d*):

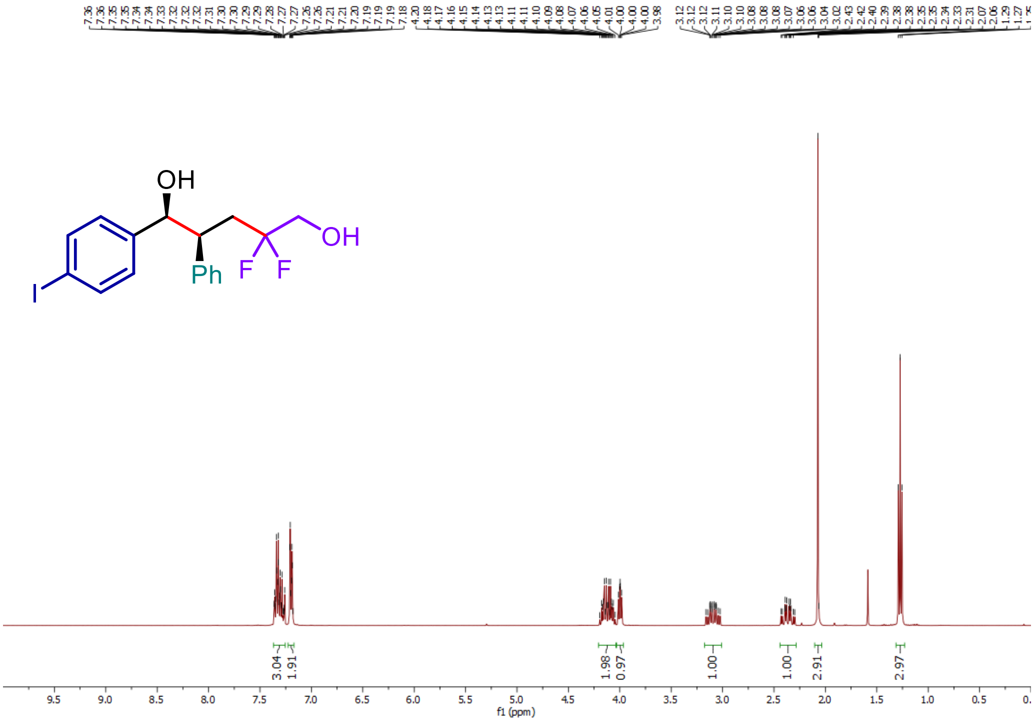

^{13}C NMR (101 MHz, Chloroform-*d*):

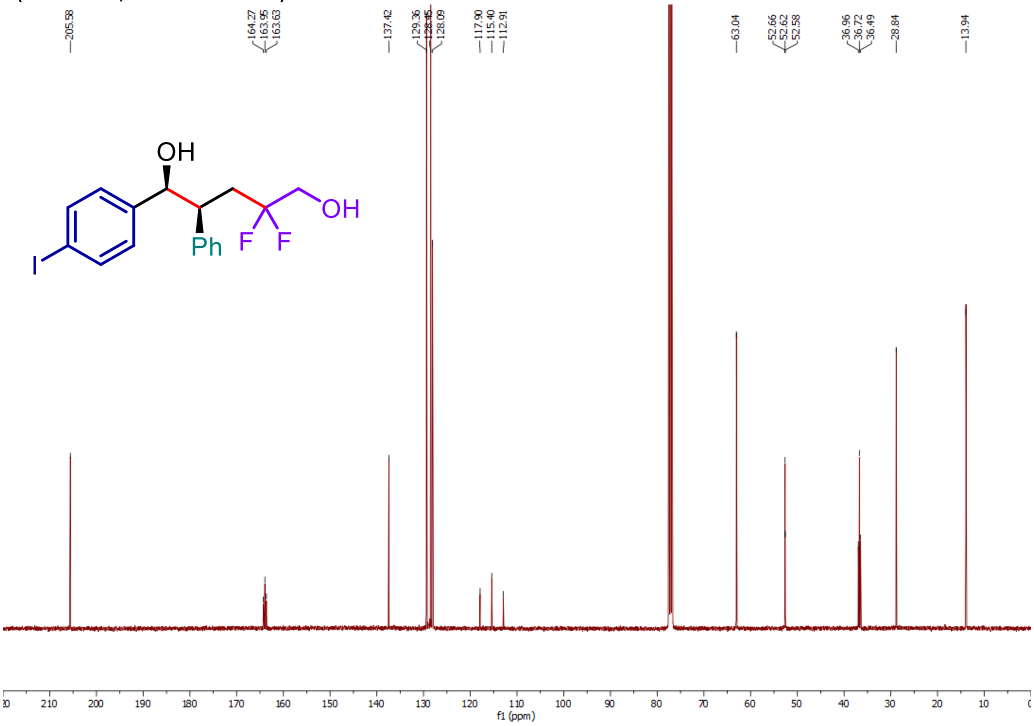

^{19}F NMR (376 MHz, Chloroform-*d*):

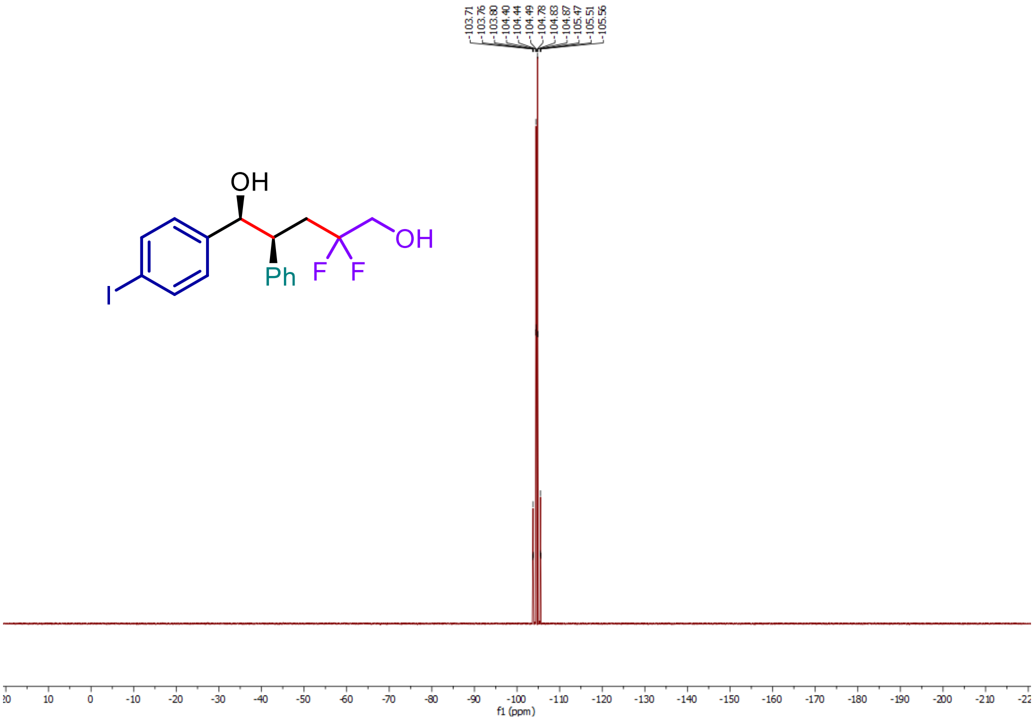

(*S*)-4,4-Dimethyl-1,2-diphenylpentan-1-one (27)

^{1}H NMR (400 MHz, Chloroform-*d*):

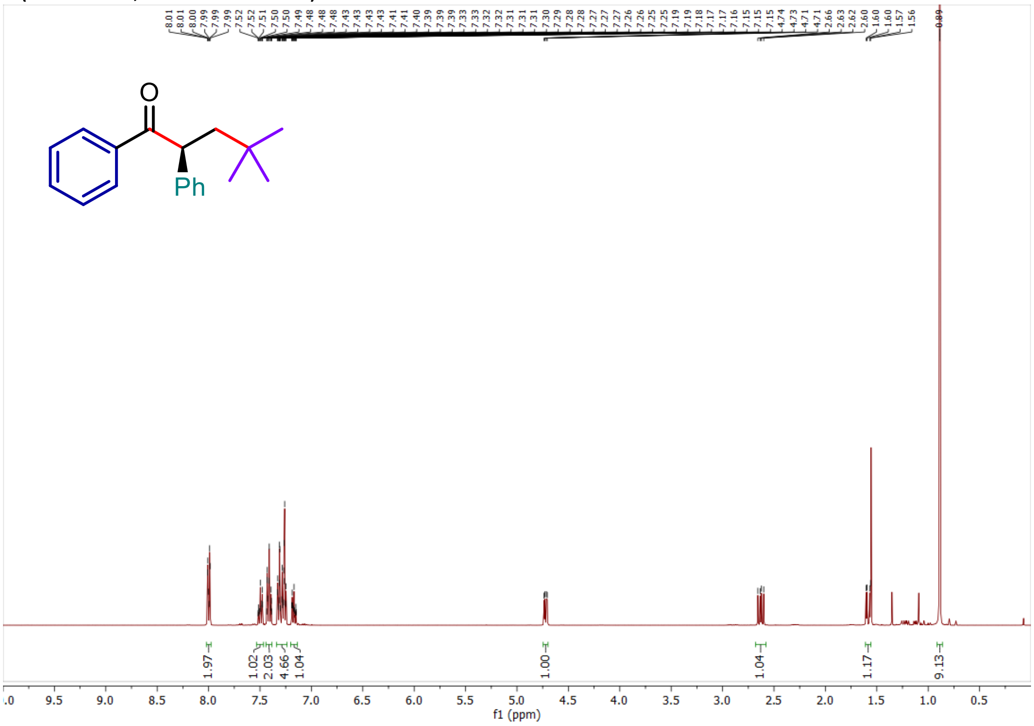

^{13}C NMR (101 MHz, Chloroform-*d*):

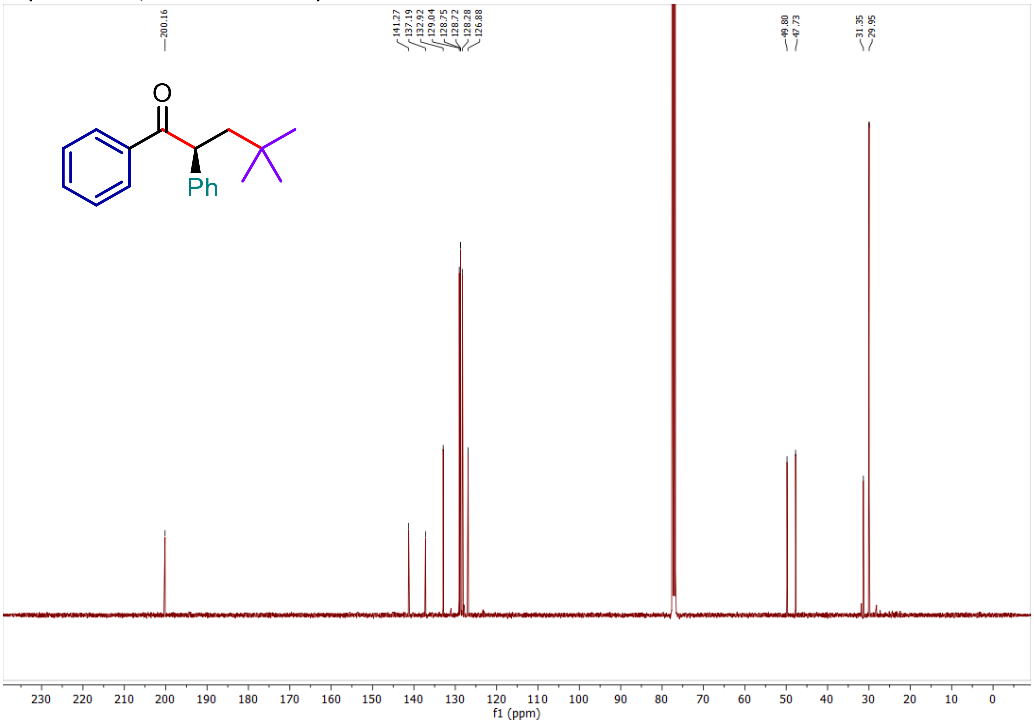

(*S*)-1,2-Diphenylpropan-1-one (28)

^{1}H NMR (400 MHz, Chloroform-*d*):

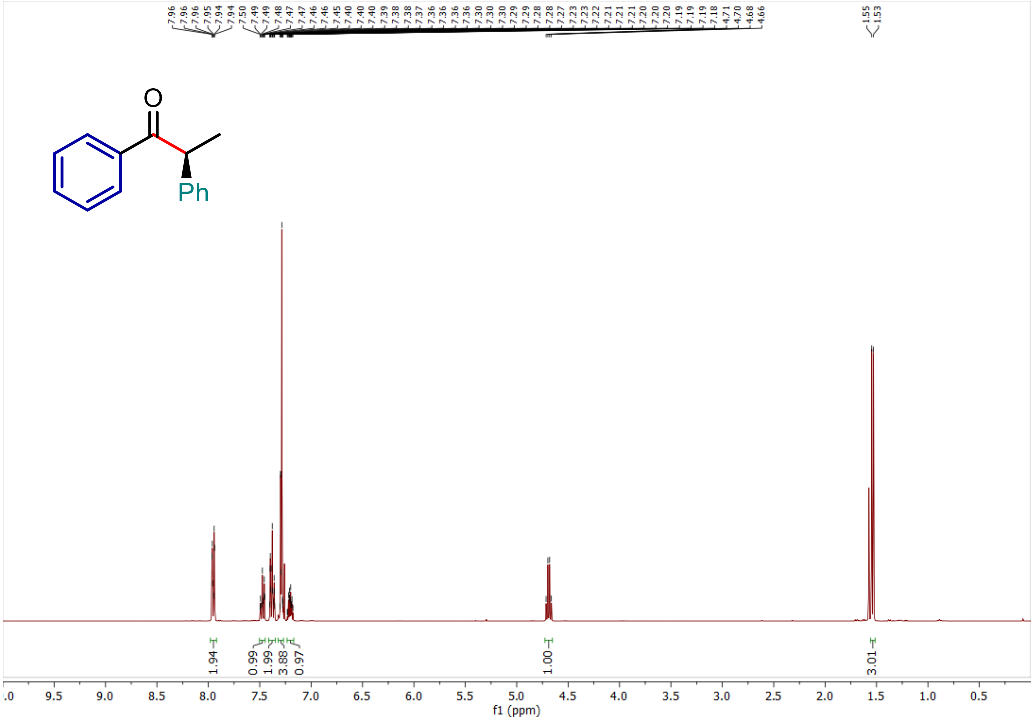

^{13}C NMR (101 MHz, Chloroform-*d*):

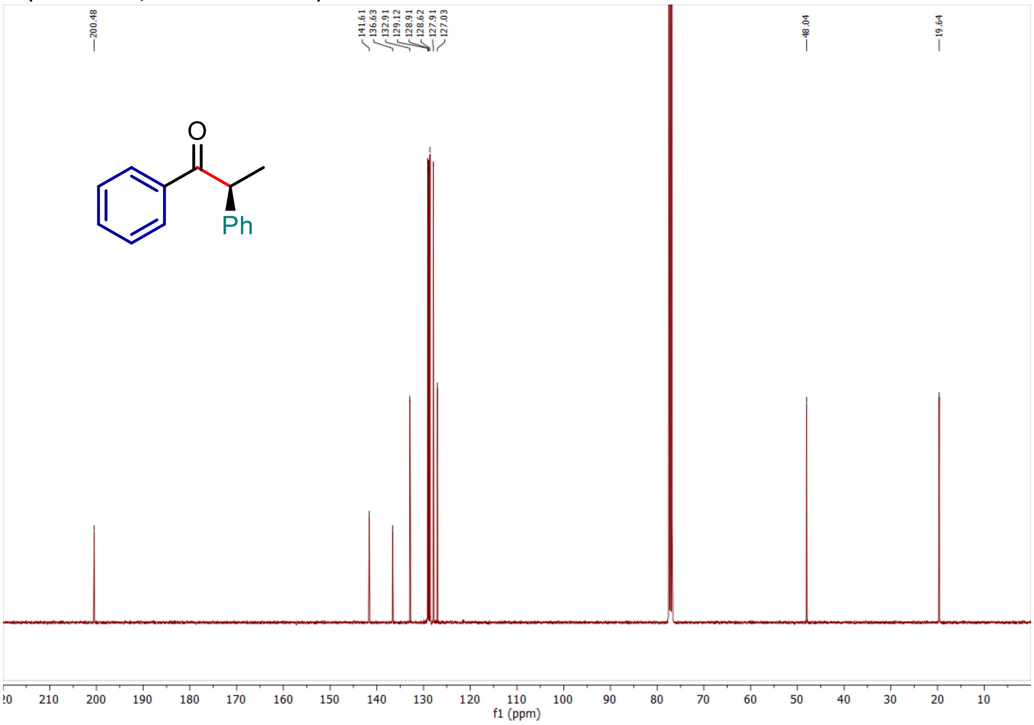

Methyl (*S*)-2-benzyl-3-oxo-3-phenylpropanoate (29)

^{1}H NMR (400 MHz, Chloroform-*d*):

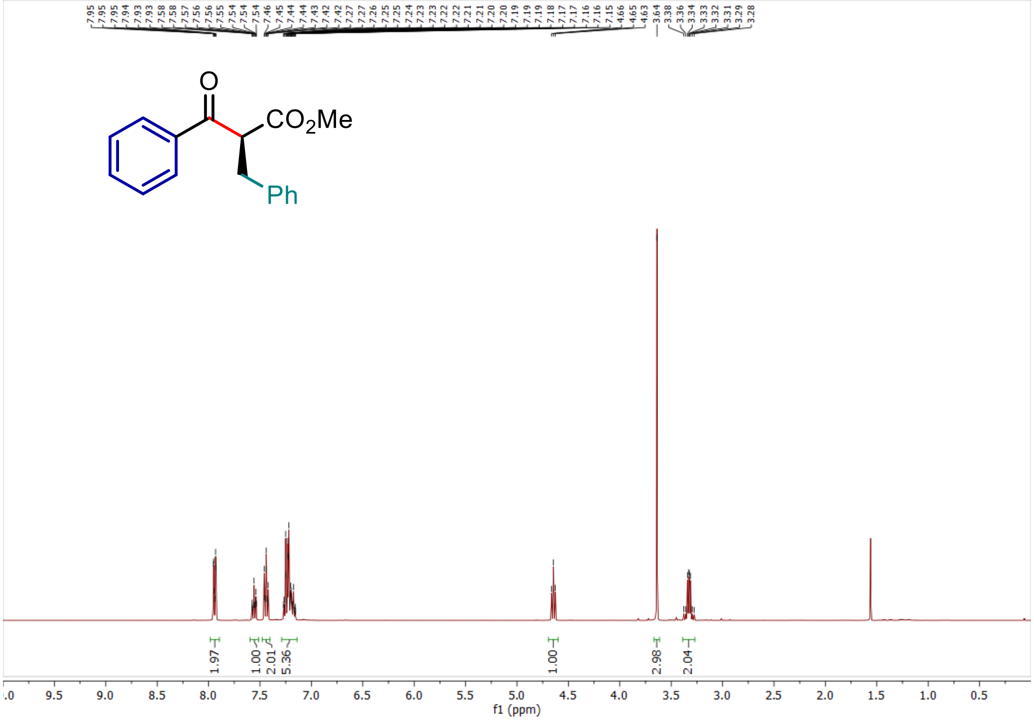

^{13}C NMR (101 MHz, Chloroform-*d*):

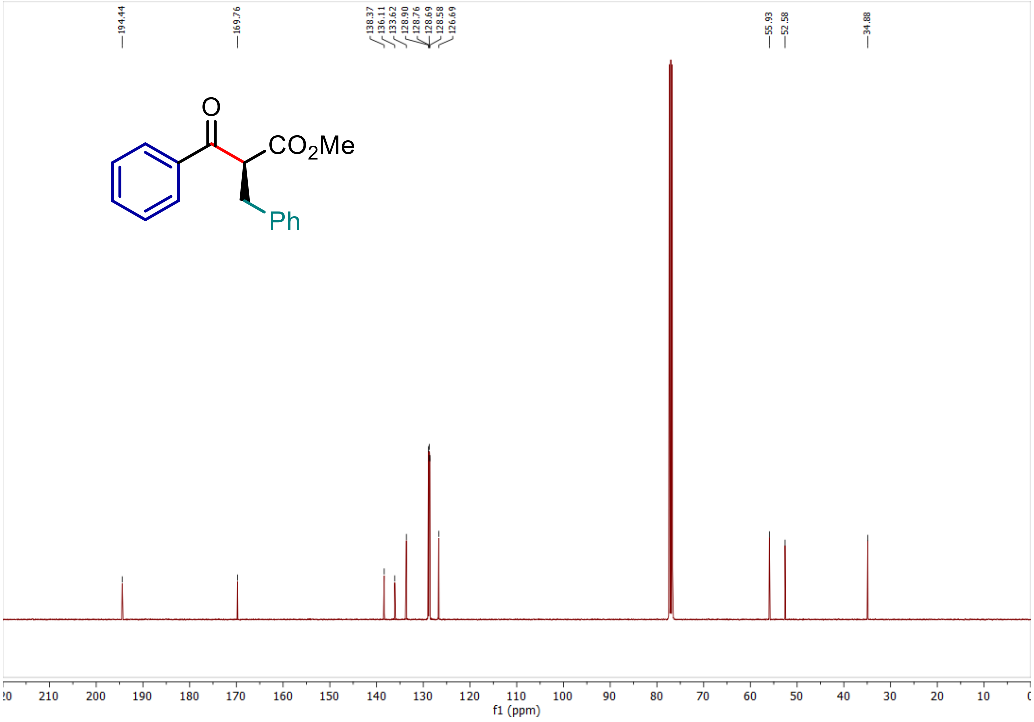

Supplement: Supplementary file 1 — ja4c11947_si_001.pdf [file ja4c11947_si_001.pdf]
